# The neuroendocrine phenotype, genomic profile and therapeutic sensitivity of GEPNET cell lines

**DOI:** 10.1530/ERC-17-0445

**Published:** 2018-01-15

**Authors:** Tobias Hofving, Yvonne Arvidsson, Bilal Almobarak, Linda Inge, Roswitha Pfragner, Marta Persson, Göran Stenman, Erik Kristiansson, Viktor Johanson, Ola Nilsson

**Affiliations:** 1 Sahlgrenska Cancer CenterDepartment of Pathology and Genetics, Institute of Biomedicine, Sahlgrenska Academy at the University of Gothenburg, Gothenburg, Sweden; 2 Institute of Pathophysiology and ImmunologyCenter for Molecular Medicine, Medical University of Graz, Graz, Austria; 3 Department of Mathematical SciencesChalmers University of Technology, Gothenburg, Sweden; 4 Department of SurgeryInstitute of Clinical Sciences, Sahlgrenska Academy at the University of Gothenburg, Gothenburg, Sweden

**Keywords:** neuroendocrine tumour, GEPNET, immunophenotyping, copy number alterations, exome sequencing, inhibitor screening, cell lines, vorinostat, trametinib

## Abstract

Experimental models of neuroendocrine tumour disease are scarce, and no comprehensive characterisation of existing gastroenteropancreatic neuroendocrine tumour (GEPNET) cell lines has been reported. In this study, we aimed to define the molecular characteristics and therapeutic sensitivity of these cell lines. We therefore performed immunophenotyping, copy number profiling, whole-exome sequencing and a large-scale inhibitor screening of seven GEPNET cell lines. Four cell lines, GOT1, P-STS, BON-1 and QGP-1, displayed a neuroendocrine phenotype while three others, KRJ-I, L-STS and H-STS, did not. Instead, these three cell lines were identified as lymphoblastoid. Characterisation of remaining authentic GEPNET cell lines by copy number profiling showed that GOT1, among other chromosomal alterations, harboured losses on chromosome 18 encompassing the *SMAD4* gene, while P-STS had a loss on 11q. BON-1 had a homozygous loss of *CDKN2A* and *CDKN2B*, and QGP-1 harboured amplifications of *MDM2* and *HMGA2*. Whole-exome sequencing revealed both disease-characteristic mutations (e.g. *ATRX* mutation in QGP-1) and, for patient tumours, rare genetic events (e.g. *TP53* mutation in P-STS, BON-1 and QGP-1). A large-scale inhibitor screening showed that cell lines from pancreatic NETs to a greater extent, when compared to small intestinal NETs, were sensitive to inhibitors of MEK. Similarly, neuroendocrine NET cells originating from the small intestine were considerably more sensitive to a group of HDAC inhibitors. Taken together, our results provide a comprehensive characterisation of GEPNET cell lines, demonstrate their relevance as neuroendocrine tumour models and explore their therapeutic sensitivity to a broad range of inhibitors.

## Introduction

Patient tumour-derived cell lines, as models of tumour disease, have been widely used for studying the molecular mechanisms of tumours and their response to therapy. However, cell lines do not perfectly reflect their tumour of origin and in terms of genomic alterations, protein expression and therapeutic sensitivity, they can differ substantially (Stein *et al*. 2004, Sandberg & Ernberg 2005, Ertel *et al*. 2006, Gillet *et al*. 2011, Domcke *et al*. 2013).

The establishment of cell lines from human neuroendocrine tumours (NETs) of the gastrointestinal tract and pancreas (GEPNETs) has proved to be difficult. This has been attributed, in part, to the low proliferation rate of GEPNETs (Modlin *et al*. 2008). Despite these challenges, several cell lines have been established from small intestinal NETs (SINETs). The first cell line derived from a human SINET was the KRJ-I cell line, reported in 1996 (Pfragner *et al*. 1996). KRJ-I was established from a primary tumour and has been used to characterise the molecular mechanisms and therapeutic sensitivity of SINET disease (Modlin *et al*. 2006, Kidd *et al*. 2007, 2008). The GOT1 cell line was established in our laboratory in 2001 from a patient with metastatic SINET disease (Kölby *et al*. 2001). GOT1 cells were derived from a liver metastasis and expressed neuroendocrine markers, including somatostatin receptor type 2 (SSTR2). This cell line has been used as a model for optimisation of SSTR2-mediated peptide receptor radionuclide therapy (PRRT) (Kölby *et al*. 2005, Bernhardt *et al*. 2007, Forssell-Aronsson *et al*. 2013, Dalmo *et al*. 2017, Spetz *et al*. 2017). More recently, in 2009, three cell lines were established from a patient with metastatic SINET disease (Pfragner *et al*. 2009). The P-STS cell line was derived from the patient’s primary tumour, L-STS from a lymph node metastasis and H-STS from a liver metastasis. A basic characterisation of these cell lines has been reported, and P-STS has been used to study the secretory response of SINETs (Rinner *et al*. 2012, Pfanzagl *et al*. 2017).

The generation of cell lines from human pancreatic NETs (PanNETs) has also been proven difficult, and only few cell lines are available. Among the most frequently used PanNET cell lines are QGP-1 and BON-1. QGP-1 was established in 1980 from a somatostatin-producing islet cell carcinoma (Kaku *et al*. 1980, Iguchi *et al*. 1990) and it has been used to study the molecular mechanisms that regulate tumour cell proliferation (Doihara *et al*. 2009, Valentino *et al*. 2014). BON-1 was established in 1991 from a lymph node metastasis of a PanNET patient (Evers *et al*. 1991) and has been used as a model for studying the molecular mechanisms of PanNETs and their sensitivity to therapy. Albeit so far lesser used, the establishment of additional GEPNET cell lines has been reported, e.g. CM, LCC-18 and CNDT2.5 (Gueli *et al*. 1987, Lundqvist *et al*. 1991, Van Buren *et al*. 2007). However, the authenticity of CNDT2.5 has been questioned (Ellis *et al*. 2010). There are also GEPNET cell lines derived from transgenic murine rodents, e.g. STC-1 (intestinal NET from SV40-expressing transgenic mouse), βTC and MIN6 (insulinomas from SV40-expressing transgenic mouse), RIN and INS-1 (X-ray induced insulinomas from NEDH rat) (Gazdar *et al*. 1980, Rindi *et al*. 1990, Efrat *et al*. 1991, Asfari *et al*. 1992, Ishihara *et al*. 1993).

The current status can be summarised as follows. Authentic GEPNET cell lines are rare, their genomic and mutational characteristics largely unknown and comprehensive information regarding their therapeutic sensitivity is lacking. Recently, efforts have been made to characterise PanNET-derived cell lines BON-1 and QGP-1 by exome sequencing and genome-wide copy number analysis. These studies have raised questions regarding their relevance as models due to the absence of PanNET-associated mutations (Boora *et al*. 2015, Vandamme *et al*. 2015). There have been no corresponding efforts to characterise NET cell lines derived from SINETs. The aim of the present study was to re-evaluate the authenticity of GEPNET cell lines and to define their genomic profile and their therapeutic sensitivity. In this paper, we report the immunophenotyping, genome-wide copy number profiling, whole-exome sequencing and comprehensive inhibitor screening of seven GEPNET cell lines. We confirmed the neuroendocrine phenotype of GOT1, P-STS, BON-1 and QGP-1 and investigated their genomic profile. The inhibitor screening established the therapeutic sensitivity profiles of the cell lines and predicted the cell tumour type-specific efficacy of MEKi and HDACi.

## Materials and methods

### Cell lines, primary cell cultures and cell culturing

The GOT1 cell line was established from a liver metastasis of a midgut carcinoid from a female patient (Kölby *et al*. 2001). GOT1 was cultured in RPMI-1640 supplemented with 10% foetal bovine serum (FBS), 5 μg/mL insulin and 5 μg/mL transferrin. KRJ-I, P-STS, L-STS and H-STS were all gifts from Prof. R Pfragner and cultured in M199:Ham’s F12 (1:1) supplemented with 10% FBS. KRJ-I was established from a male patient with a multifocal carcinoid of the small intestine. P-STS (primary tumour), L-STS (lymph node metastasis) and H-STS (liver metastasis) were all established from the same male patient with metastatic carcinoid of the terminal ileum. The PanNET cell line BON-1, a gift from Prof. B Wiedenmann, was established from the lymph node of a carcinoid tumour of the pancreas in a male patient and cultured in DMEM:Ham’s F12 (1:1) with 10% FBS. QGP-1, which has been isolated from a pancreatic carcinoma of a male patient, was acquired from Japanese Collection of Research Bioresources Cell Bank (JCRB) and maintained in RPMI-1640 supplemented with 10% FBS. MCF10A, BJ and HUV-EC-C were acquired from ATCC. MCF10A (human mammary gland epithelium) was maintained in DMEM:Ham’s F12 (1:1) supplemented with 5% horse serum, 10 µg/mL insulin, 20 ng/mL epidermal growth factor (EGF), 0.5 µg/mL hydrocortisone and 0.1 µg/mL cholera toxin. BJ (human foreskin fibroblasts) was maintained in Eagle’s minimum essential medium supplemented with 10% FBS. HUV-EC-C (human vascular endothelium) was maintained in F12K medium supplemented with 10% FBS, 100 µg/mL heparin and 50 µg/mL endothelial cell growth supplement. The medium of all cell lines also contained 200 IU/mL penicillin and 200 μg/mL streptomycin. For doubling time experiments, cells were seeded onto 25 cm^2^ tissue cell culture flasks. At 24, 48, 72, 96 and 120 h, flasks (*n* = 2) were trypsinised and counted using Countess cell counter (Invitrogen).

Primary cell cultures of small intestinal and pancreatic NETs were generated from biopsies collected at the time of surgery, as previously described (Arvidsson *et al*. 2010). Both PanNET cultures (PanNET patient 1 and 2) were derived from primary tumours, ‘SINET patient 1’ from a lymph node metastasis and ‘SINET patient 2’ from a liver metastasis. The cells were kept in RPMI-1640 supplemented with 4% FBS, 200 IU/mL penicillin and 200 μg/mL streptomycin. For the characterisation and inhibitor sensitivity experiments, only first-passage primary cell cultures were used. All cell lines and primary cell cultures were incubated at 37°C in a humidified incubator with an atmosphere of 5% CO_2_.

### Analysis of STR, Epstein-Barr virus and *Mycoplasma*

DNA was isolated from cell lines at the same passage as used for the inhibitor screening using the DNeasy Blood & Tissue Kit (Qiagen). DNA from cell lines, and from GOT1 patient tumour tissue, was subjected to STR analysis at a DANAK/ILAC DS/EN ISO 15189:2008 accredited laboratory (IdentiCell, Department of Molecular Medicine (MOMA) at Aarhus University Hospital, Denmark) (Supplementary Fig. 1, see section on supplementary data given at the end of this article). Cell lines were regularly tested for *Mycoplasma* species by PCR as described in the study by van Kuppeveld *et al*. (1994) at a Swedac SS-EN ISO 15189 accredited laboratory (Bacteriological laboratory, Sahlgrenska University Hospital, Gothenburg, Sweden). DNA from GEPNETs and lymphoblastoid cell lines was subjected to EBV DNA quantification using *artus* EBV PCR Kit (Qiagen). The PCR was performed using a 7500 Fast-Real-time PCR system (Applied Biosystems).

### Cell blocks and immunohistochemistry

Cell lines and primary cell cultures in exponential growth phase were detached and fixed in 4% buffered formaldehyde for 1 h followed by methanol fixation. The paraffin blocks were created using a Cellient automated cell block system (HOLOGIC). Sectioning and staining were carried out as previously described (Andersson *et al*. 2016). The following primary antibodies were used: anti-chromogranin A (PHE5; Chemicon; diluted 1:1000), anti-synaptophysin (SY38; Dako; diluted 1:200), anti-NCAM (ERIC1; Santa Cruz; diluted 1:10), anti-NSE (BBS/NC/VI-H14; Dako; ready-to-use), anti-PGP9.5 (Z5116; Dako; diluted 1:400), anti-CD57 (HNK-1; BD Biosciences; diluted 1:40), anti-VMAT1 (C-19; Santa Cruz; diluted 1:1000), anti-5HT (H209; Dako; diluted 1:10), anti-cytokeratin 8/18 (5D3; Leica Biosystems; diluted 1:2000), anti-pan-cytokeratin (AE1/AE3; Leica; ready-to-use), anti-SSTR2A (UMB-1; Abcam; diluted 1:100), anti-Ki67 (MIB-1; Dako; ready-to-use), anti-CD45 (2B11+PD7/26; Dako; diluted 1:200) and anti-CD20 (L26; Dako; ready-to-use). Each staining was scored by a board-certified pathologist (O N) into four categories; ‘0’ (<1% weakly stained cells), ‘+’ (<50% weakly stained cells), ‘++’ (moderate staining in >50% of cells) and ‘+++’ (strong staining in >50% of cells). A biopsy of a SINET served as a positive control (‘+++’). For Ki67, manual counting was performed on printed images and the percentage of labelled tumour cell nuclei was calculated (Reid *et al*. 2015).

### Confocal laser scanning microscopy

Adherent cells (GOT1, P-STS, QGP-1 and BON-1) were grown on µ-slide 8-well chamber slides (ibidi, Martinsried, Germany) for 3 days and fixed in 4% buffered formaldehyde in PBS. Cells growing in suspensions (KRJ-I, L-STS, and H-STS) were spun onto microscope slides with a Cytospin 2 cytocentrifuge (Shandon, UK) before fixation. The slides were incubated with primary antibodies for 1 h at room temperature in 1% BSA and 0.2% Triton X-100 in PBS and for 1 h with secondary antibodies conjugated to Alexa Fluor 594 (rabbit anti-mouse, cat. no. A11062 or goat anti-rabbit, cat. no. A11037; ThermoFisher) or to FITC (rabbit anti-goat, cat. no. F0250; Dako). All experiments included negative controls wherein the primary antibody was omitted. The fluorescent cells were analysed using a Zeiss LSM 700 confocal microscope and Zen black software was supplied by the manufacturer. Images were captured using a Zeiss LSM 700 inverted microscope with a 63× oil immersion objective.

### Analysis of copy number alterations

DNA from cell lines was extracted as mentioned earlier, and arrayCGH analysis was performed using 4x180K Human SurePrint G3 ISCA CGH + SNP microarrays (Agilent Technologies) as recommended by the manufacturer and as previously described (Barrett *et al*. 2004, Persson *et al*. 2008). Data analysis was carried out using Nexus Copy Number software, v.8.0 (BioDiscovery Inc., El Segundo, CA, USA). The FASST2 segmentation algorithm was used to define non-random regions of copy number alterations (CNAs) across the genome with a significance threshold ranging from *P* = 10^−8^ to *P* = 10^−18^, where the higher stringency threshold was used for arrays with QC >0.2. The log_2_ ratio thresholds for aberration calls of gain and loss were set to 0.2 and −0.2 for arrays with a significance threshold *P*≥**10^−8^, and to 0.3 and −0.3 for arrays with a significance threshold *P*<**10^−8^. Thresholds for amplification and homozygous loss were set to 1.5 and −1.5 in all cases. Sex chromosomes and regions partially or completely covered by a previously reported copy number variation (Database of Genomic Variants; http://dgvbeta.tcag.ca/dgv/app/news?ref=NCBI37/hg19) were excluded from the analysis (Iafrate *et al*. 2004). Each aberration was checked manually to confirm the accuracy of the call.

### Whole-exome sequencing

DNA from cell lines was extracted as described earlier and subjected to whole-exome sequencing at GATC Biotech (Cologne, Germany). FastQC (version 0.11.2) was used to assess the quality of the data. Paired end reads were aligned to the human reference genome (hg19) using a Burrows-Wheeler Aligner (mem version BWA_0.7.13) (Li & Durbin 2009). Samtools (version 1.3.1) was used to sort, index and assess mapping statistics. Picard (version 2.2.4) was used to remove duplicates. The Genome Analysis ToolKit (GATK, version 3.1-1) (McKenna *et al*. 2010) was used for realignment and variant calling. Variant calling was performed following GATK best practices with the tool HaplotypeCaller. In HaplotypeCaller, the following hard filters were applied for quality filtering; for SNPs: QD <2.0, MQ <40.0, FS >60.0, ReadPosRankSum <−8.0, MQRankSum <−12.5 and for indels: QD <2.0, FS >200.0, ReadPosRankSum <−20.0. Called variants that passed the GATK quality filtering were further filtered against 1000 Genomes (Auton *et al*. 2015) to remove variants with a MAF >0.01. Remaining variants were annotated with the knownGene database using the tool ANNOVAR (Wang *et al*. 2010).

### Screening library and inhibitor sensitivity

The screening library consisted of 1224 compounds (Inhibitor library, no. L1100; Selleckchem). The library was prepared by two-step dilution of the compounds from 10 mM to 100 µM in DMSO and batched in 96-well plates. Inhibitors were subjected to a maximum of five freeze-thaw cycles. From frozen stocks, cells were expanded 2–5 passages before being used in screening experiments. Seeding density was adjusted for each cell line so that control cells were approximately 70‒80% confluent at treatment endpoint in 100 µL cell medium/well in black solid-bottom 96-well plates. The plates were incubated for 72 h at 37°C to allow for cell attachment. Each treatment plate included DMSO control wells (*n* = 8), and each experiment included an additional plate with DMSO control wells (*n* = 96), as well as one cell-free control plate. The screening library was diluted 1:50 in 100 µL medium and added to the treatment plates (1:2; end concentration 1 µM). Cell viability was estimated using a fluorescence-based assay to measure the reducing capacity of metabolically active cells (alamarBlue, DAL1100; Life Technologies). All assay plates were incubated for 72 h at 37°C, followed by addition of 1:100 alamarBlue reagent. The plates were further incubated for 6 h at 37°C and were then analysed using a 96-well fluorescence plate reader (Victor^3^ multilabel reader, ex. 560 nm/em. 640 nm). For vorinostat and trametinib dose–response curves, first-passage primary cultures were thawed and seeded onto 96-well plates, allowed to settle for 48 h before treatment with vorinostat or trametinib for 72 h. Each concentration was added in triplicate and plates were analysed as described earlier. Fitting of the curves was done in GraphPad Prism software, v7.02 using inhibitor *vs*. response nonlinear fit with variable slope.

### Statistical analyses

For each cell line, inhibitor fluorescence intensities generated by the cell viability assay were log-transformed and normalized by subtracting the average and dividing by the standard deviation generating a Z-score. Average Z-scores were calculated for the SINET and PanNET cell lines respectively. The difference in inhibitor intensity between SINET and PanNET was then calculated and a corresponding p-score was derived from a fitted normal distribution. Testing for groups of inhibitors was done by comparing their differences to the differences of all other inhibitors using a Wilcoxon–Mann–Whitney test. False discovery rates were estimated using the Benjamini–Hochberg algorithm. Differences in the effect of MEKi and HDACi to SINET, PanNET and non-tumourigenic cell lines were analysed using Wilcoxons signed-rank test. Differences in the response of primary cell cultures to different doses of MEKi and HDACi was analysed using two-sided unpaired Student’s *t*-test for each dose and corrected for multiple testing with Benjamini–Hochberg algorithm.

## Results

### Immunophenotyping confirmed expression of neuroendocrine tumour markers and SSTR2 in four GEPNET cell lines

NETs are defined by their expression of neuroendocrine markers, notably chromogranin A and synaptophysin. The degree of neuroendocrine differentiation of NET cell lines was evaluated by performing immunohistochemical staining on cell blocks. Each staining was scored into four categories, using biopsies from SINETs as positive controls.

**Figure 1:**
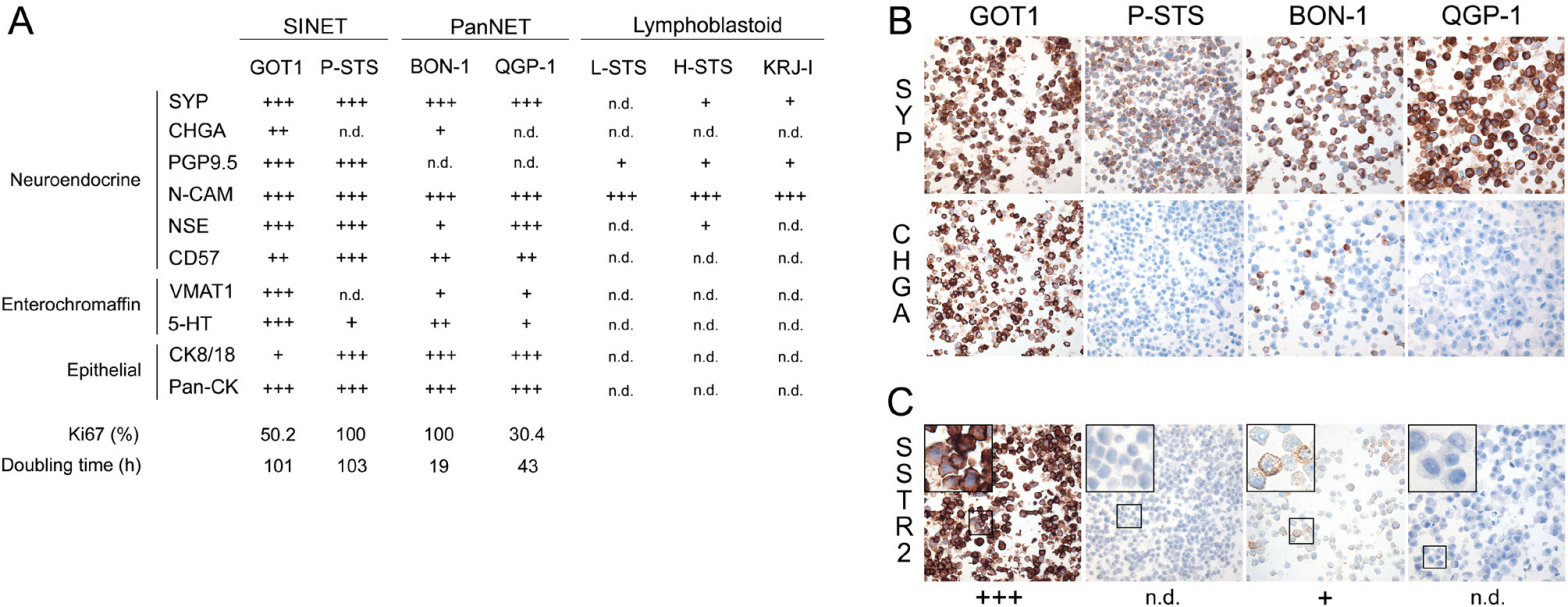
Expression of neuroendocrine markers and somatostatin receptor 2 in GEPNET cell lines. (A) Scoring of protein expression based on immunohistochemical staining of cell blocks. 5-HT was evaluated from confocal laser scanning microscopy. (B and C) Immunohistochemical staining of cell blocks, illustrating expression of the neuroendocrine markers synaptophysin and chromogranin A (B), and clinically relevant SSTR2 (C) in GEPNET cell lines. CHGA, chromogranin A; SYP, synaptophysin; PGP9.5, ubiquitin carboxyl-terminal hydrolase isozyme L1; N-CAM, neural cell adhesion molecule 1; NSE, gamma-enolase; 5-HT, serotonin; VMAT1, chromaffin granule amine transporter 1; cytokeratin 8/18, keratin type II cytoskeletal 8/type I cytoskeletal 18; pan-CK, pan-cytokeratin; SSTR2, somatostatin receptor type 2; n.d., not detected.

We found strong staining for synaptophysin in GOT1, P-STS, QGP-1 and BON-1, while staining for chromogranin A was moderate in GOT1, weak in BON-1 and absent in P-STS and QGP-1 (Fig. 1A and B). In addition, PGP9.5 staining was strong in SINET cell lines and negative in PanNET cell lines. NCAM staining was strong in all investigated cell lines. Neuron-specific enolase (NSE) showed strong staining in GOT1, P-STS and QGP-1, but was weak in BON-1. P-STS had strong staining for CD57, while GOT1, BON-1 and QGP-1 had moderate levels.

**Figure 2:**
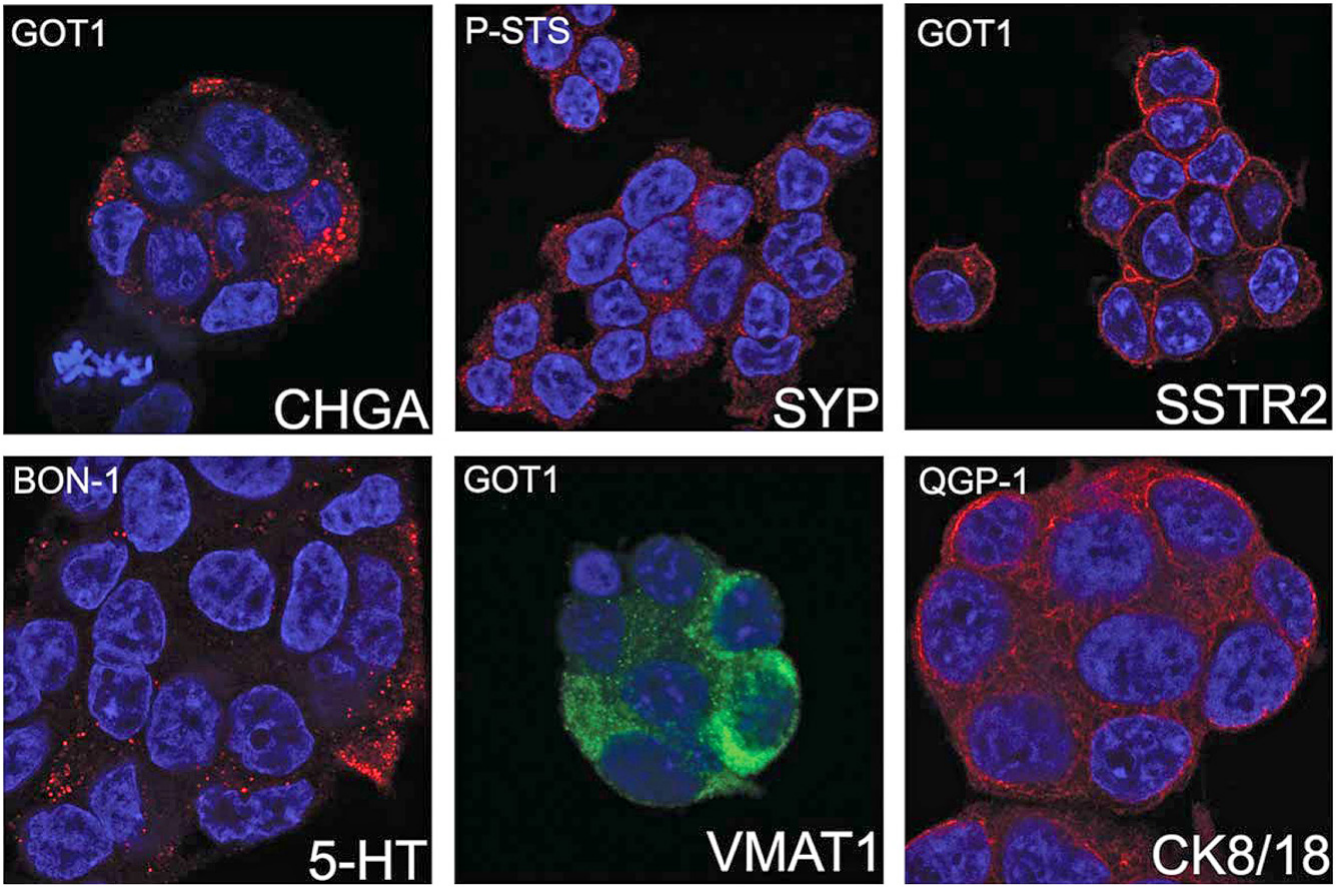
The subcellular localisation of neuroendocrine markers in GEPNET cell lines. The staining pattern of chromogranin A (CHGA), synaptophysin (SYP), chromaffin granule amine transporter 1 (VMAT1) and serotonin (5-HT) was consistent with localisation to secretory granules. The staining of somatostatin receptor type 2 (SSTR2) and cytokeratin 8/18 (CK8/18) was confirmed to be membranous and cytoskeletal, respectively.

Linage markers of SINETs (VMAT1 and 5-HT) were also investigated. VMAT1 was strongly stained in GOT1, weakly stained in BON-1 and QGP-1, and not stained in P-STS, while 5-HT was strong in GOT1, moderate in BON-1 and weak in P-STS and QGP-1. The localisation of synaptophysin, chromogranin A, VMAT1 and 5-HT to secretory granules was confirmed by confocal laser scanning microscopy (Fig. 2).

GEPNETs are derived from progenitor cells in the intestinal crypts, or pancreatic ducts, which express epithelial markers such as cytokeratins. To confirm the epithelial phenotype of NET cell lines, we stained for high-molecular-weight cytokeratins (AE1/AE3) and cytokeratin 8/18. The staining for high-molecular-weight cytokeratins was strong in all cell lines, while staining for cytokeratin 8/18 was weak to moderate in GOT1, P-STS, BON-1 and QGP-1 (Fig. 1).

Neuroendocrine marker, linage-specific marker and epithelial marker expression for GOT1, P-STS, BON-1 and QGP-1 overall well reflected that of clinical samples of GEPNETs. However, the Ki67 index of the cell lines was not representative of typical GEPNETs. GOT1 originated from a SINET liver metastasis with positivity for SYP, CgA, 5-HT, CK8/18 and with a Ki67 index of 1.3% (Grade I; data not shown). Furthermore, the Ki67 index of the cell lines correlated poorly to their doubling time (Fig. 1).

For the three putative SINET cell lines KRJ-I, L-STS and H-STS, we however, with the exception of NCAM, found only weak or no staining of neuroendocrine markers and could not detect any linage-specific markers or cytokeratins. These observations, in addition to the fact that these three cell lines grow non-adherently and form aggregates in cell culture (in contrast to the other GEPNET cell lines), made us question their authenticity. We hypothesised that KRJ-I, L-STS and H-STS were EBV-driven lymphoblastoid cell lines and thus performed immunohistochemical staining for lymphoid (CD45) and B cell markers (CD20). Both markers were highly expressed in KRJ-I, L-STS and H-STS, and the cell lines also contained high levels of EBV DNA, while the GOT1, P-STS, BON-1 and QGP-1 were negative (Supplementary Fig. 2). We therefore conclude that these cell lines are lymphoblastoid, and do not represent true GEPNET cell lines.

Somatostatin receptors are frequently over-expressed in GEPNETs and are used as a target for imaging and therapy. We performed staining for the somatostatin receptor subtype 2 (SSTR2) and found strong staining in GOT1 cells, weak staining in BON-1 and no staining in P-STS and QGP-1 (Fig. 1C). Confocal laser scanning microscopy showed strong membranous localisation of the SSTR2 protein in GOT1 cells (Fig. 2).

### GEPNET cell lines harbour multiple copy number alterations

Chromosomal changes have been linked to tumourigenesis and disease progression in patients with GEPNETs. Recurrent copy number alterations (CNAs) differ between SINETs and PanNETs, suggesting different mechanisms for tumour initiation and progression. To study CNAs in GEPNET cell lines, we performed whole-genome copy number profiling and found CNAs in all GEPNET cell lines (range 7‒44) (Supplementary Table 1).

**Figure 3:**
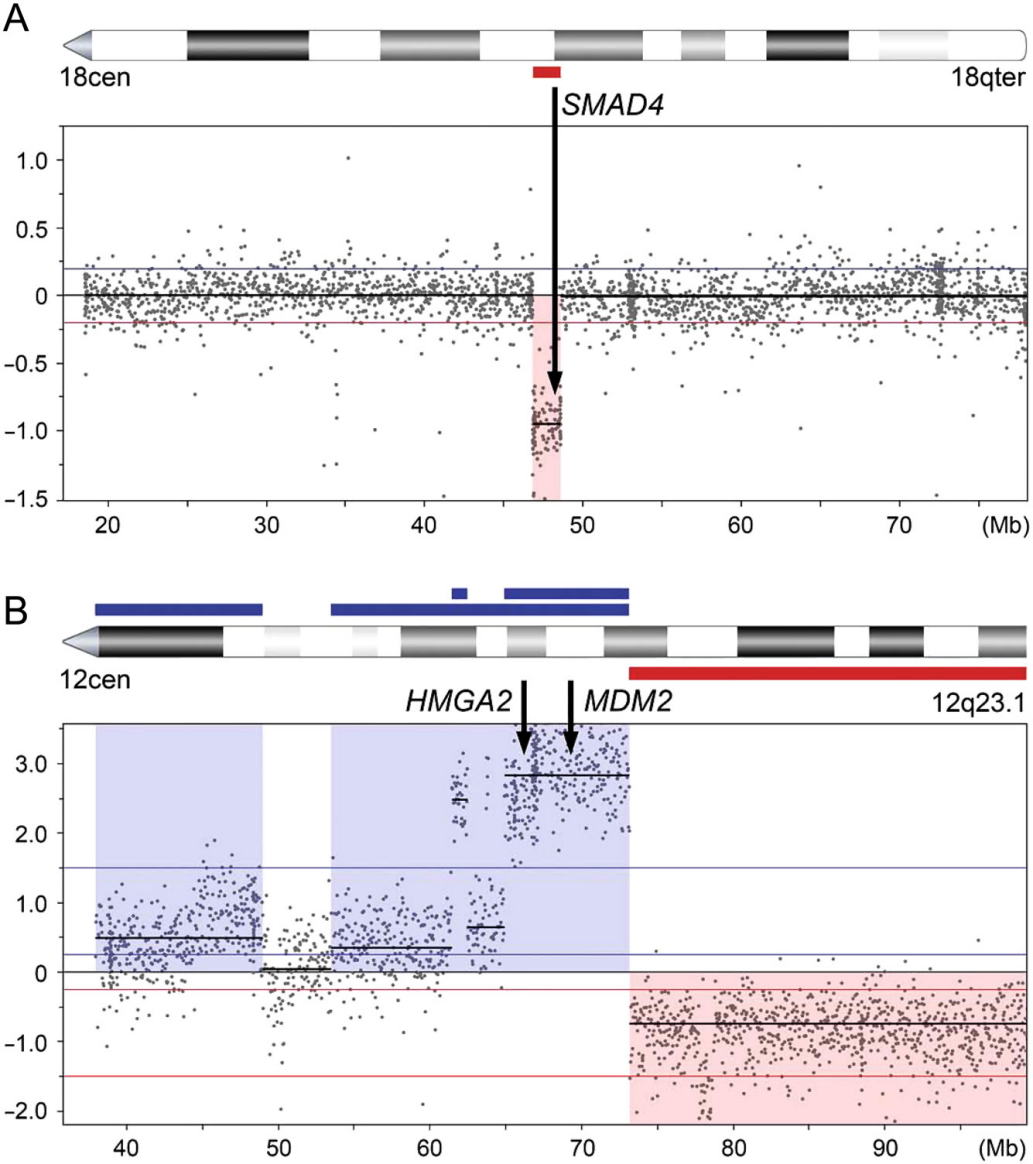
Copy number alterations detected in four GEPNET cell lines. (A) GOT1 harboured a loss of a 1.8 Mb segment on chromosome 18q, encompassing the *SMAD4* gene. (B) Of the three amplicons on chromosome 12 that QGP-1 harboured, one spanned 12q12.2–q21.1 including the *HMGA2* and *MDM2* genes.

The SINET cell lines GOT1 and P-STS had a lower frequency of alterations and a predominance of chromosomal losses. Loss of parts or whole chromosome 18 is the most common genomic event in SINETs (found in 60‒70% of the tumours). The GOT1 cell line showed loss of a 1.8 Mb segment of 18q, including the tumour suppressor *SMAD4* (Fig. 3A). The GOT1 cell line originated from a tumour that had loss of whole chromosome 18 and like GOT1, with predominance of losses and without gains of whole chromosomes (data not shown). The P-STS cell line had no losses on chromosome 18, but instead showed losses involving 11q, which is also a frequent alteration in SINETs (Kulke *et al*. 2008, Andersson *et al*. 2009). The PanNET cell lines BON-1 and QGP-1 had a higher frequency of CNAs. The BON-1 cell line had a predominance of gains, including whole chromosomes 2, 5, 7, 10 and 12, which are also frequent gains in PanNETs (Hu *et al*. 2010, Gebauer *et al*. 2014). BON-1 also harboured a homozygous loss of the *CDKN2A/B* tumour suppressor genes. The QGP-1 cell line had the highest number of CNAs and was the only cell line with gene amplifications. There were three amplicons on chromosome 12, one in 12p12.1, including *SOX5*, one in 12q14.1 and one in 12q12.2–q21.1 including *MDM2* and *HMGA2* (Fig. 3B). The lymphoblastoid cell lines L-STS and H-STS had no alterations, while KRJ-I harboured three small CNAs.

### GEPNET cell lines harbour mutations in several tumour suppressor genes, including *TP53*

Mutations in GEPNETs are rare, and the only identified recurrent gene mutation in SINETs is of *CDKN1B*, which occurs in less than a tenth of all tumours (Francis *et al*. 2013). Recurrent mutations in PanNETs have been found in several genes, including *ATRX*,* DAXX*, as well as genes involved in the Akt/mTOR pathway, DNA repair and chromatin remodelling (Jiao *et al*. 2011, Scarpa *et al*. 2017). In order to evaluate the mutational profiles of the GEPNET cell lines, we performed whole-exome sequencing and searched for tumour disease-associated genes.

**Figure 4:**
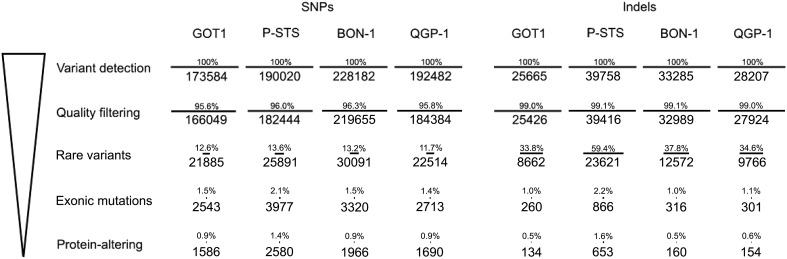
Whole-exome sequencing of GEPNET cell lines. Successive filtering with the remaining number of SNPs or indels in each step is shown. Percentage is relative to the number of variants detected in the detection step.

We detected an average of 196,067 single-nucleotide polymorphisms (SNPs) per cell line (range 173,584‒228,182). After quality filtering according to GATK best practices with the tool HaplotypeCaller, the number of reads was narrowed down to 188,133 on average (range 166,049‒219,655). In order to remove frequently occurring SNPs, all variants were further filtered against the 1000 Genomes Project database (Auton *et al*. 2015), where only SNPs with a frequency less than 0.01 were included. This generated an average of 25,095 rare SNPs per cell line. On average 1956 SNPs (range 1586‒2580) were located within exons and predicted to cause an alteration in the translated protein (Fig. 4 and Supplementary Table 2). Using the same filtering process, the number of indels was narrowed down from an average of 31,729 per cell line (range 25,665‒39,758) to an average of 275 (range 134‒653) protein-modifying events (Supplementary Table 3). Among the four GEPNET cell lines, P-STS had the highest number of protein-altering SNPs and indels and also displayed a higher degree of transitions compared to the other cell lines (Supplementary Fig. 3).

**Figure 5:**
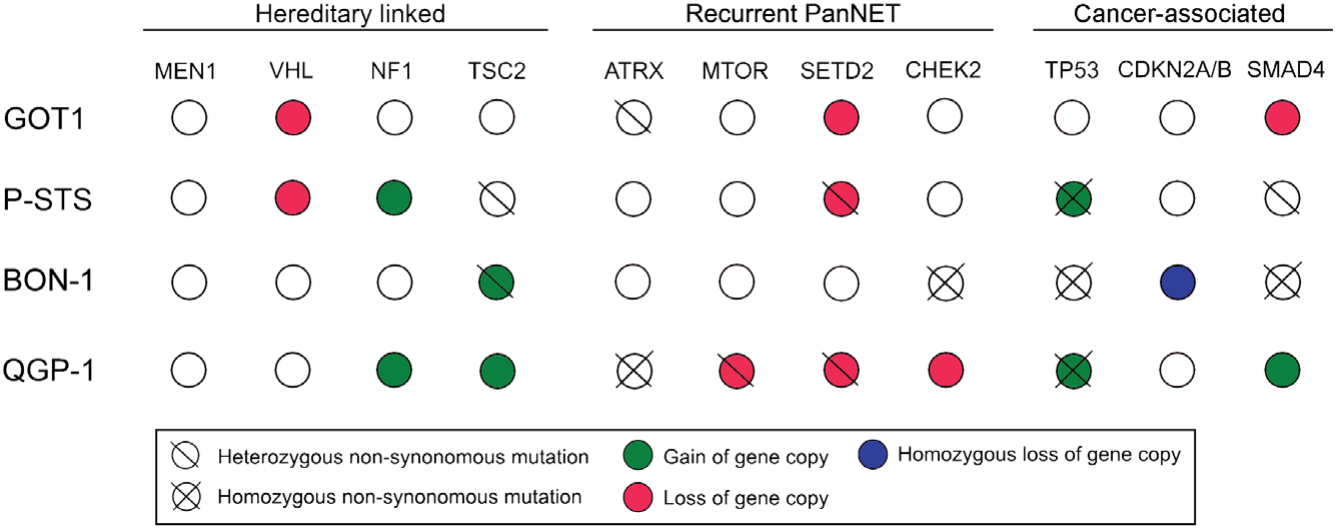
Genomic events involving genes linked to hereditary endocrine tumour syndromes, genes recurrently mutated in GEPNETs, and cancer-associated genes. Four genes have been hereditary linked to GEPNETs, none of which had bi-allelic inactivation in the cell lines. Out of previously identified recurrently mutated genes in GEPNETS, four had bi-allelic inactivations: *ATRX* (QGP-1), *MTOR* (QGP-1), *SETD2* (P-STS and QGP-1), and *CHEK2* (BON-1). Out of the 127 genes identified by the Tumor Cancer Genome Atlas, 49 had one or more protein-altering mutations in the cell lines; these genes included key tumour suppressors *TP53*, *CDKN2A*, *CDKN2B* and *SMAD4*.

We first investigated the cell lines for mutations in genes linked to hereditary endocrine tumour syndromes, i.e. *MEN1*,* VHL*, *NF1* and *TSC2* syndromes (Capelli *et al*. 2009, Corbo *et al*. 2010). Among these genes, only heterozygous* TSC2* mutations in P-STS and BON-1 were found (Fig. 5). None of the cell lines harboured mutations in the *MEN1*, *VHL* or *NF1* genes. Next, we searched for mutations in genes previously reported to be recurrently mutated in SINETs (Francis *et al*. 2013) and PanNETs (Jiao *et al*. 2011, Scarpa *et al*. 2017). *CDKN1B*, which is recurrently mutated in SINETs, was not affected in any of the cell lines. *ATRX*, *MTOR*, *SETD2* and *DIS3L2*, all of which have been found recurrently mutated in PanNETs, had bi-allelic inactivations in one or several cell lines. QGP-1 harboured homozygous mutations in *ATRX*, while GOT1 had a heterozygous mutation. QGP-1 had only one *MTOR* gene copy, which was mutated. *SETD2*, a gene coding for a histone modifier protein, had only one gene copy in GOT1, P-STS and QGP-1. This gene copy was mutated in P-STS and QGP-1 causing bi-allelic inactivation. DNA repair gene *CHEK2* showed a homozygous mutation in BON-1. Finally, we studied other cancer-associated genes, by analysing the 127 mutated genes identified in the Tumor Cancer Genome Atlas (TCGA) Pan-Cancer effort. (Kandoth *et al*. 2013) Out of these cancer-associated genes, 46 genes had heterozygous or homozygous mutations in one or more cell lines (Supplementary Table 4). Among affected genes, we found the key tumour suppressor *TP53* mutated in three out of four cell lines. *TP53* is seldom inactivated in GEPNETs, but here found mutated in P-STS, BON-1 and QGP-1. GOT1 was the only GEPNET cell line with wild-type *TP53*. The cell-cycle regulators *CDKN2A* and *CDKN2B* were inactivated by homozygous loss in BON-1. The *SMAD4* gene, involved in cell growth inhibition signalling, was lost in GOT1, had a heterozygous mutation in P-STS and a homozygous mutation in BON-1.

### PanNET and SINET cells are sensitive to inhibitors of MEK and HDAC

GEPNET cell lines have been used to study selected inhibitors for their efficacy and mechanism of action (Grozinsky-Glasberg *et al*. 2012). No comprehensive screening for effective inhibitors of GEPNETs has however been reported. One of this study’s aims was to define a therapeutic sensitivity profile for GEP-NETs. To this end, we performed an inhibitor screening with 1224 inhibitors, on the GEPNET cell lines GOT1, P-STS, BON-1 and QGP-1 using the non-tumourigenic cell lines MCF10A, BJ and HUV-EC-C as controls.

**Figure 6:**
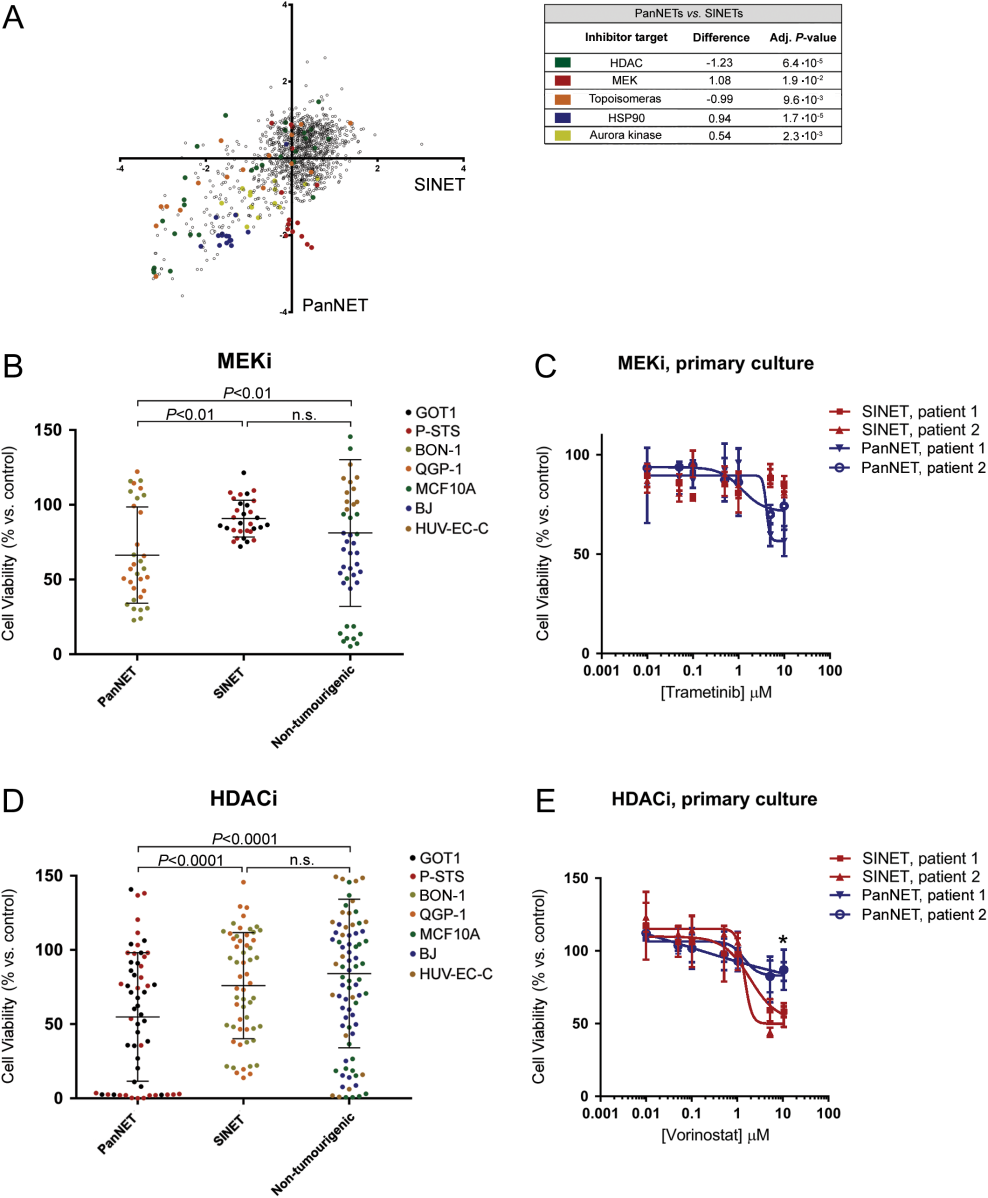
Therapeutic sensitivity of GEPNET cell lines and primary cell cultures. (A) Average *Z*-score representing effect on cell viability of individual inhibitors to SINETs (GOT1/P-STS) and PanNETs (BON-1/QGP-1), plotted against each other. Groups of inhibitors that are significantly more potent against SINETs or PanNETs are marked by colour. (B) The effect of all MEKi against SINET cells, PanNET cells and non-tumourigenic cells. MEKi are more potent against PanNET cells, compared to SINET and non-tumourigenic cells. (C) Comparing the sensitivity of PanNET and SINET first-passage primary cells to MEKi trametinib. (D) SINET cell lines are more sensitive to HDACi, compared to PanNET cells and non-tumourigenic cells. (E) First-passage primary SINET cells are seemingly more sensitive than primary PanNET cells to the HDACi vorinostat. (B and D) Bars indicate mean effect, error bars s.d. and *P* values generated from Wilcoxon signed-rank test. (C and E) Dose–response curves represent a mean of *n* = 3 and the error bars denote standard deviation (s.d.).

This resulted in data regarding the efficacy of a large number of inhibitors and protein targets (Supplementary Table 5). In the data, we observed an overall high degree of similarity in inhibitor sensitivity between cell lines derived from SINETs and PanNETs. We could however identify several groups of inhibitors that were significantly more selective for PanNET cells or SINET cells (Fig. 6A). Inhibitors of HDAC and topoisomerase were more efficient against SINETs, and inhibitors of MEK, HSP90 and Aurora kinase were more efficient against PanNETs. Looking at the anti-tumour effect of all MEKi in the screening library, SINET cells were largely unaffected, while most of the MEKi were efficient against PanNET cell lines and in a lesser degree against non-tumourigenic cells (Fig. 6B). To confirm these findings, we used first-passage primary tumour cells, prepared from patient SINETs and PanNETs collected at surgery. The authenticity of tumours was verified using immunohistochemistry (Supplementary Fig. 4 and Supplementary Table 6). FDA-approved MEK inhibitor trametinib was used to treat these cells (Fig. 6C). While many HDACi had no or limited effect, this was mostly true for PanNET cells and non-tumourigenic cells. A large number of HDACi had, in fact, a potent effect against SINET cells (Fig. 6D). In primary cells, FDA-approved HDACi vorinostat was seemingly more efficient against SINETs than PanNETs at the highest tested concentration (Fig. 6E).

## Discussion

We here report the first comprehensive characterisation of seven GEPNET-derived cell lines, including evaluation of their neuroendocrine phenotype, genomic alterations and therapeutic sensitivity profiles. We found a preserved neuroendocrine phenotype in four cell lines and that these cell lines have genomic alterations characteristic of GEPNETs, albeit while also harbouring additional genomic alterations. A large-scale inhibitor screening was performed, providing a basis for future therapeutic developments and identified HDAC and MEK as promising protein targets for therapy.

NETs are defined by their expression of neuroendocrine markers, e.g. synaptophysin and chromogranin A, which are components of small synaptic-like vesicles, and large dense-core vesicles, respectively (Rindi *et al*. 1986, Wiedenmann *et al*. 1986). Furthermore, GEPNETs are derived from progenitor cells in the intestinal crypts or pancreatic ducts, and consequently express epithelial markers, e.g. cytokeratins. We showed that four GEPNET cell lines, GOT1, P-STS, BON-1 and QGP-1, all expressed synaptophysin and NCAM strongly, while expression of chromogranin A, PGP9.5, NSE, CD57 and lineage-specific markers VMAT1 and 5-HT varied between the cell lines. In GOT1 and BON-1, we also found expression of SSTR2, a major target for symptomatic treatment of hormonal symptoms, tumour imaging and peptide receptor radiotherapy in GEPNET patients. These findings demonstrate the neuroendocrine phenotype of these cell lines, and their usefulness as models for studying somatostatin receptor-targeted radiotherapy in neuroendocrine tumours (Reubi *et al*. 2000). All four cell lines also stained positively for cytokeratins. We failed, however, to find any neuroendocrine differentiation or expression of cytokeratins in the KRJ-I, L-STS and H-STS cell lines. These cell lines grew as suspension cultures, forming floating aggregates. Based on this, we hypothesised that they might be lymphoblastoid cell lines. We went on to show that these cell lines expressed lymphoid markers CD45 and CD20 and contained high levels of EBV DNA, confirming our hypothesis. The lack of NET markers and cytokeratins, and the presence of lymphoid markers plus EBV DNA, has also been found in early passages of these cell lines at the laboratory in which they were established (R Pfragner, unpublished observations). While KRJ-I in the initial characterisation displayed a positivity for anti-chromogranin A and HISL19 staining, it was also shown that the culture consisted of clones with both high and low growth rates, and with varying anchorage dependency (Pfragner *et al*. 1996). The established cell line later consisted only of a suspension of single cells and multicellular spheroids. P-STS was shown to express neuroendocrine markers in all cells, while the staining frequency and intensity of neuroendocrine markers in L-STS and H-STS was variable (Pfragner *et al*. 2009). Based on these early observations, combined with the present investigation, has lead us to believe that the loss of a neuroendocrine phenotype in KRJ-I, L-STS and H-STS was because the cultures initially consisted of both neuroendocrine and lymphoblastoid cells. The neuroendocrine cells were then were completely taken over by the lymphoblastoid cells. We also characterised primary cell cultures, which as expected showed expression of all general neuroendocrine and epithelial markers while lymphoid markers could not be detected.

SINETs are often subcategorised into at least two groups based on their CNAs. The largest group of tumours is characterised by chromosomal losses, including loss of chromosome 18, and is associated with longer patient survival. A smaller group of tumours is characterised by multiple chromosomal gains and is associated with shorter patient survival (Kulke 2008, Andersson *et al*. 2009, Karpathakis *et al.* 2016). PanNETs harbour recurrent CNAs characterised by a predominance of gains (Gebauer *et al*. 2014). To study the genomic profile of the GEPNET cell lines, we performed arrayCGH and found that they had very different CNA profiles. The SINET cell lines GOT1 and P-STS were characterised by a predominance of chromosomal losses. The PanNET cell line BON-1, on the other hand, was characterised by a predominance of gains, including gain of several whole chromosomes. Among the cell lines investigated, QGP-1 had the highest frequency of CNAs, with an equal number of losses and gains. GOT1 had partial losses on 18q encompassing the tumour suppressor *SMAD4*, which is believed to be haploinsufficient (Howe *et al*. 1998, Xu *et al*. 2000, Alberici *et al*. 2006). BON-1 had homozygous loss of the tumour suppressor genes *CDKN2A* and *CDKN2B*, an event that has previously been reported in this cell line (Vandamme *et al*. 2015). The QGP-1 cell line had amplifications involving three segments on chromosome 12, including the genes *MDM2* and *HMGA2*. Interestingly, amplification and overexpression of *MDM2* is a well-known mechanism of TP53 inactivation occurring in 22% of all PanNETs, while overexpression of *HMGA2* is associated with a malignant phenotype (Abe *et al*. 2003, Hu *et al*. 2010).

PanNETs frequently have somatic mutations in *MEN1*, *ATRX* or *DAXX*. *ATRX*, located on the X-chromosome, was mutated in QGP-1 (male) and had a heterozygous mutation in GOT1 (female), possibly leading to a bi-allelic inactivation of the gene. No mutations in *DAXX* or *MEN1* were found in any of the cell lines. While expression studies have indicated that mTOR signalling is upregulated in most PanNET tumours (Perren *et al*. 2000, Missiaglia *et al*. 2010), whole-exome and whole-genome sequencing studies have found mutations in negative regulators of the signalling pathway in only 12–15% of patient tumours (Jiao *et al*. 2011, Scarpa *et al*. 2017). None of these genes were mutated in any of the GEPNET cell lines, but *MTOR* was altered in QGP-1. *CDKN1B* is the only gene so far reported to be recurrently mutated in a subset of SINETs (Francis 2013). We did not find any mutations affecting *CDKN1B* in any of the GEPNET cell lines. Due to selection pressure and stress during *in vitro* culturing, tumour cell lines often acquire novel mutations that may not be found in the tumour from which they were derived. Acquired mutations may affect the growth properties of cell lines, and influence their therapeutic sensitivity. *TP53*, encoding a tumour suppressor and key regulator of DNA repair and apoptosis, is usually unaffected in GEPNETs, but was found to be bi-allelically inactivated in P-STS, BON-1 and QGP1. We found a homozygous stop-loss mutation in BON-1 and a frameshift deletion in QGP-1, in accordance with a previous report (Vandamme *et al*. 2015).

To date, GEPNET cell lines have only been used to study the potency and mechanisms of action of a limited number of inhibitors. In the present study, we performed a comprehensive inhibitor screening to obtain a sensitivity profile representing a wide range of therapeutic principles. The screening identified inhibitors that are selectively efficient to SINET or PanNET cell lines. To study these findings in another model, we used first-passage primary cells. The primary cell cultures expressed neuroendocrine markers similar to the investigated GEPNET cell lines. Experiments showed that vorinostat killed SINET primary cells more efficiently than PanNET primary cells and that trametinib was more efficient against PanNET primary cells. This effect was however only prominent at high doses, suggesting that observed effects might be due to off-target activity. HDAC inhibition has also previously been suggested as a treatment option for GEPNET patients (Baradari *et al*. 2006, Arvidsson *et al*. 2016, Sun *et al*. 2016).

From these findings combined, we conclude that the four cell lines GOT1, P-STS, BON-1 and QGP-1 are indeed authentic neuroendocrine tumour cell lines, harbouring genomic events characteristic of GEPNETs, but also cancer-associated mutations that most likely have occurred in cell culture. The identification of lymphoblastoid cell lines among putative GEPNETs emphasises the need to thoroughly characterise GEPNET cell lines used as models for neuroendocrine tumour disease. Our observations also emphasises the need to be cautious when drawing conclusions from studies performed on GEPNET cell lines. One major concern is the origin of GEPNET cell lines and whether they were derived from well-differentiated neuroendocrine tumours or poorly differentiated neuroendocrine carcinomas. The difficulties in establishing GEPNET cell lines are usually attributed to the low proliferation rate of these tumours. One may argue that available GEPNET cell lines were established from more aggressive tumours that should be classified as neuroendocrine carcinomas. The occurrence of *TP53* mutations in three of the investigated cell lines (P-STS, BON-1 and QGP-1) further strengthens this concern. The original publications on GEPNET cell lines do not contain sufficient data to determine the grade of the tumours from which cell lines were derived. We have therefore reinvestigated the origin of the GOT1 cell line and found it to be a liver metastasis from a well-differentiated, serotonin-producing (enterochromaffin cell type) ileal NET. The liver metastasis was of grade 1 (WHO 2010) and harboured a loss of chromosome 18. We conclude that it remains to be proven to what extent GEPNET cell lines are representative for the tumour disease from which they were derived. Experimental data obtained from GEPNET cell lines should therefore be carefully validated using patient tumours.

## Supplementary Material

Supplementary Figure 1

Supplementary Figure 2

Supplementary Figure 3

Supplementary Figure 4

Supplementary Table 1

Supplementary Table 2

Supplementary Table 3

Supplementary Table 4

Supplementary Table 5

Supplementary Table 6

## Declaration of interest

The authors declare that there are no conflicts of interests that could be perceived as prejudicing the impartiality of the research reported.

## Funding

This study was supported by grants from the Swedish Cancer Society, the Assar Gabrielsson Research Foundation, the Wilhelm and Martina Lundgren Foundation for Scientific Research, the Sahlgrenska University Hospital Funds, the BioCARE National Strategic Research Program and by ALF grants from the Sahlgrenska University Hospital.
