# The neuroendocrine phenotype, genomic profile and therapeutic sensitivity of GEPNET cell lines

**DOI:** 10.1530/ERC-17-0445e

**Published:** 2018-04-01

**Authors:** Tobias Hofving, Yvonne Arvidsson, Bilal Almobarak, Linda Inge, Roswitha Pfragner, Marta Persson, Göran Stenman, Erik Kristiansson, Viktor Johanson, Ola Nilsson

**Affiliations:** 1 Sahlgrenska Cancer Center, Department of Pathology and Genetics, Institute of Biomedicine, Sahlgrenska Academy at the University of Gothenburg, Gothenburg, Sweden; 2 Institute of Pathophysiology and Immunology, Center for Molecular Medicine, Medical University of Graz, Graz, Austria; 3 Department of Mathematical Sciences, Chalmers University of Technology, Gothenburg, Sweden; 4 Department of Surgery, Institute of Clinical Sciences, Sahlgrenska Academy at the University of Gothenburg, Gothenburg, Sweden

The authors and journal apologise for an error in the above paper, which appeared in volume 25 part 3, pages 367–380. The error relates to the artwork of Fig. 6D on page 375, where the *x*-axis labels ‘PanNET’ and ‘SINET’ where transposed. The correct Fig. 6 is published in full below:

**Figure 6:**
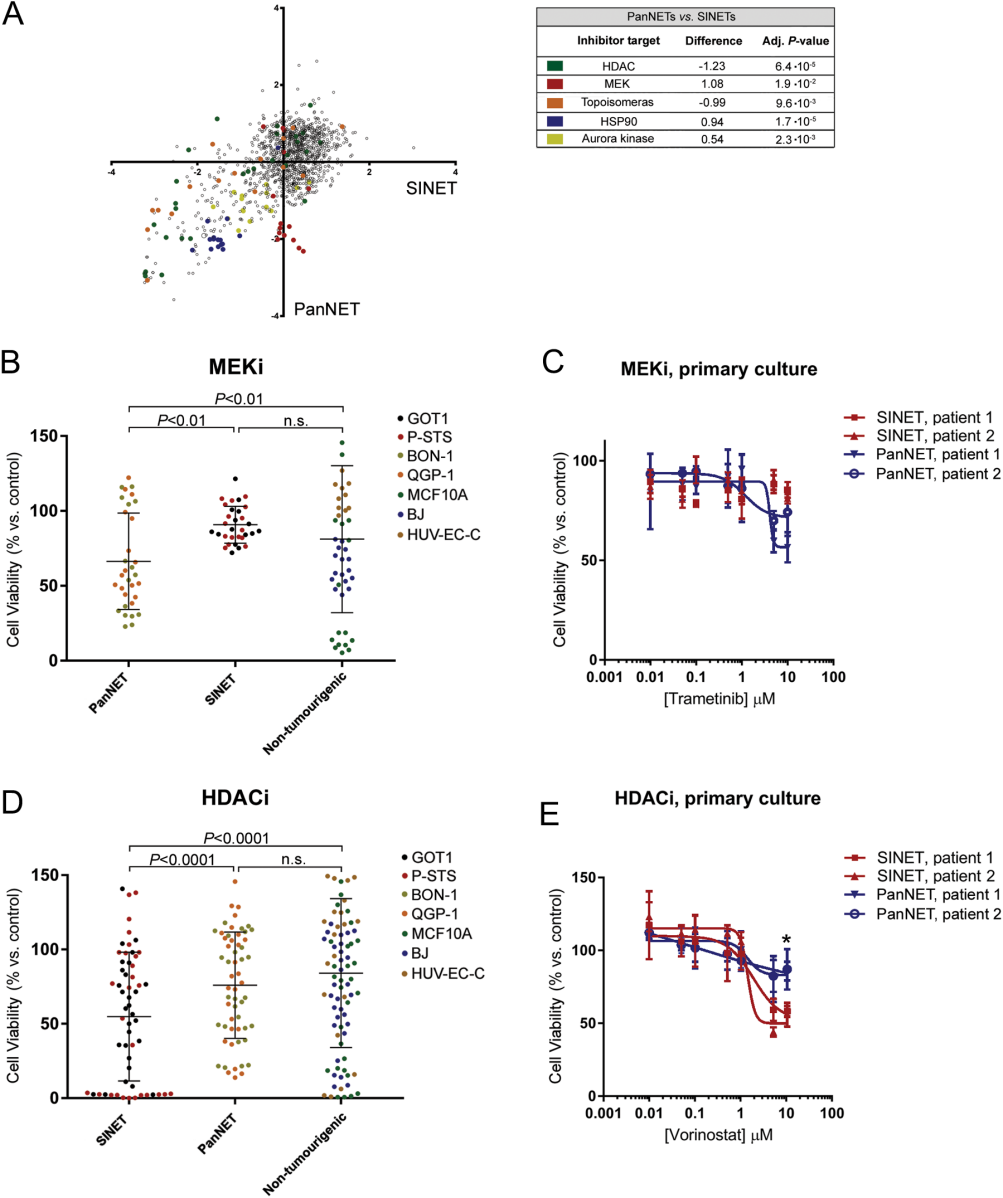
Therapeutic sensitivity of GEPNET cell lines and primary cell cultures. (A) Average *Z*-score representing the effect on cell viability of individual inhibitors to SINETs (GOT1/P-STS) and PanNETs (BON-1/QGP-1), plotted against each other. Groups of inhibitors that are significantly more potent against SINETs or PanNETs are marked by colour. (B) The effect of all MEKi against SINET cells, PanNET cells and non-tumourigenic cells. MEKi are more potent against PanNET cells, compared to SINET and non-tumourigenic cells. (C) Comparing the sensitivity of PanNET and SINET first-passage primary cells to MEKi trametinib. (D) SINET cell lines are more sensitive to HDACi, compared to PanNET cells and non-tumourigenic cells. (E) First-passage primary SINET cells are seemingly more sensitive than primary PanNET cells to the HDACi vorinostat. (B and D) Bars indicate mean effect, error bars s.d. and *P* values generated from Wilcoxon signed-rank test. (C and E) Dose–response curves represent a mean of *n* = 3 and the error bars denote standard deviation (s.d.).

