# Supplementary material for: The neuroendocrine phenotype, genomic profile and therapeutic sensitivity of GEPNET cell lines
Source: Endocr Relat Cancer. 2018 Jan 15;25(3):367–80. doi: 10.1530/ERC-17-0445 (PMC5827037; doi:10.1530/ERC-17-0445)
Supplement: Supplementary Figure 1 [file erc-25-309-s001.pdf]

| | AMEL | CSF1PO | D13S317 | D16S539 | D21S11 | D5S818 | D7S820 | TH01 | TPOX | vWA | |
|---|---|---|---|---|---|---|---|---|---|---|---|
| **GOT1** | X | 11, 12 | 12 | 12 | 30, 31.2 | 12 | 10, 12 | 6, 9.3 | 8, 11 | 15, 16 | 80.0 % (1) |
| **GOT1 Donor tissue** | X | 10, 11, 12 | 11, 12 | 11, 12 | 28, 30, 31.2 | 12, 13 | 10, 12 | 6, 9.3 | 8, 11 | 14, 15, 16, 17, 18 | |
| **KRJ-1** | X, Y | 11, 13 | 11, 12 | 11, 12 | 28, 29 | 11 | 10 | 7, 9 | 10 | 18, 19 | * |
| **P-STS** | X, Y | 10, 11 | 8, 13 | 12, 13 | 29, 31 | 12 | 10, 13 | 9, 9.3 | 8 | 20, 21, 22 | 65.1 % |
| **L-STS** | X, Y | 10, 11 | 8, 13, 14 | 12, 13 | 28, 29, 30 | 11, 12 | 10, 12 | 6, 9, 9.3 | 8 | 14, 17, 18, 19 | 55,6 % |
| **H-STS** | X, Y | 10, 11 | 8, 14 | 13 | 28, 30 | 11 | 10, 12 | 9, 9.3 | 8 | 17, 19 | 82.9 % |
| **BON1** | X, Y | 10, 11 | 11, 12 | 10, 11 | 28, 33.2 | 9, 12 | 9 | 8 | 9 | 18, 19 | * |
| **QGP-1** | X | 10, 12 | 13 | 10, 12 | 29 | 12 | 12 | 6, 9 | 8, 11 | 14, 18 | * |
| **BJ** | X, Y | 10, 12 | 8, 9 | 9, 13 | 29 | 12 | 11, 12 | 7, 8 | 10, 11 | 16, 18 | ** |
| **MCF10A** | X | 10, 12 | 8, 9 | 11, 12 | 28, 30 | 10, 13 | 10, 11 | 8, 9.3 | 9, 11 | 15, 17 | ** |
| **HUVEC** | X | 11, 12 | 9, 11 | 11, 12 | 28, 31 | 11, 12 | 8, 12 | 6, 9.3 | 8, 11 | 16 | ** |

1) ANSI/ATCC ASN-0002-2011. Authentication of Human Cell Lines: Standardization of STR Profiling. ANSI eStandards Store, 2012.

* Not deposited in ATCC or DMSZ cell banks. No > 80 % hits among deposited cell lines.

** 100 % match to corresponding cell line in ATCC/DMSZ cell banks.

Supplementary Figure 1
