# Supplementary figures and images for: The neuroendocrine phenotype, genomic profile and therapeutic sensitivity of GEPNET cell lines
Source: Endocr Relat Cancer. 2018 Jan 15;25(3):367–80. doi: 10.1530/ERC-17-0445 (PMC5827037; doi:10.1530/ERC-17-0445)

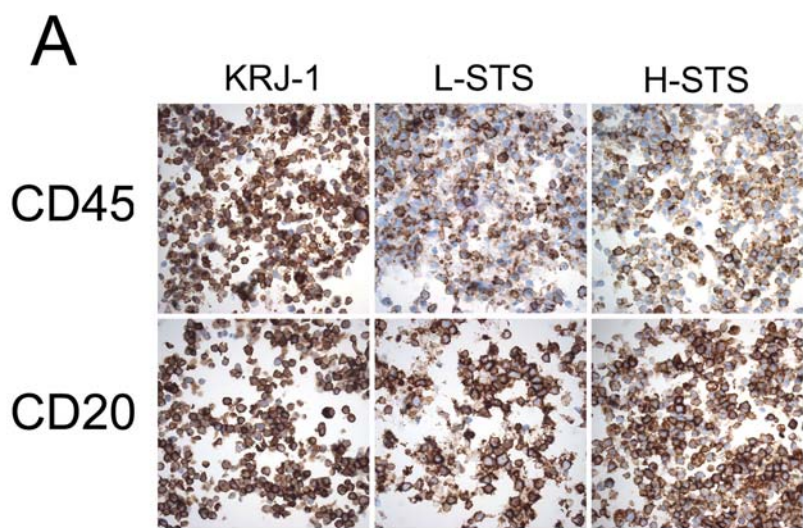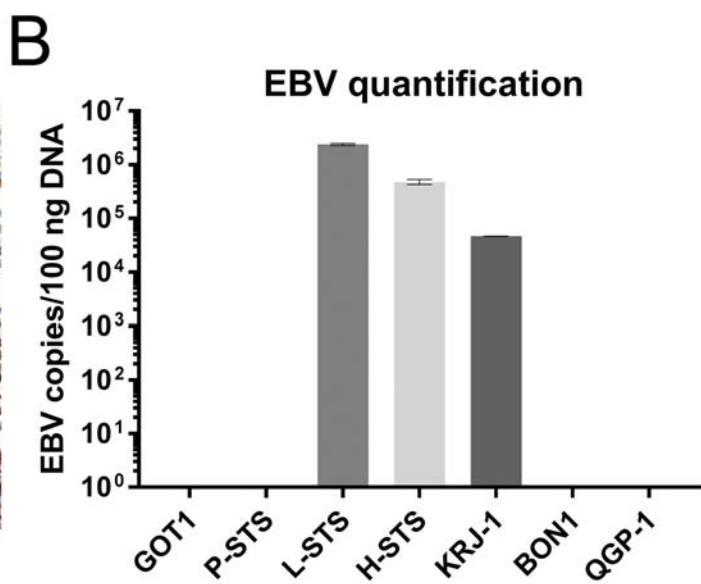

Supplementary Figure 2

Supplement: Supplementary Figure 2 [file erc-25-309-s002.pdf]

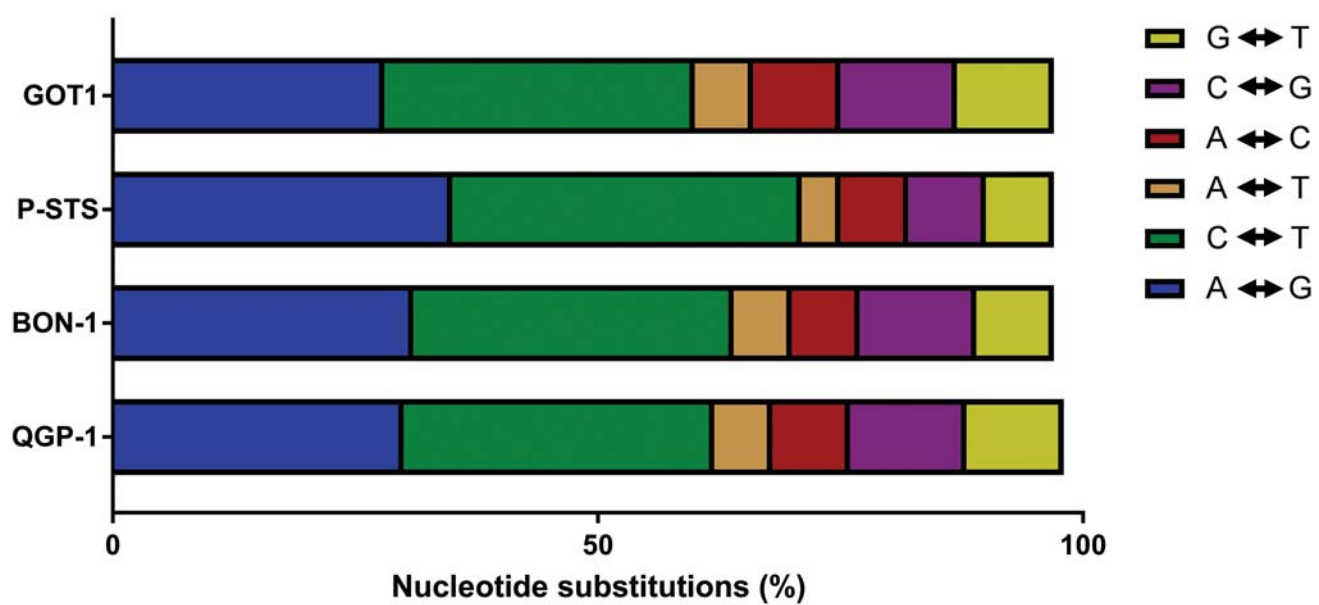

Supplementary Figure 3

Supplement: Supplementary Figure 3 [file erc-25-309-s003.pdf]
