# Supplementary material for: The neuroendocrine phenotype, genomic profile and therapeutic sensitivity of GEPNET cell lines
Source: Endocr Relat Cancer. 2018 Jan 15;25(3):367–80. doi: 10.1530/ERC-17-0445 (PMC5827037; doi:10.1530/ERC-17-0445)
Supplement: Supplementary Figure 4 [file erc-25-309-s004.pdf]

		SI-NET Pat1	SI-NET Pat2	PanNET Pat1	PanNET Pat2
Neuroendocrine	SYP	+++	+++	+++	+++
	CHGA	+++	+++	+	+
	PGP9.5	+++	++	+	+
	N-CAM	+++	+++	+++	+++
	NSE	+++	+++	+++	+++
	CD57	+++	+++	++	++
Enterochromaffin	VMAT1	+++	+++	n.d.	n.d.
	5-HT	+++	+++	n.d.	n.d.
Epithelial	CK8/18	+++	+++	++	+++
	Pan-CK	+++	+++	+++	+++
Lymphoid	CD45	n.d.	n.d.	n.d.	n.d.
	CD20	n.d.	n.d.	n.d.	n.d.
Ki67		2.2%	0.6%	1.0%	0.5%

Supplementary Figure 4
