# Supplementary material for: The neuroendocrine phenotype, genomic profile and therapeutic sensitivity of GEPNET cell lines
Source: Endocr Relat Cancer. 2018 Jan 15;25(3):367–80. doi: 10.1530/ERC-17-0445 (PMC5827037; doi:10.1530/ERC-17-0445)
Supplement: Supplementary Table 1 [file erc-25-309-t001.pdf]

Supplementary Table 1. Genomic imbalances detected by arrayCGH in GEPNET cell lines.

**Cell line**	**Cytoband**	**Length (bp)**	**Genomic imbalance**
GOT1, passage 45			
	1p36.33 - p13.3	109191259	Loss
	1p13.3-q31.1	58708655	Gain
	1q31.2-q41	28351918	Gain
	1q41 - qter	26850149	Loss
	2p24.2 - p23.3	8408045	Loss
	2p21	4441720	Loss
	2p14 - p12	6639225	Loss
	2q11.1 - q11.2	5460015	Loss
	2q33.1	719974	Loss
	3pter - p21.1	53100666	Loss
	3q13.32 - q21.3	8125366	Loss
	4p16.2 - p16.1	925203	Loss
	4q31.23 - q31.3	3579507	Loss
	4q32.3 - q34.2	10500897	Loss
	5q14.3 - q32	61412619	Loss
	6q16.2 - q22.31	20304396	Loss
	6q23.2 - q27	34455998	Loss
	12pter - p13.32	4748900	Gain
	13q11 - qter	96145131	Loss
	15q11.1 - qter	82476256	Loss
	16q23.1 - qter	14586137	Loss
	18p11.22 - p11.21	1619542	Loss
	18q21.1 - q21.2	1797368	Loss
	20pter-p12.3	9051694	Gain
	20q13.31 - qter	7402203	Gain
P-STS, passage 18			
	1pter - p21.3	96926798	Gain
	3pter - p11.2	88238786	Loss
	4q35.1 - q35.2	6612651	Loss
	11q23.2 - q23.3	4094762	Loss
	11q23.3	745292	Loss
	17pter - qter	81195210	Gain
	20pter - p12.1	14257163	Loss
BON-1, passage 19			
	1p36.33 - p34.3	35891807	Gain
	1p21.3 - qter	129467050	Gain

	2pter-qter	243199373	Gain
	5pter-qter	180915260	Gain
	7pter-qter	159138663	Gain
	8pter - p23.1	8063092	Loss
	8q22.3 - qter	41662447	Gain
	9p21.3	1924506	Homozygous loss
	10pter-qter	135534747	Gain
	12pter-qter	133851895	Gain
	14q11.2 - qter	107349540	Gain*
	15q11.1 - qter	82416085	Gain
	16pter-qter	90354753	Gain
	18q23	2255620	Loss
	19pter - q13.2	37906802	Gain
	19q13.32 - qter	12924979	Gain
	20pter-qter	63025520	Gain
	21q11.2 - qter	33671792	Gain
QGP-1, passage 3**			
	1pter - p36.31	7199334	Gain
	1p36.31 - p35.3	22521029	Loss
	1p34.3 - q25.1	117924054	Gain
	1q25.2	1266230	Loss
	1q31.1 - q31.3	9569738	Gain
	2p25.3 - p24.3	16619101	Gain
	2p22.2 - q36.1	179602820	Gain
	2q36.1 - q36.2	1292102	Loss
	2q36.2 - qter	17420200	Gain
	3p26.3 - p26.1	1979347	Loss
	3p25.1 - p14.3	42243945	Loss
	3p14.3 - p13	15473792	Loss
	4q28.1 - qter	63109313	Loss
	5pter-qter	180915260	Gain
	6p22.3	2458057	Gain
	6q11.2 - qter	107718299	Loss
	7pter-qter	159138663	Gain
	8pter - q12.2	150768471	Loss
	9pter - p23	13882622	Loss
	9p13.3 - p11.2	10878972	Loss
	10q21.1 - q21.2	5641052	Loss
	11q12.1	1795892	Loss
	11q13.5 - q22.3	31834896	Gain
	12pter - p13.31	9304751	Gain

	12p13.31 - p12.3	9139744	Loss
	12p12.3 - q13.11	30542515	Gain
	12p12.1	1143689	Amplification
	12q13.13 - q21.1	19690296	Gain
	12q14.1	1037808	Amplification
	12q14.2 - q21.1	8278841	Amplification
	12q21.1 - qter	60665996	Loss
	13q11 - qter	95941634	Loss
	14q11.2 - qter	88248859	Loss
	15q26.1 - qter	11466307	Gain
	16p11.2 - qter	56574561	Gain
	17pter-qter	81195210	Gain
	18pter - q21.33	59214742	Gain
	18q21.33 - qter	18868307	Loss
	19q12 - q13.12	7734801	Loss
	20pter - q11.22	34329358	Gain
	20q11.22 - q13.2	15647508	Loss
	20q13.2 - qter	13047522	Gain
	21q11.2 - qter	33671792	Loss
	22q11.1 - qter	35107562	Loss

* The region 14q11.2 - q21.2 (27985768 bp) contains an extra copy

** Number of passages since purchase from JCRB cell bank
