# Supplementary material for: The neuroendocrine phenotype, genomic profile and therapeutic sensitivity of GEPNET cell lines
Source: Endocr Relat Cancer. 2018 Jan 15;25(3):367–80. doi: 10.1530/ERC-17-0445 (PMC5827037; doi:10.1530/ERC-17-0445)
Supplement: Supplementary Table 2 [file erc-25-309-t002.pdf]

Supplementary Table 2. Filtered SNP mutations.

Annotation	Gene	Chr	Position	Ref	GOT1	P-STS	BON-1	QGP-1
nonsynonymous SNV	A1BG	chr19	58858850	C	C	C	A_het	C
nonsynonymous SNV	A1BG	chr19	58863725	G	G	G	C_het	G
nonsynonymous SNV	A2ML1	chr12	9010622	T	T	C_het	T	T
stopgain	A4GNT	chr3	137843333	G	A_het	G	G	G
nonsynonymous SNV	AAGAB	chr15	67495926	G	A_hom	G	G	G
nonsynonymous SNV	AANAT	chr17	74465916	C	C	T_het	C	C
nonsynonymous SNV	AASDH	chr4	57215803	G	G	A_het	G	G
nonsynonymous SNV	ABCA13	chr7	48563879	C	C	C	C	A_het
nonsynonymous SNV	ABCA3	chr16	2326692	C	C	T_het	C	C
nonsynonymous SNV	ABCA3	chr16	2329008	C	C	T_het	C	C
nonsynonymous SNV	ABCB10	chr1	229665958	C	C	T_het	C	C
nonsynonymous SNV	ABCB4	chr7	87069130	C	C	C	G_het	C
nonsynonymous SNV	ABCC11	chr16	48234330	C	C	C	C	T_het
nonsynonymous SNV	ABCC5	chr3	183700642	G	G	A_het	G	G
nonsynonymous SNV	ABCC6	chr16	16302586	T	T	C_het	T	T
nonsynonymous SNV	ABCC9	chr12	21958206	C	A_het	C	C	C
nonsynonymous SNV	ABHD14A	chr3	52014985	C	C	T_hom	C	C
nonsynonymous SNV	ABHD16B	chr20	62493357	C	C	C	T_het	C
nonsynonymous SNV	ABHD2	chr15	89719079	T	T	C_het	T	T
nonsynonymous SNV	ABI3BP	chr3	100508300	G	G	G	G	T_het
nonsynonymous SNV	ABL1	chr9	133748331	A	A	A	G_hom	A
nonsynonymous SNV	ABL2	chr1	179078211	C	C	T_het	C	C
nonsynonymous SNV	ABLIM3	chr5	148579917	G	G	G	A_het	G
nonsynonymous SNV	ABLIM3	chr5	148618835	T	T	A_het	T	T
nonsynonymous SNV	ACACB	chr12	109690904	G	G	G	A_het	G
nonsynonymous SNV	ACADM	chr1	76216149	C	C	T_het	C	C
nonsynonymous SNV	ACAN	chr15	89392915	C	C	C	C	T_het
nonsynonymous SNV	ACAN	chr15	89400023	A	G_hom	A	G_het	G_hom
nonsynonymous SNV	ACCS	chr11	44102798	G	G	C_het	G	G
nonsynonymous SNV	ACE	chr17	61557166	C	C	T_het	C	C
nonsynonymous SNV	ACIN1	chr14	23564437	T	C_het	C_het	T	T
nonsynonymous SNV	ACOT2	chr14	74036495	A	G_het	A	A	A
nonsynonymous SNV	ACOT2	chr14	74036497	A	G_het	A	A	A
nonsynonymous SNV	ACOT2	chr14	74036498	C	T_het	C	C	C
nonsynonymous SNV	ACOT2	chr14	74041748	A	A	A	G_het	A
nonsynonymous SNV	ACOT9	chrX	23749080	T	T	T	C_hom	T
nonsynonymous SNV	ACR	chr22	51176730	G	G	A_het	G	G
nonsynonymous SNV	ACSM2A	chr16	20476995	G	A_het	G	A_het	G
nonsynonymous SNV	ACSM5	chr16	20432665	C	C	C	C	G_hom
nonsynonymous SNV	ACTN1	chr14	69341653	T	A_het	T	T	T
nonsynonymous SNV	ACTN4	chr19	39207742	G	G	G	A_het	G
stopgain	ACVR1	chr2	158622634	C	C	A_het	C	C

nonsynonymous SNV	ADAM11	chr17	42848989	G	G	G	G	A_hom
stopgain	ADAM12	chr10	127755316	G	G	T_het	G	G
nonsynonymous SNV	ADAM12	chr10	127967548	C	T_het	C	C	C
nonsynonymous SNV	ADAM2	chr8	39694686	C	C	C	C	T_hom
nonsynonymous SNV	ADAM20	chr14	70990784	G	G	G	T_het	G
nonsynonymous SNV	ADAM21	chr14	70924501	C	G_hom	G_hom	G_het	G_hom
nonsynonymous SNV	ADAM21P1	chr14	70713858	T	T	C_het	C_het	C_hom
nonsynonymous SNV	ADAM3A	chr8	39323405	G	G	A_hom	G	G
nonsynonymous SNV	ADAM3A	chr8	39331471	C	A_hom	C	C	C
nonsynonymous SNV	ADAM8	chr10	135084481	C	C	C	T_het	C
nonsynonymous SNV	ADAM8	chr10	135087295	G	G	G	A_het	G
nonsynonymous SNV	ADAM8	chr10	135089031	C	C	C	T_het	C
nonsynonymous SNV	ADAM8	chr10	135089077	G	G	A_het	G	G
nonsynonymous SNV	ADAMTS10	chr19	8649788	C	C	C	T_het	C
nonsynonymous SNV	ADAMTS12	chr5	33549350	C	G_het	C	C	C
nonsynonymous SNV	ADAMTS12	chr5	33549374	G	T_het	G	G	G
nonsynonymous SNV	ADAMTS12	chr5	33576529	G	G	G	A_het	G
nonsynonymous SNV	ADAMTS13	chr9	136302063	C	C	C	C	T_hom
nonsynonymous SNV	ADAMTS14	chr10	72509710	G	G	A_het	G	G
nonsynonymous SNV	ADAMTS15	chr11	130340859	G	G	G	A_hom	G
nonsynonymous SNV	ADAMTS16	chr5	5222966	C	C	T_het	C	C
nonsynonymous SNV	ADAMTS2	chr5	178552137	C	T_het	C	C	C
nonsynonymous SNV	ADAMTS20	chr12	43846199	G	G	G	G	T_het
nonsynonymous SNV	ADAMTS3	chr4	73156658	A	A	T_het	A	A
nonsynonymous SNV	ADAMTS7	chr15	79058445	G	G	G	G	A_het
nonsynonymous SNV	ADAMTS7	chr15	79069808	G	G	G	G	C_het
nonsynonymous SNV	ADAMTS7	chr15	79089074	C	C	C	T_het	C
nonsynonymous SNV	ADAMTS9	chr3	64532504	G	G	G	G	T_het
nonsynonymous SNV	ADAMTS9	chr3	64536648	C	C	C	C	T_het
nonsynonymous SNV	ADAMTSL3	chr15	84639309	G	G	G	G	A_het
nonsynonymous SNV	ADAMTSL3	chr15	84700108	C	C	T_het	C	C
nonsynonymous SNV	ADAMTSL4	chr1	150530548	C	C	C	G_hom	C
nonsynonymous SNV	ADAMTSL4	chr1	150531905	C	C	A_het	C	C
nonsynonymous SNV	ADARB2	chr10	1405407	G	G	G	C_het	G
nonsynonymous SNV	ADAT1	chr16	75646460	T	T	T	T	G_het
nonsynonymous SNV	ADCY10	chr1	167780075	C	C	T_het	C	C
nonsynonymous SNV	ADCY10	chr1	167817638	C	C	C	C	T_het
nonsynonymous SNV	ADCY10	chr1	167825449	T	T	C_het	T	T
nonsynonymous SNV	ADCY2	chr5	7706871	C	C	T_het	C	C
nonsynonymous SNV	ADCY3	chr2	25141705	C	C	T_het	C	C
nonsynonymous SNV	ADCY4	chr14	24791282	C	C	T_het	C	C
nonsynonymous SNV	ADCY4	chr14	24792285	G	G	G	T_het	G
nonsynonymous SNV	ADCY4	chr14	24800306	G	G	A_het	G	G
nonsynonymous SNV	ADCY6	chr12	49165507	C	C	T_het	C	C
nonsynonymous SNV	ADCY6	chr12	49167458	G	G	G	C_het	G

nonsynonymous SNV	ADCY7	chr16	50322157	G	G	A_het	G	G
nonsynonymous SNV	ADCY7	chr16	50324561	C	C	C	C	T_het
nonsynonymous SNV	ADCY9	chr16	4043415	T	T	T	C_het	T
nonsynonymous SNV	ADCY9	chr16	4165257	C	C	A_het	C	C
nonsynonymous SNV	ADIG	chr20	37209999	C	C	C	T_het	C
nonsynonymous SNV	ADNP2	chr18	77893552	A	A	G_het	A	A
nonsynonymous SNV	ADNP2	chr18	77896253	G	G	A_het	G	G
nonsynonymous SNV	ADRB3	chr8	37823194	G	G	G	G	C_hom
nonsynonymous SNV	ADRM1	chr20	60883442	G	G	A_het	G	G
nonsynonymous SNV	ADSSL1	chr14	105196458	G	G	A_het	G	G
stopgain	ADTRP	chr6	11723694	A	A	T_het	A	A
nonsynonymous SNV	AFF4	chr5	132219077	T	T	C_het	T	T
nonsynonymous SNV	AGAP1	chr2	236653408	G	A_het	G	G	G
nonsynonymous SNV	AGAP1	chr2	237029075	C	C	T_het	C	C
nonsynonymous SNV	AGAP6	chr10	51748584	A	A	G_het	A	A
nonsynonymous SNV	AGAP6	chr10	51748659	C	C	T_het	C	C
nonsynonymous SNV	AGBL2	chr11	47711820	A	A	G_het	A	A
nonsynonymous SNV	AGBL2	chr11	47721049	G	G	G	G	A_het
nonsynonymous SNV	AGPAT2	chr9	139569225	G	G	G	A_hom	G
nonsynonymous SNV	AGPHD1	chr15	78825711	T	T	C_het	T	T
nonsynonymous SNV	AGTR2	chrX	115304498	G	G	G	A_het	G
nonsynonymous SNV	AGTRAP	chr1	11807600	A	A	G_het	A	A
nonsynonymous SNV	AGXT	chr2	241812427	G	G	A_het	G	G
nonsynonymous SNV	AGXT2	chr5	35037136	G	G	G	A_het	G
stopgain	AHCTF1	chr1	247076642	G	G	A_het	G	G
nonsynonymous SNV	AHI1	chr6	135611646	T	T	C_het	T	T
nonsynonymous SNV	AHI1	chr6	135768282	C	C	T_het	C	C
nonsynonymous SNV	AHNAK	chr11	62296892	T	T	T	T	C_het
nonsynonymous SNV	AHNAK	chr11	62301521	C	C	T_het	C	C
nonsynonymous SNV	AHNAK2	chr14	105406235	A	C_het	A	A	A
nonsynonymous SNV	AHNAK2	chr14	105410383	T	T	T	C_het	C_het
nonsynonymous SNV	AHNAK2	chr14	105410461	G	G	G	C_het	G
nonsynonymous SNV	AHNAK2	chr14	105412066	C	T_het	T_hom	T_het	C
nonsynonymous SNV	AHNAK2	chr14	105412138	A	A	G_hom	G_het	A
nonsynonymous SNV	AHNAK2	chr14	105412260	G	G	G	G	C_het
nonsynonymous SNV	AHNAK2	chr14	105412561	C	T_het	T_hom	T_het	T_het
nonsynonymous SNV	AHNAK2	chr14	105413042	T	T	T	T	C_het
nonsynonymous SNV	AHNAK2	chr14	105413057	G	G	G	T_het	T_het
nonsynonymous SNV	AHNAK2	chr14	105413143	G	G	G	A_het	A_het
nonsynonymous SNV	AHNAK2	chr14	105415196	T	T	T	T	C_het
nonsynonymous SNV	AHNAK2	chr14	105415253	A	A	A	A	G_het
nonsynonymous SNV	AHNAK2	chr14	105415259	C	C	C	C	G_het
nonsynonymous SNV	AHNAK2	chr14	105415352	G	C_hom	G	C_hom	C_het
nonsynonymous SNV	AHNAK2	chr14	105415607	C	C	C	C	G_het
nonsynonymous SNV	AHNAK2	chr14	105416269	T	T	T	C_het	T

nonsynonymous SNV	AHNAK2	chr14	105416594	G	G	G	G	A_het
nonsynonymous SNV	AHNAK2	chr14	105417262	C	C	C	A_het	C
nonsynonymous SNV	AHNAK2	chr14	105418127	C	C	C	T_het	T_het
nonsynonymous SNV	AHNAK2	chr14	105418136	C	C	C	T_het	T_het
nonsynonymous SNV	AHNAK2	chr14	105418344	T	G_het	G_hom	G_het	G_hom
nonsynonymous SNV	AHNAK2	chr14	105418391	C	T_het	T_hom	T_het	T_hom
nonsynonymous SNV	AHNAK2	chr14	105418481	C	C	C	C	T_het
nonsynonymous SNV	AHNAK2	chr14	105418521	G	G	G	G	C_het
nonsynonymous SNV	AHNAK2	chr14	105418535	C	C	C	G_het	G_het
nonsynonymous SNV	AHRR	chr5	422955	C	G_het	G_het	G_het	G_het
nonsynonymous SNV	AHSG	chr3	186338564	C	C	T_het	C	C
nonsynonymous SNV	AHSG	chr3	186338634	G	G	G	A_hom	G
nonsynonymous SNV	AIFM3	chr22	21328141	G	G	A_het	G	G
nonsynonymous SNV	AIM1L	chr1	26671899	C	C	C	C	T_het
nonsynonymous SNV	AK094674	chr11	69242195	T	T	T	A_hom	T
nonsynonymous SNV	AK095498	chr2	105484717	G	G	A_het	G	G
nonsynonymous SNV	AK095633	chr1	205426024	C	T_het	C	C	C
nonsynonymous SNV	AK127224	chr5	177548520	C	C	C	C	A_het
nonsynonymous SNV	AK127846	chr19	53513548	G	G	A_het	G	G
nonsynonymous SNV	AK130852	chr11	134855496	C	G_hom	G_het	G_hom	C
nonsynonymous SNV	AK130852	chr11	134855517	T	T	C_het	T	T
nonsynonymous SNV	AK296947	chr20	17949301	C	C	C	C	G_het
nonsynonymous SNV	AK302092	chr15	82934807	C	C	C	G_het	C
nonsynonymous SNV	AK302511	chr16	90232832	A	A	C_het	A	A
nonsynonymous SNV	AK302511	chr16	90232893	C	C	A_het	C	C
nonsynonymous SNV	AK302545	chr22	20640820	T	C_het	T	T	T
nonsynonymous SNV	AK307192	chr20	48894793	T	C_hom	T	T	T
nonsynonymous SNV	AK308309	chr4	119434892	G	A_het	A_het	G	A_het
nonsynonymous SNV	AK308309	chr4	119435061	C	C	C	C	T_het
nonsynonymous SNV	AK310237	chr15	63340469	C	C	G_het	C	C
nonsynonymous SNV	AK311291	chr2	129625705	T	T	T	C_het	T
nonsynonymous SNV	AK8	chr9	135668085	G	G	G	G	A_hom
nonsynonymous SNV	AK9	chr6	109871399	T	T	T	T	G_het
nonsynonymous SNV	AK9	chr6	109885396	A	A	A	A	G_het
nonsynonymous SNV	AKAP6	chr14	33015633	A	A	A	G_het	A
nonsynonymous SNV	AKAP6	chr14	33291583	G	A_hom	A_het	G	G
nonsynonymous SNV	AKAP8	chr19	15472561	G	G	G	T_het	G
nonsynonymous SNV	AKAP9	chr7	91630603	G	G	G	C_het	G
nonsynonymous SNV	AKAP9	chr7	91671490	C	T_het	C	C	C
nonsynonymous SNV	AKNA	chr9	117124168	C	C	T_het	C	C
nonsynonymous SNV	AKNAD1	chr1	109506029	C	G_het	C	C	C
nonsynonymous SNV	AKR1C1	chr10	5009203	C	C	C	C	G_het
nonsynonymous SNV	AKR1C3	chr10	5141609	C	C	T_het	C	C
nonsynonymous SNV	AKR1E2	chr10	4872888	G	G	G	A_het	G
nonsynonymous SNV	ALCAM	chr3	105264129	G	A_het	G	G	G

stopgain	ALDH1L1	chr3	125833890	G	G	G	T_hom	G
nonsynonymous SNV	ALDH1L2	chr12	105440678	C	C	T_het	C	C
nonsynonymous SNV	ALDH3B2	chr11	67431200	G	G	G	G	T_het
nonsynonymous SNV	ALDH4A1	chr1	19199448	G	T_hom	G	G	G
nonsynonymous SNV	ALDOB	chr9	104187759	G	G	G	G	T_hom
nonsynonymous SNV	ALG2	chr9	101984160	C	C	G_het	C	C
nonsynonymous SNV	ALG3	chr3	183963134	C	C	T_het	C	C
nonsynonymous SNV	ALG3	chr3	183963381	T	C_het	T	T	T
nonsynonymous SNV	ALG8	chr11	77815059	A	G_het	A	A	A
nonsynonymous SNV	ALKBH6	chr19	36501502	C	C	T_het	C	C
nonsynonymous SNV	ALKBH8	chr11	107375679	C	C	T_het	C	C
nonsynonymous SNV	ALMS1	chr2	73682351	G	G	G	A_het	G
nonsynonymous SNV	ALMS1	chr2	73799632	C	G_hom	C	C	C
nonsynonymous SNV	ALOX15	chr17	4544904	A	A	A	A	G_hom
nonsynonymous SNV	ALOX15B	chr17	7948239	C	T_het	C	C	C
nonsynonymous SNV	ALPK1	chr4	113348759	A	A	G_het	A	A
nonsynonymous SNV	ALPK1	chr4	113353303	A	A	A	A	G_het
nonsynonymous SNV	ALPP	chr2	233246249	A	G_het	A	A	A
nonsynonymous SNV	ALS2CL	chr3	46717175	C	C	T_hom	C	C
nonsynonymous SNV	ALS2CR11	chr2	202352433	G	G	G	G	A_het
nonsynonymous SNV	ALS2CR8	chr2	203826059	C	C	T_het	C	C
nonsynonymous SNV	AMACR	chr5	33989565	A	A	A	G_het	A
nonsynonymous SNV	AMN	chr14	103396004	T	C_het	T	T	T
nonsynonymous SNV	AMT	chr3	49455040	C	C	C	T_hom	C
nonsynonymous SNV	ANAPC1	chr2	87414330	T	C_het	T	T	T
nonsynonymous SNV	ANGEL1	chr14	77275980	A	A	G_het	A	A
nonsynonymous SNV	ANGPT2	chr8	6360738	A	A	A	G_het	A
nonsynonymous SNV	ANGPTL4	chr19	8430886	G	G	G	G	A_het
nonsynonymous SNV	ANK2	chr4	114294537	G	A_het	G	G	G
nonsynonymous SNV	ANK3	chr10	61830584	T	T	T	C_het	T
nonsynonymous SNV	ANKAR	chr2	190559712	A	A	A	C_het	A
nonsynonymous SNV	ANKAR	chr2	190571797	A	A	A	G_het	A
nonsynonymous SNV	ANKH	chr5	14769138	C	C	T_het	C	C
nonsynonymous SNV	ANKLE1	chr19	17397481	G	G	T_het	G	G
nonsynonymous SNV	ANKLE1	chr19	17397483	G	G	T_het	G	G
nonsynonymous SNV	ANKMY1	chr2	241463515	T	T	T	T	A_het
nonsynonymous SNV	ANKRD12	chr18	9257697	T	T	T	A_hom	T
nonsynonymous SNV	ANKRD18B	chr9	33572324	G	T_hom	G	G	G
nonsynonymous SNV	ANKRD20A5P	chr18	14183734	A	G_het	A	G_het	A
nonsynonymous SNV	ANKRD20A5P	chr18	14183752	C	C	T_het	C	C
nonsynonymous SNV	ANKRD20A5P	chr18	14183755	C	C	T_het	C	C
nonsynonymous SNV	ANKRD20A5P	chr18	14183974	A	A	A	A	G_het
nonsynonymous SNV	ANKRD20A5P	chr18	14183978	T	T	T	T	C_het
nonsynonymous SNV	ANKRD20A5P	chr18	14183979	G	G	G	G	A_het
nonsynonymous SNV	ANKRD20A5P	chr18	14184005	C	C	C	C	T_het

nonsynonymous SNV	ANKRD20A5P	chr18	14184043	G	G	G	G	A_het
nonsynonymous SNV	ANKRD20A5P	chr18	14184079	A	A	A	A	C_het
nonsynonymous SNV	ANKRD20A5P	chr18	14184100	A	A	A	A	G_het
nonsynonymous SNV	ANKRD20A5P	chr18	14186414	G	G	G	G	A_het
nonsynonymous SNV	ANKRD20A5P	chr18	14186448	C	G_het	C	C	C
nonsynonymous SNV	ANKRD20A5P	chr18	14186450	G	A_het	G	G	A_het
nonsynonymous SNV	ANKRD20A5P	chr18	14187255	A	T_het	T_het	T_het	T_het
nonsynonymous SNV	ANKRD20A5P	chr18	14187273	C	G_het	G_het	G_het	G_het
nonsynonymous SNV	ANKRD20A5P	chr18	14187304	G	A_het	A_het	A_het	A_het
nonsynonymous SNV	ANKRD20A5P	chr18	14187309	C	C	C	C	T_het
stopgain	ANKRD20A5P	chr18	14187312	G	G	G	G	T_het
nonsynonymous SNV	ANKRD20A5P	chr18	14187327	T	T	G_het	G_het	T
nonsynonymous SNV	ANKRD24	chr19	4208788	C	C	T_het	C	C
stopgain	ANKRD30A	chr10	37419292	G	G	T_het	G	G
nonsynonymous SNV	ANKRD30A	chr10	37433975	T	G_het	T	T	T
nonsynonymous SNV	ANKRD30A	chr10	37433982	C	G_het	C	C	C
nonsynonymous SNV	ANKRD30A	chr10	37433983	G	T_het	G	G	G
nonsynonymous SNV	ANKRD30B	chr18	14779986	G	G	G	A_het	G
nonsynonymous SNV	ANKRD30B	chr18	14780019	G	G	G	T_het	G
nonsynonymous SNV	ANKRD33B	chr5	10649524	G	G	G	G	A_het
nonsynonymous SNV	ANKRD35	chr1	145561190	C	C	C	C	T_het
nonsynonymous SNV	ANKRD36	chr2	97808394	C	G_het	G_het	G_het	G_het
nonsynonymous SNV	ANKRD36	chr2	97817617	T	C_het	T	C_het	C_het
nonsynonymous SNV	ANKRD36	chr2	97817640	A	G_het	A	A	A
nonsynonymous SNV	ANKRD36	chr2	97817647	A	G_het	A	G_het	A
nonsynonymous SNV	ANKRD36	chr2	97824357	A	A	T_het	A	A
nonsynonymous SNV	ANKRD36	chr2	97827839	G	G	A_het	A_het	A_het
nonsynonymous SNV	ANKRD36	chr2	97830179	G	A_het	G	G	A_het
nonsynonymous SNV	ANKRD36	chr2	97830192	T	C_het	T	T	T
nonsynonymous SNV	ANKRD36	chr2	97845607	G	G	A_het	A_het	A_het
nonsynonymous SNV	ANKRD36	chr2	97845612	C	G_het	G_het	G_het	G_het
nonsynonymous SNV	ANKRD36	chr2	97845616	G	T_het	G	T_het	T_het
nonsynonymous SNV	ANKRD36	chr2	97860487	T	C_hom	C_het	C_het	T
nonsynonymous SNV	ANKRD36	chr2	97877478	G	G	A_het	A_het	G
nonsynonymous SNV	ANKRD36	chr2	97883094	T	T	G_het	G_het	T
nonsynonymous SNV	ANKRD36C	chr2	96525640	G	T_het	G	G	G
nonsynonymous SNV	ANKRD36C	chr2	96525652	T	T	C_het	C_het	T
nonsynonymous SNV	ANKRD36C	chr2	96525718	G	T_het	T_het	T_het	T_het
nonsynonymous SNV	ANKRD36C	chr2	96525723	C	T_het	T_het	T_het	T_het
nonsynonymous SNV	ANKRD36C	chr2	96525736	T	C_het	C_het	C_het	T
nonsynonymous SNV	ANKRD36C	chr2	96525750	T	A_het	A_het	A_het	A_het
nonsynonymous SNV	ANKRD36C	chr2	96525755	A	T_het	T_het	T_het	T_het
nonsynonymous SNV	ANKRD36C	chr2	96525768	G	A_het	A_het	A_het	G
stopgain	ANKRD36C	chr2	96525771	A	C_het	C_het	C_het	A
nonsynonymous SNV	ANKRD36C	chr2	96525794	C	A_het	A_het	A_het	C

nonsynonymous SNV	ANKRD42	chr11	82938881	C	C	T_het	C	C
nonsynonymous SNV	ANKRD52	chr12	56647518	G	G	G	G	A_het
nonsynonymous SNV	ANKRD53	chr2	71206262	C	C	T_het	C	C
nonsynonymous SNV	ANKRD53	chr2	71209218	G	G	G	G	A_het
nonsynonymous SNV	ANKRD66	chr6	46719833	T	C_het	T	T	T
nonsynonymous SNV	ANKS1A	chr6	35050498	C	C	T_het	C	C
nonsynonymous SNV	ANKUB1	chr3	149485802	G	G	A_het	G	G
nonsynonymous SNV	ANLN	chr7	36450696	G	G	G	G	A_hom
nonsynonymous SNV	ANO5	chr11	22271870	A	A	T_het	A	A
nonsynonymous SNV	ANO6	chr12	45782045	G	C_het	G	G	G
nonsynonymous SNV	ANO7	chr2	242151595	G	G	G	A_het	G
nonsynonymous SNV	ANO9	chr11	433934	C	C	T_het	C	C
nonsynonymous SNV	ANXA11	chr10	81917486	T	C_het	T	T	T
nonsynonymous SNV	ANXA3	chr4	79512701	A	A	G_het	A	A
nonsynonymous SNV	ANXA5	chr4	122605873	G	G	A_het	G	G
nonsynonymous SNV	ANXA9	chr1	150955582	A	A	G_het	A	A
nonsynonymous SNV	AOC2	chr17	41001713	C	C	A_het	C	C
nonsynonymous SNV	AOX1	chr2	201505884	G	G	G	T_het	G
nonsynonymous SNV	AP1AR	chr4	113189546	C	C	C	T_hom	C
nonsynonymous SNV	AP1M2	chr19	10697886	T	T	A_het	T	T
nonsynonymous SNV	AP2A1	chr19	50305680	C	C	C	C	T_het
nonsynonymous SNV	AP3B1	chr5	77425028	A	T_het	T_hom	T_hom	T_het
nonsynonymous SNV	AP4M1	chr7	99703080	C	C	T_het	C	C
nonsynonymous SNV	AP5M1	chr14	57746932	T	T	T	A_het	T
nonsynonymous SNV	APBA2	chr15	29346242	G	G	G	A_het	G
nonsynonymous SNV	APBB2	chr4	40818179	T	T	C_het	T	T
nonsynonymous SNV	APBB2	chr4	40892408	A	A	G_het	A	A
nonsynonymous SNV	APC	chr5	112175240	G	G	G	C_het	G
nonsynonymous SNV	APC	chr5	112177788	G	G	G	G	A_het
nonsynonymous SNV	APCDD1	chr18	10485735	G	G	G	A_hom	G
nonsynonymous SNV	APEH	chr3	49720002	G	G	G	G	C_hom
nonsynonymous SNV	APEX1	chr14	20924167	G	C_het	G	G	G
nonsynonymous SNV	APOA1BP	chr1	156563851	C	C	C	T_hom	C
nonsynonymous SNV	APOB	chr2	21234770	G	G	G	A_hom	G
nonsynonymous SNV	APOB	chr2	21255355	A	A	A	G_hom	A
nonsynonymous SNV	APOBEC1	chr12	7805154	G	G	A_het	G	G
nonsynonymous SNV	APOBEC3G	chr22	39474944	G	A_het	G	G	G
nonsynonymous SNV	APOH	chr17	64225484	A	A	A	A	G_hom
nonsynonymous SNV	APOL2	chr22	36624073	T	T	A_het	T	T
nonsynonymous SNV	APOL2	chr22	36624242	G	G	C_het	G	G
nonsynonymous SNV	APOPT1	chr14	104040444	G	G	A_het	G	G
nonsynonymous SNV	AQP12B	chr2	241621869	G	G	A_het	A_het	G
nonsynonymous SNV	AQP7	chr9	33385762	C	C	C	C	T_hom
nonsynonymous SNV	AQP7	chr9	33385863	G	G	T_het	G	G
nonsynonymous SNV	AQP7	chr9	33386146	C	A_het	A_het	A_het	A_het

nonsynonymous SNV	AQP7	chr9	33386167	G	C_het	C_het	C_het	C_het
nonsynonymous SNV	AQP7	chr9	33386465	A	G_het	A	G_het	A
nonsynonymous SNV	AQP7	chr9	33386510	C	T_het	T_het	C	C
nonsynonymous SNV	AQP7	chr9	33387047	T	G_het	T	T	T
nonsynonymous SNV	AQPEP	chr5	115341638	G	C_hom	C_hom	C_hom	C_hom
nonsynonymous SNV	AQR	chr15	35178780	G	G	G	G	A_het
nonsynonymous SNV	ARAP1	chr11	72404474	G	G	A_het	G	G
nonsynonymous SNV	ARGFXP2	chr17	30478207	G	A_het	G	G	G
nonsynonymous SNV	ARHGAP11A	chr15	32929324	A	A	G_het	A	A
nonsynonymous SNV	ARHGAP18	chr6	129950541	T	T	T	C_hom	T
stopgain	ARHGAP20	chr11	110450880	G	G	G	T_hom	G
nonsynonymous SNV	ARHGAP28	chr18	6824937	C	T_het	C	C	C
nonsynonymous SNV	ARHGAP28	chr18	6898532	C	C	C	T_hom	C
stopgain	ARHGAP28	chr18	6912121	G	G	T_het	G	G
nonsynonymous SNV	ARHGAP30	chr1	161026319	C	C	C	C	G_het
nonsynonymous SNV	ARHGAP32	chr11	128844223	C	C	C	T_hom	C
nonsynonymous SNV	ARHGAP42	chr11	100847560	A	A	A	C_hom	A
nonsynonymous SNV	ARHGAP9	chr12	57870663	G	G	G	G	T_het
nonsynonymous SNV	ARHGAP9	chr12	57872447	C	C	C	T_het	C
nonsynonymous SNV	ARHGEF10	chr8	1904975	C	C	T_het	C	C
nonsynonymous SNV	ARHGEF10L	chr1	18023778	G	G	A_het	G	G
nonsynonymous SNV	ARHGEF11	chr1	156911718	C	T_het	C	C	C
nonsynonymous SNV	ARHGEF18	chr19	7532208	C	C	C	A_het	C
nonsynonymous SNV	ARHGEF28	chr5	73179607	C	C	C	C	A_het
nonsynonymous SNV	ARHGEF39	chr9	35663069	C	C	T_het	C	C
nonsynonymous SNV	ARID1A	chr1	27105886	C	C	T_het	C	C
nonsynonymous SNV	ARID4B	chr1	235383177	T	T	T	C_hom	T
nonsynonymous SNV	ARID5B	chr10	63851054	C	C	C	C	T_het
nonsynonymous SNV	ARL10	chr5	175792660	T	T	G_het	T	T
nonsynonymous SNV	ARMC9	chr2	232156084	C	T_het	C	C	C
nonsynonymous SNV	ARMC9	chr2	232234812	G	G	G	A_het	G
nonsynonymous SNV	ARMCX4	chrX	100744685	C	G_het	C	C	C
nonsynonymous SNV	ARMCX4	chrX	100750187	A	A	A	G_hom	A
nonsynonymous SNV	ARRDC2	chr19	18120784	G	G	G	A_het	G
nonsynonymous SNV	ARSA	chr22	51065524	C	C	A_het	C	C
stopgain	ARSD	chrX	2833605	C	C	T_het	T_het	C
nonsynonymous SNV	ARSD	chrX	2833638	C	C	T_het	T_het	C
nonsynonymous SNV	ARSD	chrX	2833643	C	C	A_het	A_het	C
nonsynonymous SNV	ARSD	chrX	2835863	G	G	T_het	T_het	G
nonsynonymous SNV	ARSD	chrX	2835989	A	A	C_het	C_het	A
nonsynonymous SNV	ARSD	chrX	2835995	C	C	A_het	A_het	C
nonsynonymous SNV	ARSD	chrX	2836041	A	A	T_het	T_het	A
nonsynonymous SNV	ARSD	chrX	2836047	C	C	T_het	T_het	C
nonsynonymous SNV	ARSD	chrX	2836181	A	A	T_het	T_het	A
nonsynonymous SNV	ARSD	chrX	2836184	C	C	T_het	T_het	C

nonsynonymous SNV	ARSD	chrX	2836211	A	A	T_het	T_het	A
nonsynonymous SNV	ARSD	chrX	2836238	G	G	A_het	A_het	G
nonsynonymous SNV	ARSI	chr5	149676943	A	A	C_het	A	A
nonsynonymous SNV	ART5	chr11	3661052	T	T	T	C_hom	T
nonsynonymous SNV	ASAH1	chr8	17919819	C	C	T_het	C	C
stopgain	ASAP2	chr2	9525431	C	C	T_het	C	C
stopgain	ASAP3	chr1	23763445	G	G	A_het	G	G
nonsynonymous SNV	ASB9P1	chr15	93339677	C	C	C	G_het	C
nonsynonymous SNV	ASH1L	chr1	155451576	C	C	T_het	C	C
nonsynonymous SNV	ASH1L-AS1	chr1	155532118	G	G	T_het	G	G
nonsynonymous SNV	ASIC1	chr12	50452634	G	G	A_het	G	G
nonsynonymous SNV	ASPH	chr8	62559399	C	C	C	C	T_hom
nonsynonymous SNV	ASPM	chr1	197069932	C	C	C	C	A_het
nonsynonymous SNV	ASPSCR1	chr17	79970062	G	G	A_het	G	G
nonsynonymous SNV	ASTN1	chr1	176833427	T	T	T	C_hom	T
nonsynonymous SNV	ASXL1	chr20	31024561	T	T	T	C_het	T
nonsynonymous SNV	ASXL3	chr18	31319746	T	T	A_het	T	T
nonsynonymous SNV	ATAD3B	chr1	1418004	T	T	C_het	T	T
nonsynonymous SNV	ATAD3B	chr1	1420532	C	C	C	C	T_het
nonsynonymous SNV	ATAD3B	chr1	1431165	C	T_het	T_het	T_het	T_het
nonsynonymous SNV	ATAD5	chr17	29185324	A	A	A	G_hom	A
nonsynonymous SNV	ATCAY	chr19	3909536	T	T	C_het	T	T
nonsynonymous SNV	ATG14	chr14	55836620	G	G	A_het	G	G
nonsynonymous SNV	ATG16L2	chr11	72538269	A	A	G_het	A	A
nonsynonymous SNV	ATG2A	chr11	64662869	C	C	C	T_hom	C
nonsynonymous SNV	ATG2B	chr14	96795943	A	A	A	G_het	A
nonsynonymous SNV	ATG9B	chr7	150713042	C	T_het	C	C	C
nonsynonymous SNV	ATG9B	chr7	150721014	G	G	G	A_het	G
nonsynonymous SNV	ATG9B	chr7	150721090	G	G	G	A_het	G
nonsynonymous SNV	ATL2	chr2	38536627	C	T_het	C	C	C
nonsynonymous SNV	ATM	chr11	108206666	A	A	A	A	T_het
nonsynonymous SNV	ATN1	chr12	7045844	G	G	A_het	G	G
nonsynonymous SNV	ATOH7	chr10	69991051	C	A_het	C	C	C
nonsynonymous SNV	ATP10A	chr15	25924583	G	G	A_het	G	G
stopgain	ATP10B	chr5	160033805	G	G	A_het	G	G
nonsynonymous SNV	ATP10B	chr5	160059137	A	A	A	G_het	A
nonsynonymous SNV	ATP10B	chr5	160212667	T	C_het	T	T	T
nonsynonymous SNV	ATP11B	chr3	182583338	T	A_het	T	T	T
nonsynonymous SNV	ATP1A4	chr1	160125859	G	A_het	G	G	G
nonsynonymous SNV	ATP2A1	chr16	28909579	G	G	A_het	G	G
nonsynonymous SNV	ATP6V0D2	chr8	87111214	G	G	G	A_het	G
nonsynonymous SNV	ATP8A2	chr13	26501357	G	C_hom	G	G	G
nonsynonymous SNV	ATR	chr3	142232399	G	A_het	G	G	G
nonsynonymous SNV	ATRX	chrX	76856021	T	C_het	T	T	T
nonsynonymous SNV	ATRX	chrX	76938208	A	A	A	A	G_hom

nonsynonymous SNV	ATXN1	chr6	16327894	C	C	A_het	C	C
nonsynonymous SNV	ATXN1	chr6	16327900	C	C	A_het	C	C
nonsynonymous SNV	ATXN2	chr12	111963027	T	T	T	C_het	T
nonsynonymous SNV	ATXN2L	chr16	28842286	C	C	A_het	C	C
nonsynonymous SNV	AV2S1A1	chr14	22356411	G	G	T_het	G	G
nonsynonymous SNV	AVPR1A	chr12	63541279	A	A	A	A	T_het
nonsynonymous SNV	AX746638	chr19	36806431	C	T_het	T_het	T_het	T_het
nonsynonymous SNV	AX746638	chr19	36806475	A	T_het	T_het	T_het	T_het
nonsynonymous SNV	AX746734	chr19	14302254	C	C	T_het	C	C
nonsynonymous SNV	AX746750	chr10	104213777	G	G	A_het	G	G
nonsynonymous SNV	AX746830	chr6	53516820	T	C_het	T	T	T
nonsynonymous SNV	AX746839	chr3	194219729	C	C	C	T_hom	C
nonsynonymous SNV	AX747227	chr2	24427675	T	T	T	T	C_het
nonsynonymous SNV	AX747795	chr16	87738774	G	G	A_het	G	G
nonsynonymous SNV	AX747879	chr5	179078989	T	T	C_het	T	T
nonsynonymous SNV	AX747985	chr5	178949781	A	G_het	A	G_het	A
nonsynonymous SNV	AX747988	chr1	16862282	G	A_het	A_het	G	A_het
nonsynonymous SNV	AX747988	chr1	16862296	G	C_het	C_het	G	G
nonsynonymous SNV	AX747988	chr1	16862340	G	A_het	G	G	G
nonsynonymous SNV	AX747988	chr1	16862454	T	G_het	G_het	G_het	G_het
nonsynonymous SNV	AX747988	chr1	16862461	T	A_het	A_het	A_het	A_het
nonsynonymous SNV	AX747988	chr1	16862548	T	T	T	C_het	T
nonsynonymous SNV	AX748291	chr16	89345127	G	G	G	A_het	G
nonsynonymous SNV	AZI1	chr17	79172711	G	G	A_het	G	G
nonsynonymous SNV	B3GNT8	chr19	41932474	A	A	A	C_het	A
nonsynonymous SNV	B9D2	chr19	41860841	G	G	T_het	G	G
nonsynonymous SNV	BAGE	chr21	11098728	C	T_het	T_het	T_het	C
nonsynonymous SNV	BAGE3	chr21	11058226	G	C_het	C_het	C_het	C_het
nonsynonymous SNV	BAGE3	chr21	11058229	T	C_het	C_het	C_het	C_het
nonsynonymous SNV	BAGE3	chr21	11098704	A	G_het	G_het	A	A
nonsynonymous SNV	BAHD1	chr15	40751244	G	G	A_het	G	G
stopgain	BAI1	chr8	143603403	G	G	A_het	G	G
nonsynonymous SNV	BAI1	chr8	143614798	G	G	A_het	G	G
nonsynonymous SNV	BAI1	chr8	143625720	C	C	T_het	C	C
nonsynonymous SNV	BAI2	chr1	32205162	T	C_hom	T	T	T
nonsynonymous SNV	BAI2	chr1	32209874	C	C	A_het	C	C
nonsynonymous SNV	BAI3	chr6	69758151	A	A	G_het	A	A
nonsynonymous SNV	BAIAP2L2	chr22	38505152	G	G	A_het	G	G
nonsynonymous SNV	BAIAP3	chr16	1388618	G	G	A_het	G	G
nonsynonymous SNV	BAIAP3	chr16	1395842	C	A_het	C	C	C
nonsynonymous SNV	BASP1	chr5	17275410	G	G	G	G	A_het
nonsynonymous SNV	BASP1	chr5	17275486	G	G	A_het	G	G
nonsynonymous SNV	BASP1	chr5	17275894	C	C	T_het	C	C
nonsynonymous SNV	BAZ1B	chr7	72891569	G	G	A_het	G	G
nonsynonymous SNV	BAZ2B	chr2	160182374	T	T	C_het	T	T

nonsynonymous SNV	BAZ2B	chr2	160239116	T	T	T	T	A_het
nonsynonymous SNV	BBC3	chr19	47735796	T	T	T	A_het	T
nonsynonymous SNV	BBS12	chr4	123663086	C	C	A_het	C	C
nonsynonymous SNV	BC000869	chr9	66553512	C	G_hom	C	C	C
nonsynonymous SNV	BC032117	chr9	130455532	C	C	C	T_hom	C
stopgain	BC040304	chr4	141055441	G	G	T_het	G	G
nonsynonymous SNV	BC042649	chr12	131781417	A	A	G_het	A	A
nonsynonymous SNV	BC043570	chr15	29967699	C	C	T_het	C	C
nonsynonymous SNV	BC063788	chr7	75915187	C	T_het	C	C	C
nonsynonymous SNV	BC101079	chr15	102292689	A	A	G_het	A	A
nonsynonymous SNV	BC112340	chr22	18835298	T	C_het	C_het	C_het	T
nonsynonymous SNV	BC139719	chr16	90160948	G	G	G	A_het	G
nonsynonymous SNV	BC139719	chr16	90161065	A	T_hom	T_het	T_het	T_hom
nonsynonymous SNV	BC139719	chr16	90161167	C	C	T_het	T_het	C
nonsynonymous SNV	BC139719	chr16	90161192	C	C	C	C	G_het
nonsynonymous SNV	BC139719	chr16	90161231	T	T	G_het	T	T
nonsynonymous SNV	BC215	chr7	56879665	C	C	C	A_het	C
nonsynonymous SNV	BC215	chr7	56879674	A	A	A	C_het	A
nonsynonymous SNV	BC215	chr7	56880009	G	G	G	A_hom	G
nonsynonymous SNV	BCAR1	chr16	75271175	T	T	C_het	T	T
nonsynonymous SNV	BCAR3	chr1	94140470	A	T_hom	A	T_hom	A
nonsynonymous SNV	BCAS4	chr20	49492606	G	G	A_het	G	G
nonsynonymous SNV	BCL11A	chr2	60688270	C	C	C	T_het	C
nonsynonymous SNV	BCL7C	chr16	30904601	A	A	G_het	A	A
nonsynonymous SNV	BCL9L	chr11	118769171	T	C_het	T	T	T
nonsynonymous SNV	BCLAF1	chr6	136582497	G	T_het	T_het	T_het	T_het
nonsynonymous SNV	BCLAF1	chr6	136589425	G	G	G	T_het	G
nonsynonymous SNV	BCLAF1	chr6	136589448	C	A_het	C	C	C
nonsynonymous SNV	BCLAF1	chr6	136590640	A	C_het	C_het	C_het	C_het
nonsynonymous SNV	BCLAF1	chr6	136590698	C	T_het	C	T_het	T_het
nonsynonymous SNV	BCLAF1	chr6	136597281	A	T_het	T_het	T_het	T_het
nonsynonymous SNV	BCLAF1	chr6	136597288	A	C_het	C_het	C_het	C_het
nonsynonymous SNV	BCLAF1	chr6	136597449	G	C_het	G	G	G
nonsynonymous SNV	BCLAF1	chr6	136599822	C	G_het	G_het	G_het	G_het
nonsynonymous SNV	BCO2	chr11	112064634	C	C	T_het	C	C
nonsynonymous SNV	BCORL1	chrX	129148694	C	C	A_hom	C	C
nonsynonymous SNV	BCORL1	chrX	129156916	C	C	T_hom	C	C
nonsynonymous SNV	BEND3	chr6	107391566	C	C	T_het	C	C
nonsynonymous SNV	BEND7	chr10	13542069	T	T	T	T	A_het
nonsynonymous SNV	BIRC2	chr11	102248276	G	G	G	G	T_het
nonsynonymous SNV	BIRC6	chr2	32693842	G	G	A_het	G	G
nonsynonymous SNV	BIVM-ERCC5	chr13	103506108	T	T	C_het	T	T
nonsynonymous SNV	BMP2K	chr4	79747241	G	G	A_het	G	G
nonsynonymous SNV	BMP2K	chr4	79763578	C	C	T_het	C	C
nonsynonymous SNV	BMP3	chr4	81952637	T	A_het	T	T	T

nonsynonymous SNV	BMP8A	chr1	39988084	G	A_hom	A_het	G	A_het
nonsynonymous SNV	BMP8B	chr1	40230336	C	G_het	C	C	G_het
nonsynonymous SNV	BMPR2	chr2	203329658	G	G	A_het	G	G
nonsynonymous SNV	BNIP3	chr10	133787328	G	G	A_het	G	G
nonsynonymous SNV	BNIPL	chr1	151015564	G	G	A_het	G	G
nonsynonymous SNV	BOD1	chr5	173036394	C	C	C	T_het	C
nonsynonymous SNV	BOD1L1	chr4	13601647	T	T	T	T	C_het
nonsynonymous SNV	BOD1L1	chr4	13603981	T	T	T	C_hom	T
nonsynonymous SNV	BOD1L1	chr4	13610209	G	G	A_het	G	G
nonsynonymous SNV	BOLL	chr2	198593273	A	A	A	A	G_het
nonsynonymous SNV	BPI	chr20	36917522	C	C	C	C	A_hom
nonsynonymous SNV	BPI	chr20	36952351	G	G	A_het	G	G
nonsynonymous SNV	BPI	chr20	36964056	G	G	G	G	A_hom
nonsynonymous SNV	BPIFB4	chr20	31671214	C	C	C	T_het	C
nonsynonymous SNV	BPTF	chr17	65977554	T	G_het	T	T	T
nonsynonymous SNV	BRCA1	chr17	41199702	C	C	T_het	C	C
nonsynonymous SNV	BRCA2	chr13	32907398	C	C	T_het	C	C
nonsynonymous SNV	BRCA2	chr13	32914371	G	G	G	A_het	G
nonsynonymous SNV	BRD1	chr22	50216758	T	C_het	T	T	T
nonsynonymous SNV	BRD8	chr5	137495798	C	C	T_het	C	C
nonsynonymous SNV	BRD9	chr5	878525	T	T	A_het	A_het	T
nonsynonymous SNV	BRICD5	chr16	2259625	G	G	G	A_het	G
nonsynonymous SNV	BRWD1	chr21	40568366	C	C	T_het	C	C
nonsynonymous SNV	BRWD1	chr21	40574434	T	C_het	T	T	T
nonsynonymous SNV	BSDC1	chr1	32846847	G	G	A_het	G	G
nonsynonymous SNV	BSN	chr3	49691434	C	C	T_hom	C	C
nonsynonymous SNV	BSN	chr3	49698498	G	G	G	A_hom	G
nonsynonymous SNV	BSND	chr1	55472924	G	G	G	A_hom	G
nonsynonymous SNV	BTBD16	chr10	124089009	G	T_het	G	G	G
nonsynonymous SNV	BTBD2	chr19	1987562	C	A_het	C	C	C
nonsynonymous SNV	BTN1A1	chr6	26501899	C	C	C	G_hom	C
nonsynonymous SNV	BTN3A2	chr6	26370597	G	G	T_het	G	G
stopgain	BTN3A3	chr6	26446011	G	G	A_het	G	G
nonsynonymous SNV	BTN3A3	chr6	26452164	C	C	T_het	C	C
nonsynonymous SNV	BTRC	chr10	103310574	C	C	C	C	A_het
nonsynonymous SNV	BX648961	chr5	172294194	G	T_hom	T_het	T_het	T_het
nonsynonymous SNV	C10orf114	chr10	21785739	T	T	T	T	C_het
nonsynonymous SNV	C10orf12	chr10	98742088	T	T	T	C_het	T
nonsynonymous SNV	C10orf71	chr10	50531644	A	A	G_het	A	A
nonsynonymous SNV	C10orf71	chr10	50533751	C	C	T_het	C	C
nonsynonymous SNV	C10orf90	chr10	128147737	C	C	A_het	C	C
nonsynonymous SNV	C11orf63	chr11	122756666	C	C	T_het	C	C
nonsynonymous SNV	C11orf71	chr11	114262307	A	A	A	A	T_het
nonsynonymous SNV	C11orf95	chr11	63532023	C	C	T_het	C	C
nonsynonymous SNV	C12orf42	chr12	103872172	T	G_het	G_hom	T	T

nonsynonymous SNV	C12orf49	chr12	117175643	C	C	C	A_het	C
nonsynonymous SNV	C14orf169	chr14	73957772	A	G_het	G_hom	G_hom	G_het
nonsynonymous SNV	C14orf182	chr14	50472360	A	A	A	A	C_het
nonsynonymous SNV	C14orf183	chr14	50550688	C	C	A_het	C	C
nonsynonymous SNV	C14orf37	chr14	58563642	T	T	C_het	T	T
stopgain	C14orf37	chr14	58758365	G	G	G	G	A_het
nonsynonymous SNV	C14orf39	chr14	60951790	C	T_het	C	C	C
nonsynonymous SNV	C14orf79	chr14	105461050	C	C	T_het	C	C
nonsynonymous SNV	C15orf27	chr15	76494569	A	A	A	G_het	A
nonsynonymous SNV	C15orf62	chr15	41062940	C	C	C	G_het	C
nonsynonymous SNV	C16orf62	chr16	19710890	C	C	T_het	C	C
nonsynonymous SNV	C16orf91	chr16	1470497	A	A	A	G_het	A
nonsynonymous SNV	C16orf95	chr16	87339450	C	C	T_het	C	C
nonsynonymous SNV	C17orf102	chr17	32906076	G	G	G	G	A_het
nonsynonymous SNV	C17orf66	chr17	34190069	C	A_het	C	C	C
nonsynonymous SNV	C18orf21	chr18	33557490	C	C	C	T_hom	C
nonsynonymous SNV	C18orf8	chr18	21100182	A	A	A	G_hom	A
nonsynonymous SNV	C19orf45	chr19	7566069	G	A_het	G	G	G
nonsynonymous SNV	C19orf54	chr19	41255705	T	T	T	C_het	T
nonsynonymous SNV	C1GALT1	chr7	7274054	T	T	T	G_het	T
nonsynonymous SNV	C1orf127	chr1	11008369	C	C	C	T_hom	C
nonsynonymous SNV	C1orf229	chr1	247275054	G	G	G	G	A_het
nonsynonymous SNV	C1orf27	chr1	186368120	A	A	T_het	A	A
nonsynonymous SNV	C1orf35	chr1	228290033	T	C_hom	T	T	T
nonsynonymous SNV	C1orf86	chr1	2126139	C	C	C	G_hom	G_hom
stopgain	C1QTNF9	chr13	24893015	C	C	C	C	T_hom
nonsynonymous SNV	C1QTNF9	chr13	24895559	A	G_hom	G_hom	A	A
nonsynonymous SNV	C1QTNF9B	chr13	24468711	G	G	G	G	C_het
nonsynonymous SNV	C1R	chr12	7187978	G	G	G	G	A_het
nonsynonymous SNV	C20orf203	chr20	31238320	G	G	G	A_het	G
nonsynonymous SNV	C20orf85	chr20	56728608	G	G	G	A_het	G
nonsynonymous SNV	C2CD3	chr11	73824746	G	G	G	A_het	G
nonsynonymous SNV	C2CD3	chr11	73824747	T	T	T	A_het	T
nonsynonymous SNV	C2CD3	chr11	73824748	C	C	C	A_het	C
nonsynonymous SNV	C2CD4A	chr15	62360485	C	G_hom	C	G_het	C
nonsynonymous SNV	C2CD4C	chr19	407463	G	A_het	G	G	G
nonsynonymous SNV	C2orf16	chr2	27804427	G	G	A_het	G	G
nonsynonymous SNV	C2orf53	chr2	27360410	C	C	T_het	C	C
nonsynonymous SNV	C2orf53	chr2	27360878	C	C	C	C	T_het
nonsynonymous SNV	C2orf54	chr2	241827754	G	T_het	G	G	G
nonsynonymous SNV	C2orf57	chr2	232457921	C	C	C	T_het	C
nonsynonymous SNV	C3orf52	chr3	111831909	G	G	G	C_hom	G
nonsynonymous SNV	C4orf21	chr4	113469473	G	G	G	G	A_het
nonsynonymous SNV	C4orf21	chr4	113539198	T	T	A_het	T	T
nonsynonymous SNV	C4orf21	chr4	113539540	G	G	G	G	A_het

nonsynonymous SNV	C4orf32	chr4	113066857	C	C	C	G_hom	C
nonsynonymous SNV	C4orf45	chr4	159836467	C	T_het	C	C	C
nonsynonymous SNV	C4orf51	chr4	146601588	A	A	A	G_hom	A
nonsynonymous SNV	C5orf45	chr5	179280379	T	C_hom	C_hom	C_hom	C_het
nonsynonymous SNV	C5orf51	chr5	41909851	A	A	A	G_het	A
nonsynonymous SNV	C5orf58	chr5	169661994	G	C_het	G	C_hom	C_hom
nonsynonymous SNV	C5orf60	chr5	179071925	G	G	A_het	G	G
nonsynonymous SNV	C5orf60	chr5	179071958	C	C	G_het	C	C
nonsynonymous SNV	C6	chr5	41153914	T	T	T	C_het	T
nonsynonymous SNV	C6	chr5	41186221	G	G	G	A_het	G
nonsynonymous SNV	C7orf10	chr7	40234654	T	T	T	T	A_het
nonsynonymous SNV	C8orf48	chr8	13425059	C	C	C	G_het	C
nonsynonymous SNV	C8orf58	chr8	22458457	C	G_het	C	C	C
nonsynonymous SNV	C9orf3	chr9	97686308	G	A_hom	G	G	G
nonsynonymous SNV	C9orf64	chr9	86559900	T	T	T	C_hom	T
nonsynonymous SNV	C9orf69	chr9	139008494	G	G	A_het	G	G
nonsynonymous SNV	CABIN1	chr22	24456570	C	C	T_het	C	C
nonsynonymous SNV	CABP2	chr11	67287354	C	C	C	C	T_het
nonsynonymous SNV	CACNA1A	chr19	13409404	C	C	C	T_het	C
nonsynonymous SNV	CACNA1A	chr19	13428055	G	G	G	G	A_het
nonsynonymous SNV	CACNA1B	chr9	140777299	G	T_het	T_het	T_het	G
nonsynonymous SNV	CACNA1B	chr9	140777306	C	G_het	G_het	G_het	G_het
stopgain	CACNA1C	chr12	2558145	C	C	T_het	C	C
nonsynonymous SNV	CACNA1H	chr16	1268979	C	C	T_het	C	C
nonsynonymous SNV	CACNA2D4	chr12	1969336	T	T	C_het	T	T
nonsynonymous SNV	CACNB1	chr17	37342772	C	C	T_het	C	C
nonsynonymous SNV	CACNB1	chr17	37343772	G	G	A_het	G	G
nonsynonymous SNV	CACNB3	chr12	49221416	C	C	T_het	C	C
nonsynonymous SNV	CACNB3	chr12	49221495	G	G	G	G	A_hom
nonsynonymous SNV	CACNG6	chr19	54515399	C	C	C	A_het	C
nonsynonymous SNV	CACTIN	chr19	3613058	G	G	G	A_het	G
nonsynonymous SNV	CACYBP	chr1	174973942	G	G	A_het	G	G
nonsynonymous SNV	CAD	chr2	27445170	A	G_het	A	A	A
nonsynonymous SNV	CADM3	chr1	159163314	C	T_het	C	C	C
stopgain	CADPS2	chr7	122076414	C	C	A_het	C	C
nonsynonymous SNV	CALCOCO2	chr17	46939658	C	C	C	G_hom	C
nonsynonymous SNV	CALHM3	chr10	105238546	G	G	A_het	G	G
nonsynonymous SNV	CALR3	chr19	16589986	G	G	T_het	G	G
nonsynonymous SNV	CAMK1D	chr10	12856291	G	G	G	G	A_het
nonsynonymous SNV	CAMK2A	chr5	149610876	C	C	A_het	C	C
nonsynonymous SNV	CAMKK2	chr12	121711962	C	T_het	C	C	C
nonsynonymous SNV	CAMSAP3	chr19	7676736	C	C	C	T_het	C
nonsynonymous SNV	CAMTA1	chr1	7797375	C	C	T_het	C	C
nonsynonymous SNV	CAPG	chr2	85629256	T	C_het	T	T	T
nonsynonymous SNV	CAPN1	chr11	64950432	G	G	G	G	A_het

nonsynonymous SNV	CAPN10	chr2	241526428	C	C	G_het	C	C
nonsynonymous SNV	CAPN10	chr2	241536279	C	T_het	C	C	C
nonsynonymous SNV	CAPN6	chrX	110491920	C	C	T_hom	C	C
nonsynonymous SNV	CAPSL	chr5	35910601	A	A	A	A	T_het
nonsynonymous SNV	CARD11	chr7	2977549	G	G	A_het	G	G
nonsynonymous SNV	CARD14	chr17	78157811	T	T	G_het	T	T
nonsynonymous SNV	CARD9	chr9	139265409	C	C	C	T_hom	C
nonsynonymous SNV	CARS	chr11	3037027	G	G	G	A_hom	G
nonsynonymous SNV	CASC5	chr15	40916632	T	T	T	T	A_het
nonsynonymous SNV	CASK	chrX	41414866	G	T_het	G	G	G
nonsynonymous SNV	CASKIN1	chr16	2230133	C	C	T_het	C	C
nonsynonymous SNV	CASKIN1	chr16	2231025	T	C_het	T	T	T
nonsynonymous SNV	CASP10	chr2	202074086	A	A	T_het	A	A
nonsynonymous SNV	CASP5	chr11	104869743	T	T	C_het	T	T
nonsynonymous SNV	CASP7	chr10	115457287	G	G	G	A_het	G
nonsynonymous SNV	CAT	chr11	34473746	C	C	T_het	C	C
nonsynonymous SNV	CATSPER3	chr5	134343662	G	G	A_het	G	G
nonsynonymous SNV	CATSPERD	chr19	5754231	A	A	A	G_het	A
nonsynonymous SNV	CBLC	chr19	45284495	C	C	C	T_het	C
nonsynonymous SNV	CBR3	chr21	37518679	A	A	A	T_het	A
nonsynonymous SNV	CBWD1	chr9	172167	C	C	C	T_het	T_het
nonsynonymous SNV	CBWD2	chr2	114220062	C	T_het	C	C	C
nonsynonymous SNV	CBX7	chr22	39530648	T	C_het	T	T	T
nonsynonymous SNV	CC2D1B	chr1	52821917	C	C	C	G_hom	C
nonsynonymous SNV	CC2D1B	chr1	52822724	C	C	C	G_hom	C
nonsynonymous SNV	CC2D2A	chr4	15516333	G	G	G	A_hom	G
nonsynonymous SNV	CCDC102B	chr18	66504168	T	T	G_het	T	T
nonsynonymous SNV	CCDC102B	chr18	66541943	A	A	A	A	C_hom
nonsynonymous SNV	CCDC114	chr19	48800301	C	C	C	T_het	C
nonsynonymous SNV	CCDC12	chr3	46966898	T	T	C_hom	T	T
nonsynonymous SNV	CCDC120	chrX	48924896	C	A_het	C	C	C
nonsynonymous SNV	CCDC134	chr22	42209318	C	C	C	C	T_hom
nonsynonymous SNV	CCDC135	chr16	57741426	G	G	G	A_het	G
nonsynonymous SNV	CCDC14	chr3	123650179	T	T	T	T	G_het
nonsynonymous SNV	CCDC14	chr3	123665859	T	T	T	T	C_het
nonsynonymous SNV	CCDC141	chr2	179751485	T	T	T	G_het	T
nonsynonymous SNV	CCDC146	chr7	76797103	G	G	G	A_het	G
nonsynonymous SNV	CCDC147	chr10	106139799	G	G	G	A_het	G
nonsynonymous SNV	CCDC149	chr4	24878255	A	A	T_het	A	A
nonsynonymous SNV	CCDC151	chr19	11537603	G	A_het	G	G	G
nonsynonymous SNV	CCDC157	chr22	30769727	G	G	G	A_hom	G
nonsynonymous SNV	CCDC157	chr22	30772309	C	A_het	C	C	C
nonsynonymous SNV	CCDC163P	chr1	45965209	T	T	C_het	T	T
nonsynonymous SNV	CCDC168	chr13	103390086	C	C	C	T_het	C
nonsynonymous SNV	CCDC168	chr13	103402547	A	A	G_het	A	A

nonsynonymous SNV	CCDC17	chr1	46086963	G	G	A_het	G	G
nonsynonymous SNV	CCDC171	chr9	15623392	G	G	A_het	G	G
nonsynonymous SNV	CCDC171	chr9	15744637	A	T_hom	A	A	A
nonsynonymous SNV	CCDC178	chr18	30791889	C	G_het	C	C	C
nonsynonymous SNV	CCDC178	chr18	30903516	G	G	G	C_hom	G
nonsynonymous SNV	CCDC3	chr10	12940477	G	G	A_het	G	G
nonsynonymous SNV	CCDC39	chr3	180377529	G	G	C_het	G	G
nonsynonymous SNV	CCDC40	chr17	78022378	C	C	G_het	C	G_hom
nonsynonymous SNV	CCDC41	chr12	94725534	T	T	G_het	T	T
nonsynonymous SNV	CCDC61	chr19	46520506	G	G	C_het	G	G
nonsynonymous SNV	CCDC62	chr12	123286335	C	C	T_het	C	C
nonsynonymous SNV	CCDC65	chr12	49298780	A	A	A	G_hom	A
nonsynonymous SNV	CCDC70	chr13	52439669	G	G	A_het	G	G
nonsynonymous SNV	CCDC74A	chr2	132288362	T	T	T	C_het	C_het
nonsynonymous SNV	CCDC74A	chr2	132290621	C	C	C	T_het	C
nonsynonymous SNV	CCDC74B	chr2	130899804	T	T	T	C_het	T
nonsynonymous SNV	CCDC74B	chr2	130900779	T	C_het	T	T	T
nonsynonymous SNV	CCDC81	chr11	86123573	C	C	C	A_hom	C
nonsynonymous SNV	CCDC87	chr11	66359489	C	T_het	C	C	C
nonsynonymous SNV	CCDC88B	chr11	64122821	T	T	C_het	T	T
nonsynonymous SNV	CCER1	chr12	91348056	C	C	T_het	C	C
nonsynonymous SNV	CCNE1	chr19	30312915	C	C	T_het	C	C
nonsynonymous SNV	CCNG2	chr4	78079695	T	T	G_het	T	T
nonsynonymous SNV	CCNH	chr5	86700748	G	G	A_het	G	G
nonsynonymous SNV	CCSER1	chr4	91229709	G	G	G	G	T_het
nonsynonymous SNV	CCSER2	chr10	86130899	C	C	T_het	C	C
nonsynonymous SNV	CCZ1B	chr7	6844574	C	C	G_het	C	C
nonsynonymous SNV	CD109	chr6	74496994	T	T	T	G_hom	T
nonsynonymous SNV	CD163	chr12	7636097	C	C	C	C	A_het
nonsynonymous SNV	CD163L1	chr12	7528404	C	C	T_het	C	C
nonsynonymous SNV	CD177	chr19	43865692	G	A_het	G	A_het	A_het
nonsynonymous SNV	CD24	chrY	21154426	G	A_hom	A_het	G	G
nonsynonymous SNV	CD24	chrY	21154466	T	A_hom	A_het	A_het	A_hom
nonsynonymous SNV	CD34	chr1	208061249	G	G	A_het	G	G
nonsynonymous SNV	CD36	chr7	80285893	A	A	A	G_het	A
nonsynonymous SNV	CD48	chr1	160650895	A	A	A	A	G_het
nonsynonymous SNV	CD5	chr11	60886850	A	A	A	A	G_het
nonsynonymous SNV	CD82	chr11	44636840	G	G	G	G	A_het
nonsynonymous SNV	CDADC1	chr13	49830000	C	C	C	A_het	C
nonsynonymous SNV	CDAN1	chr15	43018518	C	C	T_het	C	C
nonsynonymous SNV	CDC14C	chr7	48965308	G	A_het	G	G	G
nonsynonymous SNV	CDC16	chr13	115002283	A	A	A	G_het	A
nonsynonymous SNV	CDC20	chr1	43828711	C	C	T_het	C	C
nonsynonymous SNV	CDC20B	chr5	54468450	T	C_het	T	T	T
nonsynonymous SNV	CDC27	chr17	45234417	A	A	G_het	A	A

nonsynonymous SNV	CDC27	chr17	45234420	T	T	G_het	T	T
nonsynonymous SNV	CDC42BPB	chr14	103440459	T	T	C_het	T	T
nonsynonymous SNV	CDC42BPG	chr11	64607024	C	C	T_het	C	C
nonsynonymous SNV	CDC42EP5	chr19	54976356	C	C	T_het	C	C
nonsynonymous SNV	CDCA5	chr11	64847136	C	C	C	T_hom	C
nonsynonymous SNV	CDCP2	chr1	54605557	G	G	G	T_hom	G
nonsynonymous SNV	CDH12	chr5	21752277	C	C	C	G_het	C
nonsynonymous SNV	CDH13	chr16	83704419	G	G	A_het	G	G
nonsynonymous SNV	CDH15	chr16	89256813	C	C	T_het	C	C
nonsynonymous SNV	CDH19	chr18	64172434	C	G_het	C	C	C
nonsynonymous SNV	CDH22	chr20	44839143	C	C	C	A_het	C
nonsynonymous SNV	CDH23	chr10	73563128	G	G	A_het	G	G
nonsynonymous SNV	CDH24	chr14	23517629	C	C	C	T_het	C
nonsynonymous SNV	CDH26	chr20	58562571	C	C	G_het	C	C
nonsynonymous SNV	CDH26	chr20	58564146	A	A	G_het	A	A
nonsynonymous SNV	CDH26	chr20	58576432	A	A	G_het	A	A
nonsynonymous SNV	CDH4	chr20	59829921	G	G	G	A_het	G
nonsynonymous SNV	CDH5	chr16	66434707	A	A	A	A	G_het
nonsynonymous SNV	CDH5	chr16	66436716	C	C	T_het	C	C
stopgain	CDH6	chr5	31316383	G	G	T_het	G	G
nonsynonymous SNV	CDH7	chr18	63547855	C	G_het	C	C	C
nonsynonymous SNV	CDHR1	chr10	85961608	C	C	C	C	T_het
nonsynonymous SNV	CDHR2	chr5	176008503	C	C	C	C	T_het
nonsynonymous SNV	CDHR3	chr7	105665004	C	A_het	C	C	C
nonsynonymous SNV	CDK11A	chr1	1650797	A	G_het	G_het	G_het	G_het
nonsynonymous SNV	CDK11A	chr1	1650832	A	G_het	G_het	G_het	G_het
nonsynonymous SNV	CDK11B	chr1	1571841	A	A	A	C_het	A
nonsynonymous SNV	CDK11B	chr1	1650787	T	C_het	C_het	C_het	C_het
nonsynonymous SNV	CDK13	chr7	39991306	C	G_het	C	C	C
nonsynonymous SNV	CDK13	chr7	40127879	G	G	G	A_het	G
nonsynonymous SNV	CDK18	chr1	205493374	G	G	G	A_hom	G
nonsynonymous SNV	CDK8	chr13	26959348	C	C	T_het	C	C
nonsynonymous SNV	CDKL3	chr5	133642326	T	C_hom	T	T	T
nonsynonymous SNV	CDKN1A	chr6	36651889	C	C	C	T_hom	C
nonsynonymous SNV	CDON	chr11	125830970	A	T_het	T_hom	T_hom	T_hom
nonsynonymous SNV	CDON	chr11	125880362	C	C	C	T_hom	C
stopgain	CDRT15	chr17	14140081	G	G	G	A_het	G
nonsynonymous SNV	CDYL2	chr16	80718728	C	C	T_het	C	C
nonsynonymous SNV	CEACAM16	chr19	45206676	G	T_het	G	G	G
nonsynonymous SNV	CENPF	chr1	214813665	A	T_het	A	A	A
nonsynonymous SNV	CENPF	chr1	214813782	A	G_het	G_het	A	G_het
nonsynonymous SNV	CENPF	chr1	214815492	A	G_het	A	A	A
nonsynonymous SNV	CENPF	chr1	214820261	C	C	C	C	T_het
nonsynonymous SNV	CEP112	chr17	63923713	C	C	C	C	T_hom
nonsynonymous SNV	CEP120	chr5	122751793	G	G	T_het	G	G

nonsynonymous SNV	CEP164	chr11	117280522	A	A	A	C_het	A
nonsynonymous SNV	CEP170	chr1	243329049	G	A_het	G	G	A_het
nonsynonymous SNV	CEP290	chr12	88530476	T	C_het	T	T	T
nonsynonymous SNV	CEP350	chr1	180064805	C	C	C	C	G_het
nonsynonymous SNV	CEP44	chr4	175229940	T	T	C_het	T	T
nonsynonymous SNV	CEP57L1	chr6	109466452	G	G	G	T_hom	G
nonsynonymous SNV	CEP68	chr2	65296663	C	C	T_het	C	C
nonsynonymous SNV	CEP68	chr2	65299420	T	C_het	T	T	T
nonsynonymous SNV	CEP76	chr18	12686398	C	T_het	C	C	C
nonsynonymous SNV	CEP78	chr9	80863269	G	G	G	C_hom	G
nonsynonymous SNV	CEP89	chr19	33392223	A	A	G_het	A	A
nonsynonymous SNV	CEP89	chr19	33444556	T	C_het	T	T	T
nonsynonymous SNV	CEP89	chr19	33444576	C	T_het	C	C	C
nonsynonymous SNV	CEP89	chr19	33444588	T	G_het	T	T	T
nonsynonymous SNV	CEP95	chr17	62510449	C	C	T_het	C	C
nonsynonymous SNV	CEPT1	chr1	111726868	C	C	T_het	C	C
nonsynonymous SNV	CES1	chr16	55862691	G	A_het	A_het	A_het	G
nonsynonymous SNV	CES1	chr16	55862791	T	C_het	C_het	C_het	T
nonsynonymous SNV	CES1	chr16	55862824	C	T_het	T_het	T_het	C
nonsynonymous SNV	CES1	chr16	55862883	C	A_het	A_het	A_het	C
nonsynonymous SNV	CFI	chr4	110682728	T	T	T	T	G_het
nonsynonymous SNV	CFL1	chr11	65623512	C	C	T_het	C	C
nonsynonymous SNV	CFTR	chr7	117188736	C	C	A_het	C	C
nonsynonymous SNV	CFTR	chr7	117188750	C	C	T_het	C	C
nonsynonymous SNV	CFTR	chr7	117188797	A	A	G_het	A	A
nonsynonymous SNV	CGB7	chr19	49558216	C	C	C	C	T_het
nonsynonymous SNV	CGN	chr1	151502966	C	C	G_het	C	C
nonsynonymous SNV	CGN	chr1	151509694	C	C	A_het	C	C
nonsynonymous SNV	CHAT	chr10	50824106	C	C	T_het	C	C
nonsynonymous SNV	CHAT	chr10	50828646	G	G	T_het	G	G
nonsynonymous SNV	CHD1	chr5	98228403	T	T	T	A_het	T
nonsynonymous SNV	CHD1L	chr1	146763190	T	C_het	T	T	T
nonsynonymous SNV	CHD4	chr12	6700688	C	C	T_het	C	C
nonsynonymous SNV	CHD6	chr20	40050675	G	G	A_het	G	G
nonsynonymous SNV	CHD7	chr8	61655042	G	G	G	A_het	G
nonsynonymous SNV	CHD8	chr14	21868658	C	T_het	C	C	C
nonsynonymous SNV	CHDH	chr3	53856613	G	G	G	A_hom	G
nonsynonymous SNV	CHDH	chr3	53857989	C	C	T_hom	C	C
nonsynonymous SNV	CHEK2	chr22	29091147	A	A	A	C_hom	A
nonsynonymous SNV	CHIA	chr1	111857924	G	G	G	G	A_het
nonsynonymous SNV	CHID1	chr11	899344	G	G	A_het	G	G
nonsynonymous SNV	CHKA	chr11	67842185	G	A_het	G	G	G
nonsynonymous SNV	CHL1	chr3	430965	G	G	G	C_hom	G
nonsynonymous SNV	CHRNA3	chr15	78909414	C	C	T_het	C	C
nonsynonymous SNV	CHRNA4	chr20	61981288	C	C	T_het	C	C

nonsynonymous SNV	CHRNA7	chr15	32322882	C	C	C	C	T_het
nonsynonymous SNV	CHRND	chr2	233393032	C	C	T_het	C	C
nonsynonymous SNV	CHST15	chr10	125769753	T	T	T	C_het	T
nonsynonymous SNV	CHST2	chr3	142840872	C	C	T_het	C	C
nonsynonymous SNV	CHTF18	chr16	839627	T	T	T	C_het	T
nonsynonymous SNV	CHTF18	chr16	846757	C	A_het	C	C	C
nonsynonymous SNV	CHUK	chr10	101960465	G	G	G	G	A_het
nonsynonymous SNV	CIB1	chr15	90775572	G	G	G	G	A_het
nonsynonymous SNV	CILP	chr15	65489880	T	T	G_het	T	T
nonsynonymous SNV	CILP2	chr19	19655515	A	A	G_het	A	A
stopgain	CIR1	chr2	175213353	G	G	G	G	A_het
nonsynonymous SNV	CIR1	chr2	175213527	T	T	T	C_het	T
nonsynonymous SNV	CIRH1A	chr16	69170707	G	G	A_het	G	G
stopgain	CKAP5	chr11	46797924	G	A_het	G	G	G
nonsynonymous SNV	CLASRP	chr19	45567668	C	C	A_het	C	C
nonsynonymous SNV	CLCN2	chr3	184071983	C	C	C	T_het	C
nonsynonymous SNV	CLEC17A	chr19	14698451	T	T	T	T	G_het
nonsynonymous SNV	CLEC18A	chr16	69988364	C	C	T_het	C	C
nonsynonymous SNV	CLEC18B	chr16	74443490	C	C	C	C	T_het
nonsynonymous SNV	CLEC18B	chr16	74447514	T	C_het	C_het	C_het	C_het
nonsynonymous SNV	CLEC18C	chr16	70211370	C	C	C	G_het	C
nonsynonymous SNV	CLEC2D	chr12	9847448	C	T_het	C	C	C
nonsynonymous SNV	CLEC4D	chr12	8670768	C	C	C	C	T_het
nonsynonymous SNV	CLECL1	chr12	9885593	T	C_het	T	T	T
nonsynonymous SNV	CLIP2	chr7	73753227	G	G	A_het	G	G
nonsynonymous SNV	CLIP2	chr7	73803526	G	A_het	G	G	G
nonsynonymous SNV	CLK1	chr2	201726579	G	G	A_het	G	G
nonsynonymous SNV	CLK2P	chr7	23625289	A	A	A	T_het	A
nonsynonymous SNV	CLK3	chr15	74921021	C	C	T_het	C	C
nonsynonymous SNV	CLN6	chr15	68510957	G	G	A_het	G	G
nonsynonymous SNV	CLN8	chr8	1719231	C	C	C	T_het	C
nonsynonymous SNV	CLSTN2	chr3	140275479	G	A_het	G	G	G
nonsynonymous SNV	CLTCL1	chr22	19196615	C	C	C	T_hom	C
nonsynonymous SNV	CLUH	chr17	2595703	G	G	G	A_hom	G
nonsynonymous SNV	CLYBL	chr13	100425097	G	G	T_het	G	G
nonsynonymous SNV	CMA1	chr14	24976674	G	G	G	G	T_het
nonsynonymous SNV	CMSS1	chr3	99885236	A	A	G_het	A	A
nonsynonymous SNV	CMTM5	chr14	23847995	G	G	A_het	G	G
nonsynonymous SNV	CMTM5	chr14	23848563	G	A_het	G	G	G
nonsynonymous SNV	CMYA5	chr5	79027830	C	C	C	C	T_het
nonsynonymous SNV	CMYA5	chr5	79033534	G	G	G	G	T_het
nonsynonymous SNV	CMYA5	chr5	79034662	C	C	C	C	G_hom
nonsynonymous SNV	CNDP2	chr18	72180845	C	C	T_het	C	C
nonsynonymous SNV	CNGB3	chr8	87588042	G	G	G	C_het	G
nonsynonymous SNV	CNGB3	chr8	87588238	T	T	C_het	T	T

nonsynonymous SNV	CNOT1	chr16	58577329	C	A_het	C	C	C
nonsynonymous SNV	CNPY4	chr7	99719929	G	G	G	C_het	G
nonsynonymous SNV	CNR2	chr1	24201164	G	G	A_het	G	G
nonsynonymous SNV	CNTD2	chr19	40729096	C	C	G_het	C	C
nonsynonymous SNV	CNTF	chr11	58391937	A	A	A	A	G_hom
nonsynonymous SNV	CNTN2	chr1	205030522	C	C	T_het	C	C
nonsynonymous SNV	CNTN3	chr3	74347155	C	C	C	C	T_het
nonsynonymous SNV	CNTN4	chr3	3030052	A	A	A	G_hom	A
nonsynonymous SNV	CNTN5	chr11	99690286	T	G_het	G_het	G_het	G_het
stopgain	CNTN5	chr11	99690376	C	C	T_het	T_het	C
nonsynonymous SNV	CNTN5	chr11	99690428	T	G_het	G_het	G_het	T
nonsynonymous SNV	CNTN5	chr11	99690461	A	G_het	A	A	A
nonsynonymous SNV	CNTNAP3B	chr9	43861081	T	G_het	G_het	G_hom	G_hom
nonsynonymous SNV	CNTNAP4	chr16	76482814	G	G	A_het	G	G
stopgain	CNTNAP4	chr16	76532537	C	C	T_het	C	C
nonsynonymous SNV	CNTNAP5	chr2	125660609	C	C	C	T_het	C
nonsynonymous SNV	CNTROB	chr17	7846836	A	A	T_hom	A	A
nonsynonymous SNV	COBL	chr7	51097193	C	T_het	C	C	C
nonsynonymous SNV	COL14A1	chr8	121292257	G	G	G	A_het	G
nonsynonymous SNV	COL18A1	chr21	46875874	G	G	A_het	G	G
nonsynonymous SNV	COL20A1	chr20	61950924	G	G	T_het	G	G
nonsynonymous SNV	COL22A1	chr8	139809074	T	T	T	G_het	T
nonsynonymous SNV	COL22A1	chr8	139838920	T	T	T	C_het	T
nonsynonymous SNV	COL23A1	chr5	177673411	C	C	C	C	T_het
nonsynonymous SNV	COL24A1	chr1	86435932	G	G	G	G	C_het
nonsynonymous SNV	COL24A1	chr1	86488259	C	C	T_het	C	C
nonsynonymous SNV	COL25A1	chr4	109780843	T	T	T	C_hom	T
nonsynonymous SNV	COL26A1	chr7	101091015	C	C	T_het	C	C
nonsynonymous SNV	COL27A1	chr9	117068924	G	G	A_het	G	G
nonsynonymous SNV	COL2A1	chr12	48368604	C	C	T_het	C	C
nonsynonymous SNV	COL2A1	chr12	48380926	G	G	A_het	G	G
nonsynonymous SNV	COL2A1	chr12	48390390	C	C	C	C	T_het
nonsynonymous SNV	COL4A3	chr2	228173636	A	A	G_het	A	A
nonsynonymous SNV	COL4A4	chr2	227895261	G	G	G	C_het	G
nonsynonymous SNV	COL4A4	chr2	228012150	T	T	T	C_het	T
nonsynonymous SNV	COL4A6	chrX	107433669	C	T_het	C	C	C
nonsynonymous SNV	COL5A2	chr2	189932742	G	G	G	A_het	G
nonsynonymous SNV	COL6A2	chr21	47546080	G	G	A_het	G	G
nonsynonymous SNV	COL6A2	chr21	47549152	G	G	A_het	G	G
nonsynonymous SNV	COL6A3	chr2	238275771	G	G	G	A_het	G
nonsynonymous SNV	COL6A3	chr2	238277257	C	C	T_het	C	C
nonsynonymous SNV	COL6A3	chr2	238280781	A	A	A	A	C_het
nonsynonymous SNV	COL6A3	chr2	238287746	C	C	C	T_het	C
nonsynonymous SNV	COL7A1	chr3	48619779	G	G	G	A_hom	G
nonsynonymous SNV	COL7A1	chr3	48623857	C	C	T_hom	C	C

nonsynonymous SNV	COL9A1	chr6	70972993	T	C_het	T	T	T
nonsynonymous SNV	COL9A2	chr1	40781324	C	C	C	C	T_het
nonsynonymous SNV	COPA	chr1	160268730	A	A	A	A	G_het
nonsynonymous SNV	COPS7B	chr2	232660864	C	C	T_het	C	C
nonsynonymous SNV	COPZ2	chr17	46115079	C	C	T_het	C	C
nonsynonymous SNV	COQ2	chr4	84185512	T	T	T	T	C_het
nonsynonymous SNV	COQ2	chr4	84205995	A	A	A	A	C_het
nonsynonymous SNV	CORIN	chr4	47645061	G	A_het	G	G	G
nonsynonymous SNV	CORO7-PAM16	chr16	4410488	C	C	T_het	C	C
nonsynonymous SNV	COX10	chr17	14110294	G	G	G	T_hom	G
nonsynonymous SNV	COX19	chr7	1015109	G	G	G	T_het	G
nonsynonymous SNV	COX19	chr7	1015110	G	G	G	C_het	G
nonsynonymous SNV	CPA1	chr7	130025713	G	G	G	G	A_hom
nonsynonymous SNV	CPD	chr17	28706114	C	C	C	C	T_hom
nonsynonymous SNV	CPD	chr17	28706630	G	G	G	G	T_hom
nonsynonymous SNV	CPD	chr17	28791695	C	C	C	T_hom	C
nonsynonymous SNV	CPNE7	chr16	89649923	C	C	C	C	T_het
nonsynonymous SNV	CPS1	chr2	211456637	A	G_hom	G_het	G_hom	A
nonsynonymous SNV	CPS1	chr2	211465305	C	C	C	T_het	C
nonsynonymous SNV	CPS1	chr2	211540515	G	G	A_het	G	G
nonsynonymous SNV	CPSF1	chr8	145618976	T	T	C_het	T	T
nonsynonymous SNV	CPSF6	chr12	69651879	G	G	T_het	G	G
nonsynonymous SNV	CPXM2	chr10	125651157	C	C	C	G_het	C
nonsynonymous SNV	CPZ	chr4	8616184	T	T	T	T	C_het
nonsynonymous SNV	CRABP1	chr15	78635889	C	C	C	C	T_het
nonsynonymous SNV	CRAMP1L	chr16	1682279	G	G	A_het	G	G
nonsynonymous SNV	CRB1	chr1	197297580	G	G	G	T_hom	G
nonsynonymous SNV	CRB1	chr1	197398616	G	G	G	G	A_het
nonsynonymous SNV	CRB2	chr9	126132727	T	T	T	G_hom	T
nonsynonymous SNV	CREB1	chr2	208420462	A	A	A	G_het	A
nonsynonymous SNV	CREB3L3	chr19	4154930	A	A	T_het	A	A
nonsynonymous SNV	CREBBP	chr16	3781845	G	G	G	T_het	G
nonsynonymous SNV	CRIP1	chr14	105954538	C	C	C	T_het	C
nonsynonymous SNV	CRIPAK	chr4	1388817	C	C	A_hom	C	C
nonsynonymous SNV	CROCC	chr1	17250856	C	C	C	C	T_het
nonsynonymous SNV	CROCC	chr1	17264920	C	T_het	T_het	T_het	T_het
nonsynonymous SNV	CROCC	chr1	17265560	C	T_het	T_het	T_het	T_het
nonsynonymous SNV	CROCC	chr1	17266395	C	C	C	G_het	C
nonsynonymous SNV	CROCC	chr1	17266536	G	C_het	C_het	C_het	C_het
nonsynonymous SNV	CROCC	chr1	17267302	A	G_het	G_het	G_het	A
nonsynonymous SNV	CRTC2	chr1	153927461	G	G	G	A_hom	G
nonsynonymous SNV	CRYBB2P1	chr22	25855425	C	C	C	C	T_hom
nonsynonymous SNV	CRYBB3	chr22	25603018	G	G	G	A_hom	G
nonsynonymous SNV	CRYGC	chr2	208993181	G	G	G	A_het	G
nonsynonymous SNV	CSAG1	chrX	151904488	C	A_het	A_hom	C	A_hom

nonsynonymous SNV	CSF2RB	chr22	37325836	G	G	T_het	G	G
nonsynonymous SNV	CSMD1	chr8	2832137	C	C	T_het	C	C
nonsynonymous SNV	CSMD2	chr1	34035114	T	T	C_het	T	T
nonsynonymous SNV	CSMD2	chr1	34498249	G	G	A_het	G	G
nonsynonymous SNV	CSPG4	chr15	75969317	C	C	T_het	C	C
nonsynonymous SNV	CSPG4	chr15	75981860	C	C	C	T_het	C
nonsynonymous SNV	CSPG4	chr15	75982058	T	T	T	A_het	T
nonsynonymous SNV	CSPG4	chr15	75982271	C	C	C	T_het	C
nonsynonymous SNV	CSPG4	chr15	75982492	G	G	G	A_het	G
nonsynonymous SNV	CSPG4	chr15	75982513	A	A	A	G_het	A
nonsynonymous SNV	CSPG4	chr15	75982540	A	A	G_het	A	A
nonsynonymous SNV	CSPG4	chr15	75982581	G	G	G	C_het	G
nonsynonymous SNV	CSRP2BP	chr20	18125886	G	G	A_het	G	G
nonsynonymous SNV	CTAGE10P	chr13	50466749	C	C	G_het	C	C
nonsynonymous SNV	CTAGE10P	chr13	50466911	G	G	A_het	G	G
nonsynonymous SNV	CTAGE10P	chr13	50466990	T	T	T	T	G_het
nonsynonymous SNV	CTAGE15	chr7	143270172	A	A	G_hom	G_het	A
nonsynonymous SNV	CTAGE6	chr7	143453466	T	T	C_het	T	T
nonsynonymous SNV	CTAGE6	chr7	143453467	G	G	C_het	G	G
nonsynonymous SNV	CTBS	chr1	85040098	T	T	T	T	C_hom
nonsynonymous SNV	CTDP1	chr18	77477863	T	T	T	C_hom	T
nonsynonymous SNV	CTDSPL2	chr15	44788617	C	C	C	T_het	C
nonsynonymous SNV	CTNNA2	chr2	80646623	A	A	A	G_het	A
nonsynonymous SNV	CTNNA3	chr10	68139039	G	A_het	G	G	G
nonsynonymous SNV	CTNND2	chr5	11364898	C	C	T_het	C	C
nonsynonymous SNV	CTRL	chr16	67965099	C	C	C	T_het	C
nonsynonymous SNV	CTSW	chr11	65648889	C	C	T_het	C	C
nonsynonymous SNV	CTTN	chr11	70271499	T	G_het	T	T	T
nonsynonymous SNV	CUBN	chr10	16911740	G	G	A_het	G	G
nonsynonymous SNV	CUBN	chr10	16930419	C	C	C	G_het	C
nonsynonymous SNV	CUBN	chr10	16955889	G	G	G	G	A_het
nonsynonymous SNV	CUL1	chr7	148494914	C	C	T_het	C	C
nonsynonymous SNV	CUL9	chr6	43155718	G	A_het	G	G	G
nonsynonymous SNV	CUX1	chr7	101840472	A	T_het	A	A	A
nonsynonymous SNV	CXCL2	chr4	74964625	T	C_het	T	T	T
nonsynonymous SNV	CXorf23	chrX	19983405	G	G	A_hom	G	G
nonsynonymous SNV	CXorf40B	chrX	149101934	A	A	A	A	C_het
nonsynonymous SNV	CXXC11	chr2	242814705	C	C	C	T_het	C
nonsynonymous SNV	CYFIP2	chr5	156816401	T	T	T	T	A_het
nonsynonymous SNV	CYP11B1	chr8	143957333	G	G	G	C_het	G
nonsynonymous SNV	CYP24A1	chr20	52788189	C	C	C	T_het	C
nonsynonymous SNV	CYP2A7	chr19	41384760	C	C	C	G_het	C
nonsynonymous SNV	CYP2D6	chr22	42523505	C	C	C	C	T_het
nonsynonymous SNV	CYP2D6	chr22	42523528	C	C	T_het	C	C
nonsynonymous SNV	CYP2D6	chr22	42523636	C	C	A_het	C	C

nonsynonymous SNV	CYP2D6	chr22	42526649	C	C	T_het	C	C
nonsynonymous SNV	CYP2D7P1	chr22	42536653	T	T	C_het	T	T
nonsynonymous SNV	CYP2E1	chr10	135340999	C	C	T_het	C	C
nonsynonymous SNV	CYP2U1	chr4	108866639	G	G	G	G	C_het
nonsynonymous SNV	CYP2W1	chr7	1027121	G	G	A_het	G	G
nonsynonymous SNV	CYP2W1	chr7	1028084	G	G	G	A_het	G
nonsynonymous SNV	CYP4A22	chr1	47610574	C	T_hom	C	C	C
nonsynonymous SNV	CYP4F11	chr19	16035680	G	A_het	G	G	G
nonsynonymous SNV	CYP4F12	chr19	15791188	G	G	G	A_het	G
nonsynonymous SNV	CYP4F12	chr19	15794349	G	G	G	T_het	G
nonsynonymous SNV	CYP4F12	chr19	15794352	G	G	G	C_het	G
nonsynonymous SNV	CYP4F12	chr19	15794362	G	G	G	A_het	G
nonsynonymous SNV	CYP4F12	chr19	15794379	C	C	C	T_het	C
nonsynonymous SNV	CYP4F12	chr19	15794415	C	C	C	T_het	C
nonsynonymous SNV	CYP4F2	chr19	15989730	T	T	T	T	C_het
nonsynonymous SNV	CYP4F2	chr19	15996828	G	C_het	G	G	G
nonsynonymous SNV	CYP4F3	chr19	15754810	G	G	A_het	G	G
nonsynonymous SNV	CYP4F8	chr19	15728977	T	T	C_het	T	T
nonsynonymous SNV	CYTH4	chr22	37708215	G	G	A_het	G	G
nonsynonymous SNV	DAAM1	chr14	59834172	G	G	G	G	C_het
nonsynonymous SNV	DAAM2	chr6	39866697	T	T	C_het	T	T
nonsynonymous SNV	DAB1	chr1	57489239	C	C	T_het	C	C
nonsynonymous SNV	DAGLB	chr7	6449512	C	C	C	T_het	C
nonsynonymous SNV	DAGLB	chr7	6472559	G	G	G	A_het	G
nonsynonymous SNV	DAND5	chr19	13080436	C	T_het	C	C	C
nonsynonymous SNV	DAOA	chr13	106119412	A	A	G_het	A	A
nonsynonymous SNV	DBNDD2	chr20	44035150	C	C	C	T_het	C
stopgain	DCAF12L1	chrX	125686516	G	G	A_hom	G	G
nonsynonymous SNV	DCAF15	chr19	14070472	G	G	G	A_het	G
nonsynonymous SNV	DCAF8L1	chrX	27998602	T	T	C_hom	T	T
nonsynonymous SNV	DCDC2B	chr1	32681069	C	C	C	A_hom	C
nonsynonymous SNV	DCLK2	chr4	151153898	T	T	C_het	T	T
nonsynonymous SNV	DCLRE1A	chr10	115612695	T	T	T	G_het	T
nonsynonymous SNV	DCLRE1A	chr10	115612730	C	C	C	T_het	C
nonsynonymous SNV	DCST2	chr1	155001777	A	A	G_het	A	A
nonsynonymous SNV	DCTN1	chr2	74588705	G	G	A_het	G	G
nonsynonymous SNV	DCTN1	chr2	74596438	C	C	T_het	C	C
nonsynonymous SNV	DCTN1	chr2	74596527	C	C	T_het	C	C
nonsynonymous SNV	DCTN4	chr5	150110239	T	C_het	T	T	T
nonsynonymous SNV	DCUN1D2	chr13	114134903	T	T	C_het	T	T
nonsynonymous SNV	DDI1	chr11	103907858	G	G	G	G	C_het
nonsynonymous SNV	DDX1	chr2	15770900	G	T_het	G	G	G
nonsynonymous SNV	DDX11L11	chr12	92119	T	T	C_het	C_het	T
nonsynonymous SNV	DDX11L11	chr12	92915	C	C	G_het	C	C
nonsynonymous SNV	DDX11L11	chr12	92920	C	G_hom	C	G_het	G_het

nonsynonymous SNV	DDX20	chr1	112308782	C	G_het	C	C	C
nonsynonymous SNV	DDX23	chr12	49231419	C	C	T_het	C	C
nonsynonymous SNV	DDX31	chr9	135522272	T	C_hom	T	T	T
nonsynonymous SNV	DDX31	chr9	135538016	C	C	T_het	C	C
nonsynonymous SNV	DDX41	chr5	176939841	C	C	T_het	C	C
nonsynonymous SNV	DDX53	chrX	23018832	A	A	G_hom	A	A
nonsynonymous SNV	DDX54	chr12	113612455	T	T	C_het	T	T
nonsynonymous SNV	DDX54	chr12	113612894	G	G	A_het	G	G
nonsynonymous SNV	DDX58	chr9	32481427	G	G	C_het	G	G
nonsynonymous SNV	DEAF1	chr11	674657	T	T	C_het	T	T
nonsynonymous SNV	DECR2	chr16	461507	G	G	G	G	A_hom
nonsynonymous SNV	DENND1A	chr9	126144262	G	G	G	A_hom	G
nonsynonymous SNV	DENND1A	chr9	126202690	C	C	A_het	C	C
nonsynonymous SNV	DENND2C	chr1	115141972	C	C	T_het	C	C
nonsynonymous SNV	DENND2C	chr1	115142870	G	G	A_het	G	G
nonsynonymous SNV	DENND3	chr8	142161751	G	G	A_het	G	G
nonsynonymous SNV	DENND4B	chr1	153912094	C	C	T_het	C	C
nonsynonymous SNV	DEPDC1	chr1	68942629	G	G	A_het	G	G
nonsynonymous SNV	DEPDC1	chr1	68948107	T	T	C_het	T	T
nonsynonymous SNV	DERL3	chr22	24179166	G	G	G	A_hom	G
nonsynonymous SNV	DES	chr2	220284876	C	T_het	C	C	C
nonsynonymous SNV	DGKA	chr12	56334704	T	T	T	G_het	T
nonsynonymous SNV	DHRS11	chr17	34951458	T	T	A_het	T	T
nonsynonymous SNV	DHRS2	chr14	24114395	G	G	A_het	G	G
nonsynonymous SNV	DHRS4L2	chr14	24439209	C	C	T_het	C	C
nonsynonymous SNV	DHRS4L2	chr14	24439304	G	G	C_het	G	C_het
nonsynonymous SNV	DHRS4L2	chr14	24439455	A	A	A	A	T_hom
nonsynonymous SNV	DHRS4L2	chr14	24459443	G	G	G	C_het	G
nonsynonymous SNV	DHX34	chr19	47870350	G	G	G	G	A_het
nonsynonymous SNV	DHX34	chr19	47883126	C	T_het	C	C	C
nonsynonymous SNV	DHX34	chr19	47884536	G	G	A_het	G	G
nonsynonymous SNV	DHX37	chr12	125434714	C	C	T_het	C	C
nonsynonymous SNV	DHX38	chr16	72130820	A	A	A	A	T_het
nonsynonymous SNV	DIAPH1	chr5	140907186	A	A	A	C_het	A
nonsynonymous SNV	DIAPH1	chr5	140951547	C	C	G_het	C	C
nonsynonymous SNV	DIDO1	chr20	61525238	C	C	T_het	C	C
nonsynonymous SNV	DIEXF	chr1	210010524	G	G	A_het	G	G
nonsynonymous SNV	DIP2A	chr21	47952044	C	C	T_het	C	C
nonsynonymous SNV	DIRC1	chr2	189599497	A	C_het	A	A	A
nonsynonymous SNV	DIS3L2	chr2	232888990	G	G	G	A_het	G
nonsynonymous SNV	DISP2	chr15	40659361	C	T_hom	C	C	C
nonsynonymous SNV	DKFZp434B061	Unknown	65540	T	C_hom	C_hom	C_hom	C_hom
nonsynonymous SNV	DKFZp434J194	chr19	3121926	G	G	G	A_het	G
nonsynonymous SNV	DKFZp434P0216	chr9	133279128	G	G	G	A_hom	G
nonsynonymous SNV	DKFZp686O16217	chr14	106054692	T	T	T	T	C_het

nonsynonymous SNV	DKFZp686O16217	chr14	106054693	C	C	C	C	A_het
nonsynonymous SNV	DKFZp779M0652	chr11	45793188	G	A_het	G	G	G
nonsynonymous SNV	DLEC1	chr3	38153715	A	A	A	G_hom	A
nonsynonymous SNV	DLEC1	chr3	38158036	C	C	T_hom	C	C
nonsynonymous SNV	DLEC1	chr3	38158562	G	G	A_hom	G	G
nonsynonymous SNV	DLG4	chr17	7094050	C	C	T_het	C	C
nonsynonymous SNV	DLGAP2	chr8	1616657	G	G	G	G	A_hom
nonsynonymous SNV	DLGAP3	chr1	35365814	G	G	A_het	G	G
nonsynonymous SNV	DLK2	chr6	43418710	C	C	C	T_hom	C
nonsynonymous SNV	DLL1	chr6	170593000	C	C	T_het	C	C
nonsynonymous SNV	DMBT1	chr10	124329683	C	C	C	A_het	C
nonsynonymous SNV	DMD	chrX	32430305	T	A_het	T	T	T
nonsynonymous SNV	DMGDH	chr5	78338202	T	C_het	T	T	T
nonsynonymous SNV	DMGDH	chr5	78347175	T	C_het	T	T	T
nonsynonymous SNV	DMKN	chr19	35997077	G	C_het	G	G	G
nonsynonymous SNV	DMPK	chr19	46274311	C	C	G_het	C	C
nonsynonymous SNV	DMRT2	chr9	1051713	C	C	C	A_hom	C
nonsynonymous SNV	DMRT2	chr9	1056870	C	C	T_het	C	C
nonsynonymous SNV	DNA2	chr10	70182521	A	A	G_het	A	A
nonsynonymous SNV	DNA2	chr10	70192259	G	G	A_het	G	G
nonsynonymous SNV	DNAAF1	chr16	84203734	G	G	G	G	A_het
nonsynonymous SNV	DNAAF2	chr14	50100463	A	A	T_het	A	A
nonsynonymous SNV	DNAH10	chr12	124413089	C	C	C	A_het	C
nonsynonymous SNV	DNAH11	chr7	21698506	C	C	C	T_het	C
nonsynonymous SNV	DNAH11	chr7	21775406	A	A	A	A	G_hom
nonsynonymous SNV	DNAH11	chr7	21894071	A	A	A	C_het	A
nonsynonymous SNV	DNAH11	chr7	21932167	C	T_het	C	C	C
nonsynonymous SNV	DNAH14	chr1	225152289	C	C	C	T_hom	C
nonsynonymous SNV	DNAH14	chr1	225555519	T	T	T	A_hom	T
nonsynonymous SNV	DNAH17	chr17	76548866	C	T_het	C	C	C
nonsynonymous SNV	DNAH3	chr16	20966168	C	A_het	C	C	C
nonsynonymous SNV	DNAH3	chr16	20974857	T	T	T	C_het	T
nonsynonymous SNV	DNAH3	chr16	21042467	G	G	G	A_het	G
nonsynonymous SNV	DNAH3	chr16	21093012	C	C	C	T_het	C
nonsynonymous SNV	DNAH5	chr5	13721110	T	T	T	T	C_het
nonsynonymous SNV	DNAH5	chr5	13864592	C	C	C	G_het	C
nonsynonymous SNV	DNAH6	chr2	84771536	C	C	C	T_het	C
nonsynonymous SNV	DNAH7	chr2	196799490	C	C	C	A_het	C
nonsynonymous SNV	DNAH7	chr2	196913080	T	T	T	A_het	T
nonsynonymous SNV	DNAH8	chr6	38754694	A	A	A	A	G_het
stopgain	DNAH8	chr6	38998103	C	C	T_het	C	C
nonsynonymous SNV	DNAJC16	chr1	15863072	C	C	T_het	C	C
nonsynonymous SNV	DNAJC6	chr1	65858491	A	A	G_het	A	A
nonsynonymous SNV	DNASE1L2	chr16	2287607	T	T	C_het	T	T
nonsynonymous SNV	DNHD1	chr11	6524126	A	A	A	A	G_het

nonsynonymous SNV	DNM1L	chr12	32886670	G	G	A_het	G	G
nonsynonymous SNV	DNM1P46	chr15	100340342	T	T	C_het	T	T
nonsynonymous SNV	DNM1P46	chr15	100340393	T	T	G_het	T	T
nonsynonymous SNV	DNM1P46	chr15	100340405	C	C	G_het	C	C
nonsynonymous SNV	DNMBP	chr10	101658021	G	G	G	C_het	G
nonsynonymous SNV	DNMBP	chr10	101667814	A	G_het	A	A	A
nonsynonymous SNV	DNMT3A	chr2	25466773	C	C	T_het	C	C
nonsynonymous SNV	DNMT3B	chr20	31368253	C	C	C	T_het	C
nonsynonymous SNV	DNTT	chr10	98078174	C	C	C	T_het	C
nonsynonymous SNV	DOCK1	chr10	128798503	G	G	A_het	G	G
nonsynonymous SNV	DOCK1	chr10	129242460	C	T_het	C	C	C
nonsynonymous SNV	DOCK10	chr2	225639636	C	C	C	C	T_hom
nonsynonymous SNV	DOCK11	chrX	117809966	A	G_het	A	A	A
nonsynonymous SNV	DOCK5	chr8	25149582	T	T	C_het	T	T
nonsynonymous SNV	DOK3	chr5	176931871	C	C	C	T_het	C
nonsynonymous SNV	DOK3	chr5	176936531	A	A	A	C_het	A
nonsynonymous SNV	DOK4	chr16	57509077	C	C	C	C	T_het
nonsynonymous SNV	DONSON	chr21	34960634	G	G	G	C_het	G
nonsynonymous SNV	DPP10	chr2	116572494	T	T	T	G_het	T
nonsynonymous SNV	DPP6	chr7	154561258	G	G	G	T_het	G
nonsynonymous SNV	DPP9	chr19	4685735	C	C	T_het	C	C
nonsynonymous SNV	DPT	chr1	168683529	C	C	C	C	T_het
nonsynonymous SNV	DPY19L2P2	chr7	102920467	C	C	C	G_het	C
nonsynonymous SNV	DPYSL5	chr2	27164857	A	A	G_het	A	A
nonsynonymous SNV	DQ580909	chr8	11872705	G	G	G	A_hom	G
nonsynonymous SNV	DQ583205	chr16	89299807	A	G_hom	G_hom	G_hom	A
nonsynonymous SNV	DQ583205	chr16	89299821	G	G	G	A_het	G
nonsynonymous SNV	DQ583205	chr16	89300052	G	G	G	T_het	G
stopgain	DQ596646	chr10	118590400	A	A	A	T_het	A
nonsynonymous SNV	DQX1	chr2	74750651	C	T_hom	C	C	C
nonsynonymous SNV	DRAXIN	chr1	11775178	A	A	T_het	A	A
nonsynonymous SNV	DRD4	chr11	637335	G	C_het	G	G	G
nonsynonymous SNV	DRD4	chr11	640493	G	G	A_het	G	G
nonsynonymous SNV	DRGX	chr10	50574312	C	C	T_het	C	C
nonsynonymous SNV	DROSHA	chr5	31521275	G	G	T_het	G	G
nonsynonymous SNV	DRP2	chrX	100505962	G	G	T_hom	G	G
nonsynonymous SNV	DSCAM	chr21	41711128	C	C	A_het	C	C
nonsynonymous SNV	DSCAM	chr21	41711192	C	C	T_het	C	C
nonsynonymous SNV	DSCAM	chr21	41741083	G	G	A_het	G	G
nonsynonymous SNV	DSPP	chr4	88537035	A	A	A	A	G_het
nonsynonymous SNV	DSPP	chr4	88537349	A	A	A	A	G_het
nonsynonymous SNV	DST	chr6	56437757	G	T_het	G	G	G
nonsynonymous SNV	DST	chr6	56482923	A	G_het	A	A	A
nonsynonymous SNV	DUOXA2	chr15	45408394	C	C	T_het	C	C
nonsynonymous SNV	DUSP15	chr20	30436211	C	C	C	A_het	C

nonsynonymous SNV	DUSP15	chr20	30436310	C	C	C	T_het	C
nonsynonymous SNV	DUSP16	chr12	12629892	G	G	G	A_het	G
nonsynonymous SNV	DUSP27	chr1	167096056	C	C	T_het	C	C
nonsynonymous SNV	DUSP5	chr10	112257971	A	A	G_het	A	A
nonsynonymous SNV	DUSP8	chr11	1578634	C	C	T_het	C	C
nonsynonymous SNV	DYNAP	chr18	52265110	G	G	G	A_het	G
nonsynonymous SNV	DYNC1H1	chr14	102469286	C	C	T_het	C	C
nonsynonymous SNV	DYRK2	chr12	68051420	C	C	A_het	C	C
nonsynonymous SNV	DYRK4	chr12	4702211	A	A	T_het	A	A
nonsynonymous SNV	DYRK4	chr12	4708874	A	A	A	G_het	A
stopgain	DYSF	chr2	71778214	C	C	C	C	G_het
nonsynonymous SNV	DYTN	chr2	207527815	C	C	C	G_hom	C
nonsynonymous SNV	DZANK1	chr20	18440933	T	T	T	T	C_het
nonsynonymous SNV	E2F1	chr20	32267636	C	C	T_het	C	C
nonsynonymous SNV	EBF4	chr20	2736313	G	G	G	A_het	G
nonsynonymous SNV	EBLN1	chr10	22498039	C	C	C	C	A_het
nonsynonymous SNV	ECD	chr10	74894526	A	A	A	C_het	A
nonsynonymous SNV	ECHS1	chr10	135183495	C	C	C	A_het	C
nonsynonymous SNV	ECI1	chr16	2296927	G	A_het	G	G	G
nonsynonymous SNV	ECM1	chr1	150484272	C	C	T_het	C	C
nonsynonymous SNV	ECT2	chr3	172473297	G	G	G	A_hom	G
nonsynonymous SNV	ECT2L	chr6	139202208	G	G	G	A_hom	G
nonsynonymous SNV	EDAR	chr2	109539845	C	C	A_het	C	C
nonsynonymous SNV	EDIL3	chr5	83362312	C	G_het	C	C	C
nonsynonymous SNV	EDN3	chr20	57896131	C	T_hom	C	C	C
nonsynonymous SNV	EEA1	chr12	93196187	A	A	G_het	A	A
nonsynonymous SNV	EEF2K	chr16	22277748	G	G	G	T_het	G
nonsynonymous SNV	EEF2K	chr16	22278068	G	T_het	G	G	G
nonsynonymous SNV	EEF2K	chr16	22291668	A	A	G_het	A	A
nonsynonymous SNV	EFCAB14	chr1	47149056	T	T	T	C_hom	T
nonsynonymous SNV	EFHC1	chr6	52329845	G	G	G	A_hom	G
nonsynonymous SNV	EFHC2	chrX	44037731	C	C	C	C	T_hom
stopgain	EFR3A	chr8	132999857	T	T	A_het	T	T
nonsynonymous SNV	EFTUD1	chr15	82533742	T	T	T	C_het	T
nonsynonymous SNV	EGFLAM	chr5	38458471	C	C	C	C	T_het
nonsynonymous SNV	EHBP1L1	chr11	65352989	G	G	G	G	T_het
nonsynonymous SNV	EHMT1	chr9	140637830	G	G	T_het	G	G
nonsynonymous SNV	EIF2A	chr3	150290309	C	C	C	G_hom	C
nonsynonymous SNV	EIF2B4	chr2	27592767	C	G_het	C	C	C
nonsynonymous SNV	EIF4E1B	chr5	176072513	G	G	G	G	A_het
nonsynonymous SNV	EIF4G1	chr3	184039373	C	C	T_het	C	C
nonsynonymous SNV	ELANE	chr19	855762	C	T_het	C	C	C
nonsynonymous SNV	ELFN2	chr22	37770961	T	T	T	T	C_hom
nonsynonymous SNV	ELK2AP	chr14	106134635	C	G_het	G_het	C	C
nonsynonymous SNV	ELK2AP	chr14	106134672	G	C_het	C_het	G	G

nonsynonymous SNV	ELK2AP	chr14	106134726	T	C_het	T	T	T
nonsynonymous SNV	ELL2	chr5	95236474	G	G	T_het	G	G
nonsynonymous SNV	ELL3	chr15	44067985	A	A	G_het	A	A
nonsynonymous SNV	ELMO3	chr16	67235504	C	C	C	A_het	C
nonsynonymous SNV	ELMOD1	chr11	107501174	T	T	T	G_hom	T
nonsynonymous SNV	ELP2	chr18	33744465	A	A	A	G_hom	A
nonsynonymous SNV	ELP2	chr18	33750075	A	A	A	G_hom	A
nonsynonymous SNV	EMC7	chr15	34380331	T	T	T	A_het	T
nonsynonymous SNV	EMID1	chr22	29628260	C	C	G_het	C	C
nonsynonymous SNV	EMILIN2	chr18	2890796	G	G	A_het	G	G
nonsynonymous SNV	EML2	chr19	46124782	C	C	C	C	A_het
nonsynonymous SNV	EML5	chr14	89178711	T	T	T	C_het	T
nonsynonymous SNV	EMR1	chr19	6901923	G	G	T_het	G	G
nonsynonymous SNV	EMR2	chr19	14877799	G	C_hom	C_het	C_hom	C_hom
nonsynonymous SNV	EN1	chr2	119604082	G	G	G	G	C_het
nonsynonymous SNV	ENAM	chr4	71497408	A	A	A	A	T_het
nonsynonymous SNV	ENAM	chr4	71500185	A	A	A	A	T_het
nonsynonymous SNV	ENC1	chr5	73931581	T	T	T	T	C_het
nonsynonymous SNV	ENGASE	chr17	77077032	C	C	T_het	C	C
nonsynonymous SNV	ENPP1	chr6	132190576	A	A	A	A	G_het
nonsynonymous SNV	ENTHD1	chr22	40257832	G	G	A_het	G	G
nonsynonymous SNV	ENTPD7	chr10	101458358	C	C	C	T_het	C
nonsynonymous SNV	EP300	chr22	41546041	C	C	C	T_hom	C
nonsynonymous SNV	EP400	chr12	132445421	A	G_het	A	A	A
nonsynonymous SNV	EP400	chr12	132466835	C	C	A_het	C	C
nonsynonymous SNV	EP400	chr12	132466836	C	C	T_het	C	C
nonsynonymous SNV	EP400	chr12	132497581	G	G	A_het	G	G
nonsynonymous SNV	EP400NL	chr12	132589649	C	C	T_het	T_het	C
nonsynonymous SNV	EPB41L4A	chr5	111500730	G	G	A_het	G	G
nonsynonymous SNV	EPHA10	chr1	38227505	C	C	T_het	C	C
nonsynonymous SNV	EPHA8	chr1	22903085	C	C	T_het	C	C
nonsynonymous SNV	EPHB4	chr7	100403136	G	G	A_het	G	G
nonsynonymous SNV	EPHX1	chr1	226019508	G	G	G	A_hom	G
nonsynonymous SNV	EPN1	chr19	56188985	C	C	G_het	C	C
nonsynonymous SNV	EPN1	chr19	56206137	G	G	C_het	G	G
nonsynonymous SNV	EPPIN-WFDC6	chr20	44171347	T	T	T	T	G_hom
nonsynonymous SNV	EPPK1	chr8	144941903	G	G	G	A_het	G
nonsynonymous SNV	EPS15L1	chr19	16503140	G	G	G	G	A_het
nonsynonymous SNV	EPS8L1	chr19	55593237	G	G	A_het	G	G
nonsynonymous SNV	EPS8L1	chr19	55593703	G	G	T_het	G	G
nonsynonymous SNV	EPX	chr17	56274393	C	T_het	C	C	C
nonsynonymous SNV	ERGIC1	chr5	172341897	C	C	C	T_het	C
nonsynonymous SNV	ERI1	chr8	8860644	C	C	C	G_het	C
nonsynonymous SNV	ERI2	chr16	20809183	T	T	T	C_het	T
nonsynonymous SNV	ERO1L	chr14	53124727	C	C	C	C	A_het

nonsynonymous SNV	ERO1LB	chr1	236445020	C	C	C	T_hom	C
nonsynonymous SNV	ERRFI1	chr1	8075375	T	T	T	T	C_het
nonsynonymous SNV	ERV3-1	chr7	64452576	C	C	C	T_het	C
nonsynonymous SNV	ERVFRD-1	chr6	11105413	C	A_het	C	C	C
nonsynonymous SNV	ERVW-1	chr7	92098680	C	C	C	C	T_het
nonsynonymous SNV	ERVW-1	chr7	92098864	G	G	G	A_het	G
nonsynonymous SNV	ERVW-1	chr7	92098917	T	T	T	C_het	T
stopgain	ERVW-1	chr7	92098918	G	G	G	A_het	G
nonsynonymous SNV	ESPN	chr1	6505817	C	C	T_het	C	C
nonsynonymous SNV	ESPNL	chr2	239013338	A	A	C_het	A	A
nonsynonymous SNV	ESPNP	chr1	17017717	C	C	T_het	T_het	T_het
nonsynonymous SNV	ESPNP	chr1	17023143	C	T_het	T_het	C	T_het
nonsynonymous SNV	ESPNP	chr1	17025992	G	A_het	A_het	G	A_het
nonsynonymous SNV	ESPNP	chr1	17026004	C	G_het	G_het	G_het	G_het
nonsynonymous SNV	ESPNP	chr1	17029462	A	G_het	G_het	A	A
nonsynonymous SNV	ESPNP	chr1	17030504	T	T	A_het	A_hom	A_het
nonsynonymous SNV	ESPNP	chr1	17030515	G	G	A_het	A_hom	A_het
nonsynonymous SNV	ESPNP	chr1	17030518	C	C	T_het	T_hom	T_het
nonsynonymous SNV	ESPNP	chr1	17030519	T	T	G_het	G_hom	G_het
nonsynonymous SNV	ESPNP	chr1	17030555	T	T	T	C_het	T
nonsynonymous SNV	ESPNP	chr1	17030590	G	G	A_het	A_hom	A_het
nonsynonymous SNV	ESPNP	chr1	17033805	C	T_het	T_het	C	T_het
nonsynonymous SNV	ESPNP	chr1	17034140	C	C	C	G_het	C
nonsynonymous SNV	ESRRA	chr11	64083221	G	G	C_het	G	G
nonsynonymous SNV	ESRRA	chr11	64083269	G	G	A_het	G	A_het
nonsynonymous SNV	ESRRA	chr11	64083272	C	C	C	C	T_het
nonsynonymous SNV	ESRRA	chr11	64083290	G	G	A_het	G	A_het
nonsynonymous SNV	ESRRA	chr11	64083293	G	G	T_het	G	T_het
nonsynonymous SNV	ESRRA	chr11	64083320	T	T	C_het	T	T
nonsynonymous SNV	ESRRA	chr11	64083328	C	C	T_het	C	C
nonsynonymous SNV	ESRRA	chr11	64083331	C	C	T_het	C	C
nonsynonymous SNV	ETFA	chr15	76523730	T	T	G_het	T	T
nonsynonymous SNV	ETFA	chr15	76603710	G	G	A_het	G	G
nonsynonymous SNV	EVC	chr4	5749975	C	C	C	T_hom	C
nonsynonymous SNV	EVC2	chr4	5586448	C	C	T_het	C	C
nonsynonymous SNV	EVPL	chr17	74003591	C	C	T_het	C	C
nonsynonymous SNV	EVPL	chr17	74004016	C	C	T_het	C	C
nonsynonymous SNV	EVPL	chr17	74017554	G	T_het	G	G	T_het
nonsynonymous SNV	EXD3	chr9	140243678	G	A_hom	G	G	G
nonsynonymous SNV	EXOC3L1	chr16	67218767	G	G	G	A_het	G
nonsynonymous SNV	EXOC6	chr10	94653257	A	A	A	A	G_het
nonsynonymous SNV	EYS	chr6	64430513	A	A	A	A	C_het
nonsynonymous SNV	EYS	chr6	65300250	C	C	G_het	C	C
nonsynonymous SNV	F12	chr5	176829405	G	G	G	A_het	G
nonsynonymous SNV	F12	chr5	176831083	C	C	C	C	G_het

nonsynonymous SNV	F12	chr5	176833015	G	G	A_het	G	G
nonsynonymous SNV	F8	chrX	154124456	G	G	A_hom	G	G
stopgain	FAAH2	chrX	57475022	G	T_het	G	G	G
nonsynonymous SNV	FADS1	chr11	61584217	G	G	T_het	G	G
nonsynonymous SNV	FAM115C	chr7	143417083	T	C_hom	T	T	T
nonsynonymous SNV	FAM117B	chr2	203621999	C	C	T_het	C	C
nonsynonymous SNV	FAM120B	chr6	170627656	C	C	C	C	G_het
nonsynonymous SNV	FAM126A	chr7	22999995	T	T	C_het	T	T
nonsynonymous SNV	FAM129A	chr1	184764331	C	C	C	T_hom	C
nonsynonymous SNV	FAM129A	chr1	184787810	T	T	T	G_hom	T
nonsynonymous SNV	FAM129C	chr19	17650165	G	G	G	A_het	G
nonsynonymous SNV	FAM131C	chr1	16385042	G	G	A_het	G	G
nonsynonymous SNV	FAM135A	chr6	71246100	C	C	C	A_het	C
nonsynonymous SNV	FAM149B1	chr10	74999077	C	C	C	C	A_het
nonsynonymous SNV	FAM153B	chr5	175533585	C	T_het	C	C	T_het
nonsynonymous SNV	FAM160A2	chr11	6238968	C	C	C	A_hom	C
nonsynonymous SNV	FAM160B2	chr8	21956096	G	G	A_het	G	G
nonsynonymous SNV	FAM161B	chr14	74416864	A	A	A	G_het	A
nonsynonymous SNV	FAM166B	chr9	35563351	A	A	G_het	A	A
nonsynonymous SNV	FAM167B	chr1	32713033	G	G	A_het	G	G
nonsynonymous SNV	FAM170B	chr10	50340310	C	C	T_het	C	C
nonsynonymous SNV	FAM171A1	chr10	15256400	C	C	C	C	A_het
nonsynonymous SNV	FAM172A	chr5	93217191	C	C	T_het	C	C
nonsynonymous SNV	FAM178A	chr10	102684413	C	C	C	C	T_het
nonsynonymous SNV	FAM178A	chr10	102684656	A	G_het	A	A	A
stoploss	FAM179B	chr14	45475489	T	T	T	T	G_het
stopgain	FAM183A	chr1	43621956	G	G	A_het	G	G
nonsynonymous SNV	FAM184B	chr4	17635336	G	G	A_hom	G	G
nonsynonymous SNV	FAM185A	chr7	102389833	G	C_hom	C_hom	C_hom	C_hom
nonsynonymous SNV	FAM186A	chr12	50745821	T	T	T	A_het	T
nonsynonymous SNV	FAM186A	chr12	50745822	T	T	T	G_het	T
nonsynonymous SNV	FAM189A2	chr9	72006603	C	C	C	T_hom	C
nonsynonymous SNV	FAM189B	chr1	155217689	C	C	C	C	T_het
nonsynonymous SNV	FAM205A	chr9	34725069	T	T	T	A_het	T
nonsynonymous SNV	FAM205B	chr9	34834494	C	C	T_het	C	C
nonsynonymous SNV	FAM205B	chr9	34835175	G	G	G	G	A_het
nonsynonymous SNV	FAM205B	chr9	34835273	A	A	C_het	C_hom	A
nonsynonymous SNV	FAM207A	chr21	46380023	G	G	A_het	G	G
nonsynonymous SNV	FAM208A	chr3	56716785	C	C	C	C	T_het
nonsynonymous SNV	FAM20A	chr17	66533866	G	A_het	G	G	G
nonsynonymous SNV	FAM213A	chr10	82187221	G	G	T_het	G	G
nonsynonymous SNV	FAM214A	chr15	52901882	C	C	C	C	T_het
stopgain	FAM21A	chr10	51827929	G	A_het	G	G	G
nonsynonymous SNV	FAM21B	chr10	47915891	C	A_het	C	C	C
nonsynonymous SNV	FAM35A	chr10	88911587	G	G	G	G	A_het

nonsynonymous SNV	FAM35DP	chr10	47416908	A	A	G_het	G_het	G_het
nonsynonymous SNV	FAM35DP	chr10	47420307	T	T	T	C_het	T
nonsynonymous SNV	FAM45B	chr10	120863708	C	C	C	C	G_het
nonsynonymous SNV	FAM50B	chr6	3850338	G	G	G	G	A_het
nonsynonymous SNV	FAM53C	chr5	137681105	G	G	G	T_het	G
nonsynonymous SNV	FAM63B	chr15	59064420	C	C	T_het	C	C
nonsynonymous SNV	FAM69B	chr9	139617604	G	A_hom	G	G	G
nonsynonymous SNV	FAM71B	chr5	156589481	C	C	C	C	T_het
nonsynonymous SNV	FAM71E2	chr19	55869601	T	T	T	C_het	T
nonsynonymous SNV	FAM83D	chr20	37555060	G	G	G	G	C_hom
nonsynonymous SNV	FAM83G	chr17	18881325	C	C	T_het	C	C
nonsynonymous SNV	FAM86A	chr16	5140539	C	T_hom	C	C	C
nonsynonymous SNV	FAM86B1	chr8	12042821	T	C_het	T	T	T
nonsynonymous SNV	FAM86B1	chr8	12042822	C	T_het	C	C	C
nonsynonymous SNV	FAM86C1	chr11	71507186	T	T	T	C_het	T
nonsynonymous SNV	FAN1	chr15	31197423	C	C	C	C	A_het
nonsynonymous SNV	FANCC	chr9	97912307	T	T	A_het	T	T
nonsynonymous SNV	FANCD2	chr3	10088343	A	G_het	G_het	G_het	A
nonsynonymous SNV	FANCF	chr11	22646398	G	G	A_het	G	G
nonsynonymous SNV	FANCI	chr15	89835982	C	C	C	A_het	C
nonsynonymous SNV	FANCL	chr2	58459232	G	G	G	A_het	G
nonsynonymous SNV	FANCM	chr14	45633577	C	T_het	C	C	C
nonsynonymous SNV	FANCM	chr14	45645820	A	A	A	G_het	A
nonsynonymous SNV	FANCM	chr14	45658363	C	C	A_het	C	C
nonsynonymous SNV	FANCM	chr14	45665690	C	C	C	T_het	C
nonsynonymous SNV	FARP1	chr13	99064222	A	G_hom	A	A	A
nonsynonymous SNV	FAT1	chr4	187542704	G	G	G	G	A_het
nonsynonymous SNV	FAT2	chr5	150911379	T	G_het	T	T	T
nonsynonymous SNV	FAT2	chr5	150925848	G	G	G	C_het	G
nonsynonymous SNV	FAT2	chr5	150931117	C	T_het	C	C	C
nonsynonymous SNV	FAT3	chr11	92086345	G	G	G	G	A_het
nonsynonymous SNV	FAT3	chr11	92088327	C	C	T_het	C	C
nonsynonymous SNV	FAT4	chr4	126370909	C	C	T_het	C	C
nonsynonymous SNV	FAT4	chr4	126411388	G	G	G	A_hom	G
nonsynonymous SNV	FBLN2	chr3	13659636	C	C	T_hom	C	C
nonsynonymous SNV	FBN2	chr5	127624905	T	T	T	A_het	T
nonsynonymous SNV	FBN3	chr19	8151095	G	G	G	G	A_het
nonsynonymous SNV	FBN3	chr19	8175953	C	C	T_het	C	C
nonsynonymous SNV	FBN3	chr19	8186255	C	A_het	C	C	C
nonsynonymous SNV	FBN3	chr19	8188682	C	C	T_het	C	C
nonsynonymous SNV	FBN3	chr19	8212232	C	C	C	T_het	C
nonsynonymous SNV	FBP2	chr9	97333824	C	T_hom	C	C	C
nonsynonymous SNV	FBP2	chr9	97333839	G	G	A_het	G	G
nonsynonymous SNV	FBXL6	chr8	145580759	C	C	A_het	C	C
nonsynonymous SNV	FBXO24	chr7	100187349	T	T	A_het	T	T

nonsynonymous SNV	FBXO30	chr6	146125793	A	A	A	T_hom	A
nonsynonymous SNV	FBXO40	chr3	121341019	A	T_hom	A	A	A
nonsynonymous SNV	FBXO46	chr19	46216030	G	G	G	C_het	G
nonsynonymous SNV	FBXO6	chr1	11733864	G	A_hom	G	G	G
nonsynonymous SNV	FBXW7	chr4	153332559	T	T	T	C_hom	T
stopgain	FBXW9	chr19	12800981	C	T_het	C	C	C
nonsynonymous SNV	FCAMR	chr1	207134104	G	G	T_het	G	G
nonsynonymous SNV	FCGBP	chr19	40368330	C	T_het	T_het	C	C
nonsynonymous SNV	FCGBP	chr19	40376675	G	A_het	G	G	G
nonsynonymous SNV	FCGBP	chr19	40382310	G	G	G	C_het	G
nonsynonymous SNV	FCGBP	chr19	40392588	C	C	C	C	T_het
nonsynonymous SNV	FCGBP	chr19	40406035	T	T	T	C_het	T
nonsynonymous SNV	FCGBP	chr19	40408268	G	T_het	T_het	G	G
nonsynonymous SNV	FCGBP	chr19	40408811	G	G	G	A_het	G
nonsynonymous SNV	FCGR2B	chr1	161643269	C	C	T_het	C	C
nonsynonymous SNV	FCGR3A	chr1	161514542	A	C_het	C_het	A	C_het
nonsynonymous SNV	FCRL1	chr1	157789820	G	A_het	A_het	G	G
nonsynonymous SNV	FDXACB1	chr11	111746169	C	C	C	T_hom	C
nonsynonymous SNV	FDXR	chr17	72860442	G	G	A_het	G	G
nonsynonymous SNV	FER1L4	chr20	34170035	C	G_hom	G_hom	G_hom	G_hom
nonsynonymous SNV	FER1L6	chr8	124988167	T	T	T	C_het	T
nonsynonymous SNV	FER1L6	chr8	125052176	G	G	A_het	G	G
nonsynonymous SNV	FER1L6	chr8	125072446	T	T	C_het	T	T
nonsynonymous SNV	FFAR3	chr19	35849926	G	A_het	A_hom	A_het	A_het
nonsynonymous SNV	FGB	chr4	155488907	C	C	G_het	C	C
nonsynonymous SNV	FGD2	chr6	36976842	G	G	G	G	A_het
nonsynonymous SNV	FGF2	chr4	123797480	G	G	G	T_hom	G
nonsynonymous SNV	FGFBP3	chr10	93668219	C	C	C	C	A_het
nonsynonymous SNV	FGFR1	chr8	38279260	C	C	C	T_het	C
nonsynonymous SNV	FGFR1OP2	chr12	27109525	G	G	A_het	G	G
nonsynonymous SNV	FGR	chr1	27942079	G	G	A_het	G	G
nonsynonymous SNV	FIGN	chr2	164467428	G	G	A_het	G	G
nonsynonymous SNV	FIZ1	chr19	56109194	G	G	A_het	G	G
nonsynonymous SNV	FKBP5	chr6	35543617	C	C	C	C	T_het
nonsynonymous SNV	FKBP5	chr6	35565101	C	C	T_het	C	C
nonsynonymous SNV	FLCN	chr17	17129413	C	C	T_het	C	C
stopgain	FLG	chr1	152275298	T	T	T	T	A_hom
nonsynonymous SNV	FLG	chr1	152276699	A	A	G_het	A	A
nonsynonymous SNV	FLG	chr1	152278555	T	T	C_het	T	T
nonsynonymous SNV	FLG	chr1	152278856	T	T	G_het	T	T
stopgain	FLG	chr1	152280023	G	G	A_het	G	G
nonsynonymous SNV	FLG	chr1	152280471	C	C	G_het	C	C
nonsynonymous SNV	FLG	chr1	152280736	T	T	C_het	T	T
nonsynonymous SNV	FLG	chr1	152280788	T	T	G_het	T	T
nonsynonymous SNV	FLG	chr1	152281039	G	G	A_het	G	G

nonsynonymous SNV	FLG	chr1	152281228	C	C	G_het	C	C
nonsynonymous SNV	FLG	chr1	152281304	A	A	C_het	A	A
nonsynonymous SNV	FLG	chr1	152281523	A	A	C_het	A	A
nonsynonymous SNV	FLG	chr1	152285911	C	C	T_het	C	C
nonsynonymous SNV	FLG2	chr1	152324348	A	T_het	A	A	A
nonsynonymous SNV	FLG2	chr1	152324371	G	T_het	G	G	G
nonsynonymous SNV	FLG2	chr1	152324374	C	A_het	C	C	C
nonsynonymous SNV	FLG2	chr1	152324389	T	G_het	T	T	T
nonsynonymous SNV	FLG2	chr1	152324402	C	T_het	C	C	C
nonsynonymous SNV	FLG2	chr1	152327140	T	T	T	T	C_hom
nonsynonymous SNV	FLII	chr17	18148534	C	C	C	T_hom	C
nonsynonymous SNV	FLJ10038	chr15	50646598	G	G	G	A_het	G
nonsynonymous SNV	FLJ22184	chr19	7934076	G	G	G	G	A_het
nonsynonymous SNV	FLJ22184	chr19	7938149	G	G	G	T_het	G
nonsynonymous SNV	FLJ44635	chrX	71379777	G	G	G	A_hom	G
nonsynonymous SNV	FLJ45340	chr7	128291823	G	G	G	T_het	G
nonsynonymous SNV	FLJ45340	chr7	128291825	C	C	C	T_het	C
nonsynonymous SNV	FLNC	chr7	128488745	G	G	A_het	G	G
nonsynonymous SNV	FLOT2	chr17	27208326	C	C	C	T_hom	C
nonsynonymous SNV	FLRT1	chr11	63884146	A	G_het	A	A	A
nonsynonymous SNV	FLRT3	chr20	14306224	G	G	G	G	C_het
nonsynonymous SNV	FLT3	chr13	28623587	C	C	T_het	C	C
nonsynonymous SNV	FLT4	chr5	180030322	C	C	T_het	C	C
nonsynonymous SNV	FLYWCH1	chr16	2980828	G	G	G	A_het	G
nonsynonymous SNV	FLYWCH1	chr16	2983223	G	A_het	G	G	G
nonsynonymous SNV	FMN1	chr15	33446302	T	T	C_het	T	T
nonsynonymous SNV	FMN1	chr15	33446416	C	C	C	C	T_het
nonsynonymous SNV	FN1	chr2	216264021	T	T	T	T	G_het
nonsynonymous SNV	FN3KRP	chr17	80684784	G	G	G	A_hom	G
nonsynonymous SNV	FNDC3B	chr3	172046841	G	T_het	G	G	G
nonsynonymous SNV	FNDC7	chr1	109260498	C	C	T_het	C	C
nonsynonymous SNV	FNIP2	chr4	159753107	A	G_het	A	A	A
nonsynonymous SNV	FOLH1	chr11	49208267	G	A_het	G	G	G
nonsynonymous SNV	FOXD4	chr9	117428	G	G	C_het	G	G
nonsynonymous SNV	FOXD4	chr9	117506	G	G	A_het	A_het	A_het
nonsynonymous SNV	FOXD4	chr9	117696	G	G	A_het	G	A_het
nonsynonymous SNV	FOXD4	chr9	117713	T	T	C_het	T	T
nonsynonymous SNV	FOXD4L1	chr2	114257296	A	A	A	G_het	A
nonsynonymous SNV	FOXI3	chr2	88751648	C	C	T_het	C	C
nonsynonymous SNV	FOXJ1	chr17	74136022	G	G	A_het	G	G
nonsynonymous SNV	FOXN3	chr14	89629057	C	C	C	C	T_het
nonsynonymous SNV	FOXP4	chr6	41552560	G	G	A_het	G	G
nonsynonymous SNV	FPR2	chr19	52272131	T	C_het	T	T	T
nonsynonymous SNV	FPR2	chr19	52272707	G	G	A_het	G	G
nonsynonymous SNV	FRAS1	chr4	79458283	A	A	A	G_hom	A

nonsynonymous SNV	FREM1	chr9	14857744	A	A	A	C_hom	A
nonsynonymous SNV	FREM2	chr13	39262669	G	G	G	C_het	G
nonsynonymous SNV	FREM3	chr4	144498768	T	T	T	C_hom	T
nonsynonymous SNV	FRG1	chr4	190874234	C	T_het	C	C	C
nonsynonymous SNV	FRG1	chr4	190874256	C	T_het	T_het	T_het	T_het
nonsynonymous SNV	FRG1	chr4	190876242	G	A_het	A_het	A_het	A_het
nonsynonymous SNV	FRG1	chr4	190876272	G	G	A_het	G	G
stopgain	FRG1	chr4	190876287	G	G	A_het	G	G
nonsynonymous SNV	FRG1	chr4	190878563	C	A_het	C	C	C
nonsynonymous SNV	FRG1	chr4	190878654	C	G_het	G_het	G_het	G_het
nonsynonymous SNV	FRG1B	chr20	29625895	G	A_het	A_het	G	A_het
nonsynonymous SNV	FRG1B	chr20	29625928	G	A_het	A_het	G	A_het
nonsynonymous SNV	FRG1B	chr20	29625935	A	G_het	G_het	G_het	G_het
nonsynonymous SNV	FRG1B	chr20	29625946	A	G_het	G_het	G_het	G_het
nonsynonymous SNV	FRG1B	chr20	29625950	G	A_het	A_het	A_het	A_het
stopgain	FRG1B	chr20	29625965	G	A_het	A_het	A_het	A_het
nonsynonymous SNV	FRG1B	chr20	29632638	C	A_het	A_het	A_het	A_het
nonsynonymous SNV	FRG1B	chr20	29632643	T	T	C_het	T	C_het
nonsynonymous SNV	FRG1B	chr20	29632662	G	C_het	T_het	G	T_het
nonsynonymous SNV	FRG1B	chr20	29632709	C	T_het	T_het	T_het	C
nonsynonymous SNV	FRG1B	chr20	29632714	C	A_het	A_het	A_het	C
nonsynonymous SNV	FRG1B	chr20	29632715	T	G_het	G_het	G_het	T
nonsynonymous SNV	FRG1B	chr20	29652157	G	G	A_het	A_het	G
nonsynonymous SNV	FRG1B	chr20	29652164	G	G	A_het	A_het	G
nonsynonymous SNV	FRG1B	chr20	29652172	T	C_het	C_het	C_het	C_het
stopgain	FRG1B	chr20	29652173	G	A_het	A_het	A_het	A_het
nonsynonymous SNV	FRG1B	chr20	29652182	C	C	C	C	T_het
nonsynonymous SNV	FRG1B	chr20	29652190	T	T	T	T	G_het
nonsynonymous SNV	FRG1B	chr20	29652191	C	T_het	T_het	T_het	T_het
nonsynonymous SNV	FRG1B	chr20	29652236	G	C_het	C_het	C_het	C_het
nonsynonymous SNV	FRG1B	chr20	29652254	G	A_het	A_het	A_het	A_het
nonsynonymous SNV	FRG2B	chr10	135438929	T	G_het	G_het	G_het	T
nonsynonymous SNV	FRG2B	chr10	135438955	C	T_het	T_het	T_het	C
nonsynonymous SNV	FRG2B	chr10	135438967	C	T_het	T_het	T_het	C
nonsynonymous SNV	FRG2B	chr10	135438977	G	A_het	A_het	G	G
nonsynonymous SNV	FRK	chr6	116289765	C	C	C	C	G_het
nonsynonymous SNV	FRMD5	chr15	44166619	G	G	A_het	G	G
nonsynonymous SNV	FRMPD1	chr9	37735615	A	A	G_het	A	A
nonsynonymous SNV	FSCB	chr14	44974189	C	C	C	A_het	C
nonsynonymous SNV	FSD1	chr19	4323089	G	G	T_het	G	G
nonsynonymous SNV	FSIP2	chr2	186673830	G	C_het	G	G	G
nonsynonymous SNV	FTCD	chr21	47557213	G	G	A_het	G	G
stopgain	FTO	chr16	53859806	C	C	T_het	C	C
nonsynonymous SNV	FUBP1	chr1	78429839	T	T	C_het	T	T
nonsynonymous SNV	FUBP3	chr9	133511400	G	G	G	A_hom	G

nonsynonymous SNV	FUT3	chr19	5844132	C	C	C	C	T_het
nonsynonymous SNV	FUT4	chr11	94277711	G	G	A_het	G	G
nonsynonymous SNV	FUT5	chr19	5866736	T	C_hom	C_het	T	T
nonsynonymous SNV	FUT6	chr19	5832028	T	T	T	C_het	T
nonsynonymous SNV	FZD9	chr7	72848876	C	C	A_het	C	C
nonsynonymous SNV	GAB2	chr11	77930455	T	C_het	T	T	T
nonsynonymous SNV	GABRB3	chr15	26866498	G	G	A_het	G	G
nonsynonymous SNV	GABRG1	chr4	46043111	C	C	C	C	T_het
nonsynonymous SNV	GAD2	chr10	26513551	G	A_het	G	G	G
nonsynonymous SNV	GAK	chr4	862363	C	C	A_het	C	C
nonsynonymous SNV	GAL	chr11	68458396	C	C	C	C	T_het
nonsynonymous SNV	GALNT3	chr2	166606326	C	T_het	C	C	C
nonsynonymous SNV	GALNT5	chr2	158165232	C	C	T_het	C	C
nonsynonymous SNV	GAMT	chr19	1398782	C	C	C	T_het	C
nonsynonymous SNV	GANC	chr15	42630673	A	A	G_het	A	A
nonsynonymous SNV	GAPT	chr5	57790635	A	G_het	A	A	A
nonsynonymous SNV	GAR1	chr4	110737457	G	G	G	C_hom	G
nonsynonymous SNV	GART	chr21	34892844	T	T	T	C_het	T
nonsynonymous SNV	GAS2L2	chr17	34074182	C	C	T_het	C	C
nonsynonymous SNV	GAS2L3	chr12	101018321	G	G	G	A_het	G
nonsynonymous SNV	GAST	chr17	39871804	A	A	A	A	G_hom
nonsynonymous SNV	GATA5	chr20	61041534	G	G	A_het	G	G
stoploss	GATA6	chr18	19780784	T	T	C_het	T	T
nonsynonymous SNV	GATSL3	chr22	30683077	A	A	A	G_hom	A
stopgain	GBA2	chr9	35741755	G	G	A_het	G	G
nonsynonymous SNV	GBA2	chr9	35748376	C	C	C	T_hom	C
nonsynonymous SNV	GBP5	chr1	89729574	C	C	C	C	T_het
nonsynonymous SNV	GC	chr4	72622439	C	C	A_het	C	C
nonsynonymous SNV	GC	chr4	72631215	T	T	C_het	T	T
nonsynonymous SNV	GCDH	chr19	13007764	C	C	T_het	C	C
nonsynonymous SNV	GCFC2	chr2	75899101	T	T	T	T	C_het
nonsynonymous SNV	GCNT2	chr6	10529626	A	A	G_het	A	A
nonsynonymous SNV	GCNT2	chr6	10556664	T	T	T	C_hom	T
nonsynonymous SNV	GDF7	chr2	20871094	G	G	A_het	G	G
nonsynonymous SNV	GDPD4	chr11	76940264	T	T	T	C_hom	T
nonsynonymous SNV	GEMIN4	chr17	648893	G	G	A_het	G	G
nonsynonymous SNV	GEMIN5	chr5	154271297	C	C	C	T_het	C
nonsynonymous SNV	GFM1	chr3	158364658	A	A	G_het	A	A
nonsynonymous SNV	GFM2	chr5	74060888	G	A_het	G	G	G
nonsynonymous SNV	GGA2	chr16	23492002	C	C	C	C	T_hom
stopgain	GGN	chr19	38875072	G	G	A_het	C_het	G
nonsynonymous SNV	GGT5	chr22	24615951	G	G	A_het	G	G
nonsynonymous SNV	GGT6	chr17	4461489	G	G	G	A_hom	G
nonsynonymous SNV	GGT6	chr17	4461715	G	G	G	T_hom	G
nonsynonymous SNV	GGT6	chr17	4461777	G	G	G	A_hom	G

nonsynonymous SNV	GH2	chr17	61957744	G	G	A_het	G	G
nonsynonymous SNV	GH2	chr17	61958750	T	T	C_het	T	T
nonsynonymous SNV	GIGYF2	chr2	233612414	G	G	A_het	G	G
nonsynonymous SNV	GIMAP2	chr7	150390255	T	T	T	C_het	T
nonsynonymous SNV	GIMD1	chr4	107279576	T	T	G_het	T	T
nonsynonymous SNV	GINS3	chr16	58437168	G	G	A_het	G	G
nonsynonymous SNV	GIT2	chr12	110429443	T	T	C_het	T	T
stopgain	GJA10	chr6	90605664	C	C	T_het	C	C
nonsynonymous SNV	GJC3	chr7	99521208	G	G	A_het	G	G
nonsynonymous SNV	GJD4	chr10	35897205	G	G	G	G	A_het
nonsynonymous SNV	GLB1	chr3	33110436	G	G	T_hom	G	G
nonsynonymous SNV	GLB1L2	chr11	134226244	G	A_het	G	G	G
nonsynonymous SNV	GLI3	chr7	42188012	C	C	T_het	C	C
nonsynonymous SNV	GLOD4	chr17	663472	C	A_het	C	C	C
nonsynonymous SNV	GLRA1	chr5	151202476	C	C	T_het	C	C
nonsynonymous SNV	GLS	chr2	191797569	G	G	G	A_het	G
nonsynonymous SNV	GLT8D2	chr12	104408832	T	T	C_het	T	T
nonsynonymous SNV	GLTPD1	chr1	1262886	G	G	A_het	G	G
nonsynonymous SNV	GLYAT	chr11	58478159	C	C	C	C	T_hom
nonsynonymous SNV	GLYR1	chr16	4861709	G	C_hom	C_hom	C_hom	C_hom
stopgain	GMIP	chr19	19745615	G	G	A_het	G	G
nonsynonymous SNV	GNA12	chr7	2853995	C	C	T_het	C	C
nonsynonymous SNV	GNB4	chr3	179131231	G	G	G	A_hom	G
nonsynonymous SNV	GNL2	chr1	38032482	G	G	G	G	A_het
nonsynonymous SNV	GNLY	chr2	85924689	G	G	A_het	G	G
nonsynonymous SNV	GNPDA2	chr4	44724157	C	C	T_het	C	C
nonsynonymous SNV	GNPTAB	chr12	102158576	C	C	T_het	C	C
nonsynonymous SNV	GOLGA1	chr9	127652727	C	C	G_het	C	C
nonsynonymous SNV	GOLGA4	chr3	37368756	G	G	C_hom	G	G
nonsynonymous SNV	GOLGA4	chr3	37396678	G	G	A_hom	G	G
nonsynonymous SNV	GOLGA6L4	chr15	85787910	G	G	A_het	G	A_het
nonsynonymous SNV	GOLGA6L6	chr15	20739697	G	G	G	A_het	G
nonsynonymous SNV	GOLGA6L6	chr15	20739739	A	A	A	A	G_het
stopgain	GOLGA6L6	chr15	20740252	C	C	A_hom	A_het	C
nonsynonymous SNV	GOLGA8A	chr15	34673722	C	C	C	T_het	T_het
nonsynonymous SNV	GON4L	chr1	155720514	C	C	T_het	C	C
nonsynonymous SNV	GORAB	chr1	170508611	A	A	A	A	G_het
nonsynonymous SNV	GPATCH3	chr1	27224123	G	G	A_het	G	G
nonsynonymous SNV	GPC5	chr13	92101033	G	G	G	T_het	G
nonsynonymous SNV	GPC5	chr13	92345672	G	G	G	T_het	G
nonsynonymous SNV	GPD1L	chr3	32200408	G	G	G	A_hom	G
nonsynonymous SNV	GPHB5	chr14	63784378	G	A_het	G	G	G
nonsynonymous SNV	GPI	chr19	34868692	G	G	A_het	G	G
nonsynonymous SNV	GPLD1	chr6	24447168	C	C	G_het	C	C
nonsynonymous SNV	GPLD1	chr6	24454383	C	C	T_het	C	C

nonsynonymous SNV	GPM6A	chr4	176594911	C	C	T_het	C	C
nonsynonymous SNV	GPNMB	chr7	23299648	C	G_het	C	C	C
nonsynonymous SNV	GPNMB	chr7	23307558	C	C	T_het	C	C
stopgain	GPR1	chr2	207041527	G	G	G	G	A_het
nonsynonymous SNV	GPR1	chr2	207041779	A	A	A	A	G_het
nonsynonymous SNV	GPR113	chr2	26533808	C	C	C	C	T_het
nonsynonymous SNV	GPR113	chr2	26534302	G	G	G	A_het	G
nonsynonymous SNV	GPR116	chr6	46846043	T	A_het	T	T	T
nonsynonymous SNV	GPR123	chr10	134886550	C	C	C	T_het	C
nonsynonymous SNV	GPR124	chr8	37695366	G	G	A_het	G	G
nonsynonymous SNV	GPR125	chr4	22517301	G	G	C_het	G	G
nonsynonymous SNV	GPR126	chr6	142726951	A	A	G_het	A	A
nonsynonymous SNV	GPR132	chr14	105517471	C	C	T_het	C	C
nonsynonymous SNV	GPR133	chr12	131475622	T	T	T	C_het	T
nonsynonymous SNV	GPR133	chr12	131488800	A	A	A	C_het	A
nonsynonymous SNV	GPR137	chr11	64056742	C	C	T_het	C	C
nonsynonymous SNV	GPR137B	chr1	236368510	G	G	G	A_hom	G
nonsynonymous SNV	GPR149	chr3	154146714	C	C	C	C	T_het
nonsynonymous SNV	GPR150	chr5	94956923	G	G	A_het	G	G
nonsynonymous SNV	GPR152	chr11	67219865	A	A	G_het	A	A
nonsynonymous SNV	GPR153	chr1	6310562	C	C	G_het	C	C
nonsynonymous SNV	GPR17	chr2	128408968	G	G	G	A_het	G
nonsynonymous SNV	GPR17	chr2	128409186	G	G	A_het	G	G
nonsynonymous SNV	GPR174	chrX	78427047	G	T_het	G	G	G
nonsynonymous SNV	GPR179	chr17	36493586	A	A	T_het	A	A
nonsynonymous SNV	GPR26	chr10	125426215	G	G	A_het	G	G
nonsynonymous SNV	GPR35	chr2	241569454	G	A_het	G	G	G
nonsynonymous SNV	GPR39	chr2	133402914	A	A	C_het	A	A
nonsynonymous SNV	GPR4	chr19	46094208	C	C	C	C	T_het
nonsynonymous SNV	GPR52	chr1	174417319	C	C	T_het	C	C
nonsynonymous SNV	GPR6	chr6	110300868	G	G	A_het	G	G
nonsynonymous SNV	GPR63	chr6	97247030	T	T	T	T	A_het
nonsynonymous SNV	GPR68	chr14	91700787	G	G	A_het	G	G
nonsynonymous SNV	GPR82	chrX	41586919	T	T	C_hom	T	T
nonsynonymous SNV	GPR84	chr12	54757526	C	C	C	C	T_het
nonsynonymous SNV	GPR89A	chr1	145818789	C	C	T_het	C	C
nonsynonymous SNV	GPR98	chr5	89949347	G	G	G	A_het	G
nonsynonymous SNV	GPR98	chr5	90085621	A	A	A	A	G_het
nonsynonymous SNV	GPRC5B	chr16	19883813	C	C	C	T_het	C
nonsynonymous SNV	GPRIN2	chr10	46998894	G	A_het	A_het	A_het	A_het
nonsynonymous SNV	GPRIN2	chr10	46998995	C	G_het	G_het	G_het	G_het
nonsynonymous SNV	GPRIN2	chr10	46998999	G	A_het	A_het	A_het	A_het
nonsynonymous SNV	GPRIN2	chr10	46999019	G	A_het	A_het	A_het	A_het
nonsynonymous SNV	GPRIN2	chr10	46999151	T	C_het	C_het	C_het	C_het
nonsynonymous SNV	GPRIN2	chr10	46999178	A	C_het	C_het	C_het	C_het

nonsynonymous SNV	GPRIN2	chr10	46999484	G	T_het	T_het	T_het	T_het
nonsynonymous SNV	GPRIN2	chr10	46999577	G	T_het	T_het	T_het	T_het
nonsynonymous SNV	GPRIN2	chr10	46999922	G	T_het	T_het	T_het	T_het
nonsynonymous SNV	GPRIN2	chr10	47000079	T	C_het	C_het	C_het	C_het
nonsynonymous SNV	GPSM1	chr9	139222174	T	T	C_hom	C_hom	C_hom
nonsynonymous SNV	GRAMD1B	chr11	123479267	C	C	T_het	C	C
nonsynonymous SNV	GREB1	chr2	11780420	C	C	T_het	C	C
nonsynonymous SNV	GRHL3	chr1	24669457	C	C	C	T_hom	C
nonsynonymous SNV	GRHPR	chr9	37436754	C	C	C	T_hom	C
nonsynonymous SNV	GRID2IP	chr7	6554047	T	T	T	T	C_hom
nonsynonymous SNV	GRIK3	chr1	37270560	T	T	C_het	T	T
nonsynonymous SNV	GRIN3A	chr9	104433048	T	C_hom	T	T	T
nonsynonymous SNV	GRM1	chr6	146755132	G	G	A_het	G	G
nonsynonymous SNV	GRWD1	chr19	48950075	A	A	A	G_het	A
nonsynonymous SNV	GSDMA	chr17	38122680	G	T_hom	T_het	T_hom	G
nonsynonymous SNV	GSDMA	chr17	38126874	C	C	C	C	T_het
nonsynonymous SNV	GSE1	chr16	85697056	C	C	T_het	C	C
nonsynonymous SNV	GSG2	chr17	3628292	C	C	C	T_hom	C
nonsynonymous SNV	GSN	chr9	124073040	C	C	T_het	C	C
nonsynonymous SNV	GSN	chr9	124080722	A	A	G_het	A	A
nonsynonymous SNV	GSTM3	chr1	110282511	A	A	A	G_hom	A
nonsynonymous SNV	GSTM3	chr1	110282891	T	T	T	G_hom	T
nonsynonymous SNV	GTF2E1	chr3	120495353	C	C	T_het	C	C
nonsynonymous SNV	GTF2E1	chr3	120500103	C	C	C	T_hom	C
nonsynonymous SNV	GTF2F1	chr19	6381603	C	C	A_het	C	C
nonsynonymous SNV	GTF2IRD2B	chr7	74564033	G	G	A_het	G	G
nonsynonymous SNV	GTPBP4	chr10	1054946	A	A	A	G_het	A
nonsynonymous SNV	GTPBP8	chr3	112710155	C	C	C	C	G_het
nonsynonymous SNV	GTSE1	chr22	46722518	G	G	A_het	G	G
nonsynonymous SNV	GUCY2EP	chr11	76391277	C	C	C	T_hom	C
nonsynonymous SNV	GUCY2F	chrX	108638614	C	T_het	C	C	C
nonsynonymous SNV	GUSBP1	chr5	21491446	G	G	T_het	G	G
nonsynonymous SNV	GUSBP1	chr5	21497187	C	C	T_het	C	C
nonsynonymous SNV	GVINP1	chr11	6736620	C	C	T_het	C	C
nonsynonymous SNV	GYPA	chr4	144918755	C	C	C	C	A_het
nonsynonymous SNV	GYPB	chr4	144922436	T	T	T	T	G_het
nonsynonymous SNV	GZMH	chr14	25076877	G	G	C_het	G	G
nonsynonymous SNV	H6PD	chr1	9324002	A	A	A	G_hom	A
nonsynonymous SNV	H6PD	chr1	9324735	G	G	A_het	G	G
nonsynonymous SNV	HABP2	chr10	115335685	G	G	A_het	G	G
nonsynonymous SNV	hADV38S2	chr14	22749604	G	A_het	G	G	G
nonsynonymous SNV	HAGHL	chr16	778464	G	G	G	A_het	G
nonsynonymous SNV	HAMP	chr19	35775693	C	T_het	C	C	C
nonsynonymous SNV	HARBI1	chr11	46637628	C	C	C	C	T_het
nonsynonymous SNV	HARS	chr5	140070850	G	G	G	G	T_het

nonsynonymous SNV	HAS1	chr19	52216988	A	A	A	A	G_het
nonsynonymous SNV	HAS1	chr19	52222746	C	C	T_het	C	C
stopgain	HAS3	chr16	69147395	C	C	T_het	C	C
nonsynonymous SNV	HAUS2	chr15	42851600	T	T	T	C_het	T
nonsynonymous SNV	HAUS5	chr19	36106279	A	A	G_het	A	A
nonsynonymous SNV	HAUS7	chrX	152735964	C	C	C	T_hom	C
nonsynonymous SNV	HAVCR1	chr5	156479492	A	A	G_het	A	A
nonsynonymous SNV	HBG1	chr11	5270686	G	G	A_hom	G	G
nonsynonymous SNV	HCG11	chr6	26522435	C	C	G_het	C	C
nonsynonymous SNV	HCK	chr20	30681814	G	G	G	A_het	G
nonsynonymous SNV	HCLS1	chr3	121353085	G	G	T_het	G	G
nonsynonymous SNV	HCN2	chr19	603944	C	C	T_het	C	C
nonsynonymous SNV	HCN3	chr1	155255711	G	G	A_het	G	G
nonsynonymous SNV	HCN3	chr1	155258120	C	C	T_het	C	C
nonsynonymous SNV	HCN4	chr15	73660028	G	G	G	G	A_het
nonsynonymous SNV	HDAC4	chr2	240011662	G	G	G	A_het	G
nonsynonymous SNV	HDAC7	chr12	48189743	C	A_het	C	C	C
nonsynonymous SNV	HDAC7	chr12	48190806	G	G	G	G	A_het
nonsynonymous SNV	HDAC7	chr12	48191247	C	C	C	T_het	C
nonsynonymous SNV	HDGFRP2	chr19	4488757	G	G	G	A_het	G
nonsynonymous SNV	HEATR2	chr7	780999	C	C	A_het	C	C
nonsynonymous SNV	HEATR2	chr7	813773	C	C	T_het	C	C
nonsynonymous SNV	HEATR6	chr17	58153580	C	A_het	C	C	C
nonsynonymous SNV	HEBP1	chr12	13142236	C	C	C	C	G_het
nonsynonymous SNV	HECTD1	chr14	31609180	C	C	C	T_het	C
nonsynonymous SNV	HECTD3	chr1	45469396	C	C	C	C	T_het
nonsynonymous SNV	HECTD4	chr12	112666455	G	G	G	A_het	G
nonsynonymous SNV	HECTD4	chr12	112673475	C	C	T_het	C	C
nonsynonymous SNV	HECW1	chr7	43546798	C	C	C	T_het	C
nonsynonymous SNV	HELZ2	chr20	62193053	C	C	T_het	C	C
nonsynonymous SNV	HELZ2	chr20	62195227	A	A	A	G_het	A
nonsynonymous SNV	HELZ2	chr20	62203663	C	C	C	T_het	C
nonsynonymous SNV	HENMT1	chr1	109198302	C	C	C	T_hom	C
nonsynonymous SNV	HEPHL1	chr11	93808463	C	T_het	C	C	C
nonsynonymous SNV	HERC2	chr15	28501300	C	C	C	C	A_het
nonsynonymous SNV	HERC2	chr15	28518046	G	G	A_het	G	G
nonsynonymous SNV	HERC2	chr15	28518112	C	A_het	A_het	A_het	A_het
nonsynonymous SNV	HERC2	chr15	28518130	G	C_het	C_het	C_het	C_het
nonsynonymous SNV	HERC2	chr15	28518136	G	G	A_het	A_het	A_het
nonsynonymous SNV	HERC2P3	chr15	20588693	C	T_hom	C	C	C
nonsynonymous SNV	HERC2P3	chr15	20588705	C	C	A_het	C	A_het
nonsynonymous SNV	HEY2	chr6	126080823	G	G	A_het	G	G
nonsynonymous SNV	HIPK2	chr7	139259925	C	C	A_het	C	C
nonsynonymous SNV	HIRIP3	chr16	30005460	C	C	C	T_het	C
nonsynonymous SNV	HIST1H2BA	chr6	25727158	G	G	A_het	G	G

nonsynonymous SNV	HIST1H4F	chr6	26240711	C	C	T_het	C	C
nonsynonymous SNV	HIVEP2	chr6	143092118	G	G	G	A_hom	G
nonsynonymous SNV	HIVEP3	chr1	41978566	T	G_hom	G_hom	G_hom	G_hom
nonsynonymous SNV	HK3	chr5	176308984	G	G	G	C_het	G
nonsynonymous SNV	HLA-DQB1	chr6	32632637	T	G_hom	T	G_hom	T
stopgain	HLA-DQB1	chr6	32632638	C	A_hom	C	A_hom	C
nonsynonymous SNV	HLA-DQB1	chr6	32632688	T	A_hom	T	A_hom	T
nonsynonymous SNV	HLA-DQB1	chr6	32632749	A	A	A	A	C_hom
nonsynonymous SNV	HLA-DQB1	chr6	32632770	A	G_hom	A	G_hom	A
nonsynonymous SNV	HLA-DQB1	chr6	32632781	A	C_hom	A	C_hom	T_hom
nonsynonymous SNV	HLA-DQB1	chr6	32632782	G	C_hom	G	C_hom	A_hom
nonsynonymous SNV	HLA-DQB1	chr6	32634302	A	G_hom	A	G_hom	A
nonsynonymous SNV	HLA-DRB1	chr6	32546838	A	G_hom	A	A	A
nonsynonymous SNV	HLA-DRB1	chr6	32551958	C	C	T_het	C	C
nonsynonymous SNV	HLA-DRB1	chr6	32552078	A	T_hom	A	A	A
nonsynonymous SNV	HLA-DRB1	chr6	32552085	G	T_hom	G	G	G
nonsynonymous SNV	HLA-DRB1	chr6	32552091	G	C_hom	G	G	G
nonsynonymous SNV	HLA-DRB1	chr6	32552130	C	A_hom	C	C	C
nonsynonymous SNV	HLA-DRB1	chr6	32552131	C	A_hom	C	C	C
nonsynonymous SNV	HLA-DRB1	chr6	32552132	T	A_hom	T	T	T
nonsynonymous SNV	HLA-DRB1	chr6	32552137	G	A_hom	G	G	G
nonsynonymous SNV	HLA-DRB5	chr6	32489853	A	A	A	A	C_hom
nonsynonymous SNV	HLA-DRB5	chr6	32489856	C	C	C	C	T_hom
nonsynonymous SNV	HLCS	chr21	38137465	C	C	C	T_het	C
nonsynonymous SNV	HLCS	chr21	38308774	C	C	T_het	C	C
nonsynonymous SNV	HMCN1	chr1	185958749	G	G	G	A_hom	G
nonsynonymous SNV	HMCN1	chr1	186038870	C	C	C	C	T_het
nonsynonymous SNV	HMCN1	chr1	186088371	C	A_het	C	C	C
nonsynonymous SNV	HMCN1	chr1	186114604	G	G	G	A_hom	G
nonsynonymous SNV	HNF1A	chr12	121416795	A	A	A	G_het	A
nonsynonymous SNV	HNF1A	chr12	121432068	G	G	A_het	G	G
nonsynonymous SNV	HNRNPC	chr14	21679599	T	T	C_het	T	T
nonsynonymous SNV	HNRNPC	chr14	21679600	C	C	A_het	C	C
nonsynonymous SNV	HNRNPC	chr14	21679620	G	G	A_het	G	G
nonsynonymous SNV	HNRNPC	chr14	21679660	T	T	G_het	T	G_het
nonsynonymous SNV	HNRNPC	chr14	21679666	C	C	T_het	C	T_het
nonsynonymous SNV	HNRNPC	chr14	21679677	A	A	G_het	A	G_het
nonsynonymous SNV	HNRNPC	chr14	21679678	C	C	A_het	C	A_het
nonsynonymous SNV	HNRNPC	chr14	21679689	C	C	G_het	C	C
nonsynonymous SNV	HNRNPC	chr14	21679980	C	T_het	C	C	C
nonsynonymous SNV	HNRNPCL1	chr1	12907284	T	C_het	C_het	C_het	C_het
nonsynonymous SNV	HNRNPCL1	chr1	12907285	G	C_het	C_het	C_het	C_het
nonsynonymous SNV	HNRNPCL1	chr1	12907316	T	C_het	C_het	C_het	C_het
nonsynonymous SNV	HNRNPCL1	chr1	12907350	C	T_het	T_het	T_het	T_het
nonsynonymous SNV	HNRNPCL1	chr1	12907370	A	T_het	T_het	T_het	T_het

nonsynonymous SNV	HNRNPCL1	chr1	12907380	C	A_het	A_het	A_het	A_het
nonsynonymous SNV	HNRNPCL1	chr1	12907385	G	A_het	A_het	A_het	A_het
nonsynonymous SNV	HNRNPCL1	chr1	12907400	G	A_het	A_het	A_het	G
nonsynonymous SNV	HNRNPCL1	chr1	12907408	T	A_het	T	T	T
nonsynonymous SNV	HNRNPCL1	chr1	12907446	C	T_het	T_het	C	T_het
nonsynonymous SNV	HNRNPCL1	chr1	12907457	A	G_het	G_het	A	A
nonsynonymous SNV	HNRNPCL1	chr1	12907458	T	A_het	A_het	T	T
nonsynonymous SNV	HNRNPCL1	chr1	12907469	C	G_het	G_het	G_het	C
nonsynonymous SNV	HNRNPCL1	chr1	12907518	T	A_het	A_het	A_het	A_het
nonsynonymous SNV	HNRNPCL1	chr1	12907519	C	A_het	A_het	A_het	A_het
nonsynonymous SNV	HNRNPCL1	chr1	12907781	T	C_het	C_het	T	C_het
nonsynonymous SNV	HNRNPCL1	chr1	12907798	A	C_het	A	A	C_het
nonsynonymous SNV	HNRNPCL1	chr1	12907802	C	T_het	C	C	T_het
nonsynonymous SNV	HNRNPCL1	chr1	12907803	C	T_het	C	C	T_het
nonsynonymous SNV	HNRNPM	chr19	8550639	C	C	T_het	C	C
nonsynonymous SNV	HOMER2	chr15	83523508	C	C	C	T_het	C
nonsynonymous SNV	HOMEZ	chr14	23745533	C	T_het	T_hom	C	T_het
nonsynonymous SNV	HOXA2	chr7	27140664	C	C	C	T_het	C
nonsynonymous SNV	HOXA9	chr7	27204773	C	C	G_het	C	C
nonsynonymous SNV	HOXB-AS3	chr17	46673441	G	G	A_het	G	G
nonsynonymous SNV	HPR	chr16	72110948	C	G_het	G_het	G_het	G_het
nonsynonymous SNV	HPS5	chr11	18318354	C	T_het	C	C	C
nonsynonymous SNV	HRC	chr19	49655273	C	C	C	T_het	C
nonsynonymous SNV	HRC	chr19	49657018	C	C	C	T_het	C
nonsynonymous SNV	HRNR	chr1	152188413	C	C	T_het	C	C
nonsynonymous SNV	HRNR	chr1	152191469	C	T_het	C	C	C
stopgain	HSD17B14	chr19	49335965	G	G	G	A_het	G
nonsynonymous SNV	HSP90AB4P	chr15	58984657	G	G	G	A_het	G
nonsynonymous SNV	HSPA4L	chr4	128717040	C	C	T_het	C	C
nonsynonymous SNV	HSPG2	chr1	22165539	A	A	A	A	C_het
nonsynonymous SNV	HSPG2	chr1	22165963	T	C_hom	T	T	T
nonsynonymous SNV	HSPG2	chr1	22178047	G	G	G	A_hom	G
nonsynonymous SNV	HSPG2	chr1	22181360	G	C_hom	G	G	G
nonsynonymous SNV	HSPG2	chr1	22186384	T	T	T	C_hom	T
nonsynonymous SNV	HTR1A	chr5	63257500	G	G	G	G	A_het
nonsynonymous SNV	HTR1E	chr6	87725588	A	A	G_het	A	A
nonsynonymous SNV	HTR3C	chr3	183774749	C	C	C	A_hom	C
nonsynonymous SNV	HTR3E	chr3	183824060	C	C	T_het	C	C
nonsynonymous SNV	HTR6	chr1	20005599	G	G	A_het	G	G
nonsynonymous SNV	HTT	chr4	3076656	A	A	A	C_hom	A
nonsynonymous SNV	HVCN1	chr12	111099002	C	C	T_het	C	C
nonsynonymous SNV	HVCN1	chr12	111099003	A	A	G_het	A	A
nonsynonymous SNV	HYAL3	chr3	50332229	G	A_hom	G	G	G
nonsynonymous SNV	HYAL4	chr7	123508623	C	C	C	C	T_hom
nonsynonymous SNV	HYDIN	chr16	70841703	A	A	A	G_het	A

nonsynonymous SNV	HYDIN	chr16	70873994	A	G_het	A	A	A
nonsynonymous SNV	HYDIN	chr16	70926299	C	C	C	T_het	C
nonsynonymous SNV	HYDIN	chr16	70954682	G	G	G	A_het	G
nonsynonymous SNV	IBTK	chr6	82950189	C	C	C	T_hom	C
nonsynonymous SNV	ICOSLG	chr21	45649499	G	G	A_het	G	G
nonsynonymous SNV	ICOSLG	chr21	45649543	G	C_het	G	C_het	G
nonsynonymous SNV	IDH3B	chr20	2640690	C	C	T_hom	C	C
nonsynonymous SNV	IER2	chr19	13264571	G	G	G	A_het	G
nonsynonymous SNV	IFI44	chr1	79125148	G	G	G	A_hom	G
nonsynonymous SNV	IFI44L	chr1	79102844	T	T	T	A_hom	T
stopgain	IFIH1	chr2	163134090	C	C	A_het	C	C
nonsynonymous SNV	IFNA10	chr9	21207000	C	C	C	C	G_hom
nonsynonymous SNV	IFNA4	chr9	21187434	C	C	C	C	G_het
nonsynonymous SNV	IFNA4	chr9	21187439	C	C	C	C	G_het
stopgain	IFNA4	chr9	21187471	A	A	A	A	T_het
nonsynonymous SNV	IFRD2	chr3	50325648	G	G	A_hom	G	G
nonsynonymous SNV	IFT122	chr3	129214302	G	G	A_het	G	G
nonsynonymous SNV	IFT172	chr2	27683894	C	C	T_het	C	C
nonsynonymous SNV	IGF2BP2	chr3	185542726	C	C	G_het	C	C
nonsynonymous SNV	IGFBP6	chr12	53491584	G	G	G	G	A_het
nonsynonymous SNV	IGFN1	chr1	201178965	A	A	A	G_het	A
nonsynonymous SNV	IGFN1	chr1	201179050	A	G_het	G_het	G_het	G_het
nonsynonymous SNV	IGFN1	chr1	201179068	G	A_het	G	A_het	A_het
nonsynonymous SNV	IGFN1	chr1	201180140	C	T_het	C	C	T_het
nonsynonymous SNV	IGFN1	chr1	201180153	A	A	A	A	G_het
nonsynonymous SNV	IGFN1	chr1	201180172	G	A_het	G	G	A_het
nonsynonymous SNV	IGHE	chr14	106329435	T	G_hom	G_hom	G_het	G_hom
nonsynonymous SNV	IGHG1	chr14	106208326	G	G	G	G	C_het
nonsynonymous SNV	IGHMBP2	chr11	68700822	G	G	A_het	G	G
nonsynonymous SNV	IGSF1	chrX	130409069	C	C	C	T_hom	C
nonsynonymous SNV	IGSF9	chr1	159901284	G	G	A_het	G	G
nonsynonymous SNV	IGSF9	chr1	159906300	T	T	C_het	T	T
nonsynonymous SNV	IKZF2	chr2	213886748	C	C	C	T_het	C
nonsynonymous SNV	IL17RA	chr22	17590498	A	A	A	A	G_hom
nonsynonymous SNV	IL17RB	chr3	53892830	T	C_het	T	T	T
nonsynonymous SNV	IL17RC	chr3	9970081	G	G	A_hom	G	G
nonsynonymous SNV	IL22RA1	chr1	24447657	T	T	T	T	C_het
nonsynonymous SNV	IL23R	chr1	67666524	C	C	T_het	C	C
nonsynonymous SNV	IL23R	chr1	67685413	G	G	A_het	G	G
nonsynonymous SNV	IL23R	chr1	67724312	C	C	T_het	C	C
nonsynonymous SNV	IL3	chr5	131396404	G	A_hom	G	G	G
nonsynonymous SNV	IL32	chr16	3119304	A	G_het	G_het	A	G_het
stopgain	IL4R	chr16	27374468	G	G	T_het	G	G
nonsynonymous SNV	IL4R	chr16	27374957	C	C	T_het	C	C
nonsynonymous SNV	IL5RA	chr3	3139702	T	T	T	T	A_hom

nonsynonymous SNV	IL6ST	chr5	55272085	G	C_het	G	G	G
nonsynonymous SNV	ILKAP	chr2	239082232	C	C	T_het	C	C
nonsynonymous SNV	ILVBL	chr19	15226175	T	T	C_het	T	T
nonsynonymous SNV	igheavychain	chr14	107034707	G	T_het	T_het	G	G
nonsynonymous SNV	igheavychain	chr14	107034822	C	G_het	G_het	C	C
nonsynonymous SNV	igheavychain	chr14	107034846	T	G_het	G_het	T	G_het
nonsynonymous SNV	igheavychain	chr14	107034847	C	T_het	T_het	C	T_het
nonsynonymous SNV	igheavychain	chr14	107034854	C	A_het	A_het	C	A_het
nonsynonymous SNV	igheavychain	chr14	107034863	C	T_het	T_het	C	T_het
nonsynonymous SNV	igheavychain	chr14	107034869	A	C_het	C_het	A	C_het
nonsynonymous SNV	igheavychain	chr14	107034873	G	C_het	C_het	G	C_het
nonsynonymous SNV	igheavychain	chr14	107034874	A	C_het	C_het	A	C_het
nonsynonymous SNV	igheavychain	chr14	107034920	C	T_het	T_het	C	T_het
nonsynonymous SNV	igheavychain	chr14	107034967	T	C_het	C_het	T	C_het
nonsynonymous SNV	IMPA2	chr18	12028075	G	G	G	G	A_het
nonsynonymous SNV	INCENP	chr11	61897986	A	A	T_het	A	A
nonsynonymous SNV	INMT	chr7	30795207	G	G	A_het	G	G
nonsynonymous SNV	INO80	chr15	41348869	C	C	T_het	C	C
nonsynonymous SNV	INPP4A	chr2	99170902	G	G	G	G	A_het
nonsynonymous SNV	INPP5A	chr10	134563108	G	G	G	A_het	G
nonsynonymous SNV	INPP5D	chr2	234072413	C	C	T_het	C	C
stopgain	INPP5F	chr10	121586786	C	C	C	C	T_het
nonsynonymous SNV	INPPL1	chr11	71940249	G	G	G	G	A_het
nonsynonymous SNV	INPPL1	chr11	71942647	G	G	A_het	G	G
nonsynonymous SNV	INSM2	chr14	36004219	G	G	G	A_het	G
nonsynonymous SNV	INTS1	chr7	1523240	G	G	G	C_het	G
nonsynonymous SNV	INTS1	chr7	1523679	C	C	T_het	C	C
nonsynonymous SNV	INTS1	chr7	1538413	C	C	T_het	C	C
nonsynonymous SNV	INTS1	chr7	1539139	C	C	C	T_het	C
nonsynonymous SNV	INTS9	chr8	28627498	G	G	A_het	G	G
nonsynonymous SNV	INVS	chr9	103060280	C	C	T_het	C	C
nonsynonymous SNV	IP6K3	chr6	33695951	G	G	A_het	G	G
nonsynonymous SNV	IPO9	chr1	201839744	G	G	C_het	G	G
nonsynonymous SNV	IQCE	chr7	2649704	T	G_het	G_het	G_het	G_hom
nonsynonymous SNV	IQCE	chr7	2649728	G	G	A_het	G	G
nonsynonymous SNV	IQCF3	chr3	51864588	G	G	G	A_hom	G
nonsynonymous SNV	IQCJ-SCHIP1	chr3	159482799	A	A	G_het	A	A
nonsynonymous SNV	IQGAP1	chr15	90931584	C	C	T_het	C	C
stopgain	IRAK2	chr3	10219689	C	C	C	C	T_het
nonsynonymous SNV	IREB2	chr15	78782973	A	A	A	T_het	A
nonsynonymous SNV	IRF3	chr19	50162980	G	G	G	A_het	G
nonsynonymous SNV	ITGA1	chr5	52084209	C	G_het	C	C	C
nonsynonymous SNV	ITGA10	chr1	145533451	G	G	G	G	A_het
nonsynonymous SNV	ITGA2B	chr17	42453084	C	T_het	C	C	C
nonsynonymous SNV	ITGA3	chr17	48166525	G	G	A_het	G	G

nonsynonymous SNV	ITGA6	chr2	173330284	C	C	C	C	T_het
nonsynonymous SNV	ITGAD	chr16	31418868	G	G	A_het	G	G
nonsynonymous SNV	ITGAV	chr2	187455132	C	C	C	C	T_het
nonsynonymous SNV	ITGB3	chr17	45368350	C	C	T_het	C	C
nonsynonymous SNV	ITGB3	chr17	45368505	A	T_het	A	A	A
nonsynonymous SNV	ITGB7	chr12	53585746	A	A	G_het	A	A
nonsynonymous SNV	ITGB7	chr12	53586255	G	G	G	A_het	G
nonsynonymous SNV	ITGB8	chr7	20441504	G	G	G	G	A_hom
nonsynonymous SNV	ITIH2	chr10	7788585	A	A	A	G_het	A
nonsynonymous SNV	ITIH4	chr3	52860869	G	G	A_hom	G	G
nonsynonymous SNV	ITM2A	chrX	78618948	G	G	A_hom	G	G
nonsynonymous SNV	ITPR1	chr3	4752021	G	G	A_hom	G	G
nonsynonymous SNV	ITPR2	chr12	26647162	G	G	A_het	G	G
nonsynonymous SNV	ITPRIP	chr10	106075130	C	C	C	A_het	C
nonsynonymous SNV	ITSN1	chr21	35260555	C	C	T_het	C	C
nonsynonymous SNV	JAG1	chr20	10628626	G	G	A_het	G	G
nonsynonymous SNV	JAG2	chr14	105618349	G	A_het	G	G	G
nonsynonymous SNV	JAK2	chr9	5126443	T	T	T	A_hom	T
nonsynonymous SNV	JARID2	chr6	15513159	G	G	A_het	G	G
nonsynonymous SNV	JAZF1-AS1	chr7	28280324	C	C	T_het	C	C
nonsynonymous SNV	JRK	chr8	143745968	C	T_het	C	C	C
nonsynonymous SNV	JUP	chr17	39912040	C	C	T_het	C	C
nonsynonymous SNV	KANK1	chr9	712757	C	C	C	C	T_hom
nonsynonymous SNV	KANK2	chr19	11289063	G	G	A_het	G	G
nonsynonymous SNV	KANK2	chr19	11303467	C	C	C	C	G_het
nonsynonymous SNV	KANK2	chr19	11303468	C	C	C	G_het	C
nonsynonymous SNV	KANSL1L	chr2	210905194	T	T	C_het	T	T
nonsynonymous SNV	KANSL2	chr12	49047912	C	C	T_het	C	C
nonsynonymous SNV	KANSL2	chr12	49075201	C	C	T_het	C	C
nonsynonymous SNV	KAT6A	chr8	41791414	C	T_het	C	C	C
nonsynonymous SNV	KAT8	chr16	31129108	C	G_het	C	C	C
nonsynonymous SNV	KATNAL2	chr18	44526837	T	C_het	T	T	T
nonsynonymous SNV	KBTBD11	chr8	1951019	G	G	A_het	G	G
nonsynonymous SNV	KBTBD3	chr11	105925018	T	T	C_het	T	T
nonsynonymous SNV	KCNA10	chr1	111061270	G	G	G	G	A_het
nonsynonymous SNV	KCNC3	chr19	50832152	T	T	T	C_hom	T
nonsynonymous SNV	KCNE1	chr21	35821680	C	T_het	C	C	C
nonsynonymous SNV	KCNE1	chr21	35821910	G	G	G	A_het	G
nonsynonymous SNV	KCNG1	chr20	49626203	G	G	A_het	G	G
nonsynonymous SNV	KCNH4	chr17	40315625	G	G	A_het	G	G
nonsynonymous SNV	KCNH5	chr14	63416844	T	T	T	C_het	T
nonsynonymous SNV	KCNH7	chr2	163230011	T	C_het	T	T	T
nonsynonymous SNV	KCNH7	chr2	163374588	C	C	T_het	C	C
nonsynonymous SNV	KCNJ10	chr1	160012270	C	C	T_het	C	C
nonsynonymous SNV	KCNJ12	chr17	21318698	C	T_het	T_het	T_het	T_het

nonsynonymous SNV	KCNJ12	chr17	21318782	G	A_het	A_het	A_het	A_het
nonsynonymous SNV	KCNJ12	chr17	21318867	G	A_het	A_het	A_het	G
nonsynonymous SNV	KCNJ12	chr17	21319069	G	A_het	A_het	A_het	A_het
nonsynonymous SNV	KCNJ12	chr17	21319079	C	A_het	A_het	A_het	A_het
nonsynonymous SNV	KCNJ12	chr17	21319087	G	A_het	A_het	A_het	A_het
nonsynonymous SNV	KCNJ12	chr17	21319121	C	T_het	T_het	T_het	T_het
nonsynonymous SNV	KCNJ12	chr17	21319171	G	A_het	A_het	A_het	A_het
nonsynonymous SNV	KCNJ12	chr17	21319208	C	T_het	T_het	T_het	T_het
nonsynonymous SNV	KCNJ12	chr17	21319230	G	C_het	C_het	C_het	C_het
nonsynonymous SNV	KCNJ12	chr17	21319285	C	T_het	T_het	T_het	T_het
nonsynonymous SNV	KCNJ12	chr17	21319369	G	A_het	G	A_het	A_het
nonsynonymous SNV	KCNJ12	chr17	21319399	A	A	G_het	G_het	G_het
nonsynonymous SNV	KCNJ12	chr17	21319436	G	A_het	A_het	A_het	A_het
nonsynonymous SNV	KCNJ12	chr17	21319439	T	G_het	G_het	G_het	G_het
nonsynonymous SNV	KCNJ12	chr17	21319519	G	C_het	C_het	C_het	G
nonsynonymous SNV	KCNJ12	chr17	21319523	C	T_het	T_het	T_het	C
nonsynonymous SNV	KCNJ12	chr17	21319543	G	A_het	A_het	A_het	A_het
nonsynonymous SNV	KCNJ12	chr17	21319560	G	T_het	T_het	T_het	T_het
nonsynonymous SNV	KCNJ12	chr17	21319682	C	C	T_het	T_het	T_het
nonsynonymous SNV	KCNJ12	chr17	21319786	G	A_het	A_het	A_het	A_het
nonsynonymous SNV	KCNJ12	chr17	21319792	G	G	A_het	G	G
nonsynonymous SNV	KCNJ12	chr17	21319860	C	T_het	T_het	T_het	A_het
nonsynonymous SNV	KCNJ12	chr17	21319868	G	T_het	T_het	T_het	T_het
nonsynonymous SNV	KCNJ12	chr17	21319943	A	G_het	G_het	G_het	G_het
nonsynonymous SNV	KCNQ4	chr1	41303409	C	C	C	G_hom	C
nonsynonymous SNV	KCNT2	chr1	196227299	A	A	A	G_hom	A
nonsynonymous SNV	KCNV2	chr9	2718415	G	G	G	A_hom	G
nonsynonymous SNV	KCTD21	chr11	77885246	G	G	A_het	G	G
nonsynonymous SNV	KCTD3	chr1	215752457	C	C	T_het	C	C
nonsynonymous SNV	KDM1A	chr1	23346231	G	G	G	G	T_het
nonsynonymous SNV	KDM1B	chr6	18222452	C	C	C	T_hom	C
nonsynonymous SNV	KDM4C	chr9	6981115	G	G	A_het	G	G
nonsynonymous SNV	KDM5A	chr12	431586	T	A_het	T	T	T
nonsynonymous SNV	KDM5B	chr1	202715283	T	T	T	T	C_het
nonsynonymous SNV	KDR	chr4	55976709	C	C	C	G_hom	C
nonsynonymous SNV	KEL	chr7	142655047	C	C	T_het	C	C
nonsynonymous SNV	KIAA0146	chr8	48625353	G	G	G	G	A_hom
nonsynonymous SNV	KIAA0226	chr3	197409341	G	G	A_het	G	G
nonsynonymous SNV	KIAA0247	chr14	70171407	G	G	A_het	G	G
nonsynonymous SNV	KIAA0391	chr14	35592774	T	T	T	C_het	T
nonsynonymous SNV	KIAA0556	chr16	27784497	G	G	A_het	G	G
nonsynonymous SNV	KIAA1045	chr9	34971557	G	G	A_het	G	G
nonsynonymous SNV	KIAA1210	chrX	118221283	G	G	G	G	A_hom
nonsynonymous SNV	KIAA1217	chr10	24762382	A	A	G_het	A	A
nonsynonymous SNV	KIAA1217	chr10	24762452	G	G	A_het	G	G

nonsynonymous SNV	KIAA1217	chr10	24790335	T	T	T	T	C_het
nonsynonymous SNV	KIAA1239	chr4	37447421	C	C	C	C	A_het
nonsynonymous SNV	KIAA1407	chr3	113697171	A	A	A	A	G_het
nonsynonymous SNV	KIAA1407	chr3	113765459	G	G	T_het	G	G
nonsynonymous SNV	KIAA1429	chr8	95523925	C	T_het	C	C	C
nonsynonymous SNV	KIAA1429	chr8	95531310	G	G	A_het	G	G
nonsynonymous SNV	KIAA1429	chr8	95541459	A	C_hom	A	A	A
nonsynonymous SNV	KIAA1430	chr4	186096969	C	C	C	C	G_het
nonsynonymous SNV	KIAA1462	chr10	30315181	G	G	G	A_het	G
nonsynonymous SNV	KIAA1462	chr10	30336672	C	C	T_het	C	C
nonsynonymous SNV	KIAA1549	chr7	138603282	T	T	C_hom	T	T
nonsynonymous SNV	KIAA1683	chr19	18367995	T	T	A_het	T	T
stopgain	KIAA1683	chr19	18376448	C	C	C	T_het	C
nonsynonymous SNV	KIAA1715	chr2	176857105	T	T	A_het	T	T
nonsynonymous SNV	KIAA1751	chr1	1886890	A	A	A	A	G_het
nonsynonymous SNV	KIAA1755	chr20	36855584	A	A	T_het	A	A
nonsynonymous SNV	KIAA1817	chrX	106845063	C	T_het	C	C	C
nonsynonymous SNV	KIAA1864	chr1	160087	C	T_het	T_het	T_hom	T_hom
nonsynonymous SNV	KIAA1875	chr8	145171045	G	G	C_het	G	G
nonsynonymous SNV	KIAA1908	chr7	1627612	A	G_het	A	A	A
nonsynonymous SNV	KIDINS220	chr2	8873707	C	C	C	C	T_het
nonsynonymous SNV	KIF11	chr10	94396944	G	G	G	T_het	G
nonsynonymous SNV	KIF11	chr10	94397295	A	A	A	A	T_het
nonsynonymous SNV	KIF13B	chr8	29025045	C	C	C	T_het	C
nonsynonymous SNV	KIF14	chr1	200574427	A	A	G_het	A	A
nonsynonymous SNV	KIF16B	chr20	16486801	A	A	G_het	A	A
nonsynonymous SNV	KIF19	chr17	72350385	G	A_het	G	G	G
nonsynonymous SNV	KIF1C	chr17	4927183	G	G	G	G	A_hom
nonsynonymous SNV	KIF20A	chr5	137521324	G	G	A_het	G	G
nonsynonymous SNV	KIF23	chr15	69729049	T	T	C_het	T	T
nonsynonymous SNV	KIF26A	chr14	104638087	G	G	A_het	G	G
nonsynonymous SNV	KIF26B	chr1	245849488	T	T	C_het	T	T
nonsynonymous SNV	KIF27	chr9	86506413	C	C	T_het	C	C
nonsynonymous SNV	KIF2B	chr17	51901163	C	C	C	C	T_hom
nonsynonymous SNV	KIF3C	chr2	26178525	C	C	T_het	C	C
nonsynonymous SNV	KIF4B	chr5	154396459	C	C	A_het	C	C
nonsynonymous SNV	KIF5C	chr2	149847588	T	C_het	T	T	T
nonsynonymous SNV	KIF9	chr3	47315121	C	C	C	C	G_het
nonsynonymous SNV	KIR2DL5B	chr19	88563	C	C	T_het	C	C
nonsynonymous SNV	KIR2DL5B	chr19	89632	A	A	G_het	A	A
nonsynonymous SNV	KIR2DL5B	chr19	89698	G	G	A_het	G	G
nonsynonymous SNV	KIR2DL5B	chr19	95527	G	G	G	A_hom	G
nonsynonymous SNV	KIR2DS1	chr19	115133	A	G_hom	A	A	A
nonsynonymous SNV	KIR2DS1	chr19	120467	T	T	T	A_het	T
nonsynonymous SNV	KIR2DS4	chr19	55351014	G	A_het	G	G	G

nonsynonymous SNV	KIR2DS5	chr19	99656	C	C	G_het	C	T_hom
nonsynonymous SNV	KIR2DS5	chr19	99665	T	T	G_het	T	T
nonsynonymous SNV	KIR2DS5	chr19	102175	C	C	G_het	C	C
nonsynonymous SNV	KIR2DS5	chr19	102267	C	C	G_het	C	C
nonsynonymous SNV	KIR2DS5	chr19	102313	G	G	C_het	G	G
nonsynonymous SNV	KIR2DS5	chr19	102337	C	C	G_het	C	C
nonsynonymous SNV	KIR2DS5	chr19	102378	A	A	C_het	A	A
nonsynonymous SNV	KIR2DS5	chr19	102398	G	G	T_het	G	G
nonsynonymous SNV	KIR2DS5	chr19	104128	A	A	A	G_hom	A
nonsynonymous SNV	KIR2DS5	chr19	111816	A	A	G_het	A	A
nonsynonymous SNV	KIR3DL1	chr19	55329814	G	C_het	G	G	G
nonsynonymous SNV	KIR3DS1	chr19	71145	G	G	C_het	G	G
nonsynonymous SNV	KIR3DS1	chr19	71173	A	A	G_het	A	A
nonsynonymous SNV	KIR3DS1	chr19	73897	C	C	T_hom	C	C
nonsynonymous SNV	KISS1	chr1	204159761	G	G	G	C_hom	G
nonsynonymous SNV	KIT	chr4	55593449	A	A	C_het	A	A
stopgain	KLC2	chr11	66033654	C	C	T_het	C	C
nonsynonymous SNV	KLHDC9	chr1	161068949	C	C	C	A_hom	C
nonsynonymous SNV	KLHL29	chr2	23865589	C	C	T_het	C	C
nonsynonymous SNV	KLHL31	chr6	53519755	G	G	G	G	C_het
nonsynonymous SNV	KLHL32	chr6	97533027	A	A	A	A	G_het
stopgain	KLHL34	chrX	21674307	G	G	A_hom	G	G
nonsynonymous SNV	KLHL34	chrX	21674364	C	C	T_hom	C	C
nonsynonymous SNV	KLHL38	chr8	124664848	C	C	T_het	C	C
nonsynonymous SNV	KLHL40	chr3	42727543	T	T	T	T	C_het
nonsynonymous SNV	KLK14	chr19	51582808	G	G	A_het	G	G
nonsynonymous SNV	KLK9	chr19	51509953	T	T	C_het	T	T
nonsynonymous SNV	KLRC2	chr12	10587111	A	G_hom	G_hom	G_hom	G_het
nonsynonymous SNV	KLRC2	chr12	10588530	C	G_het	C	C	C
nonsynonymous SNV	KLRC2	chr12	10588581	T	C_het	T	C_het	C_het
nonsynonymous SNV	KLRG2	chr7	139167994	G	G	G	G	T_hom
nonsynonymous SNV	KMT2A	chr11	118390500	C	C	T_het	C	C
nonsynonymous SNV	KMT2B	chr19	36210925	C	C	T_het	C	C
nonsynonymous SNV	KMT2B	chr19	36219903	C	C	C	G_het	C
nonsynonymous SNV	KMT2C	chr7	151874580	A	A	A	G_het	A
nonsynonymous SNV	KMT2C	chr7	151878616	G	G	A_het	G	G
nonsynonymous SNV	KMT2C	chr7	151927021	C	A_het	A_het	A_het	A_het
nonsynonymous SNV	KMT2C	chr7	151932916	C	C	C	G_het	C
nonsynonymous SNV	KMT2C	chr7	151935853	T	T	T	T	C_het
nonsynonymous SNV	KMT2C	chr7	151945007	C	T_het	T_het	C	T_het
nonsynonymous SNV	KMT2C	chr7	151945204	G	A_het	A_het	A_het	G
nonsynonymous SNV	KMT2C	chr7	151970856	T	A_het	A_het	A_het	A_het
nonsynonymous SNV	KMT2D	chr12	49444046	C	T_het	C	C	C
nonsynonymous SNV	KMT2D	chr12	49445728	G	G	A_het	G	G
nonsynonymous SNV	KPNA3	chr13	50276568	T	T	T	T	C_het

nonsynonymous SNV	KRAS	chr12	25398284	C	C	C	C	A_het
nonsynonymous SNV	KRBA1	chr7	149420938	C	C	C	T_het	C
nonsynonymous SNV	KRBA1	chr7	149422444	G	A_het	G	G	G
nonsynonymous SNV	KRBA1	chr7	149430421	G	G	A_het	G	G
nonsynonymous SNV	KREMEN2	chr16	3017453	C	C	C	T_het	C
nonsynonymous SNV	KRT16	chr17	39767176	C	C	T_het	C	C
nonsynonymous SNV	KRT16	chr17	39768820	G	G	G	A_hom	G
nonsynonymous SNV	KRT18	chr12	53343007	G	A_het	G	A_het	G
nonsynonymous SNV	KRT18	chr12	53343033	G	A_het	A_het	G	G
nonsynonymous SNV	KRT18	chr12	53343036	C	T_het	T_het	C	C
nonsynonymous SNV	KRT18	chr12	53343040	C	A_het	A_het	C	C
nonsynonymous SNV	KRT18	chr12	53343051	G	T_het	T_het	G	G
nonsynonymous SNV	KRT18	chr12	53343059	C	A_het	A_het	C	C
nonsynonymous SNV	KRT18	chr12	53343069	G	T_het	T_het	G	G
nonsynonymous SNV	KRT18	chr12	53343084	G	G	C_het	G	G
nonsynonymous SNV	KRT18	chr12	53343099	G	G	A_het	G	G
nonsynonymous SNV	KRT18	chr12	53343105	C	C	T_het	C	C
nonsynonymous SNV	KRT18	chr12	53343124	G	G	A_het	G	G
nonsynonymous SNV	KRT2	chr12	53040704	C	C	C	T_het	C
nonsynonymous SNV	KRT25	chr17	38907448	C	C	T_het	C	C
nonsynonymous SNV	KRT31	chr17	39551532	C	C	A_het	C	C
nonsynonymous SNV	KRT38	chr17	39594525	C	C	T_het	C	C
stopgain	KRT38	chr17	39595484	G	G	A_het	G	G
nonsynonymous SNV	KRT6A	chr12	52885316	A	A	A	A	G_het
nonsynonymous SNV	KRT6B	chr12	52842736	T	C_het	C_het	C_het	C_het
nonsynonymous SNV	KRT6B	chr12	52844265	T	C_het	C_het	C_het	C_het
nonsynonymous SNV	KRT6B	chr12	52845435	C	T_het	C	C	C
nonsynonymous SNV	KRT6B	chr12	52845801	T	C_het	C_het	C_het	C_het
nonsynonymous SNV	KRT6C	chr12	52862928	C	C	G_het	C	C
nonsynonymous SNV	KRT6C	chr12	52867094	C	T_het	C	C	C
nonsynonymous SNV	KRT75	chr12	52818419	C	C	C	T_het	C
nonsynonymous SNV	KRT75	chr12	52827889	C	C	T_het	C	C
nonsynonymous SNV	KRT79	chr12	53225332	C	C	T_het	C	C
nonsynonymous SNV	KRT8	chr12	53298579	T	C_het	T	T	T
nonsynonymous SNV	KRT84	chr12	52772132	C	C	C	T_het	C
nonsynonymous SNV	KRT84	chr12	52775167	G	A_het	G	G	G
nonsynonymous SNV	KRT8P41	chr11	9116703	G	G	T_het	G	G
nonsynonymous SNV	KRT9	chr17	39727808	C	C	C	C	T_hom
nonsynonymous SNV	KRTAP10-4	chr21	45994110	A	G_het	G_het	A	A
nonsynonymous SNV	KRTAP10-6	chr21	46012160	G	G	G	G	C_het
nonsynonymous SNV	KRTAP10-7	chr21	46020867	A	G_hom	G_hom	G_hom	G_hom
nonsynonymous SNV	KRTAP10-8	chr21	46032769	G	G	A_het	G	G
stopgain	KRTAP10-9	chr21	46047694	C	C	A_het	C	C
stopgain	KRTAP1-1	chr17	39197499	G	G	G	G	A_het
nonsynonymous SNV	KRTAP1-3	chr17	39190758	T	T	T	T	C_hom

nonsynonymous SNV	KRTAP19-5	chr21	31874240	T	T	T	C_het	T
nonsynonymous SNV	KRTAP26-1	chr21	31692339	G	G	G	T_het	G
nonsynonymous SNV	KRTAP4-11	chr17	39274426	T	A_het	T	A_het	A_het
nonsynonymous SNV	KRTAP4-11	chr17	39274435	A	A	A	T_het	A
nonsynonymous SNV	KRTAP4-11	chr17	39274437	C	C	C	T_het	T_het
nonsynonymous SNV	KRTAP4-3	chr17	39324333	T	T	A_het	T	T
nonsynonymous SNV	KRTAP4-6	chr17	39296361	A	G_het	A	G_het	G_het
nonsynonymous SNV	KRTAP4-6	chr17	39296412	T	A_het	T	A_het	A_het
nonsynonymous SNV	KRTAP4-7	chr17	39240804	C	C	C	A_het	A_het
nonsynonymous SNV	KRTAP4-7	chr17	39240805	G	G	G	T_het	T_het
nonsynonymous SNV	KRTAP4-7	chr17	39240807	C	C	C	T_het	T_het
nonsynonymous SNV	KRTAP4-7	chr17	39240810	T	A_het	T	A_het	A_het
nonsynonymous SNV	KRTAP5-5	chr11	1651120	G	G	G	G	T_het
nonsynonymous SNV	KRTAP9-2	chr17	39383012	C	C	T_het	T_het	T_hom
nonsynonymous SNV	KRTAP9-4	chr17	39406343	A	A	A	G_het	A
nonsynonymous SNV	KRTAP9-4	chr17	39406427	T	T	T	C_het	T
nonsynonymous SNV	KSR1	chr17	25904846	G	G	G	G	T_hom
nonsynonymous SNV	KY	chr3	134338056	G	A_het	G	G	G
nonsynonymous SNV	L3HYPDH	chr14	59950730	G	G	G	G	T_het
nonsynonymous SNV	LAMA1	chr18	6977821	G	G	G	A_hom	G
nonsynonymous SNV	LAMA1	chr18	7032153	C	C	T_het	C	C
nonsynonymous SNV	LAMA2	chr6	129663525	G	G	G	A_hom	G
nonsynonymous SNV	LAMA3	chr18	21483925	A	A	A	A	G_het
nonsynonymous SNV	LAMA5	chr20	60885242	C	C	T_het	C	C
nonsynonymous SNV	LAMA5	chr20	60885365	G	G	G	A_het	G
nonsynonymous SNV	LAMA5	chr20	60890243	C	C	C	T_het	C
nonsynonymous SNV	LAMA5	chr20	60893615	C	C	C	G_het	C
nonsynonymous SNV	LAMA5	chr20	60893635	C	T_hom	C	C	C
nonsynonymous SNV	LAMA5	chr20	60894806	C	C	C	T_het	C
nonsynonymous SNV	LAMA5	chr20	60898581	T	T	T	T	C_hom
nonsynonymous SNV	LAMB1	chr7	107602034	C	C	C	T_het	C
nonsynonymous SNV	LAMB2	chr3	49162819	G	G	T_hom	G	G
nonsynonymous SNV	LAMC1	chr1	183102632	G	A_het	G	G	G
nonsynonymous SNV	LAMC2	chr1	183187550	G	G	G	G	C_het
nonsynonymous SNV	LAMTOR5	chr1	110948948	T	T	A_het	T	T
nonsynonymous SNV	LAMTOR5	chr1	110950295	C	C	C	A_hom	C
nonsynonymous SNV	LANCL1	chr2	211336713	G	G	G	A_het	G
nonsynonymous SNV	LANCL2	chr7	55433733	G	G	G	G	A_hom
nonsynonymous SNV	LARP1	chr5	154135634	C	C	C	T_het	C
nonsynonymous SNV	LARS	chr5	145538987	C	C	C	C	T_het
nonsynonymous SNV	LARS2	chr3	45527241	T	T	T	T	C_het
nonsynonymous SNV	LATS2	chr13	21562558	G	G	G	G	A_hom
nonsynonymous SNV	LAX1	chr1	203743520	G	G	G	G	C_het
nonsynonymous SNV	LBX1	chr10	102988296	T	C_het	T	T	T
nonsynonymous SNV	LCAT	chr16	67976320	A	T_het	A	A	A

nonsynonymous SNV	LCMT1	chr16	25137385	G	A_het	A_het	G	A_het
nonsynonymous SNV	LCN12	chr9	139849847	G	G	G	C_hom	G
nonsynonymous SNV	LCN15	chr9	139657893	C	C	T_het	C	C
stopgain	LCNL1	chr9	139879185	C	C	T_het	C	C
nonsynonymous SNV	LDHD	chr16	75150579	G	G	G	G	A_het
nonsynonymous SNV	LEFTY1	chr1	226076670	G	A_hom	G	G	G
nonsynonymous SNV	LEKR1	chr3	156697028	C	C	T_het	C	C
nonsynonymous SNV	LEPREL2	chr12	6943136	C	C	C	G_het	C
nonsynonymous SNV	LGALS3BP	chr17	76973249	C	C	C	T_hom	C
nonsynonymous SNV	LIFR	chr5	38502757	G	G	A_het	G	G
nonsynonymous SNV	LILRA1	chr19	55106343	G	G	A_het	G	G
nonsynonymous SNV	LILRA2	chr19	55107343	T	T	T	T	C_het
nonsynonymous SNV	LILRA6	chr19	54723995	G	G	G	G	C_het
nonsynonymous SNV	LILRA6	chr19	54743747	C	C	C	G_het	C
nonsynonymous SNV	LILRA6	chr19	54743758	C	A_het	A_het	A_het	C
nonsynonymous SNV	LILRA6	chr19	54744387	C	C	T_het	T_het	C
nonsynonymous SNV	LILRA6	chr19	54744722	T	T	C_het	C_het	T
nonsynonymous SNV	LILRA6	chr19	54744794	G	G	A_het	A_het	G
nonsynonymous SNV	LILRA6	chr19	54744799	G	G	C_het	C_het	G
nonsynonymous SNV	LILRB1	chr19	55148249	G	A_het	A_het	G	G
nonsynonymous SNV	LILRB2	chr19	54782754	A	A	A	G_het	A
nonsynonymous SNV	LILRB3	chr19	54726299	A	A	C_het	A	A
nonsynonymous SNV	LILRB3	chr19	54726324	G	C_hom	G	G	G
nonsynonymous SNV	LILRB3	chr19	54726833	T	T	T	C_het	C_het
nonsynonymous SNV	LILRB3	chr19	54746051	A	A	C_het	C_het	A
nonsynonymous SNV	LILRB3	chr19	54746081	T	C_het	C_het	T	T
nonsynonymous SNV	LILRB4	chr19	55174519	G	G	G	C_het	G
nonsynonymous SNV	LILRB4	chr19	55176381	C	C	T_het	C	C
nonsynonymous SNV	LILRB4	chr19	55177373	A	A	A	G_het	A
nonsynonymous SNV	LILRB4	chr19	55179145	A	A	A	T_het	A
nonsynonymous SNV	LIMCH1	chr4	41687843	C	C	C	C	T_het
nonsynonymous SNV	LIMK2	chr22	31671153	A	A	G_het	A	A
nonsynonymous SNV	LIMS1	chr2	109276099	G	G	A_hom	G	G
nonsynonymous SNV	LINC00115	chr1	762330	G	G	G	T_het	G
nonsynonymous SNV	LINC00115	chr1	762345	A	A	A	G_het	A
nonsynonymous SNV	LINC00207	chr22	44967283	G	G	T_het	G	G
nonsynonymous SNV	LINC00273	chr16	33961363	C	C	T_het	C	C
nonsynonymous SNV	LINC00273	chr16	33961386	G	A_het	G	G	G
nonsynonymous SNV	LINC00273	chr16	33961400	G	G	T_het	G	G
nonsynonymous SNV	LINC00273	chr16	33961453	A	G_het	C_het	A	A
nonsynonymous SNV	LINC00273	chr16	33961465	G	A_het	G	G	G
nonsynonymous SNV	LINC00273	chr16	33961471	C	C	A_het	C	C
nonsynonymous SNV	LINC00273	chr16	33961474	G	G	C_het	G	G
nonsynonymous SNV	LINC00273	chr16	33961481	A	C_het	A	A	A
nonsynonymous SNV	LINC00273	chr16	33961482	T	C_het	T	T	T

nonsynonymous SNV	LINC00273	chr16	33961483	G	T_het	G	G	G
nonsynonymous SNV	LINC00273	chr16	33961499	C	T_het	C	C	C
nonsynonymous SNV	LINC00273	chr16	33961500	A	G_het	A	A	G_het
nonsynonymous SNV	LINC00273	chr16	33961539	A	C_het	C_het	C_het	C_het
nonsynonymous SNV	LINC00273	chr16	33961563	C	A_het	A_het	G_het	A_het
nonsynonymous SNV	LINC00273	chr16	33961564	A	C_het	C_het	A	C_het
nonsynonymous SNV	LINC00273	chr16	33961581	G	T_het	T_het	G	G
nonsynonymous SNV	LINC00273	chr16	33961597	A	C_het	C_het	A	A
nonsynonymous SNV	LINC00273	chr16	33961621	G	A_het	A_het	G	G
nonsynonymous SNV	LINC00273	chr16	33961627	C	C	C	C	T_het
nonsynonymous SNV	LINC00273	chr16	33961635	T	T	T	T	A_het
nonsynonymous SNV	LINC00273	chr16	33961648	G	G	G	G	C_het
nonsynonymous SNV	LINC00273	chr16	33961659	G	A_het	A_het	A_het	A_het
nonsynonymous SNV	LINC00273	chr16	33961668	C	C	T_het	C	C
nonsynonymous SNV	LINC00273	chr16	33961671	C	A_het	A_het	A_het	A_het
nonsynonymous SNV	LINC00273	chr16	33961672	T	G_het	G_het	G_het	A_het
nonsynonymous SNV	LINC00273	chr16	33961689	G	A_het	A_het	A_het	A_het
nonsynonymous SNV	LINC00273	chr16	33961690	C	G_het	G_het	G_het	G_het
stopgain	LINC00273	chr16	33961702	C	A_het	A_het	A_het	A_het
nonsynonymous SNV	LINC00273	chr16	33961705	A	G_het	G_het	G_het	G_het
nonsynonymous SNV	LINC00273	chr16	33961714	G	G	A_het	G	G
nonsynonymous SNV	LINC00273	chr16	33961725	G	G	A_het	G	G
nonsynonymous SNV	LINC00273	chr16	33961749	G	G	G	G	A_het
nonsynonymous SNV	LINC00273	chr16	33961752	T	C_het	C_het	C_het	C_het
nonsynonymous SNV	LINC00273	chr16	33961753	T	G_het	G_het	G_het	G_het
nonsynonymous SNV	LINC00273	chr16	33961759	G	C_het	G	G	G
nonsynonymous SNV	LINC00273	chr16	33961768	T	C_het	T	G_het	T
nonsynonymous SNV	LINC00273	chr16	33961779	C	C	C	A_het	C
nonsynonymous SNV	LINC00273	chr16	33961794	C	T_het	C	C	C
nonsynonymous SNV	LINC00273	chr16	33961846	C	C	C	C	G_het
nonsynonymous SNV	LINC00273	chr16	33961855	T	G_het	T	G_het	G_het
nonsynonymous SNV	LINC00273	chr16	33961869	G	G	G	G	A_het
nonsynonymous SNV	LINC00273	chr16	33961873	G	A_het	G	A_het	G
nonsynonymous SNV	LINC00273	chr16	33961878	A	G_het	A	G_het	A
nonsynonymous SNV	LINC00273	chr16	33961881	A	G_het	A	G_het	G_het
nonsynonymous SNV	LINC00273	chr16	33961902	C	A_het	C	A_het	C
nonsynonymous SNV	LINC00273	chr16	33961936	G	A_het	G	G	G
nonsynonymous SNV	LINC00273	chr16	33961938	T	C_het	T	T	T
nonsynonymous SNV	LINC00273	chr16	33961944	G	C_het	G	G	G
nonsynonymous SNV	LINC00552	chr13	114453668	C	C	A_het	C	C
nonsynonymous SNV	LINC00636	chr3	107646722	C	C	C	A_hom	C
nonsynonymous SNV	LINC00893	chrX	148615539	C	T_hom	C	C	C
nonsynonymous SNV	LMF1	chr16	1004625	T	T	T	G_het	T
nonsynonymous SNV	LMNA	chr1	156108510	C	T_het	C	C	C
nonsynonymous SNV	LNPEP	chr5	96314971	G	G	G	A_het	G

nonsynonymous SNV	LNX2	chr13	28143388	C	C	T_het	C	C
nonsynonymous SNV	LOC100128881	chr16	89784193	G	G	G	G	A_het
nonsynonymous SNV	LOC100128881	chr16	89784336	C	C	T_het	T_hom	T_het
nonsynonymous SNV	LOC100131060	chr1	59359231	T	T	T	T	C_het
nonsynonymous SNV	LOC100132247	chr16	22545897	G	T_het	T_het	G	T_het
nonsynonymous SNV	LOC100133957	chrX	47518695	G	G	C_hom	G	G
nonsynonymous SNV	LOC100144595	chr2	155294269	A	A	A	G_het	A
nonsynonymous SNV	LOC100233156	Unknown	38873	G	T_het	T_het	G	T_het
nonsynonymous SNV	LOC100233156	Unknown	38876	T	G_het	G_het	T	G_het
nonsynonymous SNV	LOC100233156	Unknown	38943	A	G_het	G_het	A	G_het
nonsynonymous SNV	LOC100233156	Unknown	39014	A	G_het	G_het	G_het	G_het
nonsynonymous SNV	LOC100233156	Unknown	39922	A	G_het	G_het	G_het	G_het
nonsynonymous SNV	LOC100233156	Unknown	40673	A	A	A	A	G_het
nonsynonymous SNV	LOC100233156	Unknown	40716	C	C	T_het	T_het	T_het
nonsynonymous SNV	LOC100233156	Unknown	40803	C	T_het	T_het	T_het	T_het
nonsynonymous SNV	LOC100233156	Unknown	40814	T	C_het	C_het	C_het	C_het
nonsynonymous SNV	LOC100233156	Unknown	40845	G	A_het	G	G	G
nonsynonymous SNV	LOC100288332	chr16	15457835	T	T	T	C_het	T
nonsynonymous SNV	LOC100288524	chr7	331050	G	G	A_het	G	G
nonsynonymous SNV	LOC100288966	Unknown	119633	C	C	C	T_hom	C
nonsynonymous SNV	LOC100289511	chr14	70234336	G	A_het	G	G	G
nonsynonymous SNV	LOC100507003	chr19	49930352	C	A_het	C	C	C
nonsynonymous SNV	LOC150935	chr2	240721558	G	G	G	A_het	G
nonsynonymous SNV	LOC283440	chr12	2877094	G	A_het	G	G	G
nonsynonymous SNV	LOC283788	Unknown	78859	T	A_het	A_hom	A_het	A_hom
nonsynonymous SNV	LOC283788	Unknown	88326	A	A	A	G_het	A
nonsynonymous SNV	LOC283922	chr16	74366531	A	C_het	C_het	C_het	A
nonsynonymous SNV	LOC284023	chr17	7819195	T	T	C_hom	T	T
nonsynonymous SNV	LOC284889	chr22	24237333	G	G	G	G	A_het
nonsynonymous SNV	LOC285548	chr4	13548556	G	G	A_het	G	G
nonsynonymous SNV	LOC388692	chr1	149279739	C	T_het	T_het	T_het	T_het
nonsynonymous SNV	LOC389831	chr7	48976	C	T_hom	C	C	C
nonsynonymous SNV	LOC399753	chr10	49218553	T	T	T	C_hom	T
nonsynonymous SNV	LOC400927	chr22	38743585	G	G	G	A_hom	G
nonsynonymous SNV	LOC401010	chr2	132200697	C	C	C	C	G_het
nonsynonymous SNV	LOC401296	chr7	1732965	C	C	A_het	C	C
nonsynonymous SNV	LOC440243	chr15	23572552	T	T	T	C_het	T
nonsynonymous SNV	LOC440311	chr15	95398950	C	C	C	G_het	C
nonsynonymous SNV	LOC440563	chr1	13183176	C	C	C	T_het	C
nonsynonymous SNV	LOC440563	chr1	13183187	A	A	G_het	G_het	A
nonsynonymous SNV	LOC440563	chr1	13183188	T	T	T	A_het	T
nonsynonymous SNV	LOC440563	chr1	13183199	C	C	C	G_het	C
nonsynonymous SNV	LOC440563	chr1	13183238	T	C_het	C_het	C_het	C_het
nonsynonymous SNV	LOC440563	chr1	13183248	T	A_het	A_het	A_het	A_het
nonsynonymous SNV	LOC440563	chr1	13183511	T	C_het	C_het	C_het	C_het

nonsynonymous SNV	LOC440563	chr1	13183528	A	C_het	C_het	C_het	C_het
nonsynonymous SNV	LOC440563	chr1	13183532	C	T_het	T_het	T_het	T_het
nonsynonymous SNV	LOC440563	chr1	13183533	C	T_het	T_het	T_het	T_het
nonsynonymous SNV	LOC441081	chr5	69784578	G	G	G	G	C_hom
nonsynonymous SNV	LOC441081	chr5	69784611	T	T	T	T	G_hom
nonsynonymous SNV	LOC441081	chr5	69784650	C	C	G_het	C	G_hom
nonsynonymous SNV	LOC642846	chr12	9447488	C	T_het	T_het	C	C
nonsynonymous SNV	LOC642846	chr12	9451397	G	G	G	G	A_het
nonsynonymous SNV	LOC642846	chr12	9451473	G	G	G	G	A_het
nonsynonymous SNV	LOC643339	chr12	93398348	C	C	C	T_het	C
nonsynonymous SNV	LOC643406	chr20	5454416	G	G	G	A_het	G
nonsynonymous SNV	LOC643699	chr15	30700159	A	A	G_het	G_het	G_het
nonsynonymous SNV	LOC644145	chr4	56703285	T	T	T	T	A_het
nonsynonymous SNV	LOC653513	chr1	144994658	C	A_het	A_het	A_het	A_het
nonsynonymous SNV	LOC727924	chr15	22382655	A	A	C_het	C_het	A
nonsynonymous SNV	LOC727924	chr15	22382821	G	G	C_het	C_het	G
nonsynonymous SNV	LOC727924	chr15	22382828	C	C	A_het	C	C
nonsynonymous SNV	LOC727924	chr15	22382897	A	A	A	G_het	A
nonsynonymous SNV	LOC729732	chr8	12458862	G	G	C_hom	G	G
nonsynonymous SNV	LOC90925	chr14	107259734	C	C	T_het	C	C
nonsynonymous SNV	LOX	chr5	121413205	G	G	T_het	G	G
nonsynonymous SNV	LOXHD1	chr18	44181286	C	C	T_het	C	C
nonsynonymous SNV	LOXL2	chr8	23155614	G	G	G	A_het	G
nonsynonymous SNV	LOXL2	chr8	23217736	G	G	A_het	G	G
nonsynonymous SNV	LPA	chr6	160968913	C	C	C	A_het	C
nonsynonymous SNV	LPPR3	chr19	815719	G	G	G	A_het	G
nonsynonymous SNV	LRFN1	chr19	39805528	C	C	T_het	C	C
nonsynonymous SNV	LRFN2	chr6	40359689	G	G	A_het	G	G
nonsynonymous SNV	LRIG3	chr12	59272737	C	C	T_het	C	C
nonsynonymous SNV	LRP1	chr12	57539082	C	C	T_het	C	C
nonsynonymous SNV	LRP1	chr12	57603544	G	G	G	A_het	G
nonsynonymous SNV	LRP12	chr8	105509318	T	T	T	C_het	T
nonsynonymous SNV	LRP1B	chr2	140990778	C	C	C	A_het	C
stopgain	LRP1B	chr2	141200148	T	T	A_het	T	T
nonsynonymous SNV	LRP1B	chr2	141665483	C	C	C	C	G_het
nonsynonymous SNV	LRP2	chr2	169996070	C	C	T_het	C	C
nonsynonymous SNV	LRP2	chr2	170026232	G	G	A_het	G	G
nonsynonymous SNV	LRP2	chr2	170044551	C	C	T_het	C	C
nonsynonymous SNV	LRP3	chr19	33696281	C	C	T_het	C	C
nonsynonymous SNV	LRP5	chr11	68193587	G	G	A_het	G	G
nonsynonymous SNV	LRP5L	chr22	25747801	C	C	C	T_hom	C
nonsynonymous SNV	LRR1	chr14	50080982	A	A	A	A	G_het
nonsynonymous SNV	LRRC10B	chr11	61276738	C	C	T_het	C	C
stopgain	LRRC14B	chr5	191824	G	G	A_het	G	G
nonsynonymous SNV	LRRC14B	chr5	192228	G	C_het	G	G	G

nonsynonymous SNV	LRRC2	chr3	46563069	G	G	G	A_hom	G
nonsynonymous SNV	LRRC23	chr12	7021983	C	C	C	C	T_het
nonsynonymous SNV	LRRC29	chr16	67241545	C	C	C	T_het	C
nonsynonymous SNV	LRRC32	chr11	76370925	T	T	C_het	T	T
nonsynonymous SNV	LRRC37B	chr17	30372739	C	C	T_het	C	C
nonsynonymous SNV	LRRC3C	chr17	38100769	G	G	G	G	A_hom
nonsynonymous SNV	LRRC4C	chr11	40136573	A	A	G_het	A	A
nonsynonymous SNV	LRRC4C	chr11	40137550	T	A_het	T	T	T
nonsynonymous SNV	LRRC52	chr1	165532948	C	T_het	C	C	C
nonsynonymous SNV	LRRC7	chr1	70488932	G	G	G	G	A_het
nonsynonymous SNV	LRRC70	chr5	61876773	C	C	T_het	C	C
nonsynonymous SNV	LRRC73	chr6	43475272	C	C	C	A_hom	C
nonsynonymous SNV	LRRC8B	chr1	90049333	A	A	G_het	A	A
nonsynonymous SNV	LRRC8C	chr1	90180152	C	C	A_het	C	C
nonsynonymous SNV	LRRC8E	chr19	7964899	G	G	A_het	G	G
nonsynonymous SNV	LRRD1	chr7	91794308	G	G	G	A_het	G
nonsynonymous SNV	LRRFIP2	chr3	37151157	T	C_hom	T	T	T
nonsynonymous SNV	LRRIQ1	chr12	85449658	G	G	G	A_het	G
nonsynonymous SNV	LRRIQ1	chr12	85450115	C	C	C	C	A_het
nonsynonymous SNV	LRRIQ4	chr3	169546574	A	A	G_het	A	A
nonsynonymous SNV	LRRIQ4	chr3	169555396	C	T_het	C	C	C
stopgain	LRRK2	chr12	40702279	C	C	C	T_het	C
nonsynonymous SNV	LRRN1	chr3	3887535	G	G	G	G	A_hom
nonsynonymous SNV	LSMD1	chr17	7760537	G	G	G	G	A_hom
nonsynonymous SNV	LSP1	chr11	1887894	C	C	T_het	C	C
nonsynonymous SNV	LSR	chr19	35757867	C	C	T_het	C	C
nonsynonymous SNV	LTBP1	chr2	33246060	C	C	T_het	C	C
nonsynonymous SNV	LTBP2	chr14	74971769	C	C	C	C	T_het
nonsynonymous SNV	LTF	chr3	46501168	A	A	A	T_hom	A
nonsynonymous SNV	LUC7L2	chr7	139106990	C	C	C	C	A_hom
nonsynonymous SNV	LUZP1	chr1	23415438	G	G	G	A_hom	G
nonsynonymous SNV	LYPLAL1	chr1	219366593	G	A_het	G	G	G
nonsynonymous SNV	LYRM1	chr16	20935371	G	G	A_het	G	G
nonsynonymous SNV	LYST	chr1	235872576	G	G	A_het	G	G
nonsynonymous SNV	LYST	chr1	235976273	G	G	G	G	A_het
nonsynonymous SNV	LYZL2	chr10	30918549	G	G	C_het	C_het	C_het
nonsynonymous SNV	LZTS2	chr10	102766851	G	G	G	G	C_het
nonsynonymous SNV	MAATS1	chr3	119421952	C	C	C	C	A_het
nonsynonymous SNV	MACF1	chr1	39758439	G	G	G	T_hom	G
nonsynonymous SNV	MACF1	chr1	39823377	C	C	C	T_hom	C
nonsynonymous SNV	MAGEB16	chrX	35820536	G	G	A_hom	G	G
nonsynonymous SNV	MAGEE1	chrX	75649404	G	G	G	A_hom	G
nonsynonymous SNV	MAGEF1	chr3	184429305	A	A	A	A	G_het
nonsynonymous SNV	MAGEL2	chr15	23892423	C	C	T_het	C	C
nonsynonymous SNV	MAGI1	chr3	65372883	G	G	A_hom	G	G

nonsynonymous SNV	MAGI2	chr7	77756585	G	G	G	T_het	G
nonsynonymous SNV	MAGI3	chr1	113933672	A	A	C_het	A	A
nonsynonymous SNV	MAK	chr6	10804044	G	G	A_het	G	G
nonsynonymous SNV	MAMDC4	chr9	139749554	C	C	T_het	C	C
nonsynonymous SNV	MAMDC4	chr9	139751625	C	C	C	A_hom	C
nonsynonymous SNV	MAMDC4	chr9	139752899	T	G_hom	G_het	T	T
nonsynonymous SNV	MAML1	chr5	179193441	C	C	A_het	C	C
nonsynonymous SNV	MAMLD1	chrX	149638720	C	C	T_hom	C	C
nonsynonymous SNV	MAN1B1	chr9	139996489	T	C_het	T	T	T
nonsynonymous SNV	MAN1C1	chr1	26073242	G	G	A_het	G	G
nonsynonymous SNV	MAN2A1	chr5	109049423	C	C	C	C	A_het
nonsynonymous SNV	MAN2A2	chr15	91450083	G	G	A_het	G	G
nonsynonymous SNV	MAN2A2	chr15	91461571	C	C	C	C	A_het
nonsynonymous SNV	MAN2B1	chr19	12766564	C	C	T_het	C	C
nonsynonymous SNV	MAN2C1	chr15	75652470	T	T	T	C_het	T
nonsynonymous SNV	MAP1B	chr5	71493069	C	C	C	T_het	C
nonsynonymous SNV	MAP1B	chr5	71495189	G	G	G	G	C_het
nonsynonymous SNV	MAP1B	chr5	71495805	G	G	G	G	A_het
nonsynonymous SNV	MAP1S	chr19	17838106	C	C	T_het	C	C
nonsynonymous SNV	MAP2K3	chr17	21202191	C	A_het	A_het	A_het	A_het
nonsynonymous SNV	MAP2K3	chr17	21202237	G	C_het	C_het	C_het	C_het
nonsynonymous SNV	MAP2K3	chr17	21203893	T	C_het	C_het	C_het	C_het
nonsynonymous SNV	MAP2K3	chr17	21203941	G	G	A_het	A_het	A_het
nonsynonymous SNV	MAP2K3	chr17	21204187	G	T_het	T_het	T_het	T_het
nonsynonymous SNV	MAP2K3	chr17	21204192	C	T_het	T_het	T_het	T_het
stopgain	MAP2K3	chr17	21204210	C	T_het	T_het	T_het	T_het
nonsynonymous SNV	MAP2K3	chr17	21207834	C	C	T_het	T_het	T_het
nonsynonymous SNV	MAP2K3	chr17	21215557	G	A_het	A_het	A_het	A_het
nonsynonymous SNV	MAP2K3	chr17	21217513	G	A_het	A_het	A_het	A_het
nonsynonymous SNV	MAP3K1	chr5	56177843	C	C	G_het	C	C
nonsynonymous SNV	MAP3K15	chrX	19389578	C	A_het	C	C	C
nonsynonymous SNV	MAP3K19	chr2	135743411	G	G	G	G	A_het
nonsynonymous SNV	MAP3K9	chr14	71227765	C	C	T_het	C	C
nonsynonymous SNV	MAP4	chr3	47933942	C	C	C	C	T_het
nonsynonymous SNV	MAP4	chr3	47952635	C	C	C	C	G_het
stopgain	MAP4K2	chr11	64559486	G	G	G	A_het	G
nonsynonymous SNV	MAP4K2	chr11	64569794	G	G	G	G	A_hom
nonsynonymous SNV	MAPK9	chr5	179674426	C	C	T_het	C	C
nonsynonymous SNV	MAPK9	chr5	179707528	T	T	T	T	C_hom
nonsynonymous SNV	MAPKBP1	chr15	42111088	C	C	C	T_het	C
stopgain	MAPKBP1	chr15	42114200	G	T_hom	G	G	G
stopgain	MAPRE2	chr18	32585514	C	C	G_het	C	C
nonsynonymous SNV	MAPT	chr17	44073950	G	G	A_hom	G	G
nonsynonymous SNV	MARC1	chr1	220970098	G	A_het	G	G	G
nonsynonymous SNV	MARCH8	chr10	45959729	A	A	A	G_het	A

nonsynonymous SNV	MARCO	chr2	119739075	G	G	A_het	G	G
nonsynonymous SNV	MARCO	chr2	119739941	G	T_het	G	G	G
nonsynonymous SNV	MARK1	chr1	220835187	G	G	T_het	G	G
nonsynonymous SNV	MASP1	chr3	186943297	C	C	A_het	C	C
nonsynonymous SNV	MASP2	chr1	11087272	T	T	T	T	G_het
nonsynonymous SNV	MAST1	chr19	12951867	G	G	A_het	G	G
nonsynonymous SNV	MAST4	chr5	66459743	A	A	A	A	C_het
nonsynonymous SNV	MAVS	chr20	3838459	G	G	G	A_het	G
nonsynonymous SNV	MBOAT7	chr19	54678085	G	G	G	A_het	G
nonsynonymous SNV	MC1R	chr16	89986025	T	T	T	T	C_het
nonsynonymous SNV	MC4R	chr18	58038977	G	G	G	T_hom	G
nonsynonymous SNV	MCIDAS	chr5	54516531	C	C	C	T_het	C
nonsynonymous SNV	MCM8	chr20	5963642	A	A	A	A	G_het
nonsynonymous SNV	MCPH1	chr8	6302738	G	G	G	A_het	G
nonsynonymous SNV	MDH1B	chr2	207620015	C	C	T_het	C	C
nonsynonymous SNV	MDM4	chr1	204518457	A	C_het	A	A	A
nonsynonymous SNV	ME2	chr18	48458727	C	C	T_het	C	C
nonsynonymous SNV	ME3	chr11	86198407	C	C	T_het	C	C
nonsynonymous SNV	MED19	chr11	57479507	C	C	A_het	C	C
nonsynonymous SNV	MED26	chr19	16687505	G	G	G	G	C_het
nonsynonymous SNV	MED6	chr14	71051631	T	T	T	T	C_het
nonsynonymous SNV	MEGF6	chr1	3413275	G	G	A_het	G	G
nonsynonymous SNV	MEGF6	chr1	3427356	C	C	T_het	C	C
nonsynonymous SNV	MEGF8	chr19	42838360	G	G	A_het	G	G
nonsynonymous SNV	MEMO1	chr2	32168402	T	T	T	T	A_het
nonsynonymous SNV	METRN	chr16	767179	C	T_het	C	C	C
nonsynonymous SNV	METRNL	chr17	81052109	C	C	T_het	C	C
nonsynonymous SNV	METTL11B	chr1	170136876	T	T	C_het	T	T
nonsynonymous SNV	METTL13	chr1	171753281	A	A	A	A	C_het
nonsynonymous SNV	METTL23	chr17	74729764	T	C_het	T	T	T
nonsynonymous SNV	METTL2B	chr7	128119514	G	A_het	G	G	G
nonsynonymous SNV	METTL3	chr14	21971392	T	T	T	G_het	T
nonsynonymous SNV	METTL7B	chr12	56077603	G	G	A_het	G	G
nonsynonymous SNV	MEX3D	chr19	1556211	G	G	A_het	G	G
nonsynonymous SNV	MFF	chr2	228207535	C	C	C	T_het	C
nonsynonymous SNV	MFN1	chr3	179069715	G	G	G	G	A_het
nonsynonymous SNV	MFRP	chr11	119213319	C	C	A_het	C	C
nonsynonymous SNV	MFSD2B	chr2	24240405	C	C	C	T_hom	C
nonsynonymous SNV	MGA	chr15	41988737	C	C	T_het	C	C
nonsynonymous SNV	MGAM	chr7	141705413	T	T	C_het	T	T
nonsynonymous SNV	MGAM	chr7	141750579	C	T_het	C	C	C
nonsynonymous SNV	MGAM	chr7	141803238	A	A	A	G_het	A
nonsynonymous SNV	MGAT4B	chr5	179225581	G	G	A_het	G	G
nonsynonymous SNV	MGAT4B	chr5	179226203	C	C	A_het	C	C
nonsynonymous SNV	MGC21881	chr9	41954644	G	G	A_het	A_het	G

nonsynonymous SNV	MGC21881	chr9	41954767	C	C	C	C	G_het
nonsynonymous SNV	MGC21881	chr9	41954776	C	G_hom	C	C	C
nonsynonymous SNV	MIA2	chr14	39722329	A	A	A	G_het	A
nonsynonymous SNV	MIA3	chr1	222838755	G	G	A_het	G	G
nonsynonymous SNV	MICAL2	chr11	12265603	G	G	G	G	A_het
nonsynonymous SNV	MICAL3	chr22	18301508	T	C_het	T	T	T
nonsynonymous SNV	MICALL2	chr7	1484511	C	C	T_het	C	C
nonsynonymous SNV	MID2	chrX	107084062	G	G	A_het	G	G
nonsynonymous SNV	MINPP1	chr10	89268263	C	C	C	A_het	C
nonsynonymous SNV	MIOX	chr22	50926403	C	C	A_het	C	C
nonsynonymous SNV	MIR548F5	chr13	36049858	C	C	T_het	C	C
nonsynonymous SNV	MIRLET7BHG	chr22	46499094	G	G	A_het	G	G
nonsynonymous SNV	MISP	chr19	757241	G	A_het	G	G	G
nonsynonymous SNV	MKI67	chr10	129914025	C	C	T_het	C	C
nonsynonymous SNV	MKI67	chr10	129914103	C	C	T_het	C	C
nonsynonymous SNV	MKL2	chr16	14334126	C	A_het	C	C	C
nonsynonymous SNV	MKX	chr10	27964262	G	G	G	G	T_het
nonsynonymous SNV	MLF1IP	chr4	185637663	G	G	G	A_hom	G
nonsynonymous SNV	MLH3	chr14	75514179	C	C	C	T_het	C
nonsynonymous SNV	MLIP	chr6	53989529	C	C	T_het	C	C
nonsynonymous SNV	MLIP	chr6	54002036	C	T_het	C	C	C
stopgain	MLLT4	chr6	168307929	A	A	A	T_hom	A
nonsynonymous SNV	MLLT4	chr6	168352422	C	C	C	C	A_het
nonsynonymous SNV	MLLT6	chr17	36880921	C	C	T_het	C	C
nonsynonymous SNV	MLST8	chr16	2258790	G	A_het	G	G	G
nonsynonymous SNV	MLYCD	chr16	83948656	G	T_hom	G	G	G
nonsynonymous SNV	MME	chr3	154886301	G	G	G	G	A_het
nonsynonymous SNV	MMP1	chr11	102667879	G	G	G	A_hom	G
nonsynonymous SNV	MMP10	chr11	102650244	A	T_het	A	A	A
nonsynonymous SNV	MMP14	chr14	23311678	C	C	T_het	C	C
nonsynonymous SNV	MMP14	chr14	23313633	G	A_het	G	G	G
stopgain	MMP17	chr12	132323280	C	A_het	C	C	C
nonsynonymous SNV	MMP19	chr12	56233509	A	A	A	A	T_het
nonsynonymous SNV	MMP2	chr16	55536782	G	G	G	C_het	G
nonsynonymous SNV	MMP20	chr11	102464198	G	G	G	G	A_het
nonsynonymous SNV	MMP21	chr10	127462454	C	C	C	T_het	C
nonsynonymous SNV	MMP25	chr16	3100485	A	G_het	A	A	A
nonsynonymous SNV	MMP27	chr11	102575379	T	C_het	T	T	T
nonsynonymous SNV	MMP28	chr17	34093721	C	C	T_het	C	C
nonsynonymous SNV	MMP7	chr11	102395660	G	A_het	G	G	G
nonsynonymous SNV	MMP7	chr11	102395661	C	T_het	C	C	C
nonsynonymous SNV	MMP7	chr11	102398587	A	A	A	A	G_het
nonsynonymous SNV	MMRN2	chr10	88702868	C	C	G_het	C	C
nonsynonymous SNV	MMS19	chr10	99258048	C	C	T_het	C	C
nonsynonymous SNV	MNF1	chr6	33665503	A	A	A	A	G_het

nonsynonymous SNV	MOB3C	chr1	47078638	G	G	A_het	G	G
nonsynonymous SNV	MOCOS	chr18	33837033	C	C	C	C	T_het
stopgain	MOK	chr14	102749873	G	A_het	G	G	G
nonsynonymous SNV	MON1B	chr16	77227653	G	G	A_het	G	G
stoploss	MPDU1	chr17	7490761	G	G	C_het	G	G
nonsynonymous SNV	MPO	chr17	56355299	C	C	T_het	C	C
nonsynonymous SNV	MPO	chr17	56355397	G	G	A_het	G	G
nonsynonymous SNV	MPP4	chr2	202521040	G	G	G	G	C_het
nonsynonymous SNV	MPRIP	chr17	17034996	T	T	C_het	T	T
nonsynonymous SNV	MPZL2	chr11	118133173	C	C	C	C	T_hom
nonsynonymous SNV	MR1	chr1	181018212	G	A_het	G	G	G
nonsynonymous SNV	MRE11A	chr11	94224031	C	C	C	T_hom	C
nonsynonymous SNV	MRGPRE	chr11	3249990	C	C	C	C	T_het
nonsynonymous SNV	MRGPRX1	chr11	18956196	C	C	T_het	T_het	C
nonsynonymous SNV	MRGPRX2	chr11	19077769	T	T	T	T	C_het
nonsynonymous SNV	MRM1	chr17	34958573	C	C	G_het	C	C
nonsynonymous SNV	MRO	chr18	48346281	G	G	G	G	T_het
nonsynonymous SNV	MROH2B	chr5	41018011	G	G	A_het	G	G
stopgain	MROH8	chr20	35766358	C	C	A_het	C	C
nonsynonymous SNV	MRPL15	chr8	55060086	G	G	G	A_het	G
nonsynonymous SNV	MRPL16	chr11	59574082	C	C	A_het	C	C
nonsynonymous SNV	MRPL28	chr16	420116	C	C	T_het	C	C
nonsynonymous SNV	MRPL47	chr3	179322402	G	G	A_het	G	G
nonsynonymous SNV	MRPS14	chr1	174983935	C	C	C	C	T_het
nonsynonymous SNV	MRPS28	chr8	80942341	C	C	C	G_het	C
nonsynonymous SNV	MRPS30	chr5	44809446	G	C_het	C_het	G	G
nonsynonymous SNV	MS4A13	chr11	60285564	G	A_het	G	G	G
nonsynonymous SNV	MS4A3	chr11	59837695	A	A	G_het	A	A
nonsynonymous SNV	MSH2	chr2	47643457	G	A_het	G	G	G
nonsynonymous SNV	MSH2	chr2	47656972	C	C	C	C	T_het
nonsynonymous SNV	MSH2	chr2	47739533	G	G	A_het	G	G
nonsynonymous SNV	MSLN	chr16	814677	C	C	C	A_het	C
nonsynonymous SNV	MSS51	chr10	75188033	G	G	G	G	A_het
nonsynonymous SNV	MST1	chr3	49723141	A	A	A	A	G_hom
nonsynonymous SNV	MST1	chr3	49723379	T	T	T	C_het	T
nonsynonymous SNV	MST1	chr3	49723784	C	C	A_het	C	A_het
nonsynonymous SNV	MST1	chr3	49723823	A	A	A	G_het	A
nonsynonymous SNV	MST1	chr3	49725034	T	T	A_het	T	T
nonsynonymous SNV	MST1	chr3	49725038	G	G	C_het	G	G
nonsynonymous SNV	MST1	chr3	49726070	G	A_het	A_het	A_het	A_het
nonsynonymous SNV	MST1L	chr1	17084968	G	G	C_het	C_het	C_het
nonsynonymous SNV	MST1L	chr1	17085006	C	T_het	C	C	C
nonsynonymous SNV	MST1L	chr1	17085427	T	C_het	T	T	C_het
nonsynonymous SNV	MST1L	chr1	17085502	T	C_het	C_het	C_het	C_het
nonsynonymous SNV	MST1L	chr1	17085564	A	G_het	G_het	G_het	G_het

nonsynonymous SNV	MST1L	chr1	17085791	G	G	G	A_het	G
nonsynonymous SNV	MST1L	chr1	17087262	C	C	C	C	T_het
nonsynonymous SNV	MST1L	chr1	17087292	A	A	G_het	A	G_het
nonsynonymous SNV	MST1L	chr1	17087465	G	A_het	A_het	A_het	A_het
nonsynonymous SNV	MST1L	chr1	17087582	G	A_het	G	G	A_het
nonsynonymous SNV	MSX1	chr4	4864429	G	G	G	G	T_het
nonsynonymous SNV	MTBP	chr8	121466092	A	A	A	G_het	A
nonsynonymous SNV	MTDH	chr8	98657041	C	C	C	G_het	C
nonsynonymous SNV	MTG2	chr20	60775900	A	A	A	G_het	A
nonsynonymous SNV	MTMR1	chrX	149919305	G	G	A_hom	G	G
nonsynonymous SNV	MTMR2	chr11	95591718	C	A_het	C	C	C
nonsynonymous SNV	MTNR1A	chr4	187455426	G	G	G	G	A_het
nonsynonymous SNV	MTOR	chr1	11190767	C	C	C	C	T_het
nonsynonymous SNV	MTR	chr1	236992526	G	G	G	A_hom	G
nonsynonymous SNV	MTRF1L	chr6	153314094	T	T	C_het	T	T
nonsynonymous SNV	MTRF1L	chr6	153323594	C	T_hom	T_hom	C	T_het
nonsynonymous SNV	MTUS1	chr8	17612686	A	A	C_het	A	A
nonsynonymous SNV	MTUS2	chr13	29608190	T	T	C_het	T	T
nonsynonymous SNV	MUC12	chr7	100612952	G	A_het	A_het	A_het	A_het
nonsynonymous SNV	MUC12	chr7	100612961	G	C_het	C_het	C_het	C_het
nonsynonymous SNV	MUC12	chr7	100635591	C	A_hom	A_hom	A_het	C
nonsynonymous SNV	MUC12	chr7	100636383	G	G	G	A_het	G
nonsynonymous SNV	MUC12	chr7	100636408	G	G	A_hom	G	G
nonsynonymous SNV	MUC12	chr7	100645825	C	C	C	C	A_het
nonsynonymous SNV	MUC12	chr7	100646824	C	C	C	C	T_het
nonsynonymous SNV	MUC12	chr7	100648713	C	C	C	T_het	C
nonsynonymous SNV	MUC13	chr3	124632448	C	C	C	G_hom	C
nonsynonymous SNV	MUC13	chr3	124639097	C	C	T_het	C	C
nonsynonymous SNV	MUC16	chr19	8997482	C	C	C	T_het	C
nonsynonymous SNV	MUC16	chr19	8997515	A	A	A	G_het	A
nonsynonymous SNV	MUC16	chr19	8997519	C	C	C	T_het	C
nonsynonymous SNV	MUC16	chr19	8999421	G	G	G	C_het	G
nonsynonymous SNV	MUC16	chr19	8999443	T	C_het	C_het	T	C_het
nonsynonymous SNV	MUC16	chr19	8999449	G	T_het	T_het	T_het	T_het
nonsynonymous SNV	MUC16	chr19	8999478	T	C_het	C_het	C_het	C_het
nonsynonymous SNV	MUC16	chr19	8999511	G	A_het	A_het	A_het	A_het
nonsynonymous SNV	MUC16	chr19	8999512	G	G	G	C_het	G
nonsynonymous SNV	MUC16	chr19	8999518	A	A	G_het	G_het	G_het
nonsynonymous SNV	MUC16	chr19	8999530	C	C	T_het	T_het	C
nonsynonymous SNV	MUC16	chr19	8999538	T	T	C_het	C_het	C_het
nonsynonymous SNV	MUC16	chr19	8999539	T	T	C_het	C_het	C_het
nonsynonymous SNV	MUC16	chr19	8999554	C	C	C	G_het	G_het
nonsynonymous SNV	MUC16	chr19	8999560	T	T	T	C_het	T
nonsynonymous SNV	MUC16	chr19	9000169	C	T_het	T_het	T_het	C
nonsynonymous SNV	MUC16	chr19	9000187	C	T_het	T_het	T_het	C

nonsynonymous SNV	MUC16	chr19	9000205	C	C	A_het	A_het	C
nonsynonymous SNV	MUC16	chr19	9002504	T	T	C_het	C_het	C_het
nonsynonymous SNV	MUC16	chr19	9002519	G	G	C_het	C_het	C_het
nonsynonymous SNV	MUC16	chr19	9002576	T	T	T	C_het	C_het
nonsynonymous SNV	MUC16	chr19	9002587	C	C	C	C	T_het
nonsynonymous SNV	MUC16	chr19	9002597	C	C	C	C	T_het
nonsynonymous SNV	MUC16	chr19	9002612	T	T	T	T	G_het
nonsynonymous SNV	MUC16	chr19	9002623	C	C	C	C	T_het
nonsynonymous SNV	MUC16	chr19	9009614	T	C_het	T	T	T
nonsynonymous SNV	MUC16	chr19	9009637	T	T	C_het	T	T
nonsynonymous SNV	MUC16	chr19	9009640	C	C	T_het	C	C
nonsynonymous SNV	MUC16	chr19	9026247	T	T	C_het	T	T
nonsynonymous SNV	MUC16	chr19	9028242	T	T	T	T	C_het
nonsynonymous SNV	MUC16	chr19	9028253	G	G	G	G	C_het
nonsynonymous SNV	MUC16	chr19	9048137	G	A_het	G	G	G
nonsynonymous SNV	MUC16	chr19	9062443	T	T	A_het	T	T
nonsynonymous SNV	MUC16	chr19	9070873	A	A	G_het	A	A
nonsynonymous SNV	MUC16	chr19	9082961	A	A	G_het	A	A
nonsynonymous SNV	MUC17	chr7	100677279	G	G	G	C_het	G
nonsynonymous SNV	MUC17	chr7	100677333	C	C	C	G_het	C
nonsynonymous SNV	MUC17	chr7	100677645	C	C	C	A_het	C
nonsynonymous SNV	MUC17	chr7	100677704	C	C	C	T_het	C
nonsynonymous SNV	MUC17	chr7	100677714	C	C	C	T_het	C
nonsynonymous SNV	MUC17	chr7	100677816	G	G	G	A_het	G
nonsynonymous SNV	MUC17	chr7	100677867	C	C	C	C	T_hom
nonsynonymous SNV	MUC17	chr7	100677944	T	T	T	C_het	T
nonsynonymous SNV	MUC17	chr7	100677974	A	A	A	G_het	A
nonsynonymous SNV	MUC17	chr7	100677988	T	T	T	A_het	T
nonsynonymous SNV	MUC17	chr7	100678418	A	A	A	C_het	A
nonsynonymous SNV	MUC17	chr7	100678442	C	C	C	G_het	C
nonsynonymous SNV	MUC17	chr7	100678481	A	A	A	T_het	A
nonsynonymous SNV	MUC17	chr7	100678527	G	G	G	T_het	G
nonsynonymous SNV	MUC17	chr7	100678560	C	C	C	A_het	C
nonsynonymous SNV	MUC17	chr7	100678568	A	A	A	T_het	A
nonsynonymous SNV	MUC17	chr7	100678610	A	A	A	T_het	A
nonsynonymous SNV	MUC17	chr7	100678616	G	G	G	A_het	G
nonsynonymous SNV	MUC17	chr7	100678622	G	G	G	A_het	G
nonsynonymous SNV	MUC17	chr7	100678820	T	T	T	C_het	T
nonsynonymous SNV	MUC17	chr7	100679366	A	G_het	G_hom	A	G_hom
nonsynonymous SNV	MUC17	chr7	100679754	C	C	C	G_het	C
nonsynonymous SNV	MUC17	chr7	100680243	C	C	C	T_het	C
nonsynonymous SNV	MUC17	chr7	100680251	G	G	G	A_het	G
nonsynonymous SNV	MUC17	chr7	100680313	A	A	A	G_het	A
nonsynonymous SNV	MUC17	chr7	100681047	C	C	C	C	T_hom
nonsynonymous SNV	MUC17	chr7	100681211	G	C_het	G	G	G

nonsynonymous SNV	MUC17	chr7	100683036	T	T	T	C_het	T
nonsynonymous SNV	MUC17	chr7	100684400	G	G	G	A_het	G
nonsynonymous SNV	MUC2	chr11	1079672	G	G	G	A_hom	G
nonsynonymous SNV	MUC2	chr11	1092872	C	G_het	C	G_het	G_het
nonsynonymous SNV	MUC2	chr11	1092997	A	A	A	A	C_het
nonsynonymous SNV	MUC2	chr11	1092998	C	C	C	C	T_het
nonsynonymous SNV	MUC2	chr11	1093022	C	C	C	C	T_het
nonsynonymous SNV	MUC2	chr11	1093286	C	C	C	C	G_het
nonsynonymous SNV	MUC2	chr11	1093582	G	G	C_het	C_het	C_het
nonsynonymous SNV	MUC2	chr11	1093610	C	C	C	C	G_het
nonsynonymous SNV	MUC20	chr3	195447886	G	C_het	C_het	C_het	C_het
nonsynonymous SNV	MUC20	chr3	195452783	C	C	T_het	C	C
nonsynonymous SNV	MUC20	chr3	195452799	C	C	T_het	C	C
nonsynonymous SNV	MUC20	chr3	195452805	T	C_het	C_het	T	T
nonsynonymous SNV	MUC20	chr3	195452951	G	C_het	C_het	C_hom	C_hom
nonsynonymous SNV	MUC20	chr3	195452987	G	G	G	A_het	A_het
nonsynonymous SNV	MUC20	chr3	195452991	C	C	C	T_het	T_het
nonsynonymous SNV	MUC20	chr3	195453014	G	G	A_het	A_hom	A_het
nonsynonymous SNV	MUC20	chr3	195453243	C	T_het	C	T_het	T_het
nonsynonymous SNV	MUC20	chr3	195453249	T	C_het	T	T	T
nonsynonymous SNV	MUC20	chr3	195453257	G	A_het	A_het	A_het	A_het
nonsynonymous SNV	MUC20	chr3	195453288	A	A	C_het	A	A
nonsynonymous SNV	MUC20	chr3	195453434	G	A_het	G	G	G
nonsynonymous SNV	MUC20	chr3	195456561	C	C	C	G_het	G_het
nonsynonymous SNV	MUC3A	chr7	100551565	A	G_het	G_het	G_het	G_het
nonsynonymous SNV	MUC3A	chr7	100551566	T	C_het	C_het	C_het	C_het
nonsynonymous SNV	MUC3A	chr7	100551578	T	G_het	G_het	G_het	G_het
nonsynonymous SNV	MUC3A	chr7	100551646	A	G_het	G_het	G_het	G_het
nonsynonymous SNV	MUC3A	chr7	100551662	C	T_het	T_het	T_het	T_het
nonsynonymous SNV	MUC3A	chr7	100551692	C	T_het	T_het	T_het	T_het
nonsynonymous SNV	MUC3A	chr7	100551719	C	T_het	T_het	T_het	T_het
nonsynonymous SNV	MUC3A	chr7	100551773	C	A_het	A_het	A_het	A_het
nonsynonymous SNV	MUC3A	chr7	100551778	G	A_het	A_het	A_het	A_het
nonsynonymous SNV	MUC3A	chr7	100551793	G	T_het	T_het	T_het	T_het
nonsynonymous SNV	MUC3A	chr7	100551842	C	T_het	T_het	T_het	T_het
nonsynonymous SNV	MUC3A	chr7	100552018	T	G_het	G_het	G_het	G_het
nonsynonymous SNV	MUC3A	chr7	100552094	G	C_het	C_het	C_het	C_het
nonsynonymous SNV	MUC3A	chr7	100552117	G	T_het	T_het	T_het	T_het
nonsynonymous SNV	MUC3A	chr7	100552135	T	C_het	C_het	C_het	C_het
nonsynonymous SNV	MUC3A	chr7	100552358	C	G_het	G_het	G_het	G_het
nonsynonymous SNV	MUC3A	chr7	100552371	C	G_het	G_het	G_het	G_het
nonsynonymous SNV	MUC3A	chr7	100552384	A	G_het	G_het	G_het	G_het
nonsynonymous SNV	MUC3A	chr7	100552388	C	C	C	T_het	C
nonsynonymous SNV	MUC3A	chr7	100552390	T	C_het	C_het	C_het	C_het
nonsynonymous SNV	MUC3A	chr7	100552412	T	C_het	C_het	C_het	C_het

nonsynonymous SNV	MUC3A	chr7	100552435	T	A_het	A_het	A_het	A_het
nonsynonymous SNV	MUC3A	chr7	100552436	C	T_het	T_het	T_het	T_het
nonsynonymous SNV	MUC3A	chr7	100552535	G	A_het	A_het	A_het	A_het
nonsynonymous SNV	MUC3A	chr7	100552549	C	A_het	A_het	A_het	A_het
nonsynonymous SNV	MUC3A	chr7	100552577	C	C	C	T_het	C
nonsynonymous SNV	MUC3A	chr7	100552657	A	G_het	G_het	G_het	G_het
nonsynonymous SNV	MUC3A	chr7	100552675	C	T_het	T_het	T_het	T_het
nonsynonymous SNV	MUC3A	chr7	100552711	T	A_het	A_het	A_het	A_het
nonsynonymous SNV	MUC3A	chr7	100552727	G	C_het	C_het	C_het	C_het
stopgain	MUC3A	chr7	100552738	C	T_het	T_het	T_het	T_het
nonsynonymous SNV	MUC3A	chr7	100552739	A	G_het	G_het	G_het	G_het
nonsynonymous SNV	MUC3A	chr7	100607871	C	T_het	T_het	T_het	T_het
nonsynonymous SNV	MUC3A	chr7	100608370	A	C_het	C_het	C_het	C_het
nonsynonymous SNV	MUC3A	chr7	100608884	G	A_het	A_het	A_het	A_het
nonsynonymous SNV	MUC3A	chr7	100610069	C	T_het	T_het	T_het	T_het
nonsynonymous SNV	MUC4	chr3	195501103	G	G	G	G	A_het
nonsynonymous SNV	MUC4	chr3	195505843	G	G	G	A_het	G
nonsynonymous SNV	MUC4	chr3	195507107	C	C	C	T_hom	C
nonsynonymous SNV	MUC4	chr3	195507193	G	G	A_het	G	G
nonsynonymous SNV	MUC4	chr3	195507227	C	C	C	T_hom	C
nonsynonymous SNV	MUC4	chr3	195507323	T	C_het	T	T	T
nonsynonymous SNV	MUC4	chr3	195507324	G	C_het	G	G	G
nonsynonymous SNV	MUC4	chr3	195507332	A	T_het	A	A	A
nonsynonymous SNV	MUC4	chr3	195507349	A	C_het	A	A	A
nonsynonymous SNV	MUC4	chr3	195507475	G	C_het	G	G	C_het
nonsynonymous SNV	MUC4	chr3	195507494	C	T_het	C	C	T_het
nonsynonymous SNV	MUC4	chr3	195508009	G	G	A_het	G	A_het
nonsynonymous SNV	MUC4	chr3	195508010	C	C	C	C	A_het
nonsynonymous SNV	MUC4	chr3	195508228	G	G	C_het	G	G
nonsynonymous SNV	MUC4	chr3	195508475	C	C	C	T_hom	C
nonsynonymous SNV	MUC4	chr3	195508478	G	G	G	C_hom	G
nonsynonymous SNV	MUC4	chr3	195509212	G	A_het	G	G	G
nonsynonymous SNV	MUC4	chr3	195509429	A	G_het	A	A	A
nonsynonymous SNV	MUC4	chr3	195509676	C	C	C	G_het	C
nonsynonymous SNV	MUC4	chr3	195510707	T	G_het	G_het	T	G_het
nonsynonymous SNV	MUC4	chr3	195510718	G	T_het	T_het	G	T_het
nonsynonymous SNV	MUC4	chr3	195510745	G	A_het	A_het	G	A_het
nonsynonymous SNV	MUC4	chr3	195511780	G	G	A_het	G	A_het
nonsynonymous SNV	MUC4	chr3	195512004	T	T	T	G_hom	T
nonsynonymous SNV	MUC4	chr3	195512042	T	T	T	C_hom	T
nonsynonymous SNV	MUC4	chr3	195512107	T	T	T	A_hom	T
nonsynonymous SNV	MUC4	chr3	195512212	G	G	T_het	G	T_het
nonsynonymous SNV	MUC4	chr3	195512219	C	C	C	T_hom	C
nonsynonymous SNV	MUC4	chr3	195512245	T	C_het	C_hom	C_hom	C_hom
nonsynonymous SNV	MUC4	chr3	195512287	G	A_het	G	G	A_het

nonsynonymous SNV	MUC4	chr3	195512302	G	A_het	G	G	G
nonsynonymous SNV	MUC4	chr3	195513173	G	A_het	G	G	G
nonsynonymous SNV	MUC4	chr3	195513364	C	C	C	T_hom	C
nonsynonymous SNV	MUC4	chr3	195513383	T	T	T	A_hom	T
nonsynonymous SNV	MUC4	chr3	195513439	G	G	G	A_hom	G
nonsynonymous SNV	MUC4	chr3	195513598	G	G	G	A_hom	G
nonsynonymous SNV	MUC4	chr3	195513680	A	T_het	T_het	A	A
nonsynonymous SNV	MUC4	chr3	195513743	G	G	G	T_hom	G
nonsynonymous SNV	MUC4	chr3	195513779	C	T_het	T_het	C	T_het
nonsynonymous SNV	MUC4	chr3	195514324	G	A_hom	G	G	A_hom
nonsynonymous SNV	MUC4	chr3	195514379	T	C_hom	T	T	C_hom
nonsynonymous SNV	MUC4	chr3	195514393	A	G_hom	A	A	G_hom
nonsynonymous SNV	MUC4	chr3	195514403	C	T_hom	C	C	T_hom
nonsynonymous SNV	MUC4	chr3	195514470	C	C	C	G_hom	C
nonsynonymous SNV	MUC4	chr3	195514471	A	A	A	G_hom	A
nonsynonymous SNV	MUC4	chr3	195514480	C	C	C	T_hom	C
nonsynonymous SNV	MUC4	chr3	195514489	G	G	G	A_hom	G
nonsynonymous SNV	MUC4	chr3	195514510	G	G	G	A_hom	G
nonsynonymous SNV	MUC4	chr3	195514517	G	G	G	A_hom	G
nonsynonymous SNV	MUC4	chr3	195514523	C	C	C	T_hom	C
nonsynonymous SNV	MUC4	chr3	195514526	G	G	G	C_hom	G
nonsynonymous SNV	MUC4	chr3	195515017	A	A	G_het	A	G_het
nonsynonymous SNV	MUC4	chr3	195515045	A	A	G_het	A	G_het
nonsynonymous SNV	MUC4	chr3	195515051	C	C	T_het	C	T_het
nonsynonymous SNV	MUC4	chr3	195515054	G	G	C_het	G	G
nonsynonymous SNV	MUC5B	chr11	1157787	G	A_het	G	G	G
nonsynonymous SNV	MUC5B	chr11	1253976	A	G_het	G_het	G_het	G_het
nonsynonymous SNV	MUC5B	chr11	1253980	A	G_het	G_het	G_het	G_het
nonsynonymous SNV	MUC5B	chr11	1258192	A	T_het	T_het	T_het	T_het
nonsynonymous SNV	MUC5B	chr11	1258197	A	G_het	G_het	G_het	G_het
nonsynonymous SNV	MUC5B	chr11	1258240	C	T_het	T_het	T_het	T_het
nonsynonymous SNV	MUC5B	chr11	1258377	G	A_het	G	G	G
nonsynonymous SNV	MUC5B	chr11	1260217	C	C	A_het	C	C
nonsynonymous SNV	MUC5B	chr11	1260219	C	C	T_het	C	C
nonsynonymous SNV	MUC5B	chr11	1264631	A	A	A	A	G_het
nonsynonymous SNV	MUC5B	chr11	1264742	G	G	C_het	G	G
nonsynonymous SNV	MUC5B	chr11	1267960	A	G_het	G_het	G_hom	A
nonsynonymous SNV	MUC6	chr11	1016609	C	G_het	C	C	C
nonsynonymous SNV	MUC6	chr11	1016640	G	A_het	A_het	A_het	G
nonsynonymous SNV	MUC6	chr11	1016655	G	A_het	A_het	A_het	A_het
nonsynonymous SNV	MUC6	chr11	1016665	C	T_het	T_het	T_het	T_het
nonsynonymous SNV	MUC6	chr11	1016704	C	A_het	A_het	A_het	A_het
nonsynonymous SNV	MUC6	chr11	1016713	T	C_het	C_het	C_het	C_het
nonsynonymous SNV	MUC6	chr11	1016722	T	T	G_het	T	G_het
nonsynonymous SNV	MUC6	chr11	1016724	A	G_het	A	A	A

nonsynonymous SNV	MUC6	chr11	1016776	C	T_het	T_het	T_het	T_het
nonsynonymous SNV	MUC6	chr11	1016795	C	C	C	C	G_het
nonsynonymous SNV	MUC6	chr11	1016871	G	G	T_het	G	G
nonsynonymous SNV	MUC6	chr11	1016890	G	G	G	A_het	A_het
nonsynonymous SNV	MUC6	chr11	1016892	G	G	A_het	G	G
nonsynonymous SNV	MUC6	chr11	1016908	G	G	T_het	G	G
nonsynonymous SNV	MUC6	chr11	1016910	G	A_het	A_het	A_het	A_het
nonsynonymous SNV	MUC6	chr11	1016914	G	G	G	T_het	T_het
nonsynonymous SNV	MUC6	chr11	1016916	A	G_het	G_het	A	A
nonsynonymous SNV	MUC6	chr11	1016934	G	A_het	A_het	A_het	A_het
nonsynonymous SNV	MUC6	chr11	1016944	G	G	G	G	T_het
nonsynonymous SNV	MUC6	chr11	1016957	T	G_het	G_het	G_het	G_het
nonsynonymous SNV	MUC6	chr11	1016959	T	T	T	C_het	T
nonsynonymous SNV	MUC6	chr11	1016968	T	T	T	G_het	C_het
nonsynonymous SNV	MUC6	chr11	1016976	T	T	T	G_het	G_het
nonsynonymous SNV	MUC6	chr11	1016977	G	G	A_het	G	A_het
nonsynonymous SNV	MUC6	chr11	1016991	T	T	T	T	G_het
nonsynonymous SNV	MUC6	chr11	1017045	G	G	G	T_het	G
nonsynonymous SNV	MUC6	chr11	1017069	G	A_het	A_het	A_het	A_het
nonsynonymous SNV	MUC6	chr11	1017084	G	A_het	G	A_het	G
nonsynonymous SNV	MUC6	chr11	1017112	G	C_het	G	C_het	A_het
nonsynonymous SNV	MUC6	chr11	1017135	G	A_het	A_het	A_het	A_het
nonsynonymous SNV	MUC6	chr11	1017154	T	G_het	G_het	G_het	G_het
nonsynonymous SNV	MUC6	chr11	1017169	G	A_het	A_het	G	A_het
nonsynonymous SNV	MUC6	chr11	1017183	G	T_het	T_het	T_het	T_het
nonsynonymous SNV	MUC6	chr11	1017186	A	G_het	G_het	A	G_het
nonsynonymous SNV	MUC6	chr11	1017220	T	C_het	C_het	C_het	C_het
nonsynonymous SNV	MUC6	chr11	1017231	A	G_het	G_het	G_het	G_het
nonsynonymous SNV	MUC6	chr11	1017280	G	T_het	T_het	G	T_het
nonsynonymous SNV	MUC6	chr11	1017294	A	T_het	T_het	A	T_het
nonsynonymous SNV	MUC6	chr11	1017302	G	C_het	C_het	C_het	C_het
nonsynonymous SNV	MUC6	chr11	1017325	A	C_het	C_het	C_het	C_het
nonsynonymous SNV	MUC6	chr11	1017337	T	C_het	C_het	C_het	C_het
nonsynonymous SNV	MUC6	chr11	1017338	C	A_het	A_het	A_het	A_het
nonsynonymous SNV	MUC6	chr11	1017381	G	C_het	G	G	C_het
nonsynonymous SNV	MUC6	chr11	1017384	G	C_het	C_het	C_het	C_het
nonsynonymous SNV	MUC6	chr11	1017421	G	T_het	T_het	T_het	T_het
stopgain	MUC6	chr11	1017466	T	C_het	A_het	C_het	T
nonsynonymous SNV	MUC6	chr11	1017483	T	G_het	T	T	T
nonsynonymous SNV	MUC6	chr11	1017498	C	G_het	G_het	G_het	G_het
nonsynonymous SNV	MUC6	chr11	1017514	A	G_het	G_het	A	A
nonsynonymous SNV	MUC6	chr11	1017529	G	A_het	A_het	G	G
nonsynonymous SNV	MUC6	chr11	1017746	T	G_het	G_het	G_het	G_het
nonsynonymous SNV	MUC6	chr11	1017789	A	C_het	C_het	A	A
nonsynonymous SNV	MUC6	chr11	1017844	T	T	C_het	C_het	C_het

nonsynonymous SNV	MUC6	chr11	1017858	A	G_het	G_het	G_het	G_het
nonsynonymous SNV	MUC6	chr11	1017883	C	C	C	T_het	C
nonsynonymous SNV	MUC6	chr11	1017900	G	T_het	G	T_het	T_het
nonsynonymous SNV	MUC6	chr11	1017921	C	T_het	C	T_het	A_het
nonsynonymous SNV	MUC6	chr11	1017933	A	G_het	G_het	A	G_het
nonsynonymous SNV	MUC6	chr11	1017939	G	G	C_het	G	C_het
nonsynonymous SNV	MUC6	chr11	1017963	G	A_het	A_het	A_het	A_het
nonsynonymous SNV	MUC6	chr11	1017981	T	A_het	A_het	A_het	A_het
nonsynonymous SNV	MUC6	chr11	1017988	G	T_het	T_het	T_het	T_het
nonsynonymous SNV	MUC6	chr11	1018024	C	G_het	G_het	G_het	G_het
nonsynonymous SNV	MUC6	chr11	1018042	A	G_het	G_het	G_het	G_het
nonsynonymous SNV	MUC6	chr11	1018048	A	G_het	G_het	G_het	G_het
nonsynonymous SNV	MUC6	chr11	1018059	C	T_het	T_het	T_het	T_het
nonsynonymous SNV	MUC6	chr11	1018116	T	G_het	G_het	G_het	G_het
nonsynonymous SNV	MUC6	chr11	1018138	T	C_het	C_het	C_het	C_het
nonsynonymous SNV	MUC6	chr11	1018144	G	C_het	C_het	C_het	C_het
nonsynonymous SNV	MUC6	chr11	1018182	G	T_het	T_het	T_het	T_het
nonsynonymous SNV	MUC6	chr11	1018186	G	T_het	T_het	T_het	T_het
nonsynonymous SNV	MUC6	chr11	1018192	C	T_het	T_het	T_het	T_het
nonsynonymous SNV	MUC6	chr11	1018218	T	G_het	T	T	G_het
nonsynonymous SNV	MUC6	chr11	1018237	G	A_het	G	A_het	A_het
nonsynonymous SNV	MUC6	chr11	1018245	T	G_het	T	G_het	G_het
nonsynonymous SNV	MUC6	chr11	1018246	T	C_het	T	C_het	C_het
nonsynonymous SNV	MUC6	chr11	1018248	G	A_het	G	G	G
nonsynonymous SNV	MUC6	chr11	1018262	G	G	G	C_het	C_het
nonsynonymous SNV	MUC6	chr11	1018263	T	T	T	G_het	G_het
nonsynonymous SNV	MUC6	chr11	1018266	G	A_het	G	G	A_het
nonsynonymous SNV	MUC6	chr11	1018272	C	G_het	C	C	C
nonsynonymous SNV	MUC6	chr11	1018279	C	G_het	C	C	T_het
nonsynonymous SNV	MUC6	chr11	1018281	C	A_het	C	C	C
nonsynonymous SNV	MUC6	chr11	1018290	G	C_het	G	G	A_het
nonsynonymous SNV	MUC6	chr11	1018291	G	C_het	C_het	G	C_het
nonsynonymous SNV	MUC6	chr11	1018295	T	C_het	C_het	C_het	C_het
nonsynonymous SNV	MUC6	chr11	1018303	G	A_het	G	G	G
nonsynonymous SNV	MUC6	chr11	1018329	T	A_het	A_het	T	T
nonsynonymous SNV	MUC6	chr11	1018341	G	A_het	G	G	G
nonsynonymous SNV	MUC6	chr11	1018366	C	T_het	C	T_het	T_het
nonsynonymous SNV	MUM1	chr19	1360408	C	T_het	C	C	C
nonsynonymous SNV	MUM1	chr19	1360981	C	T_het	C	C	C
nonsynonymous SNV	MUSK	chr9	113562649	A	A	A	A	G_hom
nonsynonymous SNV	MUT	chr6	49399514	C	C	C	T_hom	C
nonsynonymous SNV	MUT	chr6	49409598	C	C	T_het	C	C
stopgain	MVP	chr16	29858641	C	C	T_het	C	C
nonsynonymous SNV	MYBBP1A	chr17	4455266	G	T_het	G	G	G
nonsynonymous SNV	MYBPC2	chr19	50949163	G	G	A_het	G	G

nonsynonymous SNV	MYBPC3	chr11	47369442	C	C	C	C	T_het
nonsynonymous SNV	MYC	chr8	128750527	T	T	C_het	T	T
nonsynonymous SNV	MYCBP	chr1	39338974	C	C	A_het	C	C
nonsynonymous SNV	MYCN	chr2	16082317	C	T_het	C	C	C
nonsynonymous SNV	MYF5	chr12	81111113	C	C	T_het	C	C
nonsynonymous SNV	MYF5	chr12	81111273	A	T_het	A	A	A
nonsynonymous SNV	MYH10	chr17	8409703	C	C	A_het	C	C
nonsynonymous SNV	MYH11	chr16	15835455	C	C	C	A_het	C
nonsynonymous SNV	MYH14	chr19	50730215	T	T	C_het	T	T
nonsynonymous SNV	MYH14	chr19	50747534	G	G	G	T_het	G
nonsynonymous SNV	MYH15	chr3	108117612	G	G	A_het	G	G
nonsynonymous SNV	MYH8	chr17	10299899	G	G	G	A_hom	G
nonsynonymous SNV	MYH8	chr17	10310061	C	C	T_hom	C	C
nonsynonymous SNV	MYH9	chr22	36693051	C	C	T_het	C	C
nonsynonymous SNV	MYL10	chr7	101267298	G	G	C_het	C_het	G
nonsynonymous SNV	MYO10	chr5	16672915	C	C	T_het	C	C
nonsynonymous SNV	MYO10	chr5	16694499	C	C	C	C	T_het
nonsynonymous SNV	MYO15A	chr17	18024975	C	C	C	C	T_het
nonsynonymous SNV	MYO15B	chr17	73587756	G	G	G	T_hom	G
nonsynonymous SNV	MYO18B	chr22	26423570	G	G	G	G	A_hom
nonsynonymous SNV	MYO1A	chr12	57422576	T	T	T	G_het	T
nonsynonymous SNV	MYO1A	chr12	57431816	A	A	A	A	G_het
stopgain	MYO1B	chr2	192228934	C	C	T_het	C	C
nonsynonymous SNV	MYO1D	chr17	31094761	T	T	T	T	G_hom
nonsynonymous SNV	MYO3A	chr10	26442827	G	G	G	G	A_het
nonsynonymous SNV	MYO5C	chr15	52504009	C	C	C	C	T_hom
nonsynonymous SNV	MYO7B	chr2	128364817	C	C	C	T_het	C
nonsynonymous SNV	MYO7B	chr2	128380826	G	G	G	A_het	G
nonsynonymous SNV	MYO7B	chr2	128387373	C	C	C	A_het	C
nonsynonymous SNV	MYO9B	chr19	17212694	G	G	G	A_het	G
nonsynonymous SNV	MYO9B	chr19	17322782	G	G	A_het	G	G
nonsynonymous SNV	MYOCD	chr17	12655938	G	G	G	A_hom	G
stopgain	MYOF	chr10	95139728	G	G	G	G	T_het
nonsynonymous SNV	MYOG	chr1	203054984	C	G_het	C	C	C
nonsynonymous SNV	MYOM2	chr8	2041807	C	C	C	T_het	C
nonsynonymous SNV	MYOM3	chr1	24390589	C	C	T_het	C	C
nonsynonymous SNV	MYOT	chr5	137206560	A	C_hom	C_hom	C_hom	C_hom
nonsynonymous SNV	MYRF	chr11	61548796	G	G	A_het	G	G
nonsynonymous SNV	MZF1	chr19	59082734	G	G	A_het	G	G
nonsynonymous SNV	NAA25	chr12	112478316	G	G	A_het	G	G
nonsynonymous SNV	NAA35	chr9	88633656	C	C	C	T_hom	C
nonsynonymous SNV	NAAA	chr4	76857354	C	C	T_het	C	C
nonsynonymous SNV	NAALADL2	chr3	175189480	T	T	T	G_hom	T
nonsynonymous SNV	NACC2	chr9	138903733	T	T	C_het	T	T
nonsynonymous SNV	NADK	chr1	1691199	C	C	C	T_hom	C

nonsynonymous SNV	NADSYN1	chr11	71189477	C	T_het	C	C	C
nonsynonymous SNV	NANOG	chr12	7945640	G	T_hom	T_het	T_het	G
stopgain	NANOGNB	chr12	7926427	C	C	T_het	C	C
nonsynonymous SNV	NANS	chr9	100839202	G	A_hom	G	G	G
nonsynonymous SNV	NARFL	chr16	782615	C	C	C	C	A_het
nonsynonymous SNV	NARG2	chr15	60741467	C	C	C	T_het	C
nonsynonymous SNV	NAV1	chr1	201763369	T	G_het	T	T	T
nonsynonymous SNV	NAV2	chr11	19955076	G	G	G	G	A_het
nonsynonymous SNV	NAV3	chr12	78512060	G	G	G	A_het	G
nonsynonymous SNV	NBEA	chr13	35770154	G	G	G	A_het	G
nonsynonymous SNV	NBEA	chr13	36229027	G	G	C_het	G	G
stopgain	NBEA	chr13	36241642	C	C	T_het	C	C
nonsynonymous SNV	NBEAL1	chr2	203972496	A	A	A	G_het	A
nonsynonymous SNV	NBEAL1	chr2	204073435	G	G	A_het	G	G
nonsynonymous SNV	NBEAP1	chr15	20874863	T	C_het	C_het	C_het	T
nonsynonymous SNV	NBEAP1	chr15	20874897	A	G_hom	G_het	G_het	G_het
nonsynonymous SNV	NBEAP1	chr15	20874927	T	A_het	A_het	T	T
nonsynonymous SNV	NBEAP1	chr15	20876515	A	G_hom	G_het	G_het	G_het
nonsynonymous SNV	NBN	chr8	90990521	T	T	C_het	T	T
nonsynonymous SNV	NBPF1	chr1	16890598	T	A_het	A_het	A_het	A_het
nonsynonymous SNV	NBPF1	chr1	16890602	T	T	G_het	T	T
nonsynonymous SNV	NBPF1	chr1	16890644	C	T_het	T_het	T_het	T_het
nonsynonymous SNV	NBPF1	chr1	16891365	G	A_het	G	A_het	A_het
nonsynonymous SNV	NBPF1	chr1	16893841	C	C	C	T_het	C
nonsynonymous SNV	NBPF1	chr1	16895634	T	T	G_het	T	T
nonsynonymous SNV	NBPF1	chr1	16901668	T	C_het	C_het	C_het	C_het
nonsynonymous SNV	NBPF1	chr1	16902884	T	T	C_het	T	T
nonsynonymous SNV	NBPF1	chr1	16903912	T	A_hom	T	T	T
nonsynonymous SNV	NBPF1	chr1	16918424	G	C_het	G	G	G
nonsynonymous SNV	NBPF1	chr1	16918457	G	G	C_het	G	C_het
nonsynonymous SNV	NBPF1	chr1	16918473	G	G	G	A_het	A_het
nonsynonymous SNV	NBPF10	chr1	145293425	C	C	C	C	A_het
nonsynonymous SNV	NBPF10	chr1	145293428	G	G	G	G	C_het
nonsynonymous SNV	NBPF10	chr1	145293490	G	G	G	G	C_het
nonsynonymous SNV	NBPF10	chr1	145293498	G	G	G	G	C_het
nonsynonymous SNV	NBPF10	chr1	145293510	C	C	C	C	G_het
nonsynonymous SNV	NBPF10	chr1	145293515	A	A	G_het	G_het	A
nonsynonymous SNV	NBPF10	chr1	145293566	G	A_het	A_het	A_het	A_het
nonsynonymous SNV	NBPF10	chr1	145296478	G	T_het	G	T_het	T_het
nonsynonymous SNV	NBPF10	chr1	145311110	G	C_het	C_het	G	G
nonsynonymous SNV	NBPF10	chr1	145311854	C	C	C	C	T_het
nonsynonymous SNV	NBPF10	chr1	145311931	C	T_het	C	C	C
nonsynonymous SNV	NBPF10	chr1	145323656	A	A	A	A	T_het
nonsynonymous SNV	NBPF10	chr1	145367719	G	G	G	G	A_het
nonsynonymous SNV	NBPF10	chr1	145367767	G	G	G	G	A_het

nonsynonymous SNV	NBPF10	chr1	145367777	G	G	G	G	T_het
nonsynonymous SNV	NBPF14	chr1	148252111	C	T_het	T_het	T_het	T_het
nonsynonymous SNV	NBPF14	chr1	148342488	T	C_het	T	T	T
nonsynonymous SNV	NBPF14	chr1	148344741	G	C_het	G	G	C_het
nonsynonymous SNV	NBPF22P	chr5	85586831	C	C	C	C	T_het
nonsynonymous SNV	NBPF3	chr1	21806667	C	C	G_hom	C	C
nonsynonymous SNV	NBPF3	chr1	21806710	T	T	G_hom	T	T
nonsynonymous SNV	NBPF4	chr1	108771721	T	T	C_het	T	C_het
nonsynonymous SNV	NBPF6	chr1	109007849	G	G	A_hom	G	G
nonsynonymous SNV	NBPF7	chr1	120384066	C	C	C	T_hom	C
stopgain	NBPF8	chr1	144828764	G	T_het	T_het	T_het	T_het
nonsynonymous SNV	NBPF8	chr1	144828765	A	T_het	T_het	T_het	T_het
nonsynonymous SNV	NBPF9	chr1	144618201	T	T	T	T	C_het
nonsynonymous SNV	NBPF9	chr1	145368461	A	A	C_het	C_het	A
nonsynonymous SNV	NBPF9	chr1	145368473	G	G	C_het	G	G
nonsynonymous SNV	NBPF9	chr1	145368518	C	C	C	T_het	C
nonsynonymous SNV	NBR1	chr17	41338439	C	A_het	C	C	C
stopgain	NBR1	chr17	41349060	C	C	T_het	C	C
stopgain	NCBP1	chr9	100431191	C	C	T_het	C	C
nonsynonymous SNV	NCF1C	chr7	74578584	G	G	G	A_het	G
nonsynonymous SNV	NCKAP1L	chr12	54920454	C	C	T_het	C	C
nonsynonymous SNV	NCOA2	chr8	71036271	T	T	C_het	T	T
nonsynonymous SNV	NCOA2	chr8	71069128	C	C	T_het	C	C
nonsynonymous SNV	NCOR1	chr17	16068340	C	T_het	T_het	C	C
stopgain	NCOR1	chr17	16068343	G	A_het	A_het	G	G
nonsynonymous SNV	NCOR1	chr17	16068377	C	G_het	G_het	G_het	G_het
nonsynonymous SNV	NCOR1	chr17	16068396	G	A_het	A_het	A_het	A_het
nonsynonymous SNV	NCOR1	chr17	16068463	C	C	C	C	T_het
nonsynonymous SNV	NDC80	chr18	2616503	C	C	C	T_hom	C
nonsynonymous SNV	NDE1	chr16	15788103	G	G	A_het	G	G
nonsynonymous SNV	NDNF	chr4	121957688	T	T	T	T	C_het
nonsynonymous SNV	NDST4	chr4	115997346	T	C_het	T	T	T
nonsynonymous SNV	NDUFA6	chr22	42486765	C	C	C	T_hom	C
nonsynonymous SNV	NDUFB8	chr10	102286179	C	C	C	C	T_het
nonsynonymous SNV	NDUFS7	chr19	1390985	G	G	A_het	G	G
nonsynonymous SNV	NEB	chr2	152364589	G	G	A_het	G	G
nonsynonymous SNV	NEB	chr2	152390790	T	T	T	C_het	T
nonsynonymous SNV	NEB	chr2	152448640	T	C_het	T	T	T
nonsynonymous SNV	NEB	chr2	152534466	G	G	G	G	T_het
nonsynonymous SNV	NEBL	chr10	21117502	G	G	A_het	G	G
stopgain	NEBL	chr10	21177128	G	G	C_het	G	G
nonsynonymous SNV	NEDD9	chr6	11191047	G	G	A_het	G	G
nonsynonymous SNV	NEFM	chr8	24774791	G	G	G	A_het	G
stopgain	NEIL1	chr15	75647144	C	C	T_het	C	C
nonsynonymous SNV	NEK11	chr3	130992403	T	T	T	A_hom	T

nonsynonymous SNV	NEK7	chr1	198222215	C	G_het	C	C	C
nonsynonymous SNV	NEK8	chr17	27061955	G	T_het	G	G	G
nonsynonymous SNV	NEMF	chr14	50295523	T	T	T	C_het	T
nonsynonymous SNV	NEURL	chr10	105331435	G	G	A_het	G	G
nonsynonymous SNV	NEURL4	chr17	7226183	C	C	C	C	A_het
nonsynonymous SNV	NEUROG2	chr4	113436549	G	G	G	G	A_het
nonsynonymous SNV	NFAM1	chr22	42793953	G	G	G	A_hom	G
stopgain	NFAM1	chr22	42805542	G	G	A_het	G	G
nonsynonymous SNV	NFATC1	chr18	77246538	G	A_het	G	G	G
nonsynonymous SNV	NFATC4	chr14	24842498	C	C	T_het	C	C
nonsynonymous SNV	NFE2L3	chr7	26224637	C	G_het	C	C	C
nonsynonymous SNV	NFS1	chr20	34278459	T	C_hom	T	T	T
nonsynonymous SNV	NFX1	chr9	33347727	G	G	A_het	G	G
nonsynonymous SNV	NHLRC2	chr10	115664687	G	G	A_het	G	G
nonsynonymous SNV	NHP2	chr5	177577923	C	C	T_het	C	C
nonsynonymous SNV	NHSL1	chr6	138751629	G	C_hom	G	G	G
nonsynonymous SNV	NHSL2	chrX	71359116	G	G	A_hom	G	G
nonsynonymous SNV	NIN	chr14	51211020	C	C	T_het	C	C
nonsynonymous SNV	NINL	chr20	25457375	G	G	A_het	G	G
stopgain	NKAIN4	chr20	61878743	G	G	A_het	G	G
nonsynonymous SNV	NKAPL	chr6	28227273	C	C	C	T_hom	C
nonsynonymous SNV	NKPD1	chr19	45659155	G	G	C_het	G	G
nonsynonymous SNV	NKX2-5	chr5	172661872	T	T	T	T	A_het
nonsynonymous SNV	NKX2-8	chr14	37050154	C	C	C	G_het	C
nonsynonymous SNV	NLGN3	chrX	70367951	A	A	G_hom	A	A
nonsynonymous SNV	NLRC3	chr16	3613160	G	G	A_het	G	G
nonsynonymous SNV	NLRC3	chr16	3613784	C	C	T_het	C	C
nonsynonymous SNV	NLRP13	chr19	56435974	C	C	C	T_het	C
nonsynonymous SNV	NLRP2	chr19	55481455	C	C	C	G_het	C
nonsynonymous SNV	NLRP2	chr19	55494859	C	C	T_het	C	C
nonsynonymous SNV	NLRP8	chr19	56463924	C	C	C	C	A_het
nonsynonymous SNV	NLRP8	chr19	56466133	C	C	T_het	C	C
nonsynonymous SNV	NLRX1	chr11	119044787	C	T_het	C	C	C
nonsynonymous SNV	NMT2	chr10	15175336	T	T	C_het	T	T
nonsynonymous SNV	NNT	chr5	43649341	T	T	C_het	T	T
nonsynonymous SNV	NOD1	chr7	30491528	G	A_het	G	G	G
nonsynonymous SNV	NOD2	chr16	50744877	A	A	A	G_het	A
nonsynonymous SNV	NOD2	chr16	50756540	G	G	C_het	G	G
nonsynonymous SNV	NOG	chr17	54671712	T	G_het	T	T	T
nonsynonymous SNV	NOL6	chr9	33467729	C	T_hom	C	C	C
nonsynonymous SNV	NOL7	chr6	13620523	A	A	A	G_hom	A
nonsynonymous SNV	NOL9	chr1	6614324	G	G	G	A_hom	G
nonsynonymous SNV	NOMO1	chr16	14989420	C	T_het	T_het	T_het	C
nonsynonymous SNV	NOP2	chr12	6669665	T	T	C_het	T	T
nonsynonymous SNV	NOS1	chr12	117672429	G	G	G	A_het	G

nonsynonymous SNV	NOS1	chr12	117693826	G	G	A_het	G	G
nonsynonymous SNV	NOS2	chr17	26093578	A	A	G_het	A	A
nonsynonymous SNV	NOSIP	chr19	50062213	G	G	A_het	G	G
nonsynonymous SNV	NOTCH1	chr9	139396468	C	C	A_het	C	C
nonsynonymous SNV	NOTCH1	chr9	139396913	G	G	A_het	G	G
nonsynonymous SNV	NOTCH2	chr1	120548095	C	A_het	C	C	C
nonsynonymous SNV	NOTCH2	chr1	120611964	G	C_het	G	G	C_het
nonsynonymous SNV	NOTCH2NL	chr1	145281408	C	C	C	C	T_het
nonsynonymous SNV	NOTCH2NL	chr1	145281543	C	T_het	T_het	T_het	T_het
nonsynonymous SNV	NOTCH2NL	chr1	145281613	C	A_het	C	C	A_het
nonsynonymous SNV	NOTCH2NL	chr1	145281633	C	A_het	C	C	A_het
nonsynonymous SNV	NOTCH2NL	chr1	145281656	A	T_het	A	A	A
nonsynonymous SNV	NOTCH3	chr19	15299051	G	A_het	G	G	G
nonsynonymous SNV	NOX3	chr6	155776223	G	G	A_het	G	G
nonsynonymous SNV	NPAP1	chr15	24921145	C	C	T_het	C	C
nonsynonymous SNV	NPEPL1	chr20	57290347	C	G_hom	G_het	G_het	G_hom
nonsynonymous SNV	NPEPPS	chr17	45669359	T	T	T	T	G_het
nonsynonymous SNV	NPFFR2	chr4	72897770	G	G	G	A_hom	G
nonsynonymous SNV	NPFFR2	chr4	72897807	G	G	G	G	T_het
nonsynonymous SNV	NPFFR2	chr4	73013366	C	C	C	T_hom	C
nonsynonymous SNV	NPFFR2	chr4	73013389	A	A	A	T_hom	A
nonsynonymous SNV	NPHS1	chr19	36339044	C	C	C	C	T_het
nonsynonymous SNV	NPHS1	chr19	36339212	A	C_het	A	A	A
nonsynonymous SNV	NPIPL2	chr16	74425314	G	G	A_het	G	G
nonsynonymous SNV	NPIPL2	chr16	74425548	A	A	G_het	G_het	G_hom
nonsynonymous SNV	NPIPL2	chr16	74425569	C	C	T_het	T_het	T_het
nonsynonymous SNV	NPIPL2	chr16	74425703	G	G	A_het	G	A_het
nonsynonymous SNV	NPIPL2	chr16	74425732	G	G	T_het	G	T_het
nonsynonymous SNV	NPIPL2	chr16	74425769	C	T_het	T_het	C	T_het
stopgain	NPIPL2	chr16	74425787	G	T_het	T_het	G	T_het
nonsynonymous SNV	NPIPL2	chr16	74425793	T	C_het	C_het	T	C_het
nonsynonymous SNV	NPIPL2	chr16	74425826	G	G	G	G	A_het
nonsynonymous SNV	NPL	chr1	182787717	C	C	C	C	G_het
nonsynonymous SNV	NPLOC4	chr17	79556035	C	C	T_het	C	C
nonsynonymous SNV	NPNT	chr4	106888426	T	T	T	C_hom	T
nonsynonymous SNV	NPR2	chr9	35808663	G	G	A_het	G	G
nonsynonymous SNV	NPY2R	chr4	156135501	C	G_het	C	C	C
nonsynonymous SNV	NPY4R	chr10	47087501	C	T_het	C	T_het	T_het
nonsynonymous SNV	NR0B1	chrX	30327102	C	C	C	C	T_hom
nonsynonymous SNV	NR1H2	chr19	50881866	C	C	T_het	C	C
nonsynonymous SNV	NR2C2	chr3	15073949	C	C	T_hom	C	C
nonsynonymous SNV	NR2F1	chr5	92923827	C	C	T_het	C	C
nonsynonymous SNV	NR4A2	chr2	157183338	C	C	C	C	T_het
nonsynonymous SNV	NR5A2	chr1	200017731	G	G	A_het	G	G
nonsynonymous SNV	NR5A2	chr1	200017888	A	A	A	A	T_het

nonsynonymous SNV	NRAP	chr10	115377257	G	G	G	G	A_het
nonsynonymous SNV	NRAS	chr1	115256529	T	T	T	C_hom	T
nonsynonymous SNV	NRBP2	chr8	144919852	G	G	A_het	G	G
nonsynonymous SNV	NRD1	chr1	52289381	G	G	A_het	G	G
stopgain	NRIP2	chr12	2936811	G	G	A_het	G	G
nonsynonymous SNV	NRK	chrX	105152955	G	T_het	G	G	G
nonsynonymous SNV	NRXN2	chr11	64417958	G	A_het	G	G	G
nonsynonymous SNV	NRXN3	chr14	79181449	G	G	A_het	G	G
nonsynonymous SNV	NSA2	chr5	74064783	C	C	C	C	T_het
nonsynonymous SNV	NSD1	chr5	176638468	G	G	T_het	G	G
stopgain	NSUN3	chr3	93802978	G	G	G	G	A_het
nonsynonymous SNV	NSUN5P2	chr7	72419954	A	A	C_het	A	A
nonsynonymous SNV	NSUN5P2	chr7	72419980	G	G	A_het	G	G
nonsynonymous SNV	NT5C	chr17	73126605	C	C	T_het	C	C
nonsynonymous SNV	NT5E	chr6	86159955	A	A	G_het	A	A
nonsynonymous SNV	NTF4	chr19	49565052	C	C	C	T_het	C
nonsynonymous SNV	NTHL1	chr16	2094713	G	G	A_het	G	G
nonsynonymous SNV	NTN3	chr16	2523469	G	G	A_het	G	G
nonsynonymous SNV	NTN5	chr19	49165157	A	C_het	A	A	A
nonsynonymous SNV	NTRK2	chr9	87359974	C	C	T_het	C	C
nonsynonymous SNV	NUDCD2	chr5	162884073	C	C	T_het	C	C
nonsynonymous SNV	NUFIP1	chr13	45563535	C	C	C	T_het	C
nonsynonymous SNV	NUGGC	chr8	27925128	C	C	T_het	C	C
nonsynonymous SNV	NUP133	chr1	229613430	A	A	A	A	G_het
nonsynonymous SNV	NUP50	chr22	45580364	C	C	C	C	T_hom
nonsynonymous SNV	NUP62CL	chrX	106396447	C	C	C	C	G_hom
nonsynonymous SNV	NUPL1	chr13	25914213	G	G	A_het	G	G
nonsynonymous SNV	NUSAP1	chr15	41663828	G	G	G	A_het	G
nonsynonymous SNV	NUTM2A	chr10	88988115	G	G	G	G	A_het
nonsynonymous SNV	NUTM2A	chr10	88988232	G	G	G	A_het	G
nonsynonymous SNV	NWD1	chr19	16860076	T	T	C_het	T	T
nonsynonymous SNV	NXPE1	chr11	114401284	G	G	G	A_hom	G
nonsynonymous SNV	NXPE1	chr11	114421857	T	C_het	C_het	C_het	C_het
nonsynonymous SNV	NXPE1	chr11	114421895	G	A_het	A_het	A_het	A_het
nonsynonymous SNV	NXPE3	chr3	101540638	C	C	T_het	C	C
nonsynonymous SNV	OAS1	chr12	113357307	G	G	G	A_het	G
nonsynonymous SNV	OAS3	chr12	113405328	G	G	A_het	G	G
nonsynonymous SNV	OASL	chr12	121458854	C	C	C	C	T_het
nonsynonymous SNV	OBSCN	chr1	228399980	G	G	A_het	G	G
nonsynonymous SNV	OBSCN	chr1	228404887	A	A	T_het	A	A
nonsynonymous SNV	OBSCN	chr1	228476484	G	G	G	A_hom	G
nonsynonymous SNV	OBSCN	chr1	228480317	C	C	C	T_hom	C
nonsynonymous SNV	OBSCN	chr1	228509699	G	G	A_het	G	G
nonsynonymous SNV	OBSCN	chr1	228527749	C	C	C	T_hom	C
nonsynonymous SNV	OBSCN	chr1	228529220	G	G	A_het	G	G

nonsynonymous SNV	OBSL1	chr2	220422583	G	A_het	G	G	G
nonsynonymous SNV	OBSL1	chr2	220422686	C	C	C	T_het	C
nonsynonymous SNV	OBSL1	chr2	220422934	C	C	C	C	G_het
nonsynonymous SNV	OGDHL	chr10	50953963	G	G	G	G	A_het
nonsynonymous SNV	OGFOD1	chr16	56487213	G	G	G	G	A_het
nonsynonymous SNV	OGFOD3	chr17	80352300	C	C	T_het	C	C
nonsynonymous SNV	OGT	chrX	70767673	A	A	C_het	A	C_het
nonsynonymous SNV	OIP5	chr15	41624591	C	C	C	T_het	C
nonsynonymous SNV	OLAH	chr10	15113876	G	C_het	G	G	G
nonsynonymous SNV	OMA1	chr1	58939588	G	G	G	G	A_het
nonsynonymous SNV	ONECUT1	chr15	53081859	G	C_hom	G	G	G
nonsynonymous SNV	ONECUT3	chr19	1754242	C	C	T_het	C	C
nonsynonymous SNV	OPALIN	chr10	98105759	G	G	A_het	G	G
nonsynonymous SNV	OPLAH	chr8	145112398	G	A_het	G	G	G
nonsynonymous SNV	OPN1LW	chrX	153418514	G	G	G	C_hom	G
nonsynonymous SNV	OPTC	chr1	203472838	G	A_het	G	G	G
nonsynonymous SNV	OR10A4	chr11	6898196	C	C	A_het	C	C
nonsynonymous SNV	OR10A5	chr11	6867067	G	G	C_het	G	G
nonsynonymous SNV	OR10G2	chr14	22102293	C	C	T_het	C	T_hom
nonsynonymous SNV	OR10G4	chr11	123886352	T	C_het	T	T	T
nonsynonymous SNV	OR10G4	chr11	123886681	A	G_het	G_hom	A	G_hom
nonsynonymous SNV	OR10K2	chr1	158390611	C	C	T_het	C	C
nonsynonymous SNV	OR11H2	chr14	20181502	C	T_het	T_het	C	T_het
nonsynonymous SNV	OR13C2	chr9	107367431	A	A	A	T_hom	A
nonsynonymous SNV	OR13C2	chr9	107367674	G	G	G	G	A_hom
nonsynonymous SNV	OR13C2	chr9	107367856	G	G	G	G	A_hom
nonsynonymous SNV	OR13C3	chr9	107298410	T	T	T	C_hom	T
stopgain	OR13C4	chr9	107288998	G	G	A_het	G	G
nonsynonymous SNV	OR13C5	chr9	107361460	G	A_hom	G	G	A_hom
nonsynonymous SNV	OR13D1	chr9	107457210	G	G	G	A_hom	G
nonsynonymous SNV	OR1J1	chr9	125239932	C	C	T_het	C	C
nonsynonymous SNV	OR1L3	chr9	125438019	T	T	T	A_hom	T
nonsynonymous SNV	OR1S1	chr11	57982763	A	A	A	A	G_hom
nonsynonymous SNV	OR2C1	chr16	3406875	G	G	G	A_het	G
nonsynonymous SNV	OR2G2	chr1	247751881	C	C	G_het	C	C
nonsynonymous SNV	OR2L3	chr1	248224569	G	T_het	G	T_het	G
nonsynonymous SNV	OR2L8	chr1	248112554	T	C_het	T	T	T
nonsynonymous SNV	OR2M5	chr1	248308934	C	T_hom	C	C	C
nonsynonymous SNV	OR2T12	chr1	248458676	T	C_hom	C_hom	C_hom	C_het
nonsynonymous SNV	OR2T12	chr1	248458717	C	C	G_het	G_hom	G_het
nonsynonymous SNV	OR2T2	chr1	248616408	C	T_het	T_het	T_het	T_het
nonsynonymous SNV	OR2T29	chr1	248722777	T	T	A_het	T	A_het
nonsynonymous SNV	OR2T3	chr1	248637367	A	A	G_het	A	A
nonsynonymous SNV	OR2T3	chr1	248637391	C	C	T_het	C	C
nonsynonymous SNV	OR2T3	chr1	248637480	A	A	A	G_het	G_het

nonsynonymous SNV	OR2T3	chr1	248637543	C	T_het	C	C	C
nonsynonymous SNV	OR2T33	chr1	248436996	A	A	C_het	A	A
nonsynonymous SNV	OR2T33	chr1	248437002	C	C	T_het	C	C
nonsynonymous SNV	OR2T34	chr1	248737319	G	G	A_het	A_het	A_het
nonsynonymous SNV	OR2T34	chr1	248737343	C	T_hom	C	C	C
nonsynonymous SNV	OR2T35	chr1	248801592	C	C	C	T_het	C
nonsynonymous SNV	OR2T35	chr1	248801610	G	G	G	A_het	G
nonsynonymous SNV	OR2T35	chr1	248801611	C	C	C	T_het	C
nonsynonymous SNV	OR2T4	chr1	248525100	G	G	A_het	G	A_het
nonsynonymous SNV	OR2T4	chr1	248525193	C	C	T_het	C	C
nonsynonymous SNV	OR4A16	chr11	55110740	A	C_het	A	A	A
nonsynonymous SNV	OR4A16	chr11	55110746	A	G_het	A	A	A
nonsynonymous SNV	OR4A16	chr11	55110761	T	G_het	T	T	T
nonsynonymous SNV	OR4A16	chr11	55110804	T	C_het	T	T	T
nonsynonymous SNV	OR4A16	chr11	55110806	T	G_het	T	T	T
nonsynonymous SNV	OR4A16	chr11	55110843	T	C_het	T	T	T
nonsynonymous SNV	OR4A16	chr11	55110845	A	G_het	A	A	A
nonsynonymous SNV	OR4A16	chr11	55110861	C	A_het	C	C	C
nonsynonymous SNV	OR4A16	chr11	55110877	G	A_het	G	G	G
stopgain	OR4A16	chr11	55110903	C	A_het	C	C	C
nonsynonymous SNV	OR4A16	chr11	55110918	T	C_het	T	T	T
nonsynonymous SNV	OR4A16	chr11	55110930	G	T_het	G	G	G
nonsynonymous SNV	OR4A16	chr11	55110934	T	A_het	T	T	T
nonsynonymous SNV	OR4A16	chr11	55110941	G	A_het	G	G	G
nonsynonymous SNV	OR4A16	chr11	55111071	A	C_het	A	A	A
nonsynonymous SNV	OR4A16	chr11	55111086	T	A_het	T	T	T
nonsynonymous SNV	OR4A16	chr11	55111157	C	G_het	C	C	C
nonsynonymous SNV	OR4A16	chr11	55111172	A	T_het	A	A	A
nonsynonymous SNV	OR4A16	chr11	55111200	C	T_het	C	C	C
nonsynonymous SNV	OR4A16	chr11	55111239	T	C_het	T	T	T
nonsynonymous SNV	OR4A16	chr11	55111284	T	C_het	T	T	T
nonsynonymous SNV	OR4A16	chr11	55111293	T	C_het	T	T	T
nonsynonymous SNV	OR4A16	chr11	55111298	A	G_het	A	A	A
nonsynonymous SNV	OR4A16	chr11	55111328	G	A_het	G	G	G
nonsynonymous SNV	OR4A16	chr11	55111341	T	C_het	T	T	T
nonsynonymous SNV	OR4A16	chr11	55111365	A	G_het	A	A	A
nonsynonymous SNV	OR4A16	chr11	55111382	C	T_het	C	C	C
nonsynonymous SNV	OR4A47	chr11	48510505	G	G	A_het	G	G
nonsynonymous SNV	OR4B1	chr11	48238704	G	G	G	G	A_het
nonsynonymous SNV	OR4C11	chr11	55371491	C	C	T_het	C	C
nonsynonymous SNV	OR4C15	chr11	55322140	T	T	T	T	C_het
nonsynonymous SNV	OR4C16	chr11	55340318	G	G	A_het	G	G
nonsynonymous SNV	OR4C3	chr11	48346523	C	T_het	T_het	T_het	T_het
nonsynonymous SNV	OR4C3	chr11	48346535	G	C_het	C_het	C_het	C_het
nonsynonymous SNV	OR4C3	chr11	48346541	A	T_het	T_het	T_het	T_het

nonsynonymous SNV	OR4C3	chr11	48346547	C	T_het	T_het	T_het	T_het
nonsynonymous SNV	OR4C3	chr11	48346551	C	A_het	A_het	A_het	A_het
nonsynonymous SNV	OR4C3	chr11	48346579	C	A_het	A_het	A_het	A_het
nonsynonymous SNV	OR4C3	chr11	48346588	A	C_het	C_het	C_het	C_het
nonsynonymous SNV	OR4C3	chr11	48346604	T	G_het	G_het	G_het	G_het
nonsynonymous SNV	OR4C3	chr11	48346791	C	T_het	T_het	T_het	T_het
nonsynonymous SNV	OR4C3	chr11	48346916	G	C_het	C_het	C_het	C_het
nonsynonymous SNV	OR4C3	chr11	48346932	G	A_het	A_het	A_het	A_het
nonsynonymous SNV	OR4C3	chr11	48346961	A	T_het	T_het	T_het	T_het
nonsynonymous SNV	OR4C3	chr11	48346962	A	G_het	G_het	G_het	G_het
stopgain	OR4C3	chr11	48347014	G	A_het	A_het	A_het	A_het
nonsynonymous SNV	OR4C3	chr11	48347067	C	T_het	T_het	T_het	T_het
nonsynonymous SNV	OR4C3	chr11	48347100	C	T_het	T_het	T_het	T_het
nonsynonymous SNV	OR4C3	chr11	48347124	A	G_het	G_het	G_het	G_het
nonsynonymous SNV	OR4C3	chr11	48347130	C	G_het	G_het	G_het	G_het
nonsynonymous SNV	OR4C3	chr11	48347142	C	T_het	T_het	T_het	T_het
nonsynonymous SNV	OR4C3	chr11	48347144	T	C_het	C_het	C_het	C_het
nonsynonymous SNV	OR4C3	chr11	48347267	T	C_het	C_het	C_het	C_het
nonsynonymous SNV	OR4C3	chr11	48347306	G	T_het	G	T_het	T_het
nonsynonymous SNV	OR4C3	chr11	48347342	G	A_het	A_het	A_het	A_het
nonsynonymous SNV	OR4C3	chr11	48347346	A	T_het	T_het	T_het	T_het
nonsynonymous SNV	OR4C3	chr11	48347358	C	T_het	T_het	T_het	T_het
nonsynonymous SNV	OR4C3	chr11	48347363	C	T_het	T_het	T_het	T_het
nonsynonymous SNV	OR4C45	chr11	48366958	T	G_het	G_het	G_het	G_het
nonsynonymous SNV	OR4C45	chr11	48366971	G	C_het	C_het	C_het	C_het
nonsynonymous SNV	OR4C45	chr11	48367097	A	G_het	G_het	G_het	G_het
nonsynonymous SNV	OR4C45	chr11	48367133	C	A_het	A_het	A_het	A_het
nonsynonymous SNV	OR4C45	chr11	48367155	T	A_het	A_het	A_het	A_het
nonsynonymous SNV	OR4C45	chr11	48367163	A	G_het	G_het	G_het	G_het
nonsynonymous SNV	OR4C45	chr11	48367190	A	T_het	T_het	T_het	T_het
nonsynonymous SNV	OR4C45	chr11	48367195	C	T_het	T_het	T_het	T_het
nonsynonymous SNV	OR4C45	chr11	48367311	T	C_het	C_het	C_het	C_het
nonsynonymous SNV	OR4C45	chr11	48367419	G	A_het	A_het	A_het	A_het
nonsynonymous SNV	OR4C45	chr11	48367424	C	G_het	G_het	G_het	G_het
nonsynonymous SNV	OR4C45	chr11	48367455	G	A_het	A_het	A_het	A_het
nonsynonymous SNV	OR4C45	chr11	48367470	G	A_het	A_het	A_het	A_het
nonsynonymous SNV	OR4C45	chr11	48367499	A	G_het	G_het	G_het	G_het
nonsynonymous SNV	OR4C45	chr11	48367511	T	G_het	G_het	G_het	G_het
nonsynonymous SNV	OR4C45	chr11	48373748	C	G_het	G_het	G_het	G_het
nonsynonymous SNV	OR4C45	chr11	48373770	T	A_het	A_het	A_het	A_het
nonsynonymous SNV	OR4C45	chr11	48373797	C	T_het	T_het	T_het	T_het
nonsynonymous SNV	OR4C45	chr11	48373815	A	G_het	G_het	G_het	G_het
stopgain	OR4C45	chr11	48373820	A	C_het	C_het	A	A
nonsynonymous SNV	OR4C45	chr11	48373833	G	A_het	A_het	A_het	A_het
nonsynonymous SNV	OR4C45	chr11	48373834	A	G_het	G_het	G_het	G_het

nonsynonymous SNV	OR4C45	chr11	48373885	A	G_het	G_het	G_het	G_het
nonsynonymous SNV	OR4C45	chr11	48373986	A	G_het	G_het	G_het	G_het
nonsynonymous SNV	OR4K1	chr14	20404340	A	A	G_het	A	A
nonsynonymous SNV	OR4K17	chr14	20585924	A	G_het	A	A	A
nonsynonymous SNV	OR4M1	chr14	20249176	G	G	G	A_het	A_het
nonsynonymous SNV	OR4M2	chr15	22368862	G	G	A_het	A_het	G
nonsynonymous SNV	OR4M2	chr15	22369248	T	C_hom	C_hom	C_hom	C_hom
nonsynonymous SNV	OR4M2	chr15	22369290	A	A	G_het	A	A
nonsynonymous SNV	OR4M2	chr15	22369357	C	C	T_het	C	C
nonsynonymous SNV	OR4M2	chr15	22369426	G	A_hom	A_het	A_het	A_het
nonsynonymous SNV	OR4N2	chr14	20295834	T	C_hom	C_het	T	C_het
nonsynonymous SNV	OR4N2	chr14	20296004	C	T_het	C	C	C
nonsynonymous SNV	OR4N2	chr14	20296010	G	C_het	G	G	G
nonsynonymous SNV	OR4N3P	chr15	22413825	C	C	T_het	C	C
nonsynonymous SNV	OR4N3P	chr15	22413844	T	T	T	T	G_het
nonsynonymous SNV	OR4N3P	chr15	22413940	T	C_hom	C_het	C_het	C_het
nonsynonymous SNV	OR4N3P	chr15	22413954	C	C	C	T_het	C
stopgain	OR4N3P	chr15	22414005	C	C	C	T_het	C
nonsynonymous SNV	OR4N3P	chr15	22414215	G	G	G	A_het	G
nonsynonymous SNV	OR51A4	chr11	4967651	A	G_het	A	A	A
nonsynonymous SNV	OR51B5	chr11	5364561	G	G	G	G	A_het
nonsynonymous SNV	OR51F1	chr11	4791021	C	C	T_het	C	C
nonsynonymous SNV	OR52A1	chr11	5173079	G	G	G	T_hom	G
nonsynonymous SNV	OR52D1	chr11	5510498	G	G	A_het	G	G
nonsynonymous SNV	OR52E2	chr11	5080653	C	C	C	T_hom	C
nonsynonymous SNV	OR52H1	chr11	5566002	G	A_het	G	G	G
nonsynonymous SNV	OR52Z1	chr11	5199490	T	T	T	G_hom	T
nonsynonymous SNV	OR56A5	chr11	5989191	C	C	C	G_hom	C
nonsynonymous SNV	OR56B4	chr11	6129487	T	T	T	C_hom	T
nonsynonymous SNV	OR5B12	chr11	58206714	C	C	T_het	C	C
nonsynonymous SNV	OR5C1	chr9	125551449	G	G	A_het	G	G
stopgain	OR5D13	chr11	55541505	C	C	C	C	T_het
nonsynonymous SNV	OR5D18	chr11	55587587	C	C	T_het	C	C
nonsynonymous SNV	OR5F1	chr11	55761459	G	G	T_het	G	G
nonsynonymous SNV	OR5H1	chr3	97851950	A	A	A	G_het	A
nonsynonymous SNV	OR5I1	chr11	55703506	C	C	T_het	C	C
nonsynonymous SNV	OR5K1	chr3	98188646	G	G	G	G	A_het
nonsynonymous SNV	OR5M9	chr11	56230318	C	C	C	T_hom	C
nonsynonymous SNV	OR5T1	chr11	56043293	C	C	C	T_hom	C
nonsynonymous SNV	OR6A2	chr11	6816224	C	C	C	T_hom	C
nonsynonymous SNV	OR6B3	chr2	240985347	G	A_het	G	G	G
nonsynonymous SNV	OR6C6	chr12	55688894	T	T	G_het	T	T
nonsynonymous SNV	OR6T1	chr11	123814286	G	G	G	A_hom	G
nonsynonymous SNV	OR6X1	chr11	123624673	G	A_het	G	G	G
nonsynonymous SNV	OR7G1	chr19	9225619	G	G	G	A_het	G

nonsynonymous SNV	OR8B2	chr11	124253170	G	G	G	G	A_het
nonsynonymous SNV	OR8B2	chr11	124253181	T	T	T	T	C_het
nonsynonymous SNV	OR8B3	chr11	124266906	C	C	C	C	T_het
nonsynonymous SNV	OR8G2	chr11	124096247	G	G	A_het	A_hom	A_hom
nonsynonymous SNV	OR8J3	chr11	55904499	T	T	T	T	G_het
nonsynonymous SNV	OR8U8	chr11	56143103	G	G	G	A_het	G
nonsynonymous SNV	OR8U8	chr11	56143108	C	C	C	G_het	C
nonsynonymous SNV	OR8U8	chr11	56143125	C	T_het	T_het	T_het	C
nonsynonymous SNV	OR8U8	chr11	56143250	G	A_het	A_het	A_het	A_het
nonsynonymous SNV	OR8U8	chr11	56143257	C	A_het	A_het	A_het	A_het
nonsynonymous SNV	OR8U8	chr11	56143370	G	A_het	A_het	A_het	A_het
nonsynonymous SNV	OR8U8	chr11	56143371	T	G_het	G_het	G_het	G_het
nonsynonymous SNV	OR8U8	chr11	56143382	G	A_het	A_het	A_het	G
nonsynonymous SNV	OR8U8	chr11	56143394	A	G_het	G_het	G_het	A
nonsynonymous SNV	OR8U8	chr11	56143415	A	G_het	G_het	G_het	A
nonsynonymous SNV	OR8U8	chr11	56143490	T	A_het	A_het	A_het	A_het
nonsynonymous SNV	OR8U8	chr11	56143508	A	T_het	T_het	T_het	T_het
nonsynonymous SNV	OR8U8	chr11	56143539	T	C_het	C_het	C_het	C_het
nonsynonymous SNV	OR8U8	chr11	56143544	T	C_het	C_het	C_het	C_het
nonsynonymous SNV	OR8U8	chr11	56143592	C	C	T_het	C	C
nonsynonymous SNV	OR8U8	chr11	56143716	T	C_het	C_het	C_het	C_het
nonsynonymous SNV	OR8U8	chr11	56143717	G	A_het	A_het	A_het	A_het
nonsynonymous SNV	OR8U8	chr11	56143725	C	G_het	G_het	G_het	G_het
nonsynonymous SNV	OR8U8	chr11	56143730	C	G_het	G_het	G_het	G_het
nonsynonymous SNV	OR8U8	chr11	56143767	C	T_het	T_het	T_het	C
nonsynonymous SNV	OR8U8	chr11	56143803	A	G_het	G_het	G_het	A
nonsynonymous SNV	OR8U8	chr11	56143804	G	C_het	C_het	C_het	G
nonsynonymous SNV	OR8U8	chr11	56143823	G	A_het	A_het	A_het	G
nonsynonymous SNV	OR8U8	chr11	56143898	G	G	T_het	G	G
nonsynonymous SNV	OR8U8	chr11	56143907	A	A	G_het	A	A
nonsynonymous SNV	OR9G9	chr11	56467945	G	G	T_het	G	G
nonsynonymous SNV	OR9G9	chr11	56468021	G	A_het	A_het	A_het	A_het
nonsynonymous SNV	OR9G9	chr11	56468111	C	C	T_het	T_het	T_het
nonsynonymous SNV	OR9G9	chr11	56468198	A	G_het	G_het	G_het	G_het
nonsynonymous SNV	OR9G9	chr11	56468368	C	T_het	T_het	T_het	T_het
nonsynonymous SNV	OR9G9	chr11	56468416	G	A_het	A_het	A_het	A_het
nonsynonymous SNV	OR9G9	chr11	56468440	G	G	T_het	G	G
nonsynonymous SNV	OR9G9	chr11	56468449	A	T_het	T_het	T_het	T_het
nonsynonymous SNV	OR9G9	chr11	56468452	A	C_het	C_het	C_het	C_het
nonsynonymous SNV	OR9G9	chr11	56468699	T	A_het	A_het	A_het	A_het
nonsynonymous SNV	OR9G9	chr11	56468704	T	C_het	C_het	C_het	C_het
nonsynonymous SNV	OR9G9	chr11	56468720	T	C_het	C_het	C_het	T
nonsynonymous SNV	ORC4	chr2	148693169	A	A	T_het	A	A
nonsynonymous SNV	ORM1	chr9	117085526	G	A_hom	A_hom	G	G
nonsynonymous SNV	OSBPL10	chr3	32022440	C	C	T_hom	C	C

nonsynonymous SNV	OSBPL8	chr12	76749680	T	T	T	A_het	T
nonsynonymous SNV	OSCP1	chr1	36883786	C	C	C	C	T_het
nonsynonymous SNV	OSCP1	chr1	36883837	T	T	A_het	T	T
nonsynonymous SNV	OSCP1	chr1	36883838	C	C	T_het	C	C
nonsynonymous SNV	OSM	chr22	30660198	C	C	T_het	C	C
nonsynonymous SNV	OTOA	chr16	21693115	G	G	G	A_het	G
nonsynonymous SNV	OTOA	chr16	21747633	A	C_het	A	A	A
stopgain	OTOA	chr16	21747639	G	T_het	G	G	G
nonsynonymous SNV	OTOGL	chr12	80712366	G	G	G	C_het	G
nonsynonymous SNV	OTUD4	chr4	146058639	A	A	A	A	C_het
nonsynonymous SNV	OTUD4	chr4	146065576	A	A	A	A	G_het
nonsynonymous SNV	OVCH1	chr12	29604443	G	G	G	G	C_het
nonsynonymous SNV	OVOS2	chr12	31270076	A	C_het	C_hom	C_hom	A
nonsynonymous SNV	OVOS2	chr12	31270149	G	G	G	C_hom	C_hom
nonsynonymous SNV	OVOS2	chr12	31270150	C	C	C	A_hom	A_hom
nonsynonymous SNV	OVOS2	chr12	31278451	A	A	G_het	A	A
nonsynonymous SNV	OVOS2	chr12	31282765	A	A	T_hom	T_het	T_het
nonsynonymous SNV	OXCT2	chr1	40236074	A	C_hom	A	A	A
nonsynonymous SNV	OXR1	chr8	107718960	A	A	A	C_het	A
nonsynonymous SNV	P2RX1	chr17	3801166	C	C	C	T_hom	C
nonsynonymous SNV	P2RY6	chr11	73007934	G	G	A_het	G	G
nonsynonymous SNV	PABPC1	chr8	101718932	C	G_het	C	G_het	C
nonsynonymous SNV	PABPC1	chr8	101718965	G	A_het	G	A_het	A_het
nonsynonymous SNV	PABPC1	chr8	101718968	C	T_het	C	T_het	T_het
nonsynonymous SNV	PABPC1	chr8	101719004	G	G	G	A_het	A_het
nonsynonymous SNV	PABPC1	chr8	101719121	G	G	G	A_het	A_het
nonsynonymous SNV	PABPC1	chr8	101719138	C	C	C	T_het	T_het
nonsynonymous SNV	PABPC1	chr8	101719201	A	A	A	G_het	G_het
nonsynonymous SNV	PABPC1	chr8	101721709	T	A_het	T	T	T
nonsynonymous SNV	PABPC1	chr8	101721812	G	A_het	G	A_het	A_het
nonsynonymous SNV	PABPC1	chr8	101721817	T	C_het	T	C_het	C_het
nonsynonymous SNV	PABPC1	chr8	101721839	C	A_het	C	A_het	A_het
nonsynonymous SNV	PABPC1	chr8	101721953	T	T	T	A_het	T
nonsynonymous SNV	PABPC3	chr13	25670767	A	G_het	A	A	A
nonsynonymous SNV	PABPC3	chr13	25670776	C	T_het	C	T_het	C
nonsynonymous SNV	PABPC3	chr13	25670780	C	G_het	C	G_het	C
nonsynonymous SNV	PABPC3	chr13	25670797	C	G_het	C	G_het	C
nonsynonymous SNV	PABPC3	chr13	25670803	A	G_het	A	G_het	A
nonsynonymous SNV	PABPC3	chr13	25670877	G	A_het	G	A_het	A_het
nonsynonymous SNV	PABPC3	chr13	25670907	C	A_het	C	A_het	A_het
nonsynonymous SNV	PABPC3	chr13	25670919	A	G_het	A	G_het	G_het
nonsynonymous SNV	PABPC3	chr13	25670953	G	A_het	G	A_het	A_het
nonsynonymous SNV	PABPC3	chr13	25670955	C	T_het	C	T_het	T_het
nonsynonymous SNV	PABPC3	chr13	25670988	T	G_het	T	G_het	G_het
nonsynonymous SNV	PABPC3	chr13	25671027	A	G_het	A	G_het	G_het

nonsynonymous SNV	PABPC3	chr13	25671089	G	T_het	G	T_het	T_het
nonsynonymous SNV	PABPC3	chr13	25671168	C	T_het	C	T_het	T_het
nonsynonymous SNV	PABPC3	chr13	25671172	C	A_het	C	A_het	A_het
nonsynonymous SNV	PABPC3	chr13	25671195	A	G_het	A	A	G_het
stopgain	PABPC3	chr13	25671210	C	T_het	C	T_het	T_het
nonsynonymous SNV	PABPC3	chr13	25671214	T	G_het	T	G_het	G_het
nonsynonymous SNV	PABPC3	chr13	25671268	G	T_het	G	G	G
nonsynonymous SNV	PABPC3	chr13	25671271	A	G_het	A	G_het	G_het
nonsynonymous SNV	PABPC3	chr13	25671274	C	T_het	C	T_het	T_het
nonsynonymous SNV	PABPC3	chr13	25671292	C	T_het	C	T_het	T_het
stopgain	PABPC3	chr13	25671369	G	T_het	G	G	T_het
nonsynonymous SNV	PABPC3	chr13	25671429	G	T_het	G	G	G
nonsynonymous SNV	PABPC3	chr13	25671451	A	G_het	A	A	A
nonsynonymous SNV	PABPC3	chr13	25671456	C	T_het	C	C	C
nonsynonymous SNV	PABPC3	chr13	25671742	G	A_het	G	G	G
nonsynonymous SNV	PABPC3	chr13	25671759	C	T_het	C	C	C
nonsynonymous SNV	PACSIN1	chr6	34498307	C	C	T_het	C	C
nonsynonymous SNV	PACSIN3	chr11	47202073	C	C	T_het	C	C
nonsynonymous SNV	PADI3	chr1	17609404	G	G	G	G	A_het
nonsynonymous SNV	PADI4	chr1	17657607	C	C	C	C	G_het
nonsynonymous SNV	PADI4	chr1	17664615	T	T	T	C_hom	T
nonsynonymous SNV	PADI4	chr1	17668840	T	T	T	C_hom	T
nonsynonymous SNV	PAK6	chr15	40558460	C	C	C	A_het	C
nonsynonymous SNV	PAK6	chr15	40558610	C	C	C	T_het	C
nonsynonymous SNV	PALM2-AKAP2	chr9	112900784	C	C	C	C	T_het
nonsynonymous SNV	PAPLN	chr14	73729246	C	C	A_het	C	C
nonsynonymous SNV	PAPOLG	chr2	61021198	C	C	T_het	C	C
nonsynonymous SNV	PAPPA	chr9	118950150	A	A	C_het	A	A
nonsynonymous SNV	PAPPA	chr9	118973981	A	A	G_het	A	A
nonsynonymous SNV	PARD3B	chr2	206041271	G	G	G	G	A_het
nonsynonymous SNV	PARK7	chr1	8030993	C	C	T_het	C	C
nonsynonymous SNV	PARL	chr3	183562033	G	G	A_het	G	G
nonsynonymous SNV	PARP10	chr8	145051711	C	C	C	C	A_hom
nonsynonymous SNV	PARP10	chr8	145057230	G	G	G	G	A_hom
nonsynonymous SNV	PARP15	chr3	122329458	C	T_hom	C	C	C
nonsynonymous SNV	PARP3	chr3	51980281	C	C	C	T_hom	C
nonsynonymous SNV	PARP4	chr13	25021201	G	G	G	G	A_het
nonsynonymous SNV	PARP4	chr13	25021245	A	G_het	G_het	G_het	G_het
nonsynonymous SNV	PARP4	chr13	25021263	T	C_het	T	T	C_het
nonsynonymous SNV	PARP4	chr13	25021273	C	C	C	T_het	C
nonsynonymous SNV	PARP4	chr13	25021323	A	G_het	A	A	G_het
nonsynonymous SNV	PARP8	chr5	50090747	G	G	T_het	G	G
nonsynonymous SNV	PARPBP	chr12	102517803	C	C	T_het	C	C
nonsynonymous SNV	PASK	chr2	242063459	G	G	G	A_het	G
nonsynonymous SNV	PATZ1	chr22	31741174	C	C	T_het	C	C

nonsynonymous SNV	PAX1	chr20	21686561	C	C	C	C	T_hom
nonsynonymous SNV	PAX5	chr9	36966682	G	G	G	G	A_het
nonsynonymous SNV	PAX8	chr2	113993073	A	G_het	A	A	A
nonsynonymous SNV	PAX9	chr14	37132608	G	G	A_het	G	G
nonsynonymous SNV	PAXIP1	chr7	154767716	G	G	A_het	G	G
nonsynonymous SNV	PC	chr11	66617096	C	T_het	C	C	C
nonsynonymous SNV	PCDH10	chr4	134072185	C	C	T_het	C	C
nonsynonymous SNV	PCDH11X	chrX	91133218	C	C	C	C	T_het
nonsynonymous SNV	PCDH12	chr5	141336264	G	T_hom	T_het	T_hom	T_hom
nonsynonymous SNV	PCDH15	chr10	55582636	T	T	C_het	T	T
nonsynonymous SNV	PCDH18	chr4	138450989	G	G	A_het	G	G
nonsynonymous SNV	PCDH9	chr13	67477668	G	G	G	T_het	G
nonsynonymous SNV	PCDHA1	chr5	140166686	G	G	C_het	G	G
nonsynonymous SNV	PCDHA1	chr5	140167311	C	C	T_het	C	C
nonsynonymous SNV	PCDHA1	chr5	140167463	C	C	C	C	G_hom
nonsynonymous SNV	PCDHA11	chr5	140249998	C	C	C	G_het	C
nonsynonymous SNV	PCDHA12	chr5	140257044	G	G	G	G	A_het
nonsynonymous SNV	PCDHA13	chr5	140262697	A	A	A	G_het	A
nonsynonymous SNV	PCDHA7	chr5	140214381	G	A_hom	A_het	G	A_hom
nonsynonymous SNV	PCDHA8	chr5	140221071	G	T_het	G	G	G
nonsynonymous SNV	PCDHA8	chr5	140223047	C	C	C	A_het	C
nonsynonymous SNV	PCDHA9	chr5	140228264	C	C	T_het	C	C
nonsynonymous SNV	PCDHA9	chr5	140229086	C	C	G_het	G_het	C
nonsynonymous SNV	PCDHA9	chr5	140230025	G	G	T_het	G	G
nonsynonymous SNV	PCDHAC1	chr5	140307823	A	A	A	G_het	A
nonsynonymous SNV	PCDHB10	chr5	140573754	A	C_hom	C_het	C_het	C_het
nonsynonymous SNV	PCDHB13	chr5	140594467	G	A_het	G	G	G
nonsynonymous SNV	PCDHB13	chr5	140594470	G	A_het	G	G	A_het
nonsynonymous SNV	PCDHB13	chr5	140595106	C	C	C	G_het	C
nonsynonymous SNV	PCDHB15	chr5	140627002	C	C	C	C	G_het
nonsynonymous SNV	PCDHB16	chr5	140564304	C	C	C	T_het	C
nonsynonymous SNV	PCDHB17	chr5	140536037	C	C	T_het	C	C
nonsynonymous SNV	PCDHB18	chr5	140615997	G	G	T_het	G	G
nonsynonymous SNV	PCDHB2	chr5	140476230	T	T	T	C_het	T
nonsynonymous SNV	PCDHB6	chr5	140531099	G	G	A_het	G	G
nonsynonymous SNV	PCDHB7	chr5	140553893	T	T	T	T	C_het
nonsynonymous SNV	PCDHB8	chr5	140559078	C	C	C	C	T_het
nonsynonymous SNV	PCDHB9	chr5	140568998	C	T_het	T_het	C	T_het
nonsynonymous SNV	PCDHB9	chr5	140569128	T	T	T	T	C_het
nonsynonymous SNV	PCDHGA11	chr5	140802127	G	G	G	G	T_het
nonsynonymous SNV	PCDHGA7	chr5	140762973	T	T	T	G_het	T
nonsynonymous SNV	PCDHGA8	chr5	140773158	C	C	C	T_het	C
nonsynonymous SNV	PCDHGA8	chr5	140773738	C	T_hom	C	C	C
stopgain	PCDHGA8	chr5	140774619	C	C	C	T_het	C
nonsynonymous SNV	PCDHGA8	chr5	140774628	C	C	C	G_het	C

nonsynonymous SNV	PCDHGA9	chr5	140784066	C	C	C	C	T_het
nonsynonymous SNV	PCDHGB2	chr5	140741611	G	T_hom	G	G	G
nonsynonymous SNV	PCDHGB3	chr5	140750757	G	G	A_het	G	G
nonsynonymous SNV	PCDHGB4	chr5	140768577	C	C	C	G_het	C
nonsynonymous SNV	PCDHGB7	chr5	140798860	C	C	C	A_het	C
nonsynonymous SNV	PCDHGB7	chr5	140798879	G	G	G	G	A_het
nonsynonymous SNV	PCDHGB7	chr5	140799641	G	G	T_het	G	G
nonsynonymous SNV	PCDHGC5	chr5	140871290	G	G	G	A_het	G
nonsynonymous SNV	PCED1B	chr12	47628929	C	C	G_het	C	C
nonsynonymous SNV	PCED1B	chr12	47629337	C	C	T_het	C	C
nonsynonymous SNV	PCID2	chr13	113851352	C	C	C	T_het	C
nonsynonymous SNV	PCK1	chr20	56137763	T	T	C_het	T	T
nonsynonymous SNV	PCLO	chr7	82578875	G	G	A_het	G	G
nonsynonymous SNV	PCM1	chr8	17814924	T	T	T	C_het	T
nonsynonymous SNV	PCM1	chr8	17823611	G	A_het	G	G	G
nonsynonymous SNV	PCMTD1	chr8	52733128	T	T	G_het	G_het	G_het
nonsynonymous SNV	PCNXL2	chr1	233122016	A	A	G_het	A	A
nonsynonymous SNV	PCNXL3	chr11	65394881	G	A_het	G	G	G
nonsynonymous SNV	PCOLCE2	chr3	142542415	C	T_het	C	C	C
nonsynonymous SNV	PCSK5	chr9	78601144	C	C	T_het	C	C
nonsynonymous SNV	PCSK5	chr9	78790182	G	G	G	G	C_hom
nonsynonymous SNV	PCSK5	chr9	78790187	G	C_hom	G	G	C_hom
stopgain	PCSK5	chr9	78790202	C	C	C	C	A_hom
nonsynonymous SNV	PCSK5	chr9	78790211	T	T	T	T	C_hom
stoploss	PCSK5	chr9	78790216	T	T	T	T	C_hom
nonsynonymous SNV	PCSK6	chr15	101845512	C	C	T_het	C	C
nonsynonymous SNV	PCYOX1	chr2	70504388	A	A	A	A	T_het
nonsynonymous SNV	PDCD1LG2	chr9	5534924	C	G_hom	C	C	C
nonsynonymous SNV	PDCD7	chr15	65425651	C	C	C	T_het	C
nonsynonymous SNV	PDE1B	chr12	54963024	C	C	C	C	T_het
nonsynonymous SNV	PDE1C	chr7	31862756	T	C_het	T	T	T
nonsynonymous SNV	PDE3A	chr12	20786695	G	G	A_het	G	G
stopgain	PDE4DIP	chr1	144852390	C	T_het	T_het	T_het	T_het
nonsynonymous SNV	PDE4DIP	chr1	144854581	T	C_het	C_het	T	C_het
nonsynonymous SNV	PDE4DIP	chr1	144854598	C	T_het	T_het	T_het	T_het
nonsynonymous SNV	PDE4DIP	chr1	144856941	C	C	T_het	C	C
nonsynonymous SNV	PDE4DIP	chr1	144863401	T	C_het	C_het	C_het	T
stopgain	PDE4DIP	chr1	144864277	G	A_het	G	G	G
nonsynonymous SNV	PDE4DIP	chr1	144865850	G	T_het	T_het	T_het	T_het
nonsynonymous SNV	PDE4DIP	chr1	144866643	G	A_het	A_het	A_het	A_het
nonsynonymous SNV	PDE4DIP	chr1	144868170	C	T_het	T_het	T_het	T_het
nonsynonymous SNV	PDE4DIP	chr1	144871738	C	A_het	A_het	A_het	A_het
nonsynonymous SNV	PDE4DIP	chr1	144871755	A	T_het	T_het	T_het	T_het
nonsynonymous SNV	PDE4DIP	chr1	144871782	A	G_het	G_het	G_het	G_het
nonsynonymous SNV	PDE4DIP	chr1	144874815	T	C_het	C_het	C_het	C_het

nonsynonymous SNV	PDE4DIP	chr1	144877176	C	T_het	T_het	T_het	T_het
nonsynonymous SNV	PDE4DIP	chr1	144879090	T	C_het	C_het	C_het	C_het
nonsynonymous SNV	PDE4DIP	chr1	144879339	C	C	C	C	T_het
nonsynonymous SNV	PDE4DIP	chr1	144879375	T	C_het	C_het	C_het	C_het
nonsynonymous SNV	PDE4DIP	chr1	144880832	T	C_het	C_het	C_het	C_het
nonsynonymous SNV	PDE4DIP	chr1	144882823	C	T_het	T_het	T_het	T_het
nonsynonymous SNV	PDE4DIP	chr1	144886197	A	T_het	T_het	T_het	T_het
nonsynonymous SNV	PDE4DIP	chr1	144912152	C	C	T_het	C	C
nonsynonymous SNV	PDE4DIP	chr1	144912233	C	T_het	T_het	T_het	T_het
stopgain	PDE4DIP	chr1	144915561	G	A_het	A_het	A_het	A_het
nonsynonymous SNV	PDE4DIP	chr1	144916748	C	G_het	G_het	G_het	G_het
nonsynonymous SNV	PDE4DIP	chr1	144917841	T	C_het	C_het	C_het	C_het
nonsynonymous SNV	PDE4DIP	chr1	144918957	T	A_het	A_het	A_het	A_het
nonsynonymous SNV	PDE4DIP	chr1	144922583	G	A_het	A_het	A_het	A_het
nonsynonymous SNV	PDE4DIP	chr1	144930774	G	G	A_het	G	G
nonsynonymous SNV	PDE4DIP	chr1	144930940	T	C_het	C_het	C_het	C_het
nonsynonymous SNV	PDE4DIP	chr1	144931087	T	C_het	C_het	C_het	C_het
nonsynonymous SNV	PDE4DIP	chr1	144931330	C	T_het	T_het	T_het	T_het
nonsynonymous SNV	PDE4DIP	chr1	144931392	G	A_het	A_het	A_het	A_het
nonsynonymous SNV	PDE4DIP	chr1	144931461	A	T_het	T_het	T_het	T_het
nonsynonymous SNV	PDE4DIP	chr1	145015877	G	T_het	T_het	T_het	G
stopgain	PDE4DIP	chr1	145075683	C	T_het	T_het	T_het	T_het
nonsynonymous SNV	PDE6B	chr4	628493	G	A_het	G	G	G
nonsynonymous SNV	PDE6C	chr10	95400270	C	C	T_het	C	C
nonsynonymous SNV	PDE8A	chr15	85657120	A	A	G_het	A	A
nonsynonymous SNV	PDE8B	chr5	76700601	A	A	A	G_het	A
nonsynonymous SNV	PDE8B	chr5	76714058	G	G	A_het	G	G
nonsynonymous SNV	PDGFRB	chr5	149514363	A	G_het	A	A	A
nonsynonymous SNV	PDHA2	chr4	96762158	G	G	C_het	G	G
nonsynonymous SNV	PDK4	chr7	95222104	C	C	T_het	C	C
nonsynonymous SNV	PDLIM4	chr5	131607294	T	T	C_het	T	T
nonsynonymous SNV	PDLIM7	chr5	176918893	T	T	C_het	T	T
nonsynonymous SNV	PDPR	chr16	70154486	G	G	G	C_het	G
nonsynonymous SNV	PDPR	chr16	70161263	T	C_het	C_het	C_het	T
nonsynonymous SNV	PDPR	chr16	70164334	A	G_het	G_het	G_het	A
nonsynonymous SNV	PDPR	chr16	70170222	G	T_het	T_het	G	G
nonsynonymous SNV	PDPR	chr16	70182390	T	G_het	G_het	G_het	T
nonsynonymous SNV	PDS5A	chr4	39839591	C	C	T_het	C	C
nonsynonymous SNV	PDS5B	chr13	33273890	G	G	A_het	G	G
nonsynonymous SNV	PDXDC1	chr16	15098047	C	C	G_het	G_het	G_hom
nonsynonymous SNV	PDXDC1	chr16	15111218	C	C	T_het	T_het	T_hom
nonsynonymous SNV	PDXDC2P	chr16	70016576	G	G	G	G	A_het
nonsynonymous SNV	PDZRN3	chr3	73433649	C	C	T_hom	C	C
nonsynonymous SNV	PDZRN4	chr12	41967573	A	A	C_het	A	A
nonsynonymous SNV	PDZRN4	chr12	41967652	C	C	C	C	T_het

stopgain	PEAK1	chr15	77425553	G	G	A_het	G	G
nonsynonymous SNV	PEAR1	chr1	156878473	C	T_het	C	C	C
nonsynonymous SNV	PEAR1	chr1	156882757	C	C	C	G_hom	C
nonsynonymous SNV	PEBP4	chr8	22582358	C	C	C	C	T_hom
nonsynonymous SNV	PEPD	chr19	33892653	C	C	C	C	T_het
nonsynonymous SNV	PEPD	chr19	33991878	T	T	T	T	C_het
nonsynonymous SNV	PET117	chr20	18118630	C	C	C	C	T_het
nonsynonymous SNV	PEX16	chr11	45937311	G	G	G	G	A_het
nonsynonymous SNV	PFKP	chr10	3155627	C	C	T_het	C	C
nonsynonymous SNV	PFKP	chr10	3162112	C	C	T_het	C	C
nonsynonymous SNV	PGAM4	chrX	77225061	C	C	C	C	G_hom
nonsynonymous SNV	PGLYRP1	chr19	46526272	C	C	C	C	T_het
nonsynonymous SNV	PGLYRP3	chr1	153270485	G	G	T_het	G	G
nonsynonymous SNV	PGLYRP3	chr1	153276435	A	A	G_het	A	A
nonsynonymous SNV	PGPEP1L	chr15	99511745	C	C	G_het	C	C
nonsynonymous SNV	PHF11	chr13	50097338	T	G_hom	T	T	T
nonsynonymous SNV	PHF15	chr5	133900193	G	G	A_het	G	G
nonsynonymous SNV	PHF15	chr5	133914375	C	C	T_het	C	C
nonsynonymous SNV	PHF21B	chr22	45312306	C	T_het	C	C	C
nonsynonymous SNV	PHF3	chr6	64395196	G	G	A_het	G	G
nonsynonymous SNV	PHF8	chrX	54014331	C	C	T_hom	C	C
nonsynonymous SNV	PHLDA1	chr12	76425301	C	C	C	T_het	C
nonsynonymous SNV	PHLDB2	chr3	111603969	T	C_het	T	T	T
nonsynonymous SNV	PHLDB3	chr19	43983666	G	A_het	G	G	G
nonsynonymous SNV	PHYHD1	chr9	131702702	T	T	T	T	C_hom
nonsynonymous SNV	PI4KA	chr22	21088835	G	G	A_het	G	G
nonsynonymous SNV	PI4KA	chr22	21119432	C	C	T_het	C	C
nonsynonymous SNV	PI4KA	chr22	21174032	C	C	T_het	C	C
nonsynonymous SNV	PI4KAP2	chr22	21829507	G	G	A_het	G	G
nonsynonymous SNV	PIAS4	chr19	4033463	G	G	A_het	G	G
nonsynonymous SNV	PIEZO2	chr18	10794790	G	G	T_het	G	G
nonsynonymous SNV	PIF1	chr15	65108864	C	C	C	T_het	C
nonsynonymous SNV	PIGC	chr1	172411650	C	T_het	C	C	C
nonsynonymous SNV	PIGG	chr4	520908	C	C	C	C	A_het
stopgain	PIGO	chr9	35090215	G	G	A_het	G	G
nonsynonymous SNV	PIGO	chr9	35091528	G	G	A_het	G	G
nonsynonymous SNV	PIGO	chr9	35091549	C	C	T_het	C	C
nonsynonymous SNV	PIK3C2A	chr11	17132074	A	A	A	A	G_het
nonsynonymous SNV	PIK3CB	chr3	138413742	G	G	A_het	G	G
nonsynonymous SNV	PIK3R5	chr17	8791494	T	T	C_het	T	T
nonsynonymous SNV	PIKFYVE	chr2	209215654	C	C	C	C	T_het
nonsynonymous SNV	PILRB	chr7	99956444	A	A	G_het	A	A
nonsynonymous SNV	PINX1	chr8	10690474	T	T	G_het	T	T
nonsynonymous SNV	PIP4K2A	chr10	22830790	G	G	G	T_het	G
nonsynonymous SNV	PIP5K1C	chr19	3633464	G	G	A_het	G	G

nonsynonymous SNV	PIP5K1P1	chr6	7986779	G	G	A_het	G	G
nonsynonymous SNV	PIPOX	chr17	27380585	G	G	G	G	A_hom
nonsynonymous SNV	PIPSL	chr10	95720459	T	C_het	T	T	T
nonsynonymous SNV	PIPSL	chr10	95720748	C	C	T_het	C	C
nonsynonymous SNV	PITPNM3	chr17	6358942	C	C	T_hom	C	C
nonsynonymous SNV	PITPNM3	chr17	6386999	G	G	G	A_hom	G
nonsynonymous SNV	PITRM1	chr10	3189880	C	C	C	T_het	C
nonsynonymous SNV	PKD1	chr16	2158623	T	T	T	C_het	T
nonsynonymous SNV	PKD1	chr16	2159557	C	T_het	C	C	C
nonsynonymous SNV	PKD1L1	chr7	47894550	C	C	C	C	T_het
nonsynonymous SNV	PKDREJ	chr22	46658471	G	G	A_het	G	G
nonsynonymous SNV	PKHD1	chr6	51484226	C	C	T_het	C	C
nonsynonymous SNV	PKHD1	chr6	51875133	G	G	G	A_hom	G
nonsynonymous SNV	PKHD1	chr6	51890265	T	T	T	C_hom	T
nonsynonymous SNV	PKHD1	chr6	51917987	G	G	G	C_hom	G
nonsynonymous SNV	PKHD1L1	chr8	110472023	T	T	C_het	T	T
nonsynonymous SNV	PKHD1L1	chr8	110477390	G	G	G	A_het	G
nonsynonymous SNV	PKHD1L1	chr8	110520058	C	C	T_het	C	C
nonsynonymous SNV	PKHD1L1	chr8	110535006	C	C	C	C	T_het
nonsynonymous SNV	PKN1	chr19	14562767	G	G	A_het	G	G
nonsynonymous SNV	PKP1	chr1	201297929	G	A_het	G	G	G
nonsynonymous SNV	PKP2	chr12	32955486	G	G	G	G	A_het
nonsynonymous SNV	PKP2	chr12	33030861	T	T	T	T	G_het
nonsynonymous SNV	PKP2	chr12	33031352	G	G	G	C_het	G
nonsynonymous SNV	PKP3	chr11	397331	G	G	C_het	G	G
stopgain	PLA1A	chr3	119316814	G	G	A_het	G	G
nonsynonymous SNV	PLA2G15	chr16	68279384	T	T	T	A_het	T
nonsynonymous SNV	PLA2G15	chr16	68293450	C	C	T_het	C	C
nonsynonymous SNV	PLA2G4B	chr15	42136708	C	C	C	T_het	C
stopgain	PLA2G4B	chr15	42137161	C	C	C	T_het	C
nonsynonymous SNV	PLA2G4D	chr15	42371755	C	C	T_het	C	C
nonsynonymous SNV	PLA2G4D	chr15	42377708	C	C	C	C	T_het
nonsynonymous SNV	PLA2G4E	chr15	42305933	A	A	A	A	T_het
nonsynonymous SNV	PLA2G7	chr6	46684792	A	T_het	A	A	A
nonsynonymous SNV	PLAU	chr10	75672059	G	G	A_het	G	G
nonsynonymous SNV	PLAU	chr10	75676220	G	G	G	G	T_het
nonsynonymous SNV	PLB1	chr2	28748165	A	G_het	A	A	A
nonsynonymous SNV	PLB1	chr2	28812569	G	G	A_het	G	G
nonsynonymous SNV	PLB1	chr2	28812894	C	C	C	T_het	C
nonsynonymous SNV	PLB1	chr2	28814005	G	G	G	C_het	G
nonsynonymous SNV	PLCB2	chr15	40590824	G	G	G	C_het	G
nonsynonymous SNV	PLCB3	chr11	64022778	C	C	T_het	C	C
nonsynonymous SNV	PLCB4	chr20	9440331	A	A	A	A	G_het
nonsynonymous SNV	PLCD1	chr3	38050867	C	C	T_hom	C	C
nonsynonymous SNV	PLCL1	chr2	198949344	A	A	G_het	A	A

nonsynonymous SNV	PLCL2	chr3	16926599	T	T	A_hom	T	T
nonsynonymous SNV	PLCXD2	chr3	111432752	T	T	T	T	C_het
nonsynonymous SNV	PLEC	chr8	144990961	T	T	G_het	T	T
nonsynonymous SNV	PLEC	chr8	144994028	C	C	C	T_het	C
nonsynonymous SNV	PLEK2	chr14	67859398	G	G	G	G	C_het
nonsynonymous SNV	PLEKHA6	chr1	204199639	G	G	A_het	G	G
nonsynonymous SNV	PLEKHA6	chr1	204225946	G	G	A_het	G	G
nonsynonymous SNV	PLEKHA7	chr11	16807807	G	G	A_het	G	G
nonsynonymous SNV	PLEKHA8P1	chr12	45568082	T	T	C_het	T	T
nonsynonymous SNV	PLEKHG1	chr6	151054790	G	G	T_het	G	G
nonsynonymous SNV	PLEKHG3	chr14	65209802	G	G	A_het	G	G
nonsynonymous SNV	PLEKHG4B	chr5	140781	G	G	A_het	G	G
nonsynonymous SNV	PLEKHH1	chr14	68038954	G	G	A_het	G	G
nonsynonymous SNV	PLEKHH2	chr2	43931157	G	G	A_het	G	G
nonsynonymous SNV	PLGRKT	chr9	5361143	G	G	C_het	G	G
nonsynonymous SNV	PLIN4	chr19	4511283	C	T_het	T_het	C	T_het
nonsynonymous SNV	PLIN4	chr19	4511350	T	A_het	A_hom	A_het	A_hom
nonsynonymous SNV	PLIN4	chr19	4511746	A	A	A	T_het	A
nonsynonymous SNV	PLIN4	chr19	4512413	C	T_het	C	C	C
nonsynonymous SNV	PLIN4	chr19	4512591	C	C	C	C	T_het
nonsynonymous SNV	PLK2	chr5	57754132	G	C_hom	C_hom	C_hom	C_hom
nonsynonymous SNV	PLOD2	chr3	145790358	C	C	T_het	C	C
nonsynonymous SNV	PLOD2	chr3	145878767	A	A	A	T_hom	A
nonsynonymous SNV	PLVAP	chr19	17476444	C	C	C	G_het	C
nonsynonymous SNV	PLXNA1	chr3	126710303	C	C	T_het	C	C
nonsynonymous SNV	PLXNA1	chr3	126749178	G	G	A_het	G	G
nonsynonymous SNV	PLXNA2	chr1	208234056	G	G	G	G	A_het
nonsynonymous SNV	PLXNA2	chr1	208383539	A	A	C_het	A	A
nonsynonymous SNV	PLXNA4	chr7	131865473	G	A_het	G	G	G
nonsynonymous SNV	PNPLA2	chr11	823742	C	C	C	C	T_het
nonsynonymous SNV	PNPLA6	chr19	7622110	C	C	A_het	C	C
nonsynonymous SNV	PNPLA7	chr9	140357963	G	G	A_het	G	G
nonsynonymous SNV	POC1B	chr12	89853443	A	A	A	G_het	A
nonsynonymous SNV	POC1B-GALNT4	chr12	89917365	A	A	A	T_het	A
nonsynonymous SNV	PODNL1	chr19	14043661	G	G	A_het	G	G
nonsynonymous SNV	POGK	chr1	166818310	G	C_het	G	G	G
nonsynonymous SNV	POGLUT1	chr3	119196200	C	C	T_het	C	C
nonsynonymous SNV	POLB	chr8	42226805	C	C	G_het	C	C
nonsynonymous SNV	POLD1	chr19	50918708	G	G	A_het	G	G
nonsynonymous SNV	POLE	chr12	133202816	C	T_het	C	C	C
nonsynonymous SNV	POLE2	chr14	50121532	A	A	A	G_het	A
nonsynonymous SNV	POLG	chr15	89873322	T	T	C_het	T	T
nonsynonymous SNV	POLR2H	chr3	184085970	C	C	T_het	C	C
nonsynonymous SNV	POLR2J2	chr7	102279621	C	T_het	T_het	C	C
nonsynonymous SNV	POLR3B	chr12	106786858	A	A	A	A	C_hom

nonsynonymous SNV	POLR3B	chr12	106838243	A	A	T_het	A	A
nonsynonymous SNV	POLRMT	chr19	619632	C	C	T_het	C	C
nonsynonymous SNV	POM121	chr7	72395789	C	T_het	C	C	C
nonsynonymous SNV	POM121	chr7	72413423	C	C	C	T_het	T_het
nonsynonymous SNV	POM121	chr7	72413443	G	A_het	A_het	A_het	A_het
nonsynonymous SNV	POM121	chr7	72413581	G	A_het	A_het	A_het	A_het
nonsynonymous SNV	POM121	chr7	72413593	T	C_het	C_het	C_het	C_het
nonsynonymous SNV	POM121L12	chr7	53103729	G	G	G	C_het	G
nonsynonymous SNV	POM121L4P	chr22	21045405	A	A	A	A	G_het
nonsynonymous SNV	POMT1	chr9	134381580	T	T	T	C_hom	T
nonsynonymous SNV	POMZP3	chr7	76255360	G	G	G	C_het	G
nonsynonymous SNV	PON1	chr7	94944645	A	C_het	A	A	A
nonsynonymous SNV	POSTN	chr13	38172820	A	A	G_het	A	A
nonsynonymous SNV	POTEC	chr18	14537949	A	G_het	A	A	A
nonsynonymous SNV	POTEC	chr18	14542791	C	C	T_het	T_het	C
nonsynonymous SNV	POTEC	chr18	14542931	C	T_het	C	C	C
nonsynonymous SNV	POTEC	chr18	14542949	T	C_het	T	T	T
nonsynonymous SNV	POTEC	chr18	14542979	A	A	G_het	G_het	G_het
nonsynonymous SNV	POTEC	chr18	14543039	T	T	C_het	C_het	T
nonsynonymous SNV	POTEE	chr2	131976105	G	G	A_het	G	G
nonsynonymous SNV	POTEE	chr2	132020977	C	C	C	T_het	C
nonsynonymous SNV	POTEE	chr2	132021475	G	G	A_het	A_het	G
nonsynonymous SNV	POTEF	chr2	130832358	T	T	T	C_het	T
nonsynonymous SNV	POTEF	chr2	130877752	T	C_het	C_het	C_het	C_het
nonsynonymous SNV	POTEG	chr14	19553642	A	A	G_het	A	A
nonsynonymous SNV	POTEH	chr22	16287851	G	G	G	G	A_het
nonsynonymous SNV	POTEJ	chr2	131414176	C	T_het	T_het	T_het	T_het
nonsynonymous SNV	POU6F1	chr12	51584292	C	C	T_het	C	C
nonsynonymous SNV	PPAPDC1A	chr10	122334653	G	G	A_het	G	G
nonsynonymous SNV	PPARGC1A	chr4	23886322	C	C	C	T_hom	C
nonsynonymous SNV	PPFIA3	chr19	49652315	G	G	A_het	G	G
nonsynonymous SNV	PPIAL4G	chr1	143767513	A	T_het	T_het	T_het	T_het
nonsynonymous SNV	PPIAL4G	chr1	143767628	C	T_het	C	C	C
nonsynonymous SNV	PPIAL4G	chr1	143767778	T	A_het	A_het	A_het	A_het
nonsynonymous SNV	PPIAL4G	chr1	143767833	T	C_het	T	C_het	T
nonsynonymous SNV	PPIL1	chr6	36839598	C	G_het	C	C	C
nonsynonymous SNV	PPL	chr16	4934373	G	G	A_het	G	G
nonsynonymous SNV	PPOX	chr1	161138816	G	G	G	G	A_het
nonsynonymous SNV	PPP1R13B	chr14	104204129	G	G	A_het	G	G
nonsynonymous SNV	PPP1R13L	chr19	45895586	G	G	G	G	A_het
nonsynonymous SNV	PPP1R26	chr9	138379895	A	A	C_het	A	A
nonsynonymous SNV	PPP1R36	chr14	65017661	T	T	T	A_het	T
nonsynonymous SNV	PPP1R3A	chr7	113519312	C	C	C	T_het	C
nonsynonymous SNV	PPP1R3A	chr7	113519666	G	G	G	G	A_hom
nonsynonymous SNV	PPP1R3E	chr14	23770908	A	A	A	G_het	A

nonsynonymous SNV	PPP1R3G	chr6	5086314	C	C	T_het	C	C
nonsynonymous SNV	PPP1R9A	chr7	94917985	C	C	C	G_het	C
nonsynonymous SNV	PPP2R5B	chr11	64695897	G	G	G	G	A_het
nonsynonymous SNV	PPP2R5B	chr11	64697828	G	G	A_het	G	G
nonsynonymous SNV	PPP4R2	chr3	73113180	C	C	C	C	T_het
nonsynonymous SNV	PPP6R1	chr19	55752885	C	T_het	C	C	C
nonsynonymous SNV	PQLC2	chr1	19653785	C	C	T_het	C	C
nonsynonymous SNV	PRAMEF1	chr1	12854068	T	T	T	C_het	T
stopgain	PRAMEF1	chr1	12854090	T	A_het	A_het	T	A_het
nonsynonymous SNV	PRAMEF1	chr1	12854105	A	G_het	G_het	A	G_het
nonsynonymous SNV	PRAMEF1	chr1	12854162	C	C	C	T_het	T_het
nonsynonymous SNV	PRAMEF1	chr1	12854188	T	T	T	C_het	T
nonsynonymous SNV	PRAMEF1	chr1	12854356	T	T	T	T	C_het
nonsynonymous SNV	PRAMEF1	chr1	12854414	G	G	G	G	A_het
nonsynonymous SNV	PRAMEF1	chr1	12855667	A	A	A	A	T_het
stopgain	PRAMEF1	chr1	12856105	C	C	C	C	G_het
nonsynonymous SNV	PRAMEF1	chr1	12856111	C	C	C	C	T_het
nonsynonymous SNV	PRAMEF10	chr1	12952767	C	C	C	C	G_het
nonsynonymous SNV	PRAMEF10	chr1	12952770	C	C	C	C	T_het
nonsynonymous SNV	PRAMEF13	chr1	13448199	C	C	C	C	T_hom
nonsynonymous SNV	PRAMEF2	chr1	12921365	A	A	A	A	C_het
nonsynonymous SNV	PRAMEF6	chr1	13001312	C	G_het	G_het	C	G_het
nonsynonymous SNV	PRB1	chr12	11506669	G	T_het	T_het	G	T_het
nonsynonymous SNV	PRB4	chr12	11461706	G	T_het	T_het	G	G
nonsynonymous SNV	PRDM10	chr11	129814902	C	C	T_het	C	C
nonsynonymous SNV	PRDM11	chr11	45247798	G	G	A_het	G	G
nonsynonymous SNV	PRDM11	chr11	45249009	G	G	A_het	G	G
nonsynonymous SNV	PRDM15	chr21	43254643	G	G	G	G	A_het
nonsynonymous SNV	PRDM15	chr21	43274693	C	C	C	T_het	C
nonsynonymous SNV	PRDM2	chr1	14107112	C	C	C	T_hom	C
nonsynonymous SNV	PRDM2	chr1	14149672	A	A	G_het	A	A
nonsynonymous SNV	PRDM8	chr4	81123086	G	G	A_het	G	G
nonsynonymous SNV	PRDM9	chr5	23524508	T	A_het	T	T	T
nonsynonymous SNV	PREX2	chr8	68989671	G	G	G	A_het	G
nonsynonymous SNV	PREX2	chr8	69009312	T	T	G_het	T	T
nonsynonymous SNV	PRH2	chr12	11083356	A	A	G_hom	A	G_het
nonsynonymous SNV	PRHOXNB	chr13	28552314	G	G	G	C_het	G
nonsynonymous SNV	PRIM2	chr6	57244780	G	A_het	A_het	A_het	A_het
nonsynonymous SNV	PRIM2	chr6	57246884	A	G_het	G_het	G_het	G_het
nonsynonymous SNV	PRIM2	chr6	57246982	G	T_het	T_het	T_het	T_het
nonsynonymous SNV	PRIM2	chr6	57393125	A	G_het	G_het	G_het	G_het
nonsynonymous SNV	PRIM2	chr6	57398154	T	C_het	C_het	C_het	C_het
nonsynonymous SNV	PRIM2	chr6	57398157	A	G_het	G_het	G_het	G_het
nonsynonymous SNV	PRIM2	chr6	57398163	A	G_het	G_het	G_het	G_het
nonsynonymous SNV	PRIM2	chr6	57398201	T	C_het	C_het	C_het	C_het

stopgain	PRIM2	chr6	57398207	G	G	G	G	T_het
nonsynonymous SNV	PRIM2	chr6	57398226	T	G_het	G_het	A_het	A_het
nonsynonymous SNV	PRIM2	chr6	57398264	A	G_het	G_het	G_het	G_het
stopgain	PRIM2	chr6	57398270	C	T_het	C	T_het	T_het
nonsynonymous SNV	PRIM2	chr6	57467084	A	G_het	G_het	G_het	G_het
nonsynonymous SNV	PRIM2	chr6	57467093	A	G_het	A	G_het	G_het
nonsynonymous SNV	PRIM2	chr6	57467107	C	T_het	C	T_het	T_het
nonsynonymous SNV	PRIM2	chr6	57512476	G	A_het	A_het	A_het	A_het
nonsynonymous SNV	PRIM2	chr6	57512529	C	A_het	A_het	A_het	A_het
nonsynonymous SNV	PRIM2	chr6	57512565	T	C_het	C_het	C_het	C_het
nonsynonymous SNV	PRKAG2	chr7	151257489	A	A	A	T_het	A
nonsynonymous SNV	PRKCD	chr3	53220653	G	G	G	G	T_het
nonsynonymous SNV	PRKDC	chr8	48707061	A	A	G_het	A	A
nonsynonymous SNV	PRKDC	chr8	48771426	G	G	T_het	G	G
nonsynonymous SNV	PRKDC	chr8	48852241	G	G	A_het	G	G
nonsynonymous SNV	PRLR	chr5	35068957	C	T_het	C	C	C
nonsynonymous SNV	PRMT2	chr21	48071838	C	C	C	T_het	C
nonsynonymous SNV	PROKR2	chr20	5294763	G	G	G	C_het	G
nonsynonymous SNV	PROSER1	chr13	39587813	T	T	C_het	T	T
nonsynonymous SNV	PRPF38A	chr1	52882420	G	G	G	A_hom	G
nonsynonymous SNV	PRPF4	chr9	116053207	A	A	G_het	A	A
nonsynonymous SNV	PRPF40B	chr12	50025308	C	C	T_het	C	C
nonsynonymous SNV	PRPF6	chr20	62626438	G	G	A_het	G	G
nonsynonymous SNV	PRPF6	chr20	62631075	G	G	A_het	G	G
nonsynonymous SNV	PRR12	chr19	50118173	G	G	A_het	G	G
nonsynonymous SNV	PRR14L	chr22	32081779	T	T	C_het	T	T
nonsynonymous SNV	PRR21	chr2	240982309	C	C	C	T_het	C
nonsynonymous SNV	PRR22	chr19	5783846	C	C	C	C	T_het
nonsynonymous SNV	PRR23C	chr3	138762861	G	G	A_het	G	G
nonsynonymous SNV	PRRT4	chr7	127992178	C	C	G_het	C	C
nonsynonymous SNV	PRSS1	chr7	142457343	C	C	C	T_het	C
nonsynonymous SNV	PRSS1	chr7	142457375	C	C	C	G_het	C
nonsynonymous SNV	PRSS1	chr7	142458409	C	C	G_het	G_het	C
nonsynonymous SNV	PRSS1	chr7	142458412	C	C	T_het	T_het	C
nonsynonymous SNV	PRSS1	chr7	142458414	C	C	T_het	T_het	C
nonsynonymous SNV	PRSS1	chr7	142458511	G	G	G	T_het	G
nonsynonymous SNV	PRSS1	chr7	142458523	T	T	T	A_het	T
nonsynonymous SNV	PRSS1	chr7	142458526	A	A	A	G_het	A
nonsynonymous SNV	PRSS1	chr7	142458527	C	C	C	G_het	C
stopgain	PRSS1	chr7	142458531	C	C	C	T_het	C
nonsynonymous SNV	PRSS1	chr7	142458597	C	C	T_het	C	C
nonsynonymous SNV	PRSS1	chr7	142458737	C	C	C	G_het	G_het
nonsynonymous SNV	PRSS1	chr7	142458837	A	T_het	T_het	T_het	T_het
nonsynonymous SNV	PRSS1	chr7	142460335	A	G_het	G_het	G_het	G_het
nonsynonymous SNV	PRSS1	chr7	142460369	G	A_het	A_het	A_het	A_het

nonsynonymous SNV	PRSS1	chr7	142460744	G	C_het	C_het	C_het	C_het
nonsynonymous SNV	PRSS1	chr7	142460752	C	G_het	G_het	G_het	G_het
nonsynonymous SNV	PRSS1	chr7	142460764	G	A_het	A_het	A_het	G
nonsynonymous SNV	PRSS1	chr7	142460779	G	T_het	T_het	G	G
nonsynonymous SNV	PRSS12	chr4	119203329	C	C	T_het	C	C
nonsynonymous SNV	PRSS12	chr4	119273553	G	G	G	C_hom	G
nonsynonymous SNV	PRSS3	chr9	33794809	G	G	G	A_het	G
nonsynonymous SNV	PRSS3	chr9	33794812	G	G	G	T_het	G
nonsynonymous SNV	PRSS3	chr9	33795593	G	A_het	G	G	A_het
nonsynonymous SNV	PRSS3	chr9	33795603	G	C_het	G	C_het	C_het
nonsynonymous SNV	PRSS3	chr9	33796673	G	A_het	G	G	A_het
nonsynonymous SNV	PRSS3	chr9	33796785	C	C	C	G_het	C
nonsynonymous SNV	PRSS3	chr9	33796799	A	T_het	T_het	T_het	T_het
nonsynonymous SNV	PRSS3	chr9	33797861	A	G_het	G_het	G_het	G_het
nonsynonymous SNV	PRSS3	chr9	33797951	A	C_het	C_het	C_het	C_het
nonsynonymous SNV	PRSS3	chr9	33797987	G	T_het	T_het	T_het	T_het
nonsynonymous SNV	PRSS3	chr9	33797999	A	G_het	G_het	G_het	G_het
nonsynonymous SNV	PRSS3	chr9	33798042	G	C_het	C_het	C_het	C_het
nonsynonymous SNV	PRSS3	chr9	33798075	T	C_het	C_het	C_het	C_het
nonsynonymous SNV	PRSS3	chr9	33798574	G	G	G	A_het	G
nonsynonymous SNV	PRSS3P2	chr7	142479915	T	C_hom	C_het	C_het	C_hom
nonsynonymous SNV	PRSS3P2	chr7	142479954	C	T_hom	T_het	T_het	T_hom
nonsynonymous SNV	PRSS3P2	chr7	142480002	C	A_hom	A_het	A_het	A_hom
nonsynonymous SNV	PRSS3P2	chr7	142480067	C	T_hom	T_het	T_het	T_hom
nonsynonymous SNV	PRSS8	chr16	31146797	C	C	T_het	C	C
nonsynonymous SNV	PRX	chr19	40900457	C	C	C	G_het	C
nonsynonymous SNV	PSD2	chr5	139197136	G	G	G	C_het	G
nonsynonymous SNV	PSD3	chr8	18729328	C	C	A_het	C	C
nonsynonymous SNV	PSD4	chr2	113942568	C	C	T_het	C	C
nonsynonymous SNV	PSG2	chr19	43585111	C	G_het	C	C	C
nonsynonymous SNV	PSG8	chr19	43262381	G	G	G	G	T_het
nonsynonymous SNV	PSMD2	chr3	184017128	A	A	G_hom	A	A
nonsynonymous SNV	PSMD9	chr12	122332727	C	C	C	C	T_het
nonsynonymous SNV	PSPC1	chr13	20277413	T	C_het	C_het	C_het	C_het
nonsynonymous SNV	PSPC1	chr13	20279879	T	T	T	C_het	T
nonsynonymous SNV	PSPH	chr7	56087300	C	C	T_het	T_het	T_het
nonsynonymous SNV	PSPH	chr7	56087319	T	T	G_het	G_het	G_het
nonsynonymous SNV	PSPH	chr7	56087365	A	A	G_het	G_het	G_het
nonsynonymous SNV	PSPH	chr7	56087374	C	C	T_het	T_het	T_het
nonsynonymous SNV	PSPH	chr7	56087399	G	G	A_het	A_het	A_het
nonsynonymous SNV	PSPH	chr7	56087423	G	G	A_het	A_het	A_het
nonsynonymous SNV	PSPH	chr7	56088811	T	T	C_het	C_het	C_het
nonsynonymous SNV	PSPH	chr7	56088825	T	T	A_het	A_het	A_het
nonsynonymous SNV	PSPH	chr7	56088902	C	C	C	T_het	T_het
nonsynonymous SNV	PSPH	chr7	56088907	G	G	G	C_het	C_het

nonsynonymous SNV	PSPH	chr7	56088908	C	C	C	T_het	T_het
nonsynonymous SNV	PTBP3	chr9	115024842	T	T	G_het	T	T
nonsynonymous SNV	PTCD2	chr5	71648493	T	T	T	A_het	T
nonsynonymous SNV	PTCH2	chr1	45288988	C	C	C	T_hom	C
nonsynonymous SNV	PTCH2	chr1	45293709	G	A_hom	G	G	G
nonsynonymous SNV	PTCH2	chr1	45295296	C	C	T_het	C	C
nonsynonymous SNV	PTCH2	chr1	45297968	A	A	A	A	G_het
nonsynonymous SNV	PTCSC3	chr14	36623085	C	C	C	T_het	C
nonsynonymous SNV	PTGDR2	chr11	60621078	C	T_het	C	C	C
nonsynonymous SNV	PTGIR	chr19	47126807	G	T_het	G	G	G
nonsynonymous SNV	PTGIR	chr19	47126849	G	G	A_het	G	G
nonsynonymous SNV	PTGIR	chr19	47127439	A	G_het	A	A	A
nonsynonymous SNV	PTGR2	chr14	74340799	G	A_het	G	G	G
nonsynonymous SNV	PTGR2	chr14	74346825	C	C	C	T_het	C
nonsynonymous SNV	PTGS1	chr9	125143793	A	A	G_het	A	A
nonsynonymous SNV	PTH	chr11	13514381	C	C	T_het	C	C
nonsynonymous SNV	PTH1R	chr3	46940169	G	G	A_hom	G	G
nonsynonymous SNV	PTPN21	chr14	88946499	C	C	C	T_het	C
nonsynonymous SNV	PTPN23	chr3	47454400	C	C	A_hom	C	C
nonsynonymous SNV	PTPRB	chr12	70933409	T	T	C_het	T	T
nonsynonymous SNV	PTPRC	chr1	198704373	A	A	A	A	T_het
nonsynonymous SNV	PTPRF	chr1	44057026	C	C	T_het	C	C
nonsynonymous SNV	PTPRF	chr1	44086164	C	C	C	C	T_het
nonsynonymous SNV	PTPRH	chr19	55715287	C	C	T_het	C	C
nonsynonymous SNV	PTPRJ	chr11	48142777	A	A	A	G_hom	A
nonsynonymous SNV	PTPRJ	chr11	48149491	G	G	A_het	G	G
nonsynonymous SNV	PTPRQ	chr12	80982097	A	A	G_het	A	A
nonsynonymous SNV	PTPRT	chr20	40713353	C	C	C	T_het	C
nonsynonymous SNV	PTPRU	chr1	29606026	G	G	A_het	G	G
nonsynonymous SNV	PTPRZ1	chr7	121653137	G	G	G	A_het	G
nonsynonymous SNV	PTX3	chr3	157154776	G	G	G	C_hom	G
nonsynonymous SNV	PTX3	chr3	157155647	C	C	C	T_hom	C
nonsynonymous SNV	PTX4	chr16	1536412	T	T	T	C_het	T
nonsynonymous SNV	PTX4	chr16	1537374	C	C	T_het	C	C
nonsynonymous SNV	PUS10	chr2	61187545	C	C	T_het	C	C
nonsynonymous SNV	PUS3	chr11	125766022	C	C	T_het	C	C
nonsynonymous SNV	PVRIG	chr7	99818403	C	C	C	T_het	C
nonsynonymous SNV	PXDNL	chr8	52321966	G	A_het	G	G	G
nonsynonymous SNV	PXDNL	chr8	52323908	A	A	G_het	A	A
nonsynonymous SNV	PXDNL	chr8	52366115	C	T_het	C	C	C
nonsynonymous SNV	PYGL	chr14	51382112	C	C	C	T_het	C
nonsynonymous SNV	PYGL	chr14	51382637	G	A_het	G	G	G
nonsynonymous SNV	PYGM	chr11	64519958	T	T	C_het	T	T
nonsynonymous SNV	PYGO1	chr15	55839278	G	G	C_het	G	G
nonsynonymous SNV	PYROXD2	chr10	100150491	T	T	T	G_het	T

nonsynonymous SNV	QPRT	chr16	29706344	G	G	A_het	G	G
nonsynonymous SNV	QPRT	chr16	29708541	G	G	G	A_het	G
nonsynonymous SNV	QRFPR	chr4	122251659	T	T	C_het	T	T
nonsynonymous SNV	QSOX2	chr9	139115868	C	C	T_het	C	C
nonsynonymous SNV	R3HCC1L	chr10	99968849	T	T	G_het	T	T
nonsynonymous SNV	RAB11FIP1	chr8	37730222	C	C	C	C	G_hom
nonsynonymous SNV	RAB11FIP1	chr8	37732066	G	G	G	A_het	G
nonsynonymous SNV	RAB1A	chr2	65318134	A	A	G_het	A	A
nonsynonymous SNV	RAB28	chr4	13462335	C	C	T_het	C	C
nonsynonymous SNV	RAB44	chr6	36689596	G	G	A_het	G	G
nonsynonymous SNV	RAB6A	chr11	73388956	C	G_het	C	C	C
nonsynonymous SNV	RABEP1	chr17	5281567	T	T	T	T	C_hom
nonsynonymous SNV	RAD21	chr8	117864867	A	A	A	C_het	A
nonsynonymous SNV	RAD21	chr8	117868995	C	C	C	G_het	C
nonsynonymous SNV	RAD50	chr5	131940624	G	G	A_het	G	G
nonsynonymous SNV	RAD51AP1	chr12	4662172	G	G	G	G	A_het
nonsynonymous SNV	RAD51L3-RFFL	chr17	33430313	T	T	C_het	T	T
nonsynonymous SNV	RAD54L	chr1	46715717	T	T	C_het	T	T
nonsynonymous SNV	RAG1	chr11	36595653	G	G	A_het	G	G
nonsynonymous SNV	RAI1	chr17	17696416	A	A	G_het	A	A
nonsynonymous SNV	RANBP2	chr2	109365399	C	C	C	T_het	C
nonsynonymous SNV	RANBP2	chr2	109380602	C	C	T_het	C	C
nonsynonymous SNV	RANBP2	chr2	109382936	T	T	T	A_het	T
nonsynonymous SNV	RAP1GAP	chr1	21926063	C	C	C	T_hom	C
nonsynonymous SNV	RAPGEF4	chr2	173679016	A	A	G_het	A	A
nonsynonymous SNV	RAPGEF5	chr7	22259571	C	C	T_het	C	C
nonsynonymous SNV	RAPH1	chr2	204305101	G	G	A_het	G	G
nonsynonymous SNV	RASA4	chr7	102235769	T	T	T	C_het	T
nonsynonymous SNV	RASA4	chr7	102246394	A	A	A	T_het	A
nonsynonymous SNV	RASAL2	chr1	178427309	A	A	A	A	T_het
nonsynonymous SNV	RASGEF1B	chr4	82355804	C	C	T_het	C	C
nonsynonymous SNV	RASIP1	chr19	49225167	C	C	T_het	C	C
nonsynonymous SNV	RASL11B	chr4	53731700	A	G_het	A	A	A
nonsynonymous SNV	RASSF10	chr11	13031991	C	C	C	C	T_het
nonsynonymous SNV	RASSF10	chr11	13031992	C	C	C	C	T_het
nonsynonymous SNV	RASSF4	chr10	45480414	A	A	G_het	A	A
nonsynonymous SNV	RASSF7	chr11	562408	T	T	C_het	T	T
nonsynonymous SNV	RBFOX2	chr22	36424450	A	C_hom	A	A	C_hom
nonsynonymous SNV	RBM19	chr12	114352870	C	C	T_het	C	C
nonsynonymous SNV	RBM28	chr7	127954847	G	G	A_het	G	G
nonsynonymous SNV	RBM28	chr7	127975992	C	C	G_het	C	C
nonsynonymous SNV	RBM34	chr1	235323959	T	T	C_het	T	T
nonsynonymous SNV	RBMX	chrX	135956342	C	G_het	G_het	G_het	G_het
nonsynonymous SNV	RBMX	chrX	135956408	A	G_het	G_het	G_het	G_het
nonsynonymous SNV	RBMX	chrX	135956462	G	C_het	C_het	C_het	C_het

nonsynonymous SNV	RBMX	chrX	135956467	C	T_het	T_het	T_het	T_het
nonsynonymous SNV	RBMX	chrX	135956506	C	G_het	G_het	G_het	G_het
nonsynonymous SNV	RBMXL1	chr1	89448896	A	A	A	T_het	A
nonsynonymous SNV	RBMXL1	chr1	89448914	A	A	A	G_het	A
nonsynonymous SNV	RBMXL1	chr1	89448924	T	T	T	C_het	T
nonsynonymous SNV	RBMXL1	chr1	89449278	C	T_het	C	T_het	C
nonsynonymous SNV	RBMXL1	chr1	89449298	C	G_het	C	G_het	C
nonsynonymous SNV	RBMXL1	chr1	89449390	T	C_het	T	C_het	C_het
nonsynonymous SNV	RBMXL1	chr1	89449434	T	C_het	T	T	T
nonsynonymous SNV	RBMXL1	chr1	89449483	C	G_het	C	C	C
nonsynonymous SNV	RBP3	chr10	48386030	G	G	G	T_het	G
nonsynonymous SNV	RBP3	chr10	48389149	G	G	G	G	A_het
nonsynonymous SNV	RBPJL	chr20	43942218	C	C	C	G_het	C
nonsynonymous SNV	RBPMS	chr8	30402089	C	C	T_het	C	C
nonsynonymous SNV	RDH13	chr19	55556574	G	G	C_het	G	G
nonsynonymous SNV	RDH8	chr19	10129508	G	G	G	G	A_het
nonsynonymous SNV	REC8	chr14	24647334	G	G	G	T_het	G
nonsynonymous SNV	RECQL4	chr8	145737295	C	C	C	T_het	C
nonsynonymous SNV	RECQL4	chr8	145739416	C	C	T_het	C	C
nonsynonymous SNV	RECQL5	chr17	73626706	C	C	T_het	C	C
nonsynonymous SNV	REG3A	chr2	79385787	G	A_het	A_het	G	A_het
nonsynonymous SNV	RELL2	chr5	141019830	C	G_hom	C	C	C
nonsynonymous SNV	RELN	chr7	103191623	C	C	T_het	C	C
nonsynonymous SNV	REPS1	chr6	139234084	G	G	G	A_hom	G
nonsynonymous SNV	RERE	chr1	8421186	G	A_hom	G	G	G
stopgain	REV3L	chr6	111631256	G	G	A_het	G	G
nonsynonymous SNV	RFC1	chr4	39328201	C	C	C	C	A_het
nonsynonymous SNV	RFC3	chr13	34404147	T	T	T	C_het	T
nonsynonymous SNV	RFPL2	chr22	32588901	G	G	A_het	G	G
nonsynonymous SNV	RFPL3	chr22	32756703	C	T_het	C	C	C
nonsynonymous SNV	RFPL4A	chr19	56274453	G	G	G	A_het	A_het
nonsynonymous SNV	RFPL4AL1	chr19	56284457	G	G	A_het	A_het	A_het
nonsynonymous SNV	RFPL4AL1	chr19	56284529	C	C	A_het	A_het	A_het
nonsynonymous SNV	RFPL4AL1	chr19	56284535	A	A	G_het	G_het	G_het
nonsynonymous SNV	RFWD2	chr1	176175819	A	C_het	C_het	A	A
nonsynonymous SNV	RFX5	chr1	151315500	C	C	T_het	C	C
nonsynonymous SNV	RFX8	chr2	102038824	C	T_het	C	C	C
nonsynonymous SNV	RGAG4	chrX	71349700	C	C	C	T_hom	C
nonsynonymous SNV	RGL3	chr19	11517423	G	G	G	C_het	G
nonsynonymous SNV	RGPD3	chr2	107041599	C	C	C	G_het	C
nonsynonymous SNV	RGPD3	chr2	107073501	C	T_hom	T_hom	T_het	T_hom
nonsynonymous SNV	RGPD4	chr2	108475928	C	T_het	C	C	C
nonsynonymous SNV	RGS12	chr4	3318063	G	G	G	G	A_het
nonsynonymous SNV	RGS12	chr4	3318813	C	C	C	C	T_het
nonsynonymous SNV	RGS22	chr8	101011594	G	G	G	A_het	G

nonsynonymous SNV	RGS22	chr8	101052261	A	A	A	T_het	A
nonsynonymous SNV	RHBDD1	chr2	227729791	G	G	G	A_het	G
nonsynonymous SNV	RHBDF2	chr17	74467972	C	C	T_het	C	C
nonsynonymous SNV	RHBG	chr1	156347250	G	G	G	G	A_het
nonsynonymous SNV	RHOT2	chr16	721863	C	C	C	T_het	C
nonsynonymous SNV	RHOT2	chr16	723111	G	G	A_het	G	G
nonsynonymous SNV	RHPN1	chr8	144457769	G	G	A_het	G	G
nonsynonymous SNV	RIC3	chr11	8161570	T	T	T	T	C_het
nonsynonymous SNV	RIC8A	chr11	209425	G	G	G	C_hom	G
nonsynonymous SNV	RIF1	chr2	152320892	G	G	G	G	A_het
nonsynonymous SNV	RIMBP2	chr12	130919410	C	C	C	T_het	C
nonsynonymous SNV	RIMS1	chr6	73017023	A	A	A	A	G_het
nonsynonymous SNV	RIN1	chr11	66101085	C	C	C	T_hom	C
nonsynonymous SNV	RIN1	chr11	66102135	C	C	C	C	A_het
nonsynonymous SNV	RIOK1	chr6	7395350	G	G	A_het	G	G
nonsynonymous SNV	RLBP1	chr15	89754001	G	A_hom	G	G	G
nonsynonymous SNV	RLF	chr1	40703538	G	G	G	A_hom	G
nonsynonymous SNV	RMND5B	chr5	177574579	G	G	T_het	G	G
nonsynonymous SNV	RNASE1	chr14	21270166	C	C	C	T_het	C
nonsynonymous SNV	RNASEH2A	chr19	12923929	G	G	G	G	A_het
nonsynonymous SNV	RNASEH2B	chr13	51530530	G	G	G	G	T_hom
nonsynonymous SNV	RNASET2	chr6	167343204	C	T_hom	C	C	C
nonsynonymous SNV	RNF10	chr12	121001654	C	C	T_het	C	C
nonsynonymous SNV	RNF126	chr19	651684	C	C	C	C	T_het
nonsynonymous SNV	RNF165	chr18	44036029	T	G_het	T	T	T
nonsynonymous SNV	RNF168	chr3	196199169	C	C	C	T_hom	C
nonsynonymous SNV	RNF17	chr13	25404696	G	G	G	G	A_hom
nonsynonymous SNV	RNF186	chr1	20141135	G	G	G	G	A_het
nonsynonymous SNV	RNF19B	chr1	33430102	T	T	T	T	G_hom
nonsynonymous SNV	RNF213	chr17	78252686	G	G	G	G	C_hom
nonsynonymous SNV	RNF213	chr17	78272230	C	C	G_het	C	C
nonsynonymous SNV	RNF213	chr17	78360641	C	C	T_het	C	C
nonsynonymous SNV	RNF25	chr2	219528898	C	C	G_het	C	C
nonsynonymous SNV	RNF44	chr5	175959412	C	C	T_het	C	C
nonsynonymous SNV	RNF5P1	chr8	38458651	G	G	A_het	G	G
nonsynonymous SNV	ROBO2	chr3	75986663	C	A_het	A_het	A_het	A_het
nonsynonymous SNV	ROBO2	chr3	75986717	G	A_het	A_het	A_het	G
nonsynonymous SNV	ROBO3	chr11	124744019	C	C	C	T_hom	C
nonsynonymous SNV	ROBO4	chr11	124764115	G	G	T_het	G	G
nonsynonymous SNV	ROCK2	chr2	11389842	A	C_het	A	A	A
nonsynonymous SNV	ROM1	chr11	62380800	G	A_het	G	G	G
nonsynonymous SNV	ROR2	chr9	94493395	G	G	A_het	G	G
nonsynonymous SNV	ROS1	chr6	117718232	C	C	T_het	C	C
nonsynonymous SNV	RP1	chr8	55538687	C	C	C	T_het	C
nonsynonymous SNV	RP1L1	chr8	10466726	G	G	A_het	G	G

nonsynonymous SNV	RP1L1	chr8	10467094	G	G	A_het	G	G
nonsynonymous SNV	RP1L1	chr8	10467637	T	C_het	C_hom	T	T
nonsynonymous SNV	RP1L1	chr8	10467647	G	C_het	G	G	G
nonsynonymous SNV	RP1L1	chr8	10467652	G	G	G	C_het	G
nonsynonymous SNV	RP1L1	chr8	10470757	G	G	G	A_het	G
nonsynonymous SNV	RP1L1	chr8	10480225	G	C_het	G	G	G
nonsynonymous SNV	RPAIN	chr17	5329392	G	G	G	G	T_hom
nonsynonymous SNV	RPAP1	chr15	41816145	C	C	T_het	C	C
nonsynonymous SNV	RPL21P44	chr4	54852923	A	A	A	G_hom	A
nonsynonymous SNV	RPL22	chr1	6257744	C	C	C	C	A_het
nonsynonymous SNV	RPL23AP53	chr8	169298	A	A	A	A	T_het
nonsynonymous SNV	RPL34-AS1	chr4	109478030	G	G	G	G	T_het
nonsynonymous SNV	RPL3L	chr16	1996958	C	C	T_het	C	C
nonsynonymous SNV	RPL8	chr8	146016869	T	T	C_het	T	T
nonsynonymous SNV	RPRD2	chr1	150445499	G	G	G	G	A_het
nonsynonymous SNV	RPS19	chr19	42365183	C	C	T_het	C	C
nonsynonymous SNV	RPS24	chr10	79814599	A	A	A	G_het	A
nonsynonymous SNV	RPS5	chr19	58904796	G	G	A_het	G	G
nonsynonymous SNV	RPTOR	chr17	78857695	G	G	A_het	G	G
nonsynonymous SNV	RPTOR	chr17	78936266	A	G_het	A	A	A
nonsynonymous SNV	RRBP1	chr20	17610512	C	C	T_het	C	C
nonsynonymous SNV	RRBP1	chr20	17617277	C	C	T_het	C	C
nonsynonymous SNV	RRBP1	chr20	17639876	T	T	T	T	G_het
nonsynonymous SNV	RRBP1	chr20	17640621	G	G	A_het	G	G
nonsynonymous SNV	RRM1	chr11	4148284	G	G	G	A_hom	G
nonsynonymous SNV	RRN3	chr16	15178499	G	G	G	A_het	G
nonsynonymous SNV	RRN3P1	chr16	21814036	C	C	C	T_het	C
nonsynonymous SNV	RRP15	chr1	218478430	A	A	G_het	A	A
nonsynonymous SNV	RRP7A	chr22	42912129	C	C	C	T_hom	C
nonsynonymous SNV	RRP7A	chr22	42914033	T	T	C_het	T	T
nonsynonymous SNV	RSPH10B	chr7	5983063	C	T_het	C	T_het	C
nonsynonymous SNV	RSPH10B	chr7	5983070	T	T	C_het	T	T
nonsynonymous SNV	RSPH6A	chr19	46307742	G	G	G	G	A_het
nonsynonymous SNV	RSPH6A	chr19	46318286	G	G	G	G	A_het
nonsynonymous SNV	RTEL1-TNFRSF6B	chr20	62328236	G	G	G	A_het	G
nonsynonymous SNV	RTEL1-TNFRSF6B	chr20	62329654	C	C	T_het	C	C
nonsynonymous SNV	RTN4	chr2	55277209	C	C	C	T_het	C
nonsynonymous SNV	RTN4RL1	chr17	1840143	C	C	C	T_hom	C
nonsynonymous SNV	RUFY1	chr5	179007951	T	T	T	G_het	T
nonsynonymous SNV	RUFY2	chr10	70143816	G	T_het	G	G	G
nonsynonymous SNV	RUFY3	chr4	71628284	C	C	T_het	C	C
nonsynonymous SNV	RUNX2	chr6	45296467	G	G	A_het	G	G
nonsynonymous SNV	RUSC2	chr9	35556108	C	C	T_het	C	C
stopgain	RXFP1	chr4	159568062	G	G	T_het	G	G
nonsynonymous SNV	RYR2	chr1	237774102	A	A	C_het	A	A

nonsynonymous SNV	RYR2	chr1	237778082	G	G	G	A_hom	G
nonsynonymous SNV	S100PBP	chr1	33318692	A	A	G_het	A	A
nonsynonymous SNV	SAA2	chr11	18267463	A	G_het	G_het	A	G_het
nonsynonymous SNV	SAA2	chr11	18267478	G	A_het	A_het	G	G
nonsynonymous SNV	SACS	chr13	23929373	G	G	G	A_het	G
nonsynonymous SNV	SAFB2	chr19	5594022	C	C	T_het	C	C
nonsynonymous SNV	SAG	chr2	234229296	G	G	T_het	G	G
nonsynonymous SNV	SAGE1	chrX	134983830	C	T_het	C	C	C
nonsynonymous SNV	SALL1	chr16	51175649	C	C	T_het	C	C
nonsynonymous SNV	SALL1	chr16	51175661	T	T	C_het	T	T
nonsynonymous SNV	SALL2	chr14	21994239	C	C	C	T_het	C
nonsynonymous SNV	SALL3	chr18	76753121	T	C_het	T	T	T
nonsynonymous SNV	SALL3	chr18	76754105	G	G	A_het	G	G
nonsynonymous SNV	SARS	chr1	109779073	G	G	G	A_hom	G
nonsynonymous SNV	SBF1	chr22	50885599	C	C	T_het	C	C
nonsynonymous SNV	SBF2	chr11	9861208	G	C_het	G	G	G
nonsynonymous SNV	SC5D	chr11	121178151	G	G	G	A_hom	G
nonsynonymous SNV	SCAP	chr3	47455441	C	C	T_hom	C	C
nonsynonymous SNV	SCAP	chr3	47476572	G	A_hom	G	G	G
nonsynonymous SNV	SCARA5	chr8	27737199	C	C	T_het	C	C
nonsynonymous SNV	SCARB2	chr4	77102127	T	T	C_het	T	T
nonsynonymous SNV	SCGB2A1	chr11	61977935	G	G	G	A_hom	G
nonsynonymous SNV	SCGN	chr6	25701469	G	G	A_het	G	G
nonsynonymous SNV	SCIN	chr7	12617815	G	G	G	A_het	G
nonsynonymous SNV	SCN10A	chr3	38739106	G	G	G	G	A_het
nonsynonymous SNV	SCN11A	chr3	38961462	G	G	G	G	A_het
nonsynonymous SNV	SCN2A	chr2	166201225	A	A	A	G_het	A
nonsynonymous SNV	SCN4A	chr17	62034710	C	T_het	C	C	C
nonsynonymous SNV	SCN4A	chr17	62045451	G	G	A_het	G	G
nonsynonymous SNV	SCN5A	chr3	38607989	C	C	C	T_hom	C
nonsynonymous SNV	SCNN1D	chr1	1226512	G	G	A_het	G	G
nonsynonymous SNV	SCRIB	chr8	144892984	C	C	C	T_het	C
nonsynonymous SNV	SCTR	chr2	120204442	G	C_het	G	G	G
nonsynonymous SNV	SCUBE3	chr6	35203239	G	G	T_het	G	G
nonsynonymous SNV	SCYL1	chr11	65303580	A	A	G_het	A	A
nonsynonymous SNV	SDE2	chr1	226173217	T	T	T	T	C_het
nonsynonymous SNV	SDHA	chr5	251707	T	T	T	C_het	T
nonsynonymous SNV	SDHAP1	chr3	195690216	T	T	T	C_het	C_het
nonsynonymous SNV	SDHAP1	chr3	195692311	T	T	T	C_het	T
nonsynonymous SNV	SDHAP1	chr3	195692347	G	G	G	A_het	A_het
nonsynonymous SNV	SDHAP2	chr3	195391083	C	C	C	C	G_het
nonsynonymous SNV	SDHAP2	chr3	195391111	T	T	T	C_het	T
nonsynonymous SNV	SDHAP2	chr3	195395402	C	A_het	C	C	C
nonsynonymous SNV	SDHB	chr1	17354297	A	A	G_het	A	A
nonsynonymous SNV	SDHB	chr1	17371358	G	G	A_het	G	G

nonsynonymous SNV	SDK1	chr7	4002309	C	G_het	C	C	C
nonsynonymous SNV	SDK1	chr7	4014039	C	T_het	C	C	C
nonsynonymous SNV	SDR42E1	chr16	82032939	C	C	C	C	T_het
nonsynonymous SNV	SEC14L1	chr17	75205547	G	T_het	G	G	G
nonsynonymous SNV	SEC14L4	chr22	30887836	G	G	G	G	A_hom
nonsynonymous SNV	SEC22B	chr1	145109549	G	T_het	T_het	T_het	T_het
nonsynonymous SNV	SEC22B	chr1	145109583	C	A_het	A_het	A_het	A_het
nonsynonymous SNV	SEC22B	chr1	145109661	G	A_het	A_het	A_het	A_het
nonsynonymous SNV	SEC22B	chr1	145112414	T	C_het	C_het	C_het	C_het
stopgain	SEC22B	chr1	145112420	C	T_het	T_het	T_het	T_het
nonsynonymous SNV	SEC22B	chr1	145112468	A	A	A	C_het	A
nonsynonymous SNV	SEC22B	chr1	145115810	A	G_het	G_het	G_het	G_het
nonsynonymous SNV	SEC24A	chr5	133997007	C	C	C	C	T_het
nonsynonymous SNV	SEC31B	chr10	102247813	A	G_het	A	A	A
nonsynonymous SNV	SEC61A1	chr3	127783771	G	G	A_het	G	G
nonsynonymous SNV	SEC62	chr3	169710385	G	G	G	A_het	G
nonsynonymous SNV	SEC62	chr3	169710614	G	C_het	G	G	G
nonsynonymous SNV	SEL1L3	chr4	25766981	C	C	T_het	C	C
nonsynonymous SNV	SEMA3D	chr7	84651849	G	G	T_het	G	G
nonsynonymous SNV	SEMA6A	chr5	115782398	G	G	G	A_het	G
nonsynonymous SNV	SEMA6A	chr5	115823863	C	C	C	C	T_het
nonsynonymous SNV	SENP5	chr3	196612093	A	C_het	A	A	A
nonsynonymous SNV	SEPT5-GP1BB	chr22	19711445	C	C	C	C	T_het
nonsynonymous SNV	SERPINA12	chr14	94962714	G	G	G	A_het	G
nonsynonymous SNV	SERPINA5	chr14	95057186	G	G	A_het	G	G
nonsynonymous SNV	SERPINA9	chr14	94942578	C	T_het	C	C	C
nonsynonymous SNV	SERPINB3	chr18	61323259	A	A	T_het	A	A
nonsynonymous SNV	SERTM1	chr13	37269282	T	T	T	T	G_hom
nonsynonymous SNV	SETD1A	chr16	30990750	G	G	A_het	G	G
nonsynonymous SNV	SETD1B	chr12	122246194	G	G	A_het	G	G
nonsynonymous SNV	SETD2	chr3	47163843	C	C	C	C	T_het
nonsynonymous SNV	SETD2	chr3	47164507	C	C	T_hom	C	C
nonsynonymous SNV	SETMAR	chr3	4358476	C	C	T_hom	C	C
nonsynonymous SNV	SETX	chr9	135140228	T	T	T	C_hom	T
nonsynonymous SNV	SEZ6L2	chr16	29906715	G	G	T_het	G	G
nonsynonymous SNV	SFMBT2	chr10	7262447	G	G	G	G	A_het
nonsynonymous SNV	SFTPA1	chr10	81371637	T	T	C_het	T	T
nonsynonymous SNV	SFTPA1	chr10	81371729	C	G_het	G_het	G_het	C
nonsynonymous SNV	SFTPC	chr8	22021501	C	G_hom	C	C	C
nonsynonymous SNV	SFXN3	chr10	102798401	G	A_het	G	G	G
nonsynonymous SNV	SGK223	chr8	8233941	G	G	G	T_het	G
nonsynonymous SNV	SGK223	chr8	8234904	C	T_het	C	C	C
nonsynonymous SNV	SGK223	chr8	8234980	G	G	C_het	G	G
nonsynonymous SNV	SGOL1	chr3	20202616	G	G	G	A_hom	G
nonsynonymous SNV	SGOL2	chr2	201436870	C	C	C	T_het	C

nonsynonymous SNV	SGSM3	chr22	40800386	C	C	C	C	T_hom
nonsynonymous SNV	SH2D3A	chr19	6754119	G	G	A_het	G	G
nonsynonymous SNV	SH2D4A	chr8	19171332	C	C	C	G_het	C
stopgain	SH2D4B	chr10	82298154	C	T_het	C	C	C
nonsynonymous SNV	SH2D5	chr1	21050677	G	G	G	A_hom	G
nonsynonymous SNV	SH3BP2	chr4	2835499	G	G	G	G	A_het
nonsynonymous SNV	SH3RF1	chr4	170037661	G	G	A_het	G	G
nonsynonymous SNV	SH3RF2	chr5	145435570	G	G	G	G	A_het
nonsynonymous SNV	SH3TC1	chr4	8229381	C	C	T_het	C	C
nonsynonymous SNV	SHB	chr9	37974759	C	C	C	G_hom	C
nonsynonymous SNV	SHC4	chr15	49118251	T	T	T	T	C_het
nonsynonymous SNV	SHISA9	chr16	13297254	C	C	G_het	C	C
nonsynonymous SNV	SHKBP1	chr19	41095083	C	C	C	C	T_het
nonsynonymous SNV	SHPRH	chr6	146240510	C	C	G_het	C	C
nonsynonymous SNV	SHQ1	chr3	72881614	T	T	T	C_hom	T
nonsynonymous SNV	SHROOM2	chrX	9905260	G	G	T_hom	G	G
nonsynonymous SNV	SHROOM3	chr4	77675652	C	C	T_het	C	C
nonsynonymous SNV	SHROOM3	chr4	77692020	G	G	A_het	G	G
nonsynonymous SNV	SI	chr3	164727159	G	G	A_het	G	G
stopgain	SIGLEC1	chr20	3670249	C	C	C	T_het	C
nonsynonymous SNV	SIGLEC10	chr19	51919263	G	G	G	T_het	G
nonsynonymous SNV	SIGLEC11	chr19	50463670	T	T	T	G_het	G_het
nonsynonymous SNV	SIGLEC14	chr19	52146621	T	T	T	A_het	T
nonsynonymous SNV	SIGLEC7	chr19	51650521	G	G	G	A_het	G
nonsynonymous SNV	SIK1	chr21	44839053	C	C	T_het	C	C
nonsynonymous SNV	SIN3A	chr15	75693078	C	C	T_het	C	C
nonsynonymous SNV	SIPA1L1	chr14	72137937	C	C	T_het	C	C
nonsynonymous SNV	SIPA1L3	chr19	38610252	C	G_het	C	C	C
nonsynonymous SNV	SIRPA	chr20	1895984	C	C	C	C	A_hom
nonsynonymous SNV	SIRPA	chr20	1895990	G	G	G	G	A_hom
nonsynonymous SNV	SIRPB1	chr20	1592120	G	G	G	G	T_hom
nonsynonymous SNV	SIRPB1	chr20	1592139	G	G	G	G	T_hom
nonsynonymous SNV	SIRPB1	chr20	1592141	T	T	T	T	C_hom
nonsynonymous SNV	SIRT3	chr11	233434	C	C	T_het	C	C
nonsynonymous SNV	SIT1	chr9	35649916	C	C	A_het	C	C
nonsynonymous SNV	SIT1	chr9	35649970	C	C	C	T_hom	C
nonsynonymous SNV	SKA3	chr13	21729252	T	C_het	T	T	T
nonsynonymous SNV	SKA3	chr13	21729267	G	A_het	G	G	G
nonsynonymous SNV	SKA3	chr13	21742311	C	T_het	C	C	C
stopgain	SKA3	chr13	21750538	G	A_het	G	G	G
nonsynonymous SNV	SLC10A5	chr8	82606322	G	C_het	G	G	G
nonsynonymous SNV	SLC11A1	chr2	219258856	T	T	C_het	T	T
nonsynonymous SNV	SLC11A1	chr2	219259458	G	G	G	G	A_het
nonsynonymous SNV	SLC12A7	chr5	1085534	C	C	A_het	C	C
nonsynonymous SNV	SLC13A1	chr7	122789544	A	A	A	A	G_hom

nonsynonymous SNV	SLC16A8	chr22	38477137	C	C	C	A_hom	C
nonsynonymous SNV	SLC18A1	chr8	20003282	C	C	C	C	T_hom
nonsynonymous SNV	SLC18A1	chr8	20004798	G	G	G	A_het	G
nonsynonymous SNV	SLC20A1	chr2	113414755	G	G	A_het	G	G
stopgain	SLC22A10	chr11	63064782	C	C	C	T_hom	C
nonsynonymous SNV	SLC22A10	chr11	63064845	G	G	G	A_hom	G
nonsynonymous SNV	SLC22A10	chr11	63069947	G	G	G	A_hom	G
nonsynonymous SNV	SLC22A10	chr11	63071595	G	G	G	A_hom	G
nonsynonymous SNV	SLC22A10	chr11	63072327	C	C	C	T_hom	C
nonsynonymous SNV	SLC22A17	chr14	23815957	C	T_het	C	C	C
nonsynonymous SNV	SLC22A25	chr11	62933726	C	C	G_het	C	C
nonsynonymous SNV	SLC22A25	chr11	62984811	A	A	A	G_hom	A
nonsynonymous SNV	SLC22A7	chr6	43270473	A	A	A	A	G_het
nonsynonymous SNV	SLC22A9	chr11	63137769	T	T	T	C_hom	T
nonsynonymous SNV	SLC22A9	chr11	63143238	G	C_het	G	G	G
nonsynonymous SNV	SLC23A3	chr2	220032977	G	G	G	A_het	G
nonsynonymous SNV	SLC24A6	chr12	113756808	G	G	A_het	G	G
nonsynonymous SNV	SLC24A6	chr12	113758490	C	C	T_het	C	C
nonsynonymous SNV	SLC25A46	chr5	110074872	G	G	G	T_het	G
nonsynonymous SNV	SLC25A5	chrX	118603729	G	G	A_het	A_het	A_het
nonsynonymous SNV	SLC25A5	chrX	118603742	A	A	C_het	C_het	C_het
nonsynonymous SNV	SLC25A5	chrX	118603747	A	A	T_het	T_het	T_het
nonsynonymous SNV	SLC25A5	chrX	118603864	G	G	A_het	G	G
nonsynonymous SNV	SLC25A5	chrX	118603925	G	G	G	A_het	G
nonsynonymous SNV	SLC25A5	chrX	118603958	G	G	A_het	A_het	G
nonsynonymous SNV	SLC25A5	chrX	118603984	G	G	A_het	A_het	G
nonsynonymous SNV	SLC25A5	chrX	118604006	A	G_het	G_het	G_het	A
nonsynonymous SNV	SLC25A5	chrX	118604014	G	G	A_het	A_het	G
nonsynonymous SNV	SLC25A5	chrX	118604030	T	T	C_het	T	T
nonsynonymous SNV	SLC25A5	chrX	118604399	C	C	C	G_het	C
nonsynonymous SNV	SLC25A5	chrX	118604416	A	A	A	G_het	A
nonsynonymous SNV	SLC25A5	chrX	118604428	T	T	T	C_het	T
nonsynonymous SNV	SLC26A1	chr4	983133	C	C	T_het	C	C
nonsynonymous SNV	SLC26A2	chr5	149360877	T	C_hom	C_hom	C_hom	C_hom
nonsynonymous SNV	SLC26A3	chr7	107416988	A	C_het	A	A	A
nonsynonymous SNV	SLC26A5	chr7	103033496	T	C_het	T	T	T
nonsynonymous SNV	SLC27A3	chr1	153750719	G	G	G	G	A_het
nonsynonymous SNV	SLC28A2	chr15	45559774	T	T	T	C_het	T
nonsynonymous SNV	SLC29A1	chr6	44197822	G	G	A_het	G	G
nonsynonymous SNV	SLC29A3	chr10	73111423	G	G	G	G	T_het
stopgain	SLC2A11	chr22	24225243	C	C	C	C	T_het
nonsynonymous SNV	SLC2A3	chr12	8074055	T	C_het	T	T	T
nonsynonymous SNV	SLC2A6	chr9	136340572	C	C	C	C	A_hom
nonsynonymous SNV	SLC2A6	chr9	136342228	C	C	C	C	G_hom
nonsynonymous SNV	SLC30A1	chr1	211751667	G	C_het	G	G	G

nonsynonymous SNV	SLC30A5	chr5	68414334	G	G	A_het	G	G
nonsynonymous SNV	SLC34A3	chr9	140127093	G	G	A_het	G	G
nonsynonymous SNV	SLC35C1	chr11	45832845	C	C	T_het	C	C
nonsynonymous SNV	SLC35D2	chr9	99130582	G	G	A_het	G	G
nonsynonymous SNV	SLC35E2B	chr1	1597199	A	A	A	A	G_het
nonsynonymous SNV	SLC35E2B	chr1	1599888	A	A	G_het	G_het	G_het
nonsynonymous SNV	SLC35G1	chr10	95660784	C	C	C	C	T_het
nonsynonymous SNV	SLC35G5	chr8	11189132	C	C	G_het	C	C
nonsynonymous SNV	SLC35G5	chr8	11189142	G	G	A_het	G	G
nonsynonymous SNV	SLC35G5	chr8	11189184	C	C	T_het	C	C
nonsynonymous SNV	SLC35G5	chr8	11189529	T	T	T	T	C_het
nonsynonymous SNV	SLC35G5	chr8	11189591	C	C	C	C	T_het
nonsynonymous SNV	SLC35G6	chr17	7386217	T	T	T	T	C_het
nonsynonymous SNV	SLC36A2	chr5	150723155	C	A_het	C	C	C
nonsynonymous SNV	SLC36A3	chr5	150657198	C	C	C	T_het	C
nonsynonymous SNV	SLC39A11	chr17	71080981	C	C	C	T_hom	C
nonsynonymous SNV	SLC39A6	chr18	33694152	C	C	C	C	T_het
nonsynonymous SNV	SLC43A2	chr17	1494129	G	G	G	A_hom	G
nonsynonymous SNV	SLC44A3	chr1	95305504	C	C	C	C	A_het
nonsynonymous SNV	SLC44A3	chr1	95360387	G	G	T_het	G	G
nonsynonymous SNV	SLC44A5	chr1	75677192	C	C	T_het	C	C
nonsynonymous SNV	SLC46A1	chr17	26726686	G	G	A_het	G	G
nonsynonymous SNV	SLC47A1	chr17	19437280	G	G	G	G	T_hom
nonsynonymous SNV	SLC4A5	chr2	74462286	C	C	T_het	C	C
nonsynonymous SNV	SLC5A4	chr22	32627048	C	T_het	C	C	C
nonsynonymous SNV	SLC5A6	chr2	27424603	C	C	C	T_het	C
nonsynonymous SNV	SLC6A3	chr5	1409177	C	C	T_het	C	C
nonsynonymous SNV	SLC6A7	chr5	149584465	T	T	T	T	C_het
nonsynonymous SNV	SLC7A10	chr19	33703438	G	G	T_het	G	G
nonsynonymous SNV	SLC7A2	chr8	17419551	G	G	G	A_het	G
nonsynonymous SNV	SLC7A5P2	chr16	21531133	C	G_het	C	C	C
nonsynonymous SNV	SLC7A5P2	chr16	21531154	G	C_het	G	G	G
nonsynonymous SNV	SLC7A5P2	chr16	21531457	T	C_het	T	T	T
nonsynonymous SNV	SLC7A5P2	chr16	21531473	G	A_het	G	G	G
nonsynonymous SNV	SLC7A5P2	chr16	21531494	T	C_het	C_het	C_het	C_hom
nonsynonymous SNV	SLC7A5P2	chr16	21531609	C	T_het	T_het	T_het	T_het
nonsynonymous SNV	SLC8A3	chr14	70530566	C	C	C	C	T_het
nonsynonymous SNV	SLC9A1	chr1	27427700	C	C	T_het	C	C
nonsynonymous SNV	SLC9A3	chr5	475104	A	G_hom	G_hom	G_hom	G_hom
nonsynonymous SNV	SLC9A3R1	chr17	72759545	G	G	A_het	G	G
nonsynonymous SNV	SLC9A3R2	chr16	2083386	C	C	C	T_het	C
nonsynonymous SNV	SLC9B1	chr4	103822404	G	A_het	A_het	A_het	A_het
stopgain	SLC9B1	chr4	103832611	G	G	A_het	A_het	A_het
nonsynonymous SNV	SLC9B1	chr4	103832650	T	A_het	A_het	T	T
nonsynonymous SNV	SLC9B1	chr4	103832665	T	T	C_het	T	T

nonsynonymous SNV	SLCO1B3	chr12	21033951	T	A_het	T	T	T
nonsynonymous SNV	SLCO1C1	chr12	20876168	C	T_het	C	C	C
nonsynonymous SNV	SLIT1	chr10	98791402	G	G	G	A_het	G
nonsynonymous SNV	SLIT2	chr4	20597337	G	T_het	G	G	G
nonsynonymous SNV	SLIT3	chr5	168096793	T	T	T	C_het	T
nonsynonymous SNV	SLITRK6	chr13	86368225	G	G	A_het	G	G
nonsynonymous SNV	SLK	chr10	105768046	C	C	C	C	T_het
nonsynonymous SNV	SLU7	chr5	159835658	A	G_het	G_hom	G_het	G_het
nonsynonymous SNV	SLX4	chr16	3644511	C	C	C	G_het	C
nonsynonymous SNV	SMAD4	chr18	48575096	G	G	A_het	G	G
stopgain	SMARCA1	chrX	128602861	G	G	A_hom	G	G
nonsynonymous SNV	SMARCA4	chr19	11097240	C	C	T_het	C	C
nonsynonymous SNV	SMARCA4	chr19	11143994	G	G	A_het	G	G
nonsynonymous SNV	SMARCAD1	chr4	95173928	C	C	T_het	C	C
nonsynonymous SNV	SMARCC2	chr12	56563651	C	C	C	C	A_het
nonsynonymous SNV	SMARCD3	chr7	150938544	C	C	A_het	C	C
nonsynonymous SNV	SMC1A	chrX	53423450	C	T_het	C	C	C
nonsynonymous SNV	SMC2	chr9	106889571	C	C	T_het	C	C
nonsynonymous SNV	SMG9	chr19	44237834	C	C	C	T_het	C
nonsynonymous SNV	SMO	chr7	128829102	C	C	C	C	T_het
nonsynonymous SNV	SMOC1	chr14	70478230	G	G	G	A_het	G
nonsynonymous SNV	SMPD1	chr11	6412617	G	G	C_het	G	G
nonsynonymous SNV	SMYD5	chr2	73448969	C	C	T_het	C	C
nonsynonymous SNV	SNAPC4	chr9	139287131	T	T	C_het	T	T
nonsynonymous SNV	SND1	chr7	127729597	G	T_het	G	G	G
nonsynonymous SNV	SNED1	chr2	242009364	G	G	A_het	G	G
nonsynonymous SNV	SNRNP35	chr12	123950820	G	G	T_het	G	G
nonsynonymous SNV	SNRNP48	chr6	7606299	G	G	G	G	A_het
nonsynonymous SNV	SNRPC	chr6	34725658	G	G	G	T_hom	T_het
nonsynonymous SNV	SNTB2	chr16	69333668	G	G	T_het	G	G
nonsynonymous SNV	SNUPN	chr15	75901899	C	C	C	G_het	C
nonsynonymous SNV	SNX1	chr15	64388226	G	G	G	G	A_het
nonsynonymous SNV	SNX14	chr6	86277300	A	A	A	A	G_het
nonsynonymous SNV	SNX17	chr2	27594142	A	A	C_het	A	A
nonsynonymous SNV	SNX19	chr11	130780225	C	A_hom	A_hom	A_hom	A_hom
nonsynonymous SNV	SNX20	chr16	50707477	C	C	T_het	C	C
nonsynonymous SNV	SNX33	chr15	75949311	G	G	A_het	G	G
nonsynonymous SNV	SNX7	chr1	99164391	G	G	A_het	G	G
nonsynonymous SNV	SNX8	chr7	2317934	G	G	G	A_het	G
nonsynonymous SNV	SOGA1	chr20	35422415	C	C	T_het	C	C
nonsynonymous SNV	SOGA1	chr20	35423028	C	C	T_het	C	C
nonsynonymous SNV	SOGA1	chr20	35425351	C	C	T_het	C	C
nonsynonymous SNV	SOGA2	chr18	8786120	G	G	G	G	C_het
nonsynonymous SNV	SOGA2	chr18	8786124	T	T	T	T	C_het
nonsynonymous SNV	SOGA2	chr18	8786130	T	T	T	T	C_het

nonsynonymous SNV	SOGA2	chr18	8809528	C	C	C	T_hom	C
nonsynonymous SNV	SOGA3	chr6	127767954	G	A_het	G	G	G
nonsynonymous SNV	SOHLH2	chr13	36744720	C	C	T_het	C	C
nonsynonymous SNV	SOLH	chr16	602700	G	A_het	G	G	G
nonsynonymous SNV	SORBS1	chr10	97111100	C	C	T_het	C	C
nonsynonymous SNV	SORBS2	chr4	186583380	T	C_het	T	T	T
nonsynonymous SNV	SORCS2	chr4	7666180	C	A_het	A_het	C	C
nonsynonymous SNV	SORL1	chr11	121348833	G	G	A_het	G	G
nonsynonymous SNV	SORL1	chr11	121393406	G	G	A_het	G	G
nonsynonymous SNV	SORL1	chr11	121490502	A	A	A	C_hom	A
nonsynonymous SNV	SOS2	chr14	50623669	G	G	G	A_het	G
nonsynonymous SNV	SOSTDC1	chr7	16502390	T	C_het	T	T	T
nonsynonymous SNV	SOWAHA	chr5	132149684	G	C_hom	C_hom	C_hom	C_hom
nonsynonymous SNV	SOX12	chr20	307296	A	A	A	G_het	A
nonsynonymous SNV	SOX18	chr20	62680627	G	G	C_het	G	G
nonsynonymous SNV	SOX7	chr8	10584033	G	G	A_het	G	G
nonsynonymous SNV	SOX8	chr16	1035099	C	C	C	A_het	C
nonsynonymous SNV	SP110	chr2	231042873	C	T_het	C	C	C
nonsynonymous SNV	SP140	chr2	231120209	G	G	G	C_het	G
nonsynonymous SNV	SP5	chr2	171572937	A	T_het	A	A	A
nonsynonymous SNV	SPAG1	chr8	101252681	G	G	G	A_het	G
nonsynonymous SNV	SPAG11A	chr8	7718180	T	T	T	C_het	T
nonsynonymous SNV	SPAG11B	chr8	7308386	T	C_het	T	C_het	C_het
nonsynonymous SNV	SPANX-L	chrX	140714249	G	G	A_hom	G	G
nonsynonymous SNV	SPARCL1	chr4	88415093	G	G	G	G	T_het
nonsynonymous SNV	SPATA31A6	chr9	43627444	G	G	G	G	A_hom
nonsynonymous SNV	SPATA31C2	chr9	90744936	T	T	C_het	T	T
nonsynonymous SNV	SPATA31D1	chr9	84608117	G	G	G	T_hom	G
nonsynonymous SNV	SPATA31D1	chr9	84608722	G	G	G	G	A_het
nonsynonymous SNV	SPATA31D5P	chr9	84530962	T	T	G_het	T	T
nonsynonymous SNV	SPATC1	chr8	145095062	G	A_het	G	G	G
nonsynonymous SNV	SPDL1	chr5	169028316	C	C	A_het	C	C
nonsynonymous SNV	SPDYE4	chr17	8660597	G	G	G	A_hom	G
nonsynonymous SNV	SPECC1	chr17	20000032	G	A_het	G	G	G
nonsynonymous SNV	SPEF2	chr5	35709095	C	T_het	T_het	T_het	T_het
nonsynonymous SNV	SPEF2	chr5	35709184	G	C_het	C_het	C_het	C_het
nonsynonymous SNV	SPEF2	chr5	35753763	G	G	G	G	A_het
nonsynonymous SNV	SPEG	chr2	220349387	G	G	G	C_het	G
nonsynonymous SNV	SPERT	chr13	46288080	G	G	A_het	G	G
nonsynonymous SNV	SPG11	chr15	44858159	T	T	T	T	C_het
nonsynonymous SNV	SPHK1	chr17	74383082	G	G	G	G	A_hom
nonsynonymous SNV	SPINK1	chr5	147207616	G	A_het	G	G	G
nonsynonymous SNV	SPINK5	chr5	147475388	C	T_het	C	C	C
nonsynonymous SNV	SPINK6	chr5	147593515	C	C	T_het	C	C
nonsynonymous SNV	SPINK7	chr5	147693780	G	G	A_het	G	G

nonsynonymous SNV	SPINT3	chr20	44141412	G	G	A_het	G	G
nonsynonymous SNV	SPN	chr16	29675665	A	A	G_het	A	A
nonsynonymous SNV	SPNS2	chr17	4434050	G	G	G	A_hom	G
nonsynonymous SNV	SPOCD1	chr1	32280756	G	G	T_het	G	G
nonsynonymous SNV	SPRED3	chr19	38886714	G	G	A_het	G	G
nonsynonymous SNV	SPRR3	chr1	152975810	G	G	G	A_hom	G
nonsynonymous SNV	SPRYD3	chr12	53470975	G	G	A_het	G	G
nonsynonymous SNV	SPTAN1	chr9	131355267	G	G	A_het	G	G
nonsynonymous SNV	SPTBN2	chr11	66457681	C	T_het	C	C	C
nonsynonymous SNV	SPTBN4	chr19	41075583	T	T	C_het	T	T
nonsynonymous SNV	SPTBN5	chr15	42149595	C	C	T_het	C	C
nonsynonymous SNV	SQSTM1	chr5	179260099	G	C_het	G	G	G
nonsynonymous SNV	SRCAP	chr16	30715397	G	G	G	G	A_hom
nonsynonymous SNV	SREBF1	chr17	17715971	G	G	G	G	C_hom
nonsynonymous SNV	SRGAP1	chr12	64474288	A	A	A	G_het	A
nonsynonymous SNV	SRGAP2B	chr1	144014051	G	A_het	A_het	A_het	A_het
nonsynonymous SNV	SRGAP2B	chr1	144014068	T	C_het	C_het	C_het	C_het
nonsynonymous SNV	SRGAP2D	chr1	121116688	A	G_het	G_hom	A	G_het
nonsynonymous SNV	SRGAP3	chr3	9027245	C	A_hom	C	C	C
nonsynonymous SNV	SRGAP3	chr3	9027594	G	G	A_hom	G	G
nonsynonymous SNV	SSC5D	chr19	56009424	G	A_het	G	G	G
nonsynonymous SNV	SSC5D	chr19	56011470	A	A	A	A	C_het
nonsynonymous SNV	SSC5D	chr19	56012166	G	A_het	G	G	G
nonsynonymous SNV	SSC5D	chr19	56015533	A	A	A	G_het	A
nonsynonymous SNV	SSFA2	chr2	182794297	G	A_het	G	G	G
nonsynonymous SNV	SSH2	chr17	27994186	T	T	C_het	T	T
nonsynonymous SNV	SSH2	chr17	28004744	C	C	C	C	G_hom
nonsynonymous SNV	SSPN	chr12	26383678	C	C	C	T_het	C
stopgain	SSPO	chr7	149503834	G	T_het	G	G	G
nonsynonymous SNV	SSTR5-AS1	chr16	1115849	C	C	A_het	C	C
nonsynonymous SNV	ST14	chr11	130079424	C	C	C	T_hom	C
nonsynonymous SNV	ST18	chr8	53044681	G	G	T_het	G	G
nonsynonymous SNV	ST20-MTHFS	chr15	80137689	G	G	G	G	A_het
nonsynonymous SNV	ST8SIA2	chr15	93007607	G	G	A_het	G	G
nonsynonymous SNV	ST8SIA5	chr18	44266233	C	C	C	T_hom	C
nonsynonymous SNV	STAB1	chr3	52548195	G	G	G	G	A_hom
nonsynonymous SNV	STAC	chr3	36570440	G	G	G	A_hom	G
nonsynonymous SNV	STAM	chr10	17730057	C	C	G_het	C	C
nonsynonymous SNV	STAP2	chr19	4338720	G	G	A_het	G	G
nonsynonymous SNV	STARD3	chr17	37817251	G	G	G	G	A_hom
stopgain	STARD6	chr18	51858156	A	A	A	C_hom	A
nonsynonymous SNV	STARD8	chrX	67938269	G	G	A_hom	G	G
nonsynonymous SNV	STARD8	chrX	67940130	G	G	G	G	T_het
nonsynonymous SNV	STARD9	chr15	42981874	G	G	G	A_het	G
nonsynonymous SNV	STAT6	chr12	57500533	C	C	T_het	C	C

stopgain	STATH	chr4	70866636	C	C	C	C	A_het
nonsynonymous SNV	STEAP1	chr7	89790245	T	T	T	C_het	T
nonsynonymous SNV	STEAP1B	chr7	22533452	C	C	T_het	T_het	C
nonsynonymous SNV	STIM2	chr4	27019452	C	T_het	C	C	C
nonsynonymous SNV	STK16	chr2	220112574	C	C	C	C	T_het
nonsynonymous SNV	STK17A	chr7	43647938	G	G	G	A_het	G
nonsynonymous SNV	STK25	chr2	242438129	G	G	A_het	G	G
nonsynonymous SNV	STK3	chr8	99779588	C	C	T_het	C	C
nonsynonymous SNV	STK31	chr7	23757162	G	C_hom	C_het	C_hom	C_hom
nonsynonymous SNV	STK33	chr11	8435085	A	A	G_het	A	A
nonsynonymous SNV	STOM	chr9	124116908	C	C	C	C	T_hom
nonsynonymous SNV	STPG2	chr4	99055502	G	G	A_het	G	G
nonsynonymous SNV	STRN4	chr19	47226142	G	G	T_het	G	G
nonsynonymous SNV	STXBP2	chr19	7705432	G	G	G	T_het	G
nonsynonymous SNV	STXBP2	chr19	7705825	G	G	G	A_het	G
nonsynonymous SNV	STXBP2	chr19	7706056	G	A_het	G	G	G
nonsynonymous SNV	STXBP2	chr19	7711222	G	G	G	G	A_het
nonsynonymous SNV	SUCO	chr1	172558339	G	G	G	G	T_het
nonsynonymous SNV	SULF1	chr8	70515510	G	G	T_het	G	G
nonsynonymous SNV	SULT1A1	chr16	28617485	C	T_hom	T_hom	T_hom	T_hom
nonsynonymous SNV	SULT1A1	chr16	28617514	C	T_hom	T_het	T_het	C
nonsynonymous SNV	SULT1C2	chr2	108910718	T	T	C_het	T	T
nonsynonymous SNV	SUMO3	chr21	46233868	T	C_het	T	T	T
nonsynonymous SNV	SUN1	chr7	889592	G	G	G	A_het	G
nonsynonymous SNV	SUN2	chr22	39134681	G	G	A_het	G	G
nonsynonymous SNV	SUPT3H	chr6	44921123	T	T	T	G_hom	T
nonsynonymous SNV	SUPT5H	chr19	39963065	A	A	G_het	A	A
nonsynonymous SNV	SUPT5H	chr19	39963546	G	G	A_het	G	G
nonsynonymous SNV	SUPT6H	chr17	27002049	G	G	A_het	G	G
nonsynonymous SNV	SUPT7L	chr2	27880441	T	T	T	T	A_het
nonsynonymous SNV	SURF6	chr9	136199109	G	G	T_het	G	G
nonsynonymous SNV	SUSD2	chr22	24579049	G	A_het	A_het	A_het	A_het
nonsynonymous SNV	SUSD2	chr22	24579157	G	T_het	T_het	T_het	T_het
nonsynonymous SNV	SUSD4	chr1	223402606	G	G	G	G	C_het
nonsynonymous SNV	SV2C	chr5	75427766	C	C	C	C	A_het
nonsynonymous SNV	SVIL	chr10	29783908	A	G_het	G_het	A	A
nonsynonymous SNV	SVIL	chr10	29784072	G	G	C_hom	G	G
nonsynonymous SNV	SWT1	chr1	185135747	C	C	C	A_hom	C
nonsynonymous SNV	SYCE1	chr10	135369152	C	C	C	T_het	C
nonsynonymous SNV	SYCE1L	chr16	77242418	C	C	T_het	C	C
nonsynonymous SNV	SYCE3	chr22	50989742	C	C	T_het	C	C
nonsynonymous SNV	SYCP2	chr20	58490557	G	G	G	G	A_het
nonsynonymous SNV	SYF2	chr1	25555533	T	T	T	T	C_het
nonsynonymous SNV	SYN2	chr3	12046125	G	C_hom	G	C_hom	G
nonsynonymous SNV	SYN2	chr3	12046131	A	C_hom	A	C_hom	A

nonsynonymous SNV	SYNC	chr1	33160912	G	G	G	A_hom	G
nonsynonymous SNV	SYNE1	chr6	152473122	C	C	T_het	C	C
nonsynonymous SNV	SYNE1	chr6	152501416	C	T_hom	C	C	C
nonsynonymous SNV	SYNE1	chr6	152615110	T	T	G_het	T	T
nonsynonymous SNV	SYNE1	chr6	152651557	G	G	A_het	G	G
nonsynonymous SNV	SYNE1	chr6	152734582	A	A	A	C_hom	A
nonsynonymous SNV	SYNE3	chr14	95934159	G	G	A_het	G	G
nonsynonymous SNV	SYNJ2	chr6	158516704	C	C	T_het	C	C
nonsynonymous SNV	SYNM	chr15	99645622	G	G	C_het	G	G
nonsynonymous SNV	SYNPO	chr5	150028685	G	A_het	G	G	G
nonsynonymous SNV	SYNPO2	chr4	119951813	C	C	C	A_hom	C
nonsynonymous SNV	SYNPO2	chr4	119978761	A	G_het	A	A	A
nonsynonymous SNV	SYT15	chr10	46965017	C	C	T_het	C	C
nonsynonymous SNV	SYT15	chr10	46965018	C	G_het	C	G_het	G_het
nonsynonymous SNV	SYT5	chr19	55686297	A	A	G_het	A	A
nonsynonymous SNV	SYT5	chr19	55690398	C	C	C	C	A_het
nonsynonymous SNV	SYT7	chr11	61313525	C	T_het	C	C	C
nonsynonymous SNV	SYTL2	chr11	85437499	T	C_het	T	T	T
nonsynonymous SNV	SZT2	chr1	43882221	C	C	T_het	C	C
nonsynonymous SNV	T	chr6	166572042	C	C	T_het	C	C
nonsynonymous SNV	TAAR8	chr6	132874277	G	G	G	T_hom	G
nonsynonymous SNV	TACC2	chr10	123845826	G	G	G	A_het	G
nonsynonymous SNV	TAF1A	chr1	222750877	G	G	G	G	A_het
nonsynonymous SNV	TAL1	chr1	47685559	G	G	G	G	A_het
nonsynonymous SNV	TAL1	chr1	47691350	C	C	C	T_hom	C
nonsynonymous SNV	TANC2	chr17	61176602	G	G	G	G	A_hom
nonsynonymous SNV	TANC2	chr17	61476264	G	G	T_het	G	G
nonsynonymous SNV	TANC2	chr17	61489003	C	C	T_het	C	C
nonsynonymous SNV	TANGO2	chr22	20043504	G	G	A_het	G	G
nonsynonymous SNV	TANGO6	chr16	68961901	C	C	C	G_het	C
stopgain	TAOK3	chr12	118693325	G	G	T_het	G	G
nonsynonymous SNV	TARBP1	chr1	234564951	A	A	T_het	A	A
stoploss	TAS2R19	chr12	11174271	T	C_het	C_het	C_het	C_het
nonsynonymous SNV	TAS2R19	chr12	11174282	T	C_het	C_het	C_het	C_het
nonsynonymous SNV	TAS2R19	chr12	11174286	C	A_het	A_het	A_het	A_het
nonsynonymous SNV	TAS2R19	chr12	11174302	A	G_het	G_het	A	G_het
nonsynonymous SNV	TAS2R19	chr12	11174327	C	T_het	T_het	T_het	T_het
nonsynonymous SNV	TAS2R19	chr12	11174372	C	G_het	G_het	G_het	G_het
nonsynonymous SNV	TAS2R19	chr12	11174380	C	T_het	T_het	T_het	T_het
nonsynonymous SNV	TAS2R19	chr12	11174390	G	A_het	A_het	A_het	A_het
nonsynonymous SNV	TAS2R19	chr12	11174397	T	A_het	A_het	A_het	A_het
nonsynonymous SNV	TAS2R19	chr12	11174416	A	G_het	G_het	G_het	A
nonsynonymous SNV	TAS2R19	chr12	11174467	A	A	C_het	A	A
nonsynonymous SNV	TAS2R19	chr12	11174476	G	G	A_het	G	G
nonsynonymous SNV	TAS2R30	chr12	11286249	T	C_het	C_het	T	T

nonsynonymous SNV	TAS2R30	chr12	11286276	G	C_het	C_het	G	G
nonsynonymous SNV	TAS2R30	chr12	11286282	G	A_het	A_het	G	G
nonsynonymous SNV	TAS2R30	chr12	11286289	A	T_het	T_het	A	A
nonsynonymous SNV	TAS2R30	chr12	11286713	A	A	G_het	A	A
nonsynonymous SNV	TAS2R30	chr12	11286746	A	A	G_het	A	G_het
nonsynonymous SNV	TAS2R30	chr12	11286797	A	A	G_het	A	G_het
nonsynonymous SNV	TAS2R30	chr12	11286807	T	T	C_het	T	C_het
stopgain	TAS2R31	chr12	11183035	C	C	T_het	C	T_het
nonsynonymous SNV	TAS2R31	chr12	11183042	C	G_het	C	C	G_het
nonsynonymous SNV	TAS2R31	chr12	11183046	C	T_het	T_het	C	T_het
nonsynonymous SNV	TAS2R31	chr12	11183052	G	A_het	A_het	G	A_het
nonsynonymous SNV	TAS2R31	chr12	11183066	A	T_het	T_het	A	T_het
nonsynonymous SNV	TAS2R31	chr12	11183121	G	G	A_het	G	G
nonsynonymous SNV	TAS2R31	chr12	11183123	T	T	C_het	T	T
nonsynonymous SNV	TAS2R31	chr12	11183475	G	G	G	G	A_het
nonsynonymous SNV	TAS2R31	chr12	11183484	C	C	C	C	G_het
nonsynonymous SNV	TAS2R31	chr12	11183485	T	T	T	T	A_het
nonsynonymous SNV	TAS2R31	chr12	11183496	T	C_het	T	T	C_het
nonsynonymous SNV	TAS2R31	chr12	11183506	T	A_het	T	T	A_het
nonsynonymous SNV	TAS2R31	chr12	11183541	T	T	C_het	T	C_het
nonsynonymous SNV	TAS2R31	chr12	11183576	T	C_het	C_het	T	C_het
nonsynonymous SNV	TAS2R31	chr12	11183642	A	G_het	G_het	A	G_het
nonsynonymous SNV	TAS2R31	chr12	11183676	C	T_het	T_het	C	T_het
nonsynonymous SNV	TAS2R31	chr12	11183697	C	A_het	A_het	C	A_het
nonsynonymous SNV	TAS2R31	chr12	11183708	T	C_het	T	T	C_het
nonsynonymous SNV	TAS2R31	chr12	11183722	A	C_het	C_het	A	C_het
nonsynonymous SNV	TAS2R31	chr12	11183793	G	C_het	G	G	G
nonsynonymous SNV	TAS2R43	chr12	11244015	G	A_het	G	G	G
nonsynonymous SNV	TAS2R43	chr12	11244017	T	C_het	T	T	T
nonsynonymous SNV	TAS2R43	chr12	11244027	T	C_het	T	T	T
nonsynonymous SNV	TAS2R43	chr12	11244036	T	G_het	T	T	T
nonsynonymous SNV	TAS2R43	chr12	11244091	C	T_het	C	C	T_het
nonsynonymous SNV	TAS2R43	chr12	11244096	T	C_het	T	T	C_het
nonsynonymous SNV	TAS2R43	chr12	11244102	G	C_het	C_hom	G	C_het
nonsynonymous SNV	TAS2R43	chr12	11244117	A	A	G_hom	A	A
nonsynonymous SNV	TAS2R43	chr12	11244126	G	G	A_hom	G	G
nonsynonymous SNV	TAS2R43	chr12	11244149	G	A_het	A_hom	G	A_het
nonsynonymous SNV	TAS2R43	chr12	11244199	C	C	C	C	G_het
nonsynonymous SNV	TAS2R43	chr12	11244356	A	T_het	A	A	A
nonsynonymous SNV	TAS2R43	chr12	11244369	G	A_het	G	G	G
nonsynonymous SNV	TAS2R43	chr12	11244378	C	G_het	C	C	G_het
nonsynonymous SNV	TAS2R43	chr12	11244390	T	C_het	T	T	T
nonsynonymous SNV	TAS2R43	chr12	11244435	T	C_het	T	T	T
nonsynonymous SNV	TAS2R43	chr12	11244470	T	C_het	T	T	T
nonsynonymous SNV	TAS2R43	chr12	11244687	G	C_het	G	G	C_het

nonsynonymous SNV	TAS2R43	chr12	11244721	G	C_het	G	G	C_het
nonsynonymous SNV	TAS2R43	chr12	11244723	A	C_het	A	A	C_het
nonsynonymous SNV	TAS2R43	chr12	11244731	A	G_het	A	A	G_het
nonsynonymous SNV	TAS2R43	chr12	11244797	C	A_het	A_hom	C	C
nonsynonymous SNV	TAS2R46	chr12	11214074	T	C_het	C_het	T	C_het
nonsynonymous SNV	TAS2R46	chr12	11214080	G	A_het	A_het	G	A_het
nonsynonymous SNV	TAS2R46	chr12	11214082	T	C_het	C_het	T	C_het
nonsynonymous SNV	TAS2R46	chr12	11214091	G	C_het	C_het	G	C_het
nonsynonymous SNV	TAS2R46	chr12	11214101	C	G_het	G_het	C	G_het
nonsynonymous SNV	TAS2R46	chr12	11214133	C	T_het	T_het	C	T_het
nonsynonymous SNV	TAS2R46	chr12	11214137	C	C	T_het	C	T_het
nonsynonymous SNV	TAS2R46	chr12	11214153	C	C	T_het	C	T_het
nonsynonymous SNV	TAS2R46	chr12	11214161	T	T	C_het	T	C_het
nonsynonymous SNV	TAS2R46	chr12	11214167	G	G	C_het	G	C_het
nonsynonymous SNV	TAS2R46	chr12	11214299	T	C_het	C_het	T	T
nonsynonymous SNV	TAS2R46	chr12	11214326	G	C_het	C_het	G	C_het
nonsynonymous SNV	TAS2R46	chr12	11214351	G	C_het	C_het	G	C_het
nonsynonymous SNV	TAS2R46	chr12	11214368	T	C_het	C_het	T	C_het
nonsynonymous SNV	TAS2R46	chr12	11214386	T	G_het	G_het	T	G_het
nonsynonymous SNV	TAS2R46	chr12	11214437	T	T	C_het	T	T
nonsynonymous SNV	TAS2R46	chr12	11214455	T	T	C_het	T	T
nonsynonymous SNV	TAS2R46	chr12	11214472	A	A	G_het	A	A
nonsynonymous SNV	TAS2R46	chr12	11214498	T	T	C_het	T	T
nonsynonymous SNV	TAS2R50	chr12	11139305	G	G	G	G	A_het
nonsynonymous SNV	TAS2R7	chr12	10954382	G	G	G	A_het	G
nonsynonymous SNV	TBC1D1	chr4	38053599	C	C	C	C	T_het
nonsynonymous SNV	TBC1D28	chr17	18542519	T	G_het	T	T	G_hom
nonsynonymous SNV	TBC1D2B	chr15	78310269	T	T	T	T	C_het
nonsynonymous SNV	TBC1D3	chr17	36293029	C	C	A_hom	C	C
nonsynonymous SNV	TBC1D3	chr17	36339597	G	G	T_het	G	T_hom
stoploss	TBC1D3P2	chr17	60345508	A	A	G_het	A	A
stopgain	TBC1D8	chr2	101644564	G	T_hom	G	G	G
nonsynonymous SNV	TBCD	chr17	80887386	C	C	T_het	C	C
nonsynonymous SNV	TBCK	chr4	107163646	G	G	A_het	G	G
nonsynonymous SNV	TBCK	chr4	107163667	T	A_het	T	T	T
nonsynonymous SNV	TBL3	chr16	2025587	C	T_het	C	C	C
nonsynonymous SNV	TBR1	chr2	162273555	C	C	G_het	C	C
nonsynonymous SNV	TBX3	chr12	115117353	G	T_het	G	G	G
nonsynonymous SNV	TBXAS1	chr7	139572123	G	G	G	A_het	G
nonsynonymous SNV	TCEA3	chr1	23743815	G	G	A_het	G	G
nonsynonymous SNV	TCEANC2	chr1	54562010	C	G_hom	C	C	C
nonsynonymous SNV	TCF20	chr22	42607071	G	G	G	A_hom	G
nonsynonymous SNV	TCF7L1	chr2	85533637	G	G	G	C_het	G
nonsynonymous SNV	TCF7L2	chr10	114925501	G	G	A_het	G	G
nonsynonymous SNV	TCHH	chr1	152085253	T	T	T	T	G_het

nonsynonymous SNV	TCP10	chr6	167786750	C	C	A_het	A_het	C
nonsynonymous SNV	TCP10L	chr21	33949136	G	G	G	A_het	G
nonsynonymous SNV	TCP10L2	chr6	167592524	T	T	C_hom	C_hom	C_hom
nonsynonymous SNV	TCP10L2	chr6	167592601	G	G	G	G	A_het
nonsynonymous SNV	TCP10L2	chr6	167592605	T	T	T	T	G_het
nonsynonymous SNV	TCP11L2	chr12	106729425	G	G	A_het	G	G
nonsynonymous SNV	TCR	chr14	22205009	A	A	A	G_het	A
nonsynonymous SNV	TCRA	chr14	22315394	T	T	T	T	C_het
nonsynonymous SNV	TCRBV12S2	chr7	142231694	T	C_het	T	C_het	C_het
nonsynonymous SNV	TCRBV12S2	chr7	142231703	G	T_het	G	T_het	T_het
nonsynonymous SNV	TCRBV12S2	chr7	142231744	A	G_het	G_het	G_het	G_het
nonsynonymous SNV	TCRBV12S2	chr7	142231762	A	T_het	T_het	T_het	T_het
nonsynonymous SNV	TCRBV12S2	chr7	142231769	T	A_het	A_het	A_het	A_het
nonsynonymous SNV	TCRBV12S2	chr7	142231771	T	C_het	C_het	C_het	C_het
nonsynonymous SNV	TCRBV12S2	chr7	142231772	T	G_het	G_het	G_het	G_het
stopgain	TCRBV12S2	chr7	142231780	C	T_het	T_het	T_het	T_het
nonsynonymous SNV	TCRBV12S2	chr7	142231781	A	C_het	C_het	C_het	C_het
nonsynonymous SNV	TCRBV12S2	chr7	142231795	G	T_het	G	T_het	T_het
nonsynonymous SNV	TCRBV12S2	chr7	142231806	C	G_het	G_het	G_het	G_het
nonsynonymous SNV	TCRBV12S2	chr7	142231826	T	C_het	T	T	T
nonsynonymous SNV	TCRBV12S2	chr7	142231852	T	C_het	C_het	C_het	T
nonsynonymous SNV	TCRBV12S2	chr7	142231861	C	A_het	A_het	A_het	C
nonsynonymous SNV	TCRBV12S2	chr7	142231862	T	C_het	T	T	T
nonsynonymous SNV	TCRBV12S3	chr7	142206520	C	A_het	A_het	C	A_het
nonsynonymous SNV	TCRBV12S3	chr7	142206551	G	T_het	T_het	T_het	T_het
nonsynonymous SNV	TCRBV12S3	chr7	142206574	G	A_het	A_het	A_het	A_het
nonsynonymous SNV	TCRBV12S3	chr7	142206581	T	T	C_het	C_het	C_het
nonsynonymous SNV	TCRBV12S3	chr7	142206764	T	T	T	T	C_het
nonsynonymous SNV	TCRBV12S3	chr7	142206770	A	A	A	A	G_het
nonsynonymous SNV	TCRBV12S3	chr7	142206799	C	C	C	C	A_het
nonsynonymous SNV	TCRBV13S1	chr7	142180596	G	A_het	A_het	G	G
nonsynonymous SNV	TCRBV13S1	chr7	142180618	T	C_het	C_het	C_het	T
nonsynonymous SNV	TCRBV13S1	chr7	142180633	G	T_het	T_het	T_het	G
nonsynonymous SNV	TCRBV13S1	chr7	142180635	T	G_het	G_het	G_het	T
nonsynonymous SNV	TCRBV13S1	chr7	142180641	A	G_het	G_het	G_het	G_het
nonsynonymous SNV	TCRBV13S1	chr7	142180647	G	T_het	T_het	T_het	T_het
nonsynonymous SNV	TCRBV13S1	chr7	142180704	G	T_het	T_het	T_het	T_het
nonsynonymous SNV	TCRBV13S1	chr7	142180737	T	A_het	A_het	A_het	A_het
nonsynonymous SNV	TCRBV13S1	chr7	142180770	T	C_het	C_het	C_het	T
nonsynonymous SNV	TCRBV20S1	chr7	142032373	C	T_het	C	C	C
nonsynonymous SNV	TCRBV20S1	chr7	142032374	C	T_het	C	C	C
nonsynonymous SNV	TCRBV20S1	chr7	142032400	G	C_het	G	G	G
nonsynonymous SNV	TCRBV20S1	chr7	142032421	C	A_het	C	C	C
nonsynonymous SNV	TCRBV20S1	chr7	142032571	G	G	C_het	G	G
nonsynonymous SNV	TCRBV21S1	chr7	142223959	C	C	T_het	C	T_het

nonsynonymous SNV	TCRBV21S1	chr7	142223962	G	G	C_het	G	C_het
nonsynonymous SNV	TCRBV21S1	chr7	142223964	A	A	T_het	A	T_het
nonsynonymous SNV	TCRBV21S1	chr7	142223971	C	C	T_het	C	T_het
nonsynonymous SNV	TCRBV21S1	chr7	142223997	A	A	T_het	A	T_het
nonsynonymous SNV	TCRBV21S1	chr7	142224003	C	C	C	C	A_het
nonsynonymous SNV	TCRBV5S1A1T	chr7	142021277	G	G	A_het	G	G
nonsynonymous SNV	TCRBV5S2	chr7	142131832	C	T_het	T_het	T_het	T_het
nonsynonymous SNV	TCRBV5S2	chr7	142131858	C	C	A_het	A_het	A_het
nonsynonymous SNV	TCRBV5S2	chr7	142131868	C	C	T_het	T_het	T_het
nonsynonymous SNV	TCRBV5S6A3N2T	chr7	142168414	G	C_het	C_het	C_het	C_het
nonsynonymous SNV	TCRBV5S6A3N2T	chr7	142168466	A	C_het	C_het	A	C_het
nonsynonymous SNV	TCRBV5S6A3N2T	chr7	142168511	T	A_het	A_het	T	A_het
nonsynonymous SNV	TCRBV5S6A3N2T	chr7	142168518	A	C_het	C_het	A	C_het
nonsynonymous SNV	TCRBV5S6A3N2T	chr7	142168523	T	C_het	C_het	T	C_het
stopgain	TCRBV5S6A3N2T	chr7	142168524	G	A_het	A_het	G	A_het
nonsynonymous SNV	TCRBV5S6A3N2T	chr7	142168527	A	G_het	G_het	A	G_het
nonsynonymous SNV	TCRBV5S6A3N2T	chr7	142168530	T	G_het	G_het	T	G_het
nonsynonymous SNV	TCRBV5S6A3N2T	chr7	142168538	G	A_het	A_het	G	G
nonsynonymous SNV	TCRBV5S6A3N2T	chr7	142168544	T	A_het	A_het	T	T
nonsynonymous SNV	TCRBV5S6A3N2T	chr7	142168799	C	C	T_het	C	C
nonsynonymous SNV	TCRBV5S6A3N2T	chr7	142168825	C	C	A_het	C	C
nonsynonymous SNV	TCRBV5S6A3N2T	chr7	142168835	C	C	T_het	C	C
nonsynonymous SNV	TCRBV6S1A1N1	chr7	142247271	G	G	A_het	A_het	A_het
nonsynonymous SNV	TCRBV6S1A1N1	chr7	142247380	T	T	A_het	T	T
nonsynonymous SNV	TCRBV6S1A1N1	chr7	142247540	G	A_het	A_het	A_het	A_het
nonsynonymous SNV	TCRBV6S1A1N1	chr7	142247546	C	A_het	A_het	A_het	A_het
nonsynonymous SNV	TCRBV6S2A1N1T	chr7	142099512	T	T	T	T	G_het
nonsynonymous SNV	TCRBV6S2A1N1T	chr7	142099533	T	T	C_het	T	C_het
nonsynonymous SNV	TCRBV6S2A1N1T	chr7	142099537	G	G	T_het	G	T_het
nonsynonymous SNV	TCRBV6S2A1N1T	chr7	142099548	A	G_het	G_het	A	G_het
nonsynonymous SNV	TCRBV6S3A1N1T	chr7	142139296	C	C	C	A_het	C
nonsynonymous SNV	TCRBV6S3A1N1T	chr7	142139357	C	T_het	C	T_het	C
nonsynonymous SNV	TCRBV6S3A1N1T	chr7	142139383	T	C_het	T	T	T
nonsynonymous SNV	TCRBV6S3A1N1T	chr7	142139386	G	A_het	G	G	G
nonsynonymous SNV	TCRBV6S3A1N1T	chr7	142139393	C	T_het	C	C	C
nonsynonymous SNV	TCRBV6S3A1N1T	chr7	142139400	G	T_het	G	G	G
nonsynonymous SNV	TCRBV6S6A2T	chr7	142119890	T	A_het	T	A_het	T
nonsynonymous SNV	TCRBV6S6A2T	chr7	142119899	C	T_het	C	T_het	C
nonsynonymous SNV	TCRBV6S6A2T	chr7	142119928	G	A_het	G	A_het	G
nonsynonymous SNV	TCRBV6S6A2T	chr7	142119935	C	T_het	C	T_het	C
nonsynonymous SNV	TCRBV6S6A2T	chr7	142119942	G	T_het	G	G	G
nonsynonymous SNV	TCRBV6S6A2T	chr7	142119946	G	A_het	G	A_het	G
nonsynonymous SNV	TCRBV6S6A2T	chr7	142119959	A	T_het	A	A	A
nonsynonymous SNV	TCRBV6S6A2T	chr7	142119960	A	C_het	A	A	A
nonsynonymous SNV	TCRBV6S6A2T	chr7	142119962	T	G_het	T	T	T

nonsynonymous SNV	TCRBV7S3A2	chr7	142045678	A	G_het	G_het	A	A
nonsynonymous SNV	TCRBV7S3A2	chr7	142045680	T	C_het	C_het	T	T
nonsynonymous SNV	TCRBV7S3A2	chr7	142045683	A	G_het	G_het	A	A
nonsynonymous SNV	TCRBV7S3A2	chr7	142045690	A	G_het	G_het	A	A
nonsynonymous SNV	TCRBV7S3A2	chr7	142045692	A	G_het	G_het	A	A
nonsynonymous SNV	TCRBV7S3A2	chr7	142045693	C	T_het	T_het	C	C
nonsynonymous SNV	TCRBV9S1A1T	chr7	142008434	G	A_het	A_het	G	G
nonsynonymous SNV	TCRBV9S1A1T	chr7	142008440	T	C_het	C_het	T	T
nonsynonymous SNV	TCRBV9S1A1T	chr7	142008442	T	C_het	C_het	T	T
nonsynonymous SNV	TCRBV9S1A1T	chr7	142008577	G	A_het	A_het	G	G
nonsynonymous SNV	TCRBV9S1A1T	chr7	142008638	C	A_het	A_het	C	C
nonsynonymous SNV	TCRBV9S1A1T	chr7	142008645	A	C_het	A	A	A
stopgain	TCRBV9S1A1T	chr7	142008653	T	A_het	T	T	T
nonsynonymous SNV	TCRBV9S1A1T	chr7	142008672	G	A_het	G	G	G
nonsynonymous SNV	TCRBV9S1A1T	chr7	142008675	A	G_het	A	A	A
nonsynonymous SNV	TCRBV9S1A1T	chr7	142008718	T	C_het	T	T	T
nonsynonymous SNV	TCRBV9S1A1T	chr7	142008727	G	T_het	G	G	G
nonsynonymous SNV	TCRBV9S1A1T	chr7	142008733	A	G_het	A	A	A
nonsynonymous SNV	TCRBV9S1A1T	chr7	142008745	T	C_het	T	T	T
nonsynonymous SNV	TCRBV9S1A1T	chr7	142008750	A	T_het	A	A	A
nonsynonymous SNV	TCRBV9S1A1T	chr7	142008783	A	G_het	G_het	A	A
nonsynonymous SNV	TCRBV9S1A1T	chr7	142008785	A	C_het	C_het	A	A
nonsynonymous SNV	TCRDV2	chr14	22932220	T	A_het	T	T	T
nonsynonymous SNV	TCTN1	chr12	111052219	G	G	C_het	G	G
nonsynonymous SNV	TCTN1	chr12	111080097	A	A	G_het	A	A
nonsynonymous SNV	TDRD12	chr19	33285600	C	C	T_het	C	C
nonsynonymous SNV	TDRD6	chr6	46657421	G	G	G	G	T_het
nonsynonymous SNV	TEAD1	chr11	12951749	G	G	A_het	G	G
nonsynonymous SNV	TECPR2	chr14	102898420	G	G	G	G	A_het
nonsynonymous SNV	TECTA	chr11	120998747	C	C	G_het	C	C
nonsynonymous SNV	TECTA	chr11	121016781	C	C	C	C	T_het
nonsynonymous SNV	TEKT2	chr1	36553686	A	A	A	A	G_het
nonsynonymous SNV	TEKT4	chr2	95541442	C	C	C	C	T_het
nonsynonymous SNV	TEKT4	chr2	95541447	C	C	C	C	T_het
nonsynonymous SNV	TEKT4P2	chr21	9907345	C	T_het	T_het	T_hom	T_het
nonsynonymous SNV	TEKT4P2	chr21	9907375	T	C_het	C_het	C_hom	C_het
nonsynonymous SNV	TEKT4P2	chr21	9907384	G	A_het	A_het	A_hom	A_het
nonsynonymous SNV	TEKT4P2	chr21	9907431	G	G	G	G	A_het
nonsynonymous SNV	TENC1	chr12	53453668	C	T_het	C	C	C
nonsynonymous SNV	TENM2	chr5	166711862	G	G	A_het	G	G
nonsynonymous SNV	TENM3	chr4	183651471	A	A	G_het	A	A
nonsynonymous SNV	TENM3	chr4	183721287	C	C	T_het	C	C
nonsynonymous SNV	TENM4	chr11	78413334	C	C	C	C	T_het
nonsynonymous SNV	TESK2	chr1	45810774	C	C	C	T_hom	C
nonsynonymous SNV	TET1	chr10	70332477	G	G	G	T_het	G

nonsynonymous SNV	TET1	chr10	70406124	T	T	A_het	T	T
nonsynonymous SNV	TET2	chr4	106157435	C	C	C	C	G_het
nonsynonymous SNV	TET2	chr4	106157703	T	T	T	T	G_het
nonsynonymous SNV	TET2	chr4	106196770	G	G	A_het	G	G
nonsynonymous SNV	TET3	chr2	74275179	G	G	G	A_het	G
nonsynonymous SNV	TEX13A	chrX	104464099	A	A	A	G_hom	A
nonsynonymous SNV	TEX40	chr11	64071272	G	G	C_het	G	G
nonsynonymous SNV	TFAP2B	chr6	50811001	G	G	T_het	G	G
nonsynonymous SNV	TFDP1	chr13	114288494	G	G	A_het	G	G
nonsynonymous SNV	TFDP1	chr13	114292079	G	G	T_hom	G	G
nonsynonymous SNV	TFDP3	chrX	132351428	T	A_het	T	T	T
nonsynonymous SNV	TFDP3	chrX	132351665	A	T_het	A	A	A
nonsynonymous SNV	TFEC	chr7	115624396	C	T_het	C	C	C
nonsynonymous SNV	TFR2	chr7	100218631	C	T_het	C	C	C
nonsynonymous SNV	TG	chr8	133900382	C	C	C	C	T_hom
nonsynonymous SNV	TGIF2LX	chrX	89177431	G	G	A_hom	G	G
nonsynonymous SNV	TGM4	chr3	44945459	C	C	T_hom	C	C
nonsynonymous SNV	TGM5	chr15	43527058	C	C	T_het	C	C
nonsynonymous SNV	TGOLN2	chr2	85554287	T	T	T	T	C_het
nonsynonymous SNV	THAP10	chr15	71184353	G	G	A_het	G	G
nonsynonymous SNV	THAP7	chr22	21354970	C	A_hom	C	C	C
nonsynonymous SNV	THBS2	chr6	169629714	C	C	T_het	C	C
nonsynonymous SNV	THEG	chr19	362268	C	C	T_het	C	C
nonsynonymous SNV	THEG	chr19	375735	T	C_het	T	T	T
nonsynonymous SNV	THEGL	chr4	57425674	A	A	A	G_hom	A
nonsynonymous SNV	THEGL	chr4	57462696	A	A	A	A	G_het
nonsynonymous SNV	THRA1	chr17	38250149	C	C	C	A_hom	C
nonsynonymous SNV	THSD7A	chr7	11675904	T	T	T	C_het	T
nonsynonymous SNV	THSD7B	chr2	138413146	G	G	G	C_het	G
nonsynonymous SNV	TIAM1	chr21	32638963	G	G	A_het	G	G
nonsynonymous SNV	TIAM2	chr6	155469278	C	C	G_het	C	C
nonsynonymous SNV	TIMD4	chr5	156381639	C	C	C	C	T_het
nonsynonymous SNV	TIMM21	chr18	71825639	G	G	A_het	G	G
nonsynonymous SNV	TINF2	chr14	24709874	T	T	C_het	T	T
nonsynonymous SNV	TIPARP	chr3	156422787	T	T	A_het	T	T
nonsynonymous SNV	TIPIN	chr15	66641754	T	T	T	C_het	T
nonsynonymous SNV	TISP43	chr2	131334386	G	A_het	G	G	G
nonsynonymous SNV	TJP2	chr9	71836293	G	G	A_het	G	G
stopgain	TJP2	chr9	71866260	C	C	C	C	T_het
nonsynonymous SNV	TJP3	chr19	3747982	C	C	C	T_hom	C
nonsynonymous SNV	TJP3	chr19	3750179	T	T	T	C_het	T
nonsynonymous SNV	TKTL1	chrX	153533674	C	C	C	C	A_het
nonsynonymous SNV	TLDC1	chr16	84513589	T	T	T	C_het	T
nonsynonymous SNV	TLE4	chr9	82335082	C	C	T_het	C	C
nonsynonymous SNV	TLL2	chr10	98138791	G	G	G	C_het	G

nonsynonymous SNV	TLN2	chr15	63029196	G	G	G	A_het	G
nonsynonymous SNV	TLN2	chr15	63102176	G	G	A_het	G	G
nonsynonymous SNV	TLR3	chr4	186997807	G	G	G	G	C_het
nonsynonymous SNV	TLR8	chrX	12939303	G	G	G	A_hom	G
nonsynonymous SNV	TLX2	chr2	74743403	G	G	A_het	G	G
nonsynonymous SNV	TLX3	chr5	170738586	G	G	G	G	C_het
nonsynonymous SNV	TM9SF1	chr14	24662163	G	G	A_het	G	G
nonsynonymous SNV	TMC2	chr20	2591122	G	G	G	A_het	G
nonsynonymous SNV	TMC6	chr17	76117107	C	C	T_het	C	C
nonsynonymous SNV	TMCC2	chr1	205238322	G	G	A_het	G	G
nonsynonymous SNV	TMED6	chr16	69377470	A	A	G_het	A	A
nonsynonymous SNV	TMED7	chr5	114951968	C	C	C	G_het	C
nonsynonymous SNV	TMEM104	chr17	72791667	G	G	G	A_hom	G
nonsynonymous SNV	TMEM108	chr3	133098661	G	G	G	G	C_het
nonsynonymous SNV	TMEM133	chr11	100863293	A	A	G_het	A	A
nonsynonymous SNV	TMEM144	chr4	159154076	A	A	A	A	G_het
nonsynonymous SNV	TMEM156	chr4	39033891	T	T	T	T	C_het
nonsynonymous SNV	TMEM186	chr16	8890030	G	G	A_het	G	G
nonsynonymous SNV	TMEM2	chr9	74312944	G	G	G	G	A_hom
nonsynonymous SNV	TMEM2	chr9	74359949	T	T	C_het	T	T
nonsynonymous SNV	TMEM2	chr9	74365135	A	A	G_het	A	A
nonsynonymous SNV	TMEM200C	chr18	5891041	G	G	G	G	A_het
nonsynonymous SNV	TMEM205	chr19	11456271	C	C	C	T_het	C
nonsynonymous SNV	TMEM232	chr5	109864536	C	C	C	T_het	C
nonsynonymous SNV	TMEM246	chr9	104238756	C	C	C	T_hom	C
nonsynonymous SNV	TMEM254	chr10	81838813	C	C	C	A_het	C
nonsynonymous SNV	TMEM41A	chr3	185209392	C	C	G_het	C	C
nonsynonymous SNV	TMEM5	chr12	64196106	G	G	A_het	G	G
nonsynonymous SNV	TMEM63A	chr1	226048680	T	T	T	T	C_het
nonsynonymous SNV	TMEM70	chr8	74888616	G	G	G	G	T_hom
nonsynonymous SNV	TMEM70	chr8	74888617	C	C	C	C	T_hom
nonsynonymous SNV	TMEM72	chr10	45427469	T	T	T	C_het	T
nonsynonymous SNV	TMEM8B	chr9	35846562	G	G	A_het	G	G
nonsynonymous SNV	TMEM95	chr17	7258633	A	A	G_het	A	A
nonsynonymous SNV	TMIE	chr3	46742941	C	A_hom	C	C	C
nonsynonymous SNV	TMIGD1	chr17	28651840	C	C	T_het	C	C
nonsynonymous SNV	TMPO	chr12	98927344	G	G	G	C_het	G
stopgain	TMPPE	chr3	33134898	G	G	G	A_hom	G
nonsynonymous SNV	TMPRSS11F	chr4	68930465	G	A_het	G	G	G
nonsynonymous SNV	TMPRSS11F	chr4	68935708	T	T	T	T	A_het
nonsynonymous SNV	TMPRSS2	chr21	42879910	C	C	C	G_het	C
nonsynonymous SNV	TMPRSS3	chr21	43804100	C	G_het	C	C	C
stopgain	TMPRSS7	chr3	111780659	C	C	C	C	T_het
nonsynonymous SNV	TMPRSS9	chr19	2408544	G	G	A_het	G	G
nonsynonymous SNV	TMX2	chr11	57480232	G	C_het	G	G	G

nonsynonymous SNV	TMX3	chr18	66348328	C	C	T_het	C	C
nonsynonymous SNV	TNC	chr9	117848368	C	C	T_het	C	C
nonsynonymous SNV	TNFRSF12A	chr16	3071682	A	A	A	A	G_hom
nonsynonymous SNV	TNFRSF4	chr1	1147024	G	G	A_het	G	G
nonsynonymous SNV	TNFSF11	chr13	43148519	A	A	G_het	A	A
nonsynonymous SNV	TNK2	chr3	195613853	T	T	T	A_hom	T
nonsynonymous SNV	TNKS1BP1	chr11	57068505	C	T_het	C	C	C
nonsynonymous SNV	TNKS1BP1	chr11	57076287	C	C	C	C	G_het
nonsynonymous SNV	TNN	chr1	175046762	G	G	G	A_hom	G
nonsynonymous SNV	TNN	chr1	175092631	A	C_het	A	A	A
nonsynonymous SNV	TNPO2	chr19	12832004	G	C_het	G	G	G
nonsynonymous SNV	TNRC18	chr7	5401585	C	C	T_het	C	C
nonsynonymous SNV	TNRC18	chr7	5402453	G	A_het	G	G	G
nonsynonymous SNV	TNRC18	chr7	5415796	G	G	G	G	A_het
nonsynonymous SNV	TNRC18	chr7	5428454	G	G	A_het	G	G
nonsynonymous SNV	TNRC6A	chr16	24800897	C	C	C	T_het	C
nonsynonymous SNV	TNRC6A	chr16	24801468	C	C	C	G_het	C
nonsynonymous SNV	TNRC6C	chr17	76046623	G	A_het	G	G	G
nonsynonymous SNV	TNS1	chr2	218855775	T	T	C_het	T	T
nonsynonymous SNV	TNS3	chr7	47436451	T	C_het	T	T	T
stopgain	TOE1	chr1	45808107	C	C	T_het	C	C
nonsynonymous SNV	TOM1	chr22	35730426	C	T_het	C	C	C
nonsynonymous SNV	TOM1L1	chr17	53027406	C	C	T_het	C	C
stopgain	TOP1MT	chr8	144398219	G	G	A_het	G	G
nonsynonymous SNV	TOP1MT	chr8	144406248	C	C	T_het	C	C
nonsynonymous SNV	TOP3A	chr17	18205233	G	G	G	G	T_hom
nonsynonymous SNV	TOX2	chr20	42694484	C	C	C	T_het	C
stopgain	TP53	chr17	7574003	G	G	G	A_hom	G
nonsynonymous SNV	TP53	chr17	7577121	G	G	A_hom	G	G
nonsynonymous SNV	TP53BP1	chr15	43724532	T	T	T	T	C_het
nonsynonymous SNV	TP53INP2	chr20	33296619	G	G	T_het	G	G
nonsynonymous SNV	TP63	chr3	189590764	C	C	G_het	C	C
nonsynonymous SNV	TPD52L3	chr9	6328996	G	G	A_het	G	G
nonsynonymous SNV	TPGS1	chr19	507834	C	C	T_het	C	C
nonsynonymous SNV	TPH2	chr12	72372833	C	C	T_het	C	C
nonsynonymous SNV	TPR	chr1	186315299	T	T	T	T	G_het
nonsynonymous SNV	TPSAB1	chr16	1291175	G	G	G	G	A_het
nonsynonymous SNV	TPSAB1	chr16	1291318	G	G	G	G	C_het
nonsynonymous SNV	TPSAB1	chr16	1291586	G	G	A_het	G	G
nonsynonymous SNV	TPSAB1	chr16	1291598	G	G	G	G	A_het
nonsynonymous SNV	TPSAB1	chr16	1291608	A	A	A	A	G_het
nonsynonymous SNV	TPSAB1	chr16	1291623	C	C	C	C	T_het
nonsynonymous SNV	TPSB2	chr16	1278744	C	C	C	C	T_het
nonsynonymous SNV	TPSB2	chr16	1279714	A	A	A	A	G_het
nonsynonymous SNV	TPSB2	chr16	1279717	C	C	C	C	T_het

nonsynonymous SNV	TPSD1	chr16	1306802	A	A	A	A	G_het
nonsynonymous SNV	TPSD1	chr16	1306817	G	G	G	G	A_het
nonsynonymous SNV	TPSD1	chr16	1306971	A	A	A	A	G_het
nonsynonymous SNV	TPSD1	chr16	1306973	A	A	A	A	G_het
nonsynonymous SNV	TPSD1	chr16	1306986	C	C	C	C	T_het
nonsynonymous SNV	TPT1	chr13	45912857	G	G	A_het	G	G
stopgain	TPTE	chr21	10942756	G	A_het	A_het	A_het	G
nonsynonymous SNV	TPTE	chr21	10942923	G	G	G	A_het	G
nonsynonymous SNV	TPTE	chr21	10943003	C	C	T_het	C	C
nonsynonymous SNV	TPTE2	chr13	20066994	T	T	T	C_het	T
nonsynonymous SNV	TPTE2	chr13	20067011	A	A	A	C_het	A
nonsynonymous SNV	TRAPPC8	chr18	29432443	T	T	T	T	C_het
nonsynonymous SNV	TRDN	chr6	123824975	C	C	T_het	C	C
nonsynonymous SNV	TREH	chr11	118529044	C	G_hom	C	C	C
nonsynonymous SNV	TRERF1	chr6	42204016	G	G	A_het	G	G
nonsynonymous SNV	TRHDE	chr12	73012792	C	C	A_het	C	C
nonsynonymous SNV	TRIL	chr7	28997250	G	G	A_het	G	G
nonsynonymous SNV	TRIM16L	chr17	18631034	C	C	C	G_het	C
nonsynonymous SNV	TRIM23	chr5	64906860	A	A	G_het	A	A
nonsynonymous SNV	TRIM29	chr11	120008447	G	G	C_hom	G	G
nonsynonymous SNV	TRIM44	chr11	35747665	A	G_het	A	A	A
nonsynonymous SNV	TRIM47	chr17	73874223	G	G	G	G	A_het
nonsynonymous SNV	TRIM49C	chr11	89774236	C	C	G_het	C	C
nonsynonymous SNV	TRIM50	chr7	72727296	C	T_het	C	C	C
nonsynonymous SNV	TRIM54	chr2	27529115	C	C	C	C	T_het
nonsynonymous SNV	TRIM59	chr3	160156200	G	A_het	G	G	G
nonsynonymous SNV	TRIM63	chr1	26387780	T	T	T	T	A_het
nonsynonymous SNV	TRIM68	chr11	4621993	C	C	G_het	C	C
nonsynonymous SNV	TRIM77	chr11	89450618	T	T	C_het	T	T
nonsynonymous SNV	TRIP10	chr19	6746485	G	G	G	A_het	G
nonsynonymous SNV	TRIP6	chr7	100465824	G	A_het	G	G	G
nonsynonymous SNV	TRMT1	chr19	13216136	T	T	T	C_het	T
nonsynonymous SNV	TRO	chrX	54952116	C	C	C	C	T_hom
nonsynonymous SNV	TROAP	chr12	49723191	G	G	G	A_het	G
nonsynonymous SNV	TROAP	chr12	49724993	T	T	C_het	T	T
nonsynonymous SNV	TRPC3	chr4	122854037	G	G	G	G	C_het
nonsynonymous SNV	TRPC4	chr13	38266346	C	C	T_het	C	C
nonsynonymous SNV	TRPM1	chr15	31294573	G	G	G	G	C_het
nonsynonymous SNV	TRPM5	chr11	2436670	G	G	A_het	G	G
nonsynonymous SNV	TRPS1	chr8	116430646	C	C	T_het	C	C
nonsynonymous SNV	TRPS1	chr8	116617001	G	G	G	A_het	G
nonsynonymous SNV	TRPV4	chr12	110230597	C	C	T_het	C	C
nonsynonymous SNV	TRPV5	chr7	142625305	G	G	G	A_het	G
nonsynonymous SNV	TRPV6	chr7	142571437	C	C	C	C	T_hom
nonsynonymous SNV	TSACC	chr1	156314497	C	T_het	C	C	C

nonsynonymous SNV	TSC2	chr16	2134539	G	G	G	A_het	G
nonsynonymous SNV	TSC2	chr16	2136297	C	C	T_het	C	C
nonsynonymous SNV	TSC22D2	chr3	150128626	G	G	G	A_hom	G
nonsynonymous SNV	TSEN34	chr19	54697479	T	T	T	C_het	T
nonsynonymous SNV	TSEN54	chr17	73519758	C	C	G_het	C	C
nonsynonymous SNV	TSHZ1	chr18	72999157	G	G	A_het	G	G
nonsynonymous SNV	TSKU	chr11	76507190	C	C	C	C	T_het
nonsynonymous SNV	TSNARE1	chr8	143381911	G	G	G	G	A_hom
nonsynonymous SNV	TSPAN10	chr17	79612579	C	C	T_het	C	C
nonsynonymous SNV	TSPEAR	chr21	45947285	G	G	T_het	G	G
nonsynonymous SNV	TSPEAR	chr21	45987803	G	G	A_het	G	G
nonsynonymous SNV	TTBK2	chr15	43067832	C	C	C	G_het	C
nonsynonymous SNV	TTC22	chr1	55246936	G	G	G	A_hom	G
nonsynonymous SNV	TTC22	chr1	55253419	C	C	C	T_hom	C
nonsynonymous SNV	TTC29	chr4	147724786	C	C	C	C	A_het
nonsynonymous SNV	TTC3	chr21	38538291	G	G	G	A_het	G
nonsynonymous SNV	TTC39B	chr9	15186960	T	T	C_het	T	T
nonsynonymous SNV	TTC7A	chr2	47168838	G	G	G	G	C_het
nonsynonymous SNV	TTK	chr6	80750397	A	A	A	C_hom	A
nonsynonymous SNV	TTLL3	chr3	9852037	G	G	G	G	T_het
nonsynonymous SNV	TTLL4	chr2	219611871	G	G	A_het	G	G
nonsynonymous SNV	TTLL4	chr2	219617523	A	G_het	A	A	A
stopgain	TTLL6	chr17	46865282	G	G	A_het	G	G
nonsynonymous SNV	TTN	chr2	179412830	G	G	A_het	G	G
nonsynonymous SNV	TTN	chr2	179447193	T	T	C_het	T	T
nonsynonymous SNV	TTN	chr2	179452790	C	C	T_het	C	C
nonsynonymous SNV	TTN	chr2	179453429	G	G	A_het	G	G
nonsynonymous SNV	TTN	chr2	179463495	G	G	A_het	G	G
nonsynonymous SNV	TTN	chr2	179566951	C	C	T_het	C	C
nonsynonymous SNV	TTN	chr2	179582769	C	A_het	C	C	C
nonsynonymous SNV	TTN	chr2	179596181	G	G	G	C_het	G
nonsynonymous SNV	TTN	chr2	179596554	T	C_het	T	T	T
nonsynonymous SNV	TTN	chr2	179605380	T	T	A_het	T	T
nonsynonymous SNV	TTN	chr2	179610619	C	C	C	G_het	C
nonsynonymous SNV	TTN	chr2	179658175	C	C	T_het	C	C
nonsynonymous SNV	TTPAL	chr20	43115297	G	G	A_het	G	G
nonsynonymous SNV	TTYH1	chr19	54947332	G	C_het	G	G	G
nonsynonymous SNV	TUB	chr11	8060566	G	A_het	G	G	G
nonsynonymous SNV	TUBA1C	chr12	49666509	C	T_het	T_het	T_hom	C
nonsynonymous SNV	TUBA8	chr22	18613694	A	A	A	A	T_het
nonsynonymous SNV	TUBA8	chr22	18613707	C	C	C	C	T_het
nonsynonymous SNV	TUBB4Q	chr16	90161730	C	C	C	T_het	C
nonsynonymous SNV	TUBB4Q	chr4	190903938	C	C	T_het	C	C
nonsynonymous SNV	TUBB4Q	chr4	190903950	A	A	G_het	A	A
nonsynonymous SNV	TUBB4Q	chr4	190904058	G	G	A_het	G	G

nonsynonymous SNV	TUBB8	chr10	93827	T	T	T	C_het	T
nonsynonymous SNV	TUBB8	chr10	93943	A	A	A	G_het	A
nonsynonymous SNV	TUBB8	chr10	94004	C	C	C	T_het	C
nonsynonymous SNV	TUBBP5	chr9	141069905	C	T_hom	C	T_het	C
nonsynonymous SNV	TUBGCP2	chr10	135093287	C	C	C	T_het	C
nonsynonymous SNV	TUBGCP2	chr10	135094925	C	C	T_het	C	C
nonsynonymous SNV	TUBGCP5	chr15	22846897	G	G	G	A_het	G
nonsynonymous SNV	TULP2	chr19	49384272	T	T	T	C_het	T
nonsynonymous SNV	TULP3	chr12	3018728	G	G	T_het	G	G
nonsynonymous SNV	TULP3	chr12	3029947	A	G_het	A	A	A
nonsynonymous SNV	TVP23A	chr16	10864165	C	C	C	C	A_hom
nonsynonymous SNV	TXNDC5	chr6	7883468	C	T_het	C	C	C
nonsynonymous SNV	TYK2	chr19	10464843	G	A_het	G	G	G
nonsynonymous SNV	TYR	chr11	89017973	C	T_het	C	C	C
nonsynonymous SNV	TYRO3	chr15	41862515	G	G	G	A_het	G
nonsynonymous SNV	UACA	chr15	70991990	C	C	C	C	T_het
nonsynonymous SNV	UBE2D4	chr7	43978084	G	A_het	G	G	G
nonsynonymous SNV	UBE3B	chr12	109971762	G	G	A_het	G	G
nonsynonymous SNV	UBQLN3	chr11	5528864	C	T_het	C	C	C
nonsynonymous SNV	UBR2	chr6	42612262	A	A	A	A	G_het
nonsynonymous SNV	UBR4	chr1	19490852	G	G	G	A_hom	G
nonsynonymous SNV	UBR4	chr1	19504035	G	G	T_het	G	G
nonsynonymous SNV	UBXN6	chr19	4446057	C	C	C	C	T_het
nonsynonymous SNV	UCHL5	chr1	192997236	A	A	G_het	A	A
nonsynonymous SNV	UCP3	chr11	73714902	G	G	A_het	G	G
nonsynonymous SNV	UFL1	chr6	96974255	G	G	G	G	T_het
nonsynonymous SNV	UGGT2	chr13	96511868	T	T	T	A_het	T
nonsynonymous SNV	UGT1A5	chr2	234622310	C	T_het	C	T_het	T_het
nonsynonymous SNV	UGT2B11	chr4	70070352	C	A_het	C	C	C
nonsynonymous SNV	UGT2B4	chr4	70360912	C	C	A_het	C	C
nonsynonymous SNV	UGT3A1	chr5	35965556	G	G	G	A_het	G
nonsynonymous SNV	UHRF1	chr19	4950715	G	G	A_het	G	G
nonsynonymous SNV	UHRF1BP1L	chr12	100452062	T	T	T	C_het	T
nonsynonymous SNV	ULK1	chr12	132393239	G	G	A_het	G	G
nonsynonymous SNV	ULK1	chr12	132400600	C	C	C	G_het	C
nonsynonymous SNV	UMODL1	chr21	43543193	G	G	A_het	G	G
nonsynonymous SNV	UNC13C	chr15	54305206	C	C	C	C	T_het
nonsynonymous SNV	UNC13C	chr15	54919180	C	C	C	C	A_het
nonsynonymous SNV	UNC45A	chr15	91496233	G	G	A_het	G	G
nonsynonymous SNV	UNC5B	chr10	73051273	A	G_het	A	A	A
nonsynonymous SNV	UNC5D	chr8	35425706	C	C	T_het	C	C
nonsynonymous SNV	UNC80	chr2	210704104	G	G	A_het	G	G
nonsynonymous SNV	UNC93A	chr6	167711457	C	C	C	C	T_het
nonsynonymous SNV	UNG	chr12	109541334	C	C	T_het	C	C
nonsynonymous SNV	UPF1	chr19	18966027	A	A	G_het	A	A

nonsynonymous SNV	UPRT	chrX	74519606	G	G	A_hom	G	G
nonsynonymous SNV	UQCRC1	chr3	48641060	C	C	C	C	G_het
nonsynonymous SNV	URB1	chr21	33709719	C	C	T_het	C	C
nonsynonymous SNV	URB2	chr1	229771835	C	C	C	C	T_het
nonsynonymous SNV	URB2	chr1	229787030	G	G	A_het	G	G
nonsynonymous SNV	URGCP	chr7	43921261	T	T	C_het	T	T
nonsynonymous SNV	URI1	chr19	30506476	G	G	G	A_het	G
nonsynonymous SNV	UROD	chr1	45479759	A	A	A	A	G_het
nonsynonymous SNV	UROD	chr1	45481018	G	G	G	A_hom	G
nonsynonymous SNV	USH2A	chr1	215847956	C	A_het	C	C	C
nonsynonymous SNV	USH2A	chr1	215848436	A	A	A	G_hom	A
nonsynonymous SNV	USH2A	chr1	215848748	T	T	T	C_hom	T
nonsynonymous SNV	USH2A	chr1	216062183	C	C	C	C	T_het
nonsynonymous SNV	USH2A	chr1	216166454	T	G_het	T	T	T
nonsynonymous SNV	USO1	chr4	76730178	G	C_het	G	G	G
nonsynonymous SNV	USP17L15	chr4	9237044	T	T	T	T	A_hom
nonsynonymous SNV	USP17L7	chr8	11990617	C	A_het	A_het	C	A_hom
nonsynonymous SNV	USP17L7	chr8	11990624	T	C_het	C_het	T	C_hom
nonsynonymous SNV	USP17L7	chr8	11991134	G	G	G	C_hom	G
nonsynonymous SNV	USP19	chr3	49155442	C	C	C	C	T_het
nonsynonymous SNV	USP21	chr1	161130701	C	C	T_het	C	C
stopgain	USP21	chr1	161133388	C	C	T_het	C	C
nonsynonymous SNV	USP29	chr19	57641413	A	A	A	T_het	A
nonsynonymous SNV	USP31	chr16	23091349	G	G	A_het	G	G
nonsynonymous SNV	USP31	chr16	23160057	C	C	T_het	C	C
nonsynonymous SNV	USP34	chr2	61575266	A	A	A	A	G_het
nonsynonymous SNV	USP41	chr22	20705118	A	A	A	A	G_het
nonsynonymous SNV	USP42	chr7	6194379	G	C_het	G	G	G
nonsynonymous SNV	USP54	chr10	75289273	C	C	C	C	T_het
nonsynonymous SNV	USP6	chr17	5035624	C	C	C	C	T_het
nonsynonymous SNV	USP6	chr17	5035634	G	G	G	G	T_het
nonsynonymous SNV	USP6	chr17	5035641	C	C	C	C	T_het
nonsynonymous SNV	USP6	chr17	5036205	A	A	A	A	C_het
nonsynonymous SNV	USP6	chr17	5036210	T	T	T	T	G_het
nonsynonymous SNV	USP6	chr17	5036211	C	C	C	C	T_het
nonsynonymous SNV	USP6	chr17	5036214	C	C	C	C	T_het
nonsynonymous SNV	USP6	chr17	5036224	C	C	C	C	G_het
nonsynonymous SNV	USP6	chr17	5036274	C	C	C	C	A_het
nonsynonymous SNV	USP6	chr17	5036281	G	G	G	G	C_het
nonsynonymous SNV	USP7	chr16	8997214	A	A	G_het	A	A
nonsynonymous SNV	UTP15	chr5	72875704	A	A	A	C_het	A
nonsynonymous SNV	UTRN	chr6	144832189	G	G	A_het	G	G
nonsynonymous SNV	UVSSA	chr4	1348920	G	A_het	G	G	G
nonsynonymous SNV	WARS2	chr1	119575820	G	G	A_het	G	G
nonsynonymous SNV	WASH2P	chr2	114355167	A	A	A	A	G_het

nonsynonymous SNV	WASH2P	chr2	114355998	C	G_het	C	G_het	C
nonsynonymous SNV	WASL	chr7	123388721	G	G	G	T_het	G
nonsynonymous SNV	VASN	chr16	4430910	T	T	T	T	C_hom
nonsynonymous SNV	VASN	chr16	4432619	G	G	A_het	G	G
nonsynonymous SNV	VAV1	chr19	6850712	A	A	A	T_het	A
nonsynonymous SNV	VAV3	chr1	108303467	G	G	G	G	A_het
nonsynonymous SNV	VAX1	chr10	118893836	C	C	C	T_het	C
nonsynonymous SNV	VAX2	chr2	71148390	G	G	A_het	G	G
nonsynonymous SNV	WBP11	chr12	14943486	G	A_het	G	G	G
nonsynonymous SNV	WBP2NL	chr22	42422874	C	C	C	A_hom	C
nonsynonymous SNV	VCAN	chr5	82817034	C	C	C	T_het	C
nonsynonymous SNV	VCX	chrX	7811747	T	C_het	T	T	C_hom
nonsynonymous SNV	VCX	chrX	7812017	C	T_het	C	C	C
nonsynonymous SNV	VCX2	chrX	8138171	T	C_hom	C_hom	C_hom	C_hom
nonsynonymous SNV	VCX2	chrX	8138182	A	G_hom	G_hom	G_hom	G_hom
nonsynonymous SNV	WDFY4	chr10	50040632	T	T	T	T	C_het
nonsynonymous SNV	WDFY4	chr10	50105597	G	G	G	A_het	G
nonsynonymous SNV	WDPCP	chr2	63631285	C	G_het	C	C	C
nonsynonymous SNV	VDR	chr12	48238569	G	G	A_het	G	G
nonsynonymous SNV	WDR13	chrX	48462730	C	A_het	C	C	C
nonsynonymous SNV	WDR17	chr4	177046476	C	C	C	C	T_het
nonsynonymous SNV	WDR25	chr14	100847658	C	C	T_het	C	C
nonsynonymous SNV	WDR25	chr14	100996179	G	G	A_het	G	G
nonsynonymous SNV	WDR33	chr2	128522189	T	T	T	C_het	T
nonsynonymous SNV	WDR34	chr9	131403128	C	C	C	C	T_hom
nonsynonymous SNV	WDR34	chr9	131418828	A	C_hom	C_hom	C_hom	C_hom
nonsynonymous SNV	WDR55	chr5	140049099	C	C	T_het	C	C
nonsynonymous SNV	WDR60	chr7	158684004	C	A_het	C	C	C
nonsynonymous SNV	WDR61	chr15	78584984	T	T	T	T	C_het
nonsynonymous SNV	WDR81	chr17	1631365	G	G	G	A_hom	G
nonsynonymous SNV	WDR81	chr17	1637174	G	A_het	G	G	G
nonsynonymous SNV	WDR83OS	chr19	12780204	T	T	C_het	T	T
nonsynonymous SNV	WDR87	chr19	38377302	C	C	C	C	T_het
nonsynonymous SNV	WDR87	chr19	38377773	G	G	G	A_het	A_het
nonsynonymous SNV	WDR87	chr19	38379446	C	C	C	T_hom	T_hom
nonsynonymous SNV	WDR87	chr19	38383897	A	T_het	A	A	A
nonsynonymous SNV	WDR87	chr19	38385079	C	C	C	C	T_het
nonsynonymous SNV	WDR90	chr16	706814	G	G	G	G	A_hom
nonsynonymous SNV	WDR90	chr16	712872	C	C	C	T_het	C
nonsynonymous SNV	WDR96	chr10	105926314	A	C_het	A	A	A
nonsynonymous SNV	VEGFC	chr4	177608653	T	T	C_het	T	T
nonsynonymous SNV	VENTX	chr10	135051425	C	C	C	T_het	C
stopgain	VENTX	chr10	135053446	G	G	G	G	A_het
nonsynonymous SNV	WFIKKN2	chr17	48917789	C	C	G_het	C	C
nonsynonymous SNV	VGLL3	chr3	87018099	T	A_het	T	T	T

nonsynonymous SNV	VILL	chr3	38039631	C	C	C	C	A_het
nonsynonymous SNV	WIPI2	chr7	5262288	G	G	G	A_het	G
nonsynonymous SNV	VIPR1	chr3	42567381	C	A_hom	C	C	C
stopgain	VIPR1	chr3	42573764	C	C	C	C	T_het
nonsynonymous SNV	VIPR1	chr3	42576553	C	T_hom	C	C	C
nonsynonymous SNV	VN1R4	chr19	53770764	G	G	A_het	A_het	A_het
nonsynonymous SNV	WNK1	chr12	1006664	G	G	G	G	A_het
nonsynonymous SNV	WNK3	chrX	54259287	G	G	A_hom	G	G
nonsynonymous SNV	VNN1	chr6	133013662	C	C	C	G_hom	C
nonsynonymous SNV	WNT1	chr12	49375317	C	C	C	T_het	C
nonsynonymous SNV	WNT10A	chr2	219754822	G	G	A_het	G	G
nonsynonymous SNV	VPS11	chr11	118951957	G	A_het	G	G	G
nonsynonymous SNV	VPS13B	chr8	100523657	G	G	A_het	G	G
nonsynonymous SNV	VPS13B	chr8	100791158	G	G	A_het	G	G
nonsynonymous SNV	VPS13C	chr15	62221708	A	A	A	A	G_het
nonsynonymous SNV	VPS37A	chr8	17132441	A	T_het	A	A	A
nonsynonymous SNV	VPS39	chr15	42484267	C	C	C	C	T_het
nonsynonymous SNV	VPS4B	chr18	61064426	T	T	G_het	T	T
nonsynonymous SNV	VPS4B	chr18	61064448	T	T	G_het	T	T
stopgain	VPS4B	chr18	61071042	G	G	A_het	G	G
nonsynonymous SNV	WRN	chr8	30949398	C	C	C	C	G_hom
stopgain	WSCD2	chr12	108600113	C	C	T_het	C	C
nonsynonymous SNV	VSIG2	chr11	124621425	C	A_het	C	C	C
nonsynonymous SNV	VSIG8	chr1	159825833	C	T_het	C	C	C
nonsynonymous SNV	VSX1	chr20	25057001	C	C	T_het	C	C
nonsynonymous SNV	VTA1	chr6	142525141	C	C	C	G_hom	C
nonsynonymous SNV	VWA3A	chr16	22122273	C	T_het	C	C	C
nonsynonymous SNV	VWA8	chr13	42295623	C	C	A_het	C	C
nonsynonymous SNV	VWA8	chr13	42393472	G	T_hom	G	G	G
nonsynonymous SNV	WWC3	chrX	10106944	C	C	C	T_hom	C
nonsynonymous SNV	VWF	chr12	6078487	A	A	A	G_het	A
nonsynonymous SNV	VWF	chr12	6166031	C	C	T_het	C	C
nonsynonymous SNV	WWOX	chr16	79245790	A	A	G_het	A	A
nonsynonymous SNV	WWP1	chr8	87464873	C	C	A_het	C	C
nonsynonymous SNV	WWP2	chr16	69833153	G	G	A_het	G	G
nonsynonymous SNV	X97876	chr9	66499461	C	G_het	G_het	C	C
nonsynonymous SNV	X97876	chr9	66499553	T	T	G_het	T	T
nonsynonymous SNV	XAB2	chr19	7688153	G	G	G	G	C_het
nonsynonymous SNV	XCR1	chr3	46063295	T	G_hom	T	T	T
nonsynonymous SNV	XDH	chr2	31560521	T	T	T	C_het	T
nonsynonymous SNV	XIRP1	chr3	39229196	G	T_hom	G	G	G
nonsynonymous SNV	XIRP2	chr2	168104183	C	G_het	C	C	C
nonsynonymous SNV	XKRX	chrX	100169769	A	C_het	A	A	A
nonsynonymous SNV	XRN1	chr3	142098982	C	C	T_het	C	C
nonsynonymous SNV	XRRA1	chr11	74648035	C	C	T_het	C	C

nonsynonymous SNV	XYLB	chr3	38454497	C	C	C	T_hom	C
nonsynonymous SNV	XYLT1	chr16	17211505	G	G	G	C_het	G
nonsynonymous SNV	XYLT1	chr16	17564311	C	C	A_het	C	C
nonsynonymous SNV	YIPF1	chr1	54332025	G	G	G	G	A_het
nonsynonymous SNV	YTHDC2	chr5	112888880	C	C	T_het	C	C
nonsynonymous SNV	YTHDF1	chr20	61833643	C	C	T_het	C	C
nonsynonymous SNV	YY1AP1	chr1	155630245	T	C_het	T	T	T
nonsynonymous SNV	ZAN	chr7	100350787	A	A	A	G_het	A
nonsynonymous SNV	ZAN	chr7	100364190	G	G	G	A_het	G
nonsynonymous SNV	ZAN	chr7	100365467	G	A_het	G	G	G
nonsynonymous SNV	ZAN	chr7	100370919	C	C	A_het	C	C
nonsynonymous SNV	ZAP70	chr2	98340512	G	G	A_het	G	G
nonsynonymous SNV	ZBBX	chr3	167000256	G	C_het	C_het	G	C_het
nonsynonymous SNV	ZBTB47	chr3	42700696	C	C	C	G_hom	C
nonsynonymous SNV	ZBTB7A	chr19	4054509	G	G	A_het	G	G
nonsynonymous SNV	ZBTB7C	chr18	45566421	A	A	C_het	A	A
nonsynonymous SNV	ZBTB8A	chr1	33065830	T	C_hom	T	T	T
nonsynonymous SNV	ZC3H12A	chr1	37948703	G	G	A_het	G	G
nonsynonymous SNV	ZC3H13	chr13	46543014	C	C	C	T_het	C
nonsynonymous SNV	ZC3H13	chr13	46559789	C	C	C	T_het	C
nonsynonymous SNV	ZC3H6	chr2	113069416	C	C	T_het	C	C
nonsynonymous SNV	ZC3H7A	chr16	11850150	C	C	G_het	C	C
nonsynonymous SNV	ZCCHC11	chr1	52896826	T	T	T	C_hom	T
nonsynonymous SNV	ZCCHC4	chr4	25314438	G	G	G	A_hom	G
nonsynonymous SNV	ZCCHC8	chr12	122958181	T	T	C_het	T	T
nonsynonymous SNV	ZDHHC11	chr5	837556	T	G_het	G_het	G_het	G_het
nonsynonymous SNV	ZDHHC11	chr5	843723	C	C	C	A_het	A_het
nonsynonymous SNV	ZDHHC11	chr5	843732	G	G	G	A_het	G
nonsynonymous SNV	ZDHHC18	chr1	27153585	C	C	C	A_hom	C
nonsynonymous SNV	ZDHHC23	chr3	113676683	G	G	G	C_hom	G
nonsynonymous SNV	ZDHHC3	chr3	44974433	A	A	C_hom	A	A
nonsynonymous SNV	ZFAT	chr8	135614253	G	G	A_het	G	G
nonsynonymous SNV	ZFHX2	chr14	23994091	C	C	T_het	C	C
nonsynonymous SNV	ZFHX2	chr14	23994199	C	C	T_het	C	C
nonsynonymous SNV	ZFHX3	chr16	72822645	G	A_het	G	G	G
nonsynonymous SNV	ZFHX3	chr16	72827363	C	G_het	C	C	C
stopgain	ZFHX4	chr8	77617020	C	C	T_het	C	C
nonsynonymous SNV	ZFP112	chr19	44833888	A	A	A	G_het	A
nonsynonymous SNV	ZFP30	chr19	38126838	T	T	T	T	C_het
nonsynonymous SNV	ZFP36L2	chr2	43452195	G	G	A_het	G	G
nonsynonymous SNV	ZFP36L2	chr2	43452757	A	A	A	A	C_het
nonsynonymous SNV	ZFP37	chr9	115812181	T	C_het	T	T	T
nonsynonymous SNV	ZFP69	chr1	40960863	T	T	T	T	C_het
nonsynonymous SNV	ZFP90	chr16	68597030	C	T_het	C	C	C
nonsynonymous SNV	ZFPL1	chr11	64853967	A	A	G_het	A	A

nonsynonymous SNV	ZFPM2	chr8	106814468	A	A	T_het	A	A
nonsynonymous SNV	ZFR2	chr19	3806120	C	C	C	T_het	C
nonsynonymous SNV	ZFR2	chr19	3823260	G	G	G	T_het	G
nonsynonymous SNV	ZFYVE1	chr14	73442294	C	C	T_het	C	C
nonsynonymous SNV	ZFYVE26	chr14	68215187	G	G	G	C_het	G
nonsynonymous SNV	ZFYVE27	chr10	99498266	C	C	C	T_het	C
nonsynonymous SNV	ZHX2	chr8	123965132	G	G	A_het	G	G
nonsynonymous SNV	ZIC4	chr3	147113821	G	G	C_het	G	G
nonsynonymous SNV	ZKSCAN1	chr7	99631584	G	G	A_het	G	G
nonsynonymous SNV	ZMYM1	chr1	35580382	C	C	C	C	T_het
nonsynonymous SNV	ZMYM4	chr1	35847342	A	A	G_het	A	A
nonsynonymous SNV	ZMYND8	chr20	45850015	C	C	T_het	C	C
nonsynonymous SNV	ZNF100	chr19	21910131	C	C	C	C	A_het
nonsynonymous SNV	ZNF135	chr19	58570637	G	G	A_het	G	G
nonsynonymous SNV	ZNF135	chr19	58578313	C	C	C	T_het	C
nonsynonymous SNV	ZNF135	chr19	58578497	A	A	A	G_het	A
nonsynonymous SNV	ZNF136	chr19	12297513	A	A	A	G_het	A
nonsynonymous SNV	ZNF141	chr4	338129	G	G	G	A_het	A_het
nonsynonymous SNV	ZNF141	chr4	338130	C	C	C	G_het	C
nonsynonymous SNV	ZNF141	chr4	338156	T	T	T	A_het	T
nonsynonymous SNV	ZNF141	chr4	338186	G	G	G	T_het	G
nonsynonymous SNV	ZNF141	chr4	338200	G	G	G	C_het	C_het
nonsynonymous SNV	ZNF141	chr4	338205	T	T	T	C_het	C_het
nonsynonymous SNV	ZNF141	chr4	367275	A	G_het	A	A	A
nonsynonymous SNV	ZNF142	chr2	219508604	G	G	A_het	G	G
nonsynonymous SNV	ZNF154	chr19	58214147	T	T	C_hom	T	T
nonsynonymous SNV	ZNF157	chrX	47271907	G	G	G	C_hom	G
nonsynonymous SNV	ZNF16	chr8	146157860	C	C	C	T_het	C
nonsynonymous SNV	ZNF181	chr19	35232200	T	T	T	T	G_het
nonsynonymous SNV	ZNF182	chrX	47842356	G	G	G	G	T_hom
nonsynonymous SNV	ZNF208	chr19	22154784	C	C	C	C	G_het
nonsynonymous SNV	ZNF208	chr19	22155630	G	G	G	C_het	G
nonsynonymous SNV	ZNF219	chr14	21560429	C	C	C	C	T_het
nonsynonymous SNV	ZNF22	chr10	45499336	A	A	C_het	A	A
nonsynonymous SNV	ZNF224	chr19	44610888	G	G	A_het	G	G
nonsynonymous SNV	ZNF232	chr17	5012846	G	G	A_hom	G	G
nonsynonymous SNV	ZNF235	chr19	44791447	T	T	A_het	T	T
nonsynonymous SNV	ZNF239	chr10	44053013	G	C_het	C_het	C_hom	C_het
nonsynonymous SNV	ZNF251	chr8	145948440	T	T	C_het	T	T
nonsynonymous SNV	ZNF252P	chr8	146203288	T	T	T	C_het	T
nonsynonymous SNV	ZNF254	chr19	24309080	A	A	G_het	A	A
nonsynonymous SNV	ZNF259	chr11	116652892	A	T_het	A	A	A
nonsynonymous SNV	ZNF266	chr19	9524511	T	T	A_het	T	T
nonsynonymous SNV	ZNF267	chr16	31927314	A	A	A	G_het	A
nonsynonymous SNV	ZNF28	chr19	53303246	G	G	G	C_het	G

nonsynonymous SNV	ZNF281	chr1	200378613	G	G	G	G	A_het
nonsynonymous SNV	ZNF286B	chr17	18584142	A	G_het	G_het	A	A
nonsynonymous SNV	ZNF302	chr19	35176039	A	A	A	G_het	A
nonsynonymous SNV	ZNF317	chr19	9271582	C	C	T_het	C	C
nonsynonymous SNV	ZNF324B	chr19	58966439	T	T	T	C_het	T
nonsynonymous SNV	ZNF324B	chr19	58966706	A	A	A	G_het	A
nonsynonymous SNV	ZNF333	chr19	14829589	G	G	G	T_het	G
nonsynonymous SNV	ZNF337	chr20	25655705	C	C	C	T_het	C
nonsynonymous SNV	ZNF341	chr20	32379012	G	G	A_het	G	G
nonsynonymous SNV	ZNF350	chr19	52471863	A	A	A	G_het	A
nonsynonymous SNV	ZNF366	chr5	71752361	T	T	C_het	T	T
nonsynonymous SNV	ZNF383	chr19	37733797	A	A	G_het	A	A
nonsynonymous SNV	ZNF391	chr6	27368874	T	T	T	T	C_het
nonsynonymous SNV	ZNF407	chr18	72344498	C	C	C	T_hom	C
nonsynonymous SNV	ZNF407	chr18	72347081	G	G	G	A_hom	G
nonsynonymous SNV	ZNF408	chr11	46722685	G	G	G	C_hom	G
nonsynonymous SNV	ZNF417	chr19	58420682	T	T	T	T	C_het
nonsynonymous SNV	ZNF418	chr19	58438116	C	C	C	C	T_het
nonsynonymous SNV	ZNF419	chr19	58002965	G	G	G	A_het	G
nonsynonymous SNV	ZNF425	chr7	148801170	G	G	G	A_het	G
stopgain	ZNF433	chr19	12126454	G	G	G	A_het	G
nonsynonymous SNV	ZNF438	chr10	31134047	T	T	T	C_het	T
nonsynonymous SNV	ZNF44	chr19	12383700	A	A	A	G_het	A
nonsynonymous SNV	ZNF441	chr19	11890983	G	G	G	A_het	G
nonsynonymous SNV	ZNF441	chr19	11891222	C	C	C	T_het	C
nonsynonymous SNV	ZNF443	chr19	12542561	G	A_het	G	G	G
nonsynonymous SNV	ZNF443	chr19	12543251	A	A	A	C_het	A
nonsynonymous SNV	ZNF462	chr9	109691559	G	G	A_het	G	G
nonsynonymous SNV	ZNF469	chr16	88497387	A	A	G_het	A	A
nonsynonymous SNV	ZNF469	chr16	88497738	C	C	T_het	C	C
nonsynonymous SNV	ZNF469	chr16	88500678	G	G	G	A_het	G
nonsynonymous SNV	ZNF469	chr16	88504204	G	G	C_het	G	G
nonsynonymous SNV	ZNF471	chr19	57037037	A	A	A	A	G_het
nonsynonymous SNV	ZNF479	chr7	57188019	A	A	A	A	T_het
nonsynonymous SNV	ZNF479	chr7	57193730	T	T	T	T	C_het
nonsynonymous SNV	ZNF486	chr19	20308207	A	T_het	A	A	A
nonsynonymous SNV	ZNF488	chr10	48370965	A	A	A	G_het	A
nonsynonymous SNV	ZNF492	chr19	22846697	T	T	C_het	T	T
nonsynonymous SNV	ZNF492	chr19	22846892	T	T	T	G_het	T
nonsynonymous SNV	ZNF497	chr19	58867634	C	C	G_het	C	C
nonsynonymous SNV	ZNF497	chr19	58867966	C	C	T_het	C	C
nonsynonymous SNV	ZNF497	chr19	58868716	G	G	G	C_het	G
nonsynonymous SNV	ZNF507	chr19	32845370	C	C	C	C	T_het
nonsynonymous SNV	ZNF511	chr10	135125245	G	G	G	T_het	G
nonsynonymous SNV	ZNF521	chr18	22806966	C	T_het	C	C	C

nonsynonymous SNV	ZNF525	chr19	53884455	T	C_het	T	T	T
nonsynonymous SNV	ZNF528	chr19	52919199	G	A_het	G	G	G
nonsynonymous SNV	ZNF534	chr19	52954716	G	G	G	A_het	G
nonsynonymous SNV	ZNF536	chr19	30935274	G	G	G	A_het	G
nonsynonymous SNV	ZNF546	chr19	40519797	A	C_het	A	A	A
nonsynonymous SNV	ZNF546	chr19	40520685	G	G	G	C_het	G
nonsynonymous SNV	ZNF549	chr19	58049034	G	A_het	G	G	G
nonsynonymous SNV	ZNF551	chr19	58198453	G	G	T_het	G	G
nonsynonymous SNV	ZNF554	chr19	2833943	G	G	A_het	G	G
nonsynonymous SNV	ZNF556	chr19	2877618	A	A	A	G_het	A
nonsynonymous SNV	ZNF557	chr19	7083010	G	G	G	G	A_het
nonsynonymous SNV	ZNF559	chr19	9453103	G	G	G	A_het	G
nonsynonymous SNV	ZNF563	chr19	12430462	G	A_het	G	G	G
nonsynonymous SNV	ZNF566	chr19	36940321	C	G_het	C	C	C
nonsynonymous SNV	ZNF57	chr19	2917920	C	C	T_het	C	C
nonsynonymous SNV	ZNF577	chr19	52376623	G	G	G	C_het	G
nonsynonymous SNV	ZNF608	chr5	123980164	T	C_hom	C_het	T	T
nonsynonymous SNV	ZNF615	chr19	52496486	G	G	G	C_het	G
nonsynonymous SNV	ZNF623	chr8	144732488	A	A	T_het	A	A
nonsynonymous SNV	ZNF629	chr16	30793517	C	C	A_het	C	C
nonsynonymous SNV	ZNF638	chr2	71651029	G	G	G	A_het	G
nonsynonymous SNV	ZNF648	chr1	182026052	G	C_het	G	G	G
nonsynonymous SNV	ZNF648	chr1	182026812	C	C	C	T_hom	C
nonsynonymous SNV	ZNF672	chr1	249142465	G	G	A_het	G	G
nonsynonymous SNV	ZNF701	chr19	53086675	C	C	C	T_het	C
nonsynonymous SNV	ZNF705A	chr12	8329652	A	G_het	G_het	A	G_hom
nonsynonymous SNV	ZNF705A	chr12	8329833	G	A_het	A_het	G	A_hom
nonsynonymous SNV	ZNF705G	chr8	7215694	A	G_het	G_het	G_hom	A
nonsynonymous SNV	ZNF705G	chr8	7217172	G	A_het	G	G	G
nonsynonymous SNV	ZNF705G	chr8	7217837	A	C_het	C_het	A	A
nonsynonymous SNV	ZNF705G	chr8	7218632	C	T_het	T_het	C	C
nonsynonymous SNV	ZNF705G	chr8	7218752	G	C_het	C_het	G	G
nonsynonymous SNV	ZNF708	chr19	21476107	T	C_het	T	T	T
nonsynonymous SNV	ZNF711	chrX	84526830	T	T	T	T	C_hom
nonsynonymous SNV	ZNF714	chr19	21300500	G	G	G	C_het	G
nonsynonymous SNV	ZNF717	chr3	75786662	A	T_het	A	T_het	A
nonsynonymous SNV	ZNF717	chr3	75786672	C	T_het	C	T_het	C
nonsynonymous SNV	ZNF717	chr3	75786681	G	G	G	A_het	G
nonsynonymous SNV	ZNF717	chr3	75786684	G	G	G	T_het	G
nonsynonymous SNV	ZNF717	chr3	75786737	A	T_het	T_hom	T_het	T_het
nonsynonymous SNV	ZNF717	chr3	75786748	C	T_het	T_hom	T_het	T_het
nonsynonymous SNV	ZNF717	chr3	75786888	C	T_het	T_hom	T_het	T_het
nonsynonymous SNV	ZNF717	chr3	75788105	C	C	C	T_het	C
nonsynonymous SNV	ZNF717	chr3	75788109	G	G	G	A_het	A_het
nonsynonymous SNV	ZNF717	chr3	75788130	C	C	C	T_het	T_het

nonsynonymous SNV	ZNF717	chr3	75788137	C	C	C	T_het	T_het
nonsynonymous SNV	ZNF717	chr3	75788195	A	A	A	T_het	A
nonsynonymous SNV	ZNF718	chr4	155655	C	G_het	C	C	C
nonsynonymous SNV	ZNF721	chr4	466424	C	C	C	A_het	C
nonsynonymous SNV	ZNF721	chr4	466427	G	G	G	T_het	G
nonsynonymous SNV	ZNF726	chr19	24115786	T	T	T	C_het	T
nonsynonymous SNV	ZNF728	chr19	23158869	A	A	A	G_het	A
nonsynonymous SNV	ZNF729	chr19	22497268	G	G	G	A_het	G
nonsynonymous SNV	ZNF737	chr19	20735307	T	T	T	C_het	T
nonsynonymous SNV	ZNF763	chr19	12089067	G	G	G	A_het	G
nonsynonymous SNV	ZNF77	chr19	2933747	C	C	C	T_het	C
nonsynonymous SNV	ZNF770	chr15	35273871	G	G	G	G	A_het
nonsynonymous SNV	ZNF770	chr15	35274665	C	C	G_het	C	C
nonsynonymous SNV	ZNF773	chr19	58016768	G	G	C_het	G	G
nonsynonymous SNV	ZNF780B	chr19	40541717	C	C	C	C	T_het
nonsynonymous SNV	ZNF781	chr19	38160565	C	C	T_het	C	C
nonsynonymous SNV	ZNF786	chr7	148769055	T	T	T	T	C_hom
nonsynonymous SNV	ZNF788	chr19	12204657	A	A	A	G_het	A
nonsynonymous SNV	ZNF788	chr19	12223703	G	G	G	T_het	G
nonsynonymous SNV	ZNF792	chr19	35448990	A	A	A	A	C_het
nonsynonymous SNV	ZNF799	chr19	12501374	T	T	C_het	T	T
stopgain	ZNF800	chr7	127014408	G	G	A_het	G	G
nonsynonymous SNV	ZNF814	chr19	58384719	T	T	T	T	C_het
nonsynonymous SNV	ZNF814	chr19	58385748	G	G	G	G	A_het
nonsynonymous SNV	ZNF816	chr19	53453883	G	A_het	G	G	G
nonsynonymous SNV	ZNF835	chr19	57175321	G	G	A_het	G	G
nonsynonymous SNV	ZNF837	chr19	58880486	C	C	T_het	C	C
nonsynonymous SNV	ZNF839	chr14	102792928	G	G	A_het	G	G
nonsynonymous SNV	ZNF839	chr14	102807818	T	T	T	T	C_het
nonsynonymous SNV	ZNF841	chr19	52570485	C	C	C	C	G_het
nonsynonymous SNV	ZNF845	chr19	53855861	A	A	A	G_het	A
nonsynonymous SNV	ZNF853	chr7	6660772	G	C_het	G	G	G
nonsynonymous SNV	ZNF853	chr7	6662039	C	C	T_het	C	C
nonsynonymous SNV	ZNF878	chr19	12155148	T	T	C_het	T	T
nonsynonymous SNV	ZNF879	chr5	178455119	G	G	A_het	G	G
nonsynonymous SNV	ZNF93	chr19	20044369	T	T	T	C_het	T
nonsynonymous SNV	ZNF98	chr19	22575777	T	T	T	C_het	T
nonsynonymous SNV	ZNF99	chr19	22952081	C	C	C	T_het	C
nonsynonymous SNV	ZNF99	chr19	22952084	C	C	C	T_het	C
nonsynonymous SNV	ZP3	chr7	76069902	G	G	C_het	G	G
nonsynonymous SNV	ZPLD1	chr3	102181222	G	G	A_het	G	G
nonsynonymous SNV	ZRSR2	chrX	15841063	C	G_het	C	C	C
nonsynonymous SNV	ZSCAN18	chr19	58596462	C	C	C	T_het	C
nonsynonymous SNV	ZSCAN18	chr19	58598329	C	C	T_het	C	C
nonsynonymous SNV	ZSCAN18	chr19	58601232	C	C	A_het	C	C

nonsynonymous SNV	ZSCAN29	chr15	43653578	A	A	A	A	G_het
nonsynonymous SNV	ZSCAN5B	chr19	56701639	C	C	C	C	G_het
nonsynonymous SNV	ZSWIM2	chr2	187709483	C	C	C	T_het	C
nonsynonymous SNV	ZSWIM4	chr19	13919761	A	G_het	A	A	A
