# Supplementary material for: The neuroendocrine phenotype, genomic profile and therapeutic sensitivity of GEPNET cell lines
Source: Endocr Relat Cancer. 2018 Jan 15;25(3):367–80. doi: 10.1530/ERC-17-0445 (PMC5827037; doi:10.1530/ERC-17-0445)
Supplement: Supplementary Table 3 [file erc-25-309-t003.pdf]

Supplementary Table 3. Filtered indel mutations.

Annotation	Gene	Chr	Position 1	Position 2	Ref	GOT1	P-STS	BON1	QGP-1
frameshift insertion	ABCA8	chr17	66924065	66924065	-	-	T_het	-	-
frameshift insertion	ABCC10	chr6	43409640	43409640	-	-	T_het	-	-
frameshift deletion	ABCD4	chr14	74754567	74754567	C	C	-_het	C	C
frameshift insertion	ABL2	chr1	179086419	179086419	-	A_het	-	-	-
frameshift deletion	ABL2	chr1	179086420	179086420	A	A	-_hom	A	A
frameshift insertion	ACAD9	chr3	128617302	128617302	-	-	-	CAGTGGCACGATCTTGGCTCACTGCAGC_hom	-
frameshift deletion	ACBD3	chr1	226352491	226352492	TT	TT	-_het	TT	TT
frameshift insertion	ACOT4	chr14	74060511	74060511	-	-	TCAA_hom	-	-
frameshift deletion	ACOT4	chr14	74060514	74060517	CTTA	CTTA	-_hom	CTTA	CTTA
frameshift deletion	ACVR2A	chr2	148683686	148683686	A	A	-_hom	A	A
frameshift deletion	ADAM11	chr17	42837126	42837126	G	G	-_het	G	G
frameshift deletion	ADAMTSL1	chr9	18826262	18826262	T	-_het	-_het	-_hom	-_hom
frameshift insertion	ADD3	chr10	111877100	111877100	-	-	-	-	T_het
frameshift deletion	ADD3	chr10	111893350	111893350	A	A	-_het	A	A

frameshift deletion	ADRA2B	chr2	96780921	96780921	G	G	-_het	G	G
frameshift insertion	AF035281	chr7	148991542	148991542	-	-	G_het	-	-
frameshift deletion	AFF3	chr2	100722134	100722134	T	T	-_het	T	T
frameshift deletion	AGAP6	chr10	51769223	51769236	GCAGTCATGCGAGA	GCAGTCATGCGAGA	GCAGTCATGCGAGA	GCAGTCATGCGAGA	-_het
frameshift deletion	AHNAK2	chr14	105404586	105404586	A	-_het	A	A	A
frameshift deletion	AHNAK2	chr14	105416448	105416448	G	G	-_het	G	G
frameshift deletion	AIM1L	chr1	26671523	26671535	AAATGAGGCATCA	AAATGAGGCATCA	AAATGAGGCATCA	-_hom	AAATGAGGCATCA
frameshift deletion	AIM1L	chr1	26671539	26671542	GCAC	GCAC	GCAC	-_hom	GCAC
frameshift deletion	AIM1L	chr1	26671545	26671578	GGGCCCTTCACGACCTCTTTCCAGGTGGGGAACA	GGGCCCTTCACGACCTCTTTCCAGGTGGGGAACA	GGGCCCTTCACGACCTCTTTCCAGGTGGGGAACA	-_hom	GGGCCCTTCACGACCTCTTTCCAGGTGGGGAACA
frameshift deletion	AIM2	chr1	159032487	159032487	T	T	-_hom	T	T
frameshift deletion	AK096249	chr9	138363457	138363458	AA	AA	-_het	AA	AA
frameshift deletion	AK097289	chr7	102063568	102063569	TT	TT	-_het	TT	TT
frameshift deletion	AK130759	chr15	101841426	101841426	C	C	-_het	C	C
frameshift deletion	AKAP11	chr13	42874486	42874486	A	A	-_het	A	A
frameshift insertion	AKAP3	chr12	4735965	4735965	-	-	-	C_het	C_het

frameshift deletion	AKAP3	chr12	4735970	4735970	A	A	A	-_het	-_het
frameshift insertion	ALKBH8	chr11	107422610	107422610	-	-	A_het	-	-
frameshift deletion	ANGEL2	chr1	213180442	213180443	AA	AA	-_hom	AA	AA
frameshift deletion	ANGPTL3	chr1	63064446	63064446	A	A	A	-_hom	A
frameshift deletion	ANHX	chr12	133803561	133803561	G	G	-_het	G	G
frameshift deletion	ANK3	chr10	62149221	62149221	T	T	-_het	T	T
frameshift deletion	ANKLE1	chr19	17397494	17397501	TGTGTGTT	TGTGTGTT	-_hom	TGTGTGTT	TGTGTGTT
frameshift deletion	ANKRD20A3	chr9	67966645	67966645	A	A	-_hom	A	A
frameshift deletion	ANKRD26	chr10	27322259	27322259	T	T	-_het	T	T
frameshift deletion	ANKRD34B	chr5	79854592	79854592	G	G	-_het	G	G
frameshift deletion	ANKRD36	chr2	97818261	97818262	GC	-_het	-_het	-_het	-_het
frameshift insertion	ANKRD36	chr2	97818264	97818264	-	TT_het	TT_het	TT_het	TT_het
frameshift deletion	ANKRD36	chr2	97851074	97851074	C	-_het	C	C	C
frameshift insertion	ANKRD36	chr2	97851078	97851078	-	G_het	-	-	-
frameshift insertion	ANKRD44	chr2	197943382	197943382	-	-	TG_het	-	-
stopgain	ANKUB1	chr3	149485819	149485819	-	-	-	CATT_hom	-
frameshift deletion	ANLN	chr7	36462267	36462271	TGAAT	-_het	TGAAT	TGAAT	TGAAT

frameshift deletion	ANO8	chr19	17439380	17439380	C	C	-_het	C	C
frameshift deletion	APPL2	chr12	105570678	105570678	A	A	-_het	A	A
frameshift deletion	AQP7	chr9	33385690	33385690	G	G	-_het	G	G
frameshift deletion	ARHGAP5	chr14	32561191	32561191	A	A	-_het	A	A
frameshift deletion	ARHGAP5	chr14	32562675	32562675	T	T	-_het	T	T
frameshift insertion	ARHGEF5	chr7	144059763	144059763	-	-	TGGAGGCTGAGGAGGCCCAGCG_het	TGGAGGCTGAGGAGGCCCAGCG_het	-
frameshift deletion	ARID1A	chr1	27106074	27106074	G	G	-_het	G	G
frameshift deletion	ARSA	chr22	51065757	51065757	C	C	-_het	C	C
frameshift deletion	ASPSCR1	chr17	79974910	79974910	C	C	-_het	C	C
frameshift deletion	ASTE1	chr3	130733047	130733048	TT	TT	-_hom	TT	TT
frameshift deletion	ATAD2	chr8	124384893	124384893	T	T	-_het	T	T
frameshift deletion	ATG9A	chr2	220085910	220085910	G	G	-_het	G	G
frameshift deletion	ATP13A3	chr3	194140669	194140669	A	A	-_het	A	A
frameshift insertion	ATP13A3	chr3	194147849	194147849	-	-	A_het	-	-
frameshift deletion	ATP5J2-PTCD1	chr7	99027358	99027358	G	G	-_het	G	G
frameshift	ATP6AP1	chrX	153660772	153660773	CT	CT	-_het	CT	CT

deletion									
frameshift deletion	ATP8B3	chr19	1799764	1799764	A	-_het	A	A	-_het
frameshift deletion	ATP8B4	chr15	50189465	50189465	A	A	-_het	A	A
frameshift deletion	ATR	chr3	142274740	142274740	T	T	-_het	T	T
frameshift deletion	AX746750	chr10	104214081	104214082	TT	TT	-_hom	TT	TT
frameshift deletion	AX747192	chr11	63997567	63997568	AA	AA	AA	AA	-_het
frameshift deletion	AX747828	chr3	196159011	196159011	G	G	-_het	G	G
frameshift deletion	AX747968	chr15	33010583	33010583	A	A	-_het	A	A
frameshift deletion	AX748283	chr1	19675264	19675264	C	C	C	C	-_hom
frameshift deletion	AXIN1	chr16	360055	360055	G	G	-_het	G	G
frameshift deletion	AXIN2	chr17	63533905	63533905	C	C	-_het	C	C
frameshift insertion	AXIN2	chr17	63545677	63545677	-	-	C_het	-	-
frameshift insertion	AXL	chr19	41743932	41743932	-	-	C_het	-	-
frameshift deletion	BACH1	chr21	30698479	30698479	T	T	-_het	T	T
frameshift deletion	BAHD1	chr15	40750958	40750958	C	C	-_het	C	C
frameshift deletion	BAI1	chr8	143546245	143546245	C	C	-_het	C	C
frameshift deletion	BAI2	chr1	32201169	32201169	C	C	-_het	C	C

frameshift deletion	BANK1	chr4	102951317	102951317	A	A	-_het	A	A
frameshift insertion	BC031638	chr21	47744121	47744121	-	-	C_het	-	-
frameshift deletion	BC033456	chr17	48258285	48258288	AAAA	AAAA	-_hom	AAAA	AAAA
frameshift deletion	BC046483	chr10	1095423	1095427	GAGTT	GAGTT	GAGTT	-_het	GAGTT
frameshift insertion	BCAN	chr1	156617346	156617346	-	-	T_het	-	-
frameshift deletion	BCAS3	chr17	59465981	59465981	A	A	-_hom	A	A
frameshift deletion	BCO2	chr11	112046328	112046328	T	T	-_het	T	T
frameshift deletion	BCORL1	chrX	129190011	129190011	C	C	-_hom	C	C
frameshift deletion	BDP1	chr5	70849097	70849097	A	A	-_het	A	A
frameshift insertion	BICC1	chr10	60558976	60558976	-	-	A_het	-	-
frameshift insertion	BIRC7	chr20	61870886	61870886	-	-	C_het	-	-
frameshift insertion	bK250D10.C22.8	chr22	42354487	42354487	-	-	-	GAGAGAGA_hom	-
frameshift deletion	bK250D10.C22.8	chr22	42354488	42354489	GA	GA	-_het	GA	GA
frameshift deletion	BLM	chr15	91304139	91304139	A	A	-_het	A	A
stopgain	BOD1	chr5	173036437	173036437	-	-	-	TGAACCACACTCTGCCTCAAACCATTTCGCAATTGGTTTTTGTT_het	-

frameshift insertion	BOK-AS1	chr2	242498225	242498225	-	-	-	C_het	-
frameshift deletion	BRD8	chr5	137505021	137505021	G	G	-_het	G	G
frameshift deletion	BRD9	chr5	889777	889777	A	A	-_hom	A	A
frameshift insertion	BRSK1	chr19	55815035	55815035	-	-	-	-	C_het
frameshift deletion	BTAF1	chr10	93756247	93756247	T	T	-_het	T	T
frameshift insertion	BTF3	chr5	72795052	72795052	-	-	-	G_het	-
frameshift insertion	BTF3	chr5	72795055	72795055	-	-	-	T_het	T_het
frameshift deletion	BTF3	chr5	72795056	72795056	T	T	-_het	T	T
frameshift deletion	C11orf16	chr11	8947465	8947555	GTGATTGGCCCTAGCAGAGAGCAGCAAGGGGCCCAGTGAAGGGGCCTGGGGTGCTCCCTGGTGAAAGACTTGTGCAGCCTCTCCACAGCCT	GTGATTGGCCCTAGCAGAGAGCAGCAAGGGGCCCAGTGAAGGGGCCTGGGGTGCTCCCTGGTGAAAGACTTGTGCAGCCTCTCCACAGCCT	-_het	GTGATTGGCCCTAGCAGAGAGCAGCAAGGGGCCCAGTGAAGGGGCCTGGGGTGCTCCCTGGTGAAAGACTTGTGCAGCCTCTCCACAGCCT	GTGATTGGCCCTAGCAGAGAGCAGCAAGGGGCCCAGTGAAGGGGCCTGGGGTGCTCCCTGGTGAAAGACTTGTGCAGCCTCTCCACAGCCT
frameshift insertion	C11orf40	chr11	4592706	4592706	-	AC_het	-	AC_hom	AC_het
frameshift insertion	C11orf40	chr11	4592708	4592708	-	-	AG_het	-	AG_het
frameshift deletion	C11orf40	chr11	4592711	4592711	T	T	-_het	T	T
frameshift deletion	C12orf68	chr12	48578410	48578410	G	G	-_het	G	G

frameshift insertion	C14orf23	chr14	29261307	29261307	-	C_het	-	C_het	-
frameshift insertion	C14orf23	chr14	29261309	29261309	-	AC_het	-	-	-
frameshift insertion	C17orf76-AS1	chr17	16343005	16343005	-	-	GGTA_het	-	-
frameshift insertion	C1orf127	chr1	11007946	11007946	-	-	C_het	-	-
frameshift deletion	C1S	chr12	7173239	7173239	A	A	-_het	A	A
frameshift deletion	C20orf96	chr20	271226	271228	ACC	ACC	ACC	-_het	ACC
frameshift deletion	C20orf96	chr20	271230	271231	AC	AC	AC	-_het	AC
frameshift insertion	C21orf2	chr21	45759044	45759044	-	TGCACGCTGTG CAGCT_het	-	-	-
frameshift deletion	C3orf18	chr3	50596381	50596381	G	G	-_hom	G	G
frameshift deletion	C4orf17	chr4	100451114	100451117	AAGT	AAGT	-_het	AAGT	AAGT
frameshift deletion	C7orf41	chr7	30197146	30197146	G	G	-_het	G	G
frameshift deletion	C9orf91	chr9	117379521	117379521	C	C	-_het	C	C
frameshift deletion	CACNB4	chr2	152709963	152709963	G	G	-_het	G	G
frameshift deletion	CACYBP	chr1	174976327	174976327	A	A	-_het	A	A
frameshift deletion	CAMSAP1	chr9	138774701	138774701	G	G	-_het	G	G
frameshift deletion	CANX	chr5	179149920	179149920	T	T	-_het	T	T

frameshift insertion	CAPN2	chr1	223900575	223900575	-	CACGGTAGGAA GCG_hom	-	-	-
frameshift deletion	CARD10	chr22	37887168	37887168	G	G	-_het	G	G
frameshift deletion	CASKIN2	chr17	73499267	73499267	C	C	-_het	C	C
frameshift deletion	CASP5	chr11	104874011	104874011	T	T	-_het	T	T
frameshift deletion	CASP5	chr11	104879687	104879687	T	T	-_het	T	T
frameshift insertion	CCDC136	chr7	128455758	128455758	-	-	A_het	-	-
frameshift deletion	CCDC137	chr17	79639624	79639625	GA	GA	-_het	GA	GA
frameshift deletion	CCDC150	chr2	197531519	197531520	AA	AA	-_het	AA	AA
frameshift deletion	CCDC168	chr13	103381996	103381996	A	A	-_het	A	A
frameshift deletion	CCDC28A	chr6	139097330	139097330	A	A	-_het	A	A
frameshift deletion	CCDC67	chr11	93148301	93148301	T	T	-_het	T	T
frameshift insertion	CCDC7	chr10	32740798	32740798	-	T_het	-	-	-
frameshift deletion	CCDC9	chr19	47768151	47768151	G	G	-_het	G	G
frameshift insertion	CD109	chr6	74495111	74495111	-	-	T_het	-	-
frameshift deletion	CD177	chr19	43865320	43865321	CT	-_het	-_hom	CT	-_het
frameshift insertion	CD177	chr19	43865324	43865324	-	AG_het	AG_hom	-	AG_het

frameshift deletion	CD36	chr7	80285889	80285889	A	A	A	-_het	A
frameshift deletion	CDC20B	chr5	54468432	54468436	CTTCT	CTTCT	CTTCT	-_het	CTTCT
frameshift deletion	CDC27	chr17	45234407	45234407	A	A	-_het	A	A
frameshift deletion	CDC7	chr1	91967357	91967357	A	A	-_het	A	A
frameshift deletion	CDK13	chr7	39990762	39990762	G	G	-_het	G	G
frameshift deletion	CDK20	chr9	90582463	90582463	G	G	-_het	G	G
frameshift deletion	CELA1	chr12	51740408	51740411	ATAA	-_het	-_het	ATAA	-_hom
frameshift insertion	CELA1	chr12	51740415	51740415	-	AG_het	AG_het	-	AG_hom
frameshift insertion	CELA1	chr12	51740416	51740416	-	G_het	G_het	-	G_hom
frameshift deletion	CELSR1	chr22	46793728	46793728	C	C	-_het	C	C
frameshift deletion	CELSR1	chr22	46931227	46931227	C	C	-_het	C	C
frameshift deletion	CENPE	chr4	104030036	104030036	A	A	-_het	A	A
frameshift deletion	CEP290	chr12	88524079	88524079	T	T	-_het	T	T
frameshift deletion	CETP	chr16	57007278	57007278	C	C	-_het	C	C
frameshift deletion	CHD8	chr14	21859176	21859176	T	T	-_het	T	T
frameshift insertion	CHEK1	chr11	125497710	125497710	-	-	T_het	-	-
frameshift	CHRM3	chr1	240072444	240072444	A	A	-_het	A	A

deletion									
stopgain	CLOCK	chr4	56336954	56336954	A	A	-_het	A	A
frameshift deletion	CMA1	chr14	24975317	24975317	G	G	-_het	G	G
frameshift insertion	CMTM1	chr16	66600730	66600730	-	-	-	-	AAATGAGATGGCGATC_het
frameshift deletion	CNOT1	chr16	58577316	58577316	A	A	A	-_het	-_het
frameshift deletion	CNTNAP3B	chr9	43853584	43853584	T	T	T	T	-_het
frameshift deletion	COIL	chr17	55028118	55028118	T	T	-_het	T	T
frameshift deletion	COL7A1	chr3	48612871	48612871	G	G	-_hom	G	G
frameshift deletion	COLEC12	chr18	331763	331763	T	T	-_het	T	T
frameshift deletion	CORO1B	chr11	67209168	67209168	G	G	-_hom	G	G
frameshift deletion	CREBBP	chr16	3781421	3781421	G	G	-_het	G	G
frameshift insertion	CREBBP	chr16	3807363	3807363	-	-	G_het	-	-
frameshift insertion	CRIPAK	chr4	1388350	1388350	-	TGCCCATGTGGAGTGCCCGCCTGCTCACACA_hom	TGCCCATGTGGAGTGCCCGCCTGCTCACACA_het	-	-
frameshift deletion	CRIPAK	chr4	1388594	1388622	CACGTGCCCATGTGGAGTGCCCGCCTGCT	-_hom	-_het	CACGTGCCCATGTGGAGTGCCCGCCTGCT	CACGTGCCCATGTGGAGTGCCCGCCTGCT
frameshift deletion	CRIPAK	chr4	1388944	1388947	GTGG	GTGG	GTGG	-_hom	GTGG

frameshift deletion	CRIPAK	chr4	1388949	1389037	GTGTTCGCCTGCTCACACGTGCCGATGCGGAGTGCCCGCCTGCTCACACGTGCCGATGCGGAGTGCCCGCCTGCTCACACGTGCCCATG	GTGTTCGCCTGCTCACACGTGCCGATGCGGAGTGCCCGCCTGCTCACACGTGCCGATGCGGAGTGCCCGCCTGCTCACACGTGCCCATG	GTGTTCGCCTGCTCACACGTGCCGATGCGGAGTGCCCGCCTGCTCACACGTGCCGATGCGGAGTGCCCGCCTGCTCACACGTGCCCATG	-_hom	GTGTTCGCCTGCTCACACGTGCCGATGCGGAGTGCCCGCCTGCTCACACGTGCCGATGCGGAGTGCCCGCCTGCTCACACGTGCCCATG
frameshift deletion	CROCC	chr1	17287578	17287578	C	C	-_hom	C	C
frameshift deletion	CSMD3	chr8	113241029	113241029	A	A	-_het	A	A
frameshift deletion	CSNK1E	chr22	38699188	38699188	G	G	-_het	G	G
frameshift deletion	CSPG4	chr15	75981842	75981854	CCTCCAGCACCAG	CCTCCAGCACCAG	CCTCCAGCACCAG	-_het	CCTCCAGCACCAG
frameshift deletion	CTAGE1	chr18	19995706	19995706	G	G	-_het	G	G
frameshift deletion	CTAGE10P	chr13	50467039	50467039	T	T	T	-_het	-_het
frameshift deletion	CTCF	chr16	67645339	67645339	A	A	-_het	A	A
frameshift deletion	CYP4F3	chr19	15770184	15770184	C	C	C	-_het	C
frameshift deletion	DAZAP1	chr19	1430254	1430254	C	C	-_het	C	C
frameshift deletion	DAZAP1	chr19	1434830	1434830	G	G	-_het	G	G
frameshift insertion	DCHS1	chr11	6649949	6649949	-	-	AG_het	-	-
frameshift deletion	DCHS2	chr4	155244402	155244405	TTTG	-_hom	-_hom	TTTG	-_hom

frameshift deletion	DDX17	chr22	38897211	38897211	T	T	-_het	T	T
frameshift deletion	DDX27	chr20	47858504	47858505	AA	AA	-_het	AA	AA
frameshift deletion	DECR2	chr16	460352	460388	TGAGAAGTTCTTCCGGGTGGGTGCCTCGTGCGCTCTG	-_hom	TGAGAAGTTCTTCCGGGTGGGTGCCTCGTGCGCTCTG	TGAGAAGTTCTTCCGGGTGGGTGCCTCGTGCGCTCTG	TGAGAAGTTCTTCCGGGTGGGTGCCTCGTGCGCTCTG
frameshift deletion	DEFB126	chr20	126156	126159	CAAA	-_het	-_hom	CAAA	-_het
frameshift deletion	DEFB126	chr20	126311	126312	CC	-_het	-_hom	CC	-_het
frameshift deletion	DENND1C	chr19	6468936	6468936	C	C	-_het	C	C
frameshift insertion	DENND5A	chr11	9171678	9171678	-	-	T_het	-	-
frameshift insertion	DGKK	chrX	50121146	50121146	-	G_hom	G_hom	G_hom	G_hom
frameshift deletion	DHFR	chr5	79950390	79950390	C	C	C	-_het	C
frameshift deletion	DHODH	chr16	72055148	72055170	CCCAACACTGCCGGGCTGCGGAG	CCCAACACTGCCGGGCTGCGGAG	CCCAACACTGCCGGGCTGCGGAG	CCCAACACTGCCGGGCTGCGGAG	-_het
frameshift insertion	DKFZp667P0924	chr2	96525717	96525717	-	-	T_het	T_het	-
frameshift deletion	DKFZp761D112	chr8	89046467	89046471	AAAAA	AAAAA	-_het	AAAAA	AAAAA
frameshift deletion	DLAT	chr11	111904183	111904183	A	A	-_het	A	A
frameshift deletion	DLGAP3	chr1	35365852	35365852	C	C	-_het	C	C
frameshift	DLL4	chr15	41222270	41222270	G	G	-_het	G	G

deletion									
frameshift deletion	DMXL1	chr5	118502316	118502316	A	A	-_het	A	A
frameshift insertion	DNAH12	chr3	57509312	57509312	-	-	TT_hom	-	-
frameshift insertion	DNAH14	chr1	225328438	225328438	-	-	-	-	AT_het
frameshift deletion	DNAH14	chr1	225458505	225458508	CAAA	CAAA	CAAA	-_hom	CAAA
frameshift insertion	DNAH14	chr1	225533751	225533751	-	-	A_het	-	-
frameshift insertion	DNAH2	chr17	7667348	7667348	-	AGTGC_het	-	-	-
frameshift deletion	DNAH5	chr5	13701426	13701426	A	A	-_het	A	A
frameshift deletion	DNAJB9	chr7	108213668	108213668	T	T	-_het	T	T
frameshift deletion	DNM1P41	chr15	85046529	85046529	G	G	-_het	G	G
frameshift deletion	DNM1P41	chr15	85046531	85046531	G	G	-_het	G	G
frameshift deletion	DNM1P41	chr15	85046591	85046594	GTGT	GTGT	GTGT	GTGT	-_het
frameshift insertion	DNM1P46	chr15	100340348	100340348	-	-	ATGTA_het	-	-
frameshift insertion	DNM2	chr19	10940881	10940881	-	-	C_het	-	-
frameshift deletion	DOCK3	chr3	51417604	51417604	C	C	-_hom	C	C
frameshift deletion	DOCK8	chr9	405015	405015	T	T	-_het	T	T
frameshift insertion	DPP10	chr2	116534868	116534868	-	-	A_het	-	-

frameshift deletion	DQ583205	chr16	89299877	89299899	TGCAACTCAACTCACACTGCGTA	-_hom	-_hom	-_hom	-_hom
frameshift deletion	DST	chr6	56476324	56476324	A	A	-_het	A	A
frameshift deletion	DUOX2	chr15	45393426	45393429	GAAC	GAAC	-_het	GAAC	GAAC
frameshift insertion	DYNC1I2	chr2	172546761	172546761	-	-	A_het	-	-
frameshift deletion	DYRK2	chr12	68052179	68052179	A	A	-_het	A	A
frameshift insertion	EIF3L	chr22	38274022	38274022	-	-	A_het	-	-
frameshift insertion	EIF5AL1	chr10	81272772	81272772	-	-	-	CC_het	-
frameshift deletion	EIF5AL1	chr10	81272776	81272776	A	A	A	-_het	A
frameshift deletion	EIF5AL1	chr10	81272779	81272780	AG	AG	AG	-_het	AG
frameshift insertion	ELK3	chr12	96641028	96641028	-	-	C_het	-	-
frameshift insertion	ELMSAN1	chr14	74205772	74205772	-	-	G_het	-	-
frameshift deletion	EMC10	chr19	50985132	50985132	G	G	-_het	G	G
frameshift deletion	EMC8	chr16	85832772	85832772	G	G	-_het	G	G
frameshift insertion	EML6	chr2	55176076	55176076	-	-	A_het	-	-
frameshift deletion	ENO4	chr10	118618628	118618628	A	A	-_het	A	A
frameshift deletion	ENTHD2	chr17	79202956	79202956	T	T	-_het	T	T

frameshift deletion	ENTPD2	chr9	139945517	139945517	C	C	-_het	C	C
frameshift deletion	EPB41L1	chr20	34793924	34793925	GA	-_hom	GA	GA	GA
frameshift deletion	EPHB2	chr1	23240246	23240246	A	A	-_het	A	A
frameshift insertion	EPHB6	chr7	142562308	142562308	-	-	G_het	-	-
frameshift deletion	EPPK1	chr8	144946136	144946139	TGAG	TGAG	TGAG	-_het	TGAG
frameshift deletion	ERAP2	chr5	96222415	96222415	A	A	-_het	A	A
frameshift insertion	EXO1	chr1	242042051	242042051	-	-	T_het	-	-
frameshift deletion	F13A1	chr6	6175046	6175046	T	T	-_het	T	T
frameshift deletion	F8	chrX	154157686	154157686	T	T	-_hom	T	T
frameshift insertion	FAM109B	chr22	42473376	42473376	-	-	G_het	-	-
frameshift deletion	FAM117A	chr17	47793566	47793566	G	G	-_het	G	G
frameshift insertion	FAM131A	chr3	184056454	184056454	-	-	-	AGGCCGCCCCCGTGCCGGT_hom	-
frameshift insertion	FAM151A	chr1	55081756	55081756	-	-	G_het	-	-
frameshift deletion	FAM153B	chr5	175535666	175535667	AG	AG	-_het	AG	AG

frameshift insertion	FAM186A	chr12	50745863	50745863	-	-	CTGCTGAGGGGTGAGAGGGATCCCCAGGGCCTGG_het	-	-
frameshift deletion	FAM189A1	chr15	29429273	29429273	G	G	-_het	G	G
frameshift deletion	FAM193A	chr4	2661593	2661593	C	C	-_het	C	C
stopgain	FAM20C	chr7	286468	286468	-	-	GACAGGTGAGCCCTTCCTTCCTCCCTCCATCCGC_het	GACAGGTGAGCCCTTCCTTCCTCCCTCCATCCGC_het	-
frameshift insertion	FAM217A	chr6	4070141	4070141	-	-	-	T_hom	-
frameshift deletion	FAM228B	chr2	24360917	24360917	A	A	-_het	A	A
frameshift deletion	FAM9A	chrX	8764320	8764320	T	T	-_hom	T	T
frameshift insertion	FBN3	chr19	8193948	8193948	-	-	G_het	-	-
frameshift deletion	FBRSL1	chr12	133146935	133146935	C	C	-_het	C	C
frameshift deletion	FBXO11	chr2	48050368	48050368	T	T	-_het	T	T
frameshift deletion	FER1L5	chr2	97364482	97364483	AG	AG	-_het	AG	AG
stopgain	FFAR3	chr19	35863285	35863285	-	-	-	CCTGTGCTGAAAACTAGGTCCTCCGGGGGAGGAGGGTGTAGCTGGCGTGTCATCCTCAGGGCGCT	-

								TC_het	
frameshift deletion	FGF13	chrX	137939823	137939823	G	G	-_hom	G	G
frameshift deletion	FGF22	chr19	643528	643528	G	G	-_het	G	G
frameshift deletion	FGGY	chr1	59812017	59812017	G	G	-_het	G	G
frameshift deletion	FIP1L1	chr4	54319248	54319249	AG	AG	-_het	AG	AG
frameshift insertion	FKBP10	chr17	39975558	39975558	-	-	C_het	-	-
frameshift deletion	FLJ00382	chr14	106329450	106329451	TG	-_hom	-_hom	-_het	-_hom
frameshift deletion	FLNC	chr7	128486941	128486941	G	G	-_het	G	G
frameshift deletion	FNIP2	chr4	159754866	159754866	T	T	-_het	T	T
frameshift insertion	FOXO3	chr6	108985176	108985176	-	-	-	-	G_het
frameshift deletion	FOXP2	chr7	114269649	114269649	T	T	-_het	T	T
frameshift insertion	FRG1B	chr20	29632674	29632674	-	A_het	A_het	A_het	A_het
frameshift deletion	FRYL	chr4	48636435	48636435	T	T	-_het	T	T
frameshift deletion	FUT8-AS1	chr14	65878848	65878848	G	G	-_het	G	G

frameshift deletion	FXR2	chr17	7496122	7496122	G	G	-_het	G	G
frameshift deletion	GABRE	chrX	151130987	151130987	A	A	-_hom	A	A
frameshift deletion	GBP3	chr1	89473442	89473442	T	T	-_het	T	T
frameshift insertion	GIGYF1	chr7	100285169	100285169	-	-	G_het	-	-
frameshift deletion	GJA4	chr1	35260764	35260764	A	A	-_het	A	A
frameshift deletion	GLS	chr2	191819456	191819456	T	T	-_het	T	T
frameshift deletion	GLTSCR1	chr19	48197891	48197891	C	C	-_het	C	C
frameshift deletion	GMPS	chr3	155621685	155621685	A	A	-_het	A	A
frameshift deletion	GNPNAT1	chr14	53250196	53250196	A	A	-_het	A	A
frameshift deletion	GOLGA8CP	chr15	20777480	20777480	G	G	-_het	G	G
frameshift insertion	GOLGB1	chr3	121409850	121409850	-	-	TC_het	-	-
frameshift deletion	GOSR1	chr17	28811835	28811835	T	T	-_het	T	T
frameshift insertion	GP6	chr19	55526103	55526103	-	-	-	-	CAGA_het
frameshift deletion	GPR124	chr8	37691605	37691605	G	G	-_het	G	G
frameshift deletion	GPR19	chr12	12814274	12814274	T	T	-_het	T	T
frameshift insertion	GPRC6A	chr6	117113762	117113762	-	-	-	A_hom	-

frameshift deletion	GPRIN1	chr5	176026122	176026134	CAAAGACCCAGGA	CAAAGACCCAGGA	-_het	CAAAGACCCAGGA	CAAAGACCCAGGA
frameshift deletion	GPRIN1	chr5	176026136	176026146	CCTCCTTCCTC	CCTCCTTCCTC	-_het	CCTCCTTCCTC	CCTCCTTCCTC
frameshift deletion	GRP	chr18	56892797	56892798	GA	GA	-_het	GA	GA
frameshift insertion	GSDMD	chr8	144642151	144642151	-	-	-	-	AGGGC_hom
frameshift deletion	GSDMD	chr8	144642152	144642156	AGGGC	AGGGC	AGGGC	-_het	AGGGC
frameshift deletion	GUF1	chr4	44700615	44700615	A	A	-_het	A	A
frameshift deletion	HAND2	chr4	174448221	174448221	T	T	-_het	T	T
frameshift insertion	HBS1L	chr6	135357713	135357713	-	-	-	T_hom	-
frameshift insertion	HCFC2	chr12	104473346	104473346	-	-	A_het	-	-
frameshift deletion	HCN4	chr15	73615394	73615394	C	C	-_het	C	C
frameshift deletion	HECTD3	chr1	45475782	45475782	G	G	-_het	G	G
frameshift deletion	HERC2	chr15	28518115	28518115	C	-_het	-_het	-_het	-_het
frameshift insertion	HERC6	chr4	89311864	89311864	-	-	C_het	-	-
frameshift insertion	HHEX	chr10	94449930	94449930	-	-	C_het	-	-
frameshift insertion	HIC2	chr22	21799658	21799658	-	-	C_het	-	-
frameshift deletion	HIF1A	chr14	62164608	62164608	T	T	T	-_het	T

frameshift insertion	HILPDA	chr7	128097489	128097489	-	-	A_het	-	-
frameshift deletion	HIRA	chr22	19346949	19346949	C	C	-_het	C	C
frameshift deletion	HIST2H2BE	chr1	149858153	149858153	T	T	-_het	T	T
frameshift insertion	HLA-B	chr6	31324488	31324488	-	-	-	-	GGAG_hom
frameshift deletion	HLA-B	chr6	31324493	31324493	C	C	C	C	-_hom
frameshift insertion	HLA-DRB1	chr6	32551955	32551955	-	-	CT_het	-	-
frameshift deletion	HLA-DRB1	chr6	32551961	32551962	GC	GC	-_het	GC	GC
frameshift deletion	HMGXB4	chr22	35661544	35661544	A	A	-_het	A	A
frameshift deletion	HNF1A	chr12	121432115	121432115	G	G	-_het	G	G
frameshift insertion	HNF1A	chr12	121434630	121434630	-	TCATTCAT_hom	TCATTCAT_hom	TCATTCAT_hom	TCATTCAT_het
frameshift deletion	HOMEZ	chr14	23744823	23744823	A	A	-_het	A	A
frameshift deletion	HOMEZ	chr14	23744825	23744829	CTTCC	CTTCC	-_het	CTTCC	CTTCC
frameshift insertion	HPS5	chr11	18330507	18330507	-	-	A_het	-	-
frameshift deletion	HTR4	chr5	147861099	147861099	T	T	-_het	T	T
frameshift deletion	HTT	chr4	3076659	3076660	AG	AG	AG	-_hom	AG
frameshift deletion	HTT	chr4	3076663	3076666	ACAG	ACAG	ACAG	-_hom	ACAG
frameshift	ICA1	chr7	8198251	8198251	T	T	-_het	T	T

deletion									
frameshift deletion	ICK	chr6	52880876	52880876	A	A	A	-_hom	A
frameshift deletion	IFRD1	chr7	112107983	112107986	GGTC	GGTC	-_het	GGTC	GGTC
frameshift insertion	IFRD1	chr7	112107986	112107986	-	-	AA_het	-	-
frameshift deletion	IGF2BP3	chr7	23385596	23385596	T	T	-_het	T	T
frameshift deletion	IGFN1	chr1	201177513	201177513	G	G	-_het	G	G
frameshift deletion	IK	chr5	140032593	140032594	GA	GA	-_het	GA	GA
frameshift deletion	IL10RB	chr21	34652203	34652203	A	A	-_het	A	A
frameshift insertion	IL32	chr16	3119297	3119297	-	G_het	G_het	-	G_het
frameshift deletion	IMPDH1	chr7	128049849	128049849	T	T	T	T	-_het
frameshift deletion	INO80D	chr2	206874449	206874449	T	T	-_het	T	T
frameshift deletion	INO80D	chr2	206882406	206882406	T	T	-_het	T	T
frameshift deletion	INVS	chr9	103054983	103054983	G	G	-_het	G	G
frameshift deletion	ITPR2	chr12	26589233	26589233	C	C	-_het	C	C
frameshift deletion	ITSN1	chr21	35122567	35122567	C	C	-_het	C	C
frameshift deletion	JPH4	chr14	24040436	24040436	C	C	-_het	C	C
frameshift insertion	KCNAB1	chr3	156241685	156241685	-	-	T_het	-	-

frameshift deletion	KCNC1	chr11	17794004	17794004	A	A	-_het	A	A
frameshift insertion	KCNN3	chr1	154842105	154842105	-	-	G_het	-	-
frameshift deletion	KDM5A	chr12	416953	416953	T	T	-_het	T	T
frameshift deletion	KHNYN	chr14	24900929	24900930	CT	CT	-_het	CT	CT
frameshift deletion	KIAA0040	chr1	175129946	175129946	C	C	-_het	-_hom	C
frameshift deletion	KIAA0040	chr1	175129948	175129955	TCTTCTTG	TCTTCTTG	-_het	-_hom	TCTTCTTG
frameshift insertion	KIAA0100	chr17	26962586	26962586	-	-	G_het	-	-
frameshift insertion	KIAA0913	chr10	75545602	75545602	-	-	G_het	-	-
frameshift deletion	KIAA1009	chr6	84896233	84896233	A	A	-_het	A	A
frameshift deletion	KIAA1468	chr18	59942747	59942747	T	T	-_hom	T	T
frameshift deletion	KIAA2018	chr3	113377482	113377482	T	T	-_het	T	T
frameshift deletion	KIF20B	chr10	91469743	91469743	A	A	-_het	A	A
frameshift deletion	KIRREL2	chr19	36357214	36357214	C	C	-_het	C	C
frameshift insertion	KLC3	chr19	45850096	45850096	-	-	CCTCCTTAGAATCCCACAGTCCCCAAGAT_het	-	CCTCCTTAGAATCCCACAGTCCCCAAGAT_het
frameshift insertion	KLHL35	chr11	75141630	75141630	-	CGCACGGACCCGCGCACGGCGCC_het	-	-	-

frameshift insertion	KLRG2	chr7	139164493	139164493	-	-	-	G_het	-
frameshift deletion	KMT2B	chr19	36223002	36223002	G	G	-_het	G	G
frameshift deletion	KMT2C	chr7	151855000	151855001	AG	AG	-_het	AG	AG
stopgain	KMT2C	chr7	151945071	151945071	-	T_het	T_het	T_het	-
frameshift insertion	KMT2D	chr12	49432287	49432287	-	-	G_het	-	-
frameshift insertion	KRI1	chr19	10676487	10676487	-	-	C_hom	-	-
frameshift deletion	KRIT1	chr7	91843945	91843945	T	T	-_het	T	T
frameshift deletion	KRT20	chr17	39034613	39034616	TCTT	TCTT	-_het	TCTT	TCTT
frameshift insertion	KRTAP10-1	chr21	45959556	45959556	-	TG_hom	TG_hom	-	TG_hom
frameshift deletion	KRTAP10-1	chr21	45959558	45959559	TC	-_hom	-_hom	TC	-_hom
frameshift insertion	KRTAP4-3	chr17	39324348	39324348	-	-	AGCAGCT_het	-	-
frameshift insertion	KRTAP4-3	chr17	39324350	39324350	-	-	AGATA_het	-	-
frameshift deletion	KRTAP5-4	chr11	1643225	1643226	GG	GG	GG	-_hom	GG
frameshift deletion	KRTAP5-4	chr11	1643228	1643267	GCCACAGCCCCCACAGCCAGAGCCACAGCCCCCACAGCCG	GCCACAGCCCCCACAGCCAGAGCCACAGCCCCCACAGCCG	GCCACAGCCCCCACAGCCAGAGCCACAGCCCCCACAGCCG	-_het	GCCACAGCCCCCACAGCCAGAGCCACAGCCCCCACAGCCG
frameshift insertion	LAPTM4B	chr8	98788165	98788165	-	-	GGCGGGCTCCAGGCGA_het	GGCGGGCTCCAGGCGA_het	-
frameshift	LFNG	chr7	2552881	2552881	-	GATG_het	-	GATG_hom	-

insertion									
frameshift deletion	LFNG	chr7	2552882	2552885	GATG	GATG	-_het	GATG	-_hom
frameshift insertion	LGR4	chr11	27389970	27389970	-	-	A_het	-	-
frameshift deletion	LINC00273	chr16	33961471	33961472	CG	-_het	CG	CG	CG
frameshift insertion	LINC00273	chr16	33961475	33961475	-	CA_het	-	-	-
frameshift deletion	LINC00473	chr6	166401104	166401104	A	A	-_het	A	A
frameshift deletion	LINC00552	chr13	114453592	114453605	TGTGTGTGCATGCC	-_hom	-_hom	TGTGTGTGCATGCC	TGTGTGTGCATGCC
frameshift deletion	LINC00615	chr12	91340253	91340254	TT	TT	-_het	TT	TT
frameshift insertion	LINC00636	chr3	107647001	107647001	-	-	A_het	-	-
frameshift deletion	LINC00636	chr3	107647002	107647002	A	-_het	A	A	-_het
frameshift insertion	LINC00636	chr3	107647010	107647010	-	-	C_het	-	-
frameshift deletion	LINC00907	chr18	40096276	40096280	TTTTT	TTTTT	-_hom	TTTTT	TTTTT
frameshift insertion	LINC00937	chr12	8543153	8543153	-	G_hom	-	-	-
frameshift deletion	LIPE	chr19	42911468	42911468	G	G	-_het	G	G
frameshift deletion	LMAN1	chr18	57013194	57013194	T	T	-_het	T	T
frameshift insertion	LMTK3	chr19	49002845	49002845	-	-	C_het	-	-
frameshift	LOC100129961	chr2	135625119	135625120	CT	CT	-_het	CT	CT

deletion									
frameshift deletion	LOC100272216	chr5	68928682	68928682	C	C	C	C	-_het
frameshift deletion	LOC100287036	chr16	89390982	89390982	G	G	-_het	G	G
frameshift deletion	LOC286190	chr8	71573034	71573034	T	T	-_het	T	T
frameshift insertion	LOC401010	chr2	132200620	132200620	-	-	-	G_het	-
frameshift deletion	LOC643770	chr12	98880902	98880903	AA	AA	-_het	AA	AA
frameshift deletion	LOC644189	chr19	36912179	36912185	AAAAAAA	AAAAAAA	-_het	AAAAAAA	AAAAAAA
frameshift deletion	LPHN3	chr4	62903505	62903506	AG	AG	-_het	AG	AG
frameshift deletion	LRIF1	chr1	111495032	111495032	T	T	-_het	T	T
frameshift deletion	LRP1	chr12	57560723	57560723	C	C	-_het	C	C
frameshift insertion	LRP10	chr14	23346503	23346503	-	-	C_het	-	-
frameshift deletion	LRRIQ1	chr12	85638646	85638647	AA	AA	AA	AA	-_het
frameshift deletion	LRRIQ3	chr1	74575213	74575213	T	T	-_het	T	T
frameshift deletion	LSR	chr19	35757845	35757845	C	C	-_het	C	C
frameshift insertion	LTBP3	chr11	65307031	65307031	-	-	C_het	-	-
frameshift insertion	LYPD6B	chr2	150061915	150061915	-	-	A_het	-	-
frameshift deletion	MAMDC4	chr9	139752004	139752004	C	C	-_het	C	C

frameshift deletion	MAML3	chr4	140811084	140811084	C	-_hom	-_hom	C	-_het
frameshift deletion	MAML3	chr4	140811086	140811096	GCTGCTGCTGC	-_hom	-_hom	GCTGCTGCTGC	-_het
frameshift insertion	MAN1B1	chr9	139996503	139996503	-	CATT_het	-	-	-
frameshift deletion	MAN2A1	chr5	109125195	109125195	A	A	-_het	A	A
frameshift insertion	MANEA	chr6	96034869	96034869	-	-	-	-	TATA_het
frameshift deletion	MAPKBP1	chr15	42111746	42111746	G	-_hom	G	-_het	G
frameshift deletion	MARCKS	chr6	114181210	114181210	A	A	-_het	A	A
frameshift deletion	MARS	chr12	57883053	57883053	T	T	-_het	T	T
frameshift deletion	MARVELD2	chr5	68736316	68736316	A	A	-_het	A	A
frameshift insertion	MAST4	chr5	66462580	66462580	-	-	-	-	G_het
frameshift deletion	MBD4	chr3	129155548	129155548	T	T	-_hom	T	T
frameshift deletion	MBD6	chr12	57921401	57921401	C	C	-_het	C	C
frameshift deletion	MBD6	chr12	57921732	57921732	G	G	-_het	G	G
frameshift deletion	MCF2	chrX	138708412	138708412	T	T	-_hom	T	T
frameshift deletion	MCHR2	chr6	100382358	100382358	A	A	-_het	A	A
frameshift deletion	MCOLN1	chr19	7592610	7592610	C	C	-_het	C	C
frameshift	MCPH1	chr8	6289099	6289100	AA	AA	-_het	AA	AA

deletion									
frameshift deletion	MDN1	chr6	90432675	90432675	T	T	-_het	T	T
frameshift deletion	MEPCE	chr7	100028202	100028202	G	G	-_het	G	G
frameshift insertion	MESP1	chr15	90294304	90294304	-	-	-	G_het	-
frameshift insertion	MESP1	chr15	90294306	90294306	-	-	-	ACGGGGCTCGG _het	-
frameshift deletion	MET	chr7	116381122	116381126	TTTTT	TTTTT	-_het	TTTTT	TTTTT
frameshift deletion	MFI2	chr3	196736547	196736547	G	G	-_het	G	G
frameshift deletion	MFSD7	chr4	677499	677499	C	C	-_het	C	C
frameshift deletion	MGC2752	chr19	59093604	59093607	AAAA	AAAA	-_hom	AAAA	AAAA
frameshift deletion	MICALL1	chr22	38323583	38323583	C	C	-_het	C	C
frameshift deletion	MICU2	chr13	22077129	22077129	A	A	-_het	A	A
frameshift deletion	MIS18BP1	chr14	45716019	45716020	TT	TT	-_het	TT	TT
frameshift deletion	MKI67	chr10	129902204	129902205	TG	TG	-_het	TG	TG
frameshift deletion	MLH3	chr14	75514604	75514604	T	T	-_het	T	T
frameshift deletion	MMP1	chr11	102667447	102667447	A	A	-_het	A	A
frameshift deletion	MMP11	chr22	24122806	24122806	G	G	-_het	G	G
frameshift	MST1L	chr1	17085171	17085171	-	-	-	G_het	-

insertion									
frameshift deletion	MST1L	chr1	17086000	17086003	CCCG	-_het	-_het	-_het	-_het
frameshift insertion	MST1L	chr1	17086085	17086085	-	-	C_hom	-	-
frameshift deletion	MST1L	chr1	17087542	17087546	GTGCT	-_het	-_het	-_het	-_het
frameshift deletion	MTR	chr1	237024423	237024428	AGGGCA	AGGGCA	AGGGCA	AGGGCA	-_het
frameshift insertion	MUC16	chr19	8999497	8999497	-	ACCA_het	ACCA_het	-	-
frameshift deletion	MUC16	chr19	8999498	8999501	GCTT	GCTT	GCTT	-_het	-_het
frameshift deletion	MUC16	chr19	8999499	8999502	CTTT	-_het	-_het	-_het	CTTT
frameshift insertion	MUC16	chr19	8999502	8999502	-	-	-	CCGA_het	CCGA_het
frameshift deletion	MUC17	chr7	100680294	100680294	G	G	G	-_het	G
frameshift insertion	MUC17	chr7	100680296	100680296	-	-	-	C_het	-
frameshift deletion	MUC19	chr12	40805840	40805840	T	T	-_het	T	T
frameshift deletion	MUC4	chr3	195505788	195505791	GTGA	GTGA	GTGA	GTGA	-_het
frameshift deletion	MUC4	chr3	195505793	195505836	CTGTGGATGCTGAGGAAGTGTCGGTGACAGGAAGAGGGGTGGCG	CTGTGGATGCTGAGGAAGTGTCGGTGACAGGAAGAGGGGTGGCG	CTGTGGATGCTGAGGAAGTGTCGGTGACAGGAAGAGGGGTGGCG	CTGTGGATGCTGAGGAAGTGTCGGTGACAGGAAGAGGGGTGGCG	-_het
frameshift deletion	MUC4	chr3	195506651	195506652	CA	CA	CA	-_hom	-_hom

frameshift deletion	MUC4	chr3	195507035	195507036	CC	CC	CC	-_het	CC
frameshift insertion	MUC4	chr3	195507038	195507038	-	-	-	TC_het	-
frameshift deletion	MUC4	chr3	195507364	195507373	GGGGTGGCCT	GGGGTGGCCT	GGGGTGGCCT	GGGGTGGCCT	-_het
frameshift deletion	MUC4	chr3	195507376	195507461	CCTGTGGATGCTGAGGAAGTGTCCGTGACAGGAAGACGGGTGGTGTCACCTGTGGATGCTGAGGAAGTGTCGGTGACAGGAAGAGG	CCTGTGGATGCTGAGGAAGTGTCCGTGACAGGAAGACGGGTGGTGTCACCTGTGGATGCTGAGGAAGTGTCGGTGACAGGAAGAGG	CCTGTGGATGCTGAGGAAGTGTCCGTGACAGGAAGACGGGTGGTGTCACCTGTGGATGCTGAGGAAGTGTCGGTGACAGGAAGAGG	CCTGTGGATGCTGAGGAAGTGTCCGTGACAGGAAGACGGGTGGTGTCACCTGTGGATGCTGAGGAAGTGTCGGTGACAGGAAGAGG	-_het
frameshift deletion	MUC4	chr3	195511446	195511446	C	-_het	C	C	C
frameshift deletion	MUC4	chr3	195514498	195514499	AG	AG	AG	-_hom	AG
frameshift insertion	MUC4	chr3	195514501	195514501	-	-	-	G_hom	-
frameshift insertion	MUC4	chr3	195514502	195514502	-	-	-	C_hom	-
frameshift insertion	MUC6	chr11	1017035	1017035	-	-	-	AT_het	-
frameshift deletion	MUC6	chr11	1017041	1017042	GT	GT	GT	-_het	GT
frameshift insertion	MUC6	chr11	1018215	1018215	-	CA_het	-	-	CA_het
frameshift deletion	MUC6	chr11	1018223	1018224	AT	-_het	AT	AT	-_het
frameshift insertion	MUM1	chr19	1360569	1360569	-	-	G_het	-	-

frameshift deletion	MYEOV	chr11	69063724	69063724	G	G	G	G	-_het
frameshift deletion	MYPOP	chr19	46393972	46393972	G	G	-_het	G	G
frameshift deletion	NANOS1	chr10	120789360	120789360	C	C	-_hom	C	C
frameshift insertion	NARG2	chr15	60741266	60741266	-	-	T_het	-	-
frameshift deletion	NARG2	chr15	60745782	60745782	A	A	-_hom	A	A
frameshift deletion	NBEA	chr13	35733663	35733663	A	A	-_het	A	A
frameshift deletion	NBEAL1	chr2	203922058	203922058	A	A	-_het	A	A
frameshift deletion	NCOR1	chr17	15983844	15983845	AT	-_het	AT	AT	AT
frameshift deletion	NDUFC2-KCTD14	chr11	77784147	77784147	A	A	-_het	A	A
frameshift deletion	NFATC2	chr20	50158999	50158999	C	C	-_het	C	C
frameshift deletion	NFKBIA	chr14	35872461	35872461	G	G	-_het	G	G
frameshift deletion	NGDN	chr14	23946763	23946763	A	A	-_het	A	A
frameshift deletion	NID2	chr14	52481919	52481919	G	G	-_het	G	G
frameshift deletion	NIPBL	chr5	37051862	37051862	T	T	-_hom	T	T
frameshift deletion	NKG7	chr19	51875459	51875459	G	G	-_het	G	G
frameshift deletion	NLRC5	chr16	57059488	57059488	C	C	-_het	C	C
frameshift	NMBR	chr6	142399695	142399695	T	T	-_het	T	T

deletion									
frameshift insertion	NOM1	chr7	156752697	156752697	-	-	A_het	-	-
frameshift insertion	NOP16	chr5	175811094	175811094	-	GT_hom	GT_hom	AT_hom	GT_hom
frameshift deletion	NRXN2	chr11	64410246	64410246	C	C	-_het	C	C
frameshift insertion	NSRP1	chr17	28505166	28505166	-	-	A_het	-	-
frameshift insertion	NTN5	chr19	49173723	49173723	-	-	G_het	-	-
frameshift insertion	NUDT18	chr8	21966891	21966891	-	-	CCGCTCCCAGTCCCTGCGGAGA_het	CCGCTCCCAGTCCCTGCGGAGA_hom	-
frameshift deletion	NUP214	chr9	134007993	134007993	A	A	-_het	A	A
frameshift deletion	ODF1	chr8	103573024	103573031	GCCCCTGC	GCCCCTGC	GCCCCTGC	-_het	GCCCCTGC
frameshift deletion	ODF1	chr8	103573033	103573042	ACCCGTGCAG	ACCCGTGCAG	ACCCGTGCAG	-_het	ACCCGTGCAG
frameshift deletion	OLIG2	chr21	34399986	34399986	G	G	-_het	G	G
frameshift insertion	OR11G2	chr14	20666175	20666175	-	A_hom	A_het	A_hom	A_hom
frameshift insertion	OR2B6	chr6	27925197	27925197	-	-	T_het	-	-
frameshift deletion	OR2L2	chr1	248202094	248202094	T	T	-_het	T	T
stopgain	OR2T35	chr1	248801602	248801602	-	-	-	CA_het	-
frameshift deletion	OR2T35	chr1	248801945	248801951	CAGCACG	CAGCACG	-_hom	-_hom	-_het
frameshift	OR4A16	chr11	55111232	55111233	TT	-_het	TT	TT	TT

deletion									
frameshift insertion	OR4A16	chr11	55111234	55111234	-	CA_het	-	-	-
frameshift deletion	OR4C3	chr11	48347449	48347449	A	A	-_het	A	A
frameshift insertion	OR4C45	chr11	48367050	48367050	-	AG_het	AG_het	AG_het	AG_het
frameshift insertion	OR4M2	chr15	22369023	22369023	-	-	G_het	-	-
frameshift deletion	OR52N5	chr11	5799652	5799652	A	A	-_het	A	A
frameshift deletion	OR6C76	chr12	55820959	55820960	AA	AA	-_het	AA	AA
frameshift deletion	OR7C2	chr19	15052829	15052829	T	T	-_het	T	T
frameshift insertion	OR8U8	chr11	56143255	56143255	-	GA_het	GA_het	GA_het	GA_het
frameshift deletion	OR8U8	chr11	56143260	56143261	GT	-_het	-_het	-_het	-_het
frameshift insertion	OR8U8	chr11	56143424	56143424	-	-	CGGC_het	CGGC_het	-
frameshift deletion	OR8U8	chr11	56143426	56143429	ATCA	ATCA	-_het	-_het	ATCA
frameshift insertion	OR8U8	chr11	56143782	56143782	-	GA_het	GA_het	GA_het	-
frameshift deletion	OR8U8	chr11	56143785	56143786	AT	-_het	-_het	-_het	AT
frameshift deletion	ORC5	chr7	103805692	103805692	T	T	-_het	T	T
frameshift insertion	OSMR	chr5	38923328	38923328	-	-	A_het	-	-
frameshift deletion	OTOG	chr11	17656703	17656703	G	G	-_hom	G	G

frameshift deletion	OVCA2	chr17	1945491	1945491	C	C	-_het	C	C
frameshift deletion	OXA1L	chr14	23236555	23236558	CTTC	CTTC	-_het	CTTC	CTTC
frameshift deletion	OXER1	chr2	42990728	42990728	G	G	-_het	G	G
frameshift deletion	PABPC1	chr8	101721933	101721933	T	T	T	-_het	T
frameshift insertion	PABPC3	chr13	25670801	25670801	-	G_het	-	G_het	-
frameshift deletion	PABPC3	chr13	25671273	25671273	G	-_het	G	-_het	-_het
frameshift deletion	PABPC3	chr13	25671311	25671315	TATGA	-_het	TATGA	-_het	-_het
frameshift deletion	PABPC3	chr13	25671333	25671333	A	-_het	A	-_het	-_het
frameshift insertion	PADI3	chr1	17601264	17601264	-	-	G_het	-	-
frameshift insertion	PARP2	chr14	20825321	20825321	-	-	-	TTCAC_het	-
frameshift insertion	PCDHB17	chr5	140537010	140537010	-	-	-	-	A_het
frameshift insertion	PCDHB8	chr5	140558313	140558313	-	CC_hom	CC_het	-	CC_het
frameshift deletion	PCDHB8	chr5	140558316	140558317	TC	-_hom	-_het	TC	-_het
frameshift deletion	PCDHGA10	chr5	140795209	140795209	A	A	-_het	A	A
frameshift deletion	PCDHGA12	chr5	140812776	140812776	T	T	-_het	T	T
frameshift deletion	PCLO	chr7	82582944	82582944	T	T	-_het	T	T
frameshift	PCNXL2	chr1	233337948	233337948	T	T	-_hom	T	T

deletion									
frameshift deletion	PCSK5	chr9	78790149	78790153	AATGA	-_het	AATGA	AATGA	AATGA
frameshift insertion	PCSK5	chr9	78790187	78790187	-	-	-	GAATC_hom	-
frameshift deletion	PCSK5	chr9	78790208	78790212	GAATA	GAATA	-_het	GAATA	GAATA
frameshift deletion	PDE11A	chr2	178936605	178936605	G	G	-_het	G	G
frameshift deletion	PDE3A	chr12	20522723	20522723	G	G	-_het	G	G
frameshift deletion	PDIA2	chr16	336701	336702	CT	CT	-_het	CT	CT
frameshift deletion	PDS5B	chr13	33344888	33344889	AA	AA	-_het	AA	AA
frameshift deletion	PDXDC1	chr16	15100306	15100306	A	A	-_het	A	A
frameshift deletion	PFKFB3	chr10	6268227	6268227	A	A	-_het	A	A
frameshift deletion	PHACTR1	chr6	13206056	13206056	C	C	-_het	C	C
frameshift deletion	PHACTR4	chr1	28785730	28785730	A	A	-_hom	A	A
frameshift deletion	PHC3	chr3	169889138	169889138	A	A	-_het	A	A
frameshift insertion	PHF15	chr5	133861425	133861425	-	-	G_het	-	-
frameshift deletion	PHF2	chr9	96422612	96422612	A	A	-_het	A	A
stopgain	PHKA1	chrX	71802286	71802286	T	T	-_hom	T	T
frameshift deletion	PI4KAP2	chr22	21832293	21832306	GCAGTGGCCACCAC	GCAGTGGCCACCAC	GCAGTGGCCACCAC	GCAGTGGCCACCAC	-_het

frameshift insertion	PIDD	chr11	801631	801631	-	G_hom	G_het	-	G_het
frameshift deletion	PIGM	chr1	160000813	160000813	A	A	-_het	A	A
frameshift deletion	PKD1	chr16	2143888	2143888	G	G	-_het	G	G
frameshift insertion	PKD1L3	chr16	71981414	71981414	-	TTTG_hom	TTTG_het	-	TTTG_hom
frameshift deletion	PKHD1	chr6	51930802	51930802	A	A	-_het	A	A
frameshift deletion	PLCG2	chr16	81954997	81954998	TT	TT	-_het	TT	TT
frameshift deletion	PLXNB3	chrX	153042691	153042691	G	G	-_hom	G	G
frameshift deletion	PNPLA5	chr22	44287050	44287050	G	G	-_het	G	G
frameshift insertion	PNPLA6	chr19	7620540	7620540	-	-	C_het	-	-
frameshift deletion	POTED	chr21	15000762	15000765	AGCC	AGCC	AGCC	-_het	AGCC
frameshift insertion	POU5F1B	chr8	128428147	128428147	-	-	C_het	-	-
frameshift deletion	PPARGC1B	chr5	149206386	149206386	C	C	-_het	C	C
frameshift insertion	PPFIBP1	chr12	27829483	27829483	-	-	T_het	-	-
frameshift deletion	PPIG	chr2	170460581	170460581	T	T	-_het	T	T
frameshift insertion	PPL	chr16	4944499	4944499	-	-	G_het	-	-
frameshift deletion	PPP1R12A	chr12	80201042	80201042	T	T	-_het	T	T
frameshift	PPP1R15A	chr19	49377757	49377757	C	C	-_het	C	C

deletion									
frameshift deletion	PPP2R5D	chr6	42978677	42978677	C	C	-_het	C	C
frameshift deletion	PPP6R2	chr22	50882377	50882377	C	C	-_het	C	C
frameshift deletion	PRAP1	chr10	135160928	135160944	AGGTAATGCCACCATCC	AGGTAATGCCACCATCC	AGGTAATGCCACCATCC	-_het	AGGTAATGCCACCATCC
frameshift deletion	PRB2	chr12	11546611	11546611	G	-_het	G	G	G
frameshift deletion	PRB3	chr12	11420392	11420517	GGACGAGGTGGGGGACCTTGGGACTGGTTTCCTCCTTGTGGGGGTGGTCCTTCTGGCTTTCCTGGACGAGGTGGGGGACCTTGAGGTTTGTTGCCTCCTTGTGGGGGTGGTCCTTCTGGCTTTCCC	-_het	-_het	GGACGAGGTGGGGGACCTTGGGACTGGTTTCCTCCTTGTGGGGGTGGTCCTTCTGGCTTTCCTGGACGAGGTGGGGGACCTTGAGGTTTGTTGCCTCCTTGTGGGGGTGGTCCTTCTGGCTTTCCC	GGACGAGGTGGGGGACCTTGGGACTGGTTTCCTCCTTGTGGGGGTGGTCCTTCTGGCTTTCCTGGACGAGGTGGGGGACCTTGAGGTTTGTTGCCTCCTTGTGGGGGTGGTCCTTCTGGCTTTCCC
frameshift insertion	PRIM2	chr6	57398186	57398186	-	-	-	-	A_het
stopgain	PRKCDBP	chr11	6340510	6340510	-	-	-	TCCGGCTGGGCTTCAGCGC_hom	-
frameshift deletion	PRKDC	chr8	48866910	48866910	T	T	-_hom	T	T
frameshift deletion	PROC	chr2	128186343	128186343	G	G	-_het	G	G
frameshift deletion	PRR13	chr12	53837317	53837317	C	C	-_het	C	C

frameshift deletion	PRR21	chr2	240982230	240982257	TGGGTGAAGAGCCGTGGATGAAGGGCCA	TGGGTGAAGAGCCGTGGATGAAGGGCCA	TGGGTGAAGAGCCGTGGATGAAGGGCCA	-_hom	TGGGTGAAGAGCCGTGGATGAAGGGCCA
frameshift insertion	PRRT2	chr16	29825015	29825015	-	-	-	-	C_hom
frameshift deletion	PRRT3	chr3	9990883	9990883	G	G	-_het	G	G
frameshift deletion	PRSS1	chr7	142458842	142458842	T	-_het	-_het	T	-_het
frameshift deletion	PRSS3	chr9	33794798	33794799	GA	GA	GA	-_het	GA
frameshift insertion	PRSS3	chr9	33797928	33797928	-	CC_het	CC_het	CC_het	CC_het
frameshift deletion	PRSS3	chr9	33797931	33797932	AC	-_het	-_het	-_het	-_het
frameshift deletion	PSD	chr10	104175781	104175781	G	G	-_het	G	G
frameshift deletion	PSMD1	chr2	232029972	232029972	A	A	-_het	A	A
frameshift deletion	PSME4	chr2	54093345	54093345	T	T	-_het	T	T
frameshift deletion	PSME4	chr2	54115914	54115914	A	A	-_het	A	A
frameshift deletion	PTCHD3	chr10	27702951	27702951	G	G	-_het	G	G
frameshift deletion	PTPN23	chr3	47454399	47454399	G	G	-_hom	G	G
frameshift deletion	PTPN4	chr2	120639371	120639371	T	T	-_het	T	T
frameshift deletion	PYCARD	chr16	31213996	31213997	CG	CG	-_het	CG	CG
frameshift	QSER1	chr11	32955078	32955078	A	A	-_het	A	A

deletion									
frameshift deletion	RAB36	chr22	23487615	23487615	G	G	-_het	G	G
frameshift deletion	RAD21-AS1	chr8	117887129	117887130	CT	CT	CT	CT	-_het
frameshift deletion	RAD50	chr5	131931452	131931453	AA	AA	-_het	AA	AA
frameshift deletion	RAI1	chr17	17697099	17697100	GC	GC	-_het	-_hom	-_hom
frameshift deletion	RAI1	chr17	17697102	17697102	G	G	G	-_hom	-_hom
frameshift deletion	RAI1	chr17	17699359	17699359	C	C	-_het	C	C
frameshift deletion	RALGPS1	chr9	129946262	129946262	C	C	-_het	C	C
frameshift insertion	RB1	chr13	48881488	48881488	-	-	AG_het	-	-
frameshift insertion	RB1	chr13	48955464	48955464	-	-	T_het	-	-
frameshift insertion	RBM26	chr13	79918806	79918806	-	-	T_het	-	-
frameshift deletion	RBM27	chr5	145647320	145647320	A	A	-_het	A	A
frameshift deletion	RBM28	chr7	127983764	127983764	C	C	-_het	C	C
frameshift insertion	RBMX	chrX	135960146	135960146	-	-	-	AA_het	-
frameshift deletion	RBMXL1	chr1	89449509	89449509	T	T	-_hom	T	T
frameshift deletion	RECQL	chr12	21644547	21644547	T	T	-_het	T	T
frameshift deletion	RETSAT	chr2	85570875	85570878	AGAT	AGAT	AGAT	-_het	AGAT

frameshift deletion	RFC3	chr13	34398063	34398063	A	A	-_het	A	A
frameshift insertion	RGS12	chr4	3432430	3432430	-	-	C_het	-	-
frameshift deletion	RHBDD2	chr7	75517566	75517566	C	C	-_het	C	C
frameshift deletion	RIMKLB	chr12	8930200	8930200	A	A	-_het	A	A
frameshift insertion	RNF103	chr2	86831014	86831014	-	-	T_het	-	-
frameshift deletion	RNF139	chr8	125498508	125498509	AT	AT	-_het	AT	AT
frameshift deletion	RNF43	chr17	56435161	56435161	C	C	-_hom	C	C
frameshift deletion	ROBO1	chr3	78710235	78710235	A	A	-_hom	A	A
frameshift deletion	ROCK2	chr2	11355500	11355500	T	T	-_het	T	T
frameshift deletion	ROM1	chr11	62381084	62381084	G	G	-_het	G	G
frameshift deletion	RP1L1	chr8	10467587	10467587	T	T	T	T	-_hom
frameshift deletion	RP1L1	chr8	10467591	10467637	CTCTAACTGCACCCCCTCTTCTTGCAGCCCTTCTTCTGTTTTAGTTT	CTCTAACTGCACCCCCTCTTCTTGCAGCCCTTCTTCTGTTTTAGTTT	CTCTAACTGCACCCCCTCTTCTTGCAGCCCTTCTTCTGTTTTAGTTT	CTCTAACTGCACCCCCTCTTCTTGCAGCCCTTCTTCTGTTTTAGTTT	-_hom
frameshift insertion	RPL18	chr19	49120252	49120252	-	A_het	-	A_het	-
frameshift deletion	RPL18	chr19	49120253	49120253	A	A	-_hom	A	A
frameshift insertion	RPL22	chr1	6257784	6257784	-	-	T_het	-	-

frameshift insertion	RPL23AP32	chr2	54756587	54756587	-	-	A_het	-	-
frameshift deletion	RPL2B	chr16	436727	436728	AA	AA	-_hom	AA	-_hom
frameshift deletion	RRP7A	chr22	42912343	42912343	T	T	-_hom	T	T
frameshift deletion	RSPO3	chr6	127516969	127516969	A	A	-_het	A	A
stopgain	RTN4	chr2	55253746	55253746	T	T	-_het	T	T
frameshift insertion	RUNX1-IT1	chr21	36410910	36410910	-	-	-	TG_het	-
frameshift deletion	RUNX1-IT1	chr21	36410911	36410912	TG	TG	TG	TG	-_het
frameshift deletion	RUSC2	chr9	35547399	35547399	C	C	-_het	C	C
frameshift deletion	SALL3	chr18	76755299	76755299	C	C	-_het	C	C
frameshift deletion	SAMD11	chr1	874779	874826	CCTCCCCAGCCACGGTGAGGACCCACCCTGGCATGATCCCCCTCATCA	-_hom	CCTCCCCAGCCACGGTGAGGACCCACCCTGGCATGATCCCCCTCATCA	CCTCCCCAGCCACGGTGAGGACCCACCCTGGCATGATCCCCCTCATCA	CCTCCCCAGCCACGGTGAGGACCCACCCTGGCATGATCCCCCTCATCA
frameshift deletion	SAMD15	chr14	77844152	77844155	AAAG	AAAG	AAAG	-_het	AAAG
frameshift deletion	SARM1	chr17	26699206	26699206	A	-_hom	-_hom	-_hom	-_hom
frameshift insertion	SARM1	chr17	26708298	26708298	-	GC_hom	GC_hom	GC_hom	GC_hom
frameshift deletion	SARM1	chr17	26708303	26708304	GT	-_hom	-_hom	-_hom	-_hom
frameshift deletion	SDF4	chr1	1153102	1153102	T	T	-_het	T	T

frameshift deletion	SDHA	chr5	251704	251704	G	G	G	-_het	G
frameshift deletion	SDHA	chr5	256484	256485	TT	TT	TT	-_het	-_het
frameshift deletion	SEC16B	chr1	177927333	177927333	G	G	-_het	G	G
frameshift deletion	SEC31A	chr4	83785565	83785565	T	T	-_het	T	T
frameshift deletion	SEC63	chr6	108214755	108214755	T	T	-_hom	T	T
frameshift deletion	SETD1B	chr12	122242658	122242659	CC	CC	-_het	CC	CC
frameshift deletion	SETD7	chr4	140432892	140432892	G	G	-_het	G	G
frameshift deletion	SEZ6L	chr22	26702008	26702008	A	A	-_het	A	A
frameshift deletion	SGOL2	chr2	201437004	201437004	T	T	-_het	T	T
frameshift deletion	SHC1	chr1	154938890	154938890	C	C	-_het	C	C
frameshift deletion	SHISA7	chr19	55945040	55945040	C	C	-_het	C	C
frameshift deletion	SIGLEC10	chr19	51919435	51919436	AA	AA	-_hom	AA	AA
frameshift insertion	SIRPA	chr20	1895949	1895949	-	-	-	-	GT_hom
frameshift deletion	SIRPA	chr20	1895951	1895952	CT	CT	CT	CT	-_hom
frameshift deletion	SIRPB1	chr20	1592151	1592152	GA	GA	GA	GA	-_hom
frameshift insertion	SIRPB1	chr20	1592154	1592154	-	-	-	-	AC_hom
frameshift	SKA3	chr13	21732264	21732264	-	CAAAG_het	-	-	-

insertion									
frameshift insertion	SLAIN1	chr13	78272267	78272267	-	GG_hom	GG_hom	-	GG_hom
frameshift insertion	SLC16A1	chr1	113460185	113460185	-	-	A_het	-	-
frameshift insertion	SLC16A4	chr1	110906426	110906426	-	-	A_het	-	-
frameshift deletion	SLC19A2	chr1	169446809	169446809	A	A	-_het	A	A
frameshift insertion	SLC22A2	chr6	160679656	160679656	-	-	T_hom	-	-
frameshift deletion	SLC22A20	chr11	64981489	64981489	A	A	-_het	A	A
frameshift insertion	SLC22A25	chr11	62996774	62996774	-	-	-	A_hom	-
frameshift deletion	SLC22A8	chr11	62763397	62763397	A	A	A	A	-_het
frameshift insertion	SLC25A5	chrX	118603706	118603706	-	-	G_het	G_het	G_het
frameshift deletion	SLC25A5	chrX	118603962	118603962	T	T	-_het	-_het	T
frameshift deletion	SLC27A4	chr9	131112835	131112835	C	C	-_het	C	C
frameshift deletion	SLC35G2	chr3	136573486	136573486	A	A	-_hom	A	A
frameshift deletion	SLC38A10	chr17	79225014	79225074	TTTAAAGGGGAGCAAGGGAGATAAAACGGTAATCACAAGCCATGTGACGATGCCGCAGGTG	TTTAAAGGGGAGCAAGGGAGATAAAACGGTAATCACAAGCCATGTGACGATGCCGCAGGTG	-_het	TTTAAAGGGGAGCAAGGGAGATAAAACGGTAATCACAAGCCATGTGACGATGCCGCAGGTG	TTTAAAGGGGAGCAAGGGAGATAAAACGGTAATCACAAGCCATGTGACGATGCCGCAGGTG
frameshift	SLC6A10P	chr16	32890641	32890641	-	-	-	A_het	-

insertion									
frameshift deletion	SLC6A6	chr3	14485478	14485479	CC	CC	CC	-_het	CC
frameshift deletion	SLC7A13	chr8	87242135	87242135	A	A	-_het	A	A
frameshift deletion	SLC8A2	chr19	47969003	47969003	A	A	-_het	A	A
frameshift deletion	SLC9A8	chr20	48467301	48467301	T	T	-_het	T	T
frameshift deletion	SLC9B1	chr4	103822483	103822484	AC	AC	-_het	-_het	-_het
frameshift insertion	SLC9B1	chr4	103870488	103870488	-	-	A_het	-	-
frameshift insertion	SLC9B2	chr4	103941529	103941529	-	A_het	-	-	-
frameshift deletion	SLFN12	chr17	33749493	33749493	A	A	-_het	A	A
frameshift insertion	SLITRK1	chr13	84455508	84455508	-	-	T_het	-	-
frameshift deletion	SMAD4	chr18	48604788	48604791	ACGA	ACGA	ACGA	-_hom	ACGA
frameshift deletion	SMAP1	chr6	71508370	71508371	AA	AA	-_het	AA	AA
frameshift insertion	SNAPC5	chr15	66782790	66782790	-	-	T_het	-	-
frameshift deletion	SNRNP40	chr1	31732603	31732606	AAAA	AAAA	-_hom	AAAA	AAAA
frameshift deletion	SNRNP40	chr1	31732678	31732678	A	A	-_het	A	A
frameshift deletion	SNX13	chr7	17922565	17922566	CT	-_het	CT	CT	CT
frameshift deletion	SNX27	chr1	151584936	151584936	G	G	-_het	G	G

frameshift insertion	SOGA2	chr18	8786108	8786108	-	-	-	-	CCCC_hom
frameshift insertion	SOGA2	chr18	8786117	8786117	-	-	A_hom	-	-
frameshift insertion	SOGA2	chr18	8793003	8793003	-	-	C_het	-	-
frameshift deletion	SOS2	chr14	50623682	50623685	TATA	TATA	TATA	-_het	TATA
frameshift deletion	SPANXN1	chrX	144337333	144337334	GA	GA	GA	-_hom	GA
frameshift insertion	SPG11	chr15	44912608	44912608	-	-	A_het	-	-
frameshift deletion	SPINK5	chr5	147499875	147499875	A	A	-_het	A	A
frameshift deletion	SQLE	chr8	126011893	126011893	C	C	-_het	C	C
frameshift insertion	SRMS	chr20	62175647	62175647	-	-	G_het	-	-
frameshift insertion	SRRT	chr7	100479331	100479331	-	-	G_het	-	-
frameshift deletion	SSPO	chr7	149518533	149518533	C	C	-_het	C	C
stopgain	SSTR1	chr14	38679763	38679763	-	-	GCTCTGAGCCC GGGCCACGCAG GG_hom	-	-
frameshift insertion	SSTR5-AS1	chr16	1115493	1115493	-	-	T_het	-	-
frameshift deletion	SSTR5-AS1	chr16	1115843	1115846	GGCA	-_het	GGCA	GGCA	GGCA
frameshift insertion	SSTR5-AS1	chr16	1116220	1116220	-	G_hom	G_hom	G_hom	G_hom
frameshift	ST18	chr8	53074085	53074085	-	-	T_het	-	-

insertion									
frameshift deletion	STAB2	chr12	103981277	103981277	T	T	-_het	T	T
frameshift deletion	STAC3	chr12	57637343	57637343	T	T	T	T	-_het
frameshift deletion	STARD9	chr15	42977254	42977254	A	A	-_het	A	A
frameshift deletion	STRC	chr15	43893734	43893734	G	G	-_het	G	G
frameshift deletion	STXBP2	chr19	7704893	7704894	TG	TG	TG	TG	-_het
frameshift deletion	STXBP2	chr19	7705384	7705397	ATCTGTGTGTGTGT	ATCTGTGTGTGTGT	-_hom	ATCTGTGTGTGTGT	ATCTGTGTGTGTGT
frameshift insertion	SULT1C3	chr2	108863684	108863684	-	-	A_het	-	-
stopgain	SYCP2	chr20	58444990	58444990	T	T	-_het	T	T
stopgain	SYCP2	chr20	58467047	58467047	T	T	-_het	T	T
frameshift deletion	SYNE1	chr6	152688162	152688177	AAACATCCGGGGTCAG	AAACATCCGGGGTCAG	AAACATCCGGGGTCAG	AAACATCCGGGGTCAG	-_het
frameshift deletion	T	chr6	166576104	166576104	C	C	-_het	C	C
frameshift deletion	TAF1B	chr2	9989571	9989571	A	A	-_het	A	A
frameshift insertion	TAS2R43	chr12	11244067	11244067	-	TT_het	TT_hom	-	-
frameshift deletion	TAS2R43	chr12	11244071	11244072	CC	-_het	-_hom	CC	CC
frameshift deletion	TBC1D1	chr4	38051454	38051454	G	G	-_het	G	G
frameshift deletion	TBC1D23	chr3	100039736	100039736	A	A	-_hom	A	A
frameshift	TBCK	chr4	107156505	107156505	T	T	-_het	T	T

deletion									
frameshift deletion	TBXAS1	chr7	139662016	139662016	T	T	-_het	T	T
frameshift insertion	TCEB3CL2	chr18	44549025	44549025	-	-	GC_het	-	-
frameshift deletion	TCF7L1	chr2	85360831	85360831	C	C	-_het	C	C
frameshift deletion	TCF7L1	chr2	85360833	85360834	GC	GC	-_het	GC	GC
frameshift deletion	TCHP	chr12	110344435	110344435	A	A	-_het	A	A
frameshift deletion	TCP11	chr6	35108990	35108990	C	-_het	C	C	C
stopgain	TCRAVN1	chr14	22580859	22580859	G	G	G	-_het	G
frameshift insertion	TCRBV12S2	chr7	142231793	142231793	-	T_het	-	T_het	T_het
frameshift deletion	TCRBV12S2	chr7	142231797	142231798	CA	-_het	CA	-_het	-_het
frameshift insertion	TCRBV12S2	chr7	142231800	142231800	-	A_het	-	A_het	A_het
frameshift insertion	TCRBV12S2	chr7	142231805	142231805	-	TG_het	TG_het	TG_het	TG_het
frameshift deletion	TCRBV12S2	chr7	142231809	142231810	CC	-_het	-_het	-_het	-_het
frameshift deletion	TCRBV13S1	chr7	142180585	142180586	CC	-_het	CC	CC	CC
frameshift deletion	TCRBV13S1	chr7	142180592	142180593	GG	-_het	GG	GG	GG
frameshift insertion	TCRBV13S1	chr7	142180593	142180593	-	TTTT_het	-	-	-
frameshift insertion	TCRBV20S1	chr7	142032391	142032391	-	AC_het	-	-	-

frameshift deletion	TCRBV21S1	chr7	142223949	142223950	AC	AC	-_het	AC	AC
frameshift insertion	TCRBV21S1	chr7	142223953	142223953	-	-	TC_het	-	-
frameshift insertion	TCRBV21S1	chr7	142224020	142224020	-	-	-	-	TT_het
frameshift deletion	TCRBV21S1	chr7	142224022	142224023	CA	CA	CA	CA	-_het
frameshift deletion	TCRBV5S6A3N2T	chr7	142168509	142168509	C	-_het	-_het	C	-_het
frameshift insertion	TCRBV5S6A3N2T	chr7	142168515	142168515	-	C_het	C_het	-	C_het
frameshift deletion	TCRBV6S1A1N1	chr7	142247530	142247531	GC	-_het	GC	-_het	-_het
frameshift insertion	TCRBV6S1A1N1	chr7	142247535	142247535	-	AA_het	AA_het	AA_het	AA_het
frameshift deletion	TCRBV6S1A1N1	chr7	142247539	142247540	TG	-_het	TG	TG	-_het
frameshift insertion	TCRBV6S1A1N1	chr7	142247541	142247541	-	AT_het	-	-	AT_het
frameshift deletion	TCRBV6S3A1N1T	chr7	142139335	142139335	G	-_het	G	-_het	-_het
frameshift insertion	TCRBV6S3A1N1T	chr7	142139337	142139337	-	CA_het	-	CA_het	CA_het
frameshift deletion	TCRBV6S3A1N1T	chr7	142139340	142139340	A	-_het	A	-_het	-_het
frameshift deletion	TCRBV6S6A2T	chr7	142119877	142119877	G	-_het	G	-_het	G
frameshift insertion	TCRBV6S6A2T	chr7	142119879	142119879	-	CA_het	-	CA_het	-
frameshift deletion	TCRBV6S6A2T	chr7	142119882	142119882	A	-_het	A	-_het	A
frameshift	TCRBV9S1A1T	chr7	142008635	142008636	CG	-_het	CG	CG	CG

deletion									
frameshift insertion	TCRBV9S1A1T	chr7	142008639	142008639	-	GG_het	GG_het	-	-
frameshift deletion	TENC1	chr12	53453019	53453019	C	C	-_het	C	C
frameshift insertion	TEP1	chr14	20846337	20846337	-	-	C_het	-	-
frameshift deletion	TEX13A	chrX	104464282	104464282	C	-_hom	-_hom	-_hom	-_hom
frameshift deletion	TFAM	chr10	60148570	60148570	A	A	-_het	A	A
frameshift deletion	TGFB2	chr1	218578727	218578728	CT	CT	-_het	CT	CT
frameshift deletion	TGFBR2	chr3	30691872	30691872	A	A	-_hom	A	A
frameshift deletion	TIE1	chr1	43779725	43779726	TG	-_het	-_het	TG	TG
frameshift deletion	TLCD2	chr17	1613129	1613130	GC	GC	-_hom	GC	GC
stopgain	TLR2	chr4	154624834	154624834	G	G	G	G	-_het
frameshift deletion	TLR4	chr9	120470861	120470861	T	-_het	T	T	T
frameshift deletion	TMCC3	chr12	94976258	94976258	C	C	-_het	C	C
frameshift deletion	TMEM131	chr2	98378554	98378554	G	G	-_het	G	G
frameshift deletion	TMEM185B	chr2	120979531	120979531	G	G	-_het	G	G
frameshift insertion	TMEM2	chr9	74324369	74324369	-	-	A_het	-	-

frameshift deletion	TMEM255B	chr13	114503876	114503957	CCTGCGGGAGGTGAGGGGCACCGGGGACCCCCATATCTACACCTGCGGGAGGTGAGGGGCGCTGGGGACCCCCGTATCTACA	-_hom	-_hom	CCTGCGGGAGGTGAGGGGCACCGGGGACCCCCATATCTACACCTGCGGGAGGTGAGGGGCGCTGGGGACCCCCGTATCTACA	CCTGCGGGAGGTGAGGGGCACCGGGGACCCCCATATCTACACCTGCGGGAGGTGAGGGGCGCTGGGGACCCCCGTATCTACA
frameshift insertion	TMEM260	chr14	57052611	57052611	-	-	T_het	-	-
frameshift deletion	TMEM65	chr8	125384238	125384259	AGCCGCCTGGGGCCGCCCGGCA	AGCCGCCTGGGGCCGCCCGGCA	AGCCGCCTGGGGCCGCCCGGCA	AGCCGCCTGGGGCCGCCCGGCA	-_hom
frameshift deletion	TMEM97	chr17	26653807	26653807	A	A	-_hom	A	A
frameshift deletion	TMPO	chr12	98921672	98921672	A	A	-_het	A	A
frameshift deletion	TMPRSS5	chr11	113567641	113567641	G	G	G	G	-_het
frameshift deletion	TNFAIP6	chr2	152236046	152236049	AAAA	AAAA	-_het	AAAA	AAAA
frameshift deletion	TNK2	chr3	195594633	195594633	G	G	-_het	G	G
frameshift insertion	TNK2	chr3	195595228	195595228	-	-	G_hom	-	-
frameshift deletion	TNMD	chrX	99854070	99854070	A	A	-_hom	A	A
frameshift deletion	TNRC6B	chr22	40669453	40669453	G	G	-_het	G	G
frameshift deletion	TOM1L2	chr17	17772623	17772623	A	A	A	A	-_het
frameshift deletion	TOR2A	chr9	130495628	130495628	C	C	-_het	C	C

frameshift deletion	TP53	chr17	7579394	7579394	G	G	G	G	-_hom
frameshift insertion	TP73	chr1	3644315	3644315	-	-	G_het	-	-
frameshift deletion	TPM3	chr1	154144892	154144892	T	T	-_het	T	T
frameshift deletion	TPMT	chr6	18134009	18134010	AA	AA	-_het	AA	AA
frameshift deletion	TRAM1L1	chr4	118005517	118005517	T	T	-_het	T	T
frameshift deletion	TRDN	chr6	123786033	123786033	A	A	-_het	A	A
frameshift deletion	TRERF1	chr6	42237001	42237001	C	C	-_het	C	C
frameshift deletion	TRIM22	chr11	5717765	5717765	A	A	-_het	A	A
frameshift deletion	TRIM51	chr11	55653610	55653610	A	A	-_het	A	A
frameshift insertion	TRIM77	chr11	89444610	89444610	-	-	A_het	-	-
frameshift deletion	TSPYL2	chrX	53113896	53113897	GG	GG	-_hom	GG	GG
frameshift insertion	TSPYL2	chrX	53115502	53115502	-	-	-	CC_hom	-
frameshift deletion	TTC3	chr21	38524243	38524244	AA	AA	-_het	AA	AA
frameshift deletion	TTC39A	chr1	51767913	51767913	C	C	-_hom	C	C
frameshift deletion	TTK	chr6	80751910	80751910	A	A	-_het	A	A
frameshift deletion	TTLL10	chr1	1116223	1116223	G	G	-_hom	G	G
frameshift	TTLL13	chr15	90799090	90799090	-	-	-	-	C_het

insertion									
frameshift insertion	TTN	chr2	179527086	179527086	-	-	T_het	-	-
frameshift deletion	TUBA3D	chr2	132237787	132237787	C	C	-_het	C	C
frameshift insertion	TUBA8	chr22	18613684	18613684	-	-	-	-	AT_het
frameshift deletion	TUBA8	chr22	18613688	18613689	AG	AG	AG	AG	-_het
frameshift deletion	TUBB8	chr10	93444	93444	A	A	-_het	A	A
frameshift deletion	TUBGCP4	chr15	43678086	43678086	A	A	-_het	A	A
frameshift deletion	TVP23A	chr16	10867203	10867203	A	A	-_hom	A	A
frameshift deletion	TXNDC5	chr6	7891867	7891868	AC	AC	-_het	AC	AC
frameshift deletion	UBA5	chr3	132394449	132394449	A	A	-_het	A	A
frameshift deletion	UBR5	chr8	103289349	103289349	T	T	-_het	T	T
frameshift deletion	UBXN11	chr1	26608826	26608832	GGGGCCG	GGGGCCG	-_hom	-_hom	-_het
frameshift deletion	UBXN11	chr1	26608836	26608852	CCGGGACCGGGACTGGG	CCGGGACCGGGACTGGG	-_hom	-_hom	-_het
frameshift deletion	UBXN11	chr1	26608866	26608867	CC	-_hom	CC	CC	-_het
frameshift deletion	UBXN11	chr1	26608869	26608896	GGACTGGGGCCGGGACCGGGACCGGGAC	-_hom	GGACTGGGGCCGGGACCGGGACCGGGAC	GGACTGGGGCCGGGACCGGGACCGGGAC	-_het
frameshift deletion	UGT1A3	chr2	234638283	234638283	T	T	-_het	T	T

frameshift insertion	UNC80	chr2	210778534	210778534	-	-	T_het	-	-
frameshift deletion	UNKL	chr16	1447265	1447351	CCCCGTGCTTCACACTGGGGCAGGGCGTGGACCTGGGGATGAGGAGGTGTCAGGGGGACACAGAGGACTTGGCTCCCCGCCCCCCAC	-_hom	CCCCGTGCTTCACACTGGGGCAGGGCGTGGACCTGGGGATGAGGAGGTGTCAGGGGGACACAGAGGACTTGGCTCCCCGCCCCCCAC	CCCCGTGCTTCACACTGGGGCAGGGCGTGGACCTGGGGATGAGGAGGTGTCAGGGGGACACAGAGGACTTGGCTCCCCGCCCCCCAC	CCCCGTGCTTCACACTGGGGCAGGGCGTGGACCTGGGGATGAGGAGGTGTCAGGGGGACACAGAGGACTTGGCTCCCCGCCCCCCAC
frameshift deletion	UPF3A	chr13	115057211	115057211	A	A	-_het	A	A
frameshift deletion	USP1	chr1	62910505	62910505	A	A	-_het	A	A
frameshift insertion	USP13	chr3	179472610	179472610	-	-	C_het	-	-
frameshift deletion	USP35	chr11	77911710	77911710	C	C	-_het	C	C
frameshift deletion	USP35	chr11	77920856	77920856	C	C	-_het	C	C
frameshift deletion	USP45	chr6	99916420	99916420	T	-_het	T	T	T
frameshift deletion	USP48	chr1	22033386	22033387	AA	AA	-_hom	AA	AA
frameshift insertion	WASF3	chr13	27255386	27255386	-	-	C_het	-	-
frameshift deletion	WBP1	chr2	74687410	74687410	C	C	-_het	C	C
frameshift deletion	WDR55	chr5	140049102	140049102	A	A	-_het	A	A
frameshift deletion	WDR61	chr15	78587761	78587770	GTACAACATC	GTACAACATC	GTACAACATC	GTACAACATC	-_het

frameshift insertion	VENTXP7	chr3	21447901	21447901	-	-	C_hom	-	-
frameshift deletion	VEPH1	chr3	157081227	157081227	T	T	-_het	T	T
frameshift deletion	VIP	chr6	153076471	153076471	A	A	-_het	A	A
frameshift insertion	WNK1	chr12	974308	974308	-	C_hom	C_het	C_het	-
frameshift deletion	WNK4	chr17	40945875	40945876	TT	TT	-_het	TT	TT
frameshift deletion	WNT16	chr7	120971879	120971879	G	G	-_het	G	G
frameshift deletion	VWA3B	chr2	98907090	98907090	T	T	-_het	T	T
frameshift deletion	VWA3B	chr2	98928507	98928507	G	G	-_het	G	G
frameshift deletion	VWDE	chr7	12443288	12443288	C	C	-_het	C	C
frameshift insertion	X97876	chr9	66499823	66499823	-	-	-	-	TC_het
frameshift deletion	XKR6	chr8	11058234	11058234	G	G	-_het	G	G
frameshift deletion	XRN1	chr3	142142409	142142409	T	T	-_het	T	T
frameshift insertion	YBEY	chr21	47711279	47711279	-	T_het	-	-	-
frameshift deletion	ZBTB20	chr3	114058003	114058003	G	G	-_het	G	G
frameshift insertion	ZFP36L2	chr2	43452622	43452622	-	-	C_het	-	-
frameshift deletion	ZFYVE16	chr5	79734010	79734010	T	T	-_het	T	T
frameshift	ZGLP1	chr19	10416578	10416578	C	C	-_het	C	C

deletion									
frameshift deletion	ZKSCAN4	chr6	28217592	28217592	C	C	-_het	C	C
frameshift deletion	ZMYND8	chr20	45867410	45867410	A	A	-_het	A	A
frameshift deletion	ZNF106	chr15	42742957	42742957	A	A	-_het	A	A
frameshift insertion	ZNF141	chr4	338197	338197	-	-	-	GA_het	GA_het
frameshift deletion	ZNF141	chr4	338202	338203	TC	TC	TC	-_het	-_het
frameshift insertion	ZNF142	chr2	219508768	219508768	-	-	C_het	-	-
frameshift deletion	ZNF169	chr9	97062423	97062423	G	G	-_het	G	G
frameshift insertion	ZNF175	chr19	52084690	52084690	-	-	-	-	G_het
frameshift deletion	ZNF257	chr19	22255637	22255638	TG	TG	TG	-_het	TG
frameshift insertion	ZNF335	chr20	44596241	44596241	-	-	T_het	-	-
frameshift deletion	ZNF33B	chr10	43088712	43088712	A	A	-_het	A	A
frameshift deletion	ZNF343	chr20	2473385	2473385	T	T	T	T	-_het
frameshift deletion	ZNF385C	chr17	40180106	40180107	AG	AG	-_het	AG	AG
frameshift deletion	ZNF407	chr18	72775787	72775787	C	C	-_het	C	C
frameshift deletion	ZNF429	chr19	21720435	21720435	A	A	-_het	A	A
frameshift deletion	ZNF443	chr19	12542364	12542364	A	A	-_het	A	A

frameshift insertion	ZNF486	chr19	20308051	20308051	-	-	AA_het	-	-
frameshift deletion	ZNF491	chr19	11917954	11917955	AT	AT	-_het	AT	AT
frameshift deletion	ZNF514	chr2	95815035	95815035	T	T	-_het	T	T
frameshift deletion	ZNF571	chr19	38046867	38046870	GTAA	GTAA	GTAA	GTAA	-_het
frameshift insertion	ZNF595	chr4	87313	87313	-	A_hom	A_hom	A_hom	A_hom
frameshift deletion	ZNF608	chr5	123984684	123984684	C	C	-_het	C	C
frameshift deletion	ZNF626	chr19	20807134	20807300	GCTTTGCCACATTCTTCACATTTGTAGAATTTCTCTCCAGTATGATTCTCTCATGTGTAGTAAGGATTGAGGACTGGTTGAAGGCTTTGCCACATTCTTCACATTTGTAGGGTCTCTCTCCAGTATGAATTTTCTTATGTGTAGTAAGGTTAGAGGAGCACTTAAAA	GCTTTGCCACATTCTTCACATTTGTAGAATTTCTCTCCAGTATGATTCTCTCATGTGTAGTAAGGATTGAGGACTGGTTGAAGGCTTTGCCACATTCTTCACATTTGTAGGGTCTCTCTCCAGTATGAATTTTCTTATGTGTAGTAAGGTTAGAGGAGCACTTAAAA	-_het	GCTTTGCCACATTCTTCACATTTGTAGAATTTCTCTCCAGTATGATTCTCTCATGTGTAGTAAGGATTGAGGACTGGTTGAAGGCTTTGCCACATTCTTCACATTTGTAGGGTCTCTCTCCAGTATGAATTTTCTTATGTGTAGTAAGGTTAGAGGAGCACTTAAAA	GCTTTGCCACATTCTTCACATTTGTAGAATTTCTCTCCAGTATGATTCTCTCATGTGTAGTAAGGATTGAGGACTGGTTGAAGGCTTTGCCACATTCTTCACATTTGTAGGGTCTCTCTCCAGTATGAATTTTCTTATGTGTAGTAAGGTTAGAGGAGCACTTAAA
frameshift deletion	ZNF660	chr3	44636279	44636280	AT	-_hom	AT	AT	AT
frameshift deletion	ZNF717	chr3	75786761	75786761	C	-_het	-_hom	-_het	-_het
frameshift	ZNF717	chr3	75786895	75786895	T	-_het	-_hom	-_het	-_het

deletion									
stopgain	ZNF717	chr3	75790783	75790783	-	GTCTCCAGCATCATGTCCCTGTACAGGTTTCTTCTGAGCATCATCCAGGTCCTGCTACTCCTCCCAGGTGAAGTGCATAGCTACCTCCTCAAAGGATGCCAACTCCTGTAATGATAGCAGGCTGTTATAA_het	GTCTCCAGCATCATGTCCCTGTACAGGTTTCTTCTGAGCATCATCCAGGTCCTGCTACTCCTCCCAGGTGAAGTGCATAGCTACCTCCTCAAAGGATGCCAACTCCTGTAATGATAGCAGGCTGTTATAA_hom	GTCTCCAGCATCATGTCCCTGTACAGGTTTCTTCTGAGCATCATCCAGGTCCTGCTACTCCTCCCAGGTGAAGTGCATAGCTACCTCCTCAAAGGATGCCAACTCCTGTAATGATAGCAGGCTGTTATAA_het	GTCTCCAGCATCATGTCCCTGTACAGGTTTCTTCTGAGCATCATCCAGGTCCTGCTACTCCTCCCAGGTGAAGTGCATAGCTACCTCCTCAAAGGATGCCAACTCCTGTAATGATAGCAGGCTGTTATAA_het
frameshift deletion	ZNF77	chr19	2939268	2939289	ACCACCCTTACCCAAGGAGGCA	ACCACCCTTACCCAAGGAGGCA	ACCACCCTTACCCAAGGAGGCA	ACCACCCTTACCCAAGGAGGCA	-_het
frameshift insertion	ZNF799	chr19	12501371	12501371	-	-	T_het	-	-
frameshift deletion	ZNF800	chr7	127014068	127014068	T	T	-_het	T	T
frameshift insertion	ZNF814	chr19	58385707	58385707	-	-	T_het	-	-
frameshift deletion	ZNF83	chr19	53116934	53117017	CCACACTCATTACATTTGTAAGGTTTCTCTCCAGTGTGGATTCTCTGATGTTGTGCAAGGTGTGAAATATGATGGAAGACCTTT	CCACACTCATTACATTTGTAAGGTTTCTCTCCAGTGTGGATTCTCTGATGTTGTGCAAGGTGTGAAATATGATGGAAGACCTTT	CCACACTCATTACATTTGTAAGGTTTCTCTCCAGTGTGGATTCTCTGATGTTGTGCAAGGTGTGAAATATGATGGAAGACCTTT	-_het	CCACACTCATTACATTTGTAAGGTTTCTCTCCAGTGTGGATTCTCTGATGTTGTGCAAGGTGTGAAATATGATGGAAGACCTTT
frameshift deletion	ZNF880	chr19	52887146	52887146	A	-_het	-_het	A	-_het

frameshift deletion	ZNF880	chr19	52887375	52887375	A	A	-_het	A	A
frameshift insertion	ZNF880	chr19	52888070	52888070	-	-	-	-	TTGGGAGGCCGAGGCGG_het
frameshift insertion	ZNF880	chr19	52888074	52888074	-	-	-	-	AT_het
frameshift deletion	ZNF890P	chr7	5172096	5172097	TT	-_het	-_het	-_het	TT
frameshift insertion	ZNF890P	chr7	5172099	5172099	-	-	TG_het	TG_het	-
frameshift insertion	ZSWIM5	chr1	45672021	45672021	-	-	T_het	-	-
