# Supplementary material for: The neuroendocrine phenotype, genomic profile and therapeutic sensitivity of GEPNET cell lines
Source: Endocr Relat Cancer. 2018 Jan 15;25(3):367–80. doi: 10.1530/ERC-17-0445 (PMC5827037; doi:10.1530/ERC-17-0445)
Supplement: Supplementary Table 4 [file erc-25-309-t004.pdf]

Supplementary Table 4. SNP and indel mutations in TCGA cancer-associated genes.

Annotation	Gene	Chr	Position 1	Position 2	Ref	GOT1	P-STS	BON1	QGP-1
frameshift deletion	ACVR2A	chr2	148683686	148683686	A	A	-_hom	A	A
nonsynonymous SNV	APC	chr5	112175240	112175240	G	G	G	C_het	G
nonsynonymous SNV	APC	chr5	112177788	112177788	G	G	G	G	A_het
nonsynonymous SNV	ARID1A	chr1	27105886	27105886	C	C	T_het	C	C
frameshift deletion	ARID1A	chr1	27106074	27106074	G	G	-_het	G	G
nonsynonymous SNV	ARID5B	chr10	63851054	63851054	C	C	C	C	T_het
nonsynonymous SNV	ASXL1	chr20	31024561	31024561	T	T	T	C_het	T
nonsynonymous SNV	ATM	chr11	108206666	108206666	A	A	A	A	T_het
nonsynonymous SNV	ATR	chr3	142232399	142232399	G	A_het	G	G	G
frameshift deletion	ATR	chr3	142274740	142274740	T	T	-_het	T	T
nonsynonymous SNV	ATRX	chrX	76856021	76856021	T	C_het	T	T	T
nonsynonymous SNV	ATRX	chrX	76938208	76938208	A	A	A	A	G_hom
frameshift deletion	AXIN2	chr17	63533905	63533905	C	C	-_het	C	C
frameshift insertion	AXIN2	chr17	63545677	63545677	-	-	C_het	-	-
nonsynonymous SNV	BRCA1	chr17	41199702	41199702	C	C	T_het	C	C
nonsynonymous SNV	BRCA2	chr13	32907398	32907398	C	C	T_het	C	C
nonsynonymous SNV	BRCA2	chr13	32914371	32914371	G	G	G	A_het	G
nonsynonymous SNV	CDKN1A	chr6	36651889	36651889	C	C	C	T_hom	C
nonsynonymous SNV	CHEK2	chr22	29091147	29091147	A	A	A	C_hom	A
frameshift insertion	CRIPAK	chr4	1388350	1388350	-	TGCCCATGTGGAGTGCCCGCCTGCTCACACA_hom	TGCCCATGTGGAGTGCCCGCCTGCTCACACA_het	-	-

frameshift deletion	CRIPAK	chr4	1388594	1388622	CACGTGCCCATGTGGAGTGCCCGCCTGCT	-_hom	-_het	CACGTGCCCATGTGGAGTGCCCGCCTGCT	CACGTGCCCATGTGGAGTGCCCGCCTGCT
nonsynonymous SNV	CRIPAK	chr4	1388817	1388817	C	C	A_hom	C	C
frameshift deletion	CRIPAK	chr4	1388944	1388947	GTGG	GTGG	GTGG	-_hom	GTGG
frameshift deletion	CRIPAK	chr4	1388949	1389037	GTGTTCGCCTGCTCACACGTGCCGATGCGGAGTGCCCGCCTGCTCACACGTGCCGATGCGGAGTGCCCGCCTGCTCACACGTGCCCATG	GTGTTCGCCTGCTCACACGTGCCGATGCGGAGTGCCCGCCTGCTCACACGTGCCGATGCGGAGTGCCCGCCTGCTCACACGTGCCCATG	GTGTTCGCCTGCTCACACGTGCCGATGCGGAGTGCCCGCCTGCTCACACGTGCCGATGCGGAGTGCCCGCCTGCTCACACGTGCCCATG	-_hom	GTGTTCGCCTGCTCACACGTGCCGATGCGGAGTGCCCGCCTGCTCACACGTGCCGATGCGGAGTGCCCGCCTGCTCACACGTGCCCATG
frameshift deletion	CTCF	chr16	67645339	67645339	A	A	-_het	A	A
nonsynonymous SNV	DNMT3A	chr2	25466773	25466773	C	C	T_het	C	C
nonsynonymous SNV	EP300	chr22	41546041	41546041	C	C	C	T_hom	C
frameshift insertion	EPHB6	chr7	142562308	142562308	-	-	G_het	-	-
nonsynonymous SNV	EPPK1	chr8	144941903	144941903	G	G	G	A_het	G
frameshift deletion	EPPK1	chr8	144946136	144946139	TGAG	TGAG	TGAG	-_het	TGAG
nonsynonymous SNV	FBXW7	chr4	153332559	153332559	T	T	T	C_hom	T
nonsynonymous SNV	FLT3	chr13	28623587	28623587	C	C	T_het	C	C
nonsynonymous SNV	KIT	chr4	55593449	55593449	A	A	C_het	A	A
nonsynonymous SNV	KRAS	chr12	25398284	25398284	C	C	C	C	A_het
nonsynonymous SNV	LIFR	chr5	38502757	38502757	G	G	A_het	G	G

stopgain	LRRK2	chr12	40702279	40702279	C	C	C	T_het	C
nonsynonymous SNV	MAP3K1	chr5	56177843	56177843	C	C	G_het	C	C
nonsynonymous SNV	MTOR	chr1	11190767	11190767	C	C	C	C	T_het
nonsynonymous SNV	NAV3	chr12	78512060	78512060	G	G	G	A_het	G
frameshift deletion	NCOR1	chr17	15983844	15983845	AT	-_het	AT	AT	AT
nonsynonymous SNV	NCOR1	chr17	16068340	16068340	C	T_het	T_het	C	C
stopgain	NCOR1	chr17	16068343	16068343	G	A_het	A_het	G	G
nonsynonymous SNV	NCOR1	chr17	16068377	16068377	C	G_het	G_het	G_het	G_het
nonsynonymous SNV	NCOR1	chr17	16068396	16068396	G	A_het	A_het	A_het	A_het
nonsynonymous SNV	NCOR1	chr17	16068463	16068463	C	C	C	C	T_het
nonsynonymous SNV	NFE2L3	chr7	26224637	26224637	C	G_het	C	C	C
nonsynonymous SNV	NOTCH1	chr9	139396468	139396468	C	C	A_het	C	C
nonsynonymous SNV	NOTCH1	chr9	139396913	139396913	G	G	A_het	G	G
nonsynonymous SNV	NRAS	chr1	115256529	115256529	T	T	T	C_hom	T
nonsynonymous SNV	NSD1	chr5	176638468	176638468	G	G	T_het	G	G
nonsynonymous SNV	PRX	chr19	40900457	40900457	C	C	C	G_het	C
nonsynonymous SNV	RAD21	chr8	117864867	117864867	A	A	A	C_het	A
nonsynonymous SNV	RAD21	chr8	117868995	117868995	C	C	C	G_het	C
frameshift insertion	RB1	chr13	48881488	48881488	-	-	AG_het	-	-
frameshift insertion	RB1	chr13	48955464	48955464	-	-	T_het	-	-
nonsynonymous SNV	RPL22	chr1	6257744	6257744	C	C	C	C	A_het
frameshift insertion	RPL22	chr1	6257784	6257784	-	-	T_het	-	-
nonsynonymous SNV	SETD2	chr3	47163843	47163843	C	C	C	C	T_het
nonsynonymous SNV	SETD2	chr3	47164507	47164507	C	C	T_hom	C	C
nonsynonymous SNV	SIN3A	chr15	75693078	75693078	C	C	T_het	C	C
nonsynonymous SNV	SMAD4	chr18	48575096	48575096	G	G	A_het	G	G
frameshift deletion	SMAD4	chr18	48604788	48604791	ACGA	ACGA	ACGA	-_hom	ACGA
nonsynonymous SNV	SMC1A	chrX	53423450	53423450	C	T_het	C	C	C
nonsynonymous SNV	TBX3	chr12	115117353	115117353	G	T_het	G	G	G

nonsynonymous SNV	TET2	chr4	106157435	106157435	C	C	C	C	G_het
nonsynonymous SNV	TET2	chr4	106157703	106157703	T	T	T	T	G_het
nonsynonymous SNV	TET2	chr4	106196770	106196770	G	G	A_het	G	G
frameshift deletion	TGFBR2	chr3	30691872	30691872	A	A	-_hom	A	A
frameshift deletion	TLR4	chr9	120470861	120470861	T	-_het	T	T	T
stopgain	TP53	chr17	7574003	7574003	G	G	G	A_hom	G
nonsynonymous SNV	TP53	chr17	7577121	7577121	G	G	A_hom	G	G
frameshift deletion	TP53	chr17	7579394	7579394	G	G	G	G	-_hom
