# Supplementary material for: The neuroendocrine phenotype, genomic profile and therapeutic sensitivity of GEPNET cell lines
Source: Endocr Relat Cancer. 2018 Jan 15;25(3):367–80. doi: 10.1530/ERC-17-0445 (PMC5827037; doi:10.1530/ERC-17-0445)
Supplement: Supplementary Table 5 [file erc-25-309-t005.pdf]

Supplementary Table 5. Inhibitor screening of GEPNET cell lines.

				GOT1		BON-1		QGP-1		P-STS	
**Product Name**	**CAS Number**	**Form**	**Targets**	**% of control**	**Z-score**	**% of control**	**Z-score**	**% of control**	**Z-score**	**% of control**	**Z-score**
**(-)-Blebbistatin**	856925-71-8	free base	ATPase	77.9	-0.36	77.6	-0.31			95.6	0.09
**(-)-MK 801 Maleate**	121917-57-5	maleate	GluR	93.9	0.32	114.8	1.05	97.5	-0.04	77.1	-0.50
**(-)-Parthenolide**	20554-84-1	free base	E3 Ligase	107.6	0.90	77.7	-0.30	85.6	-0.54	135.5	1.35
**(-)-p-Bromotetramisole Oxalate**	62284-79-1	free base	Others	85.7	-0.03	97.3	0.41	110.1	0.49	113.0	0.64
**(+)-Bicuculline**	485-49-4	free base	GABA Receptor	85.1	-0.05	99.2	0.48	76.5	-0.93	120.0	0.86
**(+)-JQ1**	1268524-70-4	free base	Epigenetic Reader Domain	13.8	-3.08	35.4	-1.84			3.8	-2.82
**(+)-Matrine**	519-02-8	free base	Opioid Receptor	121.8	1.50	102.0	0.58	83.6	-0.63	124.5	1.00
**(R)-Nepicastat HCl**	195881-94-8	Salt	Hydroxylase	87.9	0.06	65.7	-0.74	81.7	-0.71	127.5	1.10
**(S)-crizotinib**	1374356-45-2	free base	MTH	107.2	0.88	79.4	-0.24	105.1	0.28	97.0	0.13
**10058-F4**	403811-55-2	free base	c-Myc	82.3	-0.18	97.2	0.41			99.2	0.20
**17-AAG (Tanespimycin)**	75747-14-7	free base	HSP (e.g. HSP90)	67.9	-0.78	26.5	-2.16	59.2	-1.67	53.4	-1.25
**17-DMAG (Alvespimycin) HCl**	467214-21-7	hydrochloride	HSP (e.g. HSP90)	39.4	-1.99	26.4	-2.17	41.7	-2.41	21.9	-2.25
**1-Azakenpaullone**	676596-65-9	free base	GSK-3	64.5	-0.93	55.0	-1.13			113.8	0.66
**2-Methoxyestradiol (2-MeOE2)**	362-07-2	free base	HIF	93.0	0.28	81.3	-0.17	116.6	0.77	94.9	0.07
**3-Aminobenzamide**	3544-24-9	free base	PARP	72.5	-0.59	74.6	-0.42	85.1	-0.57	116.1	0.74
**3-Deazaneplanocin A (DZNeP)**	102052-95-9	free base	Histone Methyltransferase	75.4	-0.47	64.8	-0.77			105.3	0.40
**3-Methyladenine**	5142-23-4	free base	Autophagy,PI3K	114.2	1.18	64.4	-0.79	105.3	0.29	109.4	0.52
**4E1RCat**	328998-25-0	free base	ELF4	134.8	2.05	109.3	0.85	111.9	0.57	87.9	-0.15
**4EGI-1**	315706-13-9	free base	ELF4	80.9	-0.23	116.5	1.11	101.3	0.12	128.6	1.13
**5-hydroxymethyl Tolterodine (PNU 200577, 5-HMT, 5-HM)**	207679-81-0	free base	AChR	120.1	1.43	79.3	-0.24	111.2	0.54	87.2	-0.18
**6H05**	No	free base	Rho	90.1	0.16	116.8	1.12	108.4	0.42	132.9	1.27
**A66**	1166227-08-2	free base	PI3K	60.3	-1.11	114.1	1.02	78.7	-0.84	115.3	0.71
**A-674563**	552325-73-2	free base	PKA,CDK,Akt	51.0	-1.50	114.8	1.05	94.7	-0.16	59.7	-1.05
**A-769662**	844499-71-4	free base	AMPK	103.3	0.71	101.6	0.57	118.9	0.87	121.1	0.90

**A-803467**	944261-79-4	free base	Sodium Channel	119.4	1.40	105.0	0.69	89.5	-0.38	108.2	0.49
**A922500**	959122-11-3	free base	Transferase	85.0	-0.06	66.4	-0.71	62.4	-1.53	110.6	0.56
**ABT-199 (GDC-0199)**	1257044-40-8	free base	Bcl-2	68.4	-0.76	63.8	-0.81	102.9	0.19	41.2	-1.63
**ABT-263 (Navitoclax)**	923564-51-6	free base	Bcl-2	123.3	1.56	114.4	1.03	86.1	-0.52	4.2	-2.81
**ABT-737**	852808-04-9	free base	Autophagy,Bcl-2	128.9	1.80	114.2	1.03	97.6	-0.04	3.8	-2.82
**AC480 (BMS-599626)**	714971-09-2	free base	HER2,EGFR	93.7	0.31	105.0	0.69	113.8	0.65	106.6	0.44
**Acadesine**	2627-69-2	free base	AMPK	101.8	0.65	113.6	1.01	102.0	0.15	93.1	0.01
**Acebutolol HCl**	34381-68-5	hydrochloride	Adrenergic Receptor	94.2	0.33	115.3	1.06	111.8	0.57	135.6	1.35
**Acemetacin**	53164-05-9	free base	COX	85.6	-0.03	49.6	-1.32	122.5	1.02	107.6	0.47
**Acetylcholine Chloride**	60-31-1	chloride	AChR	108.1	0.92	105.1	0.69	108.3	0.42	92.0	-0.03
**Acetylcysteine**	616-91-1	free base	AChR	83.6	-0.12	65.4	-0.75	89.1	-0.40	98.0	0.16
**Aclidinium Bromide**	320345-99-1	free base	AChR	96.8	0.44	112.5	0.96	100.9	0.10	128.7	1.14
**Adefovir Dipivoxil**	142340-99-6	free base	Reverse Transcriptase	115.8	1.25	99.8	0.50	98.8	0.02	28.4	-2.04
**ADL5859 HCl**	850173-95-4	hydrochloride	Opioid Receptor	84.1	-0.10	116.4	1.11	110.1	0.49	64.2	-0.91
**Adrenalone HCl**	62-13-5	hydrochloride	Adrenergic Receptor	94.9	0.36	118.5	1.18	109.3	0.46	129.8	1.17
**ADX-47273**	851881-60-2	free base	GluR	82.6	-0.16	51.3	-1.26	120.8	0.95	130.7	1.20
**AEBSF HCl**	30827-99-7	Salt	Serine Protease	92.6	0.26	57.3	-1.04	102.4	0.17	112.3	0.62
**AEE788 (NVP-AEE788)**	497839-62-0	free base	HER2,VEGFR,EGFR	100.1	0.58	115.8	1.08	119.9	0.91	96.3	0.11
**Afatinib (BIBW2992)**	439081-18-2	free base	EGFR,HER2	100.7	0.61	105.4	0.71	96.0	-0.11	90.9	-0.06
**AG-1024**	65678-07-1	free base	IGF-1R	100.0	0.58	102.3	0.59	85.5	-0.55	93.0	0.01
**AG-14361**	328543-09-5	free base	PARP	106.4	0.85	55.0	-1.13	96.1	-0.10	77.9	-0.47
**AG-1478 (Tyrphostin AG-1478)**	153436-53-4	free base	EGFR	99.2	0.54	105.6	0.71	119.4	0.89	123.9	0.99
**AG-18**	118409-57-7	free base	EGFR	85.7	-0.03	66.5	-0.71	106.2	0.33	86.8	-0.19
**AG-490 (Tyrphostin B42)**	133550-30-8	free base	JAK,EGFR	102.6	0.69	117.0	1.13	117.2	0.80	93.4	0.02
**AGI-5198**	1355326-35-0	free base	Dehydrogenase	86.7	0.01	77.8	-0.30			114.5	0.69
**AGI-6780**	1432660-47-3	free base	IDH2	70.6	-0.67	107.4	0.78			102.6	0.31
**Agomelatine**	138112-76-2	free base	5-HT Receptor	113.7	1.16	112.9	0.98	111.5	0.55	67.1	-0.81
**Alfuzosin HCl**	81403-68-1	hydrochloride	Adrenergic Receptor	90.0	0.15	56.3	-1.08	108.9	0.44	91.8	-0.03
**Alisertib (MLN8237)**	1028486-01-2	free base	Aurora Kinase	91.9	0.23	73.8	-0.44	71.1	-1.16	60.7	-1.02

**Aliskiren Hemifumarate**	173334-58-2	fumarate	RAAS	87.6	0.05	66.7	-0.70	91.6	-0.29	124.8	1.01
**Alizarin**	72-48-0	free base	P450 (e.g. CYP17)	94.8	0.36	51.8	-1.25	118.4	0.85	123.8	0.98
**Allopurinol**	315-30-0	free base	OX Receptor	107.6	0.90	114.9	1.05	110.5	0.51	94.8	0.06
**Almorexant HCl**	913358-93-7	HCl	OX Receptor	114.8	1.20	67.7	-0.67	106.7	0.35	100.2	0.23
**Almotriptan Malate**	181183-52-8	malate	5-HT Receptor	134.3	2.03	114.4	1.03	97.0	-0.06	92.4	-0.01
**Alogliptin**	850649-61-5	free base	DPP-4	83.7	-0.12	68.0	-0.65	128.1	1.26	103.6	0.34
**Aloxistatin**	88321-09-9	free base	Cysteine protease	72.4	-0.59	117.1	1.13	99.5	0.04	69.1	-0.75
**Altrenogest**	850-52-2	free base	Estrogen/progestogen Receptor	93.6	0.30	114.7	1.04	115.4	0.72	125.6	1.04
**Alvelestat (AZD9668)**	848141-11-7	free base	Serine Protease	77.8	-0.36	79.4	-0.24			133.0	1.27
**AM1241**	444912-48-5	free base	Cannabinoid Receptor	101.5	0.64	114.8	1.05	110.4	0.51	84.0	-0.28
**AM251**	183232-66-8	free base	Cannabinoid Receptor	89.7	0.14	92.4	0.23	108.3	0.42	116.8	0.76
**Amantadine HCl**	665-66-7	hydrochloride	Dopamine Receptor	111.7	1.07	108.8	0.83	98.7	0.01	115.9	0.73
**Ambroxol HCl**	23828-92-4	hydrochloride	Sodium Channel	89.6	0.14	93.1	0.26	87.3	-0.47	117.7	0.79
**Amfebutamone HCl**	31677-93-7	hydrochloride	Dopamine Receptor,AChR	105.8	0.82	109.7	0.86	138.5	1.70	111.9	0.60
**AMG-458**	913376-83-7	free base	c-Met	74.7	-0.49	77.0	-0.33	86.1	-0.52	111.7	0.60
**AMG-517**	659730-32-2	free base	TRPV	80.7	-0.24	93.4	0.27			120.2	0.87
**AMG-900**	945595-80-2	free base	Aurora Kinase	74.8	-0.49	51.5	-1.25	68.3	-1.28	90.4	-0.08
**Aminoglutethimide**	125-84-8	free base	Aromatase	129.1	1.81	99.9	0.50	104.4	0.25	72.7	-0.64
**Aminophylline**	317-34-0	ethylenediamine	PDE	84.7	-0.07	112.8	0.97	100.6	0.09	93.6	0.03
**Amiodarone HCl**	19774-82-4	hydrochloride	Autophagy,Potassium Channel	89.3	0.12	114.9	1.05	101.5	0.13	80.2	-0.40
**Amisulpride**	71675-85-9	free base	Dopamine Receptor	70.3	-0.68	82.2	-0.14	137.7	1.67	94.3	0.05
**Amitriptyline HCl**	549-18-8	hydrochloride	5-HT Receptor	90.9	0.19	118.0	1.16	106.2	0.33	138.2	1.44
**Amlodipine**	88150-42-9	free base	Calcium Channel	91.6	0.22	116.6	1.11	103.6	0.22	156.6	2.02
**Amonafide**	69408-81-7	free base	Topoisomerase	69.9	-0.70	113.6	1.00	78.4	-0.85	81.4	-0.36
**Ampiroxicam**	99464-64-9	free base	COX	99.2	0.54	118.0	1.16	112.5	0.60	128.5	1.13
**Amprenavir**	161814-49-9	free base	HIV Protease	88.1	0.07	113.3	0.99	109.6	0.47	91.3	-0.05
**Amuvatinib (MP-470)**	850879-09-3	free base	FLT3,c-RET,PDGFR,c-Kit	97.7	0.48	81.8	-0.15	122.3	1.01	58.0	-1.10
**Anacetrapib (MK-0859)**	875446-37-0	free base	CETP	81.4	-0.21	63.4	-0.82	116.9	0.78	125.3	1.03

**Anagrelide HCl**	58579-51-4	hydrochloride	PDE	92.1	0.24	118.6	1.18	111.3	0.54	122.0	0.92
**Anastrozole**	120511-73-1	free base	Aromatase	83.7	-0.11	112.4	0.96	113.0	0.62	77.6	-0.48
**Andarine**	401900-40-1	free base	Androgen Receptor	101.7	0.65	117.3	1.14	115.2	0.71	57.1	-1.13
**Anidulafungin (LY303366)**	166663-25-8	free base	Others	100.7	0.61	104.5	0.67	86.9	-0.49	92.8	0.00
**Aniracetam**	72432-10-1	free base	AMPA Receptor-kainate Receptor-NMDA Receptor	96.8	0.44	73.1	-0.47	119.5	0.89	94.1	0.04
**AP26113**	1197958-12-5	free base	ALK	54.9	-1.33	60.9	-0.91	64.6	-1.44	108.9	0.51
**Apatinib**	811803-05-1	sulfate	VEGFR	113.1	1.13	113.6	1.00	100.1	0.07	92.9	0.00
**Apigenin**	520-36-5	free base	P450 (e.g. CYP17)	108.5	0.93	107.3	0.77	82.6	-0.67	119.0	0.83
**Apixaban**	503612-47-3	free base	Factor Xa	89.2	0.12	114.8	1.05	90.2	-0.35	90.7	-0.07
**Apoptosis Activator 2**	79183-19-0	free base	Caspase	78.8	-0.32	64.9	-0.77	129.1	1.30	104.6	0.37
**Apremilast (CC-10004)**	608141-41-9	free base	PDE	89.2	0.12	64.5	-0.78	112.1	0.58	96.1	0.10
**Aprepitant**	170729-80-3	free base	Substance P	90.6	0.18	104.5	0.67	117.4	0.80	71.9	-0.66
**AR-42**	935881-37-1	free base	HDAC	26.9	-2.52	39.1	-1.70	46.4	-2.21	0.2	-2.93
**AR-A014418**	487021-52-3	free base	GSK-3	78.5	-0.33	116.7	1.12	129.3	1.31	73.5	-0.61
**Arecoline**	300-08-3	HBr	AChR	85.8	-0.03	56.3	-1.08	104.0	0.24	125.4	1.03
**Aripiprazole**	129722-12-9	free base	5-HT Receptor	95.9	0.40	106.4	0.74	100.5	0.08	155.0	1.97
**ARN-509**	956104-40-8	free base	Androgen Receptor	88.3	0.08	77.1	-0.32	111.6	0.56	108.5	0.50
**ARQ 621**	1095253-39-6	free base	Kinesin	64.4	-0.93	77.2	-0.32	45.6	-2.24	58.6	-1.08
**AS-252424**	900515-16-4	free base	PI3K	67.5	-0.80	115.8	1.08	112.5	0.60	83.0	-0.31
**AS-604850**	648449-76-7	free base	PI3K	85.0	-0.06	53.2	-1.19	113.2	0.63	125.2	1.03
**Asaraldehyde**	4460-86-0	free base	COX	103.9	0.74	70.0	-0.58	118.5	0.85	117.0	0.77
**Asenapine**	85650-56-2	maleate	5-HT Receptor,Adrenergic Receptor	90.9	0.19	54.9	-1.13	113.6	0.64	94.1	0.04
**Asiatic Acid**	464-92-6	free base	p38 MAPK	108.7	0.94	69.8	-0.59	89.5	-0.38	114.3	0.68
**Aspirin**	50-78-2	free base	Proteasome	151.0	2.74	111.9	0.94	109.7	0.48	93.3	0.02
**AST-1306**	1050500-29-2	mesylate	EGFR	80.6	-0.25	64.8	-0.77	73.6	-1.06	77.0	-0.50
**Astragaloside A**	83207-58-3	free base	TGF-beta/Smad	104.7	0.77	102.9	0.61	82.9	-0.66	114.7	0.69
**Astragaloside A**	83207-58-3	free base	TGF-beta/Smad	103.1	0.71	89.5	0.13	87.7	-0.46	117.9	0.79
**AT101**	866541-93-7	free base	Bcl-2	75.5	-0.46	113.6	1.00	118.6	0.85	112.1	0.61

**AT13387**	912999-49-6	free base	HSP (e.g. HSP90)	55.4	-1.32	29.3	-2.06	43.4	-2.34	38.6	-1.72
**AT7519**	844442-38-2	free base	CDK	59.5	-1.14	27.1	-2.14	31.3	-2.86	19.4	-2.32
**AT7867**	857531-00-1	free base	S6 Kinase,Akt	86.2	-0.01	115.4	1.07	115.7	0.73	108.3	0.49
**AT9283**	896466-04-9	free base	JAK,Aurora Kinase,Bcr-Abl	77.0	-0.40	109.8	0.87	60.3	-1.62	88.1	-0.15
**Ataluren (PTC124)**	775304-57-9	free base	CFTR	88.0	0.07	56.5	-1.07	82.4	-0.68	112.9	0.64
**Atazanavir Sulfate**	229975-97-7	sulfate	HIV Protease	76.4	-0.43	94.9	0.32	98.3	-0.01	93.1	0.01
**Atglistatin**	1469924-27-3	free base	ATGL	85.8	-0.03	64.5	-0.78	109.1	0.45	108.7	0.50
**Atomoxetine HCl**	82248-59-7	hydrochloride	5-HT Receptor	85.3	-0.05	62.0	-0.87	117.2	0.79	140.4	1.51
**Atorvastatin Calcium**	134523-03-8	calcium	HMG-CoA Reductase	92.2	0.24	107.5	0.78	95.1	-0.14	111.2	0.58
**Atropine**	5908-99-6	coordination co	AChR	112.5	1.10	94.8	0.32	86.6	-0.51	105.7	0.41
**Aurora A Inhibitor I**	1158838-45-9	free base	Aurora Kinase	50.6	-1.52	46.7	-1.43	62.9	-1.51	59.3	-1.06
**AUY922 (NVP-AUY922)**	747412-49-3	free base	HSP (e.g. HSP90)	40.6	-1.94	36.3	-1.81	34.5	-2.72	47.8	-1.43
**Avagacestat (BMS-708163)**	1146699-66-2	free base	Gamma-secretase,Beta Amyloid	130.5	1.87	91.9	0.21	110.4	0.51	93.9	0.03
**Avanafil**	330784-47-9	free base	PDE	90.2	0.16	53.5	-1.18	104.3	0.25	125.9	1.05
**Avasimibe**	166518-60-1	free base	P450 (e.g. CYP17)	104.1	0.75	69.0	-0.62	94.4	-0.17	81.2	-0.37
**AVL-292**	1202757-89-8	free base	BTK	76.1	-0.43	81.3	-0.17			92.1	-0.02
**Axitinib**	319460-85-0	free base	c-Kit,VEGFR,PDGFR	121.5	1.49	114.8	1.05	80.9	-0.75	96.1	0.10
**AZ 3146**	1124329-14-1	free base	Kinesin	69.2	-0.73	90.1	0.15	139.0	1.72	132.3	1.25
**AZ 628**	878739-06-1	free base	Raf	72.3	-0.60	37.8	-1.75	56.6	-1.78	104.0	0.35
**AZ 960**	905586-69-8	free base	JAK	61.7	-1.05	66.5	-0.71	42.6	-2.37	79.8	-0.41
**AZ20**	1233339-22-4	free base	ATM/ATR	57.6	-1.22	46.2	-1.45	80.3	-0.77	58.4	-1.09
**Azacitidine**	320-67-2	free base	DNA Methyltransferase	117.9	1.33	115.6	1.08	117.0	0.79	80.7	-0.38
**Azatadine dimaleate**	3978-86-7	maleate	Histamine Receptor	96.0	0.40	81.6	-0.16	111.0	0.53	123.3	0.96
**AZD1080**	612487-72-6	free base	GSK-3	77.0	-0.40	67.0	-0.69			104.9	0.38
**AZD1208**	1204144-28-4	free base	Pim	55.7	-1.30	79.5	-0.24			45.3	-1.51
**AZD1480**	935666-88-9	free base	JAK	80.1	-0.27	103.2	0.63	77.2	-0.90	74.1	-0.59
**AZD1981**	802904-66-1	free base	GPR	117.7	1.32	113.7	1.01	102.2	0.16	115.5	0.72
**AZD2014**	1009298-59-2	free base	mTOR	57.7	-1.21	60.5	-0.93	71.9	-1.13	61.8	-0.98

**AZD2461**	1174043-16-3	free base	PARP	78.0	-0.36	72.6	-0.49	82.7	-0.67	120.3	0.87
**AZD2461**	1174043-16-3	free base	PARP	85.8	-0.03	58.4	-1.00	77.3	-0.90	121.2	0.90
**AZD2858**	486424-20-8	free base	GSK-3	74.5	-0.50	104.8	0.68			70.6	-0.70
**AZD3463**	1356962-20-3	free base	ALK	30.8	-2.36	69.0	-0.62			69.6	-0.74
**AZD3514**	1240299-33-5	free base	Androgen Receptor	80.3	-0.26	107.6	0.79	77.7	-0.88	94.7	0.06
**AZD3514**	1240299-33-5	free base	Androgen Receptor	81.4	-0.21	79.2	-0.25	79.8	-0.79	114.9	0.70
**AZD4547**	1035270-39-3	free base	FGFR	87.9	0.06	114.1	1.02	98.4	0.00	117.5	0.78
**AZD5363**	1143532-39-1	free base	Akt	48.2	-1.62	80.8	-0.19	109.7	0.48	128.7	1.14
**AZD5438**	602306-29-6	free base	CDK	69.5	-0.71	51.8	-1.25	99.0	0.02	86.4	-0.20
**AZD6482**	1173900-33-8	free base	PI3K	71.5	-0.63	115.6	1.08	98.1	-0.01	93.7	0.03
**AZD7545**	252017-04-2	free base	PDHK	78.8	-0.32	99.8	0.50	103.6	0.22	109.3	0.52
**AZD7762**	860352-01-8	free base	Chk	46.6	-1.69	13.7	-2.63	112.7	0.60	19.6	-2.32
**AZD8055**	1009298-09-2	free base	mTOR	49.7	-1.56	50.9	-1.28	46.1	-2.22	40.6	-1.65
**AZD8330**	869357-68-6	free base	MEK	100.9	0.61	23.9	-2.26	42.5	-2.38	102.7	0.31
**AZD8931 (Sapitinib)**	848942-61-0	free base	HER2,EGFR	109.5	0.98	60.2	-0.94	75.4	-0.98	79.9	-0.41
**AZD9291**	1421373-65-0	free base	EGFR	90.8	0.19	92.4	0.23	96.9	-0.06	47.9	-1.42
**Azelastine HCl**	79307-93-0	hydrochloride	Histamine Receptor	110.0	1.00	111.0	0.91	124.1	1.09	131.4	1.22
**Azelnidipine**	123524-52-7	free base	Calcium Channel	75.7	-0.45	73.9	-0.44	109.3	0.46	120.5	0.88
**Azilsartan**	147403-03-0	free base	RAAS	90.8	0.19	55.9	-1.09	116.4	0.76	112.9	0.64
**Azilsartan Medoxomil**	863031-21-4	free base	RAAS	92.6	0.26	119.9	1.23	109.9	0.49	124.7	1.01
**Azithromycin**	83905-01-5	free base	Autophagy	83.5	-0.12	108.8	0.83	114.2	0.67	81.8	-0.35
**Baicalein**	491-67-8	free base	P450 (e.g. CYP17)	84.5	-0.08	60.3	-0.94	89.2	-0.39	117.5	0.78
**Bambuterol HCl**	81732-46-9	Salt	Adrenergic Receptor	87.7	0.05	61.1	-0.91	82.3	-0.69	127.7	1.11
**BAPTA-AM**	126150-97-8	free base	Others	68.3	-0.77	70.1	-0.58	94.8	-0.16	95.6	0.09
**Barasertib (AZD1152-HQPA)**	722544-51-6	free base	Aurora Kinase	90.6	0.18	82.0	-0.14	74.4	-1.02	62.1	-0.97
**Bardoxolone Methyl**	218600-53-4	free base	IκB/IKK	45.1	-1.75	31.2	-1.99	78.1	-0.86	57.1	-1.13
**Baricitinib (LY3009104, INCB028050)**	1187594-09-7	free base	JAK	71.6	-0.63	94.9	0.33	125.6	1.15	102.8	0.32
**Batimastat (BB-94)**	130370-60-4	free base	MMP	71.7	-0.62	67.9	-0.66			106.4	0.43
**BAY 11-7082**	19542-67-7	free base	IκB/IKK,E2 conjugating	76.0	-0.44	57.1	-1.05	117.6	0.81	94.0	0.04

**Bazedoxifene HCl**	198480-56-7	free base	Estrogen/progestogen Receptor	119.9	1.42	99.9	0.50	102.9	0.19	80.0	-0.40
**Belinostat (PXD101)**	414864-00-9	free base	HDAC	35.8	-2.14	67.9	-0.66	63.3	-1.49	2.1	-2.87
**Benazepril HCl**	86541-74-4	hydrochloride	RAAS	88.1	0.07	82.2	-0.14	109.8	0.48	91.3	-0.05
**Benidipine HCl**	91599-74-5	hydrochloride	Calcium Channel	86.6	0.01	91.9	0.22	87.6	-0.46	97.6	0.15
**Benserazide HCl**	14919-77-8	hydrochloride	Dopamine Receptor,Others	113.2	1.14	66.7	-0.70	127.8	1.25	107.6	0.47
**Benztropine mesylate**	132-17-2	mesylate	Histamine Receptor	97.4	0.47	117.4	1.14	110.9	0.53	124.3	1.00
**Bepotastine Besilate**	190786-44-8	besilate	Histamine Receptor	79.9	-0.27	78.5	-0.27	107.8	0.40	120.9	0.89
**Betahistine 2HCl**	5579-84-0	2HCl	Histamine Receptor	91.9	0.23	117.5	1.14	116.5	0.77	145.1	1.66
**Beta-Lapachone**	4707-32-8	free base	Topoisomerase	91.4	0.21	103.0	0.62			86.3	-0.21
**Betaxolol**	659-18-7	free base	Adrenergic Receptor	99.4	0.55	115.9	1.09	95.2	-0.14	149.9	1.81
**Bethanechol chloride**	590-63-6	chloride	AChR	106.0	0.83	112.3	0.96	119.0	0.87	117.1	0.77
**Betulinic acid**	472-15-1	free base	Topoisomerase	95.2	0.37	116.2	1.10	114.4	0.68	95.0	0.07
**BGT226 (NVP-BGT226)**	1245537-68-1	free base	PI3K,mTOR	16.1	-2.98	14.1	-2.61	36.0	-2.65	-1.1	-2.97
**BI 2536**	755038-02-9	free base	PLK	40.8	-1.93	39.2	-1.70	43.0	-2.36	4.4	-2.80
**BIBR 1532**	321674-73-1	free base	Telomerase	102.3	0.67	116.5	1.11	121.2	0.97	62.0	-0.98
**Bicalutamide**	90357-06-5	free base	Androgen Receptor	105.7	0.82	107.2	0.77	135.0	1.55	75.0	-0.57
**BI-D1870**	501437-28-1	free base	S6 Kinase	83.2	-0.13	82.3	-0.14	105.2	0.29	73.4	-0.61
**BIIB021**	848695-25-0	free base	HSP (e.g. HSP90)	52.1	-1.45	38.7	-1.72	66.7	-1.35	27.4	-2.07
**BIO**	667463-62-9	free base	GSK-3	61.8	-1.04	91.4	0.19			132.2	1.25
**Biperiden HCl**	1235-82-1	hydrochloride	AChR	106.7	0.86	115.0	1.05	119.5	0.90	95.3	0.08
**BIRB 796 (Doramapimod)**	285983-48-4	free base	p38 MAPK	96.0	0.41	115.6	1.08	87.9	-0.45	65.2	-0.88
**Birinapant**	1260251-31-7	free base	IAP	85.4	-0.04	92.5	0.24	82.3	-0.68	127.3	1.09
**Bisoprolol fumarate**	104344-23-2	free base	Adrenergic Receptor	106.6	0.85	85.8	-0.01	112.7	0.61	100.8	0.25
**BIX 01294**	1392399-03-9	hydrochloride	Histone Methyltransferase	71.2	-0.65	62.8	-0.84	103.0	0.19	100.3	0.24
**BIX 02188**	1094614-84-2	free base	MEK	84.0	-0.10	115.8	1.08	114.4	0.68	95.2	0.08
**BIX 02189**	1094614-85-3	free base	MEK	84.5	-0.08	116.2	1.10	111.1	0.54	96.3	0.11
**BKM120 (NVP-BKM120, Buparlisib)**	944396-07-0	free base	PI3K	75.8	-0.45	98.2	0.44	76.4	-0.94	63.3	-0.93
**BML-190**	2854-32-2	free base	Cannabinoid Receptor	91.3	0.21	93.1	0.26	110.2	0.50	114.1	0.68

**BMN 673**	1207456-01-6	free base	PARP	60.2	-1.11	106.1	0.73	86.5	-0.51	33.8	-1.87
**BMS-265246**	582315-72-8	free base	CDK	81.4	-0.21	110.6	0.90	86.5	-0.51	87.1	-0.18
**BMS-345541**	445430-58-0	free base	IκB/IKK	88.7	0.10	77.7	-0.30	109.4	0.47	124.7	1.01
**BMS-378806**	357263-13-9	free base	gp120/CD4	98.0	0.49	111.9	0.94	108.8	0.44	123.7	0.98
**BMS-536924**	468740-43-4	free base	IGF-1R	33.2	-2.25	80.2	-0.21	53.2	-1.92	110.3	0.56
**BMS-707035**	729607-74-3	free base	Integrase	51.9	-1.46	46.2	-1.45	63.4	-1.49	29.1	-2.02
**BMS-754807**	1001350-96-4	free base	IGF-1R,Trk receptor,c-Met	28.2	-2.47	49.9	-1.31	45.8	-2.24	55.0	-1.20
**BMS-777607**	1025720-94-8	free base	Axl,c-Met	104.9	0.79	115.3	1.06	112.9	0.61	78.5	-0.45
**BMS-794833**	1174046-72-0	free base	VEGFR,c-Met	100.8	0.61	50.5	-1.29	138.8	1.71	98.9	0.19
**BMS-833923**	1059734-66-5	free base	Hedgehog/Smoothened	74.8	-0.49	93.8	0.28			117.3	0.78
**BMY 7378**	21102-95-4	dihydrochloride	5-HT Receptor	69.5	-0.72	91.2	0.19	137.7	1.67	91.8	-0.03
**Bortezomib (PS-341)**	179324-69-7	free base	Proteasome	5.2	-3.44	2.4	-3.04	27.5	-3.01	3.6	-2.83
**Bosentan**	147536-97-8	free base	Endothelin Receptor	84.3	-0.09	71.7	-0.52	78.6	-0.84	129.2	1.15
**Bosentan Hydrate**	157212-55-0	hydrate	Endothelin Receptor	98.0	0.49	78.5	-0.27	112.7	0.60	128.8	1.14
**Bosutinib (SKI-606)**	380843-75-4	free base	Src	80.9	-0.23	94.6	0.31	142.3	1.86	127.3	1.09
**Brefeldin A**	20350-15-6	free base	ATPase,Autophagy	1.9	-3.58	1.7	-3.06	23.2	-3.20	2.1	-2.87
**Brinzolamide**	138890-62-7	free base	Carbonic Anhydrase	89.9	0.15	63.6	-0.81	109.7	0.48	142.7	1.58
**Brivanib (BMS-540215)**	649735-46-6	free base	FGFR,VEGFR	94.4	0.34	76.5	-0.34	78.5	-0.85	116.5	0.75
**Brivanib Alaninate (BMS-582664)**	649735-63-7	alaninate	VEGFR,FGFR	97.0	0.45	88.2	0.08	92.7	-0.25	74.5	-0.58
**BRL-15572**	193611-72-2	dihydrochloride	5-HT Receptor	120.0	1.42	73.0	-0.47	123.6	1.07	113.9	0.67
**BRL-54443**	57477-39-1	free base	5-HT Receptor	102.2	0.67	93.3	0.27	127.7	1.24	84.6	-0.26
**Bromosporine**	N/A	free base	Epigenetic Reader Domain	49.5	-1.56	69.9	-0.59			62.4	-0.96
**Brompheniramine hydrogen maleate**	980-71-2	maleate	Histamine Receptor	81.8	-0.20	56.1	-1.09	123.2	1.05	91.0	-0.06
**BS-181 HCl**	1397219-81-6	hydrochloride	CDK	99.9	0.57	47.1	-1.42	106.1	0.33	93.2	0.01
**BTB06584**	219793-45-0	free base	Fo-ATPase	131.0	1.89	109.3	0.85	98.8	0.01	130.4	1.19
**Bufexamac**	2438-72-4	free base	COX	96.0	0.41	88.1	0.08	111.0	0.53	131.1	1.21
**Bupivacaine HCl**	18010-40-7	hydrochloride	cAMP	118.6	1.36	82.2	-0.14	120.6	0.94	113.5	0.65
**Butein**	487-52-5	free base	EGFR	78.9	-0.32	75.3	-0.39	85.3	-0.56	139.8	1.49

**BV-6**	1001600-56-1	free base	IAP	62.1	-1.03	83.0	-0.11	105.3	0.29	113.0	0.64
**BX-795**	702675-74-9	free base	IκB/IKK,PDK-1	69.6	-0.71	68.6	-0.63	117.0	0.79	65.8	-0.86
**BX-912**	702674-56-4	free base	PDK-1	93.0	0.28	84.5	-0.06	93.2	-0.22	54.0	-1.23
**BYL719**	1217486-61-7	free base	PI3K	54.1	-1.37	111.9	0.94	85.0	-0.57	107.0	0.45
**C646**	328968-36-1	free base	Histone Acetyltransferase	75.3	-0.47	90.8	0.17			125.5	1.04
**Cabozantinib (XL184, BMS-907351)**	849217-68-1	free base	FLT3,Tie-2,c-Kit,c-Met,VEGFR,Axl	123.9	1.59	109.6	0.86	132.4	1.44	89.1	-0.12
**Cabozantinib malate (XL184)**	1140909-48-3	Malate	VEGFR	74.8	-0.49	118.5	1.18	104.0	0.23	118.6	0.82
**Caffeic Acid Phenethyl Ester**	104594-70-9	free base	NF-κB	82.2	-0.18	116.4	1.11	102.0	0.15	55.3	-1.19
**CAL-101 (Idelalisib, GS-1101)**	870281-82-6	free base	PI3K	82.1	-0.18	75.2	-0.39	74.7	-1.01	112.4	0.62
**Camptothecin**	7689-03-4	free base	Topoisomerase	30.7	-2.36	14.5	-2.60	96.3	-0.09	3.4	-2.83
**Canagliflozin**	842133-18-0	free base	SGLT	88.8	0.10	51.4	-1.26	110.2	0.50	123.4	0.97
**Candesartan**	139481-59-7	free base	RAAS	90.9	0.19	114.7	1.04	111.0	0.53	90.2	-0.08
**Candesartan Cilexetil**	145040-37-5	free base	RAAS	121.6	1.49	93.2	0.26	111.4	0.55	111.8	0.60
**Captopril**	62571-86-2	free base	RAAS	118.3	1.35	114.4	1.03	110.2	0.50	122.2	0.93
**Carbamazepine**	298-46-4	free base	Autophagy,Sodium Channel	117.8	1.33	113.9	1.01	104.2	0.25	93.1	0.01
**Carfilzomib (PR-171)**	868540-17-4	free base	Proteasome	22.9	-2.69	1.7	-3.06	23.2	-3.20	2.2	-2.87
**Carteolol HCl**	51781-21-6	Salt	Adrenergic Receptor	75.0	-0.48	113.1	0.99	81.7	-0.71	93.0	0.00
**Carvedilol**	72956-09-3	free base	Adrenergic Receptor	129.3	1.82	112.9	0.98	109.1	0.45	105.3	0.40
**CAY10505**	1218777-13-9	free base	PI3K	76.2	-0.43	57.6	-1.03	110.6	0.51	123.2	0.96
**CCT128930**	885499-61-6	free base	Akt	87.3	0.04	64.5	-0.78	94.3	-0.18	89.9	-0.09
**CCT129202**	942947-93-5	free base	Aurora Kinase	57.2	-1.24	57.1	-1.05	86.4	-0.51	59.3	-1.06
**CCT137690**	1095382-05-0	free base	Aurora Kinase	64.5	-0.93	57.3	-1.04	68.0	-1.29	97.1	0.14
**Cediranib (AZD2171)**	288383-20-0	free base	VEGFR	93.5	0.30	115.8	1.08	103.2	0.20	109.2	0.52
**Celecoxib**	169590-42-5	free base	COX	106.7	0.86	79.4	-0.24	103.3	0.21	83.1	-0.31
**CEP-18770 (Delanzomib)**	847499-27-8	free base	Proteasome	3.5	-3.51	3.4	-3.00	33.6	-2.75	1.3	-2.90
**CEP-32496**	1188910-76-0	free base	CSF-1R,Raf	81.7	-0.20	57.8	-1.02	85.4	-0.56	67.8	-0.79
**CEP-33779**	1257704-57-6	free base	JAK	83.9	-0.11	64.0	-0.80	119.6	0.90	116.4	0.75
**Cetirizine DiHCl**	83881-52-1	hydrochloride	Histamine Receptor	91.2	0.20	54.9	-1.13	153.2	2.33	95.7	0.09

**CFTRinh-172**	307510-92-5	free base	CFTR	78.5	-0.33	50.4	-1.29			91.6	-0.04
**CGI1746**	910232-84-7	free base	BTK	77.1	-0.39	82.6	-0.12	107.5	0.38	107.6	0.47
**CGK 733**	905973-89-9	free base	ATM/ATR	79.7	-0.28	55.4	-1.11			114.9	0.70
**CGP 57380**	522629-08-9	free base	MNK	77.1	-0.40	116.0	1.09	119.6	0.90	103.6	0.34
**CGS 21680 HCl**	124431-80-7	hydrochloride	5-alpha Reductase	117.80	1.33	60.4	-0.93	92.5	-0.26	120.7	0.88
**CH5132799**	1007207-67-1	free base	mTOR,PI3K	58.6	-1.18	50.3	-1.30	102.4	0.17	126.7	1.07
**CH5138303**	959763-06-5	free base	HSP (e.g. HSP90)	42.2	-1.87	33.9	-1.89	48.0	-2.15	55.9	-1.17
**CHIR-124**	405168-58-3	free base	Chk	53.0	-1.42	30.2	-2.03	144.9	1.97	53.2	-1.25
**CHIR-99021 (CT99021) HCl**	252917-06-9	hydrochloride	GSK-3	84.2	-0.10	50.3	-1.30	116.0	0.75	119.7	0.85
**Chloroambucil**	305-03-3	free base	DNA/RNA Synthesis	85.8	-0.03	118.1	1.17	100.8	0.10	102.7	0.31
**Chlorpheniramine Maleate**	113-92-8	maleate	Histamine Receptor	105.1	0.79	117.5	1.14	112.5	0.60	94.2	0.04
**Chlorpromazine HCl**	69-09-0	hydrochloride	Potassium Channel,Dopamine Receptor	105.2	0.80	79.8	-0.23	117.9	0.83	90.5	-0.07
**Chlorprothixene**	113-59-7	free base	Dopamine Receptor	105.9	0.83	112.8	0.97	105.5	0.30	108.2	0.49
**Chrysophanic Acid**	481-74-3	free base	mTOR,EGFR	137.7	2.17	110.4	0.89	86.0	-0.53	124.8	1.01
**CI994 (Tacedinaline)**	112522-64-2	free base	HDAC	83.1	-0.14	111.9	0.94	145.7	2.01	136.8	1.39
**Ciclopirox ethanolamine**	41621-49-2	ethanolamine	ATPase	117.0	1.30	111.8	0.94	111.2	0.54	102.3	0.30
**Cilazapril Monohydrate**	92077-78-6	monohydrate	RAAS	95.3	0.38	115.8	1.08	82.2	-0.69	93.1	0.01
**Cilengitide**	188968-51-6	free base	Integrin	85.7	-0.03	70.4	-0.57	60.7	-1.60	103.8	0.35
**Cilnidipine**	132203-70-4	free base	Calcium Channel	114.2	1.18	76.6	-0.34	118.8	0.87	69.4	-0.74
**Cilomilast**	153259-65-5	free base	PDE	91.7	0.22	66.3	-0.72	132.1	1.43	67.3	-0.81
**Cilostazol**	73963-72-1	free base	PDE	74.2	-0.52	60.4	-0.93	117.4	0.80	93.2	0.01
**Cimetidine**	51481-61-9	free base	Histamine Receptor	94.4	0.34	107.3	0.77	123.3	1.06	121.0	0.89
**Cinacalcet HCl**	364782-34-3	hydrochloride	CaSR	102.0	0.66	77.5	-0.31	108.7	0.43	85.6	-0.23
**Ciprofibrate**	52214-84-3	free base	PPAR	125.4	1.65	90.1	0.15	112.5	0.60	111.7	0.60
**Ciproxifan**	184025-18-1	maleate	Histamine Receptor	91.6	0.22	46.8	-1.43	128.1	1.26	109.5	0.53
**Cisatracurium Besylate**	96946-42-8	besylate	Adrenergic Receptor	124.9	1.63	106.8	0.76	83.7	-0.63	108.1	0.48
**CK-636**	442632-72-6	free base	Arp2/3	98.1	0.49	116.9	1.12	100.8	0.10	83.5	-0.30
**Clarithromycin**	81103-11-9	free base	P450 (e.g. CYP17)	101.7	0.65	55.7	-1.10	115.2	0.71	123.6	0.98

**Clemastine Fumarate**	14976-57-9	free base	Histamine Receptor	106.5	0.85	117.1	1.13	110.4	0.51	97.9	0.16
**Clevidipine Butyrate**	167221-71-8	free base	Calcium Channel	98.6	0.52	112.7	0.97	101.1	0.11	101.5	0.27
**Clinofibrate**	30299-08-2	free base	RAAS	100.8	0.61	113.9	1.01	126.6	1.20	110.3	0.56
**Clomifene citrate**	50-41-9	citrate	Estrogen/progestogen Receptor	75.6	-0.46	51.1	-1.27	137.2	1.65	128.3	1.12
**Clomipramine HCl**	17321-77-6	hydrochloride	5-HT Receptor	95.8	0.40	49.6	-1.32	109.6	0.47	123.3	0.96
**Clonidine HCl**	4205-91-8	hydrochloride	Adrenergic Receptor,Autophagy	109.7	0.99	109.0	0.83	121.3	0.97	115.8	0.73
**Clopidogrel**	120202-66-6	sulfate	P2 Receptor	98.9	0.53	60.2	-0.94	150.6	2.21	77.5	-0.48
**Clozapine**	5786-21-0	free base	5-HT Receptor	108.6	0.94	66.8	-0.70	123.4	1.06	96.4	0.11
**CNX-2006**	1375465-09-0	free base	EGFR	72.9	-0.57	76.0	-0.37			120.9	0.89
**CNX-774**	1202759-32-7	free base	BTK	85.1	-0.05	85.1	-0.03			108.5	0.50
**CO-1686 (AVL-301)**	1374640-70-6	free base	EGFR	64.0	-0.95	116.4	1.10	96.5	-0.08	76.4	-0.52
**Cobicistat (GS-9350)**	1004316-88-4	free base	P450 (e.g. CYP17)	77.4	-0.38	115.6	1.08	121.4	0.97	96.0	0.10
**CORM-3**	475473-26-8	free base	Others	78.0	-0.36	71.6	-0.52	81.8	-0.71	80.8	-0.38
**Costunolide**	553-21-9	free base	Telomerase	75.1	-0.48	115.0	1.05	128.1	1.26	106.6	0.44
**CP-673451**	343787-29-1	free base	PDGFR	96.2	0.41	115.2	1.06	162.6	2.72	100.7	0.25
**CP-724714**	537705-08-1	free base	EGFR,HER2	97.6	0.48	106.1	0.73	116.6	0.77	87.0	-0.18
**CP-91149**	186392-40-5	free base	Phosphorylase	101.9	0.66	81.9	-0.15	124.4	1.10	125.1	1.02
**CPI-203**	1446144-04-2	free base	Epigenetic Reader Domain	26.6	-2.53	48.8	-1.35	61.2	-1.58	5.5	-2.77
**CPI-613**	95809-78-2	free base	Dehydrogenase	105.5	0.81	65.8	-0.73	115.6	0.73	112.3	0.62
**Crenolanib (CP-868596)**	670220-88-9	free base	PDGFR	66.1	-0.86	48.4	-1.37	163.6	2.77	137.3	1.41
**Crizotinib (PF-02341066)**	877399-52-5	free base	c-Met,ALK	58.4	-1.19	95.6	0.35	102.7	0.18	84.3	-0.27
**CRT0044876**	6960-45-8	free base	APE	81.8	-0.20	104.0	0.66	78.0	-0.87	69.2	-0.75
**Cryptotanshinone**	35825-57-1	free base	STAT	106.1	0.83	49.5	-1.33	97.5	-0.04	91.1	-0.05
**CTEP (RO4956371)**	871362-31-1	free base	GluR	96.8	0.44	114.7	1.04	114.5	0.68	116.7	0.76
**CUDC-101**	1012054-59-9	free base	HDAC,HER2,EGFR	7.9	-3.33	48.6	-1.36	36.2	-2.64	0.3	-2.93
**CUDC-907**	1339928-25-4	free base	PI3K,HDAC	73.1	-0.57	114.9	1.05	104.6	0.26	94.7	0.06
**CW069**		free base	Microtubule Associat	76.9	-0.40	116.1	1.10	95.5	-0.12	75.2	-0.56
**CX-6258 HCl**	1353859-00-3	Salt	Pim	58.2	-1.20	75.4	-0.39	66.6	-1.35	11.4	-2.58

**CX-6258 HCl**	1353859-00-3	Salt	Pim	64.7	-0.92	106.5	0.75	68.6	-1.27	26.4	-2.11
**CYC116**	693228-63-6	free base	Aurora Kinase,VEGFR	98.4	0.51	81.1	-0.18	92.2	-0.27	86.0	-0.22
**Cyclizine 2HCl**	5897-18-7	hydrochloride	Histamine Receptor	132.2	1.94	118.5	1.18	95.6	-0.12	124.5	1.00
**Cyclobenzaprine HCl**	6202-23-9	Salt	Others	77.0	-0.40	117.4	1.14	81.2	-0.73	101.8	0.28
**Cyproheptadine HCl**	969-33-5	hydrochloride	Histamine Receptor	100.1	0.58	110.7	0.90	97.3	-0.05	122.7	0.95
**Cyproterone Acetate**	427-51-0	acetate	Androgen Receptor	103.4	0.72	115.8	1.08	106.2	0.33	94.6	0.06
**CYT387**	1056634-68-4	free base	JAK	90.3	0.17	57.8	-1.03	96.8	-0.07	102.3	0.30
**CYT997 (Lexibulin)**	917111-44-5	free base	Microtubule Associat	55.5	-1.31	52.5	-1.22	43.3	-2.34	27.7	-2.06
**CZC24832**	1159824-67-5	free base	PI3K	84.4	-0.08	106.1	0.73	82.1	-0.69	100.5	0.24
**Dabrafenib (GSK2118436)**	1195765-45-7	free base	Raf	96.4	0.42	113.9	1.01	197.9	4.22	128.4	1.13
**Daclatasvir (BMS-790052)**	1009119-64-5	free base	HCV Protease	149.9	2.69	106.5	0.75	116.7	0.78	60.9	-1.01
**Dacomitinib (PF299804, PF299)**	1110813-31-4	free base	EGFR	113.4	1.14	114.1	1.02	101.9	0.14	87.5	-0.17
**Dalcetrapib (JTT-705, RO4607381)**	211513-37-0	free base	CETP	69.3	-0.72	115.3	1.07	121.7	0.99	139.4	1.48
**Danoprevir (ITMN-191)**	850876-88-9	free base	HCV Protease	120.2	1.43	114.2	1.02	104.6	0.26	107.3	0.46
**Danusertib (PHA-739358)**	827318-97-8	free base	c-RET,FGFR,Bcr-Abl,Aurora Kinase			71.1	-0.54	41.3	-2.43	61.0	-1.01
**Dapagliflozin**	461432-26-8	free base	SGLT	117.4	1.31	91.9	0.21	109.6	0.47	93.2	0.01
**Dapivirine (TMC120)**	244767-67-7	free base	Reverse Transcriptase	82.9	-0.15	57.1	-1.05	127.4	1.23	121.5	0.91
**Dapoxetine HCl**	129938-20-1	hydrochloride	5-HT Receptor	100.2	0.59	60.2	-0.94	106.2	0.33	133.9	1.30
**DAPT (GSI-IX)**	208255-80-5	free base	Gamma-secretase,Beta Amyloid	110.0	1.00	79.4	-0.24	81.5	-0.72	116.7	0.76
**Darapladib (SB-480848)**	356057-34-6	free base	Phospholipase (e.g.	86.3	0.00	65.3	-0.75	100.4	0.08	124.4	1.00
**Darifenacin HBr**	133099-07-7	HBr	AChR	93.2	0.29	53.4	-1.18	114.1	0.66	113.8	0.66
**Darunavir Ethanolate**	635728-49-3	ethanolate	HIV Protease	88.0	0.07	114.7	1.05	94.4	-0.17	97.1	0.14
**Dasatinib**	302962-49-8	free base	Bcr-Abl,c-Kit,Src	110.5	1.02	117.1	1.13	115.3	0.72	93.6	0.02
**Daunorubicin HCl**	23541-50-6	hydrochloride	Telomerase	25.9	-2.57	65.0	-0.76	117.3	0.80	12.1	-2.56
**DBeQ**	177355-84-9	free base	p97	77.2	-0.39	109.0	0.83			77.4	-0.49
**DCC-2036 (Rebastinib)**	1020172-07-9	free base	Bcr-Abl	69.1	-0.73	62.7	-0.85	83.4	-0.64	124.1	0.99
**Decamethonium Bromide**	541-22-0	bromide	AChR	93.5	0.30	116.6	1.11	112.4	0.59	89.1	-0.12
**Decitabine**	2353-33-5	free base	DNA Methyltransferase	90.2	0.16	102.1	0.58	104.4	0.25	58.6	-1.08

**Degrasyn (WP1130)**	856243-80-6	free base	DUB,Bcr-Abl	102.3	0.67	93.0	0.26	92.6	-0.25	77.6	-0.48
**Dehydroepiandrosterone (DHEA)**	53-43-0	free base	Androgen Receptor	109.2	0.97	102.7	0.61	125.8	1.16	127.8	1.11
**Deltarasin**	1440898-61-2	free base	PDE	85.4	-0.04	92.8	0.25			86.9	-0.19
**Demeclocycline HCl**	64-73-3	Salt	Others	67.8	-0.79	117.3	1.14	77.0	-0.91	107.4	0.46
**Desloratadine**	100643-71-8	free base	Histamine Receptor	115.8	1.24	116.0	1.09	109.3	0.46	95.0	0.07
**Desvenlafaxine**	93413-62-8	free base	5-HT Receptor	97.9	0.49	67.2	-0.68	90.4	-0.34	126.3	1.06
**Desvenlafaxine Succinate**	386750-22-7	succinate	5-HT Receptor	155.8	2.94	117.8	1.16	100.2	0.07	122.2	0.93
**Detomidine HCl**	90038-01-0	hydrochloride	Adrenergic Receptor	121.2	1.47	114.8	1.05	94.5	-0.17	125.9	1.05
**Dexamethasone (DHAP)**	50-02-2	free base	Autophagy,IL Receptor	81.8	-0.20	115.5	1.07	160.5	2.64	68.3	-0.78
**Dexmedetomidine**	113775-47-6	free base	Adrenergic Receptor	89.9	0.15	118.1	1.17	115.9	0.74	124.9	1.02
**Dibucaine HCl**	61-12-1	free base	Sodium Channel	76.2	-0.43	118.0	1.16	101.0	0.11	137.9	1.43
**Diclofenac Sodium**	15307-79-6	sodium	COX	93.1	0.28	117.5	1.14	104.6	0.26	105.3	0.39
**Dicoumarol**	66-76-2	free base	Others	74.6	-0.50	79.7	-0.23	77.6	-0.89	128.0	1.12
**Didanosine**	69655-05-6	free base	Reverse Transcriptase	95.4	0.38	114.6	1.04	79.4	-0.81	98.6	0.18
**Dienogest**	65928-58-7	free base	Estrogen/progestogen Receptor	93.0	0.28	86.3	0.01	108.3	0.42	92.4	-0.01
**Digoxin**	20830-75-5	free base	Sodium Channel	40.0	-1.97	15.9	-2.55	22.6	-3.22	16.0	-2.43
**Dinaciclib (SCH727965)**	779353-01-4	free base	CDK	51.5	-1.48	24.7	-2.23	26.3	-3.07	14.6	-2.48
**Diphemanil Methylsulfate**	62-97-5	methylsulfate	AChR	92.6	0.26	50.3	-1.30	102.1	0.16	123.8	0.98
**Diphenidol HCl**	3254-89-5	Salt	AChR	88.3	0.08	68.3	-0.64	90.1	-0.36	106.2	0.42
**Divalproex Sodium**	76584-70-8	sodium	Autophagy	98.9	0.53	110.1	0.88	102.9	0.19	93.1	0.01
**DMH1**	1206711-16-1	free base	BMP	85.5	-0.04	69.4	-0.61			135.1	1.34
**DMXAA (Vadimezan)**	117570-53-3	free base	VDA	96.5	0.43	115.0	1.05	104.9	0.27	81.6	-0.35
**Dolutegravir (GSK1349572)**	1051375-16-6	free base	Integrase	75.6	-0.46	112.7	0.97	124.0	1.08	121.6	0.91
**Domperidone**	57808-66-9	free base	Dopamine Receptor	113.9	1.16	116.4	1.11	123.8	1.08	116.1	0.74
**Dopamine HCl**	62-31-7	hydrochloride	Dopamine Receptor	100.8	0.61	59.3	-0.97	118.0	0.83	124.2	0.99
**Dovitinib (TKI-258) Dilactic Acid**	852433-84-2	dilactic Acid	PDGFR,FGFR,c-Kit,FLT3,VEGFR	52.4	-1.44	57.8	-1.02	95.1	-0.14	17.9	-2.37
**Dovitinib (TKI-258, CHIR-258)**	405169-16-6	citrate	FGFR,FLT3,c-Kit,VEGFR,PDGFR	66.7	-0.84	83.4	-0.10	87.0	-0.49	110.8	0.57
**Doxazosin Mesylate**	77883-43-3	mesylate	Adrenergic Receptor	78.9	-0.32	53.0	-1.20	115.1	0.71	105.9	0.41

**Doxorubicin (Adriamycin)**	25316-40-9	hydrochloride	Autophagy,Topoisomerase	10.7	-3.21	44.5	-1.51	71.7	-1.14	4.4	-2.80
**Doxycycline HCl**	10592-13-9	hydrochloride	MMP,Others	90.9	0.19	107.9	0.80	83.3	-0.64	120.5	0.88
**Drospirenone**	67392-87-4	free base	Estrogen/progestogen Receptor	103.4	0.72	115.5	1.07	100.0	0.07	85.4	-0.24
**Droxinostat**	99873-43-5	free base	HDAC	103.9	0.74	114.0	1.02	110.1	0.49	98.0	0.16
**Duloxetine HCl**	136434-34-9	hydrochloride	5-HT Receptor	103.5	0.72	112.3	0.96	95.2	-0.14	97.0	0.13
**Dutasteride**	164656-23-9	free base	5-alpha Reductase	97.6	0.47	89.3	0.12	107.9	0.40	95.8	0.10
**Dynasore**	304448-55-3	free base	Dynamin	73.6	-0.54	65.1	-0.76	76.7	-0.92	88.1	-0.15
**Dyphylline**	479-18-5	free base	PDE	98.8	0.53	116.6	1.11	111.6	0.56	79.3	-0.43
**E-64**	66701-25-5	free base	Cathepsin K	85.2	-0.05	65.2	-0.75	100.8	0.10	131.3	1.22
**Edoxaban**	480449-70-5	free base	Factor Xa	154.8	2.90	107.2	0.77	107.8	0.40	94.1	0.04
**EHop-016**	1380432-32-5	free base	Rac	100.0	0.57	115.1	1.06	104.6	0.26	84.4	-0.27
**EHT 1864**	754240-09-0	Salt	Rho	84.1	-0.10	95.6	0.35	107.4	0.38	67.4	-0.81
**Elesclomol (STA-4783)**	488832-69-5	free base	HSP (e.g. HSP90)	96.8	0.44	117.1	1.13	89.3	-0.39	71.0	-0.69
**Eletriptan HBr**	177834-92-3	HBr	5-HT Receptor	92.6	0.26	117.8	1.16	114.7	0.69	101.3	0.27
**Elvitegravir (GS-9137, JTK-303)**	697761-98-1	free base	Integrase	94.5	0.34	116.3	1.10	106.1	0.32	105.3	0.39
**Embelin**	550-24-3	free base	IAP	100.4	0.59	116.5	1.11	89.4	-0.39	129.0	1.15
**Empagliflozin (BI 10773)**	864070-44-0	free base	SGLT	91.9	0.23	70.7	-0.56	108.0	0.40	96.7	0.12
**Emtricitabine**	143491-57-0	free base	Reverse Transcriptase	97.0	0.45	114.5	1.03	87.3	-0.47	91.7	-0.03
**Enalapril Maleate**	76095-16-4	maleate	RAAS	93.6	0.30	113.1	0.98	103.5	0.22	92.2	-0.02
**Enalaprilat Dihydrate**	84680-54-6	dihydrate	RAAS	85.0	-0.06	115.0	1.05	87.3	-0.47	93.5	0.02
**ENMD-2076**	934353-76-1	tartaric acid	Aurora Kinase,FLT3,VEGFR	62.2	-1.02	100.2	0.52	71.5	-1.14	81.1	-0.37
**Entacapone**	130929-57-6	free base	Histone Methyltransferase	94.8	0.36	84.9	-0.04	91.7	-0.29	123.3	0.96
**Entinostat (MS-275)**	209783-80-2	free base	HDAC	50.1	-1.54	94.5	0.31	106.0	0.32	2.1	-2.87
**Enzalutamide (MDV3100)**	915087-33-1	free base	Androgen Receptor	98.7	0.52	93.0	0.26	113.5	0.64	65.0	-0.88
**Enzastaurin (LY317615)**	170364-57-5	free base	PKC	71.3	-0.64	74.7	-0.41	72.3	-1.11	119.5	0.85
**Epiandrosterone**	481-29-8	free base	Androgen Receptor,Estrogen Receptor	85.1	-0.06	113.6	1.00	106.3	0.33	113.2	0.64
**Epinephrine Bitartrate**	51-42-3	bitartrate	Adrenergic Receptor	88.5	0.09	106.4	0.74	127.0	1.21	99.4	0.21
**Epinephrine HCl**	55-31-2	hydrochloride	Adrenergic Receptor	84.7	-0.07	58.8	-0.99	115.4	0.72	111.6	0.60

**Epirubicin HCl**	56390-09-1	hydrochloride	Topoisomerase	15.6	-3.00	53.5	-1.18	67.3	-1.32	3.5	-2.83
**EPZ004777**	1338466-77-5	free base	Histone Methyltransferase	79.6	-0.29	67.9	-0.66	110.6	0.52	69.6	-0.73
**EPZ5676**	1380288-87-8	free base	Histone Methyltransferase	103.6	0.73	73.8	-0.44	99.4	0.04	88.4	-0.14
**EPZ-6438**	1403254-99-8	free base	Histone Methyltransferase	67.6	-0.80	113.8	1.01			82.2	-0.34
**Equol**	531-95-3	free base	Estrogen/progestogen Receptor	117.9	1.33	93.7	0.28	97.8	-0.03	110.4	0.56
**Erastin**	571203-78-6	free base	Ferroptosis	159.8	3.11	90.0	0.15			93.3	0.02
**Erlotinib HCl (OSI-744)**	183319-69-9	hydrochloride	Autophagy,EGFR	86.6	0.01	68.4	-0.64	92.8	-0.24	114.0	0.67
**Escitalopram Oxalate**	219861-08-2	oxalate	5-HT Receptor	80.2	-0.26	117.2	1.13	106.7	0.35	128.9	1.14
**ESI-09**	263707-16-0	free base	Others	107.3	0.88	92.8	0.25	105.7	0.31	83.2	-0.31
**Esomeprazole Sodium**	161796-78-7	free base	ATPase	108.8	0.95	115.1	1.06	88.1	-0.44	118.9	0.83
**Estradiol Benzoate**	50-50-0	free base	Others	132.1	1.93	116.2	1.10	106.6	0.35	42.1	-1.61
**Estradiol Cypionate**	313-06-4	free base	Estrogen/progestogen	88.4	0.08	117.5	1.14	107.7	0.39	158.2	2.07
**Estradiol valerate**	979-32-8	free base	Estrogen/progestogen Receptor	96.3	0.42	97.6	0.42	111.2	0.54	87.8	-0.16
**Estriol**	50-27-1	free base	Estrogen/progestogen Receptor	81.3	-0.21	117.5	1.14	123.6	1.07	108.4	0.49
**Estrone**	53-16-7	free base	Estrogen/progestogen Receptor	98.8	0.53	114.2	1.03	96.9	-0.07	90.9	-0.06
**Ethynodiol diacetate**	297-76-7	free base	Estrogen/progestogen Receptor	115.5	1.23	115.4	1.07	110.4	0.51	126.6	1.07
**Etizolam**	40054-69-1	free base	Others	104.3	0.76	118.3	1.17	100.0	0.07	122.0	0.92
**Etodolac**	41340-25-4	free base	COX	95.8	0.40	52.3	-1.22	138.3	1.69	77.3	-0.49
**Etomidate**	33125-97-2	free base	GABA Receptor	99.9	0.57	66.9	-0.69	115.6	0.73	99.7	0.22
**Etoposide**	33419-42-0	free base	Topoisomerase	75.9	-0.45	69.7	-0.59	89.7	-0.37	11.8	-2.57
**ETP-46464**	1345675-02-6	free base	mTOR,ATM/ATR	61.8	-1.04	57.3	-1.04	87.8	-0.45	75.1	-0.56
**Etravirine (TMC125)**	269055-15-4	free base	Reverse Transcriptase	119.7	1.41	115.3	1.07	115.0	0.70	98.3	0.17
**EUK 134**	81065-76-1	free base	Beta Amyloid	64.8	-0.92	114.7	1.04	83.8	-0.62	104.4	0.37
**Evacetrapib (LY2484595)**	1186486-62-3	free base	CETP	81.9	-0.19	70.3	-0.57	106.9	0.36	78.2	-0.46
**Everolimus (RAD001)**	159351-69-6	free base	mTOR	90.1	0.15	84.0	-0.07	64.7	-1.44	64.2	-0.91
**EX 527 (Selisistat)**	49843-98-3	free base	Sirtuin	89.0	0.11	51.4	-1.26	105.4	0.29	94.9	0.07
**Exemestane**	107868-30-4	free base	Aromatase	96.5	0.43	102.2	0.59	120.5	0.94	94.7	0.06

**Famotidine**	76824-35-6	free base	Histamine Receptor	98.1	0.50	116.0	1.09	103.3	0.20	99.6	0.22
**Fasudil (HA-1077) HCl**	105628-07-7	hydrochloride	ROCK,Autophagy	161.3	3.17	116.0	1.09	109.0	0.45	98.1	0.17
**Felodipine**	72509-76-3	free base	Calcium Channel	93.3	0.29	71.1	-0.54	106.8	0.35	149.7	1.80
**Ferrostatin-1 (Fer-1)**	347174-05-4	free base	Ferroptosis	72.9	-0.57	112.9	0.98			111.6	0.59
**Fesoterodine Fumarate**	286930-03-8	fumarate	AChR	108.6	0.94	59.9	-0.95	90.8	-0.32	80.9	-0.38
**Fexofenadine HCl**	153439-40-8	hydrochloride	Histamine Receptor	87.6	0.05	116.9	1.12	108.6	0.43	94.9	0.07
**FG-4592**	808118-40-3	free base	HIF	111.8	1.08	105.4	0.71	92.0	-0.27	111.0	0.58
**FH535**	108409-83-2	free base	Wnt/beta-catenin	103.7	0.73	114.7	1.04	100.8	0.10	87.9	-0.16
**Filgotinib (GLPG0634)**	1206161-97-8	free base	JAK	69.6	-0.71	64.0	-0.80	83.3	-0.64	123.8	0.98
**Finasteride**	98319-26-7	free base	5-alpha Reductase	84.5	-0.08	106.1	0.73	118.3	0.84	64.4	-0.90
**Fingolimod (FTY720) HCl**	162359-56-0	hydrochloride	S1P Receptor	75.2	-0.47	86.3	0.01	78.5	-0.85	128.8	1.14
**Flavopiridol HCl**	131740-09-5	hydrochloride	CDK	54.1	-1.37	20.7	-2.37	31.6	-2.84	21.8	-2.25
**Flavoxate HCl**	3717-88-2	hydrochloride	AChR	91.5	0.21	96.9	0.40	96.6	-0.08	121.7	0.92
**FLI-06**	313967-18-9	free base	Notch	82.8	-0.15	116.7	1.12	110.4	0.51	99.9	0.23
**Flumazenil**	78755-81-4	free base	GABA Receptor	78.2	-0.35	114.6	1.04	111.4	0.55	95.4	0.08
**Flumequine**	42835-25-6	free base	Topoisomerase	114.1	1.17	114.8	1.05	117.2	0.79	142.0	1.56
**Flunarizine 2HCl**	30484-77-6	2HCl	Calcium Channel	97.3	0.46	115.5	1.07	105.6	0.30	131.4	1.22
**Flunixin Meglumin**	42461-84-7	meglumine	COX	102.2	0.67	109.9	0.87	91.9	-0.28	95.6	0.09
**Fluoxetine HCl**	56296-78-7	hydrochloride	5-HT Receptor	86.0	-0.02	116.0	1.09	92.8	-0.24	70.1	-0.72
**Flutamide**	13311-84-7	free base	Androgen Receptor	100.2	0.58	117.1	1.13	82.9	-0.66	127.8	1.11
**Fluvastatin Sodium**	93957-55-2	sodium	HMG-CoA Reductase	101.3	0.63	116.7	1.11	102.9	0.19	86.7	-0.19
**Fluvoxamine maleate**	61718-82-9	maleate	5-HT Receptor	100.9	0.62	49.3	-1.33	87.6	-0.46	78.5	-0.45
**Foretinib (GSK1363089)**	849217-64-7	free base	VEGFR,c-Met	48.0	-1.63	76.2	-0.35	65.5	-1.40	67.1	-0.81
**Formestane**	566-48-3	free base	Aromatase	117.1	1.30	94.9	0.32	90.6	-0.33	88.1	-0.15
**Formoterol Hemifumarate**	43229-80-7	fumarate	Adrenergic Receptor	106.2	0.84	115.7	1.08	109.5	0.47	91.5	-0.04
**Forskolin**	66575-29-9	free base	cAMP	123.6	1.58	90.9	0.18	90.7	-0.33	128.0	1.12
**Fostamatinib (R788)**	901119-35-5	free base	Syk	68.1	-0.78	57.2	-1.05	108.5	0.43	92.9	0.00
**FPH2 (BRD-9424)**	957485-64-2	free base	Others	84.1	-0.10	96.6	0.39	104.0	0.23	83.2	-0.30
**Fulvestrant**	129453-61-8	free base	Estrogen/progestogen	108.2	0.92	83.2	-0.10	128.1	1.26	103.1	0.32

			Receptor								
**Gabexate Mesylate**	56974-61-9	mesylate	Proteasome	101.1	0.62	115.6	1.08	93.5	-0.21	98.7	0.19
**Galeterone**	851983-85-2	free base	P450 (e.g. CYP17),Androgen Receptor	94.8	0.36	65.1	-0.76	104.6	0.26	118.4	0.81
**Gallamine Triethiodide**	65-29-2	Iodine salt	AChR	90.8	0.19	116.9	1.12	113.6	0.64	101.8	0.28
**Ganetespib (STA-9090)**	888216-25-9	free base	HSP (e.g. HSP90)	57.0	-1.25	31.9	-1.97	49.4	-2.09	39.6	-1.69
**GDC-0068**	1001264-89-6	free base	Akt	64.6	-0.92	110.2	0.88	89.5	-0.38	106.0	0.42
**GDC-0152**	873652-48-3	free base	IAP	85.7	-0.03	115.4	1.07	82.9	-0.66	115.8	0.73
**GDC-0349**	1207360-89-1	free base	mTOR	54.1	-1.37	101.1	0.55	65.3	-1.41	105.7	0.41
**GDC-0879**	905281-76-7	free base	Raf	121.1	1.47	115.1	1.06	151.1	2.24	98.3	0.17
**GDC-0941**	957054-30-7	free base	PI3K	57.3	-1.24	84.1	-0.07	74.1	-1.03	83.3	-0.30
**GDC-0980 (RG7422)**	1032754-93-0	free base	mTOR,PI3K	55.6	-1.31	43.7	-1.54	78.7	-0.84	76.6	-0.51
**Gefitinib (ZD1839)**	184475-35-2	free base	EGFR	86.6	0.01	116.5	1.11	96.6	-0.08	118.1	0.80
**Geldanamycin**	30562-34-6	free base	HSP (e.g. HSP90),Autophagy	37.4	-2.08	38.8	-1.72	49.0	-2.10	41.5	-1.63
**Gemcitabine**	95058-81-4	free base	Autophagy,DNA/RNA Synthesis	75.6	-0.46	81.8	-0.15	65.2	-1.41	16.5	-2.42
**Genistein**	446-72-0	free base	Topoisomerase,EGFR	83.2	-0.14	116.5	1.11	125.8	1.16	68.8	-0.76
**Gestodene**	60282-87-3	free base	Estrogen/progestogen Receptor	88.1	0.07	55.9	-1.09	92.8	-0.24	68.4	-0.77
**GF109203X**	133052-90-1	free base	PKC	67.5	-0.80	74.6	-0.41			91.7	-0.04
**Gimeracil**	103766-25-2	free base	Dehydrogenase	98.3	0.50	115.9	1.09	85.8	-0.54	95.1	0.07
**Ginkgolide A**	15291-75-5	free base	GABA Receptor	100.2	0.58	116.1	1.09	103.2	0.20	89.4	-0.11
**Ginkgolide B**	15291-77-7	free base	PAFR	99.3	0.55	116.8	1.12	106.7	0.35	98.4	0.18
**Givinostat (ITF2357)**	732302-99-7	chloride	HDAC	44.6	-1.77	37.8	-1.75	46.5	-2.21	-0.4	-2.95
**Gliclazide**	21187-98-4	free base	Potassium Channel	83.2	-0.14	69.3	-0.61	97.5	-0.04	109.7	0.54
**Glimepiride**	93479-97-1	free base	DPP-4	86.3	-0.01	96.2	0.37	134.3	1.52	104.4	0.37
**Gliquidone**	33342-05-1	free base	Potassium Channel	98.3	0.50	80.1	-0.22	112.1	0.58	130.5	1.19
**GNE-0877**	1374828-69-9	free base	LRRK2	83.9	-0.10	116.0	1.09	98.1	-0.02	142.0	1.56
**GNE-7915**	1351761-44-8	free base	LRRK2	109.4	0.97	54.5	-1.14	98.9	0.02	76.2	-0.53
**GNE-9605**	1536200-31-3	free base	LRRK2	82.2	-0.18	111.2	0.92	115.4	0.72	78.2	-0.46

**GNF-2**	778270-11-4	free base	Bcr-Abl	82.0	-0.19	49.4	-1.33	102.7	0.18	98.1	0.17
**GNF-5**	778277-15-9	free base	Bcr-Abl	85.4	-0.04	60.7	-0.92	72.4	-1.11	82.8	-0.32
**Go 6983**	133053-19-7	free base	PKC	71.2	-0.64	44.4	-1.51	79.8	-0.79	98.0	0.16
**Golgicide A**	1139889-93-2	free base	ATPase	86.6	0.01	117.1	1.13	93.6	-0.21	101.1	0.26
**Golvatinib (E7050)**	928037-13-2	free base	VEGFR,c-Met	72.6	-0.59	65.1	-0.76	115.9	0.74	95.1	0.07
**GS-9973**	1229208-44-9	free base	Syk	95.3	0.38	78.9	-0.26	110.7	0.52	121.6	0.91
**GSK J4 HCl**	1373423-53-0	hydrochloride	Histone demethylases	102.2	0.67	89.1	0.11	80.0	-0.78	43.6	-1.56
**GSK1059615**	958852-01-2	free base	PI3K,mTOR	45.2	-1.75	47.8	-1.39	81.7	-0.71	42.0	-1.61
**GSK1292263**	1032823-75-8	free base	GPR	108.6	0.94	96.1	0.37	95.1	-0.14	110.1	0.55
**GSK1838705A**	1116235-97-2	free base	IGF-1R,ALK	42.4	-1.86	48.3	-1.37	91.2	-0.31	96.4	0.11
**GSK1904529A**	1089283-49-7	free base	IGF-1R	90.2	0.16	62.8	-0.84	81.6	-0.72	127.7	1.10
**GSK2126458 (GSK458)**	1086062-66-9	free base	PI3K,mTOR	33.2	-2.26	32.1	-1.96	55.3	-1.83	14.7	-2.47
**GSK2334470**	1227911-45-6	free base	PDK-1	75.9	-0.44	99.4	0.49			75.4	-0.55
**GSK256066**	801312-28-7	free base	PDE	93.1	0.28	46.8	-1.43	133.9	1.50	94.0	0.04
**GSK2606414**	1337531-36-8	free base	PERK	77.4	-0.38	63.8	-0.81	101.3	0.12	75.4	-0.55
**GSK2636771**	1372540-25-4	free base	PI3K	81.9	-0.19	73.7	-0.45	102.9	0.19	77.8	-0.47
**GSK2656157**	1337532-29-2	free base	PERK	81.2	-0.22	65.5	-0.75	75.6	-0.97	113.0	0.64
**GSK2656157**	1337532-29-2	free base	PERK	73.6	-0.54	87.7	0.06	80.2	-0.78	116.5	0.75
**GSK3787**	188591-46-0	free base	PPAR	88.0	0.07	110.5	0.89	105.8	0.31	75.0	-0.57
**GSK429286A**	864082-47-3	free base	ROCK	117.0	1.30	106.4	0.74	112.9	0.61	86.7	-0.19
**GSK461364**	929095-18-1	free base	PLK	76.3	-0.43	49.7	-1.32	58.9	-1.68	45.2	-1.51
**GSK690693**	937174-76-0	free base	Akt	71.1	-0.65	111.2	0.92	89.8	-0.37	120.1	0.86
**GSK923295**	1088965-37-0	free base	Kinesin	68.2	-0.77	85.4	-0.02			72.8	-0.63
**Guanabenz Acetate**	23256-50-0	acetate	Adrenergic Receptor	82.0	-0.19	117.0	1.13	113.1	0.62	119.0	0.83
**GW0742**	317318-84-6	free base	PPAR	71.8	-0.62	66.5	-0.71	112.1	0.58	156.2	2.01
**GW2580**	870483-87-7	free base	CSF-1R	82.1	-0.18	83.9	-0.08	100.8	0.10	84.6	-0.26
**GW3965 HCl**	405911-17-3	hydrochloride	Liver X Receptor	80.6	-0.24	92.5	0.24	117.0	0.79	108.8	0.51
**GW4064**	278779-30-9	free base	FXR	90.0	0.15	115.5	1.07	115.1	0.71	118.5	0.81
**GW5074**	220904-83-6	free base	Raf	93.3	0.29	90.0	0.15	113.6	0.64	99.7	0.22

**GW788388**	452342-67-5	free base	TGF-beta/Smad	82.0	-0.19	114.6	1.04	135.8	1.59	104.9	0.38
**GW842166X**	666260-75-9	free base	Cannabinoid Receptor	92.7	0.27	114.5	1.04	117.5	0.81	119.7	0.85
**GW9508**	885101-89-3	free base	GPR	83.5	-0.12	64.4	-0.78	79.6	-0.80	142.9	1.59
**GW9662**	22978-25-2	free base	PPAR	86.1	-0.01	70.2	-0.57	108.6	0.43	107.7	0.47
**GZD824**	1421783-64-3	mesylate	Bcr-Abl	57.5	-1.22	66.9	-0.70			106.8	0.44
**H 89 2HCl**	130964-39-5	2HCl	PKA	92.5	0.26	62.9	-0.84	100.3	0.08	84.2	-0.27
**HA14-1**	65673-63-4	free base	Bcl-2	56.7	-1.26	49.7	-1.32	63.0	-1.51	118.1	0.80
**HER2-Inhibitor-1**	937265-83-3	free base	HER2,EGFR	111.1	1.05	93.4	0.27	111.3	0.55	137.5	1.42
**Hesperadin**	422513-13-1	free base	Aurora Kinase	53.9	-1.38	52.0	-1.24	43.7	-2.32	70.5	-0.71
**Hesperetin**	520-33-2	free base	Histamine Receptor,TGF-beta/Smad	114.9	1.20	54.0	-1.16	100.0	0.06	88.2	-0.14
**Hexestrol**	84-16-2	free base	Estrogen/progestogen Receptor	97.2	0.46	117.3	1.14	124.3	1.10	129.9	1.17
**Histamine 2HCl**	56-92-8	hydrochloride	Histamine Receptor	91.9	0.23	81.8	-0.15	82.3	-0.69	90.2	-0.08
**HJC0350**	885434-70-8	free base	Others	106.0	0.83	97.7	0.43	102.0	0.15	105.5	0.40
**HMN-214**	173529-46-9	free base	PLK	57.5	-1.22	77.6	-0.31	70.7	-1.18	46.4	-1.47
**HO-3867**	1172133-28-6	free base	STAT	111.3	1.06	96.1	0.37	111.8	0.56	56.9	-1.14
**Homatropine Bromide**	51-56-9	bromide	AChR	96.8	0.44	64.4	-0.78	116.7	0.77	94.9	0.07
**Homatropine Methylbromide**	80-49-9	Methylbromid e	AChR	88.3	0.08	89.5	0.13	107.8	0.40	128.1	1.12
**Honokiol**	35354-74-6	free base	MEK,Akt	107.4	0.89	62.3	-0.86	99.2	0.03	82.1	-0.34
**HS-173**	1276110-06-5	free base	PI3K	91.2	0.20	57.3	-1.05	69.5	-1.23	48.3	-1.41
**HSP990 (NVP-HSP990)**	934343-74-5	free base	HSP (e.g. HSP90)	50.2	-1.53	32.0	-1.96			38.9	-1.71
**Hydroxyzine 2HCl**	2192-20-3	hydrochloride	Histamine Receptor	112.8	1.12	115.0	1.06	115.5	0.72	94.9	0.06
**Hyoscyamine**	101-31-5	free base	AChR	88.1	0.07	112.9	0.98	102.4	0.17	119.2	0.84
**I-BET151 (GSK1210151A)**	1300031-49-5	free base	Epigenetic Reader Domain	41.9	-1.89	89.1	0.11	77.7	-0.88	44.2	-1.54
**I-BET-762**	1260907-17-2	free base	Epigenetic Reader Domain	51.0	-1.50	55.0	-1.13			43.6	-1.56
**Ibrutinib (PCI-32765)**	936563-96-1	free base	BTK	79.4	-0.30	47.2	-1.41	117.8	0.82	117.7	0.79
**Ibuprofen**	15687-27-1	free base	COX	124.3	1.61	81.4	-0.17	108.2	0.41	78.6	-0.45
**Ibutilide Fumarate**	122647-32-9	fumarate	Sodium Channel	150.6	2.72	110.7	0.90	98.4	0.00	105.0	0.38
**Icariin**	489-32-7	free base	PDE	108.0	0.92	73.1	-0.47	93.2	-0.22	115.4	0.72

**ICG-001**	780757-88-2	free base	Wnt/beta-catenin	75.7	-0.45	67.1	-0.69	109.7	0.48	49.8	-1.36
**Icotinib**	610798-31-7	free base	EGFR	82.4	-0.17	114.8	1.05	95.3	-0.13	97.0	0.13
**Idarubicin HCl**	57852-57-0	hydrochloride	Topoisomerase	4.8	-3.46	4.9	-2.95	23.3	-3.19	2.1	-2.87
**IEM 1754 dihydrobroMide**	162831-31-4	dihydrobroMide	GluR,5-HT Receptor	76.2	-0.43	60.2	-0.94	121.8	0.99	99.5	0.21
**Ifenprodil Tartrate**	23210-58-4	tartrate	GluR	103.4	0.72	115.8	1.08	104.8	0.27	117.4	0.78
**IKK-16 (IKK Inhibitor VII)**	873225-46-8	free base	IκB/IKK	90.8	0.18	47.7	-1.39	113.9	0.65	84.6	-0.26
**Ilomastat (GM6001, Galardin)**	142880-36-2	free base	MMP	80.9	-0.23	113.5	1.00			86.9	-0.19
**IM-12**	1129669-05-1	free base	GSK-3	81.4	-0.21	70.6	-0.56	101.0	0.11	112.3	0.62
**Imatinib (STI571)**	152459-95-5	free base	PDGFR	88.9	0.10	52.2	-1.23	156.4	2.46	116.2	0.74
**Imatinib Mesylate (STI571)**	220127-57-1	mesylate	c-Kit,Bcr-Abl,PDGFR	91.2	0.20	116.9	1.12	102.5	0.17	121.2	0.90
**IMD 0354**	978-62-1	free base	IκB/IKK	65.7	-0.88	57.2	-1.05	113.1	0.62	77.8	-0.47
**Imidapril HCl**	89396-94-1	free base	RAAS	126.9	1.72	114.3	1.03	94.5	-0.17	122.2	0.93
**INCB024360**	914471-09-3	free base	IDO	89.4	0.13	106.9	0.76	75.7	-0.97	88.0	-0.15
**Indacaterol Maleate**	753498-25-8	maleate	Adrenergic Receptor	94.5	0.34	109.1	0.84	103.7	0.22	119.6	0.85
**Indirubin**	479-41-4	free base	GSK-3	104.9	0.78	72.8	-0.48	89.7	-0.37	102.3	0.30
**INH1**	313553-47-8	free base	Microtubule Associat	101.3	0.63	79.2	-0.25	87.3	-0.47	100.7	0.25
**INH6**	1001753-24-7	free base	Microtubule Associat	85.5	-0.04	92.1	0.22	99.7	0.05	88.3	-0.14
**Iniparib (BSI-201)**	160003-66-7	free base	PARP	92.9	0.28	101.2	0.55	94.7	-0.16	92.2	-0.02
**INK 128 (MLN0128)**	1224844-38-5	free base	mTOR	38.0	-2.05	39.3	-1.70	35.2	-2.69	28.9	-2.02
**INO-1001**	3544-24-9	free base	PARP	108.9	0.95	117.4	1.14	111.7	0.56	92.6	-0.01
**IOWH032**	1191252-49-9	free base	CFTR	80.4	-0.26	93.8	0.28	82.0	-0.70	103.2	0.33
**IOX1**	5852-78-8	free base	Histone demethylases	74.9	-0.49	104.0	0.66			88.2	-0.15
**IOX2**	931398-72-0	free base	HIF	92.0	0.24	110.4	0.89	99.0	0.02	107.4	0.46
**IPA-3**	42521-82-4	free base	PAK	84.1	-0.10	75.8	-0.37			80.1	-0.40
**IPI-145 (INK1197)**	1201438-56-3	free base	PI3K	81.3	-0.22	114.8	1.05	98.5	0.00	110.6	0.56
**IPI-145 (INK1197)**	1201438-56-3	free base	PI3K	89.3	0.12	100.2	0.52	91.8	-0.28	111.5	0.59
**Irinotecan**	97682-44-5	free base	Topoisomerase	56.7	-1.26	99.2	0.48	108.9	0.44	28.6	-2.03
**Irinotecan HCl Trihydrate**	136572-09-3	hydrate	Topoisomerase	66.3	-0.85	49.1	-1.34	93.8	-0.20	77.8	-0.48

**Irsogladine**	57381-26-7	free base	PDE,AChR	106.4	0.85	115.5	1.07	98.2	-0.01	84.2	-0.27
**Isoprenaline HCl**	51-30-9	hydrochloride	Adrenergic Receptor	76.2	-0.43	116.2	1.10	120.7	0.94	104.0	0.35
**Isotretinoin**	4759-48-2	free base	Hydroxylase	91.2	0.20	63.4	-0.82	121.2	0.96	93.1	0.01
**Ispinesib (SB-715992)**	336113-53-2	free base	Kinesin	46.3	-1.70	91.6	0.21	45.9	-2.23	32.4	-1.91
**Isradipine**	75695-93-1	free base	Calcium Channel	87.6	0.05	115.5	1.07	110.7	0.52	73.6	-0.61
**IU1**	314245-33-5	free base	DUB	89.3	0.12	108.7	0.82			121.7	0.91
**Ivabradine HCl**	148849-67-6	hydrochloride	Adrenergic Receptor	98.3	0.50	75.4	-0.38	88.9	-0.41	129.4	1.16
**Ivacaftor (VX-770)**	873054-44-5	free base	CFTR	98.4	0.51	113.0	0.98	113.9	0.66	95.7	0.09
**IWP-2**	686770-61-6	free base	Wnt/beta-catenin	129.7	1.83	88.1	0.07			114.5	0.69
**IWP-L6**	1427782-89-5	free base	Wnt/beta-catenin	79.2	-0.31	54.3	-1.15	97.9	-0.03	79.1	-0.44
**IWR-1-endo**	1127442-82-3	free base	Wnt/beta-catenin	106.0	0.83	100.2	0.52			113.7	0.66
**JNJ-1661010**	681136-29-8	free base	FAAH	93.5	0.30	113.4	0.99	124.3	1.10	113.4	0.65
**JNJ-26854165 (Serdemetan)**	881202-45-5	free base	p53,E3 Ligase	105.6	0.81	108.5	0.82	130.6	1.36	86.7	-0.19
**JNJ-38877605**	943540-75-8	free base	c-Met	130.2	1.86	111.7	0.93	113.9	0.65	64.2	-0.91
**JNJ-7706621**	443797-96-4	free base	CDK,Aurora Kinase	71.9	-0.62	74.2	-0.43	82.9	-0.66	56.0	-1.17
**JNJ-7777120**	459168-41-3	free base	Histamine Receptor	91.1	0.20	99.7	0.50	101.8	0.14	97.2	0.14
**JNK Inhibitor IX**	312917-14-9	free base	JNK	61.3	-1.06	49.7	-1.32	45.2	-2.26	33.8	-1.87
**JNK-IN-8**	1410880-22-6	free base	JNK	91.8	0.23	71.2	-0.54	79.7	-0.80	120.4	0.87
**JSH-23**	749886-87-1	free base	NF-κB	80.6	-0.25	102.8	0.61	99.6	0.05	115.5	0.72
**JTC-801**	244218-51-7	hydrochloride	Opioid Receptor	83.8	-0.11	61.3	-0.90	124.1	1.09	135.7	1.36
**K02288**	1431985-92-0	free base	TGF-beta/Smad	74.2	-0.52	116.4	1.11	125.4	1.15	79.8	-0.41
**Ketanserin**	74050-98-9	free base	5-HT Receptor	116.3	1.27	106.9	0.76	96.5	-0.09	84.9	-0.25
**Ketoconazole**	65277-42-1	free base	P450 (e.g. CYP17)	99.4	0.55	74.6	-0.42	111.3	0.55	69.1	-0.75
**Ketoprofen**	22071-15-4	free base	COX	83.2	-0.14	115.3	1.06	99.5	0.04	78.8	-0.44
**Ketorolac**	74103-07-4	tromethamine	COX	84.3	-0.09	115.1	1.06	89.4	-0.39	94.0	0.04
**Ketotifen Fumarate**	34580-14-8	fumarate	Histamine Receptor	98.9	0.53	115.4	1.07	101.3	0.12	147.0	1.72
**Ki16198**	355025-13-7	free base	LPA Receptor	101.2	0.63	73.0	-0.47	97.5	-0.04	94.9	0.07
**Ki16425**	355025-24-0	free base	LPA Receptor	93.8	0.31	55.9	-1.09	104.7	0.27	89.1	-0.12
**Ki8751**	228559-41-9	free base	PDGFR,c-Kit,VEGFR	71.3	-0.64	50.8	-1.28	53.3	-1.92	35.0	-1.83

**KN-62**	127191-97-3	free base	Others	121.2	1.47	105.8	0.72	105.2	0.29	103.3	0.33
**KN-93 Phosphate**	1188890-41-6	free base	Others	86.7	0.01	76.1	-0.36	97.4	-0.05	83.9	-0.28
**KPT-185**	1333151-73-7	free base	CRM1	58.5	-1.18	38.5	-1.73			3.7	-2.82
**KPT-276**	1421919-75-6	free base	CRM1	68.7	-0.75	76.9	-0.33			7.8	-2.69
**KPT-330**	1393477-72-9	free base	CRM1	59.6	-1.13	49.7	-1.32			3.6	-2.83
**K-Ras(G12C) inhibitor 6**	N/A	free base	Ras	105.9	0.83	116.9	1.12	100.5	0.08	84.3	-0.27
**K-Ras(G12C) inhibitor 9**	1469337-91-4	free base	Rho	116.1	1.26	88.8	0.10	109.7	0.48	99.6	0.21
**KRN 633**	286370-15-8	free base	PDGFR,VEGFR	80.4	-0.26	116.0	1.09	133.5	1.49	97.8	0.16
**KU-0063794**	938440-64-3	free base	mTOR	70.6	-0.67	96.5	0.38	94.0	-0.19	59.1	-1.07
**KU-55933 (ATM Kinase Inhibitor)**	587871-26-9	free base	ATM/ATR	100.4	0.59	99.2	0.48	104.5	0.25	88.0	-0.15
**KU-60019**	925701-49-1	free base	ATM/ATR	96.9	0.45	115.1	1.06	122.3	1.01	79.0	-0.44
**KW-2449**	1000669-72-6	free base	Aurora Kinase,Bcr-Abl,FLT3	85.6	-0.03	55.1	-1.12	91.7	-0.29	74.2	-0.59
**KW-2478**	819812-04-9	free base	HSP (e.g. HSP90)	53.9	-1.38	62.3	-0.86	49.5	-2.08	53.7	-1.24
**KX2-391**	897016-82-9	free base	Src	45.9	-1.72	50.2	-1.30	59.7	-1.65	26.7	-2.09
**KY02111**	1118807-13-8	free base	Wnt/beta-catenin	88.7	0.10	88.2	0.08			117.7	0.79
**Labetalol HCl**	32780-64-6	Salt	Adrenergic Receptor	84.8	-0.07	82.4	-0.13	80.3	-0.77	93.6	0.03
**Lacidipine**	103890-78-4	free base	Calcium Channel	80.6	-0.25	116.2	1.10	87.9	-0.45	123.0	0.96
**L-Adrenaline**	51-43-4	free base	Adrenergic Receptor	105.1	0.79	50.8	-1.28	134.2	1.52	117.5	0.78
**Lafutidine**	118288-08-7	free base	Histamine Receptor	100.1	0.58	116.3	1.10	96.6	-0.08	141.7	1.55
**Lamivudine**	134678-17-4	free base	Reverse Transcriptase	107.6	0.90	114.9	1.05	109.2	0.45	76.5	-0.52
**Lamotrigine**	84057-84-1	free base	Sodium Channel,5-HT Receptor	114.9	1.21	119.2	1.21	110.6	0.52	115.3	0.71
**Lansoprazole**	103577-45-3	free base	Proton Pump	78.8	-0.32	70.8	-0.55	101.8	0.14	94.9	0.07
**Lapatinib (GW-572016) Ditosylate**	388082-77-7	ditosylat	HER2,EGFR	95.7	0.39	115.7	1.08	93.2	-0.22	109.2	0.52
**LAQ824 (Dacinostat)**	404951-53-7	free base	HDAC	2.3	-3.56	21.6	-2.34	17.2	-3.45	1.9	-2.88
**Laquinimod**	248281-84-7	free base	Others	109.5	0.98	109.0	0.84	95.9	-0.11	106.0	0.42
**LB42708**	226929-39-1	free base	Ftase	72.3	-0.60	57.8	-1.03	60.4	-1.62	77.6	-0.48
**LDC000067**	1073485-20-7	free base	CDK	95.9	0.40	115.4	1.07	92.2	-0.27	77.1	-0.50
**LDE225 (NVP-LDE225,Erismodegib)**	956697-53-3	free base	Hedgehog/Smoothened	81.3	-0.21	81.4	-0.17	93.4	-0.22	117.1	0.77

**LDK378**	1032900-25-6	free base	ALK	65.0	-0.91	80.1	-0.21	72.2	-1.11	120.3	0.87
**LDN-212854**	1432597-26-6	free base	BMP	76.8	-0.41	109.5	0.85			110.0	0.54
**LDN-57444**	668467-91-2	free base	DUB	81.3	-0.22	64.9	-0.77			112.5	0.62
**LEE011**	1211441-98-3	free base	CDK	67.0	-0.82	115.8	1.08	67.3	-1.32	115.8	0.73
**Lenalidomide (CC-5013)**	191732-72-6	free base	TNF-alpha	98.0	0.49	88.3	0.08	76.8	-0.92	117.0	0.77
**Lenvatinib (E7080)**	417716-92-8	free base	VEGFR	106.7	0.86	116.9	1.12	98.2	-0.01	61.9	-0.98
**Letrozole**	112809-51-5	free base	Aromatase	94.1	0.33	77.5	-0.31	131.6	1.41	110.1	0.55
**Leupeptin Hemisulfate**	103476-89-7	free base	Serine Protease	77.0	-0.40	83.5	-0.09	97.8	-0.03	82.9	-0.32
**Levodropropizine**	99291-25-5	free base	Histamine Receptor	109.5	0.98	117.4	1.14	102.4	0.17	62.7	-0.95
**Levosulpiride**	23672-07-3	free base	Dopamine Receptor	106.1	0.83	98.6	0.46	92.8	-0.24	134.5	1.32
**LGK-974**	1243244-14-5	free base	Wnt/beta-catenin	68.5	-0.76	70.6	-0.56			141.6	1.55
**LGX818**	1269440-17-6	free base	RAF	86.0	-0.02	62.1	-0.87			120.8	0.89
**Licofelone**	156897-06-2	free base	COX	115.5	1.23	112.5	0.96	84.6	-0.59	116.3	0.74
**Lidocaine**	137-58-6	free base	Histamine Receptor	82.6	-0.16	53.9	-1.17	89.2	-0.39	88.0	-0.15
**Linagliptin**	668270-12-0	free base	DPP-4	97.3	0.46	64.8	-0.77	116.9	0.78	117.9	0.79
**Linifanib (ABT-869)**	796967-16-3	free base	CSF-1R,PDGFR,VEGFR	102.5	0.68	109.2	0.84	84.3	-0.60	122.0	0.92
**Lomeguatrib**	192441-08-0	free base	DNA Methyltransferase	75.5	-0.46	70.5	-0.56	102.9	0.19	130.5	1.19
**Lomitapide**	182431-12-5	free base	Others	77.1	-0.39	66.6	-0.71	102.6	0.18	131.0	1.21
**Lonafarnib**	193275-84-2	free base	Transferase	94.5	0.34	105.9	0.72	94.4	-0.17	92.4	-0.01
**Loperamide HCl**	34552-83-5	hydrochloride	Opioid Receptor,Autophagy	103.3	0.71	115.2	1.06	121.7	0.99	123.2	0.96
**Lopinavir**	192725-17-0	free base	HIV Protease	100.6	0.60	50.5	-1.29	122.8	1.03	75.0	-0.57
**Loratadine**	79794-75-5	free base	Histamine Receptor	85.8	-0.03	51.1	-1.27	125.9	1.17	81.8	-0.35
**Lorcaserin HCl**	846589-98-8	hydrochloride	5-HT Receptor	103.2	0.71	112.4	0.96	94.9	-0.15	89.8	-0.10
**Lornoxicam**	70374-39-9	free base	COX	99.0	0.53	115.4	1.07	101.1	0.11	127.9	1.11
**Losartan Potassium (DuP 753)**	124750-99-8	potassium	RAAS	82.9	-0.15	109.5	0.85	121.6	0.98	86.2	-0.21
**Losmapimod (GW856553X)**	585543-15-3	free base	p38 MAPK	75.3	-0.47	62.1	-0.87			105.0	0.39
**Lovastatin**	75330-75-5	free base	HMG-CoA Reductase	84.6	-0.08	63.3	-0.82	91.6	-0.29	124.5	1.00
**Loxistatin Acid (E-64C)**	76684-89-4	free base	Cysteine protease	93.3	0.29	116.7	1.12	90.9	-0.32	85.3	-0.24
**Lumiracoxib**	220991-20-8	free base	COX	101.9	0.66	67.2	-0.68	114.1	0.66	104.2	0.36

**Luteolin**	491-70-3	free base	PDE	113.9	1.16	110.2	0.88	99.8	0.06	82.3	-0.33
**LY2157299**	700874-72-2	free base	TGF-beta/Smad	100.1	0.58	54.8	-1.14	100.2	0.07	118.0	0.80
**LY2228820**	862507-23-1	mesylate	p38 MAPK	111.0	1.04	114.4	1.03	105.0	0.28	63.5	-0.93
**LY2603618**	911222-45-2	free base	Chk	63.9	-0.95	40.7	-1.65	127.6	1.24	35.2	-1.83
**LY2784544**	1229236-86-5	free base	JAK	82.7	-0.16	53.5	-1.18	65.9	-1.38	77.1	-0.50
**LY2811376**	1194044-20-6	free base	Beta Amyloid,Gamma-secretase	90.7	0.18	104.0	0.65	85.4	-0.56	82.4	-0.33
**LY2835219**	1231930-82-7	mesylate	CDK	57.3	-1.23	71.6	-0.52			76.9	-0.50
**LY294002**	154447-36-6	free base	Autophagy,PI3K	109.0	0.96	115.9	1.09	100.8	0.10	88.6	-0.13
**LY411575**	209984-57-6	free base	Gamma-secretase	115.5	1.23	64.6	-0.78	111.0	0.53	108.4	0.50
**M344**	251456-60-7	free base	HDAC	35.4	-2.16	48.2	-1.38	74.0	-1.04	2.9	-2.85
**Macitentan**	441798-33-0	free base	Endothelin Receptor	81.2	-0.22	61.0	-0.91	105.3	0.29	129.2	1.15
**Manidipine**	89226-50-6	free base	Calcium Channel	94.6	0.34	115.8	1.08	148.5	2.13	125.6	1.04
**Manidipine 2HCl**	89226-75-5	dihydrochloride	Calcium Channel	99.1	0.54	112.7	0.97	122.4	1.02	117.9	0.79
**Maprotiline HCl**	10347-81-6	hydrochloride	Adrenergic Receptor	96.3	0.42	109.9	0.87	111.5	0.56	123.1	0.96
**Maraviroc**	376348-65-1	free base	CCR	91.4	0.21	114.6	1.04	109.8	0.48	98.9	0.19
**Masitinib (AB1010)**	790299-79-5	free base	PDGFR,c-Kit	102.8	0.69	114.5	1.04	81.2	-0.73	96.7	0.12
**MC1568**	852475-26-4	free base	HDAC	140.8	2.30	94.8	0.32	114.8	0.69	74.3	-0.59
**Mdivi-1**	338967-87-6	free base	Dynamin	79.3	-0.30	61.8	-0.88			101.1	0.26
**ME0328**	1445251-22-8	free base	PARP	102.9	0.70	116.3	1.10	92.8	-0.24	89.8	-0.10
**Meclofenamate Sodium**	6385-02-0	free base	COX	106.8	0.86	117.2	1.14	93.1	-0.23	97.1	0.14
**Medetomidine HCl**	86347-15-1	hydrochloride	Adrenergic Receptor	90.4	0.17	119.1	1.20	104.1	0.24	113.1	0.64
**Medroxyprogesterone acetate**	71-58-9	free base	Estrogen/progestogen Receptor	89.4	0.13	114.8	1.05	123.9	1.08	93.0	0.01
**Mefenamic Acid**	61-68-7	free base	COX	97.8	0.48	107.9	0.80	100.8	0.10	113.7	0.66
**Mefloquine HCl**	51773-92-3	Salt	Others	83.5	-0.12	81.0	-0.18	78.7	-0.84	139.9	1.49
**Megestrol Acetate**	595-33-5	acetate	Estrogen Receptor,Androgen Receptor	83.6	-0.12	61.7	-0.88	106.6	0.34	82.2	-0.34
**MEK162 (ARRY-162, ARRY-438162)**	606143-89-9	free base	MEK	91.3	0.21	53.8	-1.17	50.7	-2.03	82.4	-0.33
**Memantine HCl**	41100-52-1	hydrochloride	AMPA Receptor-kainate	96.4	0.42	116.9	1.12	101.4	0.13	124.8	1.01

			Receptor-NMDA Receptor								
**Methazolamide**	554-57-4	free base	Carbonic Anhydrase	84.4	-0.09	118.3	1.17	109.4	0.46	126.3	1.06
**Methotrexate**	59-05-2	free base	DHFR	82.3	-0.17	63.3	-0.82	97.1	-0.06	75.6	-0.54
**Methscopolamine**	155-41-9	bromide	AChR	120.3	1.43	104.1	0.66	104.7	0.26	126.8	1.08
**MetoclopraMide HCl**	7232-21-5	Salt	Dopamine Receptor	100.4	0.59	82.9	-0.11	82.9	-0.66	132.1	1.24
**Metoprolol Tartrate**	392-17-7	tartrate	Adrenergic Receptor	93.8	0.31	71.8	-0.52	116.2	0.75	94.6	0.06
**MG-132**	133407-82-6	free base	Proteasome	2.4	-3.56	4.0	-2.98	33.5	-2.76	13.4	-2.52
**MGCD-265**	875337-44-3	free base	Tie-2,VEGFR,c-Met	99.5	0.56	51.5	-1.25	149.6	2.17	78.0	-0.47
**MI-2 (MALT1 inhibitor)**	1047953-91-2	free base	Others	83.4	-0.13	65.3	-0.75	91.9	-0.28	61.9	-0.98
**Mianserin HCl**	21535-47-7	hydrochloride	Histamine Receptor	122.4	1.52	64.8	-0.77	109.3	0.46	76.1	-0.53
**Mifepristone**	84371-65-3	free base	Estrogen/progestogen Receptor	98.5	0.51	85.9	0.00	117.5	0.81	98.6	0.18
**Milciclib (PHA-848125)**	802539-81-7	free base	CDK	65.1	-0.90	66.2	-0.72	66.8	-1.35	72.8	-0.63
**Milrinone**	78415-72-2	free base	ATPase,PDE	96.5	0.43	116.6	1.11	125.7	1.16	129.3	1.16
**Mirabegron**	223673-61-8	free base	Adrenergic Receptor	92.1	0.24	87.2	0.04	109.5	0.47	123.5	0.97
**MK-0752**	471905-41-6	free base	Gamma-secretase,Beta Amyloid	114.4	1.19	91.4	0.20	114.0	0.66	116.1	0.74
**MK-1775**	955365-80-7	free base	Wee1	86.8	0.02	24.7	-2.23	94.7	-0.16	34.7	-1.84
**MK-2048**	869901-69-9	free base	Integrase	79.1	-0.31	114.2	1.03	134.1	1.51	103.8	0.35
**MK-2206 2HCl**	1032350-13-2	dihydrochlorid e	Akt	54.4	-1.35	113.2	0.99	94.3	-0.18	99.7	0.22
**MK-2461**	917879-39-1	free base	c-Met,PDGFR,FGFR	77.3	-0.39	114.4	1.03	105.9	0.31	115.4	0.71
**MK-2866 (GTx-024)**	841205-47-8	free base	Androgen Receptor	118.0	1.34	116.1	1.09	115.2	0.71	66.5	-0.83
**MK-5108 (VX-689)**	1010085-13-8	free base	Aurora Kinase	44.6	-1.77	51.6	-1.25	68.8	-1.26	89.2	-0.11
**MK-801 (Dizocilpine)**	77086-21-6	free base	GluR	84.8	-0.07	49.5	-1.33	114.3	0.67	107.0	0.45
**MK-8245**	1030612-90-8	free base	Dehydrogenase	104.1	0.75	57.0	-1.05	105.0	0.28	96.8	0.13
**MK-8745**	885325-71-3	free base	Aurora Kinase	116.0	1.25	103.9	0.65	58.5	-1.70	90.8	-0.06
**MK-8776 (SCH 900776)**	891494-63-6	free base	CDK,Chk	68.1	-0.78	52.7	-1.21	123.4	1.06	50.0	-1.36
**ML130 (Nodinitib-1)**	799264-47-4	free base	NOD1	89.1	0.11	74.7	-0.41	103.2	0.20	102.3	0.30
**ML133 HCl**	1222781-70-5	hydrochloride	Potassium Channel	70.9	-0.66	61.9	-0.88	127.7	1.24	134.1	1.31
**ML167**	1285702-20-6	free base	CDK	90.4	0.17	65.0	-0.76	101.2	0.11	112.2	0.61

**ML323**	1572414-83-5	free base	DUB	63.9	-0.95	51.6	-1.25	101.7	0.14	80.4	-0.39
**ML347**	1062368-49-3	free base	BMP	73.4	-0.55	55.8	-1.10			128.2	1.12
**MLN2238**	1072833-77-2	free base	Proteasome	4.2	-3.48	4.0	-2.98	52.5	-1.95	2.8	-2.85
**MLN8054**	869363-13-3	free base	Aurora Kinase	60.2	-1.11	73.2	-0.47	64.4	-1.45	66.6	-0.83
**MLN9708**	1201902-80-8	free base	Proteasome	3.7	-3.50	4.2	-2.97	52.2	-1.96	2.4	-2.86
**MM-102**	1417329-24-8	free base	Histone Methyltransferase	80.3	-0.26	115.5	1.07	103.1	0.20	103.3	0.33
**MNS (3,4-Methylenedioxy-β-nitrostyrene, MDBN)**	1485-00-3	free base	p97	106.3	0.84	116.9	1.12	95.3	-0.14	124.0	0.99
**Mocetinostat (MGCD0103)**	726169-73-9	free base	HDAC	38.4	-2.03	66.8	-0.70	91.4	-0.30	0.0	-2.94
**Moclobemide (Ro 111163)**	71320-77-9	free base	MAO	94.8	0.36	80.9	-0.19	114.5	0.68	111.5	0.59
**Moexipril HCl**	82586-52-5	hydrochloride	RAAS	119.1	1.38	114.0	1.02	102.6	0.17	130.3	1.19
**Mosapride Citrate**	112885-42-4	citrate	5-HT Receptor	86.1	-0.01	72.8	-0.48	105.0	0.28	103.9	0.35
**Motesanib Diphosphate (AMG-706)**	857876-30-3	diphosphate	VEGFR,PDGFR,c-Kit	96.1	0.41	116.0	1.09	107.7	0.39	133.3	1.28
**Moxifloxacin HCl**	186826-86-8	hydrochloride	Topoisomerase	83.8	-0.11	64.8	-0.77	110.3	0.50	96.1	0.11
**MPEP**	96206-92-7	free base	GluR	92.9	0.28	79.9	-0.22	104.5	0.26	106.9	0.45
**MPI-0479605**	1246529-32-7	free base	Kinesin	99.5	0.55	98.4	0.45	84.3	-0.60	87.8	-0.16
**MRS 2578**	711019-86-2	free base	P2 Receptor	93.0	0.28	92.6	0.24	107.8	0.40	104.5	0.37
**Mubritinib (TAK 165)**	366017-09-6	free base	HER2	37.7	-2.06	45.8	-1.46	96.0	-0.11	80.2	-0.40
**Mupirocin**	12650-69-0	free base	DNA/RNA Synthesis	106.4	0.85	118.4	1.18	96.4	-0.09	125.0	1.02
**Mycophenolate Mofetil**	128794-94-5	free base	Dehydrogenase	55.7	-1.30	63.2	-0.83	86.4	-0.51	31.1	-1.96
**N6022**	1208315-24-5	free base	Others	75.5	-0.46	67.9	-0.66	74.7	-1.01	73.2	-0.62
**Nabumetone**	42924-53-8	free base	COX	99.5	0.55	116.7	1.12	102.1	0.15	119.3	0.84
**Nafamostat Mesylate**	82956-11-4	mesylate	Proteasome	83.9	-0.11	82.8	-0.11	85.5	-0.55	90.4	-0.08
**Naftopidil**	57149-07-2	free base	Adrenergic Receptor	122.9	1.55	111.6	0.93	83.3	-0.64	85.9	-0.22
**Naftopidil DiHCl**	57149-08-3	dihydrochloride	Adrenergic Receptor	86.4	0.00	51.2	-1.26	118.3	0.84	74.5	-0.58
**Naloxone HCl**	357-08-4	hydrochloride	Opioid Receptor	79.1	-0.31	118.6	1.19	109.7	0.48	147.6	1.73
**Naltrexone HCl**	16676-29-2	hydrochloride	Opioid Receptor	107.2	0.88	95.0	0.33	117.8	0.82	98.2	0.17
**Naphazoline HCl**	550-99-2	hydrochloride	Adrenergic Receptor	95.7	0.40	116.1	1.09	114.9	0.70	93.6	0.02
**Naproxen**	26159-34-2	sodium	COX	91.2	0.20	115.1	1.06	105.9	0.32	75.0	-0.56

**Naratriptan**	143388-64-1	free base	5-HT Receptor	87.3	0.04	68.1	-0.65	110.5	0.51	99.2	0.20
**Naringenin**	480-41-1	free base	P450 (e.g. CYP17)	108.8	0.95	77.7	-0.30	91.7	-0.29	105.9	0.42
**Nateglinide**	105816-04-4	free base	Potassium Channel	75.5	-0.46	47.6	-1.40	135.7	1.58	107.2	0.46
**Nebivolol**	152520-56-4	hydrochloride	Adrenergic Receptor	98.1	0.50	113.6	1.00	101.9	0.14	92.4	-0.01
**Necrostatin-1**	4311-88-0	free base	TNF-alpha	88.0	0.07	79.9	-0.22	97.7	-0.03	76.4	-0.52
**Nefiracetam**	77191-36-7	free base	GABA Receptor	91.8	0.23	116.4	1.10	106.6	0.35	143.9	1.62
**Nelfinavir Mesylate**	159989-65-8	Salt	HIV Protease	83.8	-0.11	116.9	1.12	79.4	-0.81	103.5	0.34
**Nevirapine**	129618-40-2	free base	Reverse Transcriptase	118.9	1.38	115.6	1.08	99.4	0.04	93.1	0.01
**Nexturastat A**	1403783-31-2	free base	HDAC	67.7	-0.79	109.3	0.85	103.0	0.19	138.2	1.44
**NH125**	278603-08-0	free base	ELF2	69.6	-0.71	59.9	-0.95	113.9	0.66	22.4	-2.23
**Nicorandil**	65141-46-0	free base	Potassium Channel	99.9	0.57	116.6	1.11	103.4	0.21	131.0	1.21
**Niflumic acid**	4394-00-7	free base	GABA Receptor	127.3	1.73	113.9	1.01	113.8	0.65	95.0	0.07
**Nilotinib (AMN-107)**	641571-10-0	free base	Bcr-Abl	75.6	-0.46	88.9	0.11	120.8	0.95	79.8	-0.41
**Nilvadipine**	75530-68-6	free base	Calcium Channel	78.9	-0.32	105.7	0.72	117.1	0.79	102.3	0.30
**Nimesulide**	51803-78-2	free base	COX	93.0	0.28	115.9	1.09	84.7	-0.59	97.5	0.15
**Nimodipine**	66085-59-4	free base	Calcium Channel,Autophagy	108.1	0.92	116.2	1.10	103.1	0.20	115.1	0.71
**Nintedanib (BIBF 1120)**	656247-17-5	free base	VEGFR,PDGFR,FGFR	82.7	-0.16	112.6	0.97	88.5	-0.42	119.2	0.84
**Nitrendipine**	39562-70-4	free base	Calcium Channel,Autophagy	79.0	-0.32	50.0	-1.31	117.0	0.79	98.4	0.18
**Nizatidine**	76963-41-2	free base	Histamine Receptor	121.6	1.49	101.6	0.57	103.6	0.22	124.8	1.01
**NLG919**	1402836-58-1	free base	IDO	81.7	-0.20	94.5	0.31			129.0	1.15
**NMDA (N-Methyl-D-aspartic acid)**	6384-92-5	free base	GluR	85.5	-0.04	76.5	-0.35	86.1	-0.53	121.3	0.90
**NMS-873**	1418013-75-8	free base	p97	31.4	-2.33	45.1	-1.49	149.9	2.18	36.0	-1.80
**NMS-E973**	1253584-84-7	free base	HSP90	51.4	-1.48	42.0	-1.60	44.5	-2.29	36.2	-1.79
**NMS-P937 (NMS1286937)**	1034616-18-6	free base	PLK	60.8	-1.09	50.5	-1.29			38.5	-1.72
**Nocodazole**	31430-18-9	free base	Microtubule associated	48.9	-1.59	85.1	-0.03	65.6	-1.39	27.2	-2.08
**NPS-2143**	284035-33-2	free base	CaSR	106.7	0.86	113.9	1.02	106.3	0.33	104.2	0.36
**NSC 23766**	1177865-17-6	hydrochloride	Rac	76.0	-0.44	60.9	-0.91	108.8	0.44	143.5	1.60
**NSC 319726**	71555-25-4	free base	p53	52.5	-1.44	47.5	-1.40			9.3	-2.65
**NSC 405020**	7497-07-6	free base	MMP	72.0	-0.61	114.3	1.03	93.7	-0.20	85.5	-0.23

**NSC697923**	343351-67-7	free base	E2	91.4	0.21	77.4	-0.31			47.0	-1.45
**NU6027**	220036-08-8	free base	CDK	67.4	-0.80	86.5	0.02			88.8	-0.13
**NU7441 (KU-57788)**	503468-95-9	free base	DNA-PK,PI3K	88.0	0.07	60.5	-0.93	126.9	1.21	92.8	0.00
**Nutlin-3**	890090-75-2	free base	E3 Ligase ,Mdm2	73.1	-0.56	92.0	0.22	68.8	-1.26	98.6	0.18
**Nutlin-3a**	675576-98-4	free base	Mdm2	59.7	-1.13	66.7	-0.70	81.6	-0.72	83.7	-0.29
**Nutlin-3b**	675576-97-3	free base	Mdm2	72.0	-0.61	67.8	-0.66	94.2	-0.18	78.8	-0.44
**NVP-ADW742**	475488-23-4	free base	IGF-1R	50.5	-1.52	93.3	0.27	69.6	-1.23	85.6	-0.23
**NVP-AEW541**	475489-16-8	free base	IGF-1R	48.8	-1.59	55.0	-1.13	76.2	-0.95	102.1	0.29
**NVP-BHG712**	940310-85-0	free base	Raf,Src,Bcr-Abl,VEGFR,Ephrin receptor	105.2	0.80	57.6	-1.03	94.2	-0.18	79.6	-0.42
**NVP-BSK805 2HCl**	1092499-93-8	hydrochloride	JAK	77.4	-0.38	55.0	-1.13	137.0	1.64	120.0	0.86
**NVP-BVU972**	1185763-69-2	free base	c-Met	76.2	-0.43	115.4	1.07	112.3	0.59	115.2	0.71
**Obatoclax Mesylate (GX15-070)**	803712-79-0	mesylate	Bcl-2,Autophagy	31.0	-2.35	56.4	-1.08	105.8	0.31	25.4	-2.14
**OC000459**	851723-84-7,950688-14-9	free base	GPR	87.7	0.05	50.6	-1.29	122.9	1.04	81.5	-0.36
**Odanacatib (MK-0822)**	603139-19-1	free base	Cathepsin K	131.3	1.90	113.2	0.99	107.8	0.40	68.2	-0.78
**OG-L002**	1357302-64-7	free base	Histone demethylases	70.3	-0.68	74.6	-0.42			109.5	0.53
**Olanzapine**	132539-06-1	free base	5-HT Receptor,Dopamine Receptor	80.0	-0.27	55.4	-1.11	120.5	0.94	103.8	0.35
**Olaparib (AZD2281, Ku-0059436)**	763113-22-0	free base	PARP	89.1	0.11	109.6	0.86	109.7	0.48	102.2	0.30
**Oligomycin A**	579-13-5	free base	ATPase	28.5	-2.45	93.9	0.29	110.6	0.52	13.8	-2.50
**Olmesartan Medoxomil**	144689-63-4	free base	RAAS	93.1	0.28	116.0	1.09	87.6	-0.46	72.0	-0.66
**Olopatadine HCl**	140462-76-6	hydrochloride	Histamine Receptor	84.0	-0.10	70.1	-0.58	135.0	1.55	88.5	-0.14
**Omecamtiv mecarbil (CK-1827452)**	873697-71-3	free base	ATPase	103.8	0.74	69.2	-0.61	123.3	1.05	128.6	1.13
**Omeprazole**	73590-58-6	free base	Autophagy,Proton Pump	84.7	-0.07	116.3	1.10	108.7	0.44	82.9	-0.32
**Ondansetron HCl**	99614-01-4	hydrochloride	5-HT Receptor	93.1	0.28	114.6	1.04	108.9	0.44	101.3	0.27
**ONX-0914 (PR-957)**	960374-59-8	free base	Proteasome	9.7	-3.25	3.4	-3.00			0.6	-2.92
**Oprozomib (ONX 0912)**	935888-69-0	free base	Proteasome	8.7	-3.29	2.8	-3.02	34.5	-2.72	5.4	-2.77
**Optovin**	348575-88-2	free base	Others	72.0	-0.61	113.1	0.98	98.8	0.01	113.8	0.66
**Org 27569**	868273-06-7	free base	Cannabinoid Receptor	130.9	1.88	104.9	0.69	122.0	1.00	94.0	0.04
**Orphenadrine Citrate**	4682-36-4	citrate	AChR	100.4	0.59	116.1	1.09	100.5	0.08	77.5	-0.48

**OSI-027**	936890-98-1	free base	mTOR	68.5	-0.76	69.3	-0.61	81.7	-0.71	75.6	-0.54
**OSI-420**	183320-51-6	hydrochloride	EGFR	106.9	0.87	55.3	-1.11	87.7	-0.46	109.1	0.52
**OSI-906 (Linsitinib)**	867160-71-2	free base	IGF-1R	45.9	-1.71	62.5	-0.85	58.1	-1.71	110.7	0.57
**OSI-930**	728033-96-3	free base	c-Kit,CSF-1R,VEGFR	101.9	0.65	71.5	-0.53	171.1	3.08	91.2	-0.05
**Ospemifene**	128607-22-7	free base	Estrogen/progestogen	81.9	-0.19	117.4	1.14	88.1	-0.44	109.6	0.53
**OSU-03012 (AR-12)**	742112-33-0	free base	PDK-1	226.7	5.94	109.3	0.85	96.0	-0.10	96.4	0.11
**Otilonium Bromide**	26095-59-0	free base	AChR	89.3	0.12	103.1	0.62	116.3	0.76	104.4	0.37
**OTX015**	202590-98-5	free base	BET	30.8	-2.36	47.9	-1.39	51.0	-2.02	12.2	-2.55
**Ouabain**	630-60-4	free base	Sodium Channel	41.0	-1.92	12.1	-2.69	25.7	-3.09	20.6	-2.29
**Oxcarbazepine**	28721-07-5	free base	Sodium Channel	80.7	-0.24	54.5	-1.15	126.6	1.20	99.9	0.23
**Oxiracetam**	62613-82-5	free base	Others	76.8	-0.41	78.1	-0.29	84.2	-0.61	103.3	0.33
**Oxybutynin chloride**	1508-65-2	chloride	AChR	89.3	0.12	119.2	1.21	98.6	0.01	119.7	0.85
**Oxymetazoline HCl**	2315-02-8	hydrochloride	Adrenergic Receptor	77.4	-0.38	49.8	-1.32	119.5	0.89	142.2	1.56
**Ozagrel**	82571-53-7	free base	Factor Xa	91.6	0.22	58.4	-1.00	148.5	2.13	137.9	1.43
**P22077**	1247819-59-5	free base	DUB	70.3	-0.68	73.4	-0.46			123.1	0.96
**P276-00**	920113-03-7	hydrochloride	CDK	24.7	-2.61	32.3	-1.95	70.2	-1.20	27.6	-2.07
**P5091 (P005091)**	882257-11-6	free base	DUB	72.3	-0.60	85.2	-0.03			93.8	0.03
**PAC-1**	315183-21-2	free base	Caspase	89.5	0.13	114.0	1.02	112.3	0.59	114.7	0.69
**Pacritinib (SB1518)**	937272-79-2	free base	JAK	75.0	-0.48	67.3	-0.68	103.5	0.21	91.4	-0.04
**Palbociclib (PD-0332991) HCl**	827022-32-2	hydrochloride	CDK	105.6	0.81	103.0	0.62	68.0	-1.29	54.2	-1.22
**Palomid 529 (P529)**	914913-88-5	free base	mTOR	90.8	0.19	79.7	-0.23	77.4	-0.90	106.5	0.43
**Pancuronium dibromide**	15500-66-0	bromide	AChR	97.2	0.46	111.3	0.92	122.7	1.03	118.4	0.81
**Panobinostat (LBH589)**	404950-80-7	free base	HDAC	2.4	-3.56	22.2	-2.32	13.8	-3.60	2.4	-2.87
**Paroxetine HCl**	78246-49-8	hydrochloride	AChR,5-HT Receptor	140.9	2.31	112.9	0.98	111.1	0.54	121.9	0.92
**Pazopanib**	444731-52-6	free base	PDGFR,c-Kit,VEGFR	122.9	1.55	116.5	1.11	157.6	2.51	117.6	0.78
**Pazopanib HCl (GW786034 HCl)**	635702-64-6	hydrochloride	VEGFR,PDGFR,c-Kit	83.1	-0.14	113.3	0.99	122.8	1.03	110.1	0.55
**PCI-24781 (Abexinostat)**	783355-60-2	free base	HDAC	2.6	-3.55	21.5	-2.35	19.5	-3.36	2.5	-2.86
**PCI-34051**	950762-95-5	free base	HDAC	98.1	0.49	113.3	0.99	106.5	0.34	95.4	0.08
**PD 151746**	179461-52-0	free base	Cysteine Protease	73.6	-0.54	107.1	0.77	91.6	-0.29	125.0	1.02

**PD0325901**	391210-10-9	free base	MEK	84.9	-0.06	30.8	-2.01	50.3	-2.05	109.6	0.53
**PD123319**	130663-39-7	free base	RAAS	82.7	-0.16	61.6	-0.89			95.2	0.08
**PD128907 HCl**	112960-16-4	hydrochloride	Dopamine Receptor	94.9	0.36	106.9	0.76	81.2	-0.73	83.5	-0.29
**PD168393**	194423-15-9	free base	EGFR	93.0	0.28	63.4	-0.82	69.1	-1.25	90.1	-0.09
**PD168393**	194423-15-9	free base	EGFR	72.4	-0.59	102.6	0.60	74.5	-1.02	98.0	0.16
**PD173074**	219580-11-7	free base	VEGFR,FGFR	96.3	0.42	103.3	0.63	97.1	-0.06	61.4	-1.00
**PD173955**	260415-63-2	free base	BCR-ABL	64.5	-0.93	101.5	0.56	100.0	0.06	54.7	-1.21
**PD184352 (CI-1040)**	212631-79-3	free base	MEK	93.8	0.31	57.3	-1.04	44.2	-2.31	91.5	-0.04
**PD318088**	391210-00-7	free base	MEK	87.7	0.05	30.3	-2.02	51.5	-1.99	82.7	-0.32
**PD98059**	167869-21-8	free base	MEK	77.5	-0.38	109.0	0.84	122.1	1.01	75.3	-0.55
**Pefloxacin Mesylate Dihydrate**	149676-40-4	Mesylate	Topoisomerase	89.2	0.12	85.3	-0.03	82.4	-0.68	117.8	0.79
**Pelitinib (EKB-569)**	257933-82-7	free base	EGFR	74.4	-0.51	54.7	-1.14	112.2	0.58	75.7	-0.54
**Pergolide mesylate**	66104-23-2	mesylate	Dopamine Receptor	92.2	0.25	118.4	1.18	101.8	0.14	125.3	1.03
**PF-00562271**	939791-38-5	benzenesulfon ic	FAK	62.6	-1.01	107.3	0.78	101.5	0.13	89.1	-0.12
**PF-04217903**	956905-27-4	free base	c-Met	110.8	1.03	115.1	1.06	87.2	-0.48	121.9	0.92
**PF-04620110**	1109276-89-2	free base	Transferase	75.3	-0.47	97.2	0.41			105.2	0.39
**PF-04691502**	1013101-36-4	free base	Akt,mTOR,PI3K	38.5	-2.03	36.7	-1.79	68.9	-1.25	48.8	-1.39
**PF-04929113 (SNX-5422)**	908115-27-5	free base	HSP (e.g. HSP90)	50.3	-1.53	29.2	-2.07	40.3	-2.47	52.3	-1.28
**PF-2545920**	1292799-56-4	free base	PDE	112.9	1.12	62.2	-0.87	118.5	0.85	116.2	0.74
**PF-3758309**	898044-15-0	free base	PAK	66.5	-0.84	36.4	-1.80			16.8	-2.41
**PF-3845**	1196109-52-0	free base	FAAH	89.2	0.12	111.8	0.94	131.5	1.40	100.0	0.23
**PF-4708671**	1255517-76-0	free base	S6 Kinase	100.3	0.59	100.9	0.54	108.1	0.41	106.9	0.45
**PF-477736**	952021-60-2	free base	Chk	60.4	-1.10	19.2	-2.43	106.3	0.33	30.6	-1.97
**PF-4981517**	1390637-82-7	free base	P450 (e.g. CYP17)	107.0	0.87	98.4	0.45	100.5	0.09	96.2	0.11
**PF-5274857**	1373615-35-0	free base	Hedgehog/Smoothened	116.5	1.28	98.4	0.45	111.0	0.53	115.6	0.72
**PF-543**	1415562-82-1	free base	S1P Receptor	88.2	0.08	77.4	-0.31			121.0	0.89
**PF-562271**	717907-75-0	free base	FAK	70.1	-0.69	51.6	-1.25	115.7	0.73	92.0	-0.03
**PF-562271 HCl**	N/A	hydrochloride	FAK	73.1	-0.56	77.8	-0.30	83.3	-0.64	87.5	-0.17

**PF-573228**	869288-64-2	free base	FAK	105.0	0.79	107.6	0.78	104.0	0.24	111.4	0.59
**PFI-1 (PF-6405761)**	1403764-72-6	free base	Epigenetic Reader Domain	87.1	0.03	94.9	0.32	87.7	-0.46	60.5	-1.02
**PFK15**	4382-63-2	free base	Others	80.9	-0.23	67.5	-0.67	77.2	-0.90	109.2	0.52
**PH-797804**	586379-66-0	free base	p38 MAPK	81.2	-0.22	114.3	1.03	118.8	0.86	91.8	-0.03
**PHA-665752**	477575-56-7	free base	c-Met	92.9	0.28	115.7	1.08	115.7	0.73	109.7	0.53
**PHA-680632**	398493-79-3	free base	Aurora Kinase	71.6	-0.63	54.6	-1.14	65.5	-1.40	53.7	-1.24
**PHA-767491**	845714-00-3	free base	CDK	97.6	0.48	72.8	-0.48	107.9	0.40	114.4	0.68
**PHA-793887**	718630-59-2	free base	CDK	67.1	-0.82	91.9	0.21	63.9	-1.47	18.0	-2.37
**Phenacetin**	62-44-2	free base	COX	113.7	1.16	65.3	-0.75	130.6	1.37	123.2	0.96
**Phenformin HCl**	834-28-6	hydrochloride	AMPK	81.7	-0.20	115.5	1.07	126.1	1.17	122.1	0.93
**Phenoxybenzamine HCl**	63-92-3	hydrochloride	Adrenergic Receptor	109.5	0.98	115.8	1.08	118.3	0.84	120.9	0.89
**Phentolamine Mesylate**	65-28-1	mesylate	Adrenergic Receptor	96.3	0.42	115.9	1.09	103.0	0.19	107.0	0.45
**Phenylephrine HCl**	61-76-7	hydrochloride	Adrenergic Receptor	94.4	0.34	49.6	-1.32	118.8	0.86	141.8	1.55
**Phenytoin**	57-41-0	free base	Sodium Channel	80.9	-0.23	58.1	-1.01	102.6	0.17	139.6	1.48
**Phenytoin sodium**	630-93-3	sodium	Sodium Channel	83.2	-0.14	115.7	1.08	105.9	0.32	124.4	1.00
**Phosphoramidon Disodium Salt**	164204-38-0	Salt	Others	78.0	-0.36	73.6	-0.45	126.1	1.17	74.1	-0.59
**PHT-427**	1191951-57-1	free base	PDK-1,Akt	87.7	0.05	59.1	-0.98	111.2	0.54	97.7	0.15
**PI-103**	371935-74-9	free base	PI3K,Autophagy,DNA-PK,mTOR	38.9	-2.01	49.0	-1.35	58.0	-1.72	55.3	-1.19
**PI-1840**	1401223-22-0	free base	Proteasome	90.2	0.16	107.1	0.77	101.3	0.12	104.2	0.36
**Piceatannol**	10083-24-6	free base	Syk	102.6	0.69	64.9	-0.77	127.2	1.22	120.4	0.87
**Pifithrin-α (PFTα)**	63208-82-2	HBr	p53,Autophagy	100.5	0.60	77.2	-0.32	107.4	0.38	110.0	0.54
**Pifithrin-μ**	64984-31-2	free base	p53	106.0	0.83	74.3	-0.43	128.3	1.27	108.0	0.48
**PIK-293**	900185-01-5	free base	PI3K	108.7	0.94	62.9	-0.84	88.6	-0.42	110.7	0.57
**PIK-294**	900185-02-6	free base	PI3K	111.6	1.07	57.6	-1.03	79.7	-0.80	82.4	-0.33
**PIK-75**	372196-77-5	hydrochloride	PI3K,DNA-PK	49.3	-1.57	36.8	-1.79	31.7	-2.84	11.9	-2.56
**PIK-93**	593960-11-3	free base	PI3K	64.8	-0.91	73.7	-0.45	113.9	0.66	85.4	-0.23
**Pimasertib (AS-703026)**	1236699-92-5	free base	MEK	99.7	0.56	29.6	-2.05	57.1	-1.76	76.3	-0.52
**Pimobendan**	74150-27-9	free base	PDE	77.7	-0.37	113.1	0.99	105.7	0.31	79.0	-0.44

**Pioglitazone HCl**	112529-15-4	hydrochloride	P450 (e.g. CYP17)	100.3	0.59	116.9	1.12	99.9	0.06	123.1	0.96
**Pirarubicin**	72496-41-4	free base	Topoisomerase	8.8	-3.29	48.7	-1.35	71.3	-1.15	4.4	-2.80
**Pirfenidone**	53179-13-8	free base	TGF-beta/Smad	82.9	-0.15	53.4	-1.18	100.2	0.07	109.0	0.51
**Piroxicam**	36322-90-4	free base	COX	107.7	0.90	114.1	1.02	108.3	0.42	73.1	-0.62
**PJ34**	344458-19-1	hydrochloride	PARP	81.9	-0.19	63.5	-0.82	101.6	0.13	83.3	-0.30
**PJ34 HCl**	344458-15-7	Salt	PARP	77.9	-0.36	67.5	-0.67	93.3	-0.22	119.9	0.86
**PluriSIn #1 (NSC 14613)**	91396-88-2	free base	Dehydrogenase	73.1	-0.56	112.1	0.95	94.7	-0.16	134.5	1.32
**PLX-4720**	918505-84-7	free base	Raf	121.0	1.46	116.8	1.12	190.3	3.90	69.4	-0.74
**PNU-120596**	501925-31-1	free base	AChR	75.6	-0.46	112.2	0.95	99.9	0.06	127.9	1.11
**Pomalidomide**	19171-19-8	free base	TNF-alpha	86.4	0.00	114.3	1.03	111.2	0.54	93.5	0.02
**Ponatinib (AP24534)**	943319-70-8	free base	PDGFR,FGFR,VEGFR,Bcr-Abl	73.9	-0.53	102.0	0.58	72.5	-1.10	52.8	-1.27
**PP1**	172889-26-8	free base	Src	77.0	-0.40	116.4	1.11	105.4	0.30	84.3	-0.27
**PP121**	1092788-83-4	free base	DNA-PK,PDGFR,mTOR	60.4	-1.10	52.6	-1.21	126.3	1.18	72.9	-0.63
**PP2**	172889-27-9	free base	Src	98.5	0.51	116.7	1.11	83.3	-0.65	119.0	0.83
**PP242**	1092351-67-1	free base	mTOR,Autophagy	70.9	-0.66	50.6	-1.29	79.4	-0.81	63.2	-0.94
**PQ 401**	196868-63-0	free base	IGF-1R	71.8	-0.62	56.8	-1.06	102.2	0.16	105.7	0.41
**PR-619**	2645-32-1	free base	DUB	67.4	-0.80	53.7	-1.17			88.1	-0.15
**Pracinostat (SB939)**	929016-96-6	free base	HDAC	11.1	-3.19	49.3	-1.33	53.2	-1.92	3.5	-2.83
**Pralatrexate**	146464-95-1	free base	DHFR	116.2	1.26	91.5	0.20	93.6	-0.21	70.1	-0.72
**Pramipexole**	104632-26-0	free base	Dopamine Receptor	111.0	1.04	83.9	-0.08	118.2	0.84	109.4	0.52
**Prasugrel**	150322-43-3	free base	P2 Receptor	95.7	0.39	112.1	0.95	127.3	1.23	105.0	0.39
**Pravastatin sodium**	81131-70-6	free base	HMG-CoA Reductase	117.3	1.31	113.2	0.99	113.8	0.65	129.8	1.17
**Pregnenolone**	145-13-1	free base	Estrogen/progestogen Receptor	128.2	1.77	114.0	1.02	111.2	0.54	127.3	1.09
**Pritelivir (BAY 57-1293)**	348086-71-5	free base	Others	87.5	0.05	57.3	-1.04	101.3	0.12	85.0	-0.25
**Procainamide HCl**	614-39-1	Salt	Sodium Channel	87.1	0.03	84.8	-0.04	84.0	-0.62	110.9	0.57
**Procaine HCl**	51-05-8	hydrochloride	Sodium Channel	96.9	0.44	85.5	-0.02	108.3	0.42	125.1	1.02
**Promethazine HCl**	58-33-3	Salt	Histamine Receptor	93.1	0.28	73.9	-0.44	86.7	-0.50	105.8	0.41
**Propafenone HCl**	34183-22-7	free base	Sodium Channel	89.8	0.14	117.4	1.14	112.1	0.58	105.2	0.39

**Proparacaine HCl**	5875-06-9	hydrochloride	Sodium Channel	98.3	0.50	115.3	1.06	116.0	0.75	93.6	0.03
**Propranolol HCl**	318-98-9	hydrochloride	Adrenergic Receptor	139.4	2.25	113.9	1.01	98.1	-0.02	110.4	0.56
**PRT062607 (P505-15, BIIB057) HCl**	1370261-97-4	hydrochloride	Syk	65.4	-0.89	61.7	-0.88	105.2	0.28	85.6	-0.23
**Prucalopride**	179474-81-8	free base	5-HT Receptor	101.9	0.66	81.6	-0.16	103.7	0.22	100.4	0.24
**PRX-08066 Maleic acid**	866206-55-5	maleate	5-HT Receptor	92.5	0.26	65.3	-0.75	106.3	0.33	78.6	-0.45
**PTC-209**	315704-66-6	free base	BMI	27.3	-2.50	94.5	0.31	84.0	-0.61	37.6	-1.75
**PTC-209 HBr**	1217022-63-3	free base	Others	25.0	-2.60	50.7	-1.28	105.1	0.28	37.1	-1.76
**PU-H71**	873436-91-0	free base	HSP (e.g. HSP90)	41.3	-1.91	42.7	-1.57	41.3	-2.43	50.5	-1.34
**Purmorphamine**	483367-10-8	free base	Hedgehog/Smoothened	104.3	0.76	66.2	-0.72	113.9	0.65	123.4	0.97
**Puromycin 2HCl**	58-58-2	Salt	Others	15.3	-3.01	74.1	-0.43	63.2	-1.50	1.4	-2.90
**PYR-41**	418805-02-4	free base	E1 Activating	68.7	-0.75	60.2	-0.94			139.4	1.48
**Pyridostigmine Bromide**	101-26-8	bromide	AChR	126.4	1.69	110.3	0.88	114.6	0.68	95.7	0.09
**Pyrimethamine**	58-14-0	free base	DHFR	95.7	0.39	116.5	1.11	107.1	0.37	129.5	1.16
**QNZ (EVP4593)**	545380-34-5	free base	TNF-alpha,NF-κB	57.3	-1.23	49.0	-1.34	95.3	-0.13	82.6	-0.32
**Quercetin**	117-39-5	free base	PKC,Src,PI3K,Sirtuin	130.6	1.87	99.3	0.48	85.8	-0.54	80.4	-0.39
**Quetiapine Fumarate**	111974-72-2	fumarate	Dopamine Receptor	113.6	1.15	114.0	1.02	108.9	0.44	115.2	0.71
**Quinapril HCl**	82586-55-8	hydrochloride	RAAS	94.5	0.34	56.2	-1.08	129.1	1.30	110.4	0.56
**Quisinostat (JNJ-26481585)**	875320-29-9	free base	HDAC	20.3	-2.80	20.6	-2.38	16.4	-3.49	2.5	-2.86
**Quizartinib (AC220)**	950769-58-1	free base	FLT3	77.3	-0.38	93.8	0.28	109.6	0.47	87.1	-0.18
**R406**	841290-81-1	besylate	Syk,FLT3	89.3	0.12	112.9	0.98	86.8	-0.50	78.6	-0.45
**R406 (free base)**	841290-80-0	free base	Syk	87.3	0.04	114.5	1.03	106.4	0.34	86.1	-0.21
**R547**	741713-40-6	free base	CDK	56.2	-1.28	81.1	-0.18	58.0	-1.72	28.5	-2.04
**Racecadotril**	81110-73-8	free base	Opioid Receptor	88.6	0.09	56.2	-1.08	145.5	2.00	99.0	0.20
**RAF265 (CHIR-265)**	927880-90-8	free base	VEGFR,Raf	92.4	0.25	59.4	-0.97	82.6	-0.67	59.0	-1.07
**Raloxifene HCl**	82640-04-8	hydrochloride	Estrogen/progestogen Receptor	102.4	0.68	109.4	0.85	127.7	1.24	73.0	-0.63
**Raltegravir (MK-0518)**	518048-05-0	free base	Integrase	96.4	0.42	54.2	-1.16	118.0	0.83	151.5	1.86
**Ramelteon**	196597-26-9	free base	MT Receptor	111.3	1.05	94.3	0.30	110.3	0.50	95.5	0.09
**Ramipril**	87333-19-5	free base	RAAS	104.7	0.78	117.1	1.13	103.2	0.20	96.9	0.13

**Ranitidine**	66357-59-3	hydrochloride	Histamine Receptor	105.3	0.80	116.5	1.11	108.1	0.41	97.1	0.14
**Ranolazine 2HCl**	95635-56-6	dihydrochlorid e	Calcium Channel	80.2	-0.26	78.0	-0.29	102.1	0.15	98.4	0.18
**Rapamycin (Sirolimus)**	53123-88-9	free base	Autophagy,mTOR	70.9	-0.66	59.4	-0.97	51.7	-1.99	81.0	-0.37
**Rasagiline Mesylate**	161735-79-1	mesylate	MAO	96.9	0.44	116.1	1.09	93.5	-0.21	130.0	1.18
**Refametinib (RDEA119, Bay 86-9766)**	923032-37-5	free base	MEK			33.3	-1.92	38.4	-2.55	104.2	0.36
**Regorafenib (BAY 73-4506)**	755037-03-7	free base	c-RET,VEGFR	93.4	0.30	79.8	-0.22	61.4	-1.57	66.4	-0.84
**Repaglinide**	135062-02-1	free base	Potassium Channel	76.7	-0.41	71.6	-0.52	86.1	-0.53	93.0	0.01
**RepSox**	446859-33-2	free base	TGF-beta/Smad	78.1	-0.35	54.5	-1.14			104.1	0.36
**Resminostat**	864814-88-0	free base	HDAC	56.4	-1.27	112.6	0.97	128.5	1.28	35.7	-1.81
**Resveratrol**	501-36-0	free base	Sirtuin,Autophagy	78.3	-0.34	115.4	1.07	100.1	0.07	97.5	0.15
**RG108**	48208-26-0	free base	Transferase,DNA Methyltransferase	91.1	0.20	59.6	-0.96	83.0	-0.66	112.4	0.62
**RG2833 (RGFP109)**	1215493-56-3	free base	HDAC	75.8	-0.45	101.3	0.56	129.3	1.31	98.0	0.17
**RGFP966**	1396841-57-8	free base	HDAC	91.0	0.19	93.3	0.27			120.4	0.87
**Ridaforolimus (Deforolimus, MK-8669)**	572924-54-0	free base	mTOR	66.0	-0.86	67.5	-0.67	72.7	-1.09	63.4	-0.93
**Rigosertib (ON-01910)**	1225497-78-8	sodium	PLK	83.1	-0.14	52.7	-1.21	123.5	1.06	90.7	-0.07
**Rilpivirine**	500287-72-9	free base	Reverse Transcriptase	73.5	-0.55	82.7	-0.12	72.7	-1.10	71.1	-0.69
**Riluzole**	1744-22-5	free base	Sodium Channel	88.8	0.10	114.4	1.03	101.8	0.14	89.0	-0.12
**Rimonabant**	168273-06-1	free base	Cannabinoid Receptor	135.9	2.10	109.1	0.84	117.0	0.79	93.2	0.01
**Risperidone**	106266-06-2	free base	5-HT Receptor	82.3	-0.17	114.2	1.03	104.3	0.25	77.7	-0.48
**RITA (NSC 652287)**	213261-59-7	free base	E3 Ligase ,p53	88.5	0.09	83.6	-0.09	127.7	1.24	52.3	-1.28
**Ritodrine HCl**	23239-51-2	hydrochloride	Adrenergic Receptor	92.7	0.27	115.9	1.09	113.8	0.65	122.1	0.93
**Ritonavir**	155213-67-5	free base	HIV Protease	117.1	1.30	116.3	1.10	122.6	1.02	68.0	-0.79
**Rivaroxaban**	366789-02-8	free base	Factor Xa	139.0	2.23	107.3	0.77	109.1	0.45	113.3	0.65
**Rivastigmine Tartrate**	129101-54-8	tartrate	AChR	96.4	0.42	116.3	1.10	75.1	-0.99	126.2	1.06
**Rizatriptan Benzoate**	145202-66-0	benzoate	5-HT Receptor	110.7	1.03	114.7	1.04	106.7	0.35	91.7	-0.03
**RKI-1447**	1342278-01-6	free base	ROCK	70.9	-0.65	86.1	0.00			124.5	1.00
**Ro 31-8220 Mesylate**	138489-18-6	mesylate	PKC	49.6	-1.56	74.8	-0.41			40.4	-1.66
**Ro3280**	1062243-51-9	free base	PLK	54.0	-1.37	58.6	-1.00			25.0	-2.15

**RO4929097**	847925-91-1	free base	Gamma-secretase	106.8	0.86	115.0	1.05	101.6	0.13	76.1	-0.53
**Rocilinostat (ACY-1215)**	1316214-52-4	free base	HDAC	76.6	-0.41	67.6	-0.67	122.9	1.04	111.6	0.59
**Rocuronium Bromide**	119302-91-9	bromide	AChR	74.2	-0.52	115.8	1.08	91.2	-0.31	84.5	-0.26
**Rofecoxib**	162011-90-7	free base	COX	97.4	0.47	120.1	1.24	108.1	0.41	116.0	0.74
**Roflumilast**	162401-32-3	free base	PDE	125.2	1.64	99.4	0.49	96.9	-0.07	74.7	-0.57
**Rolipram**	61413-54-5	free base	PDE	91.9	0.23	63.3	-0.83	115.0	0.70	101.5	0.27
**Roscovitine (Seliciclib,CYC202)**	186692-46-6	free base	CDK	95.5	0.39	110.6	0.90	111.1	0.54	63.5	-0.93
**Rosiglitazone**	122320-73-4	free base	PPAR	79.3	-0.30	72.3	-0.50	104.0	0.23	117.9	0.80
**Rosuvastatin Calcium**	147098-20-2	free base	RAAS	89.9	0.15	74.6	-0.42	91.0	-0.32	96.9	0.13
**Rotigotine**	99755-59-6	free base	Dopamine Receptor	106.8	0.87	116.8	1.12	89.2	-0.39	94.8	0.06
**Rotundine**	483-14-7	free base	Dopamine Receptor	107.9	0.91	111.0	0.91	80.7	-0.75	111.8	0.60
**Roxatidine Acetate HCl**	93793-83-0	hydrochloride	Histamine Receptor	97.3	0.46	115.7	1.08	118.3	0.84	160.5	2.14
**RS-127445**	199864-87-3	free base	5-HT Receptor	75.6	-0.46	47.6	-1.40	114.2	0.67	137.9	1.43
**Rucaparib (AG-014699,PF-01367338)**	459868-92-9	phosphate	PARP			79.5	-0.24	74.0	-1.04	99.5	0.21
**Rufinamide**	106308-44-5	free base	Sodium Channel	99.0	0.53	81.0	-0.18	83.3	-0.64	90.9	-0.06
**Rupatadine Fumarate**	182349-12-8	fumarate	Histamine Receptor	116.7	1.28	103.1	0.62	113.4	0.64	130.4	1.19
**Ruxolitinib (INCB018424)**	941678-49-5	free base	JAK	99.4	0.55	115.0	1.05	105.6	0.30	109.6	0.53
**RVX-208**	1044870-39-4	free base	Epigenetic Reader Domain	134.1	2.02	93.5	0.27	100.4	0.08	88.3	-0.14
**S- (+)-Rolipram**	85416-73-5	free base	PDE	121.6	1.49	96.0	0.36	92.2	-0.27	81.7	-0.35
**S3I-201**	501919-59-1	free base	STAT	98.3	0.50	84.7	-0.05	119.5	0.89	85.9	-0.22
**Sabutoclax**	1228108-65-3	free base	Bcl-2	85.2	-0.05	88.6	0.09	100.8	0.10	72.1	-0.65
**Safinamide Mesylate**	202825-46-5	mesylate	MAO	110.9	1.04	112.4	0.96	107.0	0.36	68.0	-0.79
**Sal003**	1164470-53-4	free base	ELF2	132.8	1.96	99.5	0.49	102.3	0.16	107.5	0.47
**SANT-1**	304909-07-7	free base	Hedgehog/Smoothened	93.8	0.31	105.1	0.69			90.9	-0.06
**Santacruzamate A (CAY10683)**	1477949-42-0	free base	HDAC	60.3	-1.11	91.7	0.21	82.0	-0.70	75.8	-0.54
**SAR131675**	1433953-83-3	free base	VEGFR	81.0	-0.23	121.6	1.29	116.0	0.74	114.4	0.68
**SAR245409 (XL765)**	1349796-36-6	free base	PI3K,mTOR	89.7	0.14	56.2	-1.08	111.1	0.54	97.1	0.13
**Saracatinib (AZD0530)**	379231-04-6	free base	Src,Bcr-Abl	113.4	1.14	111.0	0.91	91.9	-0.28	108.9	0.51
**Saxagliptin**	361442-04-8	free base	DPP-4	92.3	0.25	115.5	1.07	106.3	0.33	99.2	0.20

**SB202190 (FHPI)**	152121-30-7	free base	p38 MAPK			72.0	-0.51	92.1	-0.27	79.0	-0.44
**SB203580**	152121-47-6	free base	p38 MAPK	78.1	-0.35	111.3	0.92	118.3	0.84	78.0	-0.47
**SB216763**	280744-09-4	free base	GSK-3	92.3	0.25	113.2	0.99	93.0	-0.23	85.1	-0.25
**SB269970 HCl**	261901-57-9	hydrochloride	5-HT Receptor	74.4	-0.51	115.0	1.05	117.6	0.81	109.2	0.52
**SB271046**	209481-20-9	free base	5-HT Receptor	93.6	0.30	114.9	1.05	90.9	-0.32	127.7	1.11
**SB-3CT**	292605-14-2	free base	MMP	82.8	-0.15	77.2	-0.32	93.4	-0.22	72.6	-0.64
**SB408124**	288150-92-5	free base	OX Receptor	119.2	1.39	56.8	-1.06	109.6	0.47	96.3	0.11
**SB415286**	264218-23-7	free base	GSK-3	87.3	0.04	113.2	0.99	113.1	0.62	123.9	0.98
**SB431542**	301836-41-9	free base	TGF-beta/Smad	87.8	0.06	91.6	0.20	84.5	-0.59	107.0	0.45
**SB505124**	694433-59-5	free base	TGF-beta/Smad	102.6	0.69	59.5	-0.96	95.1	-0.14	92.9	0.00
**SB525334**	356559-20-1	free base	TGF-beta/Smad	101.4	0.64	105.6	0.71	116.6	0.77	75.6	-0.55
**SB590885**	405554-55-4	free base	Raf	122.0	1.51	74.4	-0.42	108.6	0.43	131.4	1.22
**SB705498**	501951-42-4	free base	TRPV	76.1	-0.44	111.6	0.93	106.7	0.35	98.3	0.17
**SB742457**	607742-69-8	free base	5-HT Receptor	90.1	0.15	78.7	-0.27	126.8	1.20	111.8	0.60
**SB743921**	940929-33-9	hydrochloride	Kinesin	72.4	-0.59	54.8	-1.14	41.3	-2.43	55.5	-1.18
**SC-514**	354812-17-2	free base	IκB/IKK	87.0	0.03	69.4	-0.60	84.2	-0.61	109.8	0.54
**SC75741**	913822-46-5	free base	NF-κB	67.6	-0.80	74.9	-0.40	110.1	0.50	71.1	-0.69
**Scopine**	498-45-3	free base	Adrenergic Receptor	81.2	-0.22	116.7	1.12	120.5	0.94	147.5	1.73
**Scopolamine HBr**	114-49-8	hydrobromide	AChR	79.9	-0.28	116.6	1.11	123.3	1.05	97.7	0.15
**Scriptaid**	287383-59-9	free base	HDAC	71.5	-0.63	64.7	-0.77	102.2	0.16	53.7	-1.24
**Selumetinib (AZD6244)**	606143-52-6	free base	MEK	121.5	1.48	106.5	0.75	48.4	-2.13	83.0	-0.31
**Semagacestat (LY450139)**	425386-60-3	free base	Gamma-secretase	87.0	0.03	115.7	1.08	122.4	1.01	92.4	-0.01
**Semaxanib (SU5416)**	194413-58-6	free base	VEGFR	83.4	-0.13	54.5	-1.15	119.4	0.89	100.8	0.25
**Sertraline HCl**	79559-97-0	hydrochloride	5-HT Receptor	106.9	0.87	59.7	-0.96	98.3	-0.01	128.2	1.12
**SF1670**	345630-40-2	free base	Others	82.6	-0.16	90.3	0.16	114.3	0.67	95.1	0.07
**SGC 0946**	N/A	free base	Histone Methyltransferase	91.6	0.22	88.0	0.07	90.6	-0.33	110.4	0.56
**SGC-CBP30**	N/A	free base	Epigenetic Reader Domain	75.5	-0.46	101.2	0.55			104.1	0.36
**SGI-1027**	1020149-73-8	free base	DNA Methyltransferase	81.2	-0.22	98.0	0.44	103.1	0.20	5.1	-2.78
**SGI-1776 free base**	1025065-69-3	free base	Pim	83.5	-0.12	75.3	-0.39	83.7	-0.63	93.1	0.01

**SGX-523**	1022150-57-7	free base	c-Met	107.2	0.88	100.8	0.54	74.8	-1.01	96.2	0.11
**SH-4-54**	1456632-40-8	free base	STAT	81.6	-0.20	57.0	-1.05	104.9	0.27	99.1	0.20
**Sildenafil Citrate**	171599-83-0	citrate	PDE	102.1	0.66	115.8	1.08	101.5	0.13	83.6	-0.29
**Silodosin**	160970-54-7	free base	Adrenergic Receptor	84.5	-0.08	114.0	1.02	113.5	0.64	96.6	0.12
**Sirtinol**	410536-97-9	free base	Sirtuin	88.1	0.07	114.6	1.04	110.0	0.49	80.5	-0.39
**Sitagliptin phosphate monohydrate**	654671-77-9	phosphate	DPP-4	93.4	0.30	94.3	0.30	100.6	0.09	117.9	0.80
**Sitaxentan sodium**	210421-74-2	free base	Endothelin Receptor	99.6	0.56	89.2	0.12	113.8	0.65	96.0	0.10
**Skepinone-L**	1221485-83-1	free base	p38 MAPK	66.3	-0.85	52.3	-1.22			94.0	0.04
**SKI II**	312636-16-1	free base	S1P Receptor	80.8	-0.24	56.0	-1.09			126.9	1.08
**SKLB1002**	1225451-84-2	free base	VEGFR	84.4	-0.08	57.3	-1.04			103.9	0.35
**SL-327**	305350-87-2	free base	MEK	75.2	-0.47	104.4	0.67	94.9	-0.15	108.2	0.49
**SMI-4a**	438190-29-5	free base	Pim	76.0	-0.44	72.1	-0.51	101.5	0.13	84.0	-0.28
**SN-38**	86639-52-3	free base	Topoisomerase	3.5	-3.51	32.3	-1.95	61.7	-1.56	4.2	-2.81
**SNS-032 (BMS-387032)**	345627-80-7	free base	CDK	52.6	-1.43	36.6	-1.80	27.8	-3.00	11.0	-2.59
**SNS-314 Mesylate**	1146618-41-8	mesylate	Aurora Kinase	58.0	-1.20	65.3	-0.75	75.0	-1.00	69.9	-0.73
**SNX-2112 (PF-04928473)**	908112-43-6	free base	HSP (e.g. HSP90)	51.3	-1.49	36.8	-1.79	40.4	-2.47	50.2	-1.35
**Sodium 4-Aminosalicylate**	6018-19-5	sodium	NF-κB	96.3	0.42	118.5	1.18	106.0	0.32	132.6	1.26
**Sodium Danshensu**	67920-52-9	sodium	Others,P450 (e.g. CYP17)	110.0	1.00	77.4	-0.31	105.2	0.29	106.8	0.44
**Sodium Phenylbutyrate**	1716-12-7	free base	HDAC	91.7	0.22	98.2	0.44	83.7	-0.63	89.1	-0.12
**Sofosbuvir (PSI-7977, GS-7977)**	1190307-88-0	free base	DNA/RNA Synthesis	104.2	0.75	104.5	0.67	101.8	0.14	90.5	-0.07
**Solifenacin succinate**	242478-38-2	succinate	AChR	81.4	-0.21	80.7	-0.19	113.0	0.62	89.4	-0.11
**Sorafenib**	284461-73-0	free base	Raf	84.4	-0.09	116.2	1.10	74.5	-1.02	97.2	0.14
**Sorafenib Tosylate**	475207-59-1	tosylate	PDGFR,Raf,VEGFR	97.8	0.48	113.7	1.01	77.3	-0.90	91.1	-0.05
**Sotalol**	959-24-0	hydrochloride	Adrenergic Receptor	89.0	0.11	116.2	1.10	125.1	1.13	137.5	1.41
**Sotrastaurin**	425637-18-9	free base	PKC	87.2	0.03	104.1	0.66	92.7	-0.25	72.7	-0.64
**SP600125**	129-56-6	free base	JNK	86.8	0.02	115.0	1.05	100.0	0.07	94.0	0.04
**Spironolactone**	52-01-7	free base	Androgen Receptor	95.1	0.37	57.3	-1.04	99.1	0.03	122.7	0.95
**SRT1720**	1001645-58-4	hydrochloride	Sirtuin	89.4	0.13	113.2	0.99	86.0	-0.53	62.1	-0.97
**S-Ruxolitinib (INCB018424)**	941685-37-6	free base	JAK	74.3	-0.51	54.9	-1.13	83.6	-0.63	99.7	0.22

**SSR128129E**	848318-25-2	free base	FGFR	71.2	-0.64	61.8	-0.88			113.0	0.64
**Stattic**	19983-44-9	free base	STAT	92.2	0.24	117.1	1.13	83.4	-0.64	93.0	0.01
**Stavudine (d4T)**	3056-17-5	free base	Reverse Transcriptase	88.9	0.10	49.7	-1.32	106.8	0.35	78.5	-0.45
**SU11274**	658084-23-2	free base	c-Met	80.1	-0.27	92.9	0.25	71.8	-1.13	127.0	1.08
**Sulfacetamide Sodium**	127-56-0	sodium	Autophagy	103.6	0.73	116.1	1.09	109.1	0.45	97.7	0.16
**Sumatriptan Succinate**	103628-48-4	succinate	5-HT Receptor	91.5	0.22	99.6	0.49	97.9	-0.02	76.0	-0.53
**Sunitinib Malate**	341031-54-7	malate	VEGFR,PDGFR,c-Kit	53.0	-1.41	109.8	0.86	83.6	-0.63	139.8	1.49
**Suvorexant (MK-4305)**	1030377-33-3	free base	OX Receptor	80.4	-0.25	117.0	1.13	106.8	0.35	101.4	0.27
**Synephrine**	94-07-5	free base	Adrenergic Receptor	93.3	0.29	80.2	-0.21	91.7	-0.29	124.8	1.01
**Synephrine HCl**	5985-28-4	hydrochloride	Adrenergic Receptor	108.9	0.95	68.8	-0.63	77.8	-0.88	111.6	0.60
**T0070907**	313516-66-4	free base	PPAR	88.9	0.10	100.1	0.51	125.5	1.15	116.8	0.76
**T0901317**	293754-55-9	free base	Liver X Receptor	98.1	0.50	62.6	-0.85	88.4	-0.43	120.2	0.87
**Tadalafil**	171596-29-5	free base	PDE	94.7	0.35	115.7	1.08	110.3	0.50	98.1	0.17
**TAE226 (NVP-TAE226)**	761437-28-9	free base	FAK	32.9	-2.27	38.0	-1.74	61.9	-1.55	77.6	-0.48
**TAK-285**	871026-44-7	free base	EGFR,HER2	106.7	0.86	72.8	-0.48	118.5	0.85	107.9	0.48
**TAK-438**	1260141-27-2	fumarate	Potassium Channel	66.9	-0.83	71.5	-0.53	107.4	0.38	111.4	0.59
**TAK-632**	1228591-30-7	free base	Raf	81.3	-0.22	98.2	0.44	60.4	-1.62	108.5	0.50
**TAK-700 (Orteronel)**	426219-18-3	free base	P450 (e.g. CYP17)	97.5	0.47	103.2	0.62	111.0	0.53	67.4	-0.80
**TAK-715**	303162-79-0	free base	p38 MAPK	80.6	-0.25	112.9	0.98	92.1	-0.27	93.7	0.03
**TAK-733**	1035555-63-5	free base	MEK	86.1	-0.01	22.8	-2.30	65.8	-1.39	87.2	-0.18
**TAK-875**	1374598-80-7	hydrate	GPR	89.1	0.11	113.5	1.00	104.5	0.26	116.7	0.76
**TAK-901**	934541-31-8	free base	Aurora Kinase	40.8	-1.93	86.4	0.01	43.3	-2.34	50.8	-1.33
**TAME**	901-47-3	free base	E3 Ligase ,APC	111.1	1.05	66.3	-0.72	86.0	-0.53	128.7	1.14
**Tamoxifen Citrate**	54965-24-1	citrate	Estrogen/progestogen Receptor,Autophagy	98.8	0.53	100.5	0.53	128.9	1.29	165.6	2.31
**Tandutinib (MLN518)**	387867-13-2	free base	FLT3	79.0	-0.31	87.5	0.05	120.0	0.92	97.1	0.14
**TAPI-1**	171235-71-5	free base	Others	79.1	-0.31	77.7	-0.30	99.1	0.03	122.0	0.93
**Tariquidar**	206873-63-4	free base	P-gp	64.1	-0.94	61.1	-0.91	96.7	-0.07	110.7	0.57
**Tasisulam**	519055-62-0	free base	Caspase	75.1	-0.48	93.8	0.28	96.9	-0.07	73.6	-0.61

**TCID**	30675-13-9	free base	DUB	79.2	-0.30	93.9	0.29			110.8	0.57
**TCS 359**	301305-73-7	free base	FLT3	98.8	0.53	68.7	-0.63	109.3	0.46	81.1	-0.37
**Telaprevir (VX-950)**	402957-28-2	free base	HCV Protease	112.4	1.10	115.5	1.07	111.4	0.55	87.0	-0.19
**Telatinib**	332012-40-5	free base	VEGFR,PDGFR,c-Kit	109.0	0.96	61.4	-0.90	98.2	-0.01	107.3	0.46
**Telmisartan**	144701-48-4	free base	RAAS	99.5	0.55	113.2	0.99	98.0	-0.02	96.2	0.11
**Telotristat Etiprate (LX 1606 Hippurate)**	11137608-69-5	Salt	Hydroxylase	103.8	0.74	54.5	-1.14	92.1	-0.27	84.7	-0.26
**Temocapril HCl**	110221-44-8	hydrochloride	RAAS	101.8	0.65	115.7	1.08	97.8	-0.03	131.0	1.21
**Temozolomide**	85622-93-1	free base	Autophagy	111.6	1.07	76.6	-0.34	108.2	0.41	85.8	-0.22
**Temsirolimus (CCI-779, NSC 683864)**	162635-04-3	free base	mTOR	65.6	-0.88	50.8	-1.28	59.6	-1.65	74.3	-0.59
**Tenofovir Disoproxil Fumarate**	202138-50-9	fumarate	Reverse Transcriptase	96.5	0.43	115.6	1.07	94.9	-0.15	78.1	-0.47
**Tenovin-1**	380315-80-0	free base	p53,E3 Ligase	82.0	-0.19	68.4	-0.64	103.7	0.22	105.1	0.39
**Tenovin-6**	1011557-82-6	free base	p53	77.5	-0.38	116.7	1.12	109.3	0.46	111.9	0.61
**Terazosin HCl**	70024-40-7	hydrochloride	Adrenergic Receptor	88.2	0.07	69.9	-0.58	99.8	0.06	117.5	0.78
**Tetracaine HCl**	136-47-0	hydrochloride	Calcium Channel	99.2	0.54	113.9	1.02	145.3	1.99	106.4	0.43
**Tetrahydrozoline HCl**	522-48-5	free base	Adrenergic Receptor	92.6	0.26	105.0	0.69	106.8	0.35	92.8	0.00
**Tetrandrine**	518-34-3	free base	Calcium Channel	114.1	1.17	113.0	0.98	88.3	-0.43	81.4	-0.36
**TG003**	300801-52-9	free base	CDK	71.6	-0.63	117.3	1.14	92.2	-0.27	67.5	-0.80
**TG100-115**	677297-51-7	free base	PI3K	88.2	0.08	51.3	-1.26	131.6	1.41	101.6	0.28
**TG100713**	925705-73-3	free base	PI3K	98.9	0.53	94.8	0.32	111.6	0.56	103.7	0.35
**TG101209**	936091-14-4	free base	JAK,FLT3,c-RET	51.7	-1.47	104.4	0.67	91.8	-0.28	52.7	-1.27
**TG101348 (SAR302503)**	936091-26-8	free base	JAK	42.8	-1.85	112.1	0.95	86.6	-0.50	54.7	-1.21
**TGX-221**	663619-89-4	free base	PI3K	88.6	0.09	75.1	-0.40	112.3	0.59	64.6	-0.89
**Thalidomide**	50-35-1	free base	TNF-alpha,E3 Ligase	97.4	0.47	101.7	0.57	106.0	0.32	104.7	0.38
**Thiazovivin**	1226056-71-8	free base	ROCK	94.6	0.35	68.1	-0.65	104.6	0.26	89.3	-0.11
**Tianeptine sodium**	30123-17-2	sodium	5-HT Receptor	97.8	0.48	49.1	-1.34	114.7	0.69	68.1	-0.78
**TIC10**	41276-02-2	free base	Akt	86.2	-0.01	63.0	-0.84			125.3	1.03
**Ticagrelor**	274693-27-5	free base	P2 Receptor	98.7	0.52	117.0	1.13	94.5	-0.17	102.3	0.30
**Tie2 kinase inhibitor**	948557-43-5	free base	Tie-2	111.7	1.07	82.8	-0.12	118.3	0.84	93.3	0.02

**Timolol Maleate**	26921-17-5	maleate	Adrenergic Receptor	86.3	0.00	117.5	1.15	93.0	-0.23	121.9	0.92
**Tiotropium Bromide hydrate**	139404-48-1	hydrate	AChR	81.0	-0.23	57.6	-1.03	118.8	0.86	124.7	1.01
**Tioxolone**	4991-65-5	free base	Carbonic Anhydrase	92.9	0.27	108.5	0.82	140.8	1.80	129.6	1.16
**Tipifarnib**	192185-72-1	free base	Transferase	70.3	-0.68	106.4	0.74	65.6	-1.40	53.2	-1.26
**Tivozanib (AV-951)**	475108-18-0	free base	VEGFR,PDGFR,c-Kit	80.0	-0.27	114.8	1.05	130.5	1.36	90.0	-0.09
**Tizanidine HCl**	64461-82-1	hydrochloride	Adrenergic Receptor	112.0	1.08	56.0	-1.09	130.1	1.34	79.5	-0.42
**TMP269**	1314890-29-3	free base	HDAC	86.1	-0.01	117.9	1.16	73.2	-1.07	77.0	-0.50
**Tofacitinib (CP-690550) Citrate**	540737-29-9	citrate	JAK	86.8	0.02	97.1	0.40	75.7	-0.97	123.7	0.98
**Tofacitinib (CP-690550,Tasocitinib)**	477600-75-2	free base	JAK	95.3	0.38	81.8	-0.15	94.3	-0.18	109.4	0.52
**Tolazoline HCl**	59-97-2	free base	Adrenergic Receptor	83.8	-0.11	117.7	1.15	76.4	-0.94	117.2	0.77
**Tolbutamide**	64-77-7	free base	Potassium Channel	121.1	1.47	67.2	-0.68	91.3	-0.30	118.6	0.82
**Tolcapone**	134308-13-7	free base	Transferase	88.5	0.09	117.8	1.16	101.0	0.11	120.6	0.88
**Tolfenamic Acid**	13710-19-5	free base	COX	89.9	0.15	68.1	-0.65	103.6	0.22	91.7	-0.03
**Tolterodine tartrate**	124937-52-6	tartrate	AChR	100.2	0.58	115.4	1.07	125.1	1.13	138.4	1.44
**Tolvaptan**	150683-30-0	free base	Vasopressin Receptor	78.9	-0.32	116.7	1.12	112.5	0.60	121.3	0.90
**Topiramate**	97240-79-4	free base	Carbonic Anhydrase	102.5	0.68	72.7	-0.48	107.1	0.37	71.8	-0.67
**Topotecan HCl**	119413-54-6	hydrochloride	Topoisomerase	47.1	-1.66	54.4	-1.15	95.5	-0.13	7.9	-2.69
**Torcetrapib**	262352-17-0	free base	CETP	98.9	0.53	90.0	0.15	98.7	0.01	101.9	0.29
**Toremifene Citrate**	89778-27-8	citrate	Estrogen/progestogen Receptor	125.7	1.66	98.1	0.44	121.2	0.97	104.1	0.36
**Torin 2**	1223001-51-1	free base	ATM/ATR,mTOR	40.2	-1.96	35.1	-1.85	57.7	-1.73	8.8	-2.66
**TPCA-1**	507475-17-4	free base	IκB/IKK	71.0	-0.65	114.5	1.04	119.5	0.89	96.2	0.11
**Trametinib (GSK1120212)**	871700-17-3	free base	MEK	72.1	-0.61	36.4	-1.80	60.2	-1.63	106.9	0.45
**Tranylcypromine (2-PCPA) HCl**	4548-34-9	hydrochloride	Histone demethylases	80.9	-0.23	62.7	-0.85	79.8	-0.79	100.3	0.24
**Trelagliptin**	865759-25-7	free base	DDP-4	83.8	-0.11	67.1	-0.69	79.2	-0.82	114.1	0.68
**Triamterene**	396-01-0	free base	Sodium Channel	93.2	0.29	102.8	0.61	99.5	0.04	109.5	0.53
**Triapine**	200933-27-3	free base	ribonucleotide reductase	78.3	-0.34	116.3	1.10	89.8	-0.37	25.7	-2.12
**Trichostatin A (TSA)**	58880-19-6	free base	HDAC	52.2	-1.45	47.0	-1.42	38.2	-2.56	2.0	-2.88
**Triciribine**	35943-35-2	free base	Akt	79.8	-0.28	116.5	1.11	118.7	0.86	52.8	-1.27

**Trifluoperazine 2HCl**	440-17-5	2HCl	Autophagy	96.9	0.45	117.4	1.14	132.5	1.44	135.3	1.35
**Triflusal**	322-79-2	free base	COX	91.6	0.22	118.0	1.16	93.7	-0.20	130.1	1.18
**Trilostane**	13647-35-3	free base	Dehydrogenase	78.0	-0.36	115.9	1.09	102.9	0.19	89.7	-0.10
**Trimebutine**	39133-31-8	free base	Opioid Receptor	101.8	0.65	115.3	1.06	95.2	-0.14	126.2	1.06
**Tripelennamine HCl**	154-69-8	hydrochloride	Histamine Receptor	87.3	0.04	118.3	1.17	115.0	0.70	108.0	0.48
**Tropicamide**	1508-75-4	free base	AChR	94.7	0.35	116.3	1.10	109.4	0.46	156.7	2.02
**Tropisetron**	105826-92-4	hydrochloride	5-HT Receptor	101.1	0.62	117.0	1.13	106.8	0.35	90.4	-0.08
**Trospium chloride**	10405-02-4	chloride	AChR	80.1	-0.27	96.8	0.39	122.0	1.00	92.9	0.00
**TSU-68 (SU6668, Orantinib)**	252916-29-3	free base	VEGFR,PDGFR,FGFR	100.6	0.60	67.0	-0.69	127.8	1.25	72.3	-0.65
**Tubacin**	537049-40-4	free base	HDAC	106.3	0.84	54.6	-1.14	77.6	-0.89	97.6	0.15
**Tubastatin A**	1252003-15-8	free base	HDAC	62.3	-1.02	61.7	-0.88	111.1	0.54	81.4	-0.36
**Tubastatin A HCl**	1310693-92-5	hydrochloride	HDAC	71.4	-0.64	115.7	1.08	107.6	0.39	103.3	0.33
**TW-37**	877877-35-5	free base	Bcl-2	103.5	0.72	106.1	0.73	109.2	0.46	85.6	-0.23
**TWS119**	601514-19-6	free base	GSK-3	102.4	0.68	115.6	1.08	101.7	0.14	83.3	-0.30
**Tyrphostin 9**	10537-47-0	free base	EGFR	49.9	-1.55	49.4	-1.33	109.2	0.46	93.4	0.02
**Tyrphostin AG 1296**	146535-11-7	free base	FGFR,c-Kit,PDGFR	88.9	0.11	70.8	-0.55	81.1	-0.74	96.2	0.11
**Tyrphostin AG 879**	148741-30-4	free base	HER2	73.5	-0.55	68.8	-0.63	102.3	0.16	109.2	0.52
**U0126-EtOH**	1173097-76-1	ethanolat	MEK	86.6	0.01	66.6	-0.71	73.5	-1.06	77.6	-0.48
**U-104**	178606-66-1	free base	Carbonic Anhydrase	84.3	-0.09	46.1	-1.45	102.9	0.19	100.8	0.25
**UK 383367**	348622-88-8	free base	Procollagen C Proteinase	104.4	0.76	88.9	0.11	90.5	-0.34	135.8	1.36
**UNC1215**	1415800-43-9	free base	Epigenetic Reader Domain	97.6	0.47	80.1	-0.21			98.3	0.17
**UNC1999**	1431612-23-5	free base	Histone Methyltransferase	77.9	-0.36	70.8	-0.55			111.4	0.59
**UNC2881**	1493764-08-1	free base	Others	75.9	-0.44	117.5	1.14	88.3	-0.43	79.4	-0.43
**UNC669**	1314241-44-5	free base	MBT	73.5	-0.55	116.8	1.12	96.4	-0.09	109.8	0.54
**UPF 1069**	1048371-03-4	free base	PARP	65.2	-0.90	85.7	-0.01	82.9	-0.66	76.5	-0.52
**Urapidil HCl**	64887-14-5	hydrochloride	5-HT Receptor	106.3	0.84	67.3	-0.68	100.2	0.07	146.7	1.71
**URB597**	546141-08-6	free base	FAAH	89.7	0.14	105.5	0.71	127.7	1.24	128.7	1.14
**Valdecoxib**	181695-72-7	free base	COX	101.2	0.63	114.7	1.04	104.6	0.26	120.9	0.89
**Valproic acid sodium salt (Sodium**	1069-66-5	free base	Autophagy,HDAC,GABA	79.8	-0.28	92.4	0.23	112.7	0.61	82.2	-0.33

**valproate)**			Receptor								
**Valsartan**	137862-53-4	free base	RAAS	101.2	0.63	116.8	1.12	101.7	0.14	83.8	-0.28
**Vandetanib (ZD6474)**	443913-73-3	free base	VEGFR	104.1	0.75	104.4	0.67	90.2	-0.35	111.9	0.61
**Vardenafil HCl Trihydrate**	224785-90-4	dydrate	PDE	97.4	0.47	51.8	-1.24	117.1	0.79	143.1	1.59
**Varespladib (LY315920)**	172732-68-2	free base	Phospholipase (e.g. PLA)	86.5	0.00	109.7	0.86	99.1	0.03	109.2	0.52
**Varlitinib**	845272-21-1	free base	EGFR	76.1	-0.44	113.5	1.00	112.9	0.61	151.7	1.87
**Vatalanib (PTK787) 2HCl**	212141-51-0	dihydrochlorid e	c-Kit,VEGFR	90.5	0.17	100.9	0.54	99.6	0.05	108.1	0.48
**WAY-100635 Maleate**	1092679-51-0	hydrochloride	5-HT Receptor	107.0	0.87	114.6	1.04	116.3	0.76	107.5	0.47
**WAY-600**	1062159-35-6	free base	mTOR	57.3	-1.23	113.5	1.00	91.0	-0.32	98.8	0.19
**VE-821**	1232410-49-9	free base	ATM/ATR	83.0	-0.15	56.8	-1.06	104.9	0.27	100.4	0.24
**VE-822**	1232416-25-9	free base	ATM/ATR	83.8	-0.11	45.3	-1.48			33.8	-1.87
**Veliparib (ABT-888)**	912444-00-9	free base	PARP	114.6	1.19	106.7	0.75	93.3	-0.22	51.0	-1.32
**Vemurafenib (PLX4032, RG7204)**	918504-65-1	free base	Raf	110.9	1.04	66.2	-0.72	184.8	3.67	86.8	-0.19
**Venlafaxine**	99300-78-4	hydrochloride	5-HT Receptor	76.3	-0.43	92.2	0.22	106.8	0.35	99.1	0.20
**VER-49009**	940289-57-6	free base	HSP90	48.1	-1.62	89.2	0.12	57.1	-1.76	73.4	-0.61
**VER-50589**	747413-08-7	free base	HSP (e.g. HSP90)	49.5	-1.57	36.3	-1.81	40.0	-2.48	50.7	-1.33
**WH-4-023**	837422-57-8	free base	Src	95.1	0.37	66.9	-0.70	109.5	0.47	111.2	0.58
**WHI-P154**	211555-04-3	free base	JAK,EGFR	80.7	-0.24	114.4	1.03	105.8	0.31	105.6	0.40
**WIKI4**	838818-26-1	free base	Wnt/beta-catenin	119.1	1.38	107.1	0.77	98.3	-0.01	89.8	-0.09
**Vildagliptin (LAF-237)**	274901-16-5	free base	DPP-4	103.5	0.72	119.3	1.21	112.9	0.61	125.5	1.04
**Vinorelbine Tartrate**	125317-39-7	Salt	Microtubule Associat	49.9	-1.55	51.4	-1.26	48.7	-2.11	23.9	-2.18
**Vismodegib (GDC-0449)**	879085-55-9	free base	Hedgehog/Smoothened	91.8	0.23	86.1	0.00	103.5	0.21	107.0	0.45
**Wnt-C59 (C59)**	1243243-89-1	free base	Wnt/beta-catenin	85.4	-0.04	73.5	-0.46	88.5	-0.42	117.3	0.78
**Wnt-C59 (C59)**	1243243-89-1	free base	Wnt/beta-catenin	92.7	0.26	62.6	-0.85	81.6	-0.72	119.7	0.85
**Volasertib (BI 6727)**	755038-65-4	free base	PLK	54.5	-1.35	43.5	-1.55	33.8	-2.75	6.1	-2.75
**Voreloxin (SNS-595)**	175414-77-4	free base	Topoisomerase	52.5	-1.44	71.9	-0.51	96.7	-0.07	66.5	-0.83
**Voriconazole**	137234-62-9	free base	P450 (e.g. CYP17)	71.6	-0.63	113.0	0.98	100.5	0.08	93.5	0.02
**Vorinostat (SAHA, MK0683)**	149647-78-9	free base	Autophagy,HDAC	71.2	-0.64	68.1	-0.65	92.3	-0.26	14.5	-2.48

**Vortioxetine (Lu AA21004) HBr**	960203-27-4	hydrobromide	5-HT Receptor	76.3	-0.43	62.3	-0.86	102.4	0.17	138.8	1.46
**Wortmannin**	19545-26-7	free base	Autophagy,ATM/ATR,PI3K	85.4	-0.04	107.7	0.79	127.8	1.25	123.1	0.96
**WP1066**	857064-38-1	free base	JAK	96.0	0.41	102.5	0.60	101.5	0.13	102.1	0.30
**VS-5584 (SB2343)**	1246560-33-7	free base	PI3K	35.0	-2.18	43.7	-1.54	45.5	-2.25	48.2	-1.41
**VU 0357121**	433967-28-3	free base	GluR	96.5	0.43	91.4	0.19	103.5	0.22	107.8	0.48
**VU 0361737**	1161205-04-4	free base	GluR	108.4	0.93	114.5	1.04	109.5	0.47	105.9	0.41
**VU 0364439**	1246086-78-1	free base	GluR	101.9	0.66	63.9	-0.80	103.4	0.21	115.9	0.73
**VU 0364770**	61350-00-3	free base	GluR	83.8	-0.11	113.5	1.00	124.5	1.11	113.4	0.65
**VUF 10166**	155584-74-0	free base	5-HT Receptor	88.6	0.09	114.4	1.03	107.2	0.37	113.0	0.64
**VX-222 (VCH-222, Lomibuvir)**	1026785-59-0	free base	HCV Protease	101.2	0.63	110.5	0.89	110.6	0.51	86.9	-0.19
**VX-661**	1152311-62-0	free base	CFTR	79.1	-0.31	74.5	-0.42	105.6	0.30	107.1	0.45
**VX-680 (Tozasertib, MK-0457)**	639089-54-6	free base	Aurora Kinase	62.0	-1.03	62.2	-0.87	46.2	-2.22	76.1	-0.53
**VX-702**	745833-23-2	free base	p38 MAPK	91.8	0.23	56.2	-1.08	81.0	-0.74	88.8	-0.13
**VX-745**	209410-46-8	free base	p38 MAPK	72.7	-0.58	112.3	0.95	95.3	-0.14	83.9	-0.28
**VX-765**	273404-37-8,851091-96-8	free base	Caspase	104.7	0.78	54.7	-1.14	93.3	-0.22	91.0	-0.06
**VX-809 (Lumacaftor)**	936727-05-8	free base	CFTR	116.1	1.26	101.5	0.56	111.8	0.57	77.1	-0.50
**WY-14643 (Pirinixic Acid)**	50892-23-4	free base	PPAR	81.9	-0.19	63.8	-0.81	104.3	0.25	129.9	1.18
**WYE-125132 (WYE-132)**	1144068-46-1	free base	mTOR	56.2	-1.28	70.6	-0.56	41.7	-2.41	39.9	-1.68
**WYE-354**	1062169-56-5	free base	mTOR	92.2	0.25	112.3	0.96	98.8	0.01	74.3	-0.59
**WZ3146**	1214265-56-1	free base	EGFR	102.5	0.68	89.2	0.12	91.7	-0.29	60.6	-1.02
**WZ4002**	1213269-23-8	free base	EGFR	117.3	1.31	79.2	-0.25	107.6	0.39	95.8	0.09
**WZ4003**	1214265-58-3	free base	AMPK	123.7	1.58	78.5	-0.27	104.4	0.25	100.1	0.23
**WZ8040**	1214265-57-2	free base	EGFR	97.1	0.45	75.5	-0.38	122.9	1.04	79.1	-0.44
**WZ811**	55778-02-4	free base	CXCR	75.3	-0.47	58.5	-1.00	101.8	0.14	108.6	0.50
**XAV-939**	284028-89-3	free base	Wnt/beta-catenin	93.5	0.30	69.2	-0.61	107.8	0.40	91.3	-0.05
**XL019**	945755-56-6	free base	JAK	76.0	-0.44	112.4	0.96	87.9	-0.45	110.9	0.57
**XL019**	945755-56-6	free base	JAK	68.7	-0.75	60.9	-0.91	86.6	-0.50	118.6	0.82
**XL147**	956958-53-5	free base	PI3K	116.1	1.26	115.1	1.06	108.0	0.40	85.0	-0.25

**XL335**	629664-81-9	free base	FXR	85.2	-0.05	114.6	1.04	114.9	0.70	107.3	0.46
**XL888**	1149705-71-4	free base	HSP (e.g. HSP90)	45.1	-1.75	30.8	-2.01			40.1	-1.67
**XMD8-92**	1234480-50-2	free base	ERK	68.8	-0.75	75.8	-0.37	94.4	-0.17	73.3	-0.62
**Xylazine HCl**	23076-35-9	hydrochloride	Adrenergic Receptor	91.8	0.23	115.5	1.07	144.9	1.97	131.2	1.22
**Y-27632 2HCl**	129830-38-2	dihydrochloric	Autophagy,ROCK	112.7	1.11	107.7	0.79	74.4	-1.02	118.9	0.83
**YH239-EE**	1364488-67-4	free base	Mdm2	126.0	1.68	107.4	0.78	108.8	0.44	84.7	-0.26
**YM155 (Sepantronium Bromide)**	781661-94-7	bromide	Survivin	35.2	-2.17	1.9	-3.06	2.1	-4.09	0.4	-2.93
**YM201636**	371942-69-7	free base	PI3K	89.9	0.15	90.5	0.16	95.6	-0.12	74.5	-0.58
**YO-01027**	209984-56-5	free base	Gamma-secretase	87.8	0.06	55.8	-1.10	138.9	1.72	116.9	0.76
**Zalcitabine**	7481-89-2	free base	Reverse Transcriptase	125.8	1.67	109.4	0.85	97.5	-0.04	95.3	0.08
**Zaltoprofen**	74711-43-6	free base	COX	130.8	1.88	115.4	1.07	112.6	0.60	121.9	0.92
**ZCL278**	587841-73-4	free base	Rac	91.4	0.21	116.7	1.12	109.5	0.47	107.8	0.47
**Zebularine**	3690-10-6	free base	DNA Methyltransferase	70.3	-0.68	52.3	-1.22			94.1	0.04
**Z-FA-FMK**	197855-65-5	free base	Cysteine Protease	136.5	2.12	106.8	0.76	115.0	0.70	106.5	0.43
**Zibotentan (ZD4054)**	186497-07-4	free base	Endothelin Receptor	105.0	0.79	112.8	0.97	108.9	0.44	67.1	-0.81
**Zidovudine**	30516-87-1	free base	Reverse Transcriptase	109.8	0.99	116.3	1.10	114.4	0.68	94.5	0.05
**Zinc Pyrithione**	13463-41-7	free base	Proton Pump	118.8	1.37	109.4	0.85	109.8	0.48	62.1	-0.97
**ZM 306416**	690206-97-4	free base	VEGFR	81.1	-0.22	114.8	1.05	98.0	-0.02	115.4	0.72
**ZM 323881 HCl**	193000-39-4	hydrochloride	VEGFR	96.0	0.41	114.0	1.02	95.5	-0.12	94.6	0.06
**ZM 336372**	208260-29-1	free base	Raf	72.6	-0.58	74.2	-0.43	111.2	0.54	128.3	1.13
**ZM 39923 HCl**	1021868-92-7	hydrochloride	JAK	60.3	-1.11	61.8	-0.88	92.9	-0.24	79.1	-0.44
**ZM 447439**	331771-20-1	free base	Aurora Kinase	100.5	0.60	77.3	-0.32	74.5	-1.02	93.8	0.03
**Zolmitriptan**	139264-17-8	free base	5-HT Receptor	87.6	0.05	115.2	1.06	97.6	-0.03	94.4	0.05
**Zosuquidar (LY335979) 3HCl**	167465-36-3	hydrochloride	P-gp	84.7	-0.07	112.2	0.95	125.6	1.15	90.4	-0.08
**Zotarolimus(ABT-578)**	221877-54-9	free base	mTOR	63.7	-0.96	81.6	-0.16			71.5	-0.68
**ZSTK474**	475110-96-4	free base	PI3K	51.6	-1.48	94.9	0.32	63.0	-1.51	90.4	-0.08
**Z-VAD-FMK**	187389-52-2	free base	Caspase	92.5	0.26	66.1	-0.72	79.1	-0.82	99.9	0.22
