# Supplementary material for: The neuroendocrine phenotype, genomic profile and therapeutic sensitivity of GEPNET cell lines
Source: Endocr Relat Cancer. 2018 Jan 15;25(3):367–80. doi: 10.1530/ERC-17-0445 (PMC5827037; doi:10.1530/ERC-17-0445)
Supplement: Supplementary Table 6 [file erc-25-309-t006.pdf]

Supplementary Table 6. Clinical data of the origin of primary cultures.

**Primary culture**	**Gender**	**Age**	**Tumour location**	**Stage**	**Grade**	**Syndome**
SI-NET #1	Male	77	Lymph node metastasis	pT3	Grade 1	Carcinoid syndrome
SI-NET #2	Female	69	Liver metastasis	pT3N1M1	Grade 1	None
PanNET #1	Female	41	Primary tumour	pT4	Grade 2	Gastrinoma
PanNET #2	Male	61	Primary tumour	pT3	Grade 1	Non-functioning
